# Health effects associated with alcohol consumption: a Burden of Proof study

**DOI:** 10.1038/s44360-026-00139-5

**Published:** 2026-06-01

**Authors:** Xiaochen Dai, Sneha I. Nicholson, Hilary R. Lawlor, Sinclair Carr, Jaimie D. Steinmetz, Natalie M. Chen, Susan A. McLaughlin, Simon I. Hay, Kanyin L. Ong, Aleksandr Y. Aravkin, Peng Zheng, Reed J. D. Sorensen, Nora M. Gilbertson, Erin C. Mullany, Erin M. O’Connell, Christopher J. L. Murray, Emmanuela Gakidou

**Affiliations:** 1 Institute for Health Metrics and Evaluation, University of Washington, Seattle, WA USA; 2 Department of Epidemiology, Harvard T.H. Chan School of Public Health, Boston, MA USA; 3 Department of Health Metrics Sciences, School of Medicine, University of Washington, Seattle, WA USA; 4 Department of Applied Mathematics, University of Washington, Seattle, WA USA

**Keywords:** Risk factors, Diseases

## Abstract

The relationship between alcohol and health is complex, and the evidence relating alcohol consumption to various cardiovascular diseases, cancers and other conditions is evolving. Moreover, alcohol drinking guidelines vary widely. Here we conducted 16 systematic reviews across four databases and conservatively re-evaluated dose–response relationships between alcohol consumption and 20 health outcomes, analysing 843 cohort and case–control studies using the Burden of Proof meta-analytic framework. We found that levels of current alcohol consumption are associated with increased risks for cancers of the breast, colorectum, oesophagus, larynx, lip and oral cavities, pharynx, liver, stomach, pancreas and prostate, as well as pancreatitis, cirrhosis and other chronic liver diseases, lower respiratory infections, tuberculosis, and atrial fibrillation and flutter. We found J- or U-shaped relationships between alcohol consumption and type 2 diabetes, Alzheimer’s disease and other dementias, ischaemic heart disease, ischaemic stroke and haemorrhagic stroke. While potential health impacts at low-to-moderate levels varied by outcome, high levels of alcohol consumption were associated with increased risk across all outcomes.

## Abstract (web summary)

A meta-analysis using the Burden of Proof framework, drawing on 16 systematic reviews and 843 observational studies, finds that alcohol consumption increases risk for most health outcomes, with high intake uniformly harmful for all tested outcomes and only limited, outcome-specific U-shaped associations at low-to-moderate levels for some cardiometabolic and neurological conditions.

## Main

Nearly half of the global population aged 15+ years regularly consumes alcohol^1^. Thus, understanding the totality of health impacts of alcohol consumption is crucial for guiding personal lifestyle choices, clinical practices and public health policies aimed at reducing alcohol-attributable disease burden. National and international drinking guidelines differ considerably^2,3^, leaving individuals unclear on what level of intake, if any, can be considered safe.

Discrepancies between guidelines reflect the complex and differential relationships between alcohol and health outcomes, modified by factors such as drinking volume, intake patterns (for example, heavy episodic drinking) and type of beverage consumed^4^. Meta-analyses of observational studies have consistently demonstrated that even low levels of alcohol intake are associated with increased risks of several cancers^5,6^ and liver disease^7,8^, with escalating risk as intake increases. Conversely, low-to-moderate consumption (generally up to two standard drinks or 20 g of pure alcohol per day) has been associated with reduced risk of cardiovascular disease^9,10^, type 2 diabetes^11,12^ and dementia^13,14^. However, these associations attenuate or reverse at higher intake.

Despite the breadth of research into alcohol consumption and its associated health consequences, existing meta-analyses have several limitations. It is common for analyses to focus on individual diseases or disease groups^5,9,15^; however, this limits the comparability of risk across outcomes. While it is widely recognized that many alcohol–outcome relationships have dose–response patterns, meta-analyses often only compare risks relative to a single reference group that is common to included studies, typically non-drinkers, sometimes combined with occasional drinkers^5,16^. This approach can introduce bias, as lifetime abstainers may avoid alcohol due to pre-existing health conditions^17,18^, and former drinkers may have stopped drinking due to illness^19,20^. Studies that compare risks between current drinkers at varying intake levels are therefore often systematically excluded. Furthermore, previous meta-analyses often do not adequately account for between-study heterogeneity or adjust for potential sources of bias when estimating relative risks (RRs) and their uncertainties, thereby limiting the ability to reflect the varying quality of the included studies accurately. These methodological constraints impede a comprehensive and accurate assessment of alcohol’s health risks, which is essential for informing individual and public health decisions.

As part of the Global Burden of Diseases, Risk Factors, and Injuries Study (GBD) 2023, the present study attempts to address these challenges by applying the Burden of Proof meta-analytic framework^21^ to systematically re-evaluate the dose–response relationships between alcohol consumption and 20 health outcomes, considering all available data from cohort and case–control studies published through 2023. Outcomes evaluated in this study were selected based on existing evidence of a relationship with alcohol and included ten cancers (breast, colorectal, oesophageal, laryngeal, liver, lip and oral, pharyngeal, pancreatic, prostate and stomach), four cardiovascular diseases (atrial fibrillation and flutter, ischaemic stroke, haemorrhagic stroke and ischaemic heart disease) and six other conditions (Alzheimer’s disease and other dementias, cirrhosis and other chronic liver diseases, lower respiratory infections, pancreatitis, tuberculosis and type 2 diabetes). Definitions of the outcomes are provided in Extended Data Table 1.

The Burden of Proof approach uses a six-step meta-analytic framework to objectively and comparatively quantify the strength of evidence linking risk factors to health outcomes^22–29^. We conducted a systematic review for each alcohol–outcome pair, except for the upper aerodigestive tract cancers (that is, oesophageal, laryngeal, lip and oral cavity, and pharyngeal cancers) and ischaemic or haemorrhagic strokes, which were each grouped together as combined systematic reviews, resulting in a total of 16 systematic reviews. We identified all relevant cohort and case–control studies indexed in PubMed, Embase, CINAHL and Web of Science through 31 December 2023. We extracted relevant data on RRs of each outcome associated with alcohol use, study characteristics and bias covariates, following a pre-established template (Supplementary Table 8). Using meta-regression–Bayesian, regularized, trimmed (MR-BRT)^30^, we estimated RR functions for each alcohol–outcome relationship, relative to no consumption, with a spline ensemble, systematically trimming outliers, accounting for varying exposure intervals between comparison groups, adjusting for potential biases arising from known study-design characteristics, and incorporating remaining ‘unexplained’ between-study heterogeneity into uncertainty estimates while accounting for small numbers of studies. For each significant association, a burden-of-proof risk function (BPRF) was further derived as the 5th (harmful) or 95th (protective) quantile of the dose–response relationship, accounting for between-study heterogeneity. The BPRF represents a conservative interpretation of the evidence, providing the smallest excess or reduced risk associated with alcohol consumption consistent with the data. To facilitate comparisons across outcomes, we calculated an average BPRF between the 15th and 85th exposure quantiles and converted it into a risk–outcome score (ROS), which was then mapped to a zero-star to five-star rating, with zero stars indicating no evidence of an association and five stars suggesting strong and consistent evidence.

A summary of the main findings, limitations and policy implications is presented in Table 1.

**Table 1:** Policy summary

Background	The relationship between alcohol consumption and health risks is controversial. Conflicting research has led to a confusing field of information for both policymakers and individual consumers. This study aimed to systematically review and comprehensively assess the RR of alcohol consumption across 20 health outcomes.
Main findings and limitations	We found alcohol use to be associated with higher RRs for cancers of the breast, colorectum, oesophagus, larynx, lip and oral cavities, pharynx, liver, stomach, pancreas, and prostate, pancreatitis, cirrhosis and other chronic liver diseases, lower respiratory infections, tuberculosis and atrial fibrillation and flutter. In contrast, alcohol consumption showed J-shaped or U-shaped relationships with type 2 diabetes, Alzheimer’s disease and other dementias, ischaemic heart disease, ischaemic stroke and haemorrhagic stroke. This study is limited first by the quality of the input data from observational studies, which can be prone to bias from unmeasured confounding. Due to a paucity of relevant data, we were unable to estimate risk relationships stratified by beverage type or pattern of consumption (for example, heavy episodic drinking), which may display differential relationships with health risks.
Policy implications	While low-to-moderate intake is modestly associated with lower risks of certain cardiovascular diseases, type 2 diabetes, and Alzheimer’s disease and other dementias, these associations are observational, uncertain and possibly biased by residual confounding. Any potential benefit must be weighed against the well-established harmful effects of alcohol use for most health outcomes, including increased cancer risk even at very low levels of consumption. Policymakers should base drinking guidelines on the totality of evidence across health outcomes, move beyond arbitrary thresholds, and prioritize clear, evidence-based messaging that promotes population-level understanding of alcohol-related risks.

## Results

This study evaluates RR functions and BPRFs for 20 health outcomes associated with alcohol consumption (Table 2) following PRISMA guidelines^31^, based on the most recent high-quality systematic review (Supplementary Table 7). Meta-analyses were based on 843 cohort and case–control studies published between 1961 and 2023, including studies identified before this study (Supplementary Fig. 1). Detailed study characteristics—including design, sample size, follow-up duration, adjusted confounders and bias covariates—are reported in Supplementary Tables 9–11.

Publication bias was assessed using Egger’s regression, and identified for eight outcomes: laryngeal cancer, lip and oral cavity cancer, Alzheimer’s disease and other dementias, atrial fibrillation and flutter, type 2 diabetes, pancreatic cancer, haemorrhagic stroke and ischaemic heart disease. All analyses adjusted for significant bias covariates reflecting study-design characteristics (Supplementary Tables 13 and 14), and remaining between-study heterogeneity was incorporated into uncertainty estimates. Additional details on candidate bias covariates, bias adjustments and heterogeneity estimates are provided in the Supplementary Information (sections VIII and X).

### Five-star associations

A harmful five-star association is assigned when the BPRF suggests that average alcohol consumption (15th–85th percentiles) increases risk by more than 85% (ROS > 0.62). Among the 20 outcomes evaluated, only other pharyngeal cancer met this criterion.

**Fig. 1:**
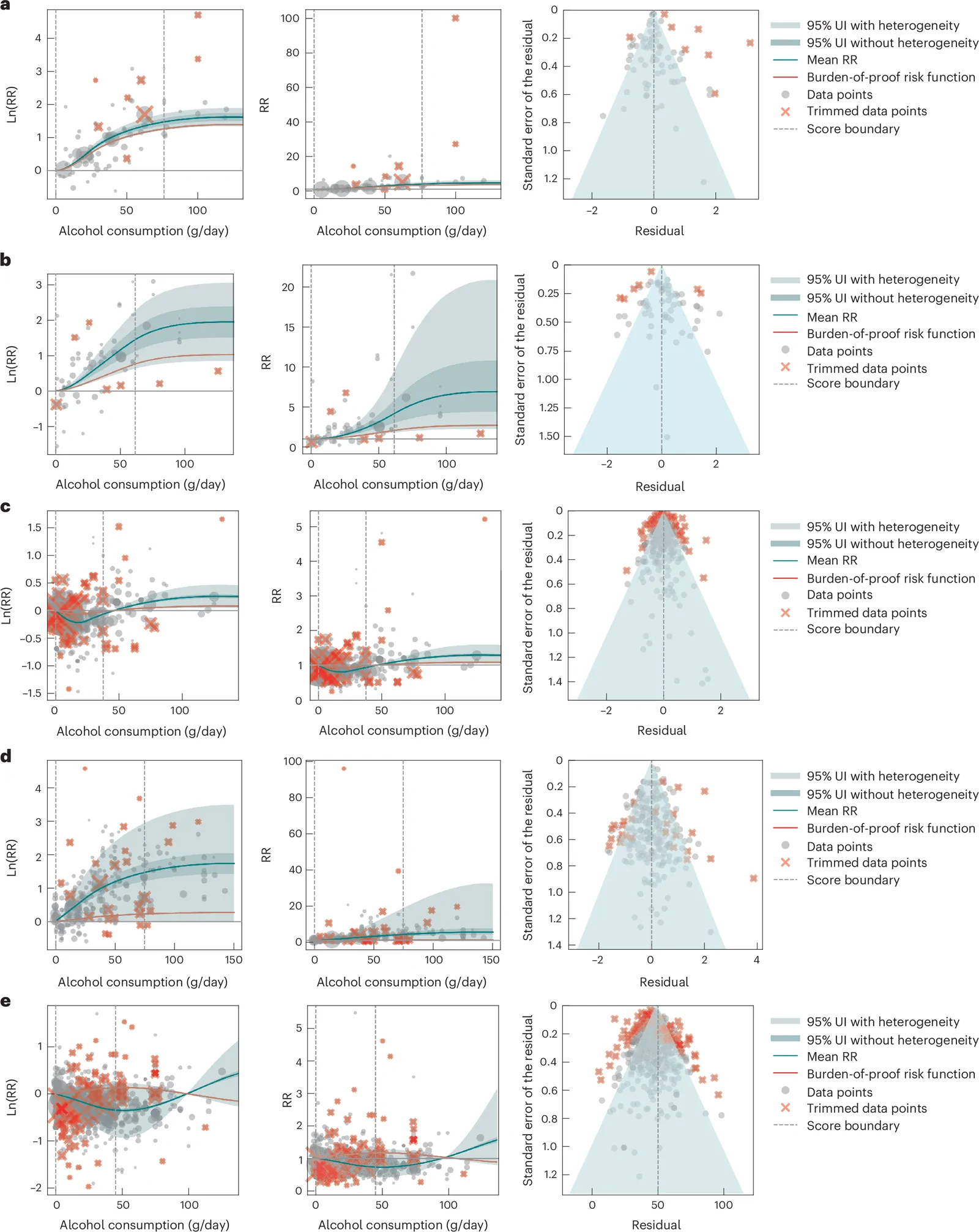
Risk–outcome-specific risk curves and funnel plots. **a**, Alcohol consumption and other pharyngeal cancer. **b**, Alcohol consumption and cirrhosis and other chronic liver diseases. **c**, Alcohol consumption and type 2 diabetes. **d**, Alcohol consumption and oesophageal cancer. **e**, Alcohol consumption and ischaemic heart disease. The risk curves are computed relative to no alcohol consumption. In the left and middle plots, the dark line indicates the mean RR across alcohol consumption levels (in grams per day); the light and dark shading show 95% UIs with and without between-study heterogeneity, respectively, with the red line highlighting the burden-of-proof function; the size of the data points corresponds to the inverse of the standard error, with those trimmed during the model fitting process marked by a red x; and the dashed lines represent the 15th percentile of the reference exposure and the 85th percentile of the alternative exposure. To visualize log-RR points in the plots on the left, we plotted each data point with the *x* value at the midpoint of the alternative group and the *y*-value offset by the difference between the reported and predicted log risk. In the middle plots, we exponentiated the *y* values from the plots on the left to yield the RR curve. Shown on the right are customized funnel plots, with the *x* axis representing residuals between predicted and observed RRs, and the *y* axis representing uncertainty from both measurement error and between-study heterogeneity.

The dose–response relationship was estimated using 78 observations from 3 cohort and 20 case–control studies across eight locations (Supplementary Tables 9 and 10)^32–54^. Alcohol exposure ranged from 0 g to 120 g per day, with the 85th percentile at 76 g per day (approximately 7.5 standard drinks; Fig. 1a). Alcohol consumption was strongly associated with increased risk of other pharyngeal cancer (Fig. 1a). Mean RR estimates were 1.16 (95% uncertainty interval (UI): 1.13–1.18) at 10 g per day, 1.56 (1.45–1.68) at 20 g per day and 2.73 (2.30–3.23) at 40 g per day; at 76 g per day, mean RR reached 4.24 (3.33–5.40). The BPRF suggested that alcohol consumption within the typical exposure range was associated with at least a 105% increase in risk (ROS = 0.72). The estimated dose–response curve was non-linear, with risk increasing steeply at lower intake levels and levelling off at higher exposures.

No bias covariates were identified by the MR-BRT algorithm for this outcome (Supplementary Tables 13 and 14). The meta-analytic model accounted for study-design differences and remaining between-study heterogeneity and showed good agreement with the observed data (Fig. 1a). After trimming 10% of observations, no evidence of publication bias was detected. Additional study details are provided in Supplementary Tables 9–11, and results without trimming are shown in Supplementary Fig. 3.10.

### Four-star associations

Four-star associations correspond to BPRF estimates indicating a 50–85% increase in risk (ROS > 0.41–0.62). No outcomes met this threshold in the present analysis.

### Three-star associations

A three-star association is assigned when the BPRF suggests that alcohol consumption increases risk by 15–50% (ROS > 0.14–0.41). Five outcomes met this criterion: laryngeal cancer (at least 49% higher risk; ROS 0.40), cirrhosis and other chronic liver diseases ( ≥ 40%; 0.34), pancreatitis, colon and rectal cancer, and lip and oral cavity cancer (each ≥22%; 0.20).

As an exemplar, we evaluated alcohol consumption and cirrhosis and other chronic liver diseases using 67 observations from 13 cohort and 1 case–control study across six locations (Supplementary Tables 9 and 10)^55–68^. Alcohol exposure ranged from 0 g to 180 g per day, with the 85th percentile at 61 g per day. Alcohol consumption showed a moderately harmful association with risk of cirrhosis and other chronic liver diseases (Fig. 1b). Mean RR estimates were 1.12 (95% UI: 1.05–1.19) at 10 g per day, 1.40 (1.16–1.69) at 20 g per day and 2.43 (1.47–4.03) at 40 g per day; at 61 g per day the mean RR reached 4.25 (1.87–9.66). The BPRF suggested at least a 40% increase in risk (ROS = 0.34). The dose–response relationship was non-linear, with risk rising rapidly at lower intake levels and attenuating at higher exposures (Fig. 1b).

Observations lacking adjustment for age or reporting incidence rather than mortality were adjusted as bias covariates (Supplementary Tables 13 and 14). Substantial between-study heterogeneity remained after adjustment. The meta-analytic model accounted for study-design differences and heterogeneity and fit the data well (Fig. 1b). After trimming 10% of observations, no evidence of publication bias was detected. Additional study details are provided in Supplementary Tables 9–11; results without trimming are shown in Supplementary Fig. 3.03.

**Extended Data Fig. 2:**
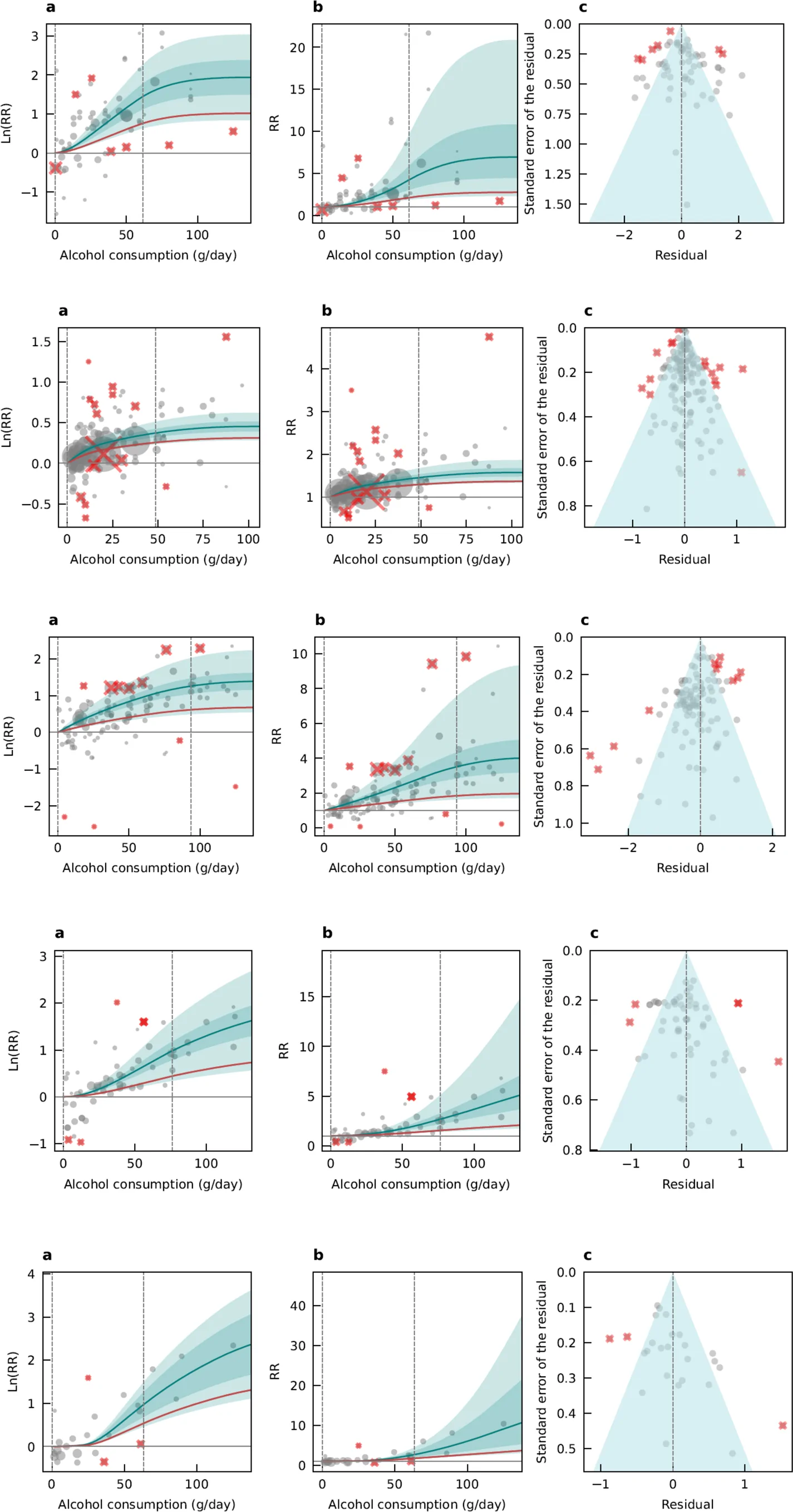
3-Star Relative Risk Curves. From top (1) to bottom (5), the plots correspond to the following outcomes: “Cirrhosis and other chronic liver diseases”, “Colorectal cancer”, “Laryngeal cancer”, “Lip and oral cavity cancer”, and “Pancreatitis”. The following risk curves are computed relative to no alcohol consumption. In panels (**a**) and (**b**), the dark line indicates mean relative risk across alcohol consumption levels (in g/day); the light and dark shading show 95% uncertainty intervals with and without between-study heterogeneity, respectively, with the red line highlighting the burden of proof function; the size of the data points corresponds to the inverse of the standard error, with those trimmed during the model fitting process marked by a red x; and the dashed lines represent the 15th percentile of the reference exposure and the 85th percentile of the alternative exposure. To visualize log-relative- risk points in panel (**a**), we plotted each data point with the x-value at the midpoint of the alternative group and the y-value offset by the difference between the reported and predicted log risk. In panel (**b**), we exponentiated the y-values from panel (**a**) to yield the relative risk curve. Panel (**c**) depicts a customized funnel plot, with the x-axis representing residuals between predicted and observed relative risks, and the y-axis representing uncertainty from both measurement error and between-study heterogeneity. RR relative risk, UI uncertainty interval.

Results for other outcomes with three-star associations are summarized in Tables 2 and 3, Extended Data Fig. 2 and Extended Data Table 2.

**Table 2:** Strength of the evidence for the relationship between alcohol consumption (grams per day) and the 20 health outcomes analysed

Health outcome	85th percentile risk level	RR (95% UI) at 85th percentile risk level	Exposure-averaged BPRF	Conservative interpretation of the average risk increase/decrease	ROS	Star rating	Publication bias	No. of studies	Selected bias covariates
Other pharyngeal cancer	76.25	4.24 (3.33, 5.4)	2.05	105.4%	0.72	⭐⭐⭐⭐⭐	No	23	None
Laryngeal cancer	93.44	3.51 (1.63, 7.56)	1.49	49.2%	0.4	⭐⭐⭐	Yes	30	Adjusted for age and whether the outcome is not only incidence (that is, incidence + mortality combined)
Cirrhosis and other chronic liver diseases	61.49	4.25 (1.87, 9.66)	1.40	40.5%	0.34	⭐⭐⭐	No	14	Adjusted for age and whether outcome is not only incidence (that is, incidence + mortality combined)
Pancreatitis	63.31	2.65 (1.58, 4.45)	1.22	22.1%	0.2	⭐⭐⭐	No	7	None
Colon and rectal cancer	48.81	1.45 (1.26, 1.67)	1.22	22.1%	0.2	⭐⭐⭐	No	38	Adjusted for whether the outcome is not only incidence (that is, incidence + mortality combined)
Lip and oral cavity cancer	76.25	2.68 (1.41, 5.09)	1.22	22.1%	0.2	⭐⭐⭐	Yes	19	None
Oesophageal cancer	74.72	4.32 (0.99, 18.83)	1.15	15.0%	0.14	⭐⭐	No	66	Adjusted for age
Breast cancer	29.39	1.33 (1.19, 1.47)	1.12	11.6%	0.11	⭐⭐	No	68	None
Alzheimer’s disease and other dementias	57.44	1.00 (0.99, 1.00)	0.94	-6.4%	0.07	⭐⭐	Yes	24	None
Atrial fibrillation and flutter	38.21	1.27 (1.13, 1.43)	1.06	6.2%	0.06	⭐⭐	Yes	32	Adjusted for age and whether outcome is not only incidence (that is, incidence + mortality combined)
Type 2 diabetes	37.50	0.93 (0.88, 0.99)	0.96	-4.5%	0.05	⭐⭐	Yes	83	None
Liver cancer	80.50	2.26 (0.95, 5.34)	1.03	3.0%	0.03	⭐⭐	No	59	Adjusted for age and whether outcome is not only incidence (that is, incidence + mortality combined)
Pancreatic cancer	50.29	1.18 (1.03, 1.35)	1.03	3.0%	0.03	⭐⭐	Yes	16	None
Lower respiratory infections	62.48	1.39 (1.03, 1.86)	1.02	2.0%	0.02	⭐⭐	No	7	None
Prostate cancer	45.18	1.12 (1, 1.25)	1.01	1.0%	0.01	⭐⭐	No	25	None
Stomach cancer	50.00	1.15 (0.97, 1.36)	NA	NA	−6 × 10^−4^	⭐	No	35	None
Haemorrhagic stroke	49.99	1.3 (0.94, 1.79)	NA	NA	−0.005	⭐	Yes	57	Adjusted for sex
Ischaemic stroke	45.00	1.17 (0.95, 1.45)	NA	NA	−0.01	⭐	No	67	None
Tuberculosis	52.50	3.13 (0.77, 12.79)	NA	NA	−0.02	⭐	No	9	Adjusted for age and whether the population was unrepresentative of the study location
Ischaemic heart disease	44.95	0.71 (0.4, 1.24)	NA	NA	−0.07	⭐	Yes	164	None

The reported RR and its 95% UI reflect the risk of developing each outcome of interest when comparing levels of current alcohol use versus no alcohol use after accounting for between-study heterogeneity. The BPRF is calculated for risk–outcome pairs that were found to have significant relationships at a 0.05 level of significance when not incorporating between-study heterogeneity (that is, the lower bound of the conventional 95% UI does not cross the null RR value of one). The BPRF corresponds to the 5th quantile estimate of the RR accounting for between-study heterogeneity closest to the null for each risk–outcome pair, and it reflects the most conservative estimate of increased (for harmful effects) or decreased (for protective effects) risk associated with high alcohol consumption that is consistent with the available data. Negative ROSs indicate that the evidence of the association is very weak and inconsistent. For ease of interpretation, we have transformed the ROS and BPRF into a star rating (0–5) with a higher rating representing a larger effect with stronger evidence. The potential existence of publication bias, which, if present, would affect the validity of the results, was tested using Egger’s regression. Included studies represent all available relevant data identified through our systematic reviews. The selected bias covariates were chosen for inclusion in the model using an algorithm that systematically detects bias covariates that correspond to significant sources of bias in the observations included. If selected, the observations were adjusted to better reflect the gold-standard values of the covariate. See the Supplementary Information for more information about the candidate bias covariates that were selected within each model. NA, not applicable.

**Table 3:** RRs of alcohol consumption at intervals of one standard drink (10 g of alcohol per day) for each health outcome

Health outcome		Number of standard drinks									
		1	2	3	4	5	6	7	8	9	10
**Cancers**	Breast cancer	1.13 (1.08, 1.19)	1.25 (1.15, 1.35)	1.33 (1.2, 1.48)	1.4 (1.23, 1.58)	1.44 (1.26, 1.64)	1.46 (1.27, 1.68)	1.47 (1.27, 1.69)	1.48 (1.28, 1.71)	1.49 (1.28, 1.72)	1.49 (1.29, 1.73)
	Colon and rectal cancer	1.17 (1.1, 1.24)	1.28 (1.17, 1.4)	1.35 (1.21, 1.51)	1.41 (1.24, 1.60)	1.46 (1.27, 1.68)	1.5 (1.29, 1.75)	1.53 (1.31, 1.8)	1.56 (1.32, 1.84)	1.57 (1.33, 1.86)	1.57 (1.33, 1.87)
	Oesophageal cancer	1.32 (1.0, 1.75)	1.74 (1.0, 3.05)	2.26 (1.0, 5.14)	2.79 (0.99, 7.80)	3.28 (0.99, 10.8)	3.73 (0.99, 14.01)	4.14 (0.99, 17.3)	4.51 (0.99, 20.52)	4.83 (0.99, 23.55)	5.11 (0.99, 26.27)
	Laryngeal cancer	1.23 (1.09, 1.4)	1.48 (1.17, 1.88)	1.74 (1.24, 2.45)	2.02 (1.32, 3.10)	2.31 (1.39, 3.84)	2.61 (1.46, 4.69)	2.92 (1.52, 5.62)	3.20 (1.58, 6.51)	3.44 (1.62, 7.31)	3.64 (1.66, 7.99)
	Lip and oral cavity cancer	1.03 (1.01, 1.05)	1.11 (1.04, 1.2)	1.26 (1.08, 1.46)	1.46 (1.14, 1.87)	1.73 (1.21, 2.47)	2.05 (1.28, 3.28)	2.43 (1.36, 4.34)	2.83 (1.43, 5.58)	3.24 (1.5, 7)	3.68 (1.57, 8.62)
	Liver cancer	1.02 (1.0, 1.04)	1.07 (1.0, 1.15)	1.17 (0.99, 1.37)	1.3 (0.98, 1.71)	1.48 (0.98, 2.24)	1.71 (0.97, 3.01)	1.98 (0.96, 4.1)	2.24 (0.95, 5.29)	2.46 (0.95, 6.41)	2.64 (0.94, 7.38)
	Pancreatic cancer	1.05 (1.01, 1.1)	1.1 (1.02, 1.19)	1.13 (1.03, 1.26)	1.16 (1.03, 1.31)	1.18 (1.03, 1.34)	1.19 (1.03, 1.36)	1.2 (1.04, 1.38)	1.2 (1.04, 1.39)	1.21 (1.04, 1.4)	1.21 (1.04, 1.41)
	Other pharyngeal cancer	1.16 (1.13, 1.18)	1.56 (1.45, 1.68)	2.16 (1.9, 2.46)	2.73 (2.3, 3.23)	3.23 (2.65, 3.93)	3.67(2.95, 4.56)	4.04 (3.2, 5.11)	4.35 (3.4, 5.56)	4.58 (3.55, 5.92)	4.75 (3.66, 6.17)
	Prostate cancer	1.04 (1.0, 1.09)	1.08 (1.0, 1.15)	1.1 (1.0, 1.2)	1.11 (1.0, 1.23)	1.13 (1.0, 1.27)	1.14 (1.0, 1.3)	1.16 (1.0, 1.33)	1.17 (1.0, 1.36)	1.18 (1.0, 1.38)	1.19 (1.0, 1.41)
	Stomach cancer	1.03 (0.99, 1.06)	1.06 (0.99, 1.14)	1.09 (0.98, 1.22)	1.12 (0.98, 1.29)	1.15 (0.97, 1.36)	1.17 (0.97, 1.43)	1.19 (0.97, 1.48)	1.21 (0.96, 1.51)	1.21 (0.96, 1.53)	1.22 (0.96, 1.54)
**Cardiovascular diseases**	Atrial fibrillation and flutter	1.02 (1.01, 1.04)	1.1 (1.05, 1.15)	1.2 (1.1, 1.31)	1.29 (1.14, 1.46)	1.37 (1.17, 1.59)	1.44 (1.21, 1.71)	1.5 (1.23, 1.82)	1.54 (1.25, 1.9)	1.58 (1.26, 1.97)	1.6 (1.27, 2.01)
	Ischaemic heart disease	0.92 (0.81, 1.05)	0.84 (0.63, 1.12)	0.77 (0.5, 1.18)	0.72 (0.42, 1.23)	0.7 (0.39, 1.25)	0.71 (0.41, 1.24)	0.75 (0.47, 1.2)	0.81 (0.58, 1.14)	0.9 (0.76, 1.07)	1.01 (0.99, 1.04)
	Ischaemic stroke	0.87 (0.72, 1.04)	0.89 (0.77, 1.03)	1.01 (1.0, 1.03)	1.12 (0.97, 1.3)	1.22 (0.94, 1.59)	1.31 (0.92, 1.87)	1.39 (0.91, 2.13)	1.46 (0.89, 2.37)	1.51 (0.88, 2.57)	1.55 (0.88, 2.73)
	Haemorrhagic stroke	0.92 (0.83, 1.02)	0.97 (0.93, 1.01)	1.09 (0.98, 1.2)	1.2 (0.96, 1.49)	1.3 (0.94, 1.79)	1.38 (0.92, 2.08)	1.46 (0.91, 2.35)	1.53 (0.9, 2.6)	1.58 (0.89, 2.81)	1.62 (0.89, 2.97)
**Other diseases**	Alzheimer’s disease and other dementias	0.89 (0.83, 0.95)	0.83 (0.74, 0.92)	0.82 (0.73, 0.92)	0.87 (0.80, 0.94)	0.94 (0.90, 0.97)	1.02 (1.01, 1.03)	1.10 (1.04, 1.16)	1.19 (1.08, 1.32)	1.29 (1.12, 1.49)	1.40 (1.16, 1.69)
	Cirrhosis and other chronic liver diseases	1.12 (1.05, 1.19)	1.4 (1.16, 1.69)	1.83 (1.3, 2.59)	2.43 (1.47, 4.03)	3.19 (1.65, 6.16)	4.11 (1.85, 9.15)	5.00 (2.01, 12.44)	5.71 (2.13, 15.3)	6.23 (2.21, 17.55)	6.56 (2.26, 19.05)
	Type 2 diabetes	0.84 (0.73, 0.97)	0.8 (0.67, 0.96)	0.87 (0.78, 0.98)	0.95 (0.91, 0.99)	1.02 (1.0, 1.04)	1.09 (1.02, 1.17)	1.14 (1.02, 1.28)	1.19 (1.03, 1.38)	1.23 (1.04, 1.46)	1.26 (1.04, 1.52)
	Lower respiratory infections	1.00 (1.0, 1)	1.00 (1.0, 1.00)	1.01 (1.00, 1.01)	1.06 (1.01, 1.11)	1.17 (1.02, 1.34)	1.34 (1.03, 1.73)	1.56 (1.05, 2.34)	1.85 (1.07, 3.22)	2.2 (1.08, 4.46)	2.61 (1.1, 6.18)
	Pancreatitis	1.01 (1.0, 1.01)	1.03 (1.01, 1.05)	1.13 (1.06, 1.21)	1.41 (1.17, 1.69)	1.85 (1.33, 2.56)	2.43 (1.52, 3.89)	3.15 (1.71, 5.78)	3.99 (1.91, 8.32)	4.95 (2.12, 11.58)	6.02 (2.32, 15.64)
	Tuberculosis	1.07 (0.98, 1.17)	1.29 (0.94, 1.76)	1.66 (0.89, 3.11)	2.2 (0.83, 5.82)	2.92 (0.78, 10.95)	3.83 (0.73, 20.07)	4.76 (0.7, 32.61)	5.55 (0.67, 45.96)	6.19 (0.65, 58.65)	6.67 (0.64, 69.3)

The reported RR and its 95% UI reflect the risk of developing each outcome of interest when comparing current alcohol use versus no alcohol use after accounting for between-study heterogeneity.

### Two-star associations

Two-star associations occur when the BPRF suggests a change in risk of 0–15% (increase) or 0–13% (decrease; ROS 0.00–0.14). Nine outcomes met this criterion: oesophageal cancer ( ≥ 15% increase; ROS 0.14), breast cancer ( ≥ 12%; 0.11), Alzheimer’s disease and other dementias ( ≥ 6.4% decrease; 0.07), atrial fibrillation and flutter ( ≥ 6%; 0.06), type 2 diabetes ( ≥ 5% decrease; 0.05), liver cancer ( ≥ 3%; 0.03), pancreatic cancer ( ≥ 3%; 0.03), lower respiratory infections ( ≥ 2%; 0.02) and prostate cancer ( ≥ 1%; 0.01).

Here, we present two exemplars: type 2 diabetes and oesophageal cancer. For type 2 diabetes, 452 observations from 83 cohort studies across 68 locations were analysed (Supplementary Tables 9 and 10)^69–151^. Alcohol exposure was 0–158 g per day, with the 85th percentile at 38 g per day. The estimated relationship was U-shaped (Fig. 1c), with decreasing risk at low consumption levels and increasing risk at higher consumption. The theoretical minimum risk exposure level (TMREL) occurred at 18 g per day (RR 0.80, 95% UI 0.67–0.97). Above the TMREL, the protective association weakens and disappears at 47 g per day—the non-drinker equivalence level—beyond which alcohol consumption is associated with higher risk than no drinking. At 85th percentile of exposure (37.5 g per day), the mean RR was 0.93 (0.88–0.99). Mean RR estimates were 0.84 (0.73–0.97) at 10 g per day, 0.80 (0.67–0.96) at 20 g per day, and 0.95 (0.91–0.99) at 40 g per day. The BPRF (Fig. 1c) suggested at least a 5% reduction in risk (ROS = 0.05). Observations based on mortality RRs or lacking age adjustment were corrected as bias covariates (Supplementary Tables 13 and 14). Moderate between-study heterogeneity remained after adjustment, but the model fit the data well (Fig. 1c). Evidence of publication bias remained after trimming 10% of outliers.

For oesophageal cancer, 258 observations from 18 cohort and 48 case–control studies were included (Supplementary Tables 9 and 10)^42,53,60,152–214^. Alcohol exposure was 0–189 g per day, with the 85th percentile at 75 g per day. Alcohol consumption showed a gradually increasing harmful association with oesophageal cancer risk (Fig. 1d). Mean RR estimates were 1.32 (95% UI: 1.0–1.75) at 10 g per day, 1.74 (1.0–3.05) at 20 g per day and 2.79 (0.99–7.8) at 40 g per day; at 75 g per day mean RR reached 4.32 (0.99–18.83). The BPRF (Fig. 1d) indicated at least a 15% increase in risk (ROS = 0.14). Observations lacking age adjustment were adjusted as bias covariates. The meta-analytic model accounted for study-design differences and remaining heterogeneity and showed good agreement with the observed data (Fig. 1d). No publication bias was detected after trimming 10% of observations. Further details on study characteristics and selected bias covariates can be found in Supplementary Tables 9–11.

**Extended Data Fig. 3:**
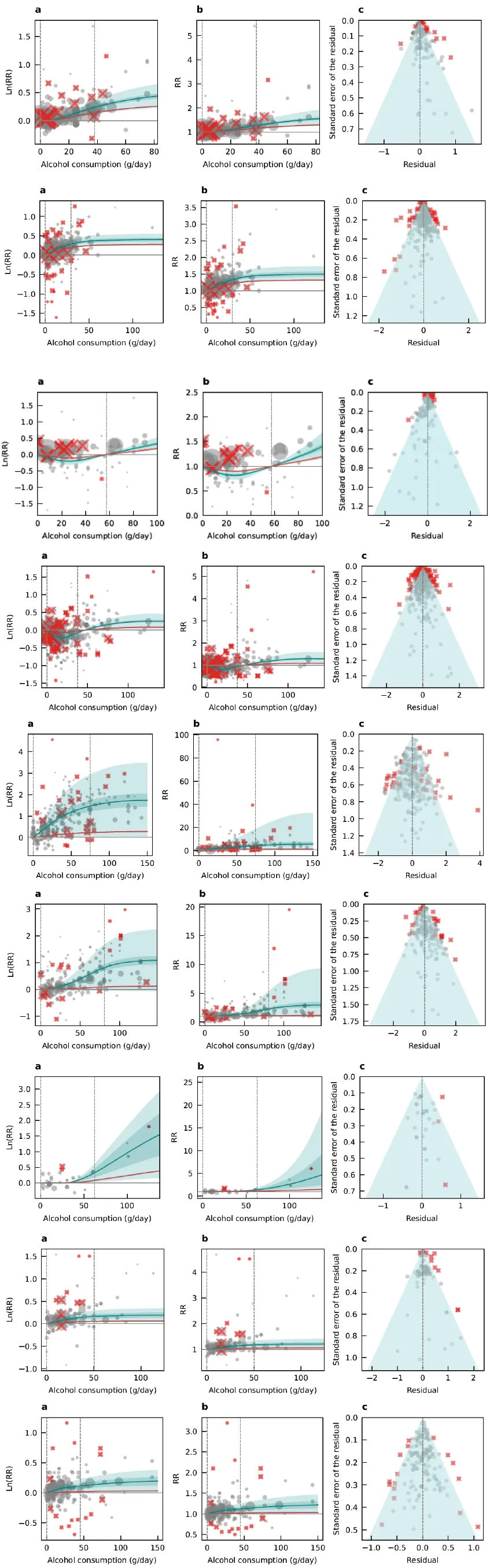
2-Star Relative Risk Curves. From top (1) to bottom (9), the plots correspond to the following outcomes: "Atrial fibrillation and flutter", "Breast cancer", "Alzheimer’s disease and other dementias", "Type 2 diabetes", "Oesophageal cancer", "Liver cancer", "Lower respiratory infections", "Pancreatic cancer", and "Prostate cancer". The following risk curves are computed relative to no alcohol consumption. In panels (**a**) and (**b**), the dark line indicates mean relative risk across alcohol consumption levels (in g/day); the light and dark shading show 95% uncertainty intervals with and without between-study heterogeneity, respectively, with the red line highlighting the burden of proof function; the size of the data points corresponds to the inverse of the standard error, with those trimmed during the model fitting process marked by a red x; and the dashed lines represent the 15th percentile of the reference exposure and the 85th percentile of the alternative exposure. To visualize log-relative- risk points in panel (**a**), we plotted each data point with the x-value at the midpoint of the alternative group and the y-value offset by the difference between the reported and predicted log risk. In panel (**b**), we exponentiated the y-values from panel (**a**) to yield the relative risk curve. Panel (**c**) depicts a customized funnel plot, with the x-axis representing residuals between predicted and observed relative risks, and the y-axis representing uncertainty from both measurement error and between-study heterogeneity. RR relative risk, UI uncertainty interval.

Results for other two-star outcomes are summarized in Tables 2 and 3 and Extended Data Fig. 3 and Extended Data Table 2.

### One-star associations

One-star associations occur when the ROS is negative, but conventional UIs (excluding between-study heterogeneity) exclude the null (RR = 1), suggesting weak or inconsistent evidence. Five outcomes met this criterion: stomach cancer (ROS − 0.0006), haemorrhagic stroke ( − 0.005), ischaemic stroke ( − 0.01), tuberculosis ( − 0.02) and ischaemic heart disease ( − 0.07).

As an illustration, we evaluated alcohol consumption and ischaemic heart disease using 1,043 observations from 157 cohort and 7 case–control studies across 79 locations (Supplementary Tables 9 and 10)^55,56,60,125,127,215–373^. Alcohol exposure ranged from 0 g to 225 g per day, with the 85th percentile at 45 g per day.

The estimated relationship between alcohol intake and ischaemic heart disease risk was U-shaped (Fig. 1e), with lower risk at moderate intake and higher risk at greater consumption. Risk declined until the TMREL of 52 g per day (RR 0.70, 95% UI 0.39–1.25), then increased until exceeding 99 g per day—the non-drinker equivalence level—beyond which risk exceeded that of non-drinkers. Mean RR estimates were 0.92 (0.81–1.05) at 10 g per day, 0.84 (0.63–1.12) at 20 g per day and 0.72 (0.42–1.23) at 40 g per day; at 45 g per day the mean RR was 0.71 (0.40–1.24). Because the BPRF crossed the null and the ROS was negative, the conservative interpretation indicates no clear association between alcohol consumption and ischaemic heart disease risk (Fig. 1e). Observations based only on mortality outcomes were adjusted as a bias covariate (Supplementary Tables 13 and 14). Substantial between-study heterogeneity remained after adjustment, although the model fit the data well (Fig. 1e). Publication bias was still detectable after trimming 10% of outliers. Additional study details are provided in Supplementary Tables 9–11.

**Extended Data Fig. 4:**
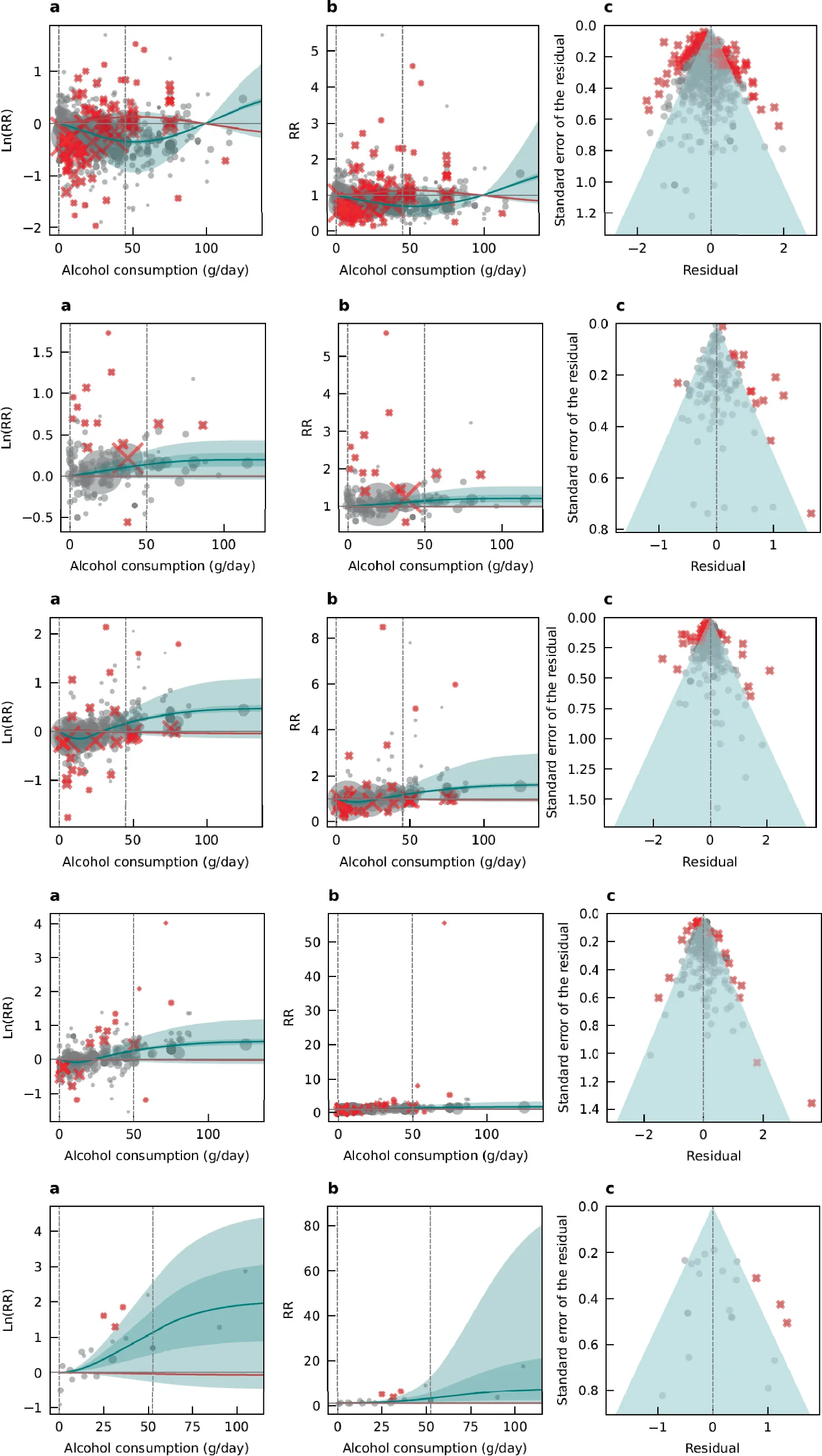
1-Star Relative Risk Curves. From top (1) to bottom (5), the plots correspond to the following outcomes: "Ischaemic heart disease", "Stomach cancer", "Ischaemic stroke", "Haemorrhagic stroke", and "Tuberculosis". The following risk curves are computed relative to no alcohol consumption. In panels (**a**) and (**b**), the dark line indicates mean relative risk across alcohol consumption levels (in g/day); the light and dark shading show 95% uncertainty intervals with and without between-study heterogeneity, respectively, with the red line highlighting the burden of proof function; the size of the data points corresponds to the inverse of the standard error, with those trimmed during the model fitting process marked by a red x; and the dashed lines represent the 15th percentile of the reference exposure and the 85th percentile of the alternative exposure. To visualize log-relative- risk points in panel (**a**), we plotted each data point with the x-value at the midpoint of the alternative group and the y-value offset by the difference between the reported and predicted log risk. In panel (**b**), we exponentiated the y-values from panel (**a**) to yield the relative risk curve. Panel (**c**) depicts a customized funnel plot, with the x-axis representing residuals between predicted and observed relative risks, and the y-axis representing uncertainty from both measurement error and between-study heterogeneity. RR relative risk, UI uncertainty interval.

Results for the remaining one-star outcomes are summarized in Tables 2 and 3, Extended Data Fig. 4 and Extended Data Table 2.

### Zero-star associations

Zero-star associations occur when the conventional UIs include the null (RR = 1), indicating no robust evidence of an association. No outcomes met this criterion.

## Discussion

Using the Burden of Proof approach, we re-evaluated dose–response relationships between current alcohol use and risks of 20 health outcomes, finding both harmful and protective associations. Based on the BPRF metrics, which provide a conservative interpretation of the existing data, evidence for an association with other pharyngeal cancer (excluding nasopharyngeal cancers) was very strong, with alcohol use at average intake levels associated with at least a 105% increased risk (five-star rating). Moderate evidence (three stars; >15–50% increased risk at average intake) was observed for cirrhosis and other chronic liver diseases, pancreatitis and cancers of the colorectal, larynx and mouth. Weak but consistent evidence (two stars) suggested small ( > 0–15%) increases in risk of several cancers, atrial fibrillation and flutter, and lower respiratory infections, and small reductions in risk for Alzheimer’s disease and other dementias (6.4%) and type 2 diabetes (5%). Weak or inconsistent evidence (one star) suggested lower risk of ischaemic heart disease and stroke, and higher risk of tuberculosis and stomach cancer, partly driven by substantial between-study heterogeneity.

Consistent with previous meta-analyses^4,5^, we observed moderate-to-strong harmful associations between alcohol use and nine cancers, with risk monotonically increasing with consumption. Even low consumption ( < 10 g per day) was associated with elevated risk for cancers of the pharynx, colorectum, larynx, lip and oral cavity, oesophagus, breast, liver, pancreas and prostate, which together accounted for 5.6% of global deaths in 2021^374^. For stomach cancer, evidence was weak after accounting for between-study heterogeneity, although mean risk increased with higher consumption^5,375^. Overall, our findings reinforce the well-established carcinogenic effects of alcohol, even at low levels of consumption^376^.

For cardiometabolic and dementia outcomes, our results broadly align with prior meta-analyses. Compared to abstinence, low-to-moderate alcohol consumption was associated with lower risks of ischaemic heart disease, ischaemic and haemorrhagic stroke, type 2 diabetes, and Alzheimer’s disease and other dementias, whereas higher intake was associated with increased risk^9,11,14,377,378^. In our previous Burden of Proof analysis of ischaemic heart disease, average consumption was associated with at least a 4% lower risk (two-star rating)^29^. In this re-evaluation, the overall relationship remained similar, but the inclusion of 42 additional studies increased between-study heterogeneity, resulting in a one-star rating. Short-term trials suggest plausible biological pathways through which low-to-moderate intake could reduce cardiometabolic risk^379,380^. However, trials also show that reducing intake among individuals consuming more than 24 g per day lowers systolic and diastolic blood pressure^381^. Lower dementia risk may partly reflect shared risk factors with cardiovascular diseases and type 2 diabetes^378,382^. However, no trial has yet investigated the long-term health impacts of alcohol consumption, and our findings from available observational studies may be biased due to residual confounding^383^. Consistent with this, the recent American Heart Association Scientific Statement on Alcohol Use and Cardiovascular Disease^384^ similarly concluded that although low-to-moderate alcohol intake ( ≤ 1–2 drinks per day) may be associated with lower cardiovascular risk, the evidence is primarily observational and subject to bias.

Findings from Mendelian randomization studies, which assess risk using genetically predicted lifetime alcohol use as the exposure, predominantly align with our results, supporting a claim of elevated risks for the cancer types examined^385,386^. However, they do not provide evidence to support lower risks for ischaemic heart disease, stroke, type 2 diabetes or Alzheimer’s disease and other dementias. Findings for ischaemic heart disease are mixed, suggesting no or positive associations^29^^,313^^,387^. Similarly, they suggest no or positive associations with ischaemic^388–390^ and haemorrhagic stroke^313,390^, type 2 diabetes^387,391–393^ and all-cause dementia^394,395^, and no association with late-onset Alzheimer’s disease^395,396^. Available Mendelian randomization studies, however, (i) do not disentangle different dimensions of alcohol use because genetically predicted lifetime average intake combines average consumption with heavy episodic drinking; (ii) typically assume log-linear associations, which may oversimplify complex dose–response relationships^397^; and (iii) do not account for variation in alcohol use over time^398^. Advancements in Mendelian randomization to improve estimation of non-linear relationships and evaluation of time-varying exposures may yield more nuanced insight into dose–response risk relationships and reconcile inconsistencies between study designs^399–401^.

This meta-analysis presents results that have several implications for drinking guidelines and public health messaging. Our findings provide a comprehensive assessment of relationships between current alcohol consumption and a range of major diseases, reinforcing the need for evidence-based recommendations. While existing guidelines vary widely, defining lower-risk thresholds from 8 g to 42 g per day for females and 10 g to 52 g per day for males^2^, current evidence does not support sex-specific thresholds. Specifically, we did not observe systematic differences in effect estimates stratified by sex, and the proportion of males was not selected as a significant bias covariate for any outcome in the meta-regression models (Table 2). Additionally, guidelines should emphasize reducing overall alcohol intake and discouraging heavy episodic drinking, both consistently associated with increased health risks^402^. Importantly, while risks are more pronounced at higher levels of consumption, our findings indicate that even low-to-moderate levels of alcohol intake are associated with substantial increases in risk for several outcomes (Table 3). This underscores the need for public health messaging that remains objective, while clearly communicating these elevated risks at low consumption levels.

Given global variation in disease burden, drinking patterns and underlying risk factors, public health recommendations may need to be tailored at the population level, rather than relying on a single, universal threshold. Where guidelines are absent, their development should be informed by the most up-to-date and robust evidence across health outcomes; existing guidelines, such as the Dietary Guidelines for Americans, should be regularly revised to incorporate the most updated evidence on the health risks associated with alcohol consumption. Guidelines should move beyond arbitrary thresholds and instead emphasize clear, evidence-based public health messaging that informs population-level understanding of alcohol-related risks^403^.

Our findings suggest elevated risks for major cancers associated with any level of alcohol consumption, increasing progressively as intake rises (Table 3). These include colorectal, oesophageal, breast and pancreatic cancers, which rank among leading contributors to global disease burden and premature mortality^374,404^. Despite strong evidence, public awareness of the link between alcohol and cancers remains considerably low, particularly for breast and colon cancer^405,406^. This underscores an urgent need for clear, evidence-based communication about alcohol’s carcinogenic effects. While any level of alcohol consumption promotes cancer formation, our study has shown that low-to-moderate alcohol consumption is associated with a lower risk of cardiovascular diseases, type 2 diabetes, and Alzheimer’s disease and other dementias. Public health strategies for alcohol consumption should reflect this complexity. Ultimately, alcohol’s impact on individual health varies considerably by factors such as sex, age, drinking patterns, socioeconomic status and other behavioural risk factors^407^. For instance, older adults, who face a high burden of cardiovascular diseases^408^, may experience some cardiovascular benefits from low-to-moderate alcohol consumption. Conversely, younger populations face a relatively low burden due to cardiovascular disease, type 2 diabetes and dementia^409^, and thus should avoid consuming alcohol. This heterogeneity of impact underscores the need for tailored public health guidance rather than one-size-fits-all recommendations, stressing the potential harms associated with high intake levels.

By providing a systematic, comparative and conservative assessment of alcohol-related risks, this study informs evidence-based policy and public health efforts. Our findings, including the mean risk function, BPRF, ROS, average excess or reduced risk and star rating for each alcohol–outcome association, provide valuable insights for a wide range of stakeholders. Policymakers can use the ROSs and star ratings to guide alcohol regulation, prioritize public health interventions and allocate resources efficiently. Clinicians and public health practitioners can leverage the estimates of risks and star ratings to identify patients at high risk for early intervention and educate patients about health risks associated with alcohol consumption, guiding discussions on harm reduction and responsible drinking. For researchers, the BPRFs, ROSs and star ratings enable comparisons of evidence strength across risk factors and outcomes, with low star ratings identifying areas of research opportunity. For individuals, these results offer a better, clearer picture of health risks and potential benefits associated with alcohol consumption, empowering informed and personalized decisions about alcohol consumption.

Our study has several limitations. Observational studies of alcohol-related health risks are susceptible to bias from unmeasured confounding^410^. In the included studies, alcohol consumption and key confounders such as smoking and diet were self-reported, which may introduce measurement error. Although we adjusted for study quality using selected bias covariates, these reflect observable study characteristics and cannot fully eliminate residual bias from unmeasured factors. We also did not assess other potential forms of publication or reporting bias, such as unexpectedly high agreement across studies^21^. Due to limited data, we did not estimate risk relationships by beverage type or patterns of consumption (for example, the same weekly consumption distributed across different drinking frequencies or heavy episodic drinking). Furthermore, to maximize use of the available evidence, we did not distinguish between subtypes of certain health outcomes, although alcohol may have different associations across the subtypes. Additionally, our study included only cohort and case–control studies; for some outcomes, results from Mendelian randomization studies may differ. Finally, while the star-rating system provides a useful heuristic for understanding the strength of evidence for an association between alcohol consumption and a health outcome, as a summary metric, it does not fully convey all relevant information. It is particularly important to regularly update the analyses for one-star and two-star associations as new evidence may substantially alter the estimated relationships.

Given substantial variation in burden from these diseases across ages and regions^409^, current evidence does not support a universally applicable threshold for alcohol consumption that maximizes health for all^411^. Instead, public health guidance should be population-specific, considerate of both RRs across intake levels and the overall burden of these outcomes in populations. Importantly, our findings should not be interpreted as endorsing alcohol consumption for health benefits. While low-to-moderate intake is modestly associated with lower risks of certain cardiovascular diseases, type 2 diabetes, and Alzheimer’s disease and other dementias, these associations are observational and uncertain, and must be considered alongside well-established harms for most outcomes, including increased cancer risks even at very low levels of consumption.

## Methods

### Overview

To evaluate the health effects of alcohol consumption, we conducted systematic reviews and applied the Burden of Proof framework^21^ to estimate RRs associated with alcohol consumption (in grams per day) for 20 health outcomes: atrial fibrillation and flutter, Alzheimer’s disease and other dementias, ischaemic heart disease, ischaemic and haemorrhagic stroke, cancers of the breast, colorectum, oesophagus, larynx, liver, mouth, pharynx, pancreas, prostate, stomach, cirrhosis and other chronic liver diseases, lower respiratory infections, pancreatitis, type 2 diabetes and tuberculosis. Alzheimer’s disease and other dementias, along with stomach, pancreatic and prostate cancers, were newly incorporated for GBD 2023, while evidence for previously included outcomes was updated using recent literature^411,412^. Injuries, a substantial proportion of which are attributable to alcohol use and occur across several complex modalities, merit additional attention and were not included in the scope of this study.

The Burden of Proof approach uses MR-BRT^30^ to assess the dose–response relationships between alcohol consumption and outcome-specific RRs. This approach incorporates between-study heterogeneity into uncertainty estimates, improves estimation of non-linear dose–response relationships, allows varying exposure reference ranges across studies, identifies and trims potential outliers and adjusts for significant bias covariates. Estimating alcohol-related risks requires harmonizing RRs across studies with differing exposure definitions, accounting for substantial heterogeneity reported in prior meta-analyses^413,414^ and adjusting for study-level biases.

As described previously^21^, the Burden of Proof framework involves six steps: (1) systematic review of evidence for each risk–outcome pair; (2) estimation of the shape of exposure–response relationship; (3) identification and adjustment of bias covariates; (4) incorporation of between-study heterogeneity into uncertainty estimates; (5) assessment of publication bias; and (6) estimation of the BPRF, a conservative representation of the exposure–risk relationship. These analyses generate a ROS, which is translated into a one-to-five-star evidence rating^22–29^. The study follows the Guidelines for Accurate and Transparent Health Estimates Reporting (GATHER)^415^ (Supplementary Table 18) and PRISMA recommendations^31^ (Supplementary Tables 16 and 17). The study was registered with PROSPERO on 16 June 2022 (ID: 337630; https://www.crd.york.ac.uk/PROSPERO/view/CRD42022337630/).

### The pragmatic systematic review

We conducted a pragmatic systematic review to identify peer-reviewed studies examining the association between alcohol consumption and each outcome. First, we identified recent high-quality systematic reviews for each outcome and evaluated all studies included in those reviews for eligibility. The reported end date of each review’s literature search served as the start date for our updated search. Selected reviews and their corresponding search dates are listed in Supplementary Table 7. This approach allowed the review to focus on newly published literature not covered by prior systematic reviews. Search strategies were developed in consultation with a University of Washington Health Sciences Librarian and applied to four databases: PubMed, Embase, CINAHL and Web of Science. Records indexed from the designated start dates through 31 December 2023 were retrieved. Outcome-specific search strings are provided in Supplementary Tables 2–6. Retrieved references were collated and de-duplicated using DistillerSR (Evidence Partners), a systematic review management platform designed to support screening and data extraction^416,417^.

Studies were eligible if they (1) used cohort or case–control designs, (2) reported RRs for discrete levels of alcohol consumption (or sufficient information to calculate them) and (3) defined outcomes consistent with GBD case definitions (Extended Data Table 1). Additional outcome-specific exclusion criteria are described in Supplementary Table 1.

After de-duplication, titles and abstracts were independently screened by S.I.N. and H.R.L. until 50% of records were reviewed without disagreement. Remaining records were screened using DistillerSR AI^417^, including studies with a predicted inclusion probability greater than 0.5. Full-text articles were independently reviewed by S.I.N. and H.R.L., with disagreements resolved through discussion with X.D.

Data were extracted using standardized forms in DistillerSR (Supplementary Table 8). Extracted information included study design, exposure levels, outcome definitions, adjusted confounders and reported effect sizes (RRs, hazard ratios, or odds ratios) with corresponding uncertainty. When necessary, effect estimates were converted to reflect alcohol intake in grams of pure ethanol per day. Additional details are provided in the Supplementary Information (section VII).

### Estimating the shape of the exposure–RR risk relationship

Dose–response relationships between alcohol consumption (0–150 g per day) and the risk of each outcome were estimated using MR-BRT, a meta-regression framework. To allow flexible, non-log-linear relationships, we applied quadratic splines with two interior knots. MR-BRT integrates exposure ranges from both reference and alternative categories, enabling estimation of dose–response relationships across studies with differing exposure definitions. RRs, odds ratios and hazard ratios were included as effect measures when accompanied by uncertainty estimates. To reduce sensitivity to knot placement, we estimated ensemble dose–response curves by combining 50 component models with randomly positioned knots, weighted by model fit and total variation. Potential influence from outliers was addressed using a least-trimmed-squares procedure that removed 10% of observations. Additional methodological details are described elsewhere^21^.

For each outcome, an unconstrained model was first estimated. Based on visual inspection of the resulting curves, three additional specifications were evaluated: monotonicity constraints, linear tail constraints in regions with sparse exposure data, and a combination of both. Results for all model specifications are provided in the Supplementary Information (section XI and Figs. 2.01–2.20). Final models were selected based on model fit and clinical plausibility following consultation with subject-matter experts. Parameter and constraint details for each outcome are reported in the Supplementary Information (section X).

As a sensitivity analysis, we re-estimated dose–response relationships using the selected model constraints but without trimming potential outliers (Supplementary Figs. 3.01–3.20).

### Testing and adjusting for biases across study designs and characteristics

We evaluated and adjusted for potential systematic biases arising from study characteristics. For each included study, indicators were created for six possible sources of bias based on the GRADE framework^418^ criteria: representativeness of the study population, methods used to assess exposure and outcome, reverse causation, control for confounding and selection bias. Significant bias covariates were identified using a stepwise Lasso procedure^419^. Interaction terms between the estimated dose–response relationship from the previous modelling step and each bias covariate were sequentially added to a linear meta-regression model until no additional covariates remained significant. Candidate covariates, such as age, sex, body mass index and potential bias from ‘sick quitting’, were considered for the final models. Definitions of bias covariates, their study-specific values, model specifications and the covariates included in each outcome model are reported in Supplementary Tables 11–14.

### Quantifying between-study heterogeneity

Unexplained between-study heterogeneity was estimated while accounting for uncertainty in the heterogeneity parameter and the limited number of available studies. We fitted a linear mixed-effects model to the log RRs, including the estimated dose–response relationship and bias covariates identified previously. The model incorporated a random intercept to capture within-study correlation and a study-specific slope for the exposure–response relationship. Because heterogeneity estimates can be unstable or underestimated when few studies are available, uncertainty in the between-study variance was quantified using the Fisher information matrix. This approach allowed calculation of 95% uncertainty intervals that incorporate both between-study heterogeneity and uncertainty in its estimation.

### Evaluating the potential publication and reporting biases

Potential publication or reporting bias was evaluated using Egger’s regression^420^, which assesses the relationship between model residuals and their standard errors. We additionally examined modified funnel plots that display residuals from the dose–response model against their corresponding standard deviations.

### Estimating the BPRF

The BPRF provides a conservative estimate of the dose–response relationship while accounting for between-study heterogeneity and systematic biases related to study characteristics. For outcomes positively associated with alcohol consumption, the BPRF corresponds to the 5th quantile of the estimated curve closest to the null (natural log(RR) = 0); for inverse associations, the 95th quantile is used. See Extended Data Figs. 1.1, 2.1–2.5, 3.1, 3.2, 3.5–3.9, 4.2 and 4.5 for positive associations, and Extended Data Figs. 3.3, 3.4, 4.1, 4.3 and 4.4 for inverse associations. The BPRF therefore represents the smallest plausible harmful or protective effect consistent with the available evidence. When the BPRF crosses the null, the evidence is considered insufficient to confirm an association; greater separation from the null indicates stronger evidence and larger effect sizes.

To quantify evidence strength, we calculated the ROS as the signed average of the natural logarithm of the BPRF across the 15th–85th percentiles of observed alcohol exposure. Positive ROS values indicate stronger and more consistent associations, whereas negative values suggest weak or inconsistent evidence after accounting for between-study heterogeneity.

Associations were summarized using a five-star evidence rating system^21^. A zero-star rating indicates no ROS because conventional uncertainty intervals include the null (RR = 1). One-star ratings correspond to negative ROS values with conventional uncertainty intervals excluding the null, reflecting weak and inconsistent evidence. Two-star associations have ROS values between 0 and 0.14, corresponding to small risk changes ( > 0–15% increase or >0–13% decrease). Three-star associations have ROS values ranging from >0.14 to 0.41 (15–50% increase or 13–34% decrease). Four-star associations range from >0.41 to 0.62 (50–85% increase or 34–46% decrease). Five-star associations exceed 0.62, indicating risk increases greater than 85% or risk reductions exceeding 46%.

### Model validation

The Burden of Proof framework applied in this study has been validated previously^21^. Final model specifications for each alcohol–outcome relationship were selected based on model fit, data availability and clinical plausibility. Dose–response curves were reviewed and discussed by all authors before finalization.

### Ethics

This study exclusively used publicly available, de-identified aggregated data; accordingly, no institutional review board approval was required.

### Reporting summary

Further information on research design is available in the Nature Portfolio Reporting Summary linked to this article.

## Supplementary information


Supplementary InformationSupplementary Methods, Tables 1–18 and Figs. 1.01–1.16, 2.01–2.20 and 3.01–3.20.
Reporting Summary


## Figures and Tables

**Extended Data Fig. 1:**
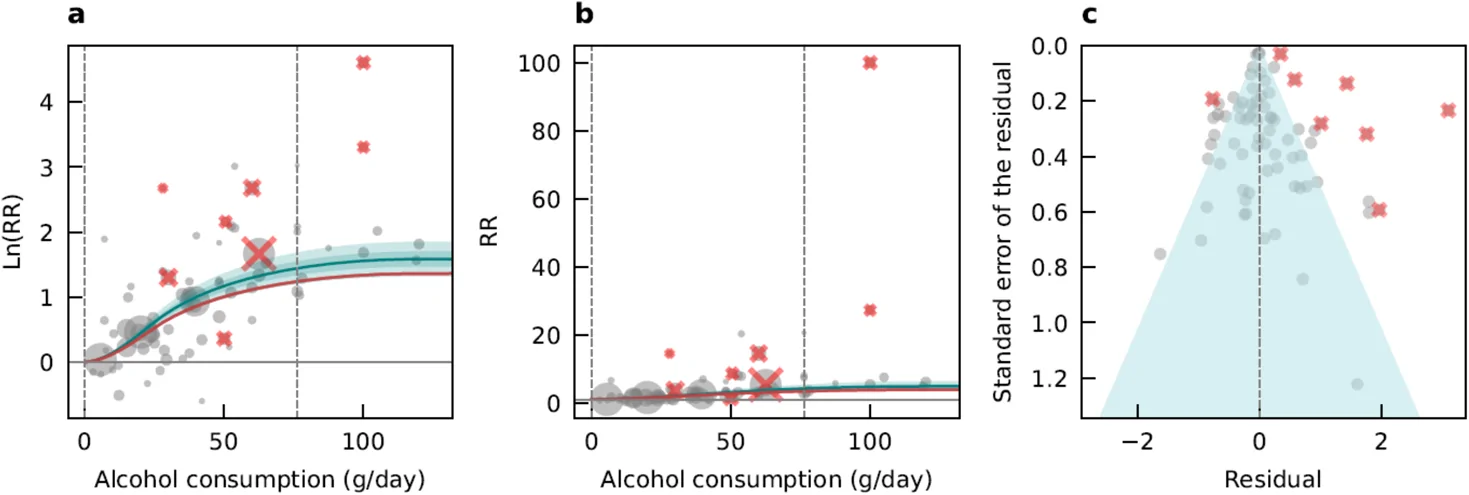
5-Star Relative Risk Curve. The plot is for alcohol consumption and other pharyngeal cancer. Risk curves are computed relative to no alcohol consumption. In panels (**a**) and (**b**), the dark line indicates mean relative risk across alcohol consumption levels (in g/day); the light and dark shading show 95% uncertainty intervals with and without between-study heterogeneity, respectively, with the red line highlighting the burden of proof function; the size of the data points corresponds to the inverse of the standard error, with those trimmed during the model fitting process marked by a red x; and the dashed lines represent the 15th percentile of the reference exposure and the 85th percentile of the alternative exposure. To visualize log-relative- risk points in panel (**a**), we plotted each data point with the x-value at the midpoint of the alternative group and the y-value offset by the difference between the reported and predicted log risk. In panel (**b**), we exponentiated the y-values from panel (**a**) to yield the relative risk curve. Panel (**c**) depicts a customized funnel plot, with the x-axis representing residuals between predicted and observed relative risks, and the y-axis representing uncertainty from both measurement error and between-study heterogeneity. RR relative risk, UI uncertainty interval.

**Extended Data Table 1:**
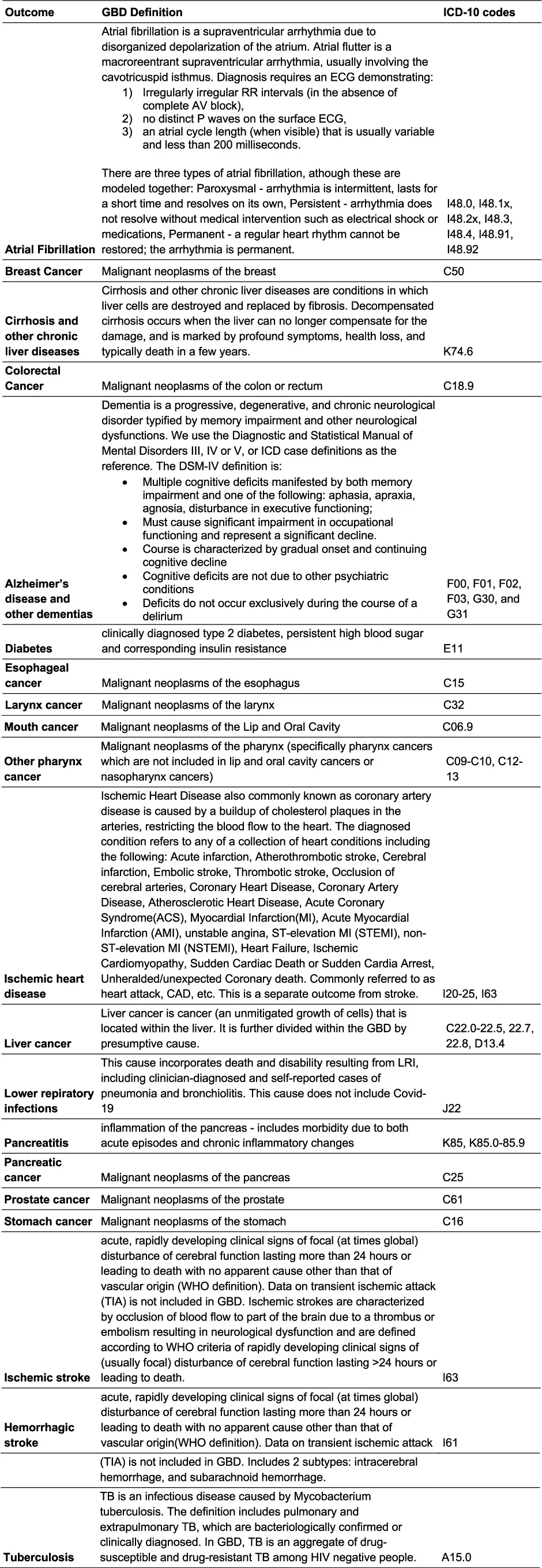
Outcome Definitions

Each outcome considered for this review was defined using the corresponding definition used in the Global Burden of Diseases, Risk Factors, and Injuries Study (GBD).

**Extended Data Table 2:**
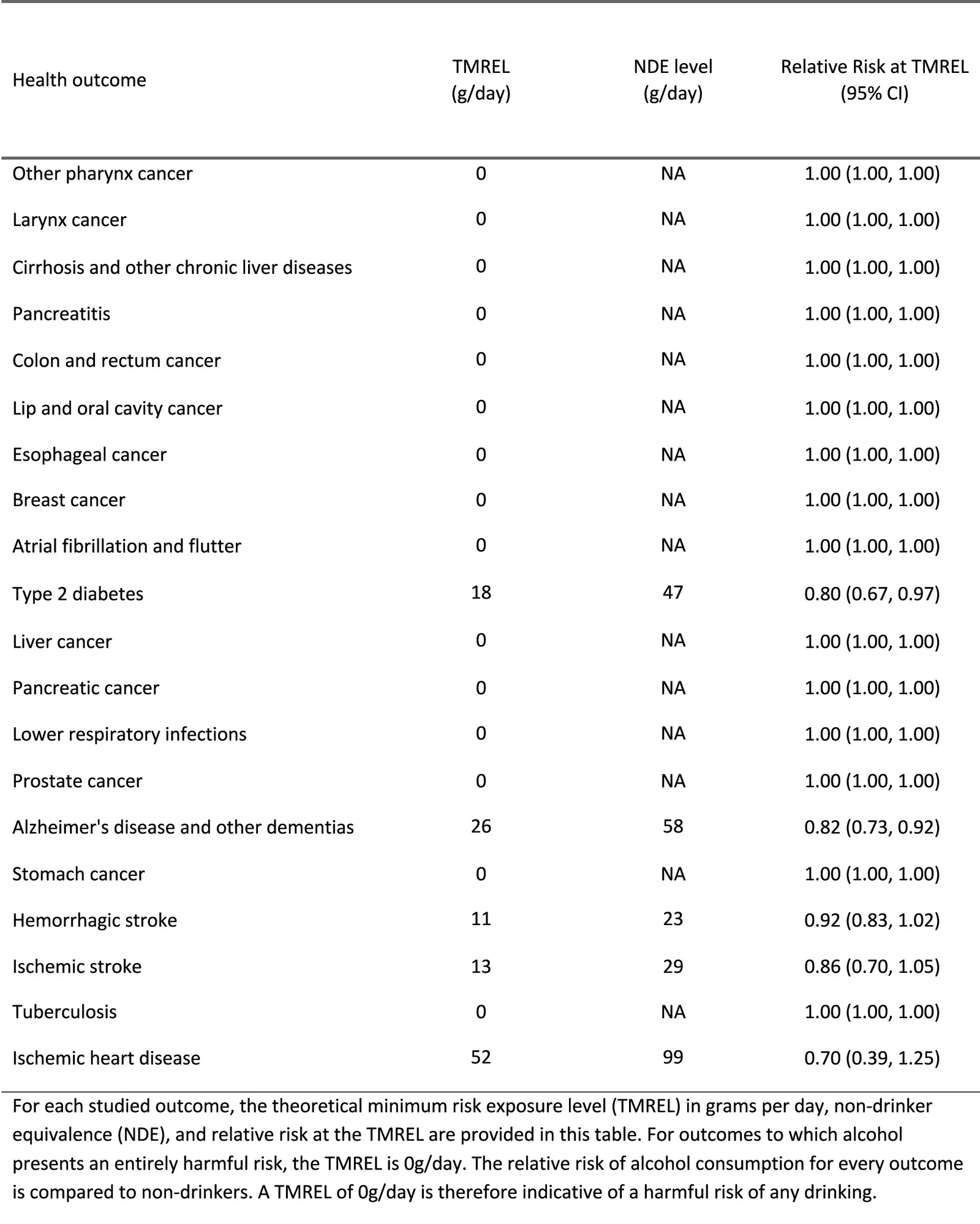
Theoretical Minimum Exposure Level and Non-Drinker Equivalence by Health Outcome

## Data Availability

The analyses in this study rely on data from publicly available repositories and published literature. Data sources and references for each risk–outcome pair can be downloaded from the corresponding risk curve pages on the Burden of Proof visualization platform (https://vizhub.healthdata.org/burden-of-proof/) using the ‘Download’ function. Characteristics of all input studies are also reported in Supplementary Tables 9 and 10.

## Code availability

All code used for the analyses is publicly available online (https://github.com/ihmeuw-msca/burden-of-proof/tree/main/). Analyses were conducted using R (version 4.2.0) and Python (version 3.12.14).
