# Supplementary material for: Health effects associated with alcohol consumption: a Burden of Proof study
Source: Nat Health. 2026 Jun 1;1(9):979–99. doi: 10.1038/s44360-026-00139-5 (PMC13533843; doi:10.1038/s44360-026-00139-5)
Supplement: Supplementary file 1 [file 44360_2026_139_MOESM1_ESM.pdf]

nature health

Supplementary information https://doi.org/10.1038/s44360-026-00139-5

Health effects associated with alcohol consumption: a Burden of Proof study

In the format provided by the authors and unedited

Table of Contents

I. Data Sources 3

II. Identification of Sources 3

III. Data Assessment 3

- A. Using DistillerSR 3
- B. Exposure Definitions 4
- C. Outcome Definitions 4
- D. Inclusion & Exclusion Criteria 4

IV. Search Strings 6

- A. Base Search Strings Across Databases 6
- B. Outcome and Database Specific Search Strings 8
- C. Best Systematic Reviews 23

V. Prisma Flow Diagrams 27

VI. Data Extraction 43

- A. Data Extraction Template 44

VII. Data Processing 51

VIII. Supplementary Methods of the Burden of Proof Approach 52

IX. Relative Risk Sources 55

- A. Study Characteristics 55
- B. Summary of Data Inputs 127
- C. Risk Of Bias By Study 180

X. Model Specifications 204

- A. MR-BRT Model Specifications by Risk-Outcome Pair 204
- B. Selected Bias Covariates and Gamma Solution by Risk-Outcome Pair 206
- C. Definitions of Considered Bias Covariates 207

XI. Sensitivity Analyses 209

- A. Model Settings 209
- B. Relative Risk Models Without Trimming 230

XII. Mean Risk at Different Levels of Consumption with Conventional 95% Uncertainty Intervals 236
XIII. PRISMA Checklists 239
A. PRISMA 2020 Abstract Checklist 239
B. PRISMA 2020 manuscript checklist 242
XIV. GATHER Checklist 249

I. Data Sources

This systematic review includes case-control and cohort studies examining the relationship between alcohol consumption and any of the twenty outcomes. These sources were identified by searching four major databases: PUBMED, CINAHL, EMBASE, and Web of Science.

II. Identification of Sources

For each outcome considered in the scope of this systematic review, the literature was reviewed to find a most-recent, high quality systematic review to use as a starting point for source identification. The underlying sources of each Best-MASR were reviewed for inclusion within this study, and the end dates of the review were used as the beginning dates for our search. This methodology was developed to increase efficiency while continuing to build on previous research. For each outcome included in this systematic review, the identified best previously conducted systematic review is detailed below.

Once the start date for each outcome's search was determined, we searched PUBMED, CINAHL, EMBASE, and web of science from the relevant date to present and collated the results within DistillerSR. Using DistillerSR's inbuilt duplicate detection, we automatically excluded any study with a match of over 63% likelihood of duplication. Any remaining studies that were flagged by the system as potential duplicates but with a likelihood less than 63% were manually reviewed. When de-duplicating, PUBMED sources were prioritized for inclusion. The relevant search terms for each database are listed under each outcome below.

For every systematic review and meta-analysis identified during our search for sources, we extracted and screened any alcohol consumption and/or outcome relevant studies (listed as "citation searching" in the PRISMA diagrams under each outcome).

III. Data Assessment

A. Using DistillerSR

Data assessment was completed in DistillerSR using a hierarchical format of data evaluation. First, all sources identified across the four databases were imported into DistillerSR using the reference upload tool. We used DistillerSR's automatic deduplication tool to obtain a list of unique records for review, excluding any records with a match of more than 63% as potential duplicates.

Studies were reviewed by two independent screeners for inclusion based on titles and abstracts. After 50% of the total number of sources per outcome were manually double-screened and any conflicts resolved, we enabled the DistillerSR AI screening to complete the remaining sources. We used a threshold of 0.5, where studies with a likelihood of inclusion score greater than 0.5 were included and studies with a likelihood of inclusion score less than 0.5 were excluded. This formed our set of sources that were passed on to FT screening.

B. Exposure Definitions

For the purposes of this review, alcohol consumption was considered to be consumption of any beverage containing greater than 1g of ethanol. When reported, specific drink concentrations were extracted and converted into grams of pure ethanol per day (See: "Data Processing" for additional details).

C. Outcome Definitions

Each outcome considered for this review was defined using the corresponding definition used in the Global Burden of Diseases, Risk Factors, and Injuries Study (GBD), as detailed in Extended Data Table E1.

D. Inclusion & Exclusion Criteria

At the full-text screening stage, every source was reviewed by two independent screeners for inclusion. The specific inclusion and exclusion criteria is listed below under each relevant outcome, however, in general studies were included if they:

(1) Were case control or cohort studies
(2) Categorized alcohol consumption in a discrete measure (ie g/day, drinks/week)
(3) Reported a direct alcohol-outcome relative risk AND included a measure of uncertainty

Studies were excluded if they:

(1) Were cross-sectional studies
(2) Were a duplicate of another study
(3) Could not quantify alcohol consumption in g/day (ie only reports a composite measure of alcohol consumption)

(4) Did not report measure for specific outcome of interest (for example, stroke instead of ischemic or hemorrhagic stroke)
(5) Restricted analysis to a highly specific population (such as diabetics, cancer patients, etc.)

In addition to these criteria, specific exclusion reasons were identified for each outcome.

Table S1. Outcome-specific inclusion and exclusion criteria

	Inclusion criteria	Exclusion criteria
**Atrial fibrillation**		• Non-specific tachycardia
**Breast cancer**		• Receiving hormone replacement
**Cirrhosis and other chronic liver diseases**		• NAFLD – related cirrhosis
**Colorectal cancer**		
**Alzheimer's disease and other dementias**	• Cognitive decline or diagnosed dementia	
**Type 2 diabetes**		• Type 1 diabetes<br>• Metabolic syndrome
**Esophageal cancer**		
**Larynx cancer**		
**Mouth cancer**		
**Other pharynx cancer**	• Cancers of the pharynx	
**Ischemic heart disease**	• Heart disease, heart attack	• Non-specific heart failure
**Liver cancer**		• Diagnosis of liver disease or cirrhosis
**Lower respiratory infections**	• Pneumonias	• Diagnosis of COVID-19
**Pancreatitis**		
**Pancreatic cancer**		
**Prostate cancer**		
**Stomach cancer**		
**Stroke**	• Ischemic or hemorrhagic stroke	• Non-specific stroke
**Tuberculosis**		

IV. Search Strings

Each search string was developed through consultation with a librarian at the University of Washington Health Sciences library.

A. Base Search Strings Across Databases

Table S2. Base search strings across databases

PUBMED	(alcoholic beverage[Mesh] OR drinking behavior[Mesh] OR “alcohol”[tiab] OR "alcoholic beverage*"[tiab] OR "drinking behavior*"[tiab] OR "drinking behaviour*"[tiab]) AND ("Risk"[Mesh] OR "relative risk"[tiab] OR "hazard ratio"[tiab] OR "odds ratio"[tiab] OR "rate"[tiab] OR "risk"[tiab] OR "RRs"[tiab]) AND ("Cohort Studies"[Mesh] OR "Prospective Studies"[Mesh] OR ("cohort"[tiab] AND ("prospective"[tiab] OR "pooled"[tiab] OR "follow-up"[tiab] OR "meta-analysis"[tiab])) OR "Case-Control Studies"[Mesh] OR "case-control"[tiab] OR "case control"[tiab] OR "case-crossover"[tiab]) NOT ("cross-sectional"[tiab] OR ("cohort"[tiab] AND "retrospective"[tiab])) AND (*Date of the most recent, best-quality SR (see Supp Table XX. Best Systematic Reviews)* : "2022/12/31"[PDat]) NOT (animals[Mesh] NOT Humans[Mesh]) AND
CINAHL	(MH("Alcoholic Beverages+" OR "Drinking Behavior+") OR TI("alcohol" OR "alcoholic beverage*" OR "drinking behavior*" OR "drinking behaviour*") OR AB("alcohol" OR "alcoholic beverage*" OR "drinking behavior*" OR "drinking behaviour*")) AND (MH("Odds Ratio" OR "Relative Risk" OR "Attributable Risk") OR TI("risk" OR "odds ratio" OR "cross-product ratio" OR "hazards ratio" OR "hazard ratio") OR AB("risk" OR "odds ratio" OR "cross-product ratio" OR "hazards ratio" OR "hazard ratio")) AND
EMBASE	('alcoholic beverage'/exp OR 'drinking behavior'/exp OR ('alcohol' OR 'alcoholic beverage*' OR 'drinking behavior*' OR 'drinking behaviour*'):ti,ab,kw) AND ('risk'/exp OR 'odds ratio'/exp OR 'hazard ratio'/exp OR 'attributable risk'/exp OR ('risk' OR 'odds ratio' OR 'cross-product ratio' OR 'hazards ratio' OR 'hazard ratio'):ti,ab,kw) AND [2020-2023]/py NOT ('animal'/exp NOT 'human'/exp) AND ('Cohort Studies'/exp OR 'Prospective Studies'/exp OR (cohort:ti,ab AND (prospective:ti,ab OR pooled:ti,ab OR follow-up:ti,ab OR meta-analysis:ti,ab)) OR 'Case-Control Studies'/exp OR case-control:ti,ab OR 'case control':ti,ab OR case-crossover:ti,ab) NOT (cross-sectional:ti,ab OR (cohort:ti,ab AND retrospective:ti,ab)) AND
WEB OF SCIENCE	TS=("alcohol" OR "alcoholic beverage*" OR "drinking behavior*" OR "drinking behaviour*") AND TS=("risk" OR "odds ratio" OR "cross-product ratio" OR "hazards ratio" OR "hazard ratio") AND (ALL="Cohort Studies" OR ALL="Prospective Studies" OR ((TI=cohort OR AB=cohort) AND ((TI=prospective OR AB=prospective) OR (TI=pooled OR AB=pooled) OR (TI=follow-up OR AB=follow-up) OR (TI=meta-analysis OR AB=meta-

	analysis))) OR ALL="Case-Control Studies" OR (TI=case-control OR AB=case-control) OR (TI="case control" OR AB="case control") OR (TI=case-crossover OR AB=case-crossover)) NOT ((TI=cross-sectional OR AB=cross-sectional) OR ((TI=cohort OR AB=cohort) AND (TI=retrospective OR AB=retrospective))) AND

B. Outcome and Database Specific Search Strings

Table S3. Outcome specific search strings for PUBMED

PUBMED BASE SEARCH	(alcoholic beverage[Mesh] OR drinking behavior[Mesh] OR “alcohol”[tiab] OR "alcoholic beverage*"[tiab] OR "drinking behavior*"[tiab] OR "drinking behaviour*"[tiab]) AND ("Risk"[Mesh] OR "relative risk"[tiab] OR "hazard ratio"[tiab] OR "odds ratio"[tiab] OR "rate"[tiab] OR "risk"[tiab] OR "RRs"[tiab]) AND ("Cohort Studies"[Mesh] OR "Prospective Studies"[Mesh] OR ("cohort"[tiab] AND ("prospective"[tiab] OR "pooled"[tiab] OR "follow-up"[tiab] OR "meta-analysis"[tiab])) OR "Case-Control Studies"[Mesh] OR "case-control"[tiab] OR "case control"[tiab] OR "case-crossover"[tiab]) NOT ("cross-sectional"[tiab] OR ("cohort"[tiab] AND "retrospective"[tiab])) AND ("1970/01/01"[PDat] : "2022/12/31"[PDat]) NOT (animals[Mesh] NOT Humans[Mesh]) AND
**Atrial fibrillation**	(Atrial Fibrillation[Mesh] OR Atrial Flutter[Mesh] OR "atrial fibrillation*"[tiab] OR "atrial fibrilation*"[tiab] OR "auricular fibrillation*"[tiab] OR "auricular fibrilation*"[tiab] OR "atrial flutter*"[tiab] OR "atrium flutter*"[tiab] OR "auricular flutter*"[tiab])
**Breast cancer**	(Breast Neoplasm[Mesh] OR "breast cancer*"[tiab] OR "breast neoplasm*"[tiab] OR "breast carcinoma*"[tiab] OR "breast tumor*"[tiab] OR "breast tumour*"[tiab] OR "mammary cancer*"[tiab] OR "mammary neoplasm*"[tiab] OR "mammary carcinoma*"[tiab] OR "mammary tumor*"[tiab] OR "mammary tumour*"[tiab] OR "breast malignant neoplasm*"[tiab] OR "cancer of the breast"[tiab] OR "cancers of the breast"[tiab] OR "neoplasm of the breast"[tiab] OR "neoplasms of the breast"[tiab] OR "tumor of the breast"[tiab] OR "tumors of the breast"[tiab] OR "tumour of the breast"[tiab] OR "tumours of the breast"[tiab])
**Cirrhosis and other chronic liver diseases**	(Liver Cirrhosis[Mesh] OR End Stage Liver Disease[Mesh] OR cirrhosis[tiab] OR cirrhoses[tiab] OR "liver fibrosis"[tiab] OR "liver fibroses"[tiab] OR "chronic liver failure"[tiab] OR "chronic hepatic failure'"[tiab] OR "end stage liver disease"[tiab] OR "chronic liver disease"[tiab])
**Colorectal cancer**	(Colorectal Neoplasm[Mesh] OR "colorectal cancer*"[tiab] OR "colorectal neoplasm*"[tiab] OR "colorectal tumor*"[tiab] OR "colorectal tumour*"[tiab] OR "colorectal carcinoma*"[tiab] OR "colonic neoplasm*"[tiab] OR "colonic cancer*"[tiab] OR "colon cancer*"[tiab] OR "colon neoplasm*"[tiab] OR "rectal cancer*"[tiab] OR "rectal neoplasm*"[tiab] OR "rectal tumor*"[tiab] OR "rectal tumour*"[tiab] OR "rectum neoplasm*"[tiab] OR "rectum cancer*"[tiab] OR "rectal tumor*"[tiab] OR "rectal tumour*"[tiab] OR "cancer of the colon"[tiab] OR "cancers of the colon"[tiab] OR "cancer of the rectum"[tiab] OR "cancers of the rectum"[tiab] OR "bowel cancer"[tiab] OR "large bowel cancer"[tiab])
**Alzheimer’s disease and other dementias**	(dementia[Mesh] OR dementia[TiAb] OR cognition[TiAb] OR cognitive decline[TiAb] OR alzheimer disease[MeSH] OR Alzheimer[TiAb])
**Type 2 diabetes**	(Diabetes Mellitus, Type 2[Mesh] OR diabetes[tiab])
**Esophageal cancer**	("esophageal cancer*"[tiab] OR "esophageal neoplasm*"[tiab] OR "esophagus cancer*"[tiab] OR "esophagus neoplasm*"[tiab] OR "esophageal

	squamous cell carcinoma"[tiab] OR "cancer of the esophagus"[tiab] OR "cancers of the esophagus"[tiab] OR "neoplasm of the esophagus"[tiab] OR "neoplasms of the esophagus"[tiab] OR "oesophageal cancer*"[tiab] OR "oesophageal neoplasm*"[tiab] OR "oesophagus cancer*"[tiab] OR "oesophagus neoplasm*"[tiab] OR "oesophageal squamous cell carcinoma"[tiab] OR "cancer of the oesophagus"[tiab] OR "cancers of the oesophagus"[tiab] OR "neoplasm of the oesophagus"[tiab] OR "neoplasms of the oesophagus"[tiab])
**Larynx cancer**	("laryngeal cancer*"[tiab] OR "laryngeal neoplasm*"[tiab] OR "larynx cancer*"[tiab] OR "larynx neoplasm*"[tiab] OR "cancer of the larynx"[tiab] OR "cancers of the larynx"[tiab] OR "neoplasm of the larynx"[tiab] OR "neoplasms of the larynx"[tiab])
**Mouth cancer**	("mouth cancer*"[tiab] OR "mouth neoplasm*"[tiab] OR "cancer of mouth"[tiab] OR "cancer of the mouth"[tiab] OR "cancers of the mouth"[tiab] OR "oral cancer*"[tiab] OR "oral neoplasm*"[tiab] OR "gingival neoplasm*"[tiab] OR "gingival cancer*"[tiab])
**Other pharynx cancer**	("pharyngeal cancer*"[tiab] OR "pharyngeal neoplasm*"[tiab] OR "pharynx cancer*"[tiab] OR "pharynx neoplasm*"[tiab] OR "cancer of the pharynx"[tiab] OR "cancers of the pharynx"[tiab])
**Ischemic heart disease**	(Coronary Artery Disease[Mesh] OR Myocardial Ischemia[Mesh] OR atherosclerosis[Mesh] OR "coronary artery disease"[tiab] OR "myocardial ischemia"[tiab] OR "myocardial ischaemia"[tiab] OR "cardiac ischemia"[tiab] OR "cardiac ischaemia"[tiab] OR "silent ischemia"[tiab] OR "silent ischaemia"[tiab] OR "atherosclerosis"[tiab] OR "ischaemic heart disease"[tiab] OR "ischemic heart disease"[tiab] OR "coronary heart disease"[tiab] OR "myocardial infarct*"[tiab] OR "heart attack"[tiab] OR "heart infarct*"[tiab] OR "cardiac infarct*"[tiab])
**Liver cancer**	(Liver Neoplasm[Mesh] OR cholangiocarcinoma[Mesh] OR "liver cancer*"[tiab] OR "cancer of the liver"[tiab] OR "cancers of the liver"[tiab] OR "liver neoplasm*"[tiab] OR "hepatic cancer*"[tiab] OR "hepatic neoplasm*"[tiab] OR "hepatocellular neoplasm*"[tiab] OR "hepatocellular cancer*"[tiab] OR "liver cell carcinoma*"[tiab] OR "hepatoma*"[tiab] OR "hepatocellular carcinoma*"[tiab] OR "hepatic carcinoma*"[tiab] OR "cholangiocarcinoma*"[tiab] OR "intrahepatic bile duct carcinoma*"[tiab])
**Lower respiratory infections**	(pneumonia[Mesh] OR bronchiolitis[Mesh] OR "lower respiratory"[tiab] OR pneumonia[tiab] OR bronchiolitis[tiab] OR "pulmonary inflammation"[tiab] OR "lung inflammation"[tiab])
**Pancreatitis**	(Pancreatitis[Mesh] OR pancreatitis[tiab] OR pancreatitides[tiab])
**Pancreatic cancer**	(Pancreatic Neoplasm[Mesh] OR "pancreas cancer*"[tiab] OR "pancreas neoplasm*"[tiab] OR "pancreas carcinoma*"[tiab] OR "pancreas tumor*"[tiab] OR "pancreas tumour*"[tiab] OR "pancreatic cancer*"[tiab] OR "pancreatic neoplasm*"[tiab] OR "pancreatic carcinoma*"[tiab] OR "pancreatic tumor*"[tiab] OR "pancreatic tumour*"[tiab] OR "pancreatic malignant neoplasm*"[tiab] OR "cancer of the pancreas"[tiab] OR "cancers of the pancreas"[tiab] OR "neoplasm of the pancreas"[tiab] OR "neoplasms of the pancreas"[tiab] OR "tumor of the pancreas"[tiab] OR "tumors of the

	pancreas"[tiab] OR "tumour of the pancreas"[tiab] OR "tumours of the pancreas"[tiab])
**Prostate cancer**	(Prostatic Neoplasms[Mesh] OR "prostate cancer*"[tiab] OR "prostate neoplasm*"[tiab] OR "prostate carcinoma*"[tiab] OR "prostate tumor*"[tiab] OR "prostate tumour*"[tiab] OR "prostate tumor*"[tiab] OR "prostate tumour*"[tiab] OR "prostate malignant neoplasm*"[tiab] OR "cancer of the prostate"[tiab] OR "cancers of the prostate"[tiab] OR "neoplasm of the prostate"[tiab] OR "neoplasms of the prostate"[tiab] OR "tumor of the prostate"[tiab] OR "tumors of the prostate"[tiab] OR "tumour of the prostate"[tiab] OR "tumours of the prostate"[tiab] OR "neoplasms, prostate" [ot] OR "neoplasms, prostatic" [ot])
**Stomach cancer**	(Stomach Neoplasms[Mesh] OR Gastric Neoplasms[Mesh] OR "stomach cancer*"[tiab] OR "stomach neoplasm*"[tiab] OR "stomach carcinoma*"[tiab] OR "stomach tumor*"[tiab] OR "stomach tumour*"[tiab] OR "gastric cancer*"[tiab] OR "gastric neoplasm*"[tiab] OR "gastric carcinoma*"[tiab] OR "gastric tumor*"[tiab] OR "gastric tumour*"[tiab] OR "gastric malignant neoplasm*"[tiab] OR "cancer of the stomach"[tiab] OR "cancers of the stomach"[tiab] OR "neoplasm of the stomach"[tiab] OR "neoplasms of the stomach"[tiab] OR "tumor of the stomach"[tiab] OR "tumors of the stomach"[tiab] OR "tumour of the stomach"[tiab] OR "tumours of the stomach"[tiab])
**Stroke**	(brain infarction[Mesh] OR stroke[Mesh] OR intracranial hemorrhages[Mesh] OR stroke[tiab] OR "cerebrovascular accident"[tiab] OR "brain infarct*"[tiab] OR "cerebral infarct*"[tiab] OR "cortex infarct*"[tiab] OR "cerebrovascular infarct*"[tiab] OR "cortical infarct*"[tiab] OR "hemisphere infarct*"[tiab] OR "hemispheric infarct*"[tiab] OR "intracerebral hemorrhage"[tiab] OR "intracerebral haemorrhage"[tiab] OR "subarachnoid hemorrhage"[tiab] OR "subarachnoid haemorrhage"[tiab] OR "brain hemorrhage"[tiab] OR "brain haemorrhage"[tiab])
**Tuberculosis**	(tuberculosis[Mesh] OR "Mycobacterium tuberculosis"[Mesh] OR tuberculosis[tiab] OR TB[tiab])

Table S4. Outcome specific search strings for CINAHL

CINAHL BASE SEARCH	(MH("Alcoholic Beverages+" OR "Drinking Behavior+") OR TI("alcohol" OR "alcoholic beverage*" OR "drinking behavior*" OR "drinking behaviour*") OR AB("alcohol" OR "alcoholic beverage*" OR "drinking behavior*" OR "drinking behaviour*")) AND (MH("Odds Ratio" OR "Relative Risk" OR "Attributable Risk") OR TI("risk" OR "odds ratio" OR "cross-product ratio" OR "hazards ratio" OR "hazard ratio") OR AB("risk" OR "odds ratio" OR "cross-product ratio" OR "hazards ratio" OR "hazard ratio")) AND
**Atrial fibrillation**	((MH "Atrial Fibrillation"+) OR (MH "Atrial Flutter"+) OR (TI "atrial fibrillation*" OR AB "atrial fibrillation*") OR (TI "atrial fibrilation*" OR AB "atrial fibrilation*") OR (TI "auricular fibrillation*" OR AB "auricular fibrillation*") OR (TI "auricular fibrilation*" OR AB "auricular fibrilation*") OR (TI "atrial flutter*" OR AB "atrial flutter*") OR (TI "atrium flutter*" OR AB "atrium flutter*") OR (TI "auricular flutter*" OR AB "auricular flutter*"))
**Breast cancer**	"breast cancer*"
**Cirrhosis and other chronic liver diseases**	((MH "Liver Cirrhosis"+) OR (MH "End Stage Liver Disease"+) OR (TI cirrhosis OR AB cirrhosis) OR (TI cirrhoses OR AB cirrhoses) OR (TI "liver fibrosis" OR AB "liver fibrosis") OR (TI "liver fibroses" OR AB "liver fibroses") OR (TI "chronic liver failure" OR AB "chronic liver failure") OR (TI "chronic hepatic failure'" OR AB "chronic hepatic failure'") OR (TI "end stage liver disease" OR AB "end stage liver disease") OR (TI "chronic liver disease" OR AB "chronic liver disease"))
**Colorectal cancer**	((MH "Colorectal Neoplasm"+) OR (TI "colorectal cancer*" OR AB "colorectal cancer*") OR (TI "colorectal neoplasm*" OR AB "colorectal neoplasm*") OR (TI "colorectal tumor*" OR AB "colorectal tumor*") OR (TI "colorectal tumour*" OR AB "colorectal tumour*") OR (TI "colorectal carcinoma*" OR AB "colorectal carcinoma*") OR (TI "colonic neoplasm*" OR AB "colonic neoplasm*") OR (TI "colonic cancer*" OR AB "colonic cancer*") OR (TI "colon cancer*" OR AB "colon cancer*") OR (TI "colon neoplasm*" OR AB "colon neoplasm*") OR (TI "rectal cancer*" OR AB "rectal cancer*") OR (TI "rectal neoplasm*" OR AB "rectal neoplasm*") OR (TI "rectal tumor*" OR AB "rectal tumor*") OR (TI "rectal tumour*" OR AB "rectal tumour*") OR (TI "rectum neoplasm*" OR AB "rectum neoplasm*") OR (TI "rectum cancer*" OR AB "rectum cancer*") OR (TI "rectal tumor*" OR AB "rectal tumor*") OR (TI "rectal tumour*" OR AB "rectal tumour*") OR (TI "cancer of the colon" OR AB "cancer of the colon") OR (TI "cancers of the colon" OR AB "cancers of the colon") OR (TI "cancer of the rectum" OR AB "cancer of the rectum") OR (TI "cancers of the rectum" OR AB "cancers of the rectum") OR (TI "bowel cancer" OR AB "bowel cancer") OR (TI "large bowel cancer" OR AB "large bowel cancer"))
**Alzheimer’s disease and other dementias**	(MM "Dementia") OR (MM "alzheimer*") OR (MM “cognitive decline”)
**Type 2 diabetes**	((MH "Diabetes Mellitus, Type 2"+) OR (TI diabetes OR AB diabetes))
**Esophageal cancer**	((TI "esophageal cancer*" OR AB "esophageal cancer*") OR (TI "esophageal neoplasm*" OR AB "esophageal neoplasm*") OR (TI "esophagus cancer*" OR

	AB "esophagus cancer*") OR (TI "esophagus neoplasm*" OR AB "esophagus neoplasm*") OR (TI "esophageal squamous cell carcinoma" OR AB "esophageal squamous cell carcinoma") OR (TI "cancer of the esophagus" OR AB "cancer of the esophagus") OR (TI "cancers of the esophagus" OR AB "cancers of the esophagus") OR (TI "neoplasm of the esophagus" OR AB "neoplasm of the esophagus") OR (TI "neoplasms of the esophagus" OR AB "neoplasms of the esophagus") OR (TI "oesophageal cancer*" OR AB "oesophageal cancer*") OR (TI "oesophageal neoplasm*" OR AB "oesophageal neoplasm*") OR (TI "oesophagus cancer*" OR AB "oesophagus cancer*") OR (TI "oesophagus neoplasm*" OR AB "oesophagus neoplasm*") OR (TI "oesophageal squamous cell carcinoma" OR AB "oesophageal squamous cell carcinoma") OR (TI "cancer of the oesophagus" OR AB "cancer of the oesophagus") OR (TI "cancers of the oesophagus" OR AB "cancers of the oesophagus") OR (TI "neoplasm of the oesophagus" OR AB "neoplasm of the oesophagus") OR (TI "neoplasms of the oesophagus" OR AB "neoplasms of the oesophagus"))
**Larynx cancer**	((TI "laryngeal cancer*" OR AB "laryngeal cancer*") OR (TI "laryngeal neoplasm*" OR AB "laryngeal neoplasm*") OR (TI "larynx cancer*" OR AB "larynx cancer*") OR (TI "larynx neoplasm*" OR AB "larynx neoplasm*") OR (TI "cancer of the larynx" OR AB "cancer of the larynx") OR (TI "cancers of the larynx" OR AB "cancers of the larynx") OR (TI "neoplasm of the larynx" OR AB "neoplasm of the larynx") OR (TI "neoplasms of the larynx" OR AB "neoplasms of the larynx"))
**Mouth cancer**	((TI "mouth cancer*" OR AB "mouth cancer*") OR (TI "mouth neoplasm*" OR AB "mouth neoplasm*") OR (TI "cancer of mouth" OR AB "cancer of mouth") OR (TI "cancer of the mouth" OR AB "cancer of the mouth") OR (TI "cancers of the mouth" OR AB "cancers of the mouth") OR (TI "oral cancer*" OR AB "oral cancer*") OR (TI "oral neoplasm*" OR AB "oral neoplasm*") OR (TI "gingival neoplasm*" OR AB "gingival neoplasm*") OR (TI "gingival cancer*" OR AB "gingival cancer*"))
**Other pharynx cancer**	((TI "pharyngeal cancer*" OR AB "pharyngeal cancer*") OR (TI "pharyngeal neoplasm*" OR AB "pharyngeal neoplasm*") OR (TI "pharynx cancer*" OR AB "pharynx cancer*") OR (TI "pharynx neoplasm*" OR AB "pharynx neoplasm*") OR (TI "cancer of the pharynx" OR AB "cancer of the pharynx") OR (TI "cancers of the pharynx" OR AB "cancers of the pharynx"))
**Ischemic heart disease**	((MH "Coronary Artery Disease"+) OR (MH "Myocardial Ischemia"+) OR (MH atherosclerosis+) OR (TI "coronary artery disease" OR AB "coronary artery disease") OR (TI "myocardial ischemia" OR AB "myocardial ischemia") OR (TI "myocardial ischaemia" OR AB "myocardial ischaemia") OR (TI "cardiac ischemia" OR AB "cardiac ischemia") OR (TI "cardiac ischaemia" OR AB "cardiac ischaemia") OR (TI "silent ischemia" OR AB "silent ischemia") OR (TI "silent ischaemia" OR AB "silent ischaemia") OR (TI atherosclerosis OR AB atherosclerosis) OR (TI "ischaemic heart disease" OR AB "ischaemic heart disease") OR (TI "ischemic heart disease" OR AB "ischemic heart disease") OR (TI "coronary heart disease" OR AB "coronary heart disease") OR (TI "myocardial infarct*" OR AB "myocardial infarct*") OR (TI "heart attack" OR AB "heart attack") OR (TI "heart infarct*" OR AB "heart infarct*") OR (TI "cardiac infarct*" OR AB "cardiac infarct*"))

**Liver cancer**	((MH "Liver Neoplasm"+) OR (MH cholangiocarcinoma+) OR (TI "liver cancer*" OR AB "liver cancer*") OR (TI "cancer of the liver" OR AB "cancer of the liver") OR (TI "cancers of the liver" OR AB "cancers of the liver") OR (TI "liver neoplasm*" OR AB "liver neoplasm*") OR (TI "hepatic cancer*" OR AB "hepatic cancer*") OR (TI "hepatic neoplasm*" OR AB "hepatic neoplasm*") OR (TI "hepatocellular neoplasm*" OR AB "hepatocellular neoplasm*") OR (TI "hepatocellular cancer*" OR AB "hepatocellular cancer*") OR (TI "liver cell carcinoma*" OR AB "liver cell carcinoma*") OR (TI hepatoma* OR AB hepatoma*) OR (TI "hepatocellular carcinoma*" OR AB "hepatocellular carcinoma*") OR (TI "hepatic carcinoma*" OR AB "hepatic carcinoma*") OR (TI cholangiocarcinoma* OR AB cholangiocarcinoma*) OR (TI "intrahepatic bile duct carcinoma*" OR AB "intrahepatic bile duct carcinoma*"))
**Lower respiratory infections**	((MH pneumonia+) OR (MH bronchiolitis+) OR (TI "lower respiratory" OR AB "lower respiratory") OR (TI pneumonia OR AB pneumonia) OR (TI bronchiolitis OR AB bronchiolitis) OR (TI "pulmonary inflammation" OR AB "pulmonary inflammation") OR (TI "lung inflammation" OR AB "lung inflammation"))
**Pancreatitis**	((MH Pancreatitis+) OR (TI pancreatitis OR AB pancreatitis) OR (TI pancreatitides OR AB pancreatitides))
**Pancreatic cancer**	((MH "Pancreatic Neoplasm"+) OR (TI "pancreas cancer*" OR AB "pancreas cancer*") OR (TI "pancreas neoplasm*" OR AB "pancreas neoplasm*") OR (TI "pancreas carcinoma*" OR AB "pancreas carcinoma*") OR (TI "pancreas tumor*" OR AB "pancreas tumor*") OR (TI "pancreas tumour*" OR AB "pancreas tumour*") OR (TI "pancreatic cancer*" OR AB "pancreatic cancer*") OR (TI "pancreatic neoplasm*" OR AB "pancreatic neoplasm*") OR (TI "pancreatic carcinoma*" OR AB "pancreatic carcinoma*") OR (TI "pancreatic tumor*" OR AB "pancreatic tumor*") OR (TI "pancreatic tumour*" OR AB "pancreatic tumour*") OR (TI "pancreatic malignant neoplasm*" OR AB "pancreatic malignant neoplasm*") OR (TI "cancer of the pancreas" OR AB "cancer of the pancreas") OR (TI "cancers of the pancreas" OR AB "cancers of the pancreas") OR (TI "neoplasm of the pancreas" OR AB "neoplasm of the pancreas") OR (TI "neoplasms of the pancreas" OR AB "neoplasms of the pancreas") OR (TI "tumor of the pancreas" OR AB "tumor of the pancreas") OR (TI "tumors of the pancreas" OR AB "tumors of the pancreas") OR (TI "tumour of the pancreas" OR AB "tumour of the pancreas") OR (TI "tumours of the pancreas" OR AB "tumours of the pancreas"))
**Prostate cancer**	((MH "Prostatic Neoplasms"+))
**Stomach cancer**	((MH "Stomach Neoplasms"+))
**Ischemic & Hemorrhagic Stroke**	((MH "brain infarction"+) OR (MH stroke+) OR (MH "intracranial hemorrhages"+) OR (TI stroke OR AB stroke) OR (TI "cerebrovascular accident" OR AB "cerebrovascular accident") OR (TI "brain infarct*" OR AB "brain infarct*") OR (TI "cerebral infarct*" OR AB "cerebral infarct*") OR (TI "cortex infarct*" OR AB "cortex infarct*") OR (TI "cerebrovascular infarct*" OR AB "cerebrovascular infarct*") OR (TI "cortical infarct*" OR AB "cortical infarct*") OR (TI "hemisphere infarct*" OR AB "hemisphere infarct*") OR (TI "hemispheric infarct*" OR AB "hemispheric infarct*") OR (TI "intracerebral hemorrhage" OR AB "intracerebral hemorrhage") OR (TI "intracerebral haemorrhage" OR AB "intracerebral haemorrhage") OR (TI "subarachnoid

	hemorrhage" OR AB "subarachnoid hemorrhage") OR (TI "subarachnoid haemorrhage" OR AB "subarachnoid haemorrhage") OR (TI "brain hemorrhage" OR AB "brain hemorrhage") OR (TI "brain haemorrhage" OR AB "brain haemorrhage"))
**Tuberculosis**	((MH tuberculosis+) OR (MH "Mycobacterium tuberculosis"+) OR (TI tuberculosis OR AB tuberculosis) OR (TI TB OR AB TB))

Table S5. Outcome specific search strings for EMBASE

EMBASE<br>BASE SEARCH	('alcoholic beverage'/exp OR 'drinking behavior'/exp OR ('alcohol' OR 'alcoholic beverage*' OR 'drinking behavior*' OR 'drinking behaviour*'):ti,ab,kw) AND ('risk'/exp OR 'odds ratio'/exp OR 'hazard ratio'/exp OR 'attributable risk'/exp OR ('risk' OR 'odds ratio' OR 'cross-product ratio' OR 'hazards ratio' OR 'hazard ratio'):ti,ab,kw) AND [2020-2023]/py NOT ('animal'/exp NOT 'human'/exp) AND ('Cohort Studies'/exp OR 'Prospective Studies'/exp OR (cohort:ti,ab AND (prospective:ti,ab OR pooled:ti,ab OR follow-up:ti,ab OR meta-analysis:ti,ab)) OR 'Case-Control Studies'/exp OR case-control:ti,ab OR 'case control':ti,ab OR case-crossover:ti,ab) NOT (cross-sectional:ti,ab OR (cohort:ti,ab AND retrospective:ti,ab)) AND
**Atrial fibrillation**	('Atrial Fibrillation'/exp OR 'Atrial Flutter'/exp OR 'atrial fibrillation*':ti,ab OR 'atrial fibrilation*':ti,ab OR 'auricular fibrillation*':ti,ab OR 'auricular fibrilation*':ti,ab OR 'atrial flutter*':ti,ab OR 'atrium flutter*':ti,ab OR 'auricular flutter*':ti,ab)
**Breast cancer**	('Breast Neoplasm'/exp OR 'breast cancer*':ti,ab OR 'breast neoplasm*':ti,ab OR 'breast carcinoma*':ti,ab OR 'breast tumor*':ti,ab OR 'breast tumour*':ti,ab OR 'mammary cancer*':ti,ab OR 'mammary neoplasm*':ti,ab OR 'mammary carcinoma*':ti,ab OR 'mammary tumor*':ti,ab OR 'mammary tumour*':ti,ab OR 'breast malignant neoplasm*':ti,ab OR 'cancer of the breast':ti,ab OR 'cancers of the breast':ti,ab OR 'neoplasm of the breast':ti,ab OR 'neoplasms of the breast':ti,ab OR 'tumor of the breast':ti,ab OR 'tumors of the breast':ti,ab OR 'tumour of the breast':ti,ab OR 'tumours of the breast':ti,ab)
**Cirrhosis and other chronic liver diseases**	('Liver Cirrhosis'/exp OR 'End Stage Liver Disease'/exp OR cirrhosis:ti,ab OR cirrhoses:ti,ab OR 'liver fibrosis':ti,ab OR 'liver fibroses':ti,ab OR 'chronic liver failure':ti,ab OR 'chronic hepatic failure':ti,ab OR 'end stage liver disease':ti,ab OR 'chronic liver disease':ti,ab)
**Colorectal cancer**	('Colorectal Neoplasm'/exp OR 'colorectal cancer*':ti,ab OR 'colorectal neoplasm*':ti,ab OR 'colorectal tumor*':ti,ab OR 'colorectal tumour*':ti,ab OR 'colorectal carcinoma*':ti,ab OR 'colonic neoplasm*':ti,ab OR 'colonic cancer*':ti,ab OR 'colon cancer*':ti,ab OR 'colon neoplasm*':ti,ab OR 'rectal cancer*':ti,ab OR 'rectal neoplasm*':ti,ab OR 'rectal tumor*':ti,ab OR 'rectal tumour*':ti,ab OR 'rectum neoplasm*':ti,ab OR 'rectum cancer*':ti,ab OR 'rectal tumor*':ti,ab OR 'rectal tumour*':ti,ab OR 'cancer of the colon':ti,ab OR 'cancers of the colon':ti,ab OR 'cancer of the rectum':ti,ab OR 'cancers of the rectum':ti,ab OR 'bowel cancer':ti,ab OR 'large bowel cancer':ti,ab)
**Alzheimer's disease and other dementias**	('dementia'/exp OR 'alzheimer disease'/exp OR 'dementia':ab,ti OR 'cognition':ab,ti OR 'cognitive decline':ab,ti OR 'alzheimer':ab,ti)
**Type 2 diabetes**	('Diabetes Mellitus, Type 2'/exp OR diabetes:ti,ab)
**Esophageal cancer**	('esophageal cancer*':ti,ab OR 'esophageal neoplasm*':ti,ab OR 'esophagus cancer*':ti,ab OR 'esophagus neoplasm*':ti,ab OR 'esophageal squamous cell carcinoma':ti,ab OR 'cancer of the esophagus':ti,ab OR 'cancers of the esophagus':ti,ab OR 'neoplasm of the esophagus':ti,ab OR 'neoplasms of the

	esophagus':ti,ab OR 'oesophageal cancer*':ti,ab OR 'oesophageal neoplasm*':ti,ab OR 'oesophagus cancer*':ti,ab OR 'oesophagus neoplasm*':ti,ab OR 'oesophageal squamous cell carcinoma':ti,ab OR 'cancer of the oesophagus':ti,ab OR 'cancers of the oesophagus':ti,ab OR 'neoplasm of the oesophagus':ti,ab OR 'neoplasms of the oesophagus':ti,ab)
**Larynx cancer**	('laryngeal cancer*':ti,ab OR 'laryngeal neoplasm*':ti,ab OR 'larynx cancer*':ti,ab OR 'larynx neoplasm*':ti,ab OR 'cancer of the larynx':ti,ab OR 'cancers of the larynx':ti,ab OR 'neoplasm of the larynx':ti,ab OR 'neoplasms of the larynx':ti,ab)
**Mouth cancer**	('mouth cancer*':ti,ab OR 'mouth neoplasm*':ti,ab OR 'cancer of mouth':ti,ab OR 'cancer of the mouth':ti,ab OR 'cancers of the mouth':ti,ab OR 'oral cancer*':ti,ab OR 'oral neoplasm*':ti,ab OR 'gingival neoplasm*':ti,ab OR 'gingival cancer*':ti,ab)
**Other pharynx cancer**	('pharyngeal cancer*':ti,ab OR 'pharyngeal neoplasm*':ti,ab OR 'pharynx cancer*':ti,ab OR 'pharynx neoplasm*':ti,ab OR 'cancer of the pharynx':ti,ab OR 'cancers of the pharynx':ti,ab)
**Ischemic heart disease**	('Coronary Artery Disease'/exp OR 'Myocardial Ischemia'/exp OR atherosclerosis/exp OR 'coronary artery disease':ti,ab OR 'myocardial ischemia':ti,ab OR 'myocardial ischaemia':ti,ab OR 'cardiac ischemia':ti,ab OR 'cardiac ischaemia':ti,ab OR 'silent ischemia':ti,ab OR 'silent ischaemia':ti,ab OR atherosclerosis:ti,ab OR 'ischaemic heart disease':ti,ab OR 'ischemic heart disease':ti,ab OR 'coronary heart disease':ti,ab OR 'myocardial infarct*':ti,ab OR 'heart attack':ti,ab OR 'heart infarct*':ti,ab OR 'cardiac infarct*':ti,ab)
**Liver cancer**	('Liver Neoplasm'/exp OR cholangiocarcinoma/exp OR 'liver cancer*':ti,ab OR 'cancer of the liver':ti,ab OR 'cancers of the liver':ti,ab OR 'liver neoplasm*':ti,ab OR 'hepatic cancer*':ti,ab OR 'hepatic neoplasm*':ti,ab OR 'hepatocellular neoplasm*':ti,ab OR 'hepatocellular cancer*':ti,ab OR 'liver cell carcinoma*':ti,ab OR hepatoma*:ti,ab OR 'hepatocellular carcinoma*':ti,ab OR 'hepatic carcinoma*':ti,ab OR cholangiocarcinoma*:ti,ab OR 'intrahepatic bile duct carcinoma*':ti,ab)
**Lower respiratory infections**	(pneumonia/exp OR bronchiolitis/exp OR 'lower respiratory':ti,ab OR pneumonia:ti,ab OR bronchiolitis:ti,ab OR 'pulmonary inflammation':ti,ab OR 'lung inflammation':ti,ab)
**Pancreatitis**	(Pancreatitis/exp OR pancreatitis:ti,ab OR pancreatitides:ti,ab)
**Pancreatic cancer**	('Pancreatic Neoplasm'/exp OR 'pancreas cancer*':ti,ab OR 'pancreas neoplasm*':ti,ab OR 'pancreas carcinoma*':ti,ab OR 'pancreas tumor*':ti,ab OR 'pancreas tumour*':ti,ab OR 'pancreatic cancer*':ti,ab OR 'pancreatic neoplasm*':ti,ab OR 'pancreatic carcinoma*':ti,ab OR 'pancreatic tumor*':ti,ab OR 'pancreatic tumour*':ti,ab OR 'pancreatic malignant neoplasm*':ti,ab OR 'cancer of the pancreas':ti,ab OR 'cancers of the pancreas':ti,ab OR 'neoplasm of the pancreas':ti,ab OR 'neoplasms of the pancreas':ti,ab OR 'tumor of the pancreas':ti,ab OR 'tumors of the pancreas':ti,ab OR 'tumour of the pancreas':ti,ab OR 'tumours of the pancreas':ti,ab)
**Prostate cancer**	('Prostatic Neoplasms'/exp OR 'prostate cancer*':ti,ab OR 'prostate neoplasm*':ti,ab OR 'prostate carcinoma*':ti,ab OR 'prostate tumor*':ti,ab OR

	'prostate tumour*':ti,ab OR 'prostate tumor*':ti,ab OR 'prostate tumour*':ti,ab OR 'prostate malignant neoplasm*':ti,ab OR 'cancer of the prostate':ti,ab OR 'cancers of the prostate':ti,ab OR 'neoplasm of the prostate':ti,ab OR 'neoplasms of the prostate':ti,ab OR 'tumor of the prostate':ti,ab OR 'tumors of the prostate':ti,ab OR 'tumour of the prostate':ti,ab OR 'tumours of the prostate':ti,ab OR 'neoplasms, prostate':kw OR 'neoplasms, prostatic':kw)
**Stomach cancer**	('Stomach Neoplasms'/exp OR 'Gastric Neoplasms'/exp OR 'stomach cancer*':ti,ab OR 'stomach neoplasm*':ti,ab OR 'stomach carcinoma*':ti,ab OR 'stomach tumor*':ti,ab OR 'stomach tumour*':ti,ab OR 'gastric cancer*':ti,ab OR 'gastric neoplasm*':ti,ab OR 'gastric carcinoma*':ti,ab OR 'gastric tumor*':ti,ab OR 'gastric tumour*':ti,ab OR 'gastric malignant neoplasm*':ti,ab OR 'cancer of the stomach':ti,ab OR 'cancers of the stomach':ti,ab OR 'neoplasm of the stomach':ti,ab OR 'neoplasms of the stomach':ti,ab OR 'tumor of the stomach':ti,ab OR 'tumors of the stomach':ti,ab OR 'tumour of the stomach':ti,ab OR 'tumours of the stomach':ti,ab)
**Ischemic & Hemorrhagic Stroke**	('brain infarction'/exp OR stroke/exp OR 'intracranial hemorrhages'/exp OR stroke:ti,ab OR 'cerebrovascular accident':ti,ab OR 'brain infarct*':ti,ab OR 'cerebral infarct*':ti,ab OR 'cortex infarct*':ti,ab OR 'cerebrovascular infarct*':ti,ab OR 'cortical infarct*':ti,ab OR 'hemisphere infarct*':ti,ab OR 'hemispheric infarct*':ti,ab OR 'intracerebral hemorrhage':ti,ab OR 'intracerebral haemorrhage':ti,ab OR 'subarachnoid hemorrhage':ti,ab OR 'subarachnoid haemorrhage':ti,ab OR 'brain hemorrhage':ti,ab OR 'brain haemorrhage':ti,ab)
**Tuberculosis**	(tuberculosis/exp OR 'Mycobacterium tuberculosis'/exp OR tuberculosis:ti,ab OR TB:ti,ab)

Table S6. Outcome specific search strings for WEB OF SCIENCE

WEB OF SCIENCE BASE SEARCH	TS=("alcohol" OR "alcoholic beverage*" OR "drinking behavior*" OR "drinking behaviour*") AND TS=("risk" OR "odds ratio" OR "cross-product ratio" OR "hazards ratio" OR "hazard ratio") AND (ALL="Cohort Studies" OR ALL="Prospective Studies" OR ((TI=cohort OR AB=cohort) AND ((TI=prospective OR AB=prospective) OR (TI=pooled OR AB=pooled) OR (TI=follow-up OR AB=follow-up) OR (TI=meta-analysis OR AB=meta-analysis))) OR ALL="Case-Control Studies" OR (TI=case-control OR AB=case-control) OR (TI="case control" OR AB="case control") OR (TI=case-crossover OR AB=case-crossover)) NOT ((TI=cross-sectional OR AB=cross-sectional) OR ((TI=cohort OR AB=cohort) AND (TI=retrospective OR AB=retrospective))) AND
**Atrial fibrillation**	(ALL="Atrial Fibrillation" OR ALL="Atrial Flutter" OR (TI="atrial fibrillation*" OR AB="atrial fibrillation*") OR (TI="atrial fibrilation*" OR AB="atrial fibrilation*") OR (TI="auricular fibrillation*" OR AB="auricular fibrillation*") OR (TI="auricular fibrilation*" OR AB="auricular fibrilation*") OR (TI="atrial flutter*" OR AB="atrial flutter*") OR (TI="atrium flutter*" OR AB="atrium flutter*") OR (TI="auricular flutter*" OR AB="auricular flutter*"))
**Breast cancer**	(ALL="Breast Neoplasm" OR (TI="breast cancer*" OR AB="breast cancer*") OR (TI="breast neoplasm*" OR AB="breast neoplasm*") OR (TI="breast carcinoma*" OR AB="breast carcinoma*") OR (TI="breast tumor*" OR AB="breast tumor*") OR (TI="breast tumour*" OR AB="breast tumour*") OR (TI="mammary cancer*" OR AB="mammary cancer*") OR (TI="mammary neoplasm*" OR AB="mammary neoplasm*") OR (TI="mammary carcinoma*" OR AB="mammary carcinoma*") OR (TI="mammary tumor*" OR AB="mammary tumor*") OR (TI="mammary tumour*" OR AB="mammary tumour*") OR (TI="breast malignant neoplasm*" OR AB="breast malignant neoplasm*") OR (TI="cancer of the breast" OR AB="cancer of the breast") OR (TI="cancers of the breast" OR AB="cancers of the breast") OR (TI="neoplasm of the breast" OR AB="neoplasm of the breast") OR (TI="neoplasms of the breast" OR AB="neoplasms of the breast") OR (TI="tumor of the breast" OR AB="tumor of the breast") OR (TI="tumors of the breast" OR AB="tumors of the breast") OR (TI="tumour of the breast" OR AB="tumour of the breast") OR (TI="tumours of the breast" OR AB="tumours of the breast"))
**Cirrhosis and other chronic liver diseases**	(ALL="Liver Cirrhosis" OR ALL="End Stage Liver Disease" OR (TI=cirrhosis OR AB=cirrhosis) OR (TI=cirrhoses OR AB=cirrhoses) OR (TI="liver fibrosis" OR AB="liver fibrosis") OR (TI="liver fibroses" OR AB="liver fibroses") OR (TI="chronic liver failure" OR AB="chronic liver failure") OR (TI="chronic hepatic failure'" OR AB="chronic hepatic failure'") OR (TI="end stage liver disease" OR AB="end stage liver disease") OR (TI="chronic liver disease" OR AB="chronic liver disease"))
**Colorectal cancer**	(ALL="Colorectal Neoplasm" OR (TI="colorectal cancer*" OR AB="colorectal cancer*") OR (TI="colorectal neoplasm*" OR AB="colorectal neoplasm*") OR (TI="colorectal tumor*" OR AB="colorectal tumor*") OR (TI="colorectal tumour*" OR AB="colorectal tumour*") OR (TI="colorectal carcinoma*" OR AB="colorectal carcinoma*") OR (TI="colonic neoplasm*" OR AB="colonic neoplasm*") OR (TI="colonic cancer*" OR AB="colonic cancer*") OR

	(TI="colon cancer*" OR AB="colon cancer*") OR (TI="colon neoplasm*" OR AB="colon neoplasm*") OR (TI="rectal cancer*" OR AB="rectal cancer*") OR (TI="rectal neoplasm*" OR AB="rectal neoplasm*") OR (TI="rectal tumor*" OR AB="rectal tumor*") OR (TI="rectal tumour*" OR AB="rectal tumour*") OR (TI="rectum neoplasm*" OR AB="rectum neoplasm*") OR (TI="rectum cancer*" OR AB="rectum cancer*") OR (TI="rectal tumor*" OR AB="rectal tumor*") OR (TI="rectal tumour*" OR AB="rectal tumour*") OR (TI="cancer of the colon" OR AB="cancer of the colon") OR (TI="cancers of the colon" OR AB="cancers of the colon") OR (TI="cancer of the rectum" OR AB="cancer of the rectum") OR (TI="cancers of the rectum" OR AB="cancers of the rectum") OR (TI="bowel cancer" OR AB="bowel cancer") OR (TI="large bowel cancer" OR AB="large bowel cancer"))
**Alzheimer's disease and other dementias**	TI=("dementia" OR "cognition" OR "cognitive decline" OR "alzheimer disease" OR "Alzheimer")
**Type 2 diabetes**	(ALL="Diabetes Mellitus, Type 2" OR (TI=diabetes OR AB=diabetes))
**Esophageal cancer**	((TI="esophageal cancer*" OR AB="esophageal cancer*") OR (TI="esophageal neoplasm*" OR AB="esophageal neoplasm*") OR (TI="esophagus cancer*" OR AB="esophagus cancer*") OR (TI="esophagus neoplasm*" OR AB="esophagus neoplasm*") OR (TI="esophageal squamous cell carcinoma" OR AB="esophageal squamous cell carcinoma") OR (TI="cancer of the esophagus" OR AB="cancer of the esophagus") OR (TI="cancers of the esophagus" OR AB="cancers of the esophagus") OR (TI="neoplasm of the esophagus" OR AB="neoplasm of the esophagus") OR (TI="neoplasms of the esophagus" OR AB="neoplasms of the esophagus") OR (TI="oesophageal cancer*" OR AB="oesophageal cancer*") OR (TI="oesophageal neoplasm*" OR AB="oesophageal neoplasm*") OR (TI="oesophagus cancer*" OR AB="oesophagus cancer*") OR (TI="oesophagus neoplasm*" OR AB="oesophagus neoplasm*") OR (TI="oesophageal squamous cell carcinoma" OR AB="oesophageal squamous cell carcinoma") OR (TI="cancer of the oesophagus" OR AB="cancer of the oesophagus") OR (TI="cancers of the oesophagus" OR AB="cancers of the oesophagus") OR (TI="neoplasm of the oesophagus" OR AB="neoplasm of the oesophagus") OR (TI="neoplasms of the oesophagus" OR AB="neoplasms of the oesophagus"))
**Larynx cancer**	((TI="laryngeal cancer*" OR AB="laryngeal cancer*") OR (TI="laryngeal neoplasm*" OR AB="laryngeal neoplasm*") OR (TI="larynx cancer*" OR AB="larynx cancer*") OR (TI="larynx neoplasm*" OR AB="larynx neoplasm*") OR (TI="cancer of the larynx" OR AB="cancer of the larynx") OR (TI="cancers of the larynx" OR AB="cancers of the larynx") OR (TI="neoplasm of the larynx" OR AB="neoplasm of the larynx") OR (TI="neoplasms of the larynx" OR AB="neoplasms of the larynx"))
**Mouth cancer**	((TI="mouth cancer*" OR AB="mouth cancer*") OR (TI="mouth neoplasm*" OR AB="mouth neoplasm*") OR (TI="cancer of mouth" OR AB="cancer of mouth") OR (TI="cancer of the mouth" OR AB="cancer of the mouth") OR (TI="cancers of the mouth" OR AB="cancers of the mouth") OR (TI="oral cancer*" OR AB="oral cancer*") OR (TI="oral neoplasm*" OR AB="oral

	neoplasm*") OR (TI="gingival neoplasm*" OR AB="gingival neoplasm*") OR (TI="gingival cancer*" OR AB="gingival cancer*"))
**Other pharynx cancer**	((TI="pharyngeal cancer*" OR AB="pharyngeal cancer*") OR (TI="pharyngeal neoplasm*" OR AB="pharyngeal neoplasm*") OR (TI="pharynx cancer*" OR AB="pharynx cancer*") OR (TI="pharynx neoplasm*" OR AB="pharynx neoplasm*") OR (TI="cancer of the pharynx" OR AB="cancer of the pharynx") OR (TI="cancers of the pharynx" OR AB="cancers of the pharynx"))
**Ischemic heart disease**	(ALL="Coronary Artery Disease" OR ALL="Myocardial Ischemia" OR ALL=atherosclerosis OR (TI="coronary artery disease" OR AB="coronary artery disease") OR (TI="myocardial ischemia" OR AB="myocardial ischemia") OR (TI="myocardial ischaemia" OR AB="myocardial ischaemia") OR (TI="cardiac ischemia" OR AB="cardiac ischemia") OR (TI="cardiac ischaemia" OR AB="cardiac ischaemia") OR (TI="silent ischemia" OR AB="silent ischemia") OR (TI="silent ischaemia" OR AB="silent ischaemia") OR (TI=atherosclerosis OR AB=atherosclerosis) OR (TI="ischaemic heart disease" OR AB="ischaemic heart disease") OR (TI="ischemic heart disease" OR AB="ischemic heart disease") OR (TI="coronary heart disease" OR AB="coronary heart disease") OR (TI="myocardial infarct*" OR AB="myocardial infarct*") OR (TI="heart attack" OR AB="heart attack") OR (TI="heart infarct*" OR AB="heart infarct*") OR (TI="cardiac infarct*" OR AB="cardiac infarct*"))
**Liver cancer**	(ALL="Liver Neoplasm" OR ALL=cholangiocarcinoma OR (TI="liver cancer*" OR AB="liver cancer*") OR (TI="cancer of the liver" OR AB="cancer of the liver") OR (TI="cancers of the liver" OR AB="cancers of the liver") OR (TI="liver neoplasm*" OR AB="liver neoplasm*") OR (TI="hepatic cancer*" OR AB="hepatic cancer*") OR (TI="hepatic neoplasm*" OR AB="hepatic neoplasm*") OR (TI="hepatocellular neoplasm*" OR AB="hepatocellular neoplasm*") OR (TI="hepatocellular cancer*" OR AB="hepatocellular cancer*") OR (TI="liver cell carcinoma*" OR AB="liver cell carcinoma*") OR (TI=hepatoma* OR AB=hepatoma*) OR (TI="hepatocellular carcinoma*" OR AB="hepatocellular carcinoma*") OR (TI="hepatic carcinoma*" OR AB="hepatic carcinoma*") OR (TI=cholangiocarcinoma* OR AB=cholangiocarcinoma*) OR (TI="intrahepatic bile duct carcinoma*" OR AB="intrahepatic bile duct carcinoma*"))
**Lower respiratory infections**	(ALL=pneumonia OR ALL=bronchiolitis OR (TI="lower respiratory" OR AB="lower respiratory") OR (TI=pneumonia OR AB=pneumonia) OR (TI=bronchiolitis OR AB=bronchiolitis) OR (TI="pulmonary inflammation" OR AB="pulmonary inflammation") OR (TI="lung inflammation" OR AB="lung inflammation"))
**Pancreatitis**	(ALL=Pancreatitis OR (TI=pancreatitis OR AB=pancreatitis) OR (TI=pancreatitides OR AB=pancreatitides))
**Pancreatic cancer**	(ALL="Pancreatic Neoplasm" OR (TI="pancreas cancer*" OR AB="pancreas cancer*") OR (TI="pancreas neoplasm*" OR AB="pancreas neoplasm*") OR (TI="pancreas carcinoma*" OR AB="pancreas carcinoma*") OR (TI="pancreas tumor*" OR AB="pancreas tumor*") OR (TI="pancreas tumour*" OR AB="pancreas tumour*") OR (TI="pancreatic cancer*" OR AB="pancreatic cancer*") OR (TI="pancreatic neoplasm*" OR AB="pancreatic neoplasm*") OR (TI="pancreatic carcinoma*" OR AB="pancreatic carcinoma*") OR

	(TI="pancreatic tumor*" OR AB="pancreatic tumor*") OR (TI="pancreatic tumour*" OR AB="pancreatic tumour*") OR (TI="pancreatic malignant neoplasm*" OR AB="pancreatic malignant neoplasm*") OR (TI="cancer of the pancreas" OR AB="cancer of the pancreas") OR (TI="cancers of the pancreas" OR AB="cancers of the pancreas") OR (TI="neoplasm of the pancreas" OR AB="neoplasm of the pancreas") OR (TI="neoplasms of the pancreas" OR AB="neoplasms of the pancreas") OR (TI="tumor of the pancreas" OR AB="tumor of the pancreas") OR (TI="tumors of the pancreas" OR AB="tumors of the pancreas") OR (TI="tumour of the pancreas" OR AB="tumour of the pancreas") OR (TI="tumours of the pancreas" OR AB="tumours of the pancreas"))
**Prostate cancer**	(ALL="Prostatic Neoplasms" OR (TI="prostate cancer*" OR AB="prostate cancer*") OR (TI="prostate neoplasm*" OR AB="prostate neoplasm*") OR (TI="prostate carcinoma*" OR AB="prostate carcinoma*") OR (TI="prostate tumor*" OR AB="prostate tumor*") OR (TI="prostate tumour*" OR AB="prostate tumour*") OR (TI="prostate tumor*" OR AB="prostate tumor*") OR (TI="prostate tumour*" OR AB="prostate tumour*") OR (TI="prostate malignant neoplasm*" OR AB="prostate malignant neoplasm*") OR (TI="cancer of the prostate" OR AB="cancer of the prostate") OR (TI="cancers of the prostate" OR AB="cancers of the prostate") OR (TI="neoplasm of the prostate" OR AB="neoplasm of the prostate") OR (TI="neoplasms of the prostate" OR AB="neoplasms of the prostate") OR (TI="tumor of the prostate" OR AB="tumor of the prostate") OR (TI="tumors of the prostate" OR AB="tumors of the prostate") OR (TI="tumour of the prostate" OR AB="tumour of the prostate") OR (TI="tumours of the prostate" OR AB="tumours of the prostate") OR (KP="neoplasms, prostate" OR AK="neoplasms, prostate") OR (KP="neoplasms, prostatic" OR AK="neoplasms, prostatic"))
**Stomach cancer**	(ALL="Stomach Neoplasms" OR ALL="Gastric Neoplasms" OR (TI="stomach cancer*" OR AB="stomach cancer*") OR (TI="stomach neoplasm*" OR AB="stomach neoplasm*") OR (TI="stomach carcinoma*" OR AB="stomach carcinoma*") OR (TI="stomach tumor*" OR AB="stomach tumor*") OR (TI="stomach tumour*" OR AB="stomach tumour*") OR (TI="gastric cancer*" OR AB="gastric cancer*") OR (TI="gastric neoplasm*" OR AB="gastric neoplasm*") OR (TI="gastric carcinoma*" OR AB="gastric carcinoma*") OR (TI="gastric tumor*" OR AB="gastric tumor*") OR (TI="gastric tumour*" OR AB="gastric tumour*") OR (TI="gastric malignant neoplasm*" OR AB="gastric malignant neoplasm*") OR (TI="cancer of the stomach" OR AB="cancer of the stomach") OR (TI="cancers of the stomach" OR AB="cancers of the stomach") OR (TI="neoplasm of the stomach" OR AB="neoplasm of the stomach") OR (TI="neoplasms of the stomach" OR AB="neoplasms of the stomach") OR (TI="tumor of the stomach" OR AB="tumor of the stomach") OR (TI="tumors of the stomach" OR AB="tumors of the stomach") OR (TI="tumour of the stomach" OR AB="tumour of the stomach") OR (TI="tumours of the stomach" OR AB="tumours of the stomach"))
**Ischemic & Hemorrhagic Stroke**	(ALL="brain infarction" OR ALL=stroke OR ALL="intracranial hemorrhages" OR (TI=stroke OR AB=stroke) OR (TI="cerebrovascular accident" OR AB="cerebrovascular accident") OR (TI="brain infarct*" OR AB="brain infarct*") OR (TI="cerebral infarct*" OR AB="cerebral infarct*") OR (TI="cortex

	infarct*" OR AB="cortex infarct*") OR (TI="cerebrovascular infarct*" OR AB="cerebrovascular infarct*") OR (TI="cortical infarct*" OR AB="cortical infarct*") OR (TI="hemisphere infarct*" OR AB="hemisphere infarct*") OR (TI="hemispheric infarct*" OR AB="hemispheric infarct*") OR (TI="intracerebral hemorrhage" OR AB="intracerebral hemorrhage") OR (TI="intracerebral haemorrhage" OR AB="intracerebral haemorrhage") OR (TI="subarachnoid hemorrhage" OR AB="subarachnoid hemorrhage") OR (TI="subarachnoid haemorrhage" OR AB="subarachnoid haemorrhage") OR (TI="brain hemorrhage" OR AB="brain hemorrhage") OR (TI="brain haemorrhage" OR AB="brain haemorrhage"))
**Tuberculosis**	(ALL=tuberculosis OR ALL="Mycobacterium tuberculosis" OR (TI=tuberculosis OR AB=tuberculosis) OR (TI=TB OR AB=TB))

C. Best Systematic Reviews

Table S7. Best systematic reviews

Outcome	Most recent high-quality systematic review for each outcome	Start date of review	# underlying sources
**Atrial fibrillation**	Alcohol and incident atrial fibrillation – A systematic review and meta-analysis Gallagher, Celine et al. International Journal of Cardiology, Volume 246, 46 - 52	2/1/2016	30
**Breast cancer**	Poorolajal J, Heidarimoghis F, Karami M, Cheraghi Z, Gohari-Ensaf F, Shahbazi F, Zareie B, Ameri P, Sahraee F. Factors for the Primary Prevention of Breast Cancer: A Meta-Analysis of Prospective Cohort Studies. J Res Health Sci. 2021 Jul 20;21(3):e00520. doi: 10.34172/jrhs.2021.57. PMID: 34698654; PMCID: PMC8957681.	1/1/2021	53
**Cirrhosis and other chronic liver diseases**	Glyn-Owen, K., Böhning, D., Parkes, J., Roderick, P., Buchanan, R., 2021. The combined effect of alcohol and body mass index on risk of chronic liver disease: A systematic review and meta-analysis of cohort studies. Liver International 41, 1216–1226.. https://doi.org/10.1111/liv.14754	6/17/2020	9
**Colorectal cancer**	Ubago-Guisado, E.; Rodríguez-Barranco, M.; ChingLópez, A.; Petrova, D.; MolinaMontes, E.; Amiano, P.; BarricarteGurrea, A.; Chirlaque, M.-D.; Agudo, A.; Sánchez, M.-J. Evidence Update on the Relationship between Diet and the Most Common Cancers from the European Prospective Investigation into Cancer and Nutrition (EPIC) Study: A Systematic Review. Nutrients 2021, 13, 3582. https:// doi.org/10.3390/nu13103582	3/1/2021	110
**Alzheimer's disease and other dementias**	Dementia prevention, intervention, and care: 2020 report of the Lancet Commission Livingston, Gill et al. The Lancet, Volume 396, Issue 10248, 413 - 446	9/8/2018	7
**Type 2 diabetes**	Schlesinger S, Neuenschwander M, Ballon A, et al. J Epidemiol Community Health 2020;74:481–487.	9/1/2019	19
**Head and neck cancers**			

**Esophageal cancer**	Sun LP, Yan LB, Liu ZZ, Zhao WJ, Zhang CX, Chen YM, Lao XQ, Liu X. Dietary factors and risk of mortality among patients with esophageal cancer: a systematic review. BMC Cancer. 2020 Apr 6;20(1):287. doi: 10.1186/s12885-020-06767-8. PMID: 32252671; PMCID: PMC7137267.	1/1/2020	15
**Larynx cancer**	Farhad Islami, Irene Tramacere, Matteo Rota, Vincenzo Bagnardi, Veronika Fedirko, Lorenza Scotti, Werner Garavello, Mazda Jenab, Giovanni Corrao, Kurt Straif, Eva Negri, Paolo Boffetta, Carlo La Vecchia,<br>Alcohol drinking and laryngeal cancer: Overall and dose–risk relation – A systematic review and meta-analysis,<br>Oral Oncology,<br>Volume 46, Issue 11,<br>2010,<br>Pages 802-810,<br>ISSN 1368-8375,<br>https://doi.org/10.1016/j.oraloncology.2010.07.015.<br>(https://www.sciencedirect.com/science/article/pii/S1368837510002319)	5/13/2010	40
**Mouth cancer**	Bagnardi, V., Rota, M., Botteri, E. et al. Alcohol consumption and site-specific cancer risk: a comprehensive dose–response meta-analysis. Br J Cancer 112, 580–593 (2015). https://doi.org/10.1038/bjc.2014.579	7/9/2021	572
**Other pharynx cancer**	Batistella, E.Â., Gondak, R., Rivero, E.R.C. et al. Comparison of tobacco and alcohol consumption in young and older patients with oral squamous cell carcinoma: a systematic review and meta-analysis. Clin Oral Invest 26, 6855–6869 (2022). https://doi-org.offcampus.lib.washington.edu/10.1007/s00784-022-04719-z	1/1/2013	38
**Ischemic heart disease**	Ding, C., O'Neill, D., Bell, S. et al. Association of alcohol consumption with morbidity and mortality in patients with cardiovascular disease: original data and meta-analysis of 48,423 men and women. BMC Med 19, 167 (2021). https://doi.org/10.1186/s12916-021-02040-2	7/30/2021	14
**Liver cancer**	Park, H., Shin, S.K., Joo, I., Song, D.S., Jang, J.W., Park, J.-W., 2020. Systematic Review with	7/1/2019	28

	Meta-Analysis: Low-Level Alcohol Consumption and the Risk of Liver Cancer. Gut and Liver 14, 792–807.. https://doi.org/10.5009/gnl19163		
**Lower respiratory infections**	Wood, S., Harrison, S.E., Judd, N., Bellis, M.A., Hughes, K., Jones, A., 2021. The impact of behavioural risk factors on communicable diseases: a systematic review of reviews. BMC Public Health 21.. https://doi.org/10.1186/s12889-021-12148-y	10/28/2020	14
**Pancreatitis**	Samokhvalov AV, Rehm J, Roerecke M. Alcohol Consumption as a Risk Factor for Acute and Chronic Pancreatitis: A Systematic Review and a Series of Meta-analyses. EBioMedicine. 2015 Nov 14;2(12):1996-2002. doi: 10.1016/j.ebiom.2015.11.023. PMID: 26844279; PMCID: PMC4703772.	5/1/2015	3
**Pancreatic cancer**	Lu, P.-Y., Shu, L., Shen, S.-S., Chen, X.-J., Zhang, X.-Y., 2017. Dietary Patterns and Pancreatic Cancer Risk: A Meta-Analysis. Nutrients 9, 38.. https://doi.org/10.3390/nu9010038	5/1/2016	32
**Prostate cancer**	Hong, S., Khil, H., Lee, D.H., Keum, N., Giovannucci, E.L., 2020. Alcohol Consumption and the Risk of Prostate Cancer: A Dose-Response Meta-Analysis. Nutrients 12, 2188.. https://doi.org/10.3390/nu12082188	4/1/2020	11
**Stomach cancer**	Wenting Deng, Lan Jin, Haoran Zhuo, Vasilis Vasiliou, Yawei Zhang,<br>Alcohol consumption and risk of stomach cancer: A meta-analysis,<br>Chemico-Biological Interactions,<br>Volume 336,<br>2021,<br>109365,<br>ISSN 0009-2797,<br>https://doi.org/10.1016/j.cbi.2021.109365.<br>(https://www.sciencedirect.com/science/article/pii/S0009279721000016)	6/1/2019	81
**Ischemic & Hemorrhagic Stroke**	Wang S, Zou XL, Wu LX, Zhou HF, Xiao L, Yao T, Zhang Y, Ma J, Zeng Y, Zhang L. Epidemiology of intracerebral hemorrhage: A systematic review and meta-analysis. Front Neurol. 2022 Sep 16;13:915813. doi: 10.3389/fneur.2022.915813. PMID: 36188383; PMCID: PMC9523083.	1/1/2020	52

**Tuberculosis**	Wood, S., Harrison, S.E., Judd, N., Bellis, M.A., Hughes, K., Jones, A., 2021. The impact of behavioural risk factors on communicable diseases: a systematic review of reviews. BMC Public Health 21.. https://doi.org/10.1186/s12889-021-12148-y	10/28/2020	44

V. Prisma Flow Diagrams

Figure S1.01. Atrial fibrillation

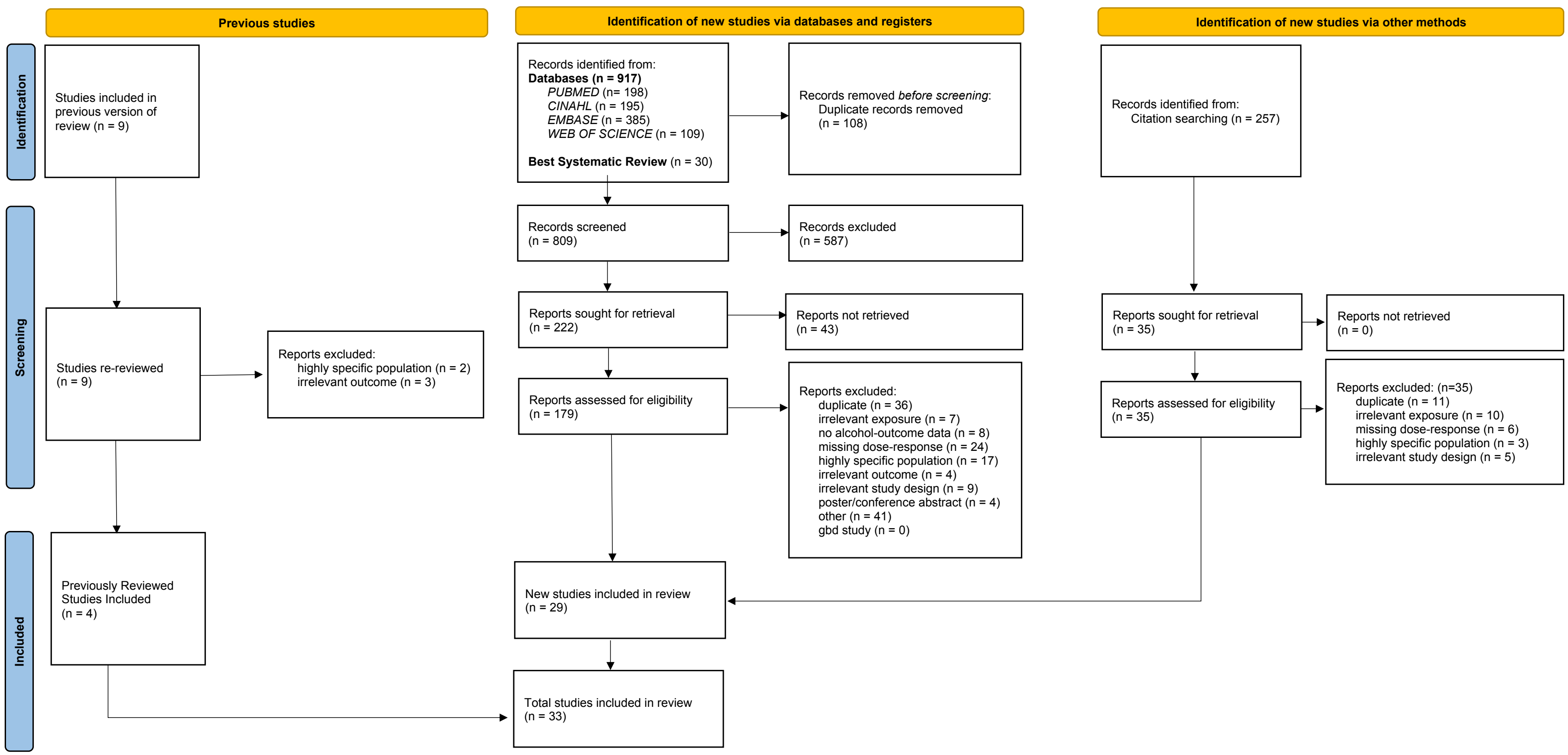

Figure S1.02. Breast cancer

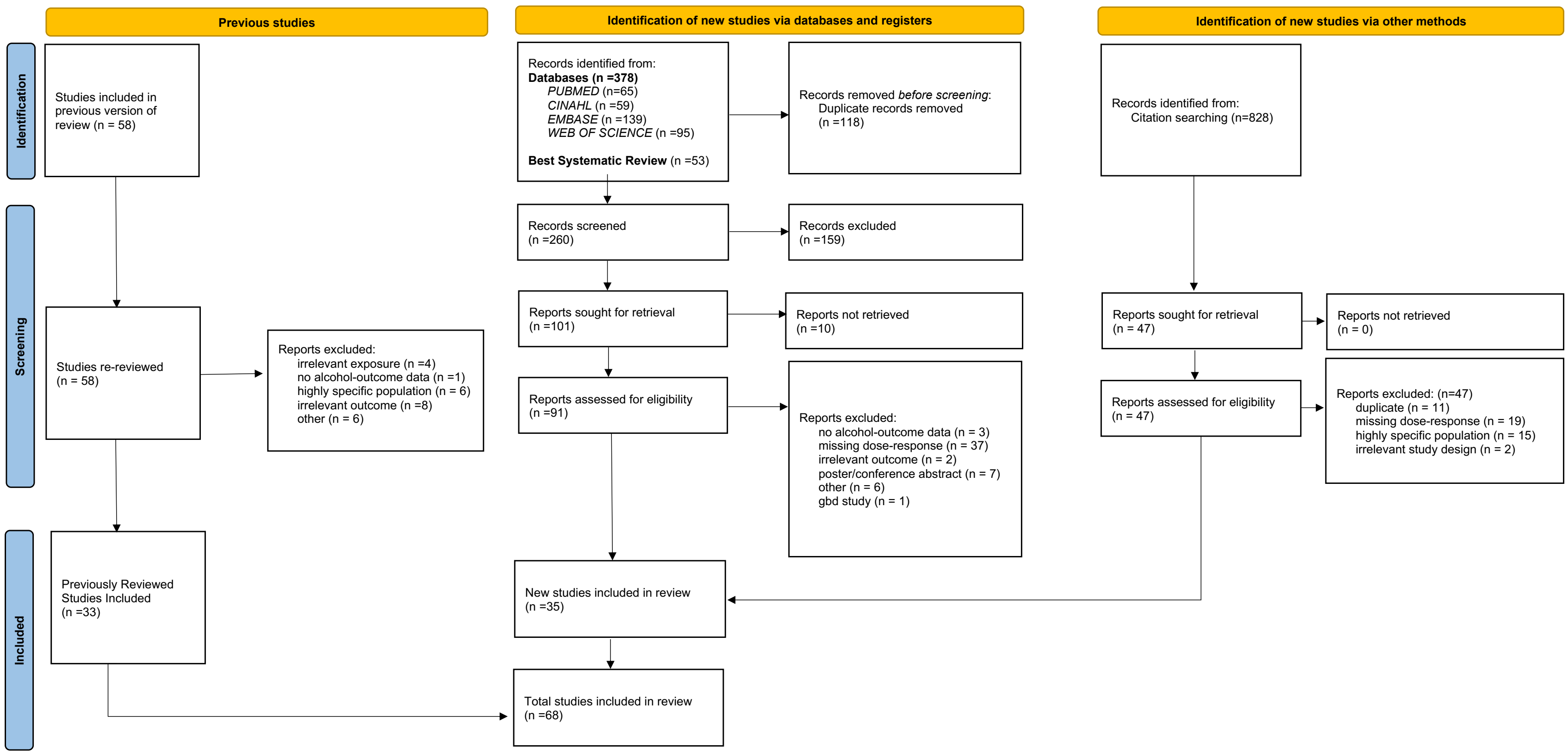

Figure S1.03. Cirrhosis and other chronic liver diseases

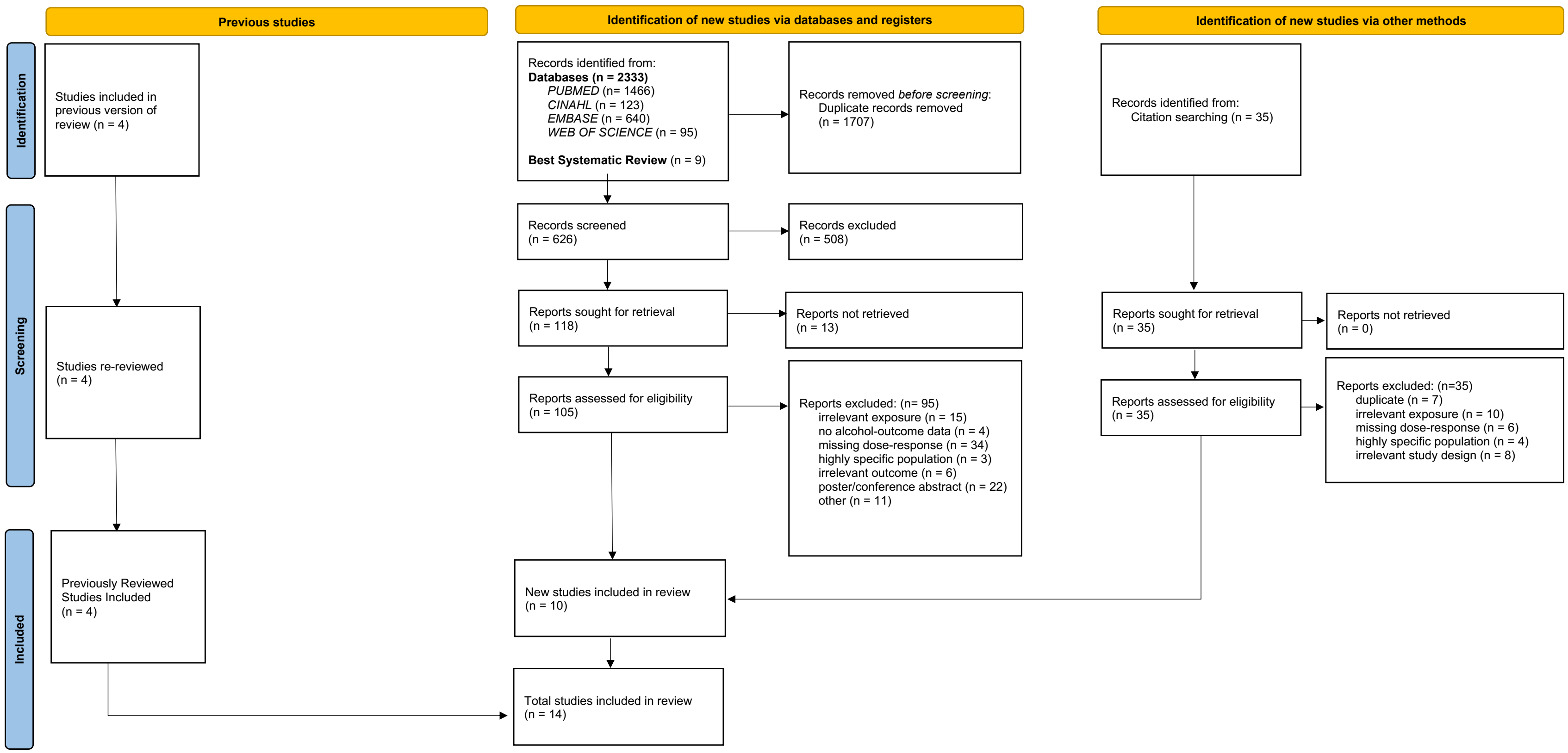

Figure S1.04. Colorectal cancer

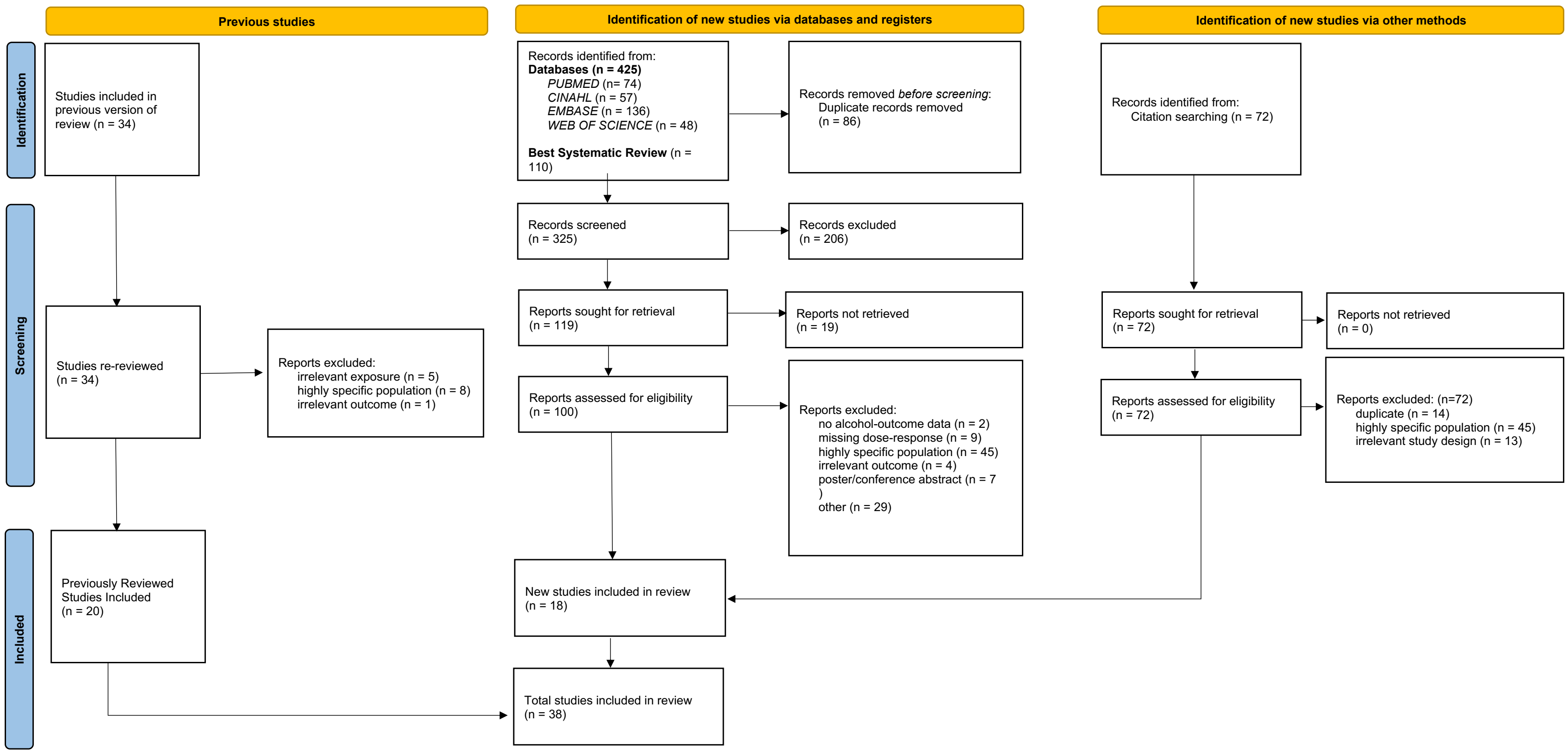

Figure S1.05. Alzheimer's disease and other dementias

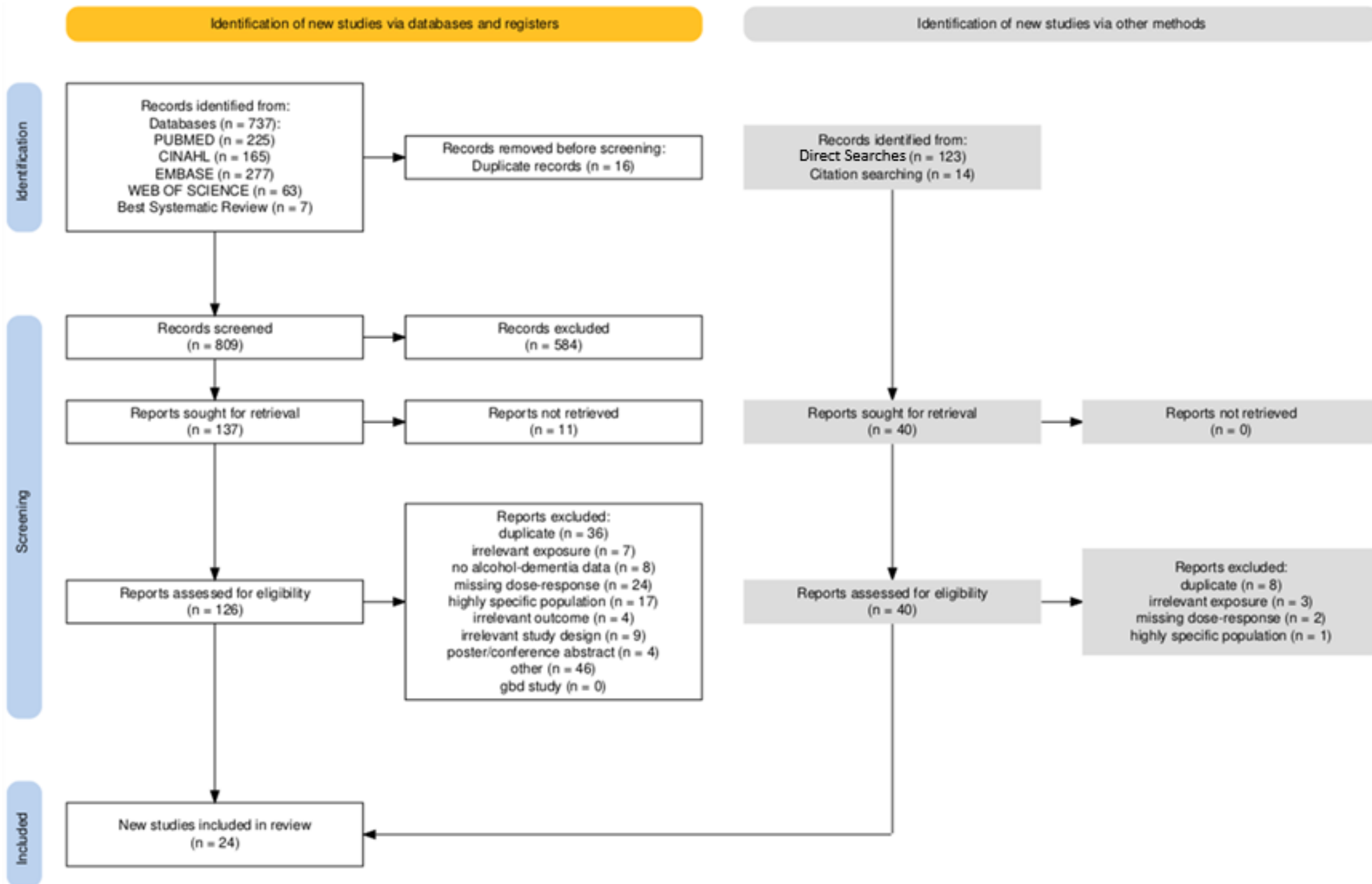

Figure S1.06. Type 2 diabetes

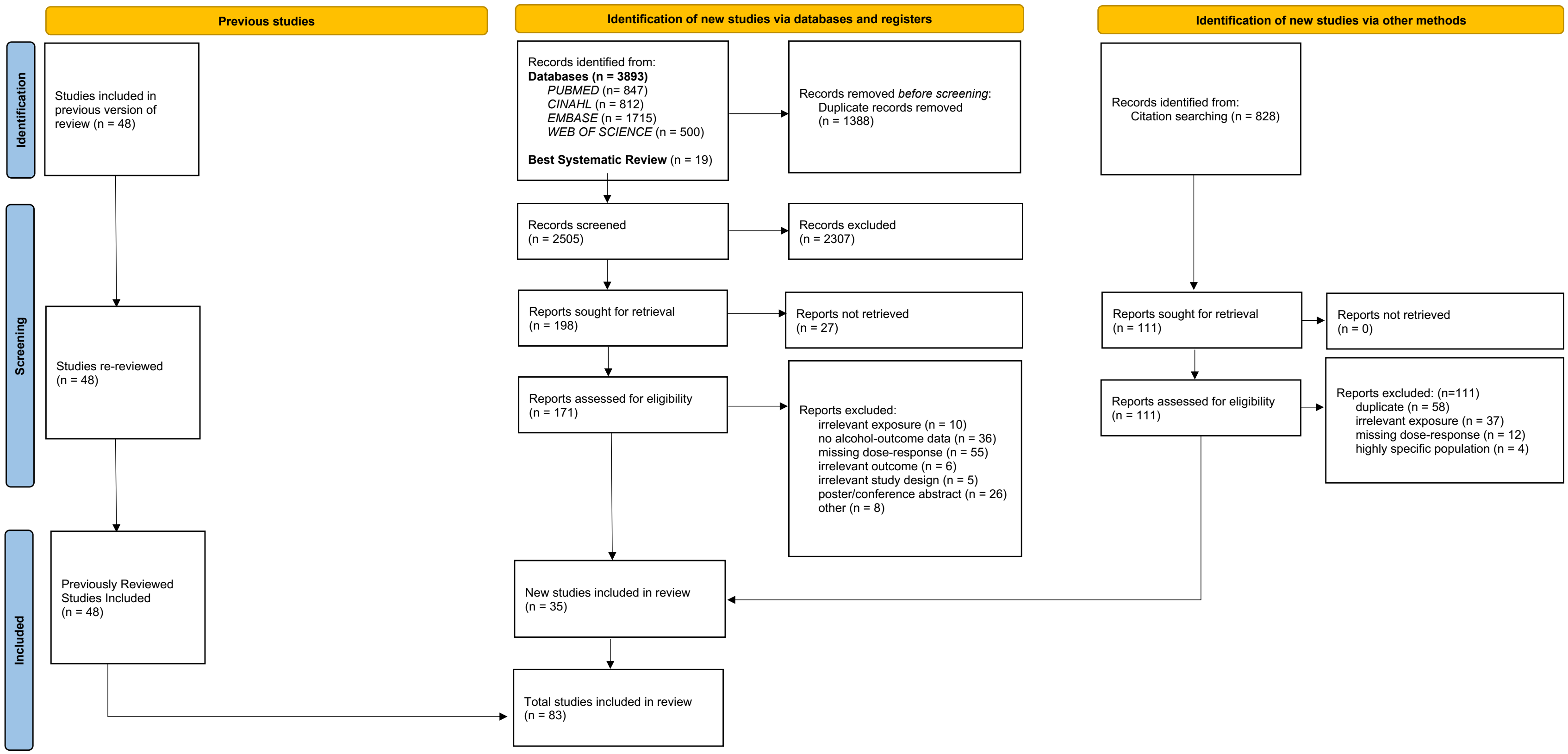

Figure S1.07. Head and Neck Cancers

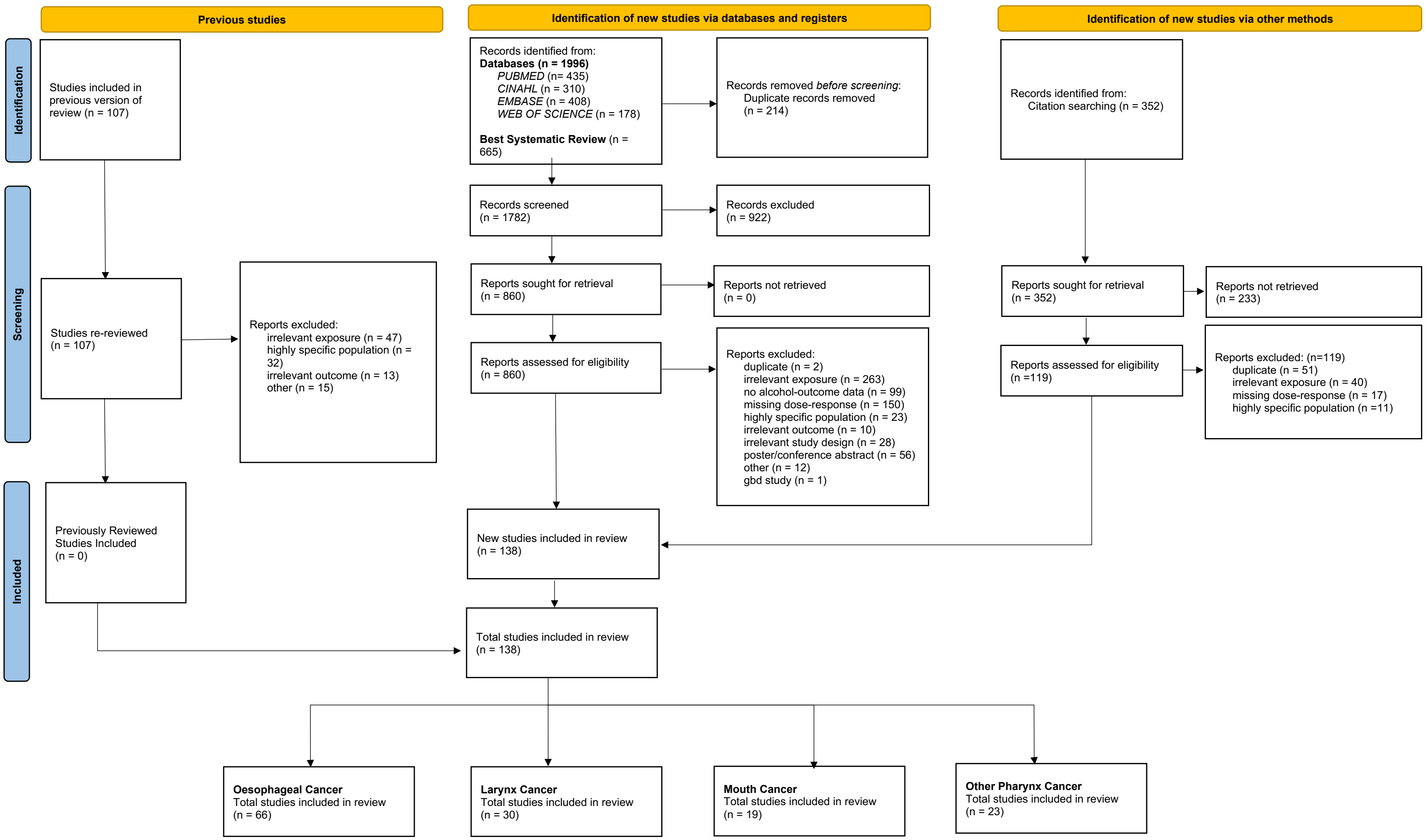

Figure S1.08. Ischemic heart disease

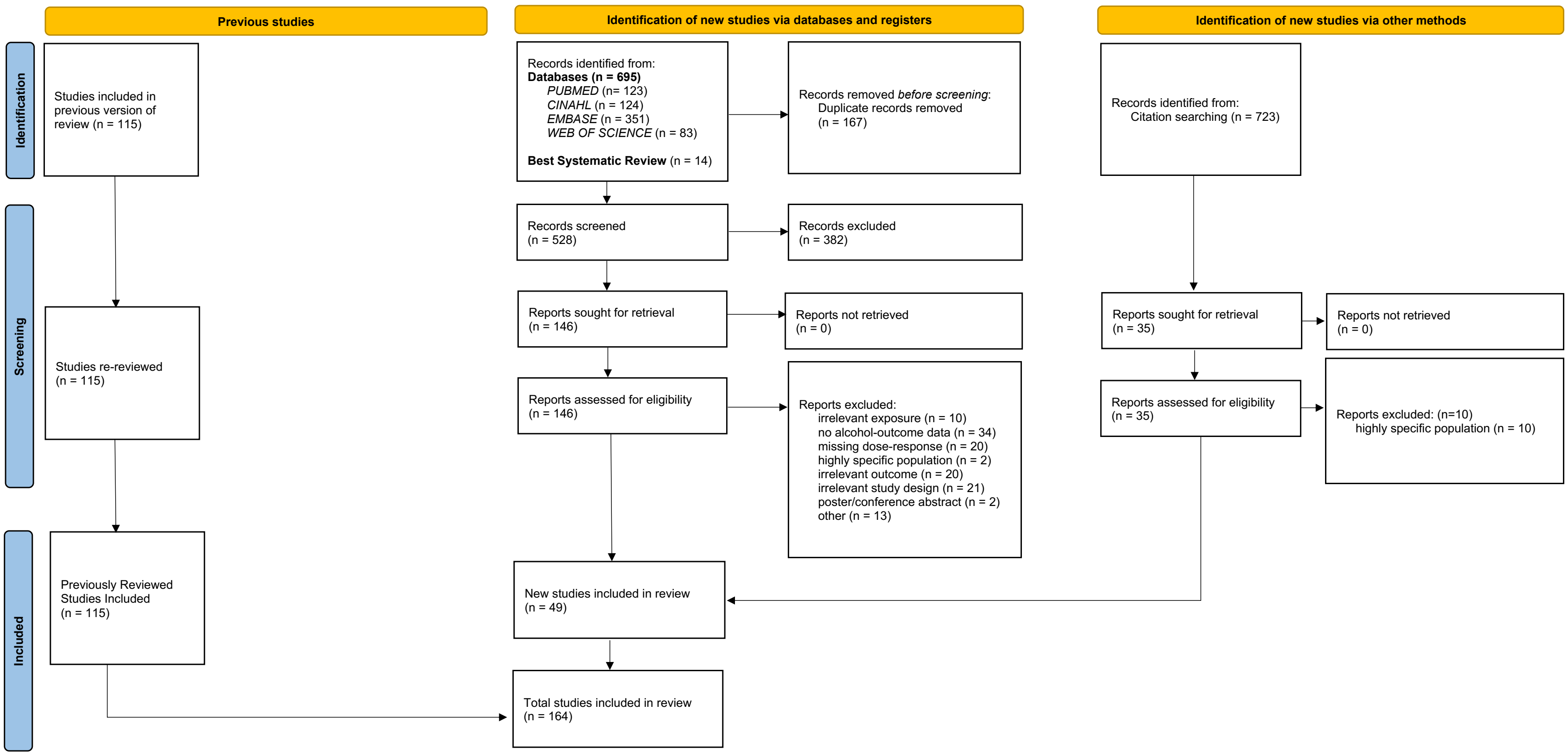

Figure S1.09. Liver cancer

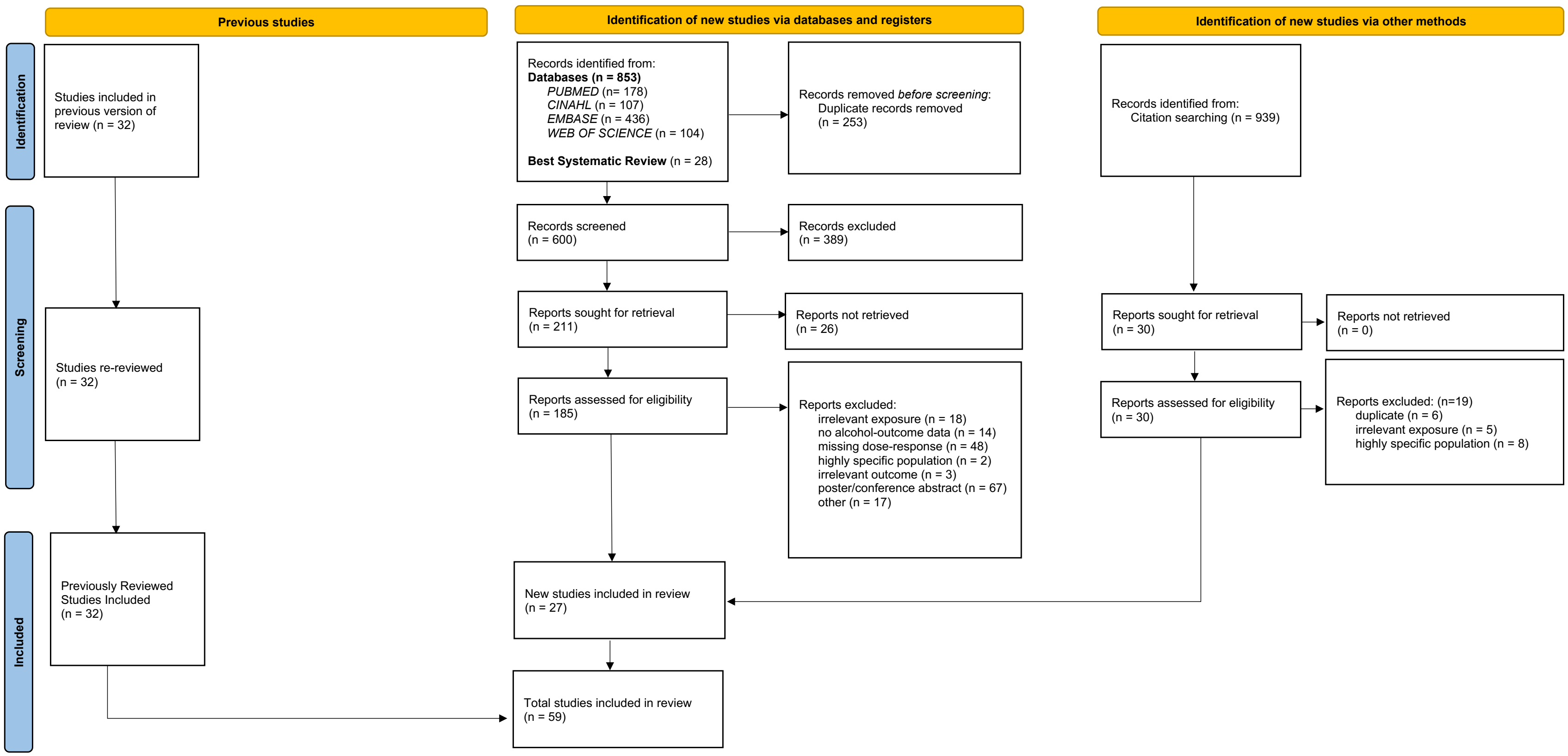

Figure S1.10. Lower respiratory infections

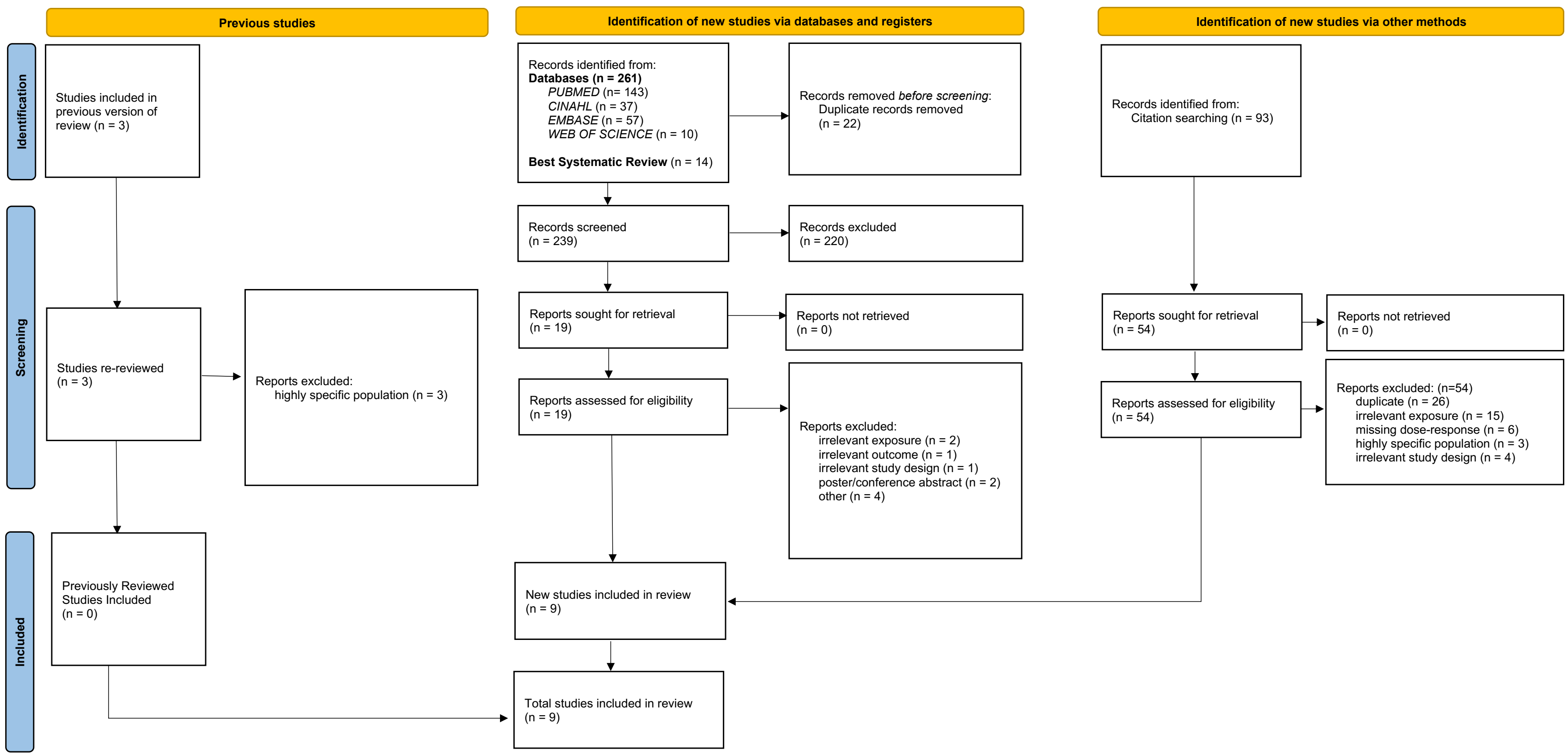

Figure S1.11. Pancreatitis

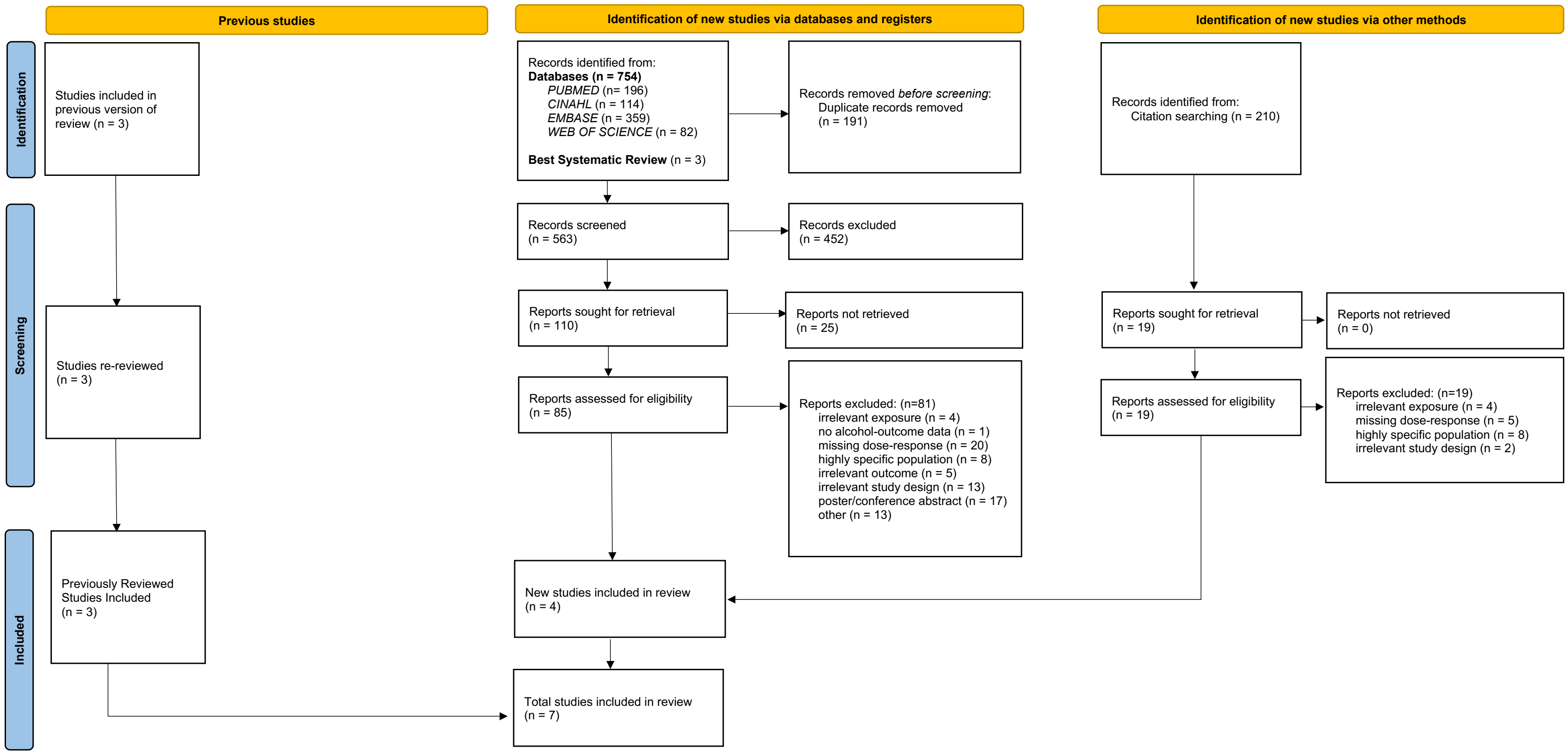

Figure S1.12. Pancreatic cancer

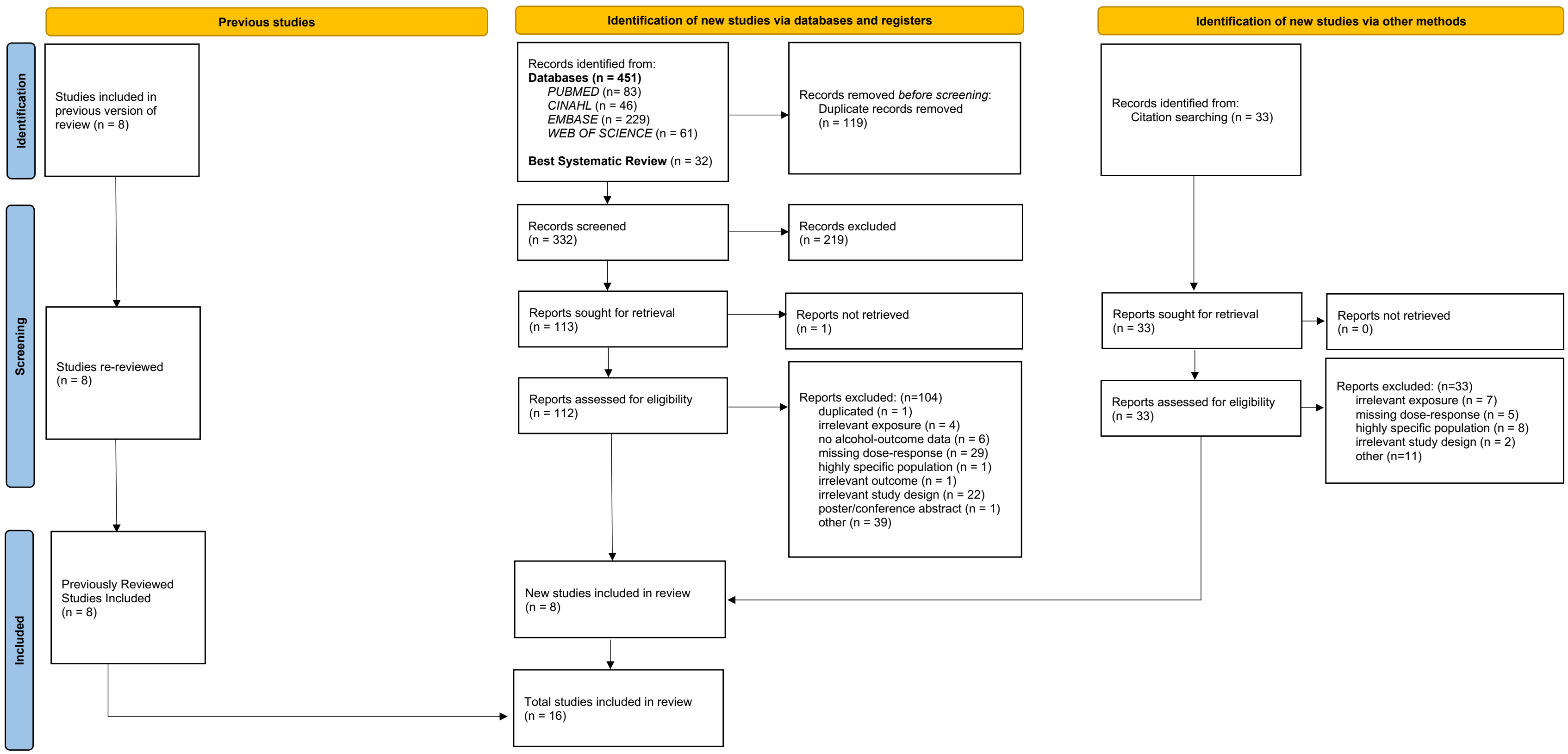

Figure S1.13. Prostate cancer

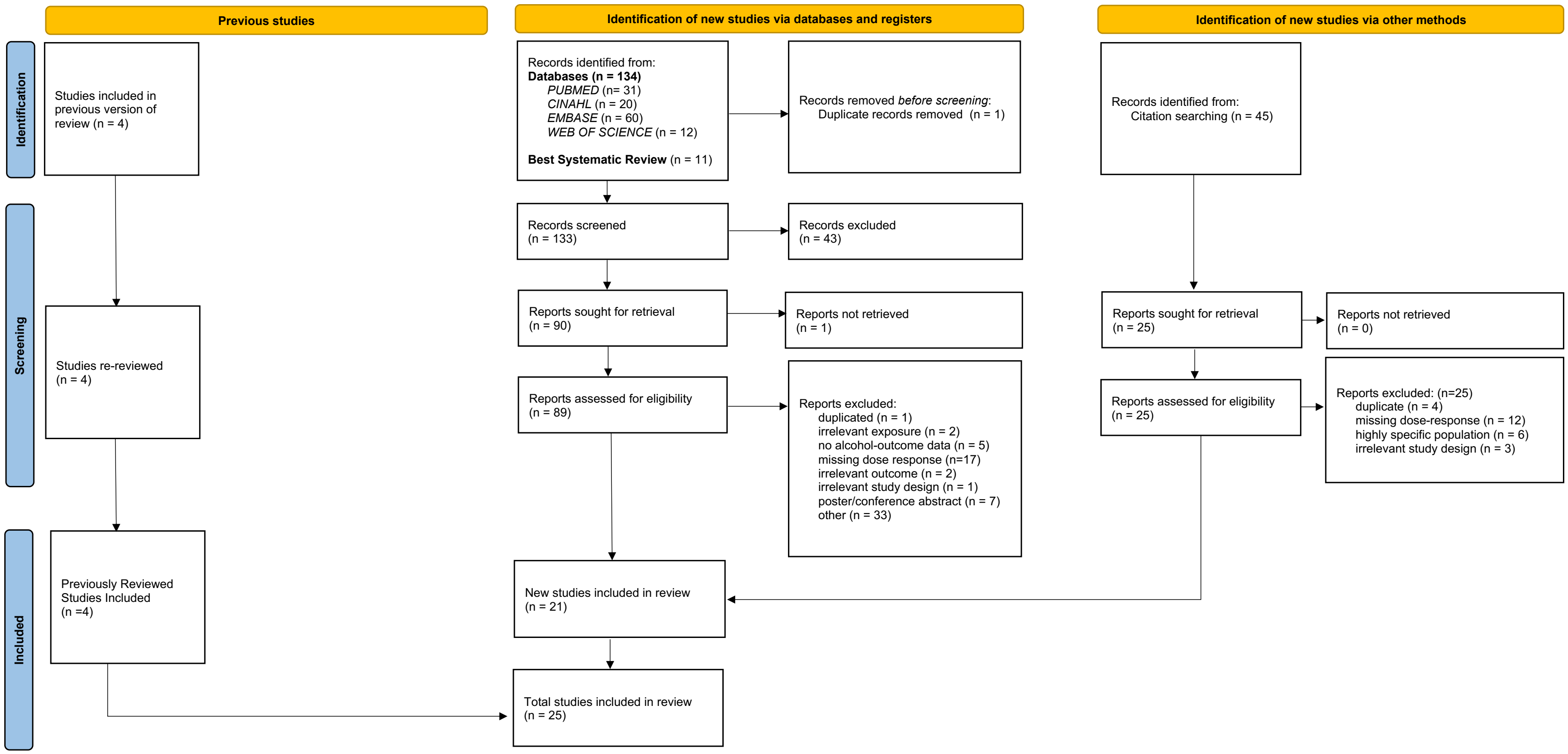

Figure S1.14. Stomach cancer

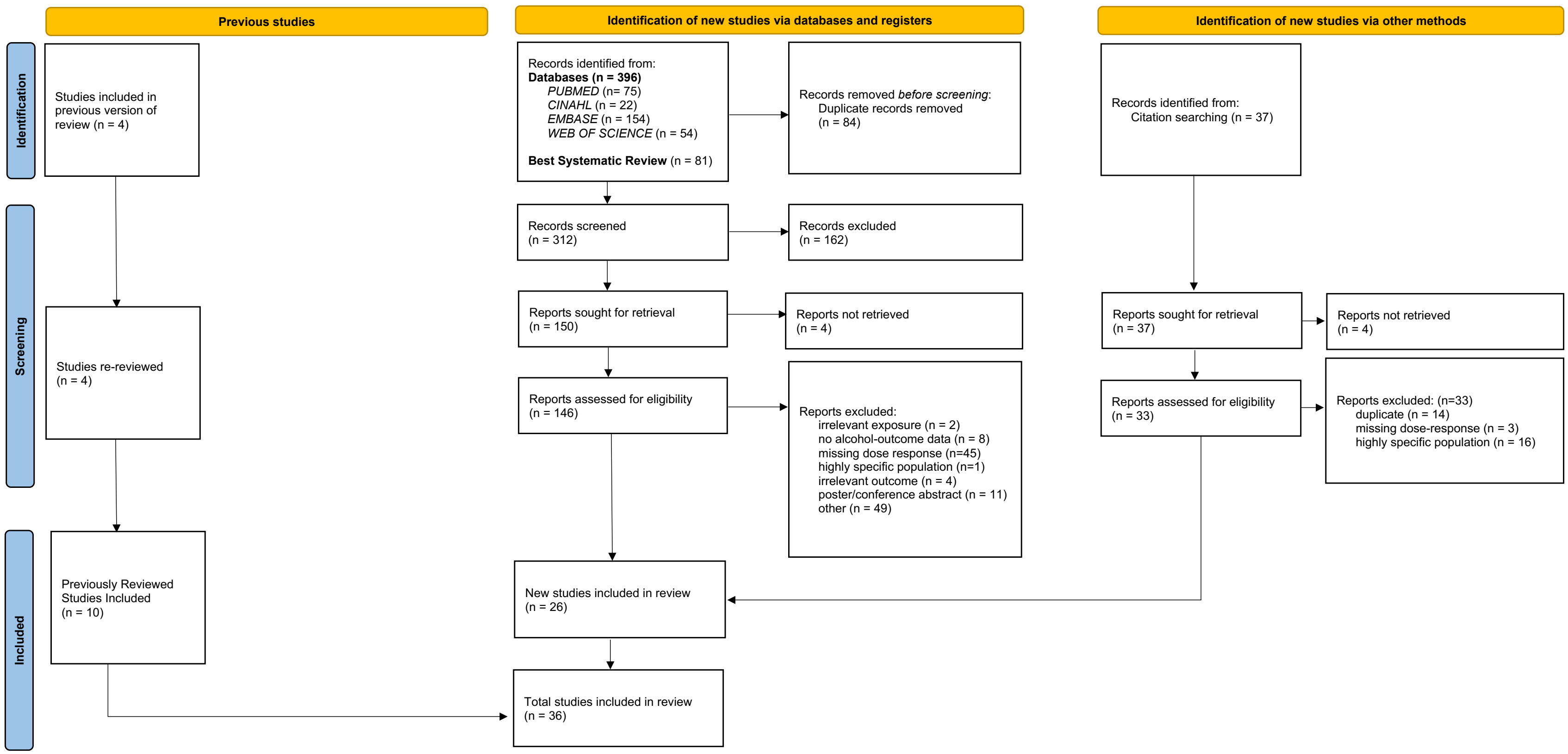

Figure S1.15. Ischemic and hemorrhagic stroke

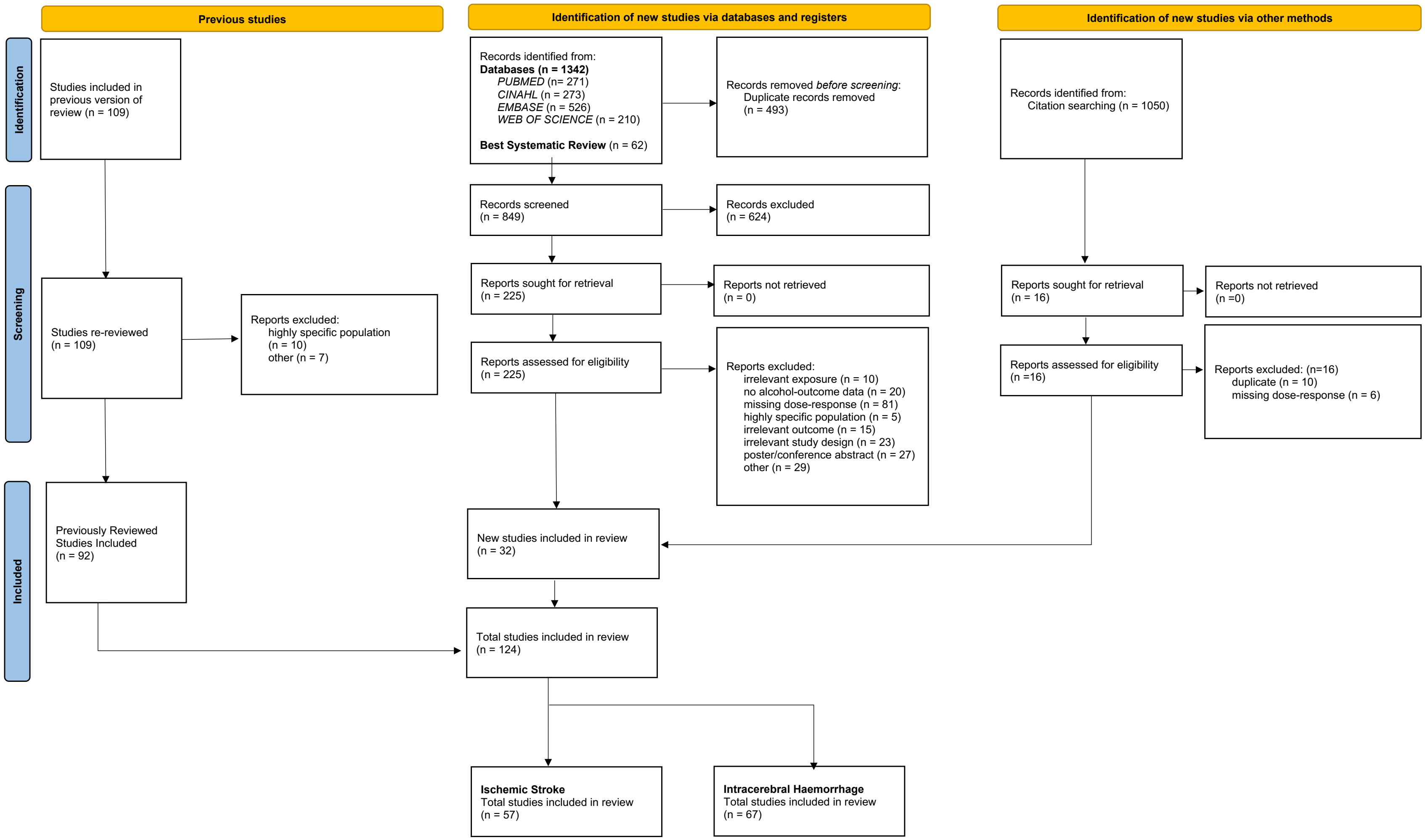

Figure S1.16. Tuberculosis

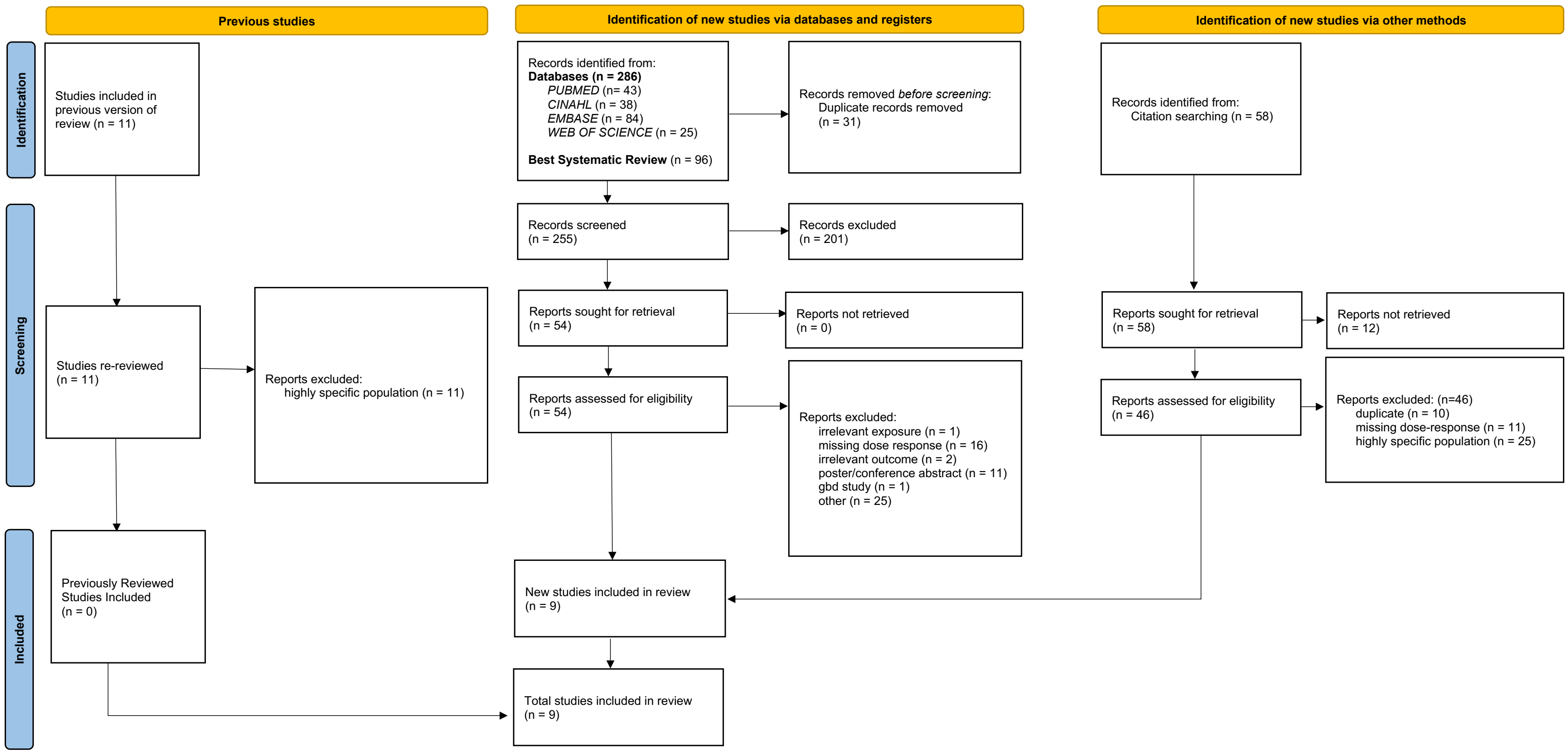

VI. Data Extraction

Data extraction was performed by one member of the research team for every included source. Effect sizes and relevant study characteristics were extracted. Data extraction was done in the DistillerSR platform. A detailed explanation for each extracted variable follows below.

A. Data Extraction Template

Table S8. Data extraction template

**Category**	Variable	Definition
**Source**	seq	
	underlying_nid	Underlying NID: Enter the underlying NID of the study (if applicable). Always talk to a data indexer if you don't know if an underlying NID is needed. They may be used for meta-analyses, certain database sources, and in some other specific cases.
	nid	Found in GHDx, created through the epi form, or created by Data Indexer
	field_citation_value	IHME Zotero format or if source has NID, citation info from GHDx
	file_path	optional; full file path of article; Only needed if source doesn't have NID, to facilitate NID creation.
**Risk-outcome pair**	risk	Risk: Select the risk factor, if not listed here, contact the causal criteria team
	risk_mapping	the relationship between study definition of risk and GBD definition of risk for a particular effect size
	outcome	Outcome: Select the outcome.
	outcome_mapping	the relationship between study definition of outcome and GBD definition of outcome for a particular effect size
**Location**	location_name	location name (from locations tab). Do a fast double-click in this field to get the drop-down menu, then start typing the location_name. For location_names with special characters, you may need to use the scroll bar.
	location_id	autopopulated from location_name
	rep_geography	Were the study partcipants representative of the geography? 1=yes, 0=no
	rep_selection_criteria	If rep_geography is 0, please specify the selection criteria of the study that is used in the analysis
	rep_prevalent_disease	Is the study aiming to evaluate the risk or mortality of people who have already developed the outcome? 1=yes 0=no (i.e. yes if for SBP-IHD paper, all partcipants have IHD at baseline and the paper is looking at mortality due to SBP, no if for SBP-IHD paper the participants have other prevalent diseases)
**Study Population**	year_start	year the study was started. If not specified, leave blank
	year_end	year the study was finished (including most recent follow up). If not specified, leave blank
	age_start	ages from 1 and above must be entered as an integer. Ages <1 can be entered as decimal values, e.g., 3 days = 3/365.
	age_end	ages from 1 and above must be entered as an integer. Ages <1 can be entered as decimal values, e.g., 3 days = 3/365.
	age_mean	Mean age
	age_sd	SD of age

	age_issue	0 = no issue flagged; 1 = issue flagged for modeler; always include explanatory notes the note_SR column
	percent_male<br><br>sex_issue	what percent of the population is male (0-1), if pop is all female then it would be 0<br>sex_issue
**Study Design**	design	Study design: Specify the design of the study
	study_name	Study Name: Enter the name of the study (e.g., Nurses' Health Study), if provided. Do not enter the title of the article.
**Exposure**	exp_assess_level	Level of exposure assessment: The exposure was assessed...
	exp_instrument	Exposure assessment instrument: Specify the name of the exposure assessment instrument. For self-reported exposures, please specify the name of the questionnaire e.g., International Physical Activity Questionnaire (IPAQ). If more than one instrument specify all
	exp_assess_period	What was the frequency of exposure assessment?
	exp_assess_num	if multiple, specify the number of times that exposure was assessed (excluding baseline)
	exp_method_1	Please specify the method of exposure assessment. If there are more than 1, please add in the next columns labeled "exp_method_2".
	exp_method_2	Please specify the method of exposure assessment. If there are more than 2, please add in the next columns labeled "exp_method_3".
	exp_method_3	Please specify the method of exposure assessment.
	exp_recall_period	This field describes the unit of exposure recall used in data collection ONLY for self-report. Select the correct option from the drop-down menu. If the unit is days, weeks, months, or years, please enter the number in exp_recall_period_value (next column). If the unit is 'lifetime', nothing needs to be entered in exp_recall_period_value. For example, if the study said the recall period was 4 weeks, enter 4 in exp_recall_period_value, and 'weeks' in the field exp_recall_period. If 'other' is selected, please describe in exp_recall_period_other
	exp_recall_period_value	If you entered days, weeks, months, or years in the field 'exp_recall_period', please enter the corresponding integer in this field. For example, if the study said the recall period was 4 weeks, enter 4 in exp_recall_period_value, and 'weeks' in the field exp_recall_period.
	exp_recall_period_other	If 'other' was selected in exp_recall_period, please describe the exposure recall period that the study specified (e.g., recall of exposure from 12 to 18 years).
	exp_type	Which form of the exposure was included in relative risk estimation analysis?
**Outcome**	outcome_def	Outcome definition: Provide a brief description of the outcome as reported in the study.

	outcome_type	Outcome type: please specify if the outcome definition included incidence of or mortality from a disease endpoint
	outcome_assess_1	Method of outcome assessment: Specify the method of assessment of the study outcome. If more than 1 are appropriate, enter additional methods in the next column labeled "outcome_assess_2"
	outcome_assess_2	Method of outcome assessment: Specify the method of assessment of the study outcome. If more than 2 are appropriate, enter additional methods in the next column labeled "outcome_assess_3"
	outcome_assess_3	Method of outcome assessment: Specify the method of assessment of the study outcome.
**Follow up**	duration_fup_measure	Type of follow up measure (i.e. mean, median, max, min)
	duration_fup_units	Units of follow up duration
	value_of_duration_fup	Enter the length of participant follow-up.
**Confounders**	cov_incidence	relative risk is reported for incidence of outcome. mark 1 for incidence. Mark 0 for mortality, incidence & mortality combined
	cov_age	study controls for age
	cov_sex	study controls for sex
	cov_bmi	study controls for BMI
	cov_edu	study controls for years of education
	cov_inc	study controls for income or socioeconomic status
	cov_activity	study controls for physical activity
	cov_smoking	study controls for smoking
	cov_menopause	study controls for menopause (breast cancer outcome only)
	cov_otherdrug	study controls for consumption of drug (not nicotine or alcohol)
	cov_diet	study controls for dietary factors
	cov_other	study controls for other factors (list)
	cov_hypertension	study controls for evidence of hypertension
	cov_diabetes	study controls for diabetes or high blood glucose
	cov_vitmain	study controls for vitamin defiiciency
	cov_otherdisease	study controls for other disease (specify)
	cov_sick_excl	study excludes sick quitters
	cov_former_separate	study treats former drinkers as separate from current trinkers
	cov_former_excl	study excludes former drinkers
	cov_sick_lag	study includes lag time for sick patients
	cov_sick_na	study does not account for sick quitters
	cov_representativeness	study population is representative of location
	cov_rep_prevalent_disease	study population has a prevalent disease (not related to outcome)
	cov_adjusted_by_outcome	original relative risk is adjusted by outcome of interest (for studies where outcome of interest is not main focus)

	cov_followup_excl	study excludes participants based on followup
	cov_APOE	study controls for APOE gene (dementia outcome only)
	cov_familyhx	study controls for family history of outcome
	cov_covid19	study controls for covid-19
	cov_alcohol_type_any	relative risk is reported for consumption of any alcohol
	cov_alcohol_type_beer	relative risk is reported for beer only
	cov_alcohol_type_wine	relative risk is reported for wine only
	cov_alcohol_type_liquor	relative risk is reported for liquor only
	cov_reverse_causation	risk of reverse causation
	cov_selection_bias	risk of selection bias
	cov_hypercholesterolemia	study controls for hypercholesterolemia
	cov_stroke_parent	study controls for parent's ischemic stroke
	cov_stroke_hem_parent	study controls for parent's hemorrhagic stroke
	cov_ihd	study controls for previous diagnosis of ischemic heart disease
	confounders_other	For other confounders that not listed, list here
**Effect Size**	page_num_effect_size	Page number (where you found effect_size) from literature, or survey question where you found effect size; Use page number(s) of article, not page # of pdf
	effect_size_measure	Effect size measure: Specify the measure of effect size
	effect_size	Effect size estimate: Provide the effect size estimate
	lower	Provide the lower limit of the confidence interval. Enter on a "per 1" basis. (If the CI is reported as a percent, you must convert to a decimal.) These 3 fields must all be filled in if any of them are filled in: lower, upper, uncertainty_type_value.
	upper	Provide the upper limit of the confidence interval. Enter on a "per 1" basis. (If the CI is reported as a percent, you must convert to a decimal.) These 3 fields must all be filled in if any of them are filled in: lower, upper, uncertainty_type_value.
	CI_uncertainty_type_value	This field is required if 'lower' & 'upper' are entered. This column represents the confidence level which is reported at (Eg. 95, 90, 99). These 3 fields must all be filled in if any of them are filled in: lower, upper, uncertainty_type_value.
	nonCI_uncertainty_value	Numerical value of the nonCI_uncertainty_type entered in that column. For example, if SD=5.3, you'd put 5.3 in this column, and choose SD from the drop down menu in nonCI_uncertainty_type.
	nonCI_uncertainty_type	Enter SE or SD if appropriate. For example, if SD=5.3, you'd put 5.3 in nonCI_uncertainty_value, and choose SD from the drop down menu in this column (nonCI_uncertainty_type).
	uncertainty_issue	Mark with a 1 if no uncertainty is reported, if some sort of uncertainty is reported, mark 0
	subgroup_analysis	1 if RR is from main analysis (all participants), 0 if sub-analysis (only males, or among a specific age group, etc.)
	subgroup_analysis_free_text	if a sub-analysis, describe it (i.e. age, sex, etc.)

	effect_size_multi_location	1 if the reported effect size is from a multi-country study and only one effect size has been reported for all locations, otherwise 0
	effect_size_multi_location_specify	which geography level is the RR for
	pooled_cohort	1 if the reported effect size is from a pooled analysis and only pooled effect size has been reported, otherwise 0
	dose_response	Does the study support a dose-response relationship between the exposure and the outcome? (1= yes, 0=no)
	dose_response_detail	If "1" was specified in the dose_response field, please specify in this field the type of evidence supporting the dose-response relationship. For example, "statistically significant p value for linear trend".
**Cohorts**	cohort_person_years_exp	Please specify the person years of follow up in the exposed group
	cohort_person_years_unexp	Please specify the person years of follow up in the unexposed group
	cohort_person_years_total	Enter the total person-years of follow-up if person-years of follow up in exposed and unexposed not reported
	cohort_number_events_exp	Please specify the number of events in the exposed group
	cohort_number_events_unexp	Please specify the number of events in the unexposed group
	cohort_number_events_total	Enter the total number of events/cases if number of events in exposed and unexpoxed not reported
	cohort_sample_size_exp	Please specify the number of people in the exposed group if person-years of follow up in exposed not reported
	cohort_sample_size_unexp	Please specify the number of people in the unexposed group if person-years of follow up in unexposed not reported
	cohort_sample_size_total	Please specify the number of people included in the analysis if total person-years of follow up in not reported
	cohort_dropout_rate	Dropout rate: Specify the dropout rate (%) at the end of the study. Enter on a "per 1" basis. For example: 23% is entered as .23.
	cohort_dropout_assess	Specify how dropout rate was defined in the study.
	cohort_exposed_def	exposed group definition: Provide a brief description of the exposed group (i.e., the comparison group) as used in estimation of the relative risk (e.g., never smokers)
	cohort_exp_unit_rr	Exposure unit (for continuous risks): Specify the unit of exposure (e.g., grams/day).
	cohort_exp_level_rr	Exposure level in the exposed group (for continuous risks): Specify the mean/median level of exposure in the exposed group.
	cohort_unexp_def	unexposed group definition: Provide a brief description of the unexposed group (i.e., the comparison group) as used in estimation of the relative risk (e.g., never smokers)
	cohort_unexp_unit_rr	Exposure unit (for continuous risks): Specify the unit of exposure (e.g., grams/day) for the unexposed group

	cohort_unexp_level_rr	Exposure level in the unexposed group (for continuous risks): Specify the mean/median level of exposure in the unexposed group.
	cohort_exp_level_dr	Exposure level in for dose-repose RRs (for continuous risks): If the study reports dose-repose RR, please specify the level of exposure for the reported RR
**Case-control**	cc_community	Were the controls selected from the community? 1 = yes, 0=no
	cc_cases	Number of cases
	cc_control	Number of controls
	cc_exposed_def	Exposed group definition: Provide a brief description of the exposed group for which the the relative risk is reported (e.g., current smokers)
	cc_exp_unit_rr	Exposure unit (for continuous risks): Specify the unit of exposure (e.g., grams/day).
	cc_exp_level_rr	Exposure level in the exposed group (for continuous risks): Specify the mean/median level of exposure in the exposed group.
	cc_unexposed_def	Unexposed group definition: Provide a brief description of the unexposed group (i.e., the comparison group) as used in estimation of the relative risk (e.g., never smokers)
	cc_unexp_unit_rr	
	cc_unexp_level_rr	Exposure level in the unexposed group (for continuous risks): Specify the mean/median level of exposure in the unexposed group.
	cc_exp_level_dr	Exposure level in for dose-repose RRs (for continuous risks): If the study reports dose-repose RR, please specify the level of exposure for the reported RR
**Other**	note_modeler	for modelers only, audience is modeler, not for correspondence
	note_sr	notes related to extraction, including assumptions, data adjustment, problems with source, any other notes that may be relevant, etc.
	extractor	uwnet id of person who extracted the data
**Custom**	custom_exp_meas_num	If the exposure level was assessed multiple times at a given time point (e.g., systolic blood pressure), specify the number of measurements at each time point.
	custom_exp_biomarker	If the exposure level was assessed via a biomarker, specify the full name of the biomarker.
	custom_age_demographer	A binary flag to identify if ages are presented in demographer notation or not in the source. This value is currently not used to adjust any age_start or age_end values, but in the future, that is the intention; 0 = article does not use demographer notation (4 = 4.00 not 4.99); 1 = article uses demographer notation (4=4.99 not 4.00)
	ref_risk_lower	lower value of the reference group's exposure

ref_risk_upper	upper value of the referenfe group's exposure
alt_risk_lower	lower value of the alternate group's exposure
alt_risk_upper	upper value of the alternate group's exposure
quant_convert	conversion value for quantity measurement
freq_convert	conversion value for frequency measurement

VII. Data Processing

After data extraction, all data was reviewed to ensure that no extraction errors had occurred. After confirming that the extracted data was correct, data was processed in the following ways:

1. Any exposure data that was not already in grams of pure ethanol per day was converted
   a. For RRs that were estimated for specific alcohol, we used the following estimates to convert from specific alcohol to total ethanol;

Alcohol type	Conversion factor
Wine	0.12 (12% ABV, a strong red wine)
Beer	0.05 (5% ABV, standard beer)
Liquor	0.40 (40% ABV or 80-proof)

   b. Any exposure that was in volume was converted using the density of alcohol, 0.789 g/mL;
   c. One drink was considered to be a standard drink of alcohol, equivalent to 10g when otherwise unspecified. This corresponds to 12oz of beer/5oz of wine/or 1.5oz of liquor.
   d. Amount per month was converted into per day by assuming one month equal to 30 days.

2. For each outcome, data was analyzed using MRBRT to generate risk curves. No parameters were enforced nor were bias covariates selected a priori.

3. Data distributions were examined to identify any outliers

VIII. Supplementary Methods of the Burden of Proof Approach

Overview

We applied the Burden of Proof (BoP) meta-analytic framework to assess the strength of evidence for associations between levels of alcohol consumption and health outcomes. The BoP approach is designed to provide a conservative and transparent synthesis of epidemiological evidence by explicitly accounting for nonlinear dose–response relationships, study-level bias, between-study heterogeneity, small study effects, and potential outliers. The analytical workflow followed six sequential steps, consistent with the description in the main manuscript.

Step 1. Comprehensive systematic review for each risk–outcome pair

For each alcohol–outcome pair, we conducted a comprehensive systematic review following PRISMA guidelines to identify eligible epidemiologic studies. We included observational studies (cohort and case–control studies) that reported relative risks (RRs), hazard ratios, or odds ratios comparing different levels of alcohol consumption.

From each eligible study, we extracted effect estimates and associated uncertainty, alcohol exposure categories includnig the chosen reference group(s), outcome definitions, study design characteristics, and covariates adjusted for in the original analyses. When multiple estimates were reported within a study, these were retained and handled using appropriate within-study correlation structures during model fitting.

Step 2. Determination of the exposure–outcome dose–response relationship

Alcohol exposure was harmonized across studies and expressed as grams of pure alcohol consumed per day (g/day). When alcohol intake was reported categorically, we recorded the lower and upper bounds of each exposure category for both the exposed and reference groups.

We estimated dose–response relationships using the MR-BRT (Meta-Regression–Bayesian, Regularized, Trimmed) method, which allows flexible estimation of dose–response curves. Rather than assigning midpoints to exposure categories, MR-BRT integrates over reported exposure ranges, enabling consistent estimation when categories overlap across studies.

The dose–response risk function was modeled using spline-based regression, allowing for nonlinear relationships between alcohol consumption and health outcomes. Where biologically or epidemiologically justified, shape constraints were applied (e.g., non-decreasing risk with increasing alcohol consumption for cancer outcomes).

Step 3. Characterization and adjustment for bias covariates across studies

To account for systematic differences in effect estimates due to study design and analysis, we characterized potential sources of bias using study-level covariates. These included indicators related to exposure measurement quality, outcome ascertainment, representativeness of the study population, adjustment for key confounders, and potential for reverse causation.

Candidate bias covariates were evaluated within the MR-BRT meta-regression framework. Covariate selection was performed using a regularized (lasso-based) procedure to reduce overfitting. Bias covariates retained by the selection procedure were included as fixed effects, and final dose–response predictions were generated under preferred (lowest-bias) covariate settings.

Step 4. Incorporation of unexplained between-study heterogeneity into uncertainty estimation

Between-study heterogeneity not explained by bias covariates was modeled using random effects that scale the estimated dose–response relationship at the study level. Uncertainty in the heterogeneity parameter was explicitly propagated into uncertainty intervals for the estimated risk curves.

For outcomes with sparse data, we applied Fisher information–based corrections to stabilize heterogeneity estimation and avoid underestimation of uncertainty when the number of contributing studies was small.

Step 5. Testing for and reporting potential publication bias

We assessed potential publication or reporting bias using a modified Egger's regression approach applied to the residuals of the fitted meta-regression models. This analysis tested for associations between residual magnitude and standard error after accounting for the dose–response relationship and bias covariates.

Risk–outcome pairs with statistically significant evidence of publication bias were flagged. Consistent with prior BoP applications, we did not apply formal corrections for publication bias; instead, evidence of potential bias was reported as a limitation.

Step 6. Calculation of the Burden of Proof Risk Function (BPRF)

The Burden of Proof Risk Function (BPRF) was derived as a conservative estimate of the alcohol–outcome relationship that incorporates uncertainty from all previous steps, including bias adjustment and between-study heterogeneity.

For harmful associations, the BPRF was defined as the 5th percentile of the estimated dose–response distribution closest to the null (RR = 1). For protective associations, the BPRF was defined as the 95th percentile closest to the null. The BPRF represents the smallest harmful or protective association at each level of alcohol consumption that remains consistent with the available evidence.

To summarize the strength of evidence for each alcohol–outcome pair, we averaged the BPRF over the exposure range supported by the literature (defined as the 15th to 85th percentile of observed exposure) to compute a Risk–Outcome Score (ROS). ROS values were then mapped to a five-category star rating system, reflecting the strength of evidence under a conservative interpretation.

Implementation notes

All analyses were conducted using a standardized BoP pipeline in Python, with consistent exposure harmonization, spline specifications, trimming procedures, and bias-covariate definitions applied across outcomes to ensure comparability.

IX. Relative Risk Sources

A. Study Characteristics

Table S9. Summary of study characteristics

Author	Year	Health outcome	Location	Study design	Sex	Age Start	Age End	Exposure Assessment	Outcome Assessment Method	Outcome type	Sample Size	Reference	Exposed group	Cases	Controls	Control Pool	Alcohol Type
Filippini	2019	Alzheimer's disease and other dementias	Italy	Case-control	Both	45.0	65.0	Self-report (human/environment)	Physician diagnosis	Morbidity	54	Light drinkers	Moderate drinkers	N/A	N/A	Family or Friends	Any alcoholic beverages
Handing	2010	Alzheimer's disease and other dementias	Sweden	Prospective Cohort	Both	36.5	71.9	Self-report (human/environment)	Administrative medical records or disease registry	Morbidity	12326	Light drinkers	Light drinkers	N/A	N/A	N/A	Any alcoholic beverages
Heffernan	2015	Alzheimer's disease and other dementias	Australia	Prospective Cohort	Both	70.0	90.0	Self-report (human/environment)	Physician diagnosis	Morbidity	821	Non-drinkers	Heavy drinkers	N/A	N/A	N/A	Any alcoholic beverages
Järvenpää	2005	Alzheimer's disease and other dementias	Finland	Prospective Cohort	Both	56.7	92.5	Self-report (human/environment)	Physician diagnosis	Morbidity	554	Non-drinkers	Light drinkers	N/A	N/A	N/A	Any alcoholic beverages
Järvenpää	2005	Alzheimer's disease and other dementias	Finland	Prospective Cohort	Both	56.7	92.5	Self-report (human/environment)	Physician diagnosis	Morbidity	554	Non-drinkers	Moderate drinkers	N/A	N/A	N/A	Any alcoholic beverages
Kawakami	2020	Alzheimer's disease and other dementias	Japan	Prospective Cohort	Both	31.1	86.9	Self-report (human/environment)	Physician diagnosis	Morbidity	N/A	Non-drinkers	Moderate drinkers	N/A	N/A	N/A	Any alcoholic beverages
Kawakami	2020	Alzheimer's disease and other dementias	Japan	Prospective Cohort	Male	31.1	86.9	Self-report (human/environment)	Physician diagnosis	Morbidity	N/A	Non-drinkers	Heavy drinkers	N/A	N/A	N/A	Any alcoholic beverages
Kawakami	2020	Alzheimer's disease and other dementias	Japan	Prospective Cohort	Female	31.1	86.9	Self-report (human/environment)	Physician diagnosis	Morbidity	N/A	Non-drinkers	Heavy drinkers	N/A	N/A	N/A	Any alcoholic beverages

Table S9. Summary of study characteristics

Author	Year	Health outcome	Location	Study design	Sex	Age Start	Age End	Exposure Assessment	Outcome Assessment Method	Outcome type	Sample Size	Reference	Exposed group	Cases	Controls	Control Pool	Alcohol Type
Kawakami	2020	Alzheimer's disease and other dementias	Japan	Prospective Cohort	Both	31.1	86.9	Self-report (human/environment)	Physician diagnosis	Morbidity	N/A	Non-drinkers	Heavy drinkers	N/A	N/A	N/A	Any alcoholic beverages
Larrieu	2004	Alzheimer's disease and other dementias	France	Prospective Cohort	Both	65.0	95.0	Self-report (human/environment)	Physician diagnosis	Morbidity	2950	Light drinkers	Heavy drinkers	N/A	N/A	N/A	Wine
Mewton	2022	Alzheimer's disease and other dementias	Global	Prospective Cohort	Both	15.0	99.0	N/A	N/A	Morbidity	N/A	Non-drinkers	Light drinkers	N/A	N/A	N/A	Any alcoholic beverages
Ruitenberg	1999	Alzheimer's disease and other dementias	Netherlands	Prospective Cohort	Both	55.0	99.0	Self-report (human/environment)	Administrative medical records or disease registry	Morbidity	5395	Non-drinkers	Heavy drinkers	N/A	N/A	N/A	Any alcoholic beverages
Sabia	2017	Alzheimer's disease and other dementias	United Kingdom	Prospective Cohort	Both	58.2	93.0	Self-report (human/environment)	Administrative medical records or disease registry	Morbidity	9087	Light drinkers	Light drinkers	N/A	N/A	N/A	Any alcoholic beverages
Sabia	2017	Alzheimer's disease and other dementias	United Kingdom	Prospective Cohort	Both	58.2	93.0	Self-report (human/environment)	Administrative medical records or disease registry	Morbidity	9087	Light drinkers	Moderate drinkers	N/A	N/A	N/A	Any alcoholic beverages
Weyerer	2010	Alzheimer's disease and other dementias	Germany	Prospective Cohort	Both	75.0	99.0	Self-report (human/environment)	Physician diagnosis	Morbidity	3180	Non-drinkers	Moderate drinkers	N/A	N/A	N/A	Any alcoholic beverages
Jeon	2023	Alzheimer's disease and other dementias	Republic of Korea	Prospective Cohort	Males	40	85	Self-report (human/environment)	Administrative medical records or disease registry	Morbidity	1218764	non drinker	Current drinker, Moderate	N/A	N/A	N/A	Any Alcoholic Beverages
Jeon	2023	Alzheimer's disease and	Republic of Korea	Prospective Cohort	Females	40	85	Self-report (human/environment)	Administrative medical records or	Morbidity	1574295	non drinker	Current drinker, Moderate	N/A	N/A	N/A	Any Alcoholic Beverages

Table S9. Summary of study characteristics

Author	Year	Health outcome	Location	Study design	Sex	Age Start	Age End	Exposure Assessment	Outcome Assessment Method	Outcome type	Sample Size	Reference	Exposed group	Cases	Controls	Control Pool	Alcohol Type
		other dementias							disease registry								
Feter	2022	Alzheimer's disease and other dementias	United Kingdom	Prospective Cohort	Both	42.6	82.2	Self-report (human/environment)	Administrative medical records or disease registry	Morbidity	6021	Current drinker, Moderate	Current drinker, Heavy	N/A	N/A	N/A	Any Alcoholic Beverages
Koch	2019	Alzheimer's disease and other dementias	United States of America	Prospective Cohort	Both	72	99	Self-report (human/environment)	Physician Diagnosis	Morbidity	2548	Current drinker, Light	Current drinker, Light	N/A	N/A	N/A	Any Alcoholic Beverages
Shimizu	2023	Alzheimer's disease and other dementias	Japan	Prospective Cohort	Both	40	69	Self-report (human/environment)	Administrative medical records or disease registry	Morbidity	42870	Current drinker, Light	Non Drinker	N/A	N/A	N/A	Any Alcoholic Beverages
Jung	2024	Alzheimer's disease and other dementias	Republic of Korea	Prospective Cohort	Both	40	59	Self-report (human/environment)	Administrative medical records or disease registry	Morbidity	4640148	Non Drinker	Current drinker, Light	N/A	N/A	N/A	Any Alcoholic Beverages
Topiwala	2025	Alzheimer's disease and other dementias	United Kingdom	Prospective Cohort	Both	41.9	86.3	Self-report (human/environment)	Administrative medical records or disease registry	Morbidity	204366	Current drinker, Light	Current drinker, Moderate	N/A	N/A	N/A	Any Alcoholic Beverages
Larsson	2018	Alzheimer's disease and other dementias	Sweden	Prospective Cohort	Females	62.6	81.4	Self-report (human/environment)	Administrative medical records or disease registry	Morbidity	13401	Lifetime Abstainer	Current drinker, Light	N/A	N/A	N/A	Any Alcoholic Beverages
Larsson	2018	Alzheimer's disease and other dementias	Sweden	Prospective Cohort	Males	62.8	79.6	Self-report (human/environment)	Administrative medical records or disease registry	Morbidity	15374	Lifetime Abstainer	Current drinker, Light	N/A	N/A	N/A	Any Alcoholic Beverages
Ariansen	2019	Atrial fibrillation and flutter	Norway	Prospective Cohort	Both	15.0	73.5	Self-report (human/environment)	Administrative medical records or	Morbidity	234392	Light drinkers	Light drinkers	N/A	N/A	N/A	Any alcoholic beverages

Table S9. Summary of study characteristics

Author	Year	Health outcome	Location	Study design	Sex	Age Start	Age End	Exposure Assessment	Outcome Assessment Method	Outcome type	Sample Size	Reference	Exposed group	Cases	Controls	Control Pool	Alcohol Type
									disease registry								
Bazal	2017	Atrial fibrillation and flutter	Spain	Randomized Control Trial	Both	47.9	84.7	Self-report (human/environment)	Administrative medical records or disease registry	Morbidity	N/A	Non-drinkers	Light drinkers	N/A	N/A	N/A	Any alcoholic beverages
Bazal	2017	Atrial fibrillation and flutter	Spain	Randomized Control Trial	Both	49.2	85.4	Self-report (human/environment)	Administrative medical records or disease registry	Morbidity	N/A	Non-drinkers	Light drinkers	N/A	N/A	N/A	Any alcoholic beverages
Bazal	2017	Atrial fibrillation and flutter	Spain	Randomized Control Trial	Both	46.9	82.8	Self-report (human/environment)	Administrative medical records or disease registry	Morbidity	N/A	Non-drinkers	Light drinkers	N/A	N/A	N/A	Any alcoholic beverages
Bazal	2017	Atrial fibrillation and flutter	Spain	Randomized Control Trial	Both	47.9	84.7	Self-report (human/environment)	Administrative medical records or disease registry	Morbidity	N/A	Non-drinkers	Moderate drinkers	N/A	N/A	N/A	Any alcoholic beverages
Bazal	2017	Atrial fibrillation and flutter	Spain	Randomized Control Trial	Both	49.2	85.4	Self-report (human/environment)	Administrative medical records or disease registry	Morbidity	N/A	Non-drinkers	Moderate drinkers	N/A	N/A	N/A	Any alcoholic beverages
Bazal	2017	Atrial fibrillation and flutter	Spain	Randomized Control Trial	Both	46.9	82.8	Self-report (human/environment)	Administrative medical records or disease registry	Morbidity	N/A	Non-drinkers	Moderate drinkers	N/A	N/A	N/A	Any alcoholic beverages
Biddinger	2016	Atrial fibrillation and flutter	United Kingdom	Prospective Cohort	Both	33.2	80.8	Self-report (human/environment)	Administrative medical records or disease registry	Morbidity	N/A	Non-drinkers	Light drinkers	N/A	N/A	N/A	Any alcoholic beverages

Table S9. Summary of study characteristics

Author	Year	Health outcome	Location	Study design	Sex	Age Start	Age End	Exposure Assessment	Outcome Assessment Method	Outcome type	Sample Size	Reference	Exposed group	Cases	Controls	Control Pool	Alcohol Type
Cha	2015	Atrial fibrillation and flutter	Republic of Korea	Prospective Cohort	Both	15.9	78.3	Self-report (human/environment)	Administrative medical records or disease registry	Morbidity	19634	Light drinkers	Light drinkers	N/A	N/A	N/A	Any alcoholic beverages
Csengeri	2010	Atrial fibrillation and flutter	Denmark	Prospective Cohort	Both	15.0	99.0	Self-report (human/environment)	Administrative medical records or disease registry	Morbidity	N/A	Non-drinkers	Light drinkers	N/A	N/A	N/A	Any alcoholic beverages
Csengeri	2010	Atrial fibrillation and flutter	Denmark	Prospective Cohort	Both	15.0	99.0	Self-report (human/environment)	Administrative medical records or disease registry	Morbidity	N/A	Non-drinkers	Moderate drinkers	N/A	N/A	N/A	Any alcoholic beverages
Di Benedetto	2017	Atrial fibrillation and flutter	United Kingdom	Prospective Cohort	Female	31.2	86.4	Self-report (human/environment)	Administrative medical records or disease registry	Morbidity	N/A	Light drinkers	Moderate drinkers	N/A	N/A	N/A	Any alcoholic beverages
Di Benedetto	2017	Atrial fibrillation and flutter	United Kingdom	Prospective Cohort	Female	30.4	86.2	Self-report (human/environment)	Administrative medical records or disease registry	Morbidity	N/A	Light drinkers	Moderate drinkers	N/A	N/A	N/A	Any alcoholic beverages
Di Castelnuovo	2015	Atrial fibrillation and flutter	Italy	Prospective Cohort	Both	20.0	86.0	Self-report (human/environment)	Administrative medical records or disease registry	Morbidity	N/A	Non-drinkers	Light drinkers	N/A	N/A	N/A	Any alcoholic beverages
Di Castelnuovo	2015	Atrial fibrillation and flutter	Italy	Prospective Cohort	Both	18.0	90.0	Self-report (human/environment)	Administrative medical records or disease registry	Morbidity	N/A	Non-drinkers	Light drinkers	N/A	N/A	N/A	Any alcoholic beverages
Di Castelnuovo	2015	Atrial fibrillation and flutter	Italy	Prospective Cohort	Both	21.0	93.0	Self-report (human/environment)	Administrative medical records or disease registry	Morbidity	N/A	Non-drinkers	Light drinkers	N/A	N/A	N/A	Any alcoholic beverages

Table S9. Summary of study characteristics

Author	Year	Health outcome	Location	Study design	Sex	Age Start	Age End	Exposure Assessment	Outcome Assessment Method	Outcome type	Sample Size	Reference	Exposed group	Cases	Controls	Control Pool	Alcohol Type
Di Castelnuovo	2015	Atrial fibrillation and flutter	Italy	Prospective Cohort	Both	24.0	90.0	Self-report (human/environment)	Administrative medical records or disease registry	Morbidity	N/A	Non-drinkers	Light drinkers	N/A	N/A	N/A	Any alcoholic beverages
Di Castelnuovo	2015	Atrial fibrillation and flutter	Italy	Prospective Cohort	Both	20.0	86.0	Self-report (human/environment)	Administrative medical records or disease registry	Morbidity	N/A	Non-drinkers	Heavy drinkers	N/A	N/A	N/A	Any alcoholic beverages
Di Castelnuovo	2015	Atrial fibrillation and flutter	Italy	Prospective Cohort	Both	18.0	90.0	Self-report (human/environment)	Administrative medical records or disease registry	Morbidity	N/A	Non-drinkers	Heavy drinkers	N/A	N/A	N/A	Any alcoholic beverages
Di Castelnuovo	2015	Atrial fibrillation and flutter	Italy	Prospective Cohort	Both	21.0	93.0	Self-report (human/environment)	Administrative medical records or disease registry	Morbidity	N/A	Non-drinkers	Heavy drinkers	N/A	N/A	N/A	Any alcoholic beverages
Di Castelnuovo	2015	Atrial fibrillation and flutter	Italy	Prospective Cohort	Both	24.0	90.0	Self-report (human/environment)	Administrative medical records or disease registry	Morbidity	N/A	Non-drinkers	Heavy drinkers	N/A	N/A	N/A	Any alcoholic beverages
Dixit	2013	Atrial fibrillation and flutter	United States of America	Prospective Cohort	Both	36.4	71.2	Self-report (human/environment)	Physician diagnosis	Morbidity	15222	Non-drinkers	Light drinkers	N/A	N/A	N/A	Any alcoholic beverages
Djousse	1998	Atrial fibrillation and flutter	United States of America	Case-control	Both	15.0	99.0	N/A	N/A	Morbidity	N/A	Non-drinkers	Light drinkers	325	1430	N/A	Any alcoholic beverages
Djousse	1998	Atrial fibrillation and flutter	United States of America	Case-control	Both	15.0	99.0	N/A	N/A	Morbidity	N/A	Non-drinkers	Heavy drinkers	318	1257	N/A	Any alcoholic beverages
Djousse	1998	Atrial fibrillation and flutter	United States of America	Case-control	Both	15.0	99.0	N/A	N/A	Morbidity	N/A	Non-drinkers	Light drinkers	656	2983	N/A	Any alcoholic beverages

Table S9. Summary of study characteristics

Author	Year	Health outcome	Location	Study design	Sex	Age Start	Age End	Exposure Assessment	Outcome Assessment Method	Outcome type	Sample Size	Reference	Exposed group	Cases	Controls	Control Pool	Alcohol Type
Frederiksen	2017	Atrial fibrillation and flutter	Denmark	Prospective Cohort	Both	15.0	99.0	Self-report (human/environment)	Administrative medical records or disease registry	Morbidity	43758	Light drinkers	Light drinkers	N/A	N/A	N/A	Any alcoholic beverages
Frederiksen	2017	Atrial fibrillation and flutter	Denmark	Prospective Cohort	Both	15.0	99.0	Self-report (human/environment)	Administrative medical records or disease registry	Morbidity	43758	Light drinkers	Moderate drinkers	N/A	N/A	N/A	Any alcoholic beverages
Frederiksen	2017	Atrial fibrillation and flutter	Denmark	Prospective Cohort	Both	15.0	99.0	Self-report (human/environment)	Administrative medical records or disease registry	Morbidity	43758	Moderate drinkers	Light drinkers	N/A	N/A	N/A	Any alcoholic beverages
Frederiksen	2017	Atrial fibrillation and flutter	Denmark	Prospective Cohort	Both	15.0	99.0	Self-report (human/environment)	Administrative medical records or disease registry	Morbidity	43758	Moderate drinkers	Moderate drinkers	N/A	N/A	N/A	Any alcoholic beverages
Frederiksen	2017	Atrial fibrillation and flutter	Denmark	Prospective Cohort	Both	15.0	99.0	Self-report (human/environment)	Administrative medical records or disease registry	Morbidity	43758	Heavy drinkers	Moderate drinkers	N/A	N/A	N/A	Any alcoholic beverages
Frost	1997	Atrial fibrillation and flutter	Denmark	Prospective Cohort	Both	15.0	99.0	N/A	N/A	Morbidity	N/A	Light drinkers	Moderate drinkers	71	N/A	N/A	Any alcoholic beverages
Frost	1997	Atrial fibrillation and flutter	Denmark	Prospective Cohort	Both	15.0	99.0	N/A	N/A	Morbidity	N/A	Light drinkers	Heavy drinkers	150	N/A	N/A	Any alcoholic beverages
Frost	1997	Atrial fibrillation and flutter	Denmark	Prospective Cohort	Both	15.0	99.0	N/A	N/A	Morbidity	N/A	Light drinkers	Light drinkers	124	N/A	N/A	Any alcoholic beverages
Frost	1997	Atrial fibrillation and flutter	Denmark	Prospective Cohort	Both	15.0	99.0	N/A	N/A	Morbidity	N/A	Light drinkers	Light drinkers	75	N/A	N/A	Any alcoholic beverages

Table S9. Summary of study characteristics

Author	Year	Health outcome	Location	Study design	Sex	Age Start	Age End	Exposure Assessment	Outcome Assessment Method	Outcome type	Sample Size	Reference	Exposed group	Cases	Controls	Control Pool	Alcohol Type
Frost	1997	Atrial fibrillation and flutter	Denmark	Prospective Cohort	Both	15.0	99.0	N/A	N/A	Morbidity	N/A	Light drinkers	Light drinkers	71	N/A	N/A	Any alcoholic beverages
Gorecka	2014	Atrial fibrillation and flutter	Ireland	Prospective Cohort	Both	65.0	99.0	Self-report (human/environment)	Physician diagnosis	Morbidity	N/A	Non-drinkers	Light drinkers	N/A	N/A	N/A	Any alcoholic beverages
Gorecka	2014	Atrial fibrillation and flutter	Ireland	Prospective Cohort	Both	65.0	99.0	Self-report (human/environment)	Physician diagnosis	Morbidity	N/A	Non-drinkers	Moderate drinkers	N/A	N/A	N/A	Any alcoholic beverages
Gémes,	2015	Atrial fibrillation and flutter	Norway	Prospective Cohort	Both	15.0	99.0	Self-report (human/environment)	Administrative medical records or disease registry	Morbidity	47002	Non-drinkers	Light drinkers	N/A	N/A	N/A	Any alcoholic beverages
Gémes,	2015	Atrial fibrillation and flutter	Norway	Prospective Cohort	Both	15.0	95.0	Self-report (human/environment)	Administrative medical records or disease registry	Morbidity	47002	Non-drinkers	Light drinkers	N/A	N/A	N/A	Any alcoholic beverages
Gémes,	2015	Atrial fibrillation and flutter	Norway	Prospective Cohort	Both	15.0	91.5	Self-report (human/environment)	Administrative medical records or disease registry	Morbidity	47002	Non-drinkers	Light drinkers	N/A	N/A	N/A	Any alcoholic beverages
Gémes,	2015	Atrial fibrillation and flutter	Norway	Prospective Cohort	Both	15.0	94.1	Self-report (human/environment)	Administrative medical records or disease registry	Morbidity	47002	Non-drinkers	Light drinkers	N/A	N/A	N/A	Any alcoholic beverages
Halford	2019	Atrial fibrillation and flutter	United Kingdom	Prospective Cohort	Both	40.0	69.0	Self-report (human/environment)	Administrative medical records or disease registry	Morbidity	376776	Non-drinkers	Heavy drinkers	N/A	N/A	N/A	Any alcoholic beverages
Hamada	2015	Atrial fibrillation and flutter	Japan	Retrospective Cohort	Both	30.0	81.0	Self-report (human/environment)	Physician diagnosis	Morbidity	N/A	Non-drinkers	N/A	N/A	N/A	N/A	Any alcoholic beverages

Table S9. Summary of study characteristics

Author	Year	Health outcome	Location	Study design	Sex	Age Start	Age End	Exposure Assessment	Outcome Assessment Method	Outcome type	Sample Size	Reference	Exposed group	Cases	Controls	Control Pool	Alcohol Type
Hamada	2015	Atrial fibrillation and flutter	Japan	Retrospective Cohort	Female	40.0	79.0	Self-report (human/environment)	Physician diagnosis	Morbidity	65984	Light drinkers	Light drinkers	N/A	N/A	N/A	Any alcoholic beverages
Han	2012	Atrial fibrillation and flutter	Republic of Korea	Retrospective Cohort	Both	17.3	41.8	Self-report (human/environment)	Administrative medical records or disease registry	Morbidity	N/A	Non-drinkers	Light drinkers	N/A	N/A	N/A	Any alcoholic beverages
Han	2012	Atrial fibrillation and flutter	Republic of Korea	Retrospective Cohort	Both	17.3	41.8	Self-report (human/environment)	Administrative medical records or disease registry	Morbidity	N/A	Non-drinkers	Moderate drinkers	N/A	N/A	N/A	Any alcoholic beverages
Johansson	2014	Atrial fibrillation and flutter	Sweden	Prospective Cohort	Female	18.5	74.3	Self-report (human/environment)	Administrative medical records or disease registry	Morbidity	109230	Light drinkers	Light drinkers	N/A	N/A	N/A	Any alcoholic beverages
Johansson	2014	Atrial fibrillation and flutter	Sweden	Prospective Cohort	Female	18.7	73.9	Self-report (human/environment)	Administrative medical records or disease registry	Morbidity	109230	Light drinkers	Light drinkers	N/A	N/A	N/A	Any alcoholic beverages
Kim	2017	Atrial fibrillation and flutter	Republic of Korea	Retrospective Cohort	Both	15.0	93.5	Self-report (human/environment)	Administrative medical records or disease registry	Morbidity	N/A	Light drinkers	Light drinkers	N/A	N/A	N/A	Any alcoholic beverages
Kim	2017	Atrial fibrillation and flutter	Republic of Korea	Retrospective Cohort	Both	15.0	80.5	Self-report (human/environment)	Administrative medical records or disease registry	Morbidity	N/A	Light drinkers	Light drinkers	N/A	N/A	N/A	Any alcoholic beverages
Kim	2017	Atrial fibrillation and flutter	Republic of Korea	Retrospective Cohort	Both	15.0	82.3	Self-report (human/environment)	Administrative medical records or disease registry	Morbidity	N/A	Light drinkers	Light drinkers	N/A	N/A	N/A	Any alcoholic beverages

Table S9. Summary of study characteristics

Author	Year	Health outcome	Location	Study design	Sex	Age Start	Age End	Exposure Assessment	Outcome Assessment Method	Outcome type	Sample Size	Reference	Exposed group	Cases	Controls	Control Pool	Alcohol Type
Kim	2017	Atrial fibrillation and flutter	Republic of Korea	Retrospective Cohort	Both	15.0	93.5	Self-report (human/environment)	Administrative medical records or disease registry	Morbidity	N/A	Light drinkers	Moderate drinkers	N/A	N/A	N/A	Any alcoholic beverages
Kim	2017	Atrial fibrillation and flutter	Republic of Korea	Retrospective Cohort	Both	15.0	80.5	Self-report (human/environment)	Administrative medical records or disease registry	Morbidity	N/A	Light drinkers	Moderate drinkers	N/A	N/A	N/A	Any alcoholic beverages
Kim	2017	Atrial fibrillation and flutter	Republic of Korea	Retrospective Cohort	Both	15.0	82.3	Self-report (human/environment)	Administrative medical records or disease registry	Morbidity	N/A	Light drinkers	Moderate drinkers	N/A	N/A	N/A	Any alcoholic beverages
Lee	2015	Atrial fibrillation and flutter	Republic of Korea	Prospective Cohort	Both	66.0	66.0	Self-report (human/environment)	Physician diagnosis	Morbidity	N/A	Light drinkers	Moderate drinkers	N/A	N/A	N/A	Any alcoholic beverages
Marcus	2007	Atrial fibrillation and flutter	United States of America	Case-control	Both	15.0	99.0	N/A	N/A	Morbidity	N/A	Non-drinkers	Light drinkers	N/A	N/A	N/A	Any alcoholic beverages
Martín-Pérez	2005	Atrial fibrillation and flutter	United Kingdom	Prospective Cohort	Both	20.0	90.0	Self-report (human/environment)	Administrative medical records or disease registry	Morbidity	4489	Non-drinkers	Moderate drinkers	N/A	N/A	N/A	Any alcoholic beverages
McManus	2013	Atrial fibrillation and flutter	United States of America	Prospective Cohort	Both	18.2	94.4	Self-report (human/environment)	Physician diagnosis	Morbidity	5220	Light drinkers	Light drinkers	N/A	N/A	N/A	Any alcoholic beverages
Morseth	2020	Atrial fibrillation and flutter	Denmark	Prospective Cohort	Both	40.0	49.0	Self-report (human/environment)	Administrative medical records or disease registry	Morbidity	66951	Light drinkers	Moderate drinkers	N/A	N/A	N/A	Any alcoholic beverages
Morseth	2020	Atrial fibrillation and flutter	Denmark	Prospective Cohort	Both	50.0	59.0	Self-report (human/environment)	Administrative medical records or disease registry	Morbidity	66951	Light drinkers	Moderate drinkers	N/A	N/A	N/A	Any alcoholic beverages

Table S9. Summary of study characteristics

Author	Year	Health outcome	Location	Study design	Sex	Age Start	Age End	Exposure Assessment	Outcome Assessment Method	Outcome type	Sample Size	Reference	Exposed group	Cases	Controls	Control Pool	Alcohol Type
Morseth	2020	Atrial fibrillation and flutter	Denmark	Prospective Cohort	Both	60.0	69.0	Self-report (human/environment)	Administrative medical records or disease registry	Morbidity	66951	Light drinkers	Moderate drinkers	N/A	N/A	N/A	Any alcoholic beverages
Morseth	2020	Atrial fibrillation and flutter	Denmark	Prospective Cohort	Both	70.0	99.0	Self-report (human/environment)	Administrative medical records or disease registry	Morbidity	66951	Light drinkers	Moderate drinkers	N/A	N/A	N/A	Any alcoholic beverages
Morseth	2020	Atrial fibrillation and flutter	Norway	Prospective Cohort	Both	40.0	49.0	Self-report (human/environment)	Administrative medical records or disease registry	Morbidity	66951	Light drinkers	Moderate drinkers	N/A	N/A	N/A	Any alcoholic beverages
Morseth	2020	Atrial fibrillation and flutter	Norway	Prospective Cohort	Both	50.0	59.0	Self-report (human/environment)	Administrative medical records or disease registry	Morbidity	66951	Light drinkers	Moderate drinkers	N/A	N/A	N/A	Any alcoholic beverages
Morseth	2020	Atrial fibrillation and flutter	Norway	Prospective Cohort	Both	60.0	69.0	Self-report (human/environment)	Administrative medical records or disease registry	Morbidity	66951	Light drinkers	Moderate drinkers	N/A	N/A	N/A	Any alcoholic beverages
Morseth	2020	Atrial fibrillation and flutter	Norway	Prospective Cohort	Both	70.0	99.0	Self-report (human/environment)	Administrative medical records or disease registry	Morbidity	66951	Light drinkers	Moderate drinkers	N/A	N/A	N/A	Any alcoholic beverages
Morseth	2020	Atrial fibrillation and flutter	Sweden	Prospective Cohort	Both	40.0	49.0	Self-report (human/environment)	Administrative medical records or disease registry	Morbidity	66951	Light drinkers	Moderate drinkers	N/A	N/A	N/A	Any alcoholic beverages
Morseth	2020	Atrial fibrillation and flutter	Sweden	Prospective Cohort	Both	50.0	59.0	Self-report (human/environment)	Administrative medical records or disease registry	Morbidity	66951	Light drinkers	Moderate drinkers	N/A	N/A	N/A	Any alcoholic beverages

Table S9. Summary of study characteristics

Author	Year	Health outcome	Location	Study design	Sex	Age Start	Age End	Exposure Assessment	Outcome Assessment Method	Outcome type	Sample Size	Reference	Exposed group	Cases	Controls	Control Pool	Alcohol Type
Morseth	2020	Atrial fibrillation and flutter	Sweden	Prospective Cohort	Both	60.0	69.0	Self-report (human/environment)	Administrative medical records or disease registry	Morbidity	66951	Light drinkers	Moderate drinkers	N/A	N/A	N/A	Any alcoholic beverages
Morseth	2020	Atrial fibrillation and flutter	Sweden	Prospective Cohort	Both	70.0	99.0	Self-report (human/environment)	Administrative medical records or disease registry	Morbidity	66951	Light drinkers	Moderate drinkers	N/A	N/A	N/A	Any alcoholic beverages
Morseth	2020	Atrial fibrillation and flutter	Italy	Prospective Cohort	Both	40.0	49.0	Self-report (human/environment)	Administrative medical records or disease registry	Morbidity	66951	Light drinkers	Moderate drinkers	N/A	N/A	N/A	Any alcoholic beverages
Morseth	2020	Atrial fibrillation and flutter	Italy	Prospective Cohort	Both	50.0	59.0	Self-report (human/environment)	Administrative medical records or disease registry	Morbidity	66951	Light drinkers	Moderate drinkers	N/A	N/A	N/A	Any alcoholic beverages
Morseth	2020	Atrial fibrillation and flutter	Italy	Prospective Cohort	Both	60.0	69.0	Self-report (human/environment)	Administrative medical records or disease registry	Morbidity	66951	Light drinkers	Moderate drinkers	N/A	N/A	N/A	Any alcoholic beverages
Morseth	2020	Atrial fibrillation and flutter	Italy	Prospective Cohort	Both	70.0	99.0	Self-report (human/environment)	Administrative medical records or disease registry	Morbidity	66951	Light drinkers	Moderate drinkers	N/A	N/A	N/A	Any alcoholic beverages
Morseth	2020	Atrial fibrillation and flutter	Finland	Prospective Cohort	Both	40.0	49.0	Self-report (human/environment)	Administrative medical records or disease registry	Morbidity	66951	Light drinkers	Moderate drinkers	N/A	N/A	N/A	Any alcoholic beverages
Morseth	2020	Atrial fibrillation and flutter	Finland	Prospective Cohort	Both	50.0	59.0	Self-report (human/environment)	Administrative medical records or disease registry	Morbidity	66951	Light drinkers	Moderate drinkers	N/A	N/A	N/A	Any alcoholic beverages

Table S9. Summary of study characteristics

Author	Year	Health outcome	Location	Study design	Sex	Age Start	Age End	Exposure Assessment	Outcome Assessment Method	Outcome type	Sample Size	Reference	Exposed group	Cases	Controls	Control Pool	Alcohol Type
Morseth	2020	Atrial fibrillation and flutter	Finland	Prospective Cohort	Both	60.0	69.0	Self-report (human/environment)	Administrative medical records or disease registry	Morbidity	66951	Light drinkers	Moderate drinkers	N/A	N/A	N/A	Any alcoholic beverages
Morseth	2020	Atrial fibrillation and flutter	Finland	Prospective Cohort	Both	70.0	99.0	Self-report (human/environment)	Administrative medical records or disease registry	Morbidity	66951	Light drinkers	Moderate drinkers	N/A	N/A	N/A	Any alcoholic beverages
Park	2012	Atrial fibrillation and flutter	Republic of Korea	Prospective Cohort	Both	22.0	93.4	Self-report (human/environment)	Administrative medical records or disease registry	Morbidity	2551046	Non-drinkers	Light drinkers	N/A	N/A	N/A	Any alcoholic beverages
Psaty	1993	Atrial fibrillation and flutter	United States of America	Prospective Cohort	Both	65.0	99.0	Self-report (human/environment)	Physician diagnosis	Morbidity	4292	Light drinkers	Light drinkers	N/A	N/A	N/A	Any alcoholic beverages
Ruigomez	1997	Atrial fibrillation and flutter	United Kingdom	Case-control	Both	15.0	99.0	N/A	N/A	Morbidity	N/A	Non-drinkers	Heavy drinkers	437	1698	N/A	Any alcoholic beverages
Ruigomez	1997	Atrial fibrillation and flutter	United Kingdom	Case-control	Both	15.0	99.0	N/A	N/A	Morbidity	N/A	Non-drinkers	Moderate drinkers	468	1970	N/A	Any alcoholic beverages
Ruigomez	1997	Atrial fibrillation and flutter	United Kingdom	Case-control	Both	15.0	99.0	N/A	N/A	Morbidity	N/A	Non-drinkers	Light drinkers	545	2392	N/A	Any alcoholic beverages
Siddiqi	2020	Atrial fibrillation and flutter	United States of America	Prospective Cohort	Female	44.7	87.3	Self-report (human/environment)	Self-report	Morbidity	N/A	Light drinkers	Light drinkers	N/A	N/A	N/A	Any alcoholic beverages
Siddiqi	2020	Atrial fibrillation and flutter	United States of America	Prospective Cohort	Female	47.7	88.5	Self-report (human/environment)	Self-report	Morbidity	N/A	Light drinkers	Light drinkers	N/A	N/A	N/A	Any alcoholic beverages
Siddiqi	2020	Atrial fibrillation and flutter	United States of America	Prospective Cohort	Male	44.7	87.3	Self-report (human/environment)	Self-report	Morbidity	N/A	Light drinkers	Light drinkers	N/A	N/A	N/A	Any alcoholic beverages

Table S9. Summary of study characteristics

Author	Year	Health outcome	Location	Study design	Sex	Age Start	Age End	Exposure Assessment	Outcome Assessment Method	Outcome type	Sample Size	Reference	Exposed group	Cases	Controls	Control Pool	Alcohol Type
Siddiqi	2020	Atrial fibrillation and flutter	United States of America	Prospective Cohort	Male	47.7	88.5	Self-report (human/environment)	Self-report	Morbidity	N/A	Light drinkers	Light drinkers	N/A	N/A	N/A	Any alcoholic beverages
Tu	2020	Atrial fibrillation and flutter	United Kingdom	Prospective Cohort	Both	34.5	82.5	Self-report (human/environment)	Physician diagnosis	Morbidity	403281	Light drinkers	Light drinkers	N/A	N/A	N/A	Any alcoholic beverages
Tu	2020	Atrial fibrillation and flutter	United Kingdom	Prospective Cohort	Both	38.6	77.6	Self-report (human/environment)	Physician diagnosis	Morbidity	403281	Light drinkers	Light drinkers	N/A	N/A	N/A	Any alcoholic beverages
Tu	2020	Atrial fibrillation and flutter	United Kingdom	Prospective Cohort	Both	39.1	77.2	Self-report (human/environment)	Physician diagnosis	Morbidity	403281	Light drinkers	Light drinkers	N/A	N/A	N/A	Any alcoholic beverages
Tu	2020	Atrial fibrillation and flutter	United Kingdom	Prospective Cohort	Both	38.8	76.6	Self-report (human/environment)	Physician diagnosis	Morbidity	403281	Light drinkers	Light drinkers	N/A	N/A	N/A	Any alcoholic beverages
Tu	2020	Atrial fibrillation and flutter	United Kingdom	Prospective Cohort	Both	34.5	82.5	Self-report (human/environment)	Physician diagnosis	Morbidity	403281	Light drinkers	Moderate drinkers	N/A	N/A	N/A	Any alcoholic beverages
Tu	2020	Atrial fibrillation and flutter	United Kingdom	Prospective Cohort	Both	38.6	77.6	Self-report (human/environment)	Physician diagnosis	Morbidity	403281	Light drinkers	Moderate drinkers	N/A	N/A	N/A	Any alcoholic beverages
Tu	2020	Atrial fibrillation and flutter	United Kingdom	Prospective Cohort	Both	39.1	77.2	Self-report (human/environment)	Physician diagnosis	Morbidity	403281	Light drinkers	Moderate drinkers	N/A	N/A	N/A	Any alcoholic beverages
Tu	2020	Atrial fibrillation and flutter	United Kingdom	Prospective Cohort	Both	38.8	76.6	Self-report (human/environment)	Physician diagnosis	Morbidity	403281	Light drinkers	Moderate drinkers	N/A	N/A	N/A	Any alcoholic beverages
Adami	1985	Breast cancer	Global	Case-control	Both	15.0	99.0	N/A	N/A	Morbidity	N/A	Non-drinkers	Light drinkers	172	213	N/A	Any alcoholic beverages
Bassett	1994	Breast cancer	Australia	Prospective Cohort	Female	15.0	99.0	Self-report (human/environment)	Administrative medical records or disease registry	Morbidity	22756	Non-drinkers	Light drinkers	N/A	N/A	N/A	Any alcoholic beverages

Table S9. Summary of study characteristics

Author	Year	Health outcome	Location	Study design	Sex	Age Start	Age End	Exposure Assessment	Outcome Assessment Method	Outcome type	Sample Size	Reference	Exposed group	Cases	Controls	Control Pool	Alcohol Type
Bassett	1994	Breast cancer	Australia	Prospective Cohort	Female	15.0	99.0	Self-report (human/environment)	Administrative medical records or disease registry	Morbidity	22756	Non-drinkers	Moderate drinkers	N/A	N/A	N/A	Any alcoholic beverages
Benzon	2006	Breast cancer	Denmark	Nested Case-control	Both	15.0	99.0	N/A	N/A	Morbidity	N/A	Non-drinkers	Light drinkers	183	225	N/A	Any alcoholic beverages
Berstad	2003	Breast cancer	United States of America	Case-control	Both	15.0	99.0	N/A	N/A	Morbidity	N/A	Non-drinkers	Moderate drinkers	674	156	N/A	Any alcoholic beverages
Bowlin	1986	Breast cancer	United States of America	Case-control	Both	15.0	99.0	N/A	N/A	Morbidity	N/A	Non-drinkers	Light drinkers	664	664	N/A	Any alcoholic beverages
Chen	2008	Breast cancer	United States of America	Prospective Cohort	Both	15.0	99.0	N/A	N/A	Morbidity	N/A	Non-drinkers	Light drinkers	3792	N/A	N/A	Any alcoholic beverages
Donat-Vargas	2011	Breast cancer	Spain	Case-control	Female	15.0	99.0	Self-report (human/environment)	Physician diagnosis	Morbidity	724	Light drinkers	Light drinkers	N/A	N/A	From the community, general population	Any alcoholic beverages
Dumeaux	2001	Breast cancer	Norway	Prospective Cohort	Both	15.0	99.0	N/A	N/A	Morbidity	N/A	Non-drinkers	Light drinkers	340	N/A	N/A	Any alcoholic beverages
Ericson	2003	Breast cancer	Sweden	Prospective Cohort	Both	15.0	99.0	N/A	N/A	Morbidity	N/A	Non-drinkers	Moderate drinkers	41	N/A	N/A	Any alcoholic beverages
Ewertz	1984	Breast cancer	Denmark	Case-control	Both	15.0	99.0	N/A	N/A	Morbidity	N/A	Non-drinkers	Light drinkers	1038	921	N/A	Any alcoholic beverages
Ewertz	1984	Breast cancer	Denmark	Case-control	Both	15.0	99.0	N/A	N/A	Morbidity	N/A	Non-drinkers	Light drinkers	351	297	N/A	Any alcoholic beverages
Ewertz	1984	Breast cancer	Denmark	Case-control	Both	15.0	99.0	N/A	N/A	Morbidity	N/A	Non-drinkers	Light drinkers	579	502	N/A	Any alcoholic beverages
Ewertz	1984	Breast cancer	Denmark	Case-control	Both	15.0	99.0	N/A	N/A	Morbidity	N/A	Non-drinkers	Light drinkers	1077	982	N/A	Any alcoholic beverages
Ewertz	1984	Breast cancer	Denmark	Case-control	Both	15.0	99.0	N/A	N/A	Morbidity	N/A	Non-drinkers	Light drinkers	1034	958	N/A	Any alcoholic beverages
Ewertz	1984	Breast cancer	Denmark	Case-control	Both	15.0	99.0	N/A	N/A	Morbidity	N/A	Non-drinkers	Light drinkers	339	299	N/A	Any alcoholic beverages

Table S9. Summary of study characteristics

Author	Year	Health outcome	Location	Study design	Sex	Age Start	Age End	Exposure Assessment	Outcome Assessment Method	Outcome type	Sample Size	Reference	Exposed group	Cases	Controls	Control Pool	Alcohol Type
Ewertz	1984	Breast cancer	Denmark	Case-control	Both	15.0	99.0	N/A	N/A	Morbidity	N/A	Non-drinkers	Light drinkers	1004	905	N/A	Any alcoholic beverages
Ewertz	1984	Breast cancer	Denmark	Case-control	Both	15.0	99.0	N/A	N/A	Morbidity	N/A	Non-drinkers	Light drinkers	516	430	N/A	Any alcoholic beverages
Ewertz	1984	Breast cancer	Denmark	Case-control	Both	15.0	99.0	N/A	N/A	Morbidity	N/A	Non-drinkers	Light drinkers	577	492	N/A	Any alcoholic beverages
Ewertz	1984	Breast cancer	Denmark	Case-control	Both	15.0	99.0	N/A	N/A	Morbidity	N/A	Non-drinkers	Light drinkers	430	387	N/A	Any alcoholic beverages
Ferraroni	1985	Breast cancer	Italy	Case-control	Both	15.0	99.0	N/A	N/A	Morbidity	N/A	Non-drinkers	Light drinkers	73	89	N/A	Any alcoholic beverages
Floud	2001	Breast cancer	United Kingdom	Prospective Cohort	Female	41.9	70.7	Self-report (human/environment)	Administrative medical records or disease registry	Morbidity	795121	Light drinkers	Light drinkers	N/A	N/A	N/A	Any alcoholic beverages
Floud	2001	Breast cancer	United Kingdom	Prospective Cohort	Female	42.0	70.2	Self-report (human/environment)	Administrative medical records or disease registry	Morbidity	795121	Light drinkers	Light drinkers	N/A	N/A	N/A	Any alcoholic beverages
Floud	2001	Breast cancer	United Kingdom	Prospective Cohort	Female	42.0	69.6	Self-report (human/environment)	Administrative medical records or disease registry	Morbidity	795121	Light drinkers	Light drinkers	N/A	N/A	N/A	Any alcoholic beverages
Floud	2001	Breast cancer	United Kingdom	Prospective Cohort	Female	41.9	70.7	Self-report (human/environment)	Administrative medical records or disease registry	Morbidity	795121	Light drinkers	Moderate drinkers	N/A	N/A	N/A	Any alcoholic beverages
Floud	2001	Breast cancer	United Kingdom	Prospective Cohort	Female	42.0	70.2	Self-report (human/environment)	Administrative medical records or disease registry	Morbidity	795121	Light drinkers	Moderate drinkers	N/A	N/A	N/A	Any alcoholic beverages

Table S9. Summary of study characteristics

Author	Year	Health outcome	Location	Study design	Sex	Age Start	Age End	Exposure Assessment	Outcome Assessment Method	Outcome type	Sample Size	Reference	Exposed group	Cases	Controls	Control Pool	Alcohol Type
Floud	2001	Breast cancer	United Kingdom	Prospective Cohort	Female	42.0	69.6	Self-report (human/environment)	Administrative medical records or disease registry	Morbidity	795121	Light drinkers	Moderate drinkers	N/A	N/A	N/A	Any alcoholic beverages
Gapstur	1989	Breast cancer	United States of America	Prospective Cohort	Both	15.0	99.0	N/A	N/A	Morbidity	N/A	Non-drinkers	Light drinkers	283	N/A	N/A	Any alcoholic beverages
Harris	2008	Breast cancer	Sweden	Prospective Cohort	Both	15.0	99.0	N/A	N/A	Morbidity	N/A	Non-drinkers	Light drinkers	N/A	N/A	N/A	Any alcoholic beverages
Hirose	2000	Breast cancer	Japan	Case-control	Both	15.0	99.0	N/A	N/A	Morbidity	N/A	Non-drinkers	Light drinkers	N/A	N/A	N/A	Any alcoholic beverages
Hoyer	1989	Breast cancer	Denmark	Prospective Cohort	Both	15.0	99.0	N/A	N/A	Morbidity	N/A	Non-drinkers	Light drinkers	N/A	N/A	N/A	Any alcoholic beverages
Jain	1993	Breast cancer	Canada	Prospective Cohort	Both	15.0	99.0	N/A	N/A	Morbidity	N/A	Non-drinkers	Moderate drinkers	75	N/A	N/A	Any alcoholic beverages
Jain	1993	Breast cancer	Canada	Prospective Cohort	Both	15.0	99.0	N/A	N/A	Morbidity	N/A	Non-drinkers	Light drinkers	84	N/A	N/A	Any alcoholic beverages
Jain	1993	Breast cancer	Canada	Prospective Cohort	Both	15.0	99.0	N/A	N/A	Morbidity	N/A	Non-drinkers	Light drinkers	162	N/A	N/A	Any alcoholic beverages
Karavasiloglou	2010	Breast cancer	United Kingdom	Prospective Cohort	Female	15.0	99.0	Self-report (human/environment)	Administrative medical records or disease registry	Morbidity	232848	Moderate drinkers	Light drinkers	N/A	N/A	N/A	Any alcoholic beverages
Kinney	1996	Breast cancer	United States of America	Case-control	Both	15.0	99.0	N/A	N/A	Morbidity	N/A	Non-drinkers	Moderate drinkers	288	329	N/A	Any alcoholic beverages
Kwan	2013	Breast cancer	United States of America	Prospective Cohort	Female	23.3	94.7	Disease Registry (human)	Self-report	Morbidity	3659	Light drinkers	Light drinkers	N/A	N/A	N/A	Any alcoholic beverages
Kwan	2013	Breast cancer	United States of America	Prospective Cohort	Female	15.0	99.0	Disease Registry (human)	Self-report	Morbidity	3659	Light drinkers	Light drinkers	N/A	N/A	N/A	Any alcoholic beverages
La	1988	Breast cancer	Italy	Case-control	Both	15.0	99.0	N/A	N/A	Morbidity	N/A	Non-drinkers	Light drinkers	N/A	N/A	N/A	Any alcoholic beverages

Table S9. Summary of study characteristics

Author	Year	Health outcome	Location	Study design	Sex	Age Start	Age End	Exposure Assessment	Outcome Assessment Method	Outcome type	Sample Size	Reference	Exposed group	Cases	Controls	Control Pool	Alcohol Type
Le	1980	Breast cancer	France	Case-control	Both	15.0	99.0	N/A	N/A	Morbidity	N/A	Non-drinkers	Light drinkers	494	930	N/A	Any alcoholic beverages
Le	1980	Breast cancer	France	Case-control	Both	15.0	99.0	N/A	N/A	Morbidity	N/A	Non-drinkers	Light drinkers	477	899	N/A	Any alcoholic beverages
Le	1980	Breast cancer	France	Case-control	Both	15.0	99.0	N/A	N/A	Morbidity	N/A	Non-drinkers	Light drinkers	340	718	N/A	Any alcoholic beverages
Le	1980	Breast cancer	France	Case-control	Both	15.0	99.0	N/A	N/A	Morbidity	N/A	Non-drinkers	Light drinkers	488	905	N/A	Any alcoholic beverages
Le	1980	Breast cancer	France	Case-control	Both	15.0	99.0	N/A	N/A	Morbidity	N/A	Non-drinkers	Light drinkers	443	859	N/A	Any alcoholic beverages
Levi	1995	Breast cancer	Switzerland	Case-control	Both	15.0	99.0	N/A	N/A	Morbidity	N/A	Non-drinkers	Light drinkers	214	284	N/A	Any alcoholic beverages
Li	1999	Breast cancer	United States of America	Case-control	Both	15.0	99.0	N/A	N/A	Morbidity	N/A	Non-drinkers	Light drinkers	538	581	N/A	Any alcoholic beverages
Li	1999	Breast cancer	United States of America	Case-control	Both	15.0	99.0	N/A	N/A	Morbidity	N/A	Non-drinkers	Light drinkers	592	652	N/A	Any alcoholic beverages
Li	2004	Breast cancer	United States of America	Prospective Cohort	Both	15.0	99.0	N/A	N/A	Morbidity	N/A	Non-drinkers	Light drinkers	1203	N/A	N/A	Any alcoholic beverages
Li	2004	Breast cancer	United States of America	Prospective Cohort	Both	15.0	99.0	N/A	N/A	Morbidity	N/A	Non-drinkers	Light drinkers	1338	N/A	N/A	Any alcoholic beverages
Li	2005	Breast cancer	United States of America	Prospective Cohort	Both	15.0	99.0	N/A	N/A	Morbidity	N/A	Non-drinkers	Light drinkers	459	N/A	N/A	Any alcoholic beverages
Li	2005	Breast cancer	United States of America	Prospective Cohort	Both	15.0	99.0	N/A	N/A	Morbidity	N/A	Non-drinkers	Light drinkers	883	N/A	N/A	Any alcoholic beverages
Li	2005	Breast cancer	United States of America	Prospective Cohort	Both	15.0	99.0	N/A	N/A	Morbidity	N/A	Non-drinkers	Light drinkers	987	N/A	N/A	Any alcoholic beverages
Lucas	1993	Breast cancer	United States of America	Case-control	Both	15.0	99.0	N/A	N/A	Morbidity	N/A	Non-drinkers	Light drinkers	6	373	N/A	Any alcoholic beverages
Lucas	1993	Breast cancer	United States of America	Case-control	Both	15.0	99.0	N/A	N/A	Morbidity	N/A	Non-drinkers	Light drinkers	13	736	N/A	Any alcoholic beverages
McDonald	1998	Breast cancer	United States of America	Case-control	Both	15.0	99.0	N/A	N/A	Morbidity	N/A	Non-drinkers	Light drinkers	3993	4169	N/A	Any alcoholic beverages

Table S9. Summary of study characteristics

Author	Year	Health outcome	Location	Study design	Sex	Age Start	Age End	Exposure Assessment	Outcome Assessment Method	Outcome type	Sample Size	Reference	Exposed group	Cases	Controls	Control Pool	Alcohol Type
Meara	1984	Breast cancer	United Kingdom	Case-control	Both	15.0	99.0	N/A	N/A	Morbidity	N/A	Non-drinkers	Light drinkers	N/A	N/A	N/A	Any alcoholic beverages
Meara	1984	Breast cancer	United Kingdom	Case-control	Both	15.0	99.0	N/A	N/A	Morbidity	N/A	Non-drinkers	Moderate drinkers	N/A	N/A	N/A	Any alcoholic beverages
Meulepas	2007	Breast cancer	United States of America	Case-control	Both	15.0	99.0	N/A	N/A	Morbidity	N/A	Non-drinkers	N/A	854	905	N/A	Any alcoholic beverages
Morch	2001	Breast cancer	Denmark	Prospective Cohort	Both	15.0	99.0	N/A	N/A	Morbidity	N/A	Light drinkers	Light drinkers	157	N/A	N/A	Any alcoholic beverages
Morch	2001	Breast cancer	Denmark	Prospective Cohort	Both	15.0	99.0	N/A	N/A	Morbidity	N/A	Light drinkers	Light drinkers	160	N/A	N/A	Any alcoholic beverages
Morch	2001	Breast cancer	Denmark	Prospective Cohort	Both	15.0	99.0	N/A	N/A	Morbidity	N/A	Light drinkers	Light drinkers	139	N/A	N/A	Any alcoholic beverages
Morch	2001	Breast cancer	Denmark	Prospective Cohort	Both	15.0	99.0	N/A	N/A	Morbidity	N/A	Light drinkers	Heavy drinkers	107	N/A	N/A	Any alcoholic beverages
Newcomb	2000	Breast cancer	United States of America	Case-control	Both	15.0	99.0	N/A	N/A	Morbidity	N/A	Non-drinkers	Light drinkers	1824	2172	N/A	Any alcoholic beverages
Newcomb	2000	Breast cancer	United States of America	Case-control	Both	15.0	99.0	N/A	N/A	Morbidity	N/A	Non-drinkers	Light drinkers	2572	3110	N/A	Any alcoholic beverages
Newcomb	2000	Breast cancer	United States of America	Case-control	Both	15.0	99.0	N/A	N/A	Morbidity	N/A	Non-drinkers	Light drinkers	3251	4091	N/A	Any alcoholic beverages
Nielsen	2002	Breast cancer	Denmark	Prospective Cohort	Both	15.0	99.0	N/A	N/A	Morbidity	N/A	Light drinkers	Light drinkers	208	N/A	N/A	Any alcoholic beverages
Ong	2015	Breast cancer	Denmark	Prospective Cohort	Female	15.0	99.0	Self-report (human/environment)	Administrative medical records or disease registry	Morbidity	65126	Non-drinkers	Light drinkers	N/A	N/A	N/A	Any alcoholic beverages
Ong	2015	Breast cancer	Denmark	Prospective Cohort	Female	15.0	99.0	Self-report (human/environment)	Administrative medical records or disease registry	Morbidity	60903	Non-drinkers	Light drinkers	N/A	N/A	N/A	Any alcoholic beverages

Table S9. Summary of study characteristics

Author	Year	Health outcome	Location	Study design	Sex	Age Start	Age End	Exposure Assessment	Outcome Assessment Method	Outcome type	Sample Size	Reference	Exposed group	Cases	Controls	Control Pool	Alcohol Type
Ong	2015	Breast cancer	Denmark	Prospective Cohort	Female	15.0	99.0	Self-report (human/environment)	Administrative medical records or disease registry	Morbidity	62867	Non-drinkers	Light drinkers	N/A	N/A	N/A	Any alcoholic beverages
Richardson	1986	Breast cancer	France	Case-control	Both	15.0	99.0	N/A	N/A	Morbidity	N/A	Light drinkers	N/A	178	299	N/A	Any alcoholic beverages
Rosenberg	1986	Breast cancer	Canada	Case-control	Both	15.0	99.0	N/A	N/A	Morbidity	N/A	Light drinkers	Moderate drinkers	248	424	N/A	Any alcoholic beverages
Rosenberg	1986	Breast cancer	Canada	Case-control	Both	15.0	99.0	N/A	N/A	Morbidity	N/A	Light drinkers	Light drinkers	248	424	N/A	Any alcoholic beverages
Rosenberg	1986	Breast cancer	Canada	Case-control	Both	15.0	99.0	N/A	N/A	Morbidity	N/A	Light drinkers	Moderate drinkers	259	466	N/A	Any alcoholic beverages
Rosenberg	1986	Breast cancer	Canada	Case-control	Both	15.0	99.0	N/A	N/A	Morbidity	N/A	Light drinkers	Light drinkers	259	466	N/A	Any alcoholic beverages
Rosenberg	1995	Breast cancer	Sweden	Case-control	Both	15.0	99.0	N/A	N/A	Morbidity	N/A	Non-drinkers	Light drinkers	224	2064	N/A	Any alcoholic beverages
Rosenberg	1995	Breast cancer	Sweden	Case-control	Both	15.0	99.0	N/A	N/A	Morbidity	N/A	Non-drinkers	Light drinkers	509	1239	N/A	Any alcoholic beverages
Rosenberg	1995	Breast cancer	Sweden	Case-control	Both	15.0	99.0	N/A	N/A	Morbidity	N/A	Non-drinkers	Light drinkers	860	2064	N/A	Any alcoholic beverages
Rosenberg	1995	Breast cancer	Sweden	Case-control	Both	15.0	99.0	N/A	N/A	Morbidity	N/A	Non-drinkers	Light drinkers	136	1355	N/A	Any alcoholic beverages
Rosenberg	1995	Breast cancer	Sweden	Case-control	Both	15.0	99.0	N/A	N/A	Morbidity	N/A	Non-drinkers	Light drinkers	598	2064	N/A	Any alcoholic beverages
Rosenberg	1995	Breast cancer	Sweden	Case-control	Both	15.0	99.0	N/A	N/A	Morbidity	N/A	Non-drinkers	Light drinkers	388	1355	N/A	Any alcoholic beverages
Rustagi	2013	Breast cancer	United States of America	Case-control	Female	15.0	99.0	Self-report (human/environment)	Administrative medical records or disease registry	Morbidity	4548	Non-drinkers	Light drinkers	N/A	N/A	From the community, general population	Any alcoholic beverages

Table S9. Summary of study characteristics

Author	Year	Health outcome	Location	Study design	Sex	Age Start	Age End	Exposure Assessment	Outcome Assessment Method	Outcome type	Sample Size	Reference	Exposed group	Cases	Controls	Control Pool	Alcohol Type
Sarich	2013	Breast cancer	Australia	Prospective Cohort	Female	28.6	98.2	Self-report (human/environment)	Administrative medical records or disease registry	Morbidity	226162	Light drinkers	Light drinkers	N/A	N/A	N/A	Any alcoholic beverages
Sarich	2013	Breast cancer	Australia	Prospective Cohort	Female	28.8	94.8	Self-report (human/environment)	Administrative medical records or disease registry	Morbidity	226162	Light drinkers	Light drinkers	N/A	N/A	N/A	Any alcoholic beverages
Sarich	2013	Breast cancer	Australia	Prospective Cohort	Female	30.3	91.5	Self-report (human/environment)	Administrative medical records or disease registry	Morbidity	226162	Light drinkers	Light drinkers	N/A	N/A	N/A	Any alcoholic beverages
Sarich	2013	Breast cancer	Australia	Prospective Cohort	Female	32.7	87.3	Self-report (human/environment)	Administrative medical records or disease registry	Morbidity	226162	Light drinkers	Light drinkers	N/A	N/A	N/A	Any alcoholic beverages
Sarich	2013	Breast cancer	Australia	Prospective Cohort	Female	29.1	94.5	Self-report (human/environment)	Administrative medical records or disease registry	Morbidity	226162	Light drinkers	Light drinkers	N/A	N/A	N/A	Any alcoholic beverages
Sarich	2013	Breast cancer	Australia	Prospective Cohort	Female	31.3	90.1	Self-report (human/environment)	Administrative medical records or disease registry	Morbidity	226162	Light drinkers	Light drinkers	N/A	N/A	N/A	Any alcoholic beverages
Simon	1987	Breast cancer	United States of America	Prospective Cohort	Both	15.0	99.0	N/A	N/A	Morbidity	N/A	Non-drinkers	Light drinkers	N/A	N/A	N/A	Any alcoholic beverages
Smith	1985	Breast cancer	United Kingdom	Case-control	Both	15.0	99.0	N/A	N/A	Morbidity	N/A	Non-drinkers	Light drinkers	442	433	N/A	Any alcoholic beverages
Sneyd	1987	Breast cancer	New Zealand	Case-control	Both	15.0	99.0	N/A	N/A	Morbidity	N/A	Non-drinkers	Light drinkers	490	983	N/A	Any alcoholic beverages
Sneyd	1987	Breast cancer	New Zealand	Case-control	Both	15.0	99.0	N/A	N/A	Morbidity	N/A	Non-drinkers	Light drinkers	81	172	N/A	Any alcoholic beverages

Table S9. Summary of study characteristics

Author	Year	Health outcome	Location	Study design	Sex	Age Start	Age End	Exposure Assessment	Outcome Assessment Method	Outcome type	Sample Size	Reference	Exposed group	Cases	Controls	Control Pool	Alcohol Type
Sneyd	1987	Breast cancer	New Zealand	Case-control	Both	15.0	99.0	N/A	N/A	Morbidity	N/A	Non-drinkers	Light drinkers	171	367	N/A	Any alcoholic beverages
Song	2013	Breast cancer	Republic of Korea	Case-control	Female	15.0	99.0	Self-report (human/environment)	Self-report	Morbidity	36290	Non-drinkers	Light drinkers	N/A	N/A	From the community, general population	Any alcoholic beverages
Suzuki	2004	Breast cancer	Sweden	Prospective Cohort	Both	15.0	99.0	N/A	N/A	Morbidity	N/A	Non-drinkers	Light drinkers	790	N/A	N/A	Any alcoholic beverages
Suzuki	2004	Breast cancer	Sweden	Prospective Cohort	Both	15.0	99.0	N/A	N/A	Morbidity	N/A	Non-drinkers	Light drinkers	657	N/A	N/A	Any alcoholic beverages
Suzuki	2006	Breast cancer	Japan	Prospective Cohort	Both	15.0	99.0	N/A	N/A	Morbidity	N/A	Non-drinkers	Light drinkers	422	N/A	N/A	Any alcoholic beverages
Thun	1991	Breast cancer	United States of America	Prospective Cohort	Both	15.0	99.0	N/A	N/A	Morbidity	N/A	Non-drinkers	Light drinkers	N/A	N/A	N/A	Any alcoholic beverages
Thun	1991	Breast cancer	United States of America	Prospective Cohort	Both	15.0	99.0	N/A	N/A	Morbidity	N/A	Non-drinkers	Heavy drinkers	N/A	N/A	N/A	Any alcoholic beverages
Tjonneland	2004	Breast cancer	Global	Prospective Cohort	Both	15.0	99.0	N/A	N/A	Morbidity	N/A	Light drinkers	Heavy drinkers	907	N/A	N/A	Any alcoholic beverages
Tjonneland	2004	Breast cancer	Global	Prospective Cohort	Both	15.0	99.0	N/A	N/A	Morbidity	N/A	Light drinkers	Light drinkers	1313	N/A	N/A	Any alcoholic beverages
Tjonneland	2004	Breast cancer	Global	Prospective Cohort	Both	15.0	99.0	N/A	N/A	Morbidity	N/A	Light drinkers	Light drinkers	1424	N/A	N/A	Any alcoholic beverages
Tjonneland	2004	Breast cancer	Global	Prospective Cohort	Both	15.0	99.0	N/A	N/A	Morbidity	N/A	Light drinkers	Moderate drinkers	912	N/A	N/A	Any alcoholic beverages
Tjonneland	2004	Breast cancer	Global	Prospective Cohort	Both	15.0	99.0	N/A	N/A	Morbidity	N/A	Light drinkers	Light drinkers	1432	N/A	N/A	Any alcoholic beverages
Tjonneland	2004	Breast cancer	Global	Prospective Cohort	Both	15.0	99.0	N/A	N/A	Morbidity	N/A	Light drinkers	Moderate drinkers	855	N/A	N/A	Any alcoholic beverages
Tjonneland	2004	Breast cancer	Global	Prospective Cohort	Both	15.0	99.0	N/A	N/A	Morbidity	N/A	Light drinkers	Light drinkers	1460	N/A	N/A	Any alcoholic beverages
Toniolo	1984	Breast cancer	Italy	Case-control	Both	15.0	99.0	N/A	N/A	Morbidity	N/A	Non-drinkers	Moderate drinkers	126	270	N/A	Any alcoholic beverages

Table S9. Summary of study characteristics

Author	Year	Health outcome	Location	Study design	Sex	Age Start	Age End	Exposure Assessment	Outcome Assessment Method	Outcome type	Sample Size	Reference	Exposed group	Cases	Controls	Control Pool	Alcohol Type
Viladiu	1989	Breast cancer	Spain	Case-control	Both	15.0	99.0	N/A	N/A	Morbidity	N/A	Non-drinkers	Light drinkers	253	202	N/A	Any alcoholic beverages
Viladiu	1989	Breast cancer	Spain	Case-control	Both	15.0	99.0	N/A	N/A	Morbidity	N/A	Non-drinkers	Light drinkers	262	303	N/A	Any alcoholic beverages
Wang	2004	Breast cancer	Global	Case-control	Both	15.0	99.0	N/A	N/A	Morbidity	N/A	Non-drinkers	Light drinkers	253	1633	N/A	Any alcoholic beverages
Zeinomar	2019	Breast cancer	United States of America	Prospective Cohort	Female	15.0	99.0	Self-report (human/environment)	Administrative medical records or disease registry	Morbidity	1926	Non-drinkers	Light drinkers	N/A	N/A	N/A	Any alcoholic beverages
Zhang	2005	Breast cancer	China	Case-control	Both	15.0	99.0	N/A	N/A	Morbidity	N/A	Non-drinkers	Light drinkers	688	571	N/A	Any alcoholic beverages
Zhang	2005	Breast cancer	China	Case-control	Both	15.0	99.0	N/A	N/A	Morbidity	N/A	Non-drinkers	Light drinkers	885	895	N/A	Any alcoholic beverages
van den Brandt	1989	Breast cancer	Netherlands	Case-cohort	Both	15.0	99.0	N/A	N/A	Morbidity	N/A	Non-drinkers	Light drinkers	127	N/A	N/A	Any alcoholic beverages
van't Veer	1987	Breast cancer	Netherlands	Case-control	Both	15.0	99.0	N/A	N/A	Morbidity	N/A	Non-drinkers	Light drinkers	N/A	N/A	N/A	Any alcoholic beverages
van't Veer	1987	Breast cancer	Netherlands	Case-control	Both	15.0	99.0	N/A	N/A	Morbidity	N/A	Non-drinkers	Moderate drinkers	N/A	N/A	N/A	Any alcoholic beverages
Becker	1992	Cirrhosis and other chronic liver diseases	Denmark	Prospective Cohort	Both	15.0	99.0	N/A	N/A	Morbidity	N/A	Light drinkers	Moderate drinkers	N/A	N/A	N/A	Any alcoholic beverages
Becker	1998	Cirrhosis and other chronic liver diseases	Denmark	Prospective Cohort	Both	15.0	99.0	N/A	N/A	Morbidity	N/A	Light drinkers	Light drinkers	12	5539	N/A	Any alcoholic beverages
Becker	1998	Cirrhosis and other chronic liver diseases	Denmark	Prospective Cohort	Both	15.0	99.0	N/A	N/A	Morbidity	N/A	Light drinkers	Moderate drinkers	44	1815	N/A	Any alcoholic beverages
Fuchs	1992	Cirrhosis and other chronic liver diseases	United States of America	Prospective Cohort	Female	30.0	55.0	Self-report (human/environment)	Death certificates	Morbidity	85709	Non-drinkers	Light drinkers	N/A	N/A	N/A	Any alcoholic beverages

Table S9. Summary of study characteristics

Author	Year	Health outcome	Location	Study design	Sex	Age Start	Age End	Exposure Assessment	Outcome Assessment Method	Outcome type	Sample Size	Reference	Exposed group	Cases	Controls	Control Pool	Alcohol Type
Fuchs	1992	Cirrhosis and other chronic liver diseases	United States of America	Prospective Cohort	Female	30.0	55.0	Self-report (human/environment)	Death certificates	Morbidity	85709	Non-drinkers	Moderate drinkers	N/A	N/A	N/A	Any alcoholic beverages
Setiawan	2013	Cirrhosis and other chronic liver diseases	United States of America	Prospective Cohort	Both	38.5	84.0	Self-report (human/environment)	Death certificates	Morbidity	18379	Non-drinkers	Light drinkers	N/A	N/A	N/A	Any alcoholic beverages
Setiawan	2013	Cirrhosis and other chronic liver diseases	United States of America	Prospective Cohort	Both	36.2	81.8	Self-report (human/environment)	Death certificates	Morbidity	18379	Non-drinkers	Light drinkers	N/A	N/A	N/A	Any alcoholic beverages
Tao	2018	Cirrhosis and other chronic liver diseases	United States of America	Prospective Cohort	Both	19.0	99.0	Self-report (human/environment)	Biomarker	Morbidity	4166	Non-drinkers	Moderate drinkers	N/A	N/A	N/A	Any alcoholic beverages
Choi	1990	Colon and rectum cancer	Republic of Korea	Case-control	Male	53.9	99.0	Self-report (human/environment)	Physician diagnosis	Morbidity	189	Non-drinkers	Moderate drinkers	N/A	N/A	Hospital Patients	Any alcoholic beverages
Choi	1990	Colon and rectum cancer	Republic of Korea	Case-control	Male	52.6	99.0	Self-report (human/environment)	Physician diagnosis	Morbidity	189	Non-drinkers	Moderate drinkers	N/A	N/A	Hospital Patients	Any alcoholic beverages
Choi	1990	Colon and rectum cancer	Republic of Korea	Case-control	Male	53.9	99.0	Self-report (human/environment)	Physician diagnosis	Morbidity	189	Non-drinkers	Heavy drinkers	N/A	N/A	Hospital Patients	Any alcoholic beverages
Choi	1990	Colon and rectum cancer	Republic of Korea	Case-control	Male	52.6	99.0	Self-report (human/environment)	Physician diagnosis	Morbidity	189	Non-drinkers	Heavy drinkers	N/A	N/A	Hospital Patients	Any alcoholic beverages
Choi	1990	Colon and rectum cancer	Republic of Korea	Case-control	Male	53.9	99.0	Self-report (human/environment)	Physician diagnosis	Morbidity	201	Non-drinkers	Moderate drinkers	N/A	N/A	Hospital Patients	Any alcoholic beverages
Choi	1990	Colon and rectum cancer	Republic of Korea	Case-control	Male	52.6	99.0	Self-report (human/environment)	Physician diagnosis	Morbidity	201	Non-drinkers	Moderate drinkers	N/A	N/A	Hospital Patients	Any alcoholic beverages
Choi	1990	Colon and rectum cancer	Republic of Korea	Case-control	Male	53.9	99.0	Self-report (human/environment)	Physician diagnosis	Morbidity	201	Non-drinkers	Heavy drinkers	N/A	N/A	Hospital Patients	Any alcoholic beverages
Choi	1990	Colon and rectum cancer	Republic of Korea	Case-control	Male	52.6	99.0	Self-report (human/environment)	Physician diagnosis	Morbidity	201	Non-drinkers	Heavy drinkers	N/A	N/A	Hospital Patients	Any alcoholic beverages

Table S9. Summary of study characteristics

Author	Year	Health outcome	Location	Study design	Sex	Age Start	Age End	Exposure Assessment	Outcome Assessment Method	Outcome type	Sample Size	Reference	Exposed group	Cases	Controls	Control Pool	Alcohol Type
Chyou	1995	Colon and rectum cancer	United States of America	Prospective Cohort	Both	15.0	99.0	N/A	N/A	Morbidity	N/A	Non-drinkers	Light drinkers	263	4403	N/A	Any alcoholic beverages
Crockett	2006	Colon and rectum cancer	United States of America	Case-control	Both	15.0	99.0	N/A	N/A	Morbidity	N/A	Non-drinkers	Light drinkers	729	621	N/A	Any alcoholic beverages
Flood	1998	Colon and rectum cancer	United States of America	Case-control	Both	15.0	99.0	N/A	N/A	Morbidity	N/A	Non-drinkers	Moderate drinkers	195	18559	N/A	Any alcoholic beverages
Flood	1998	Colon and rectum cancer	United States of America	Case-control	Both	15.0	99.0	N/A	N/A	Morbidity	N/A	Non-drinkers	Light drinkers	234	23013	N/A	Any alcoholic beverages
Floud	2001	Colon and rectum cancer	United Kingdom	Prospective Cohort	Female	41.9	70.7	Self-report (human/environment)	Administrative medical records or disease registry	Morbidity	795121	Light drinkers	Light drinkers	N/A	N/A	N/A	Any alcoholic beverages
Floud	2001	Colon and rectum cancer	United Kingdom	Prospective Cohort	Female	42.0	70.2	Self-report (human/environment)	Administrative medical records or disease registry	Morbidity	795121	Light drinkers	Light drinkers	N/A	N/A	N/A	Any alcoholic beverages
Floud	2001	Colon and rectum cancer	United Kingdom	Prospective Cohort	Female	42.0	69.6	Self-report (human/environment)	Administrative medical records or disease registry	Morbidity	795121	Light drinkers	Light drinkers	N/A	N/A	N/A	Any alcoholic beverages
Floud	2001	Colon and rectum cancer	United Kingdom	Prospective Cohort	Female	41.9	70.7	Self-report (human/environment)	Administrative medical records or disease registry	Morbidity	795121	Light drinkers	Moderate drinkers	N/A	N/A	N/A	Any alcoholic beverages
Floud	2001	Colon and rectum cancer	United Kingdom	Prospective Cohort	Female	42.0	70.2	Self-report (human/environment)	Administrative medical records or disease registry	Morbidity	795121	Light drinkers	Moderate drinkers	N/A	N/A	N/A	Any alcoholic beverages
Floud	2001	Colon and rectum cancer	United Kingdom	Prospective Cohort	Female	42.0	69.6	Self-report (human/environment)	Administrative medical records or disease registry	Morbidity	795121	Light drinkers	Moderate drinkers	N/A	N/A	N/A	Any alcoholic beverages

Table S9. Summary of study characteristics

Author	Year	Health outcome	Location	Study design	Sex	Age Start	Age End	Exposure Assessment	Outcome Assessment Method	Outcome type	Sample Size	Reference	Exposed group	Cases	Controls	Control Pool	Alcohol Type
Goldbohm	1989	Colon and rectum cancer	Netherlands	Case-control	Both	15.0	99.0	N/A	N/A	Morbidity	N/A	Non-drinkers	Light drinkers	95	517	N/A	Any alcoholic beverages
Goldbohm	1989	Colon and rectum cancer	Netherlands	Case-control	Both	15.0	99.0	N/A	N/A	Morbidity	N/A	Non-drinkers	Moderate drinkers	85	559	N/A	Any alcoholic beverages
Goldbohm	1989	Colon and rectum cancer	Netherlands	Case-control	Both	15.0	99.0	N/A	N/A	Morbidity	N/A	Non-drinkers	Moderate drinkers	103	542	N/A	Any alcoholic beverages
Goldbohm	1989	Colon and rectum cancer	Netherlands	Case-control	Both	15.0	99.0	N/A	N/A	Morbidity	N/A	Non-drinkers	Moderate drinkers	96	634	N/A	Any alcoholic beverages
Goldbohm	1989	Colon and rectum cancer	Netherlands	Case-control	Both	15.0	99.0	N/A	N/A	Morbidity	N/A	Non-drinkers	Light drinkers	138	1032	N/A	Any alcoholic beverages
Hatime	2020	Colon and rectum cancer	Morocco	Case-control	Both	15.0	98.3	Self-report (human/environment)	Physician diagnosis	Morbidity	1453	Light drinkers	Light drinkers	N/A	N/A	Facility Visitors	Any alcoholic beverages
Hur	2021	Colon and rectum cancer	United States of America	Prospective Cohort	Female	37.4	96.8	Self-report (human/environment)	Self-report	Morbidity	92858	Non-drinkers	Light drinkers	N/A	N/A	N/A	Any alcoholic beverages
Hur	2021	Colon and rectum cancer	United States of America	Prospective Cohort	Female	21.3	74.1	Self-report (human/environment)	Self-report	Morbidity	92858	Non-drinkers	Light drinkers	N/A	N/A	N/A	Any alcoholic beverages
Hur	2021	Colon and rectum cancer	United States of America	Prospective Cohort	Female	34.4	99.0	Self-report (human/environment)	Self-report	Morbidity	92858	Non-drinkers	Light drinkers	N/A	N/A	N/A	Any alcoholic beverages
Hur	2021	Colon and rectum cancer	United States of America	Prospective Cohort	Female	37.4	96.8	Self-report (human/environment)	Self-report	Morbidity	36393	Non-drinkers	Light drinkers	N/A	N/A	N/A	Any alcoholic beverages
Hur	2021	Colon and rectum cancer	United States of America	Prospective Cohort	Female	21.3	74.1	Self-report (human/environment)	Self-report	Morbidity	36393	Non-drinkers	Light drinkers	N/A	N/A	N/A	Any alcoholic beverages
Hur	2021	Colon and rectum cancer	United States of America	Prospective Cohort	Female	34.4	99.0	Self-report (human/environment)	Self-report	Morbidity	36393	Non-drinkers	Light drinkers	N/A	N/A	N/A	Any alcoholic beverages
Kabat	2000	Colon and rectum cancer	Canada	Prospective Cohort	Both	15.0	99.0	N/A	N/A	Morbidity	N/A	Non-drinkers	Moderate drinkers	N/A	N/A	N/A	Any alcoholic beverages

Table S9. Summary of study characteristics

Author	Year	Health outcome	Location	Study design	Sex	Age Start	Age End	Exposure Assessment	Outcome Assessment Method	Outcome type	Sample Size	Reference	Exposed group	Cases	Controls	Control Pool	Alcohol Type
Kabat	2000	Colon and rectum cancer	Canada	Prospective Cohort	Both	15.0	99.0	N/A	N/A	Morbidity	N/A	Non-drinkers	Light drinkers	N/A	N/A	N/A	Any alcoholic beverages
Kono	1983	Colon and rectum cancer	Japan	Prospective Cohort	Both	15.0	99.0	N/A	N/A	Morbidity	N/A	Non-drinkers	Moderate drinkers	N/A	N/A	N/A	Any alcoholic beverages
Kono	1983	Colon and rectum cancer	Japan	Prospective Cohort	Both	15.0	99.0	N/A	N/A	Morbidity	N/A	Non-drinkers	N/A	16	N/A	N/A	Any alcoholic beverages
Lightfoot	2000	Colon and rectum cancer	United Kingdom	Case-control	Both	15.0	99.0	N/A	N/A	Morbidity	N/A	Non-drinkers	Light drinkers	449	219	N/A	Any alcoholic beverages
Murata	1997	Colon and rectum cancer	Japan	Case-control	Both	15.0	99.0	N/A	N/A	Morbidity	N/A	Non-drinkers	Moderate drinkers	137	224	N/A	Any alcoholic beverages
Nakaya	1997	Colon and rectum cancer	Japan	Prospective Cohort	Both	15.0	99.0	N/A	N/A	Morbidity	N/A	Non-drinkers	Light drinkers	30	N/A	N/A	Any alcoholic beverages
Newcomb	1991	Colon and rectum cancer	United States of America	Case-control	Both	15.0	99.0	N/A	N/A	Morbidity	N/A	Non-drinkers	Light drinkers	241	633	N/A	Any alcoholic beverages
Newcomb	1991	Colon and rectum cancer	United States of America	Case-control	Both	15.0	99.0	N/A	N/A	Morbidity	N/A	Non-drinkers	Light drinkers	294	809	N/A	Any alcoholic beverages
Newcomb	1991	Colon and rectum cancer	United States of America	Case-control	Both	15.0	99.0	N/A	N/A	Morbidity	N/A	Non-drinkers	Light drinkers	501	1502	N/A	Any alcoholic beverages
Pedersen	1993	Colon and rectum cancer	Denmark	Prospective Cohort	Both	15.0	99.0	N/A	N/A	Morbidity	N/A	Light drinkers	Moderate drinkers	235	10358	N/A	Any alcoholic beverages
Pedersen	1993	Colon and rectum cancer	Denmark	Prospective Cohort	Both	15.0	99.0	N/A	N/A	Morbidity	N/A	Light drinkers	Heavy drinkers	149	6721	N/A	Any alcoholic beverages
Pedersen	1993	Colon and rectum cancer	Denmark	Prospective Cohort	Both	15.0	99.0	N/A	N/A	Morbidity	N/A	Light drinkers	Light drinkers	313	15121	N/A	Any alcoholic beverages
Pedersen	1993	Colon and rectum cancer	Denmark	Prospective Cohort	Both	15.0	99.0	N/A	N/A	Morbidity	N/A	Light drinkers	Light drinkers	244	11464	N/A	Any alcoholic beverages
Peters	1982	Colon and rectum cancer	United States of America	Prospective Cohort	Both	15.0	99.0	N/A	N/A	Morbidity	N/A	Light drinkers	Moderate drinkers	100	101	N/A	Any alcoholic beverages
Sarich	2013	Colon and rectum cancer	Australia	Prospective Cohort	Both	25.0	85.0	Self-report (human/environment)	Administrative medical records or disease registry	Morbidity	226162	Light drinkers	Light drinkers	N/A	N/A	N/A	Any alcoholic beverages

Table S9. Summary of study characteristics

Author	Year	Health outcome	Location	Study design	Sex	Age Start	Age End	Exposure Assessment	Outcome Assessment Method	Outcome type	Sample Size	Reference	Exposed group	Cases	Controls	Control Pool	Alcohol Type
Sarich	2013	Colon and rectum cancer	Australia	Prospective Cohort	Both	25.0	85.0	Self-report (human/environment)	Administrative medical records or disease registry	Morbidity	226162	Light drinkers	Heavy drinkers	N/A	N/A	N/A	Any alcoholic beverages
Seol	2004	Colon and rectum cancer	Republic of Korea	Case-control	Both	30.2	87.8	Self-report (human/environment)	Physician diagnosis	Morbidity	1001	Non-drinkers	Light drinkers	N/A	N/A	Hospital Patients	Any alcoholic beverages
Sharpe	1985	Colon and rectum cancer	Canada	Case-control	Both	15.0	99.0	N/A	N/A	Morbidity	N/A	Non-drinkers	Moderate drinkers	183	177	N/A	Any alcoholic beverages
Stern	2002	Colon and rectum cancer	Singapore	Case-control	Both	15.0	99.0	N/A	N/A	Morbidity	N/A	Non-drinkers	Light drinkers	276	1129	N/A	Any alcoholic beverages
Stern	2002	Colon and rectum cancer	Singapore	Case-control	Both	15.0	99.0	N/A	N/A	Morbidity	N/A	Non-drinkers	Light drinkers	259	1012	N/A	Any alcoholic beverages
Tsong	2004	Colon and rectum cancer	Singapore	Prospective Cohort	Both	15.0	99.0	N/A	N/A	Morbidity	N/A	Non-drinkers	Light drinkers	775	N/A	N/A	Any alcoholic beverages
Tverdal	2018	Colon and rectum cancer	Norway	Prospective Cohort	Male	20.0	79.0	Self-report (human/environment)	Administrative medical records or disease registry	Morbidity	250010	Non-drinkers	Light drinkers	N/A	N/A	N/A	Any alcoholic beverages
Tverdal	2018	Colon and rectum cancer	Norway	Prospective Cohort	Female	20.0	79.0	Self-report (human/environment)	Administrative medical records or disease registry	Morbidity	250010	Non-drinkers	Light drinkers	N/A	N/A	N/A	Any alcoholic beverages
Wang	2004	Colon and rectum cancer	Global	Case-control	Both	15.0	99.0	N/A	N/A	Morbidity	N/A	Non-drinkers	Light drinkers	224	1810	N/A	Any alcoholic beverages
Wang	2004	Colon and rectum cancer	Global	Case-control	Both	15.0	99.0	N/A	N/A	Morbidity	N/A	Non-drinkers	Light drinkers	204	1887	N/A	Any alcoholic beverages
Wang	2004	Colon and rectum cancer	Global	Case-control	Both	15.0	99.0	N/A	N/A	Morbidity	N/A	Non-drinkers	Light drinkers	199	2028	N/A	Any alcoholic beverages
Wu	1985	Colon and rectum cancer	United States of America	Prospective Cohort	Both	15.0	99.0	N/A	N/A	Morbidity	N/A	Non-drinkers	Moderate drinkers	9082	N/A	N/A	Any alcoholic beverages

Table S9. Summary of study characteristics

Author	Year	Health outcome	Location	Study design	Sex	Age Start	Age End	Exposure Assessment	Outcome Assessment Method	Outcome type	Sample Size	Reference	Exposed group	Cases	Controls	Control Pool	Alcohol Type
Yamada	1993	Colon and rectum cancer	Japan	Case-control	Both	15.0	99.0	N/A	N/A	Morbidity	N/A	Non-drinkers	Light drinkers	23	47	N/A	Any alcoholic beverages
Yi	2005	Colon and rectum cancer	Republic of Korea	Prospective Cohort	Both	15.0	99.0	N/A	N/A	Morbidity	N/A	Non-drinkers	Heavy drinkers	14	N/A	N/A	Any alcoholic beverages
Baik	2012	Diabetes mellitus type 2	Republic of Korea	Prospective Cohort	Both	40.0	69.0	Self-report (human/environment)	Biomarker	Morbidity	2338	Non-drinkers	Light drinkers	N/A	N/A	N/A	Any alcoholic beverages
Beulens	2000	Diabetes mellitus type 2	Denmark	Prospective Cohort	Male	31.0	70.0	Self-report (human/environment)	Administrative medical records or disease registry	Morbidity	N/A	Light drinkers	Light drinkers	N/A	N/A	N/A	Any alcoholic beverages
Beulens	2000	Diabetes mellitus type 2	Denmark	Prospective Cohort	Male	31.0	70.0	Self-report (human/environment)	Administrative medical records or disease registry	Morbidity	N/A	Light drinkers	Heavy drinkers	N/A	N/A	N/A	Any alcoholic beverages
Beulens	2000	Diabetes mellitus type 2	France	Prospective Cohort	Male	31.0	70.0	Self-report (human/environment)	Administrative medical records or disease registry	Morbidity	N/A	Light drinkers	Light drinkers	N/A	N/A	N/A	Any alcoholic beverages
Beulens	2000	Diabetes mellitus type 2	France	Prospective Cohort	Male	31.0	70.0	Self-report (human/environment)	Administrative medical records or disease registry	Morbidity	N/A	Light drinkers	Heavy drinkers	N/A	N/A	N/A	Any alcoholic beverages
Beulens	2000	Diabetes mellitus type 2	Germany	Prospective Cohort	Male	31.0	70.0	Self-report (human/environment)	Administrative medical records or disease registry	Morbidity	N/A	Light drinkers	Light drinkers	N/A	N/A	N/A	Any alcoholic beverages
Beulens	2000	Diabetes mellitus type 2	Germany	Prospective Cohort	Male	31.0	70.0	Self-report (human/environment)	Administrative medical records or disease registry	Morbidity	N/A	Light drinkers	Heavy drinkers	N/A	N/A	N/A	Any alcoholic beverages

Table S9. Summary of study characteristics

Author	Year	Health outcome	Location	Study design	Sex	Age Start	Age End	Exposure Assessment	Outcome Assessment Method	Outcome type	Sample Size	Reference	Exposed group	Cases	Controls	Control Pool	Alcohol Type
Beulens	2000	Diabetes mellitus type 2	Italy	Prospective Cohort	Male	31.0	70.0	Self-report (human/environment)	Administrative medical records or disease registry	Morbidity	N/A	Light drinkers	Light drinkers	N/A	N/A	N/A	Any alcoholic beverages
Beulens	2000	Diabetes mellitus type 2	Italy	Prospective Cohort	Male	31.0	70.0	Self-report (human/environment)	Administrative medical records or disease registry	Morbidity	N/A	Light drinkers	Heavy drinkers	N/A	N/A	N/A	Any alcoholic beverages
Beulens	2000	Diabetes mellitus type 2	Netherlands	Prospective Cohort	Male	31.0	70.0	Self-report (human/environment)	Administrative medical records or disease registry	Morbidity	N/A	Light drinkers	Light drinkers	N/A	N/A	N/A	Any alcoholic beverages
Beulens	2000	Diabetes mellitus type 2	Netherlands	Prospective Cohort	Male	31.0	70.0	Self-report (human/environment)	Administrative medical records or disease registry	Morbidity	N/A	Light drinkers	Heavy drinkers	N/A	N/A	N/A	Any alcoholic beverages
Beulens	2000	Diabetes mellitus type 2	Spain	Prospective Cohort	Male	31.0	70.0	Self-report (human/environment)	Administrative medical records or disease registry	Morbidity	N/A	Light drinkers	Light drinkers	N/A	N/A	N/A	Any alcoholic beverages
Beulens	2000	Diabetes mellitus type 2	Spain	Prospective Cohort	Male	31.0	70.0	Self-report (human/environment)	Administrative medical records or disease registry	Morbidity	N/A	Light drinkers	Heavy drinkers	N/A	N/A	N/A	Any alcoholic beverages
Beulens	2000	Diabetes mellitus type 2	Sweden	Prospective Cohort	Male	31.0	70.0	Self-report (human/environment)	Administrative medical records or disease registry	Morbidity	N/A	Light drinkers	Light drinkers	N/A	N/A	N/A	Any alcoholic beverages
Beulens	2000	Diabetes mellitus type 2	Sweden	Prospective Cohort	Male	31.0	70.0	Self-report (human/environment)	Administrative medical records or disease registry	Morbidity	N/A	Light drinkers	Heavy drinkers	N/A	N/A	N/A	Any alcoholic beverages

Table S9. Summary of study characteristics

Author	Year	Health outcome	Location	Study design	Sex	Age Start	Age End	Exposure Assessment	Outcome Assessment Method	Outcome type	Sample Size	Reference	Exposed group	Cases	Controls	Control Pool	Alcohol Type
Beulens	2000	Diabetes mellitus type 2	United Kingdom	Prospective Cohort	Male	31.0	70.0	Self-report (human/environment)	Administrative medical records or disease registry	Morbidity	N/A	Light drinkers	Light drinkers	N/A	N/A	N/A	Any alcoholic beverages
Beulens	2000	Diabetes mellitus type 2	United Kingdom	Prospective Cohort	Male	31.0	70.0	Self-report (human/environment)	Administrative medical records or disease registry	Morbidity	N/A	Light drinkers	Heavy drinkers	N/A	N/A	N/A	Any alcoholic beverages
Beulens	2002	Diabetes mellitus type 2	Netherlands	Prospective Cohort	Female	49.0	70.0	Self-report (human/environment)	Self-report	Morbidity	16330	Non-drinkers	Light drinkers	N/A	N/A	N/A	Any alcoholic beverages
Choi	2008	Diabetes mellitus type 2	Republic of Korea	Prospective Cohort	Both	15.0	81.8	Self-report (human/environment)	Physician diagnosis	Morbidity	96129	Non-drinkers	Light drinkers	N/A	N/A	N/A	Any alcoholic beverages
Freisling	2007	Diabetes mellitus type 2	Global	Prospective Cohort	Both	17.6	81.8	Self-report (human/environment)	Administrative medical records or disease registry	Morbidity	291778	Light drinkers	Light drinkers	N/A	N/A	N/A	Any alcoholic beverages
He	2013	Diabetes mellitus type 2	United States of America	Prospective Cohort	Female	18.0	99.0	Self-report (human/environment)	Biomarker	Morbidity	6631	Light drinkers	Light drinkers	N/A	N/A	N/A	Any alcoholic beverages
He	2013	Diabetes mellitus type 2	United States of America	Prospective Cohort	Female	18.0	99.0	Self-report (human/environment)	Biomarker	Morbidity	6631	Light drinkers	Moderate drinkers	N/A	N/A	N/A	Any alcoholic beverages
He	2013	Diabetes mellitus type 2	United States of America	Prospective Cohort	Male	18.0	99.0	Self-report (human/environment)	Biomarker	Morbidity	5411	Light drinkers	Light drinkers	N/A	N/A	N/A	Any alcoholic beverages
Hinkle	2017	Diabetes mellitus type 2	United States of America	Prospective Cohort	Female	24.0	44.0	Self-report (human/environment)	Self-report	Morbidity	4740	Non-drinkers	Light drinkers	N/A	N/A	N/A	Any alcoholic beverages
Joosten	2006	Diabetes mellitus type 2	Netherlands	Prospective Cohort	Both	20.0	70.0	Self-report (human/environment)	Self-report	Morbidity	35625	Non-drinkers	Light drinkers	N/A	N/A	N/A	Any alcoholic beverages

Table S9. Summary of study characteristics

Author	Year	Health outcome	Location	Study design	Sex	Age Start	Age End	Exposure Assessment	Outcome Assessment Method	Outcome type	Sample Size	Reference	Exposed group	Cases	Controls	Control Pool	Alcohol Type
Kao	1998	Diabetes mellitus type 2	United States of America	Prospective Cohort	Female	45.0	64.0	Self-report (human/environment)	Biomarker	Morbidity	6838	Light drinkers	Light drinkers	N/A	N/A	N/A	Any alcoholic beverages
Kao	1998	Diabetes mellitus type 2	United States of America	Prospective Cohort	Female	45.0	64.0	Self-report (human/environment)	Biomarker	Morbidity	6838	Light drinkers	Moderate drinkers	N/A	N/A	N/A	Any alcoholic beverages
Kao	1998	Diabetes mellitus type 2	United States of America	Prospective Cohort	Male	45.0	64.0	Self-report (human/environment)	Biomarker	Morbidity	5423	Light drinkers	Moderate drinkers	N/A	N/A	N/A	Any alcoholic beverages
Li	2012	Diabetes mellitus type 2	China	Prospective Cohort	Both	18.0	99.0	Self-report (human/environment)	Biomarker	Morbidity	15601	Non-drinkers	Light drinkers	N/A	N/A	N/A	Any alcoholic beverages
Li	2015	Diabetes mellitus type 2	Japan	Prospective Cohort	Both	30.0	79.0	Self-report (human/environment)	Biomarker	Morbidity	5972	Non-drinkers	Heavy drinkers	N/A	N/A	N/A	Any alcoholic beverages
Lu	2008	Diabetes mellitus type 2	Global	Prospective Cohort	Both	45.0	69.0	Self-report (human/environment)	Biomarker	Morbidity	30882	Non-drinkers	Light drinkers	N/A	N/A	N/A	Any alcoholic beverages
Lv	2013	Diabetes mellitus type 2	China	Prospective Cohort	Both	30.0	79.0	Self-report (human/environment)	Administrative medical records or disease registry	Morbidity	461211	Non-drinkers	Light drinkers	N/A	N/A	N/A	Any alcoholic beverages
Maty	1999	Diabetes mellitus type 2	United States of America	Prospective Cohort	Both	15.0	99.0	N/A	N/A	Morbidity	N/A	Non-drinkers	Light drinkers	N/A	N/A	N/A	Any alcoholic beverages
Maty	1999	Diabetes mellitus type 2	United States of America	Prospective Cohort	Both	15.0	99.0	N/A	N/A	Morbidity	N/A	Light drinkers	Light drinkers	N/A	N/A	N/A	Any alcoholic beverages
Mejldal	2020	Diabetes mellitus type 2	Denmark	Prospective Cohort	Both	60.0	70.0	Self-report (human/environment)	Administrative medical records or disease registry	Morbidity	4000	Light drinkers	Heavy drinkers	N/A	N/A	N/A	Any alcoholic beverages
Mejldal	2020	Diabetes mellitus type 2	Denmark	Prospective Cohort	Both	25.0	64.0	Self-report (human/environment)	Biomarker	Morbidity	4000	Light drinkers	Heavy drinkers	N/A	N/A	N/A	Any alcoholic beverages

Table S9. Summary of study characteristics

Author	Year	Health outcome	Location	Study design	Sex	Age Start	Age End	Exposure Assessment	Outcome Assessment Method	Outcome type	Sample Size	Reference	Exposed group	Cases	Controls	Control Pool	Alcohol Type
Nagaya	2001	Diabetes mellitus type 2	Gifu	Prospective Cohort	Male	15.0	99.0	N/A	N/A	Morbidity	N/A	Non-drinkers	Moderate drinkers	N/A	N/A	N/A	Any alcoholic beverages
Nakanishi	2001	Diabetes mellitus type 2	Japan	Prospective Cohort	Male	35.0	59.0	Self-report (human/environment)	Biomarker	Morbidity	3018	Moderate drinkers	Light drinkers	N/A	N/A	N/A	Any alcoholic beverages
Okamura	2016	Diabetes mellitus type 2	Japan	Prospective Cohort	Male	17.3	71.9	Self-report (human/environment)	Biomarker	Morbidity	9948	Non-drinkers	Light drinkers	N/A	N/A	N/A	Any alcoholic beverages
Strodl	1999	Diabetes mellitus type 2	Australia	Prospective Cohort	Female	15.0	99.0	N/A	N/A	Morbidity	N/A	Non-drinkers	Light drinkers	N/A	N/A	N/A	Any alcoholic beverages
Waki	2000	Diabetes mellitus type 2	Japan	Prospective Cohort	Male	40.0	59.0	Self-report (human/environment)	Self-report	Morbidity	12913	Non-drinkers	Light drinkers	N/A	N/A	N/A	Any alcoholic beverages
Wändell	2004	Diabetes mellitus type 2	Sweden	Prospective Cohort	Male	60.0	61.0	Self-report (human/environment)	Self-report	Morbidity	1973	Non-drinkers	Light drinkers	N/A	N/A	N/A	Any alcoholic beverages
Wändell	2004	Diabetes mellitus type 2	Sweden	Prospective Cohort	Female	60.0	61.0	Self-report (human/environment)	Self-report	Morbidity	2133	Non-drinkers	Light drinkers	N/A	N/A	N/A	Any alcoholic beverages
Wändell	2004	Diabetes mellitus type 2	Sweden	Prospective Cohort	Female	60.0	61.0	Self-report (human/environment)	Self-report	Morbidity	2133	Non-drinkers	Moderate drinkers	N/A	N/A	N/A	Any alcoholic beverages
Almodova	2012	Esophageal cancer	Brazil	Case-control	Both	37.0	79.0	Self-report (human/environment)	Administrative medical records or disease registry	Morbidity	49	Non-drinkers	Light drinkers	N/A	N/A	Hospital Patients	Any alcoholic beverages
Chen	2006	Esophageal cancer	China	Case-control	Both	18.0	60.0	Self-report (human/environment)	Administrative medical records or disease registry	Morbidity	835	Non-drinkers	Heavy drinkers	N/A	N/A	From the community, neighborhood	Any alcoholic beverages
Choi	2016	Esophageal cancer	Republic of Korea	Prospective Cohort	Both	20.0	99.0	Self-report (human/environment)	Administrative medical records or	Morbidity	23179312	Non-drinkers	Light drinkers	N/A	N/A	N/A	Any alcoholic beverages

Table S9. Summary of study characteristics

Author	Year	Health outcome	Location	Study design	Sex	Age Start	Age End	Exposure Assessment	Outcome Assessment Method	Outcome type	Sample Size	Reference	Exposed group	Cases	Controls	Control Pool	Alcohol Type
									disease registry								
De Stefani	2004	Esophageal cancer	Uruguay	Case-control	Both	15.0	99.0	N/A	N/A	Morbidity	N/A	Non-drinkers	Moderate drinkers	118	674	N/A	Any alcoholic beverages
De Stefani	2004	Esophageal cancer	Uruguay	Case-control	Both	15.0	99.0	N/A	N/A	Morbidity	N/A	Non-drinkers	Heavy drinkers	111	510	N/A	Any alcoholic beverages
Freedman	2000	Esophageal cancer	United States of America	Prospective Cohort	Both	15.0	99.0	N/A	N/A	Morbidity	N/A	Light drinkers	Light drinkers	71	N/A	N/A	Any alcoholic beverages
Freedman	2000	Esophageal cancer	United States of America	Prospective Cohort	Both	15.0	99.0	N/A	N/A	Morbidity	N/A	Light drinkers	Light drinkers	188	N/A	N/A	Any alcoholic beverages
Freedman	2000	Esophageal cancer	United States of America	Prospective Cohort	Both	15.0	99.0	N/A	N/A	Morbidity	N/A	Light drinkers	Light drinkers	74	N/A	N/A	Any alcoholic beverages
Freedman	2000	Esophageal cancer	United States of America	Prospective Cohort	Both	15.0	99.0	N/A	N/A	Morbidity	N/A	Light drinkers	Light drinkers	190	N/A	N/A	Any alcoholic beverages
Freedman	2000	Esophageal cancer	United States of America	Prospective Cohort	Both	15.0	99.0	N/A	N/A	Morbidity	N/A	Light drinkers	Light drinkers	174	N/A	N/A	Any alcoholic beverages
Freedman	2000	Esophageal cancer	United States of America	Prospective Cohort	Both	15.0	99.0	N/A	N/A	Morbidity	N/A	Light drinkers	Heavy drinkers	174	N/A	N/A	Any alcoholic beverages
Freedman	2000	Esophageal cancer	United States of America	Prospective Cohort	Both	15.0	99.0	N/A	N/A	Morbidity	N/A	Light drinkers	Light drinkers	128	N/A	N/A	Any alcoholic beverages
Freedman	2000	Esophageal cancer	United States of America	Prospective Cohort	Both	15.0	99.0	N/A	N/A	Morbidity	N/A	Light drinkers	Heavy drinkers	128	N/A	N/A	Any alcoholic beverages
Gao	1993	Esophageal cancer	China	Case-control	Both	15.0	99.0	N/A	N/A	Morbidity	N/A	Non-drinkers	Light drinkers	247	652	N/A	Any alcoholic beverages
Garidou	1991	Esophageal cancer	Greece	Case-control	Both	15.0	99.0	N/A	N/A	Morbidity	N/A	Non-drinkers	Light drinkers	N/A	N/A	N/A	Any alcoholic beverages
Han	2019	Esophageal cancer	China	Prospective Cohort	Both	30.0	79.0	Self-report (human/environment)	Administrative medical records or disease registry	Morbidity	510145	Non-drinkers	Light drinkers	N/A	N/A	N/A	Any alcoholic beverages
Hashibe	2002	Esophageal cancer	Global	Case-control	Both	15.0	99.0	N/A	N/A	Morbidity	N/A	Non-drinkers	Light drinkers	16	641	N/A	Any alcoholic beverages

Table S9. Summary of study characteristics

Author	Year	Health outcome	Location	Study design	Sex	Age Start	Age End	Exposure Assessment	Outcome Assessment Method	Outcome type	Sample Size	Reference	Exposed group	Cases	Controls	Control Pool	Alcohol Type
Ishiguro	2004	Esophageal cancer	Japan	Prospective Cohort	Both	15.0	99.0	N/A	N/A	Morbidity	N/A	Non-drinkers	Light drinkers	65	N/A	N/A	Any alcoholic beverages
Jayaprakash	1998	Esophageal cancer	United States of America	Case-control	Both	15.0	99.0	N/A	N/A	Morbidity	N/A	Non-drinkers	Light drinkers	99	339	N/A	Any alcoholic beverages
Kabat	1990	Esophageal cancer	United States of America	Case-control	Both	15.0	99.0	N/A	N/A	Morbidity	N/A	Light drinkers	Heavy drinkers	N/A	N/A	N/A	Any alcoholic beverages
Kabat	1990	Esophageal cancer	United States of America	Case-control	Both	15.0	99.0	N/A	N/A	Morbidity	N/A	Light drinkers	Moderate drinkers	N/A	N/A	N/A	Any alcoholic beverages
Kimm	2006	Esophageal cancer	Republic of Korea	Prospective Cohort	Both	15.0	99.0	N/A	N/A	Morbidity	N/A	Non-drinkers	Heavy drinkers	297	N/A	N/A	Any alcoholic beverages
Laaksonen	2018	Esophageal cancer	Australia	Prospective Cohort	Both	18.0	99.0	Self-report (human/environment)	Administrative medical records or disease registry	Morbidity	365052	Non-drinkers	Light drinkers	N/A	N/A	N/A	Any alcoholic beverages
Lin	1997	Esophageal cancer	Sweden	Case-control	Both	18.0	80.0	Self-report (human/environment)	Physician diagnosis	Morbidity	806	Non-drinkers	Light drinkers	N/A	N/A	From the community, general population	Any alcoholic beverages
Pandeya	2005	Esophageal cancer	Australia	Case-control	Both	15.0	99.0	N/A	N/A	Morbidity	N/A	Non-drinkers	Light drinkers	N/A	N/A	N/A	Any alcoholic beverages
Steevens	2002	Esophageal cancer	Netherlands	Prospective Cohort	Both	15.0	99.0	N/A	N/A	Morbidity	N/A	Non-drinkers	Moderate drinkers	46	N/A	N/A	Any alcoholic beverages
Steevens	2002	Esophageal cancer	Netherlands	Prospective Cohort	Both	15.0	99.0	N/A	N/A	Morbidity	N/A	Non-drinkers	Light drinkers	55	N/A	N/A	Any alcoholic beverages
Steevens	2002	Esophageal cancer	Netherlands	Prospective Cohort	Both	15.0	99.0	N/A	N/A	Morbidity	N/A	Non-drinkers	Moderate drinkers	56	N/A	N/A	Any alcoholic beverages
Steevens	2002	Esophageal cancer	Netherlands	Prospective Cohort	Both	15.0	99.0	N/A	N/A	Morbidity	N/A	Non-drinkers	Light drinkers	31	N/A	N/A	Any alcoholic beverages
Wu	2007	Esophageal cancer	China	Case-control	Both	34.0	93.4	Self-report (human/environment)	Administrative medical records or disease registry	Morbidity	1079	Non-drinkers	Light drinkers	N/A	N/A	N/A	Any alcoholic beverages

Table S9. Summary of study characteristics

Author	Year	Health outcome	Location	Study design	Sex	Age Start	Age End	Exposure Assessment	Outcome Assessment Method	Outcome type	Sample Size	Reference	Exposed group	Cases	Controls	Control Pool	Alcohol Type
Yang	2004	Esophageal cancer	China	Case-control	Both	15.0	99.0	N/A	N/A	Morbidity	N/A	Non-drinkers	Light drinkers	104	150	N/A	Any alcoholic beverages
Yang	2005	Esophageal cancer	China	Prospective Cohort	Male	40.0	79.0	Self-report (human/environment)	Administrative medical records or disease registry	Morbidity	218189	Non-drinkers	Light drinkers	N/A	N/A	N/A	Any alcoholic beverages
Yi	2005	Esophageal cancer	Republic of Korea	Prospective Cohort	Both	45.1	88.0	Self-report (human/environment)	Administrative medical records or disease registry	Morbidity	N/A	Non-drinkers	Light drinkers	6	N/A	N/A	Any alcoholic beverages
Yi	2005	Esophageal cancer	Republic of Korea	Prospective Cohort	Both	45.1	88.0	Self-report (human/environment)	Administrative medical records or disease registry	Morbidity	6291	Non-drinkers	Light drinkers	N/A	N/A	N/A	Any alcoholic beverages
Yu	2015	Esophageal cancer	China	Prospective Cohort	Both	19.4	82.4	Self-report (human/environment)	Administrative medical records or disease registry	Morbidity	456155	Light drinkers	Light drinkers	N/A	N/A	N/A	Any alcoholic beverages
Zhao	2017	Esophageal cancer	China	Case-control	Both	30.0	79.0	Self-report (human/environment)	Administrative medical records or disease registry	Morbidity	212	Non-drinkers	Moderate drinkers	N/A	N/A	Hospital Patients	Any alcoholic beverages
Askgaard	2013	Haemorrhagic stroke	Denmark	Prospective Cohort	Both	20.0	49.0	Self-report (human/environment)	Physician diagnosis	Morbidity	10259	Light drinkers	Light drinkers	N/A	N/A	N/A	Any alcoholic beverages
Askgaard	2013	Haemorrhagic stroke	Denmark	Prospective Cohort	Both	20.0	49.0	Self-report (human/environment)	Physician diagnosis	Morbidity	10259	Light drinkers	Heavy drinkers	N/A	N/A	N/A	Any alcoholic beverages
Goldberg	1980	Haemorrhagic stroke	United States of America	Prospective Cohort	Male	15.0	99.0	N/A	N/A	Morbidity	N/A	Non-drinkers	Moderate drinkers	N/A	N/A	N/A	Any alcoholic beverages
Gordon	1972	Haemorrhagic stroke	United States of America	Prospective Cohort	Male	15.0	99.0	N/A	N/A	Morbidity	N/A	Non-drinkers	Moderate drinkers	N/A	N/A	N/A	Any alcoholic beverages

Table S9. Summary of study characteristics

Author	Year	Health outcome	Location	Study design	Sex	Age Start	Age End	Exposure Assessment	Outcome Assessment Method	Outcome type	Sample Size	Reference	Exposed group	Cases	Controls	Control Pool	Alcohol Type
Iso	2002	Haemorrhagic stroke	Japan	Prospective Cohort	Male	40.0	59.0	Self-report (human/environment)	Administrative medical records or disease registry	Morbidity	19544	Light drinkers	Moderate drinkers	N/A	N/A	N/A	Any alcoholic beverages
Kiyohara	1987	Haemorrhagic stroke	Japan	Prospective Cohort	Both	40.0	99.0	Self-report (human/environment)	Physician diagnosis	Morbidity	1621	Non-drinkers	Heavy drinkers	N/A	N/A	N/A	Any alcoholic beverages
Klatsky	1996	Haemorrhagic stroke	United States of America	Prospective Cohort	Both	15.0	99.0	N/A	N/A	Morbidity	N/A	Non-drinkers	Light drinkers	N/A	N/A	N/A	Any alcoholic beverages
Lu	2020	Haemorrhagic stroke	China	Prospective Cohort	Male	15.0	93.8	Self-report (human/environment)	Physician diagnosis	Morbidity	1736	Non-drinkers	Light drinkers	N/A	N/A	N/A	Any alcoholic beverages
Millwood	2017	Haemorrhagic stroke	China	Prospective Cohort	Female	19.0	85.0	Self-report (human/environment)	Administrative medical records or disease registry	Morbidity	302510	N/A	Heavy drinkers	N/A	N/A	N/A	Any alcoholic beverages
Sankai	1993	Haemorrhagic stroke	Japan	Prospective Cohort	Both	40.0	69.0	Self-report (human/environment)	Administrative medical records or disease registry	Morbidity	12372	Non-drinkers	Moderate drinkers	N/A	N/A	N/A	Any alcoholic beverages
Biddinger	2016	Ischemic heart disease	United Kingdom	Prospective Cohort	Both	33.2	80.8	Self-report (human/environment)	Administrative medical records or disease registry	Morbidity	371463	Non-drinkers	Light drinkers	N/A	N/A	N/A	Any alcoholic beverages
Biddinger	2016	Ischemic heart disease	United Kingdom	Prospective Cohort	Both	33.2	80.8	Self-report (human/environment)	Administrative medical records or disease registry	Morbidity	371463	Non-drinkers	Moderate drinkers	N/A	N/A	N/A	Any alcoholic beverages
Bos	2006	Ischemic heart disease	Netherlands	Prospective Cohort	Female	20.0	70.0	Self-report (human/environment)	Administrative medical records or disease registry	Morbidity	10530	Non-drinkers	Light drinkers	N/A	N/A	N/A	Any alcoholic beverages

Table S9. Summary of study characteristics

Author	Year	Health outcome	Location	Study design	Sex	Age Start	Age End	Exposure Assessment	Outcome Assessment Method	Outcome type	Sample Size	Reference	Exposed group	Cases	Controls	Control Pool	Alcohol Type
Britton	2007	Ischemic heart disease	United States of America	Prospective Cohort	Male	27.1	85.9	Self-report (human/environment)	Self-report	Morbidity	5164	Light drinkers	Light drinkers	N/A	N/A	N/A	Any alcoholic beverages
Britton	2007	Ischemic heart disease	United States of America	Prospective Cohort	Male	29.9	88.7	Self-report (human/environment)	Self-report	Morbidity	5164	Light drinkers	Light drinkers	N/A	N/A	N/A	Any alcoholic beverages
Britton	2007	Ischemic heart disease	United States of America	Prospective Cohort	Male	31.3	88.3	Self-report (human/environment)	Self-report	Morbidity	5164	Light drinkers	Light drinkers	N/A	N/A	N/A	Any alcoholic beverages
Bryson	2001	Ischemic heart disease	United States of America	Prospective Cohort	Both	65.0	99.0	Self-report (human/environment)	Self-report	Morbidity	5146	Non-drinkers	Moderate drinkers	N/A	N/A	N/A	Any alcoholic beverages
Camargo	1993	Ischemic heart disease	United States of America	Prospective Cohort	Male	15.0	99.0	N/A	N/A	Morbidity	N/A	Light drinkers	Light drinkers	N/A	N/A	N/A	Any alcoholic beverages
Cho	2017	Ischemic heart disease	Republic of Korea	Prospective Cohort	Both	23.0	80.6	Self-report (human/environment)	Administrative medical records or disease registry	Morbidity	11595191	Non-drinkers	Moderate drinkers	N/A	N/A	N/A	Any alcoholic beverages
Cho	2017	Ischemic heart disease	Republic of Korea	Prospective Cohort	Both	24.1	78.7	Self-report (human/environment)	Administrative medical records or disease registry	Morbidity	11595191	Non-drinkers	Moderate drinkers	N/A	N/A	N/A	Any alcoholic beverages
Cho	2017	Ischemic heart disease	Republic of Korea	Prospective Cohort	Both	23.7	80.7	Self-report (human/environment)	Administrative medical records or disease registry	Morbidity	11595191	Non-drinkers	Moderate drinkers	N/A	N/A	N/A	Any alcoholic beverages
Cullen	1989	Ischemic heart disease	Australia	Prospective Cohort	Both	40.0	99.0	Self-report (human/environment)	Administrative medical records or disease registry	Morbidity	2171	Non-drinkers	Light drinkers	N/A	N/A	N/A	Any alcoholic beverages
Dam	2012	Ischemic heart disease	Denmark	Prospective Cohort	Female	15.0	99.0	N/A	N/A	Morbidity	N/A	Light drinkers	Light drinkers	N/A	N/A	N/A	Any alcoholic beverages

Table S9. Summary of study characteristics

Author	Year	Health outcome	Location	Study design	Sex	Age Start	Age End	Exposure Assessment	Outcome Assessment Method	Outcome type	Sample Size	Reference	Exposed group	Cases	Controls	Control Pool	Alcohol Type
Friedman	1962	Ischemic heart disease	United States of America	Prospective Cohort	Female	15.0	99.0	N/A	N/A	Morbidity	N/A	Non-drinkers	Moderate drinkers	N/A	N/A	N/A	Any alcoholic beverages
Friedman	1962	Ischemic heart disease	United States of America	Prospective Cohort	Female	15.0	99.0	N/A	N/A	Morbidity	N/A	Non-drinkers	Heavy drinkers	N/A	N/A	N/A	Any alcoholic beverages
Fuchs	1992	Ischemic heart disease	United States of America	Prospective Cohort	Female	30.0	55.0	Self-report (human/environment)	Administrative medical records or disease registry	Morbidity	85709	Non-drinkers	Moderate drinkers	N/A	N/A	N/A	Any alcoholic beverages
Garg	1987	Ischemic heart disease	United States of America	Prospective Cohort	Female	45.0	74.0	Self-report (human/environment)	Administrative medical records or disease registry	Morbidity	3718	Non-drinkers	Moderate drinkers	N/A	N/A	N/A	Any alcoholic beverages
Han	2017	Ischemic heart disease	China	Prospective Cohort	Both	30.0	79.0	Self-report (human/environment)	Administrative medical records or disease registry	Morbidity	461047	Light drinkers	Moderate drinkers	N/A	N/A	N/A	Any alcoholic beverages
Hart	2003	Ischemic heart disease	United Kingdom	Prospective Cohort	Male	35.0	64.0	Self-report (human/environment)	Administrative medical records or disease registry	Morbidity	1016	Non-drinkers	Moderate drinkers	N/A	N/A	N/A	Any alcoholic beverages
Hein	1986	Ischemic heart disease	Denmark	Prospective Cohort	Male	53.0	74.0	Self-report (human/environment)	Administrative medical records or disease registry	Morbidity	2826	Non-drinkers	Heavy drinkers	N/A	N/A	N/A	Any alcoholic beverages
Hein	1986	Ischemic heart disease	Denmark	Prospective Cohort	Male	53.0	74.0	Self-report (human/environment)	Administrative medical records or disease registry	Morbidity	2826	Non-drinkers	Light drinkers	N/A	N/A	N/A	Any alcoholic beverages
Higashiyama	2007	Ischemic heart disease	Japan	Prospective Cohort	Male	30.0	79.0	Self-report (human/environment)	Administrative medical records or disease registry	Morbidity	1573	Non-drinkers	Light drinkers	N/A	N/A	N/A	Any alcoholic beverages

Table S9. Summary of study characteristics

Author	Year	Health outcome	Location	Study design	Sex	Age Start	Age End	Exposure Assessment	Outcome Assessment Method	Outcome type	Sample Size	Reference	Exposed group	Cases	Controls	Control Pool	Alcohol Type
Higashiyama	2007	Ischemic heart disease	Japan	Prospective Cohort	Male	30.0	79.0	Self-report (human/environment)	Administrative medical records or disease registry	Morbidity	1573	Non-drinkers	Heavy drinkers	N/A	N/A	N/A	Any alcoholic beverages
Hu	2015	Ischemic heart disease	China	Prospective Cohort	Male	15.0	86.8	Self-report (human/environment)	Self-report	Morbidity	40386	Non-drinkers	Heavy drinkers	N/A	N/A	N/A	Any alcoholic beverages
Hu	2015	Ischemic heart disease	China	Prospective Cohort	Male	19.2	84.6	Self-report (human/environment)	Self-report	Morbidity	40386	Non-drinkers	Heavy drinkers	N/A	N/A	N/A	Any alcoholic beverages
Hu	2015	Ischemic heart disease	China	Prospective Cohort	Male	20.2	83.8	Self-report (human/environment)	Self-report	Morbidity	40386	Non-drinkers	Heavy drinkers	N/A	N/A	N/A	Any alcoholic beverages
Jiang	2008	Ischemic heart disease	China	Case-control	Both	25.3	94.9	Self-report (human/environment)	N/A	Morbidity	4985	Non-drinkers	Light drinkers	N/A	N/A	N/A	Any alcoholic beverages
Kim	2016	Ischemic heart disease	Republic of Korea	Prospective Cohort	Both	20.0	99.0	Self-report (human/environment)	Administrative medical records or disease registry	Morbidity	375710	Non-drinkers	Light drinkers	N/A	N/A	N/A	Any alcoholic beverages
Kivela	1984	Ischemic heart disease	Finland	Prospective Cohort	Male	15.0	99.0	N/A	N/A	Morbidity	N/A	Non-drinkers	Light drinkers	N/A	N/A	N/A	Any alcoholic beverages
Klatsky	1991	Ischemic heart disease	United States of America	Prospective Cohort	Male	50.0	99.0	Self-report (human/environment)	Administrative medical records or disease registry	Morbidity	99574	Non-drinkers	Light drinkers	N/A	N/A	N/A	Any alcoholic beverages
Klatsky	1991	Ischemic heart disease	United States of America	Prospective Cohort	Male	50.0	99.0	Self-report (human/environment)	Administrative medical records or disease registry	Morbidity	99574	Non-drinkers	Heavy drinkers	N/A	N/A	N/A	Any alcoholic beverages
Leong	2003	Ischemic heart disease	Global	Case-control	Both	21.0	93.0	Self-report (human/environment)	Physician diagnosis	Morbidity	26778	Non-drinkers	Light drinkers	N/A	N/A	N/A	Any alcoholic beverages

Table S9. Summary of study characteristics

Author	Year	Health outcome	Location	Study design	Sex	Age Start	Age End	Exposure Assessment	Outcome Assessment Method	Outcome type	Sample Size	Reference	Exposed group	Cases	Controls	Control Pool	Alcohol Type
Malyutina	1998	Ischemic heart disease	Novosibirsk oblast	Prospective Cohort	Male	15.0	99.0	N/A	N/A	Morbidity	N/A	Non-drinkers	Moderate drinkers	N/A	N/A	N/A	Any alcoholic beverages
Mukamal	1998	Ischemic heart disease	United States of America	Prospective Cohort	Male	40.0	75.0	Self-report (human/environment)	Administrative medical records or disease registry	Morbidity	38077	Non-drinkers	Light drinkers	N/A	N/A	N/A	Any alcoholic beverages
Mukamal	2000	Ischemic heart disease	United States of America	Case-control	Female	30.0	55.0	Self-report (human/environment)	Self-report	Morbidity	733	Non-drinkers	Light drinkers	N/A	N/A	N/A	Any alcoholic beverages
Mukamal	2000	Ischemic heart disease	United States of America	Case-control	Female	40.0	75.0	Self-report (human/environment)	Self-report	Morbidity	733	Non-drinkers	Light drinkers	N/A	N/A	N/A	Any alcoholic beverages
Mukamal	2000	Ischemic heart disease	United States of America	Case-control	Male	30.0	55.0	Self-report (human/environment)	Self-report	Morbidity	798	Non-drinkers	Light drinkers	N/A	N/A	N/A	Any alcoholic beverages
Mukamal	2000	Ischemic heart disease	United States of America	Case-control	Male	40.0	75.0	Self-report (human/environment)	Self-report	Morbidity	798	Non-drinkers	Light drinkers	N/A	N/A	N/A	Any alcoholic beverages
Mukamal	2001	Ischemic heart disease	United States of America	Prospective Cohort	Both	65.0	99.0	Self-report (human/environment)	Death certificates	Morbidity	4410	Non-drinkers	Light drinkers	N/A	N/A	N/A	Any alcoholic beverages
Mukamal	2001	Ischemic heart disease	United States of America	Prospective Cohort	Both	65.0	99.0	Self-report (human/environment)	Death certificates	Morbidity	4410	Non-drinkers	Moderate drinkers	N/A	N/A	N/A	Any alcoholic beverages
Muraki	1990	Ischemic heart disease	Japan	Prospective Cohort	Male	40.0	79.0	Self-report (human/environment)	Death certificates	Morbidity	32004	Non-drinkers	Light drinkers	N/A	N/A	N/A	Any alcoholic beverages
Murray	1998	Ischemic heart disease	United States of America	Prospective Cohort	Male	35.0	60.0	Self-report (human/environment)	Administrative medical records or disease registry	Morbidity	3702	Non-drinkers	Moderate drinkers	N/A	N/A	N/A	Any alcoholic beverages
Murray	1999	Ischemic heart disease	Canada	Prospective Cohort	Female	18.0	64.0	Self-report (human/environment)	Physician diagnosis	Morbidity	535	Light drinkers	Light drinkers	N/A	N/A	N/A	Any alcoholic beverages

Table S9. Summary of study characteristics

Author	Year	Health outcome	Location	Study design	Sex	Age Start	Age End	Exposure Assessment	Outcome Assessment Method	Outcome type	Sample Size	Reference	Exposed group	Cases	Controls	Control Pool	Alcohol Type
Palmer	1993	Ischemic heart disease	United Kingdom	Prospective Cohort	Male	15.0	87.8	Self-report (human/environment)	Administrative medical records or disease registry	Morbidity	2091	Non-drinkers	Moderate drinkers	N/A	N/A	N/A	Any alcoholic beverages
Palmer	1993	Ischemic heart disease	United Kingdom	Prospective Cohort	Male	15.0	92.5	Self-report (human/environment)	Administrative medical records or disease registry	Morbidity	2091	Non-drinkers	Moderate drinkers	N/A	N/A	N/A	Any alcoholic beverages
Palmer	1993	Ischemic heart disease	United Kingdom	Prospective Cohort	Female	15.0	87.8	Self-report (human/environment)	Administrative medical records or disease registry	Morbidity	1970	Non-drinkers	Light drinkers	N/A	N/A	N/A	Any alcoholic beverages
Palmer	1993	Ischemic heart disease	United Kingdom	Prospective Cohort	Female	15.0	92.5	Self-report (human/environment)	Administrative medical records or disease registry	Morbidity	1970	Non-drinkers	Light drinkers	N/A	N/A	N/A	Any alcoholic beverages
Reddiess	2017	Ischemic heart disease	Switzerland	Prospective Cohort	Both	41.0	99.0	Self-report (human/environment)	Administrative medical records or disease registry	Morbidity	3852	Non-drinkers	Light drinkers	N/A	N/A	N/A	Any alcoholic beverages
Renaud	1996	Ischemic heart disease	France	Prospective Cohort	Male	40.0	60.0	Self-report (human/environment)	Death certificates	Morbidity	22093	Non-drinkers	Light drinkers	N/A	N/A	N/A	Wine
Renaud	1996	Ischemic heart disease	France	Prospective Cohort	Male	40.0	60.0	Self-report (human/environment)	Death certificates	Morbidity	22093	Non-drinkers	Light drinkers	N/A	N/A	N/A	Beer or cider
Renaud	1996	Ischemic heart disease	France	Prospective Cohort	Male	40.0	60.0	Self-report (human/environment)	Death certificates	Morbidity	22093	Non-drinkers	Heavy drinkers	N/A	N/A	N/A	Wine
Renaud	1996	Ischemic heart disease	France	Prospective Cohort	Male	40.0	60.0	Self-report (human/environment)	Death certificates	Morbidity	22093	Non-drinkers	Heavy drinkers	N/A	N/A	N/A	Beer or cider

Table S9. Summary of study characteristics

Author	Year	Health outcome	Location	Study design	Sex	Age Start	Age End	Exposure Assessment	Outcome Assessment Method	Outcome type	Sample Size	Reference	Exposed group	Cases	Controls	Control Pool	Alcohol Type
Ruidavets	2004	Ischemic heart disease	Northern Ireland	Prospective Cohort	Male	15.0	99.0	N/A	N/A	Morbidity	N/A	Light drinkers	Moderate drinkers	N/A	N/A	N/A	Any alcoholic beverages
Schooling	2003	Ischemic heart disease	Hong Kong Special Administrative Region of China	Prospective Cohort	Male	15.0	99.0	N/A	N/A	Morbidity	N/A	Non-drinkers	Light drinkers	N/A	N/A	N/A	Any alcoholic beverages
Schutte	2019	Ischemic heart disease	United Kingdom	Prospective Cohort	Both	40.0	69.0	Self-report (human/environment)	Administrative medical records or disease registry	Morbidity	354969	Non-drinkers	Moderate drinkers	N/A	N/A	N/A	Any alcoholic beverages
Shaper	1980	Ischemic heart disease	England	Prospective Cohort	Male	15.0	99.0	N/A	N/A	Morbidity	N/A	Light drinkers	Light drinkers	N/A	N/A	N/A	Any alcoholic beverages
Shaper	1980	Ischemic heart disease	Scotland	Prospective Cohort	Male	15.0	99.0	N/A	N/A	Morbidity	N/A	Light drinkers	Light drinkers	N/A	N/A	N/A	Any alcoholic beverages
Shaper	1980	Ischemic heart disease	Wales	Prospective Cohort	Male	15.0	99.0	N/A	N/A	Morbidity	N/A	Light drinkers	Light drinkers	N/A	N/A	N/A	Any alcoholic beverages
Smith	2017	Ischemic heart disease	United Kingdom	Prospective Cohort	Both	15.0	99.0	Self-report (human/environment)	Administrative medical records or disease registry	Morbidity	28967	Non-drinkers	Moderate drinkers	N/A	N/A	N/A	Any alcoholic beverages
Tolstrup	2004	Ischemic heart disease	Denmark	Case-cohort	Male	50.0	65.0	Self-report (human/environment)	Administrative medical records or disease registry	Morbidity	618	Light drinkers	Light drinkers	N/A	N/A	N/A	Any alcoholic beverages
Tolstrup	2004	Ischemic heart disease	Denmark	Case-cohort	Male	50.0	65.0	Self-report (human/environment)	Administrative medical records or disease registry	Morbidity	618	Light drinkers	Heavy drinkers	N/A	N/A	N/A	Any alcoholic beverages
Valmadrid	1996	Ischemic heart disease	United States of America	Prospective Cohort	Both	31.6	99.0	Self-report (human/environment)	Death certificates	Morbidity	983	Non-drinkers	Light drinkers	N/A	N/A	N/A	Any alcoholic beverages

Table S9. Summary of study characteristics

Author	Year	Health outcome	Location	Study design	Sex	Age Start	Age End	Exposure Assessment	Outcome Assessment Method	Outcome type	Sample Size	Reference	Exposed group	Cases	Controls	Control Pool	Alcohol Type
Valmadrid	1996	Ischemic heart disease	United States of America	Prospective Cohort	Both	32.8	98.8	Self-report (human/environment)	Death certificates	Morbidity	983	Non-drinkers	Light drinkers	N/A	N/A	N/A	Any alcoholic beverages
Valmadrid	1996	Ischemic heart disease	United States of America	Prospective Cohort	Both	34.6	93.4	Self-report (human/environment)	Death certificates	Morbidity	983	Non-drinkers	Light drinkers	N/A	N/A	N/A	Any alcoholic beverages
Vliegenthart	2003	Ischemic heart disease	Netherlands	Prospective Cohort	Both	55.0	85.0	Self-report (human/environment)	Physician diagnosis	Morbidity	2013	Non-drinkers	Light drinkers	N/A	N/A	N/A	Any alcoholic beverages
Walsh	2001	Ischemic heart disease	United States of America	Prospective Cohort	Female	28.0	62.0	Self-report (human/environment)	Administrative medical records or disease registry	Morbidity	3493	Non-drinkers	Light drinkers	N/A	N/A	N/A	Any alcoholic beverages
Wannamethee	2010	Ischemic heart disease	United Kingdom	Prospective Cohort	Male	40.0	59.0	Self-report (human/environment)	Administrative medical records or disease registry	Morbidity	3530	Light drinkers	Light drinkers	N/A	N/A	N/A	Any alcoholic beverages
Wellmann	1997	Ischemic heart disease	Germany	Prospective Cohort	Male	25.0	64.0	Self-report (human/environment)	Administrative medical records or disease registry	Morbidity	1294	Non-drinkers	Moderate drinkers	N/A	N/A	N/A	Any alcoholic beverages
Cho	2017	Ischemic stroke	Republic of Korea	Prospective Cohort	Both	18.0	99.0	Self-report (human/environment)	Administrative medical records or disease registry	Morbidity	11595191	Non-drinkers	Light drinkers	N/A	N/A	N/A	Any alcoholic beverages
Cho	2017	Ischemic stroke	Republic of Korea	Prospective Cohort	Both	18.0	99.0	Self-report (human/environment)	Administrative medical records or disease registry	Morbidity	11595191	Non-drinkers	Moderate drinkers	N/A	N/A	N/A	Any alcoholic beverages
Donahue	1977	Ischemic stroke	United States of America	Prospective Cohort	Male	15.0	99.0	N/A	N/A	Morbidity	N/A	Non-drinkers	Light drinkers	N/A	N/A	N/A	Any alcoholic beverages
Goldberg	1980	Ischemic stroke	United States of America	Prospective Cohort	Male	15.0	99.0	N/A	N/A	Morbidity	N/A	Non-drinkers	Moderate drinkers	N/A	N/A	N/A	Any alcoholic beverages

Table S9. Summary of study characteristics

Author	Year	Health outcome	Location	Study design	Sex	Age Start	Age End	Exposure Assessment	Outcome Assessment Method	Outcome type	Sample Size	Reference	Exposed group	Cases	Controls	Control Pool	Alcohol Type
Iso	2002	Ischemic stroke	Japan	Prospective Cohort	Male	40.0	59.0	Self-report (human/environment)	Administrative medical records or disease registry	Morbidity	19544	Light drinkers	Light drinkers	N/A	N/A	N/A	Any alcoholic beverages
Iso	2002	Ischemic stroke	Japan	Prospective Cohort	Male	40.0	59.0	Self-report (human/environment)	Administrative medical records or disease registry	Morbidity	19544	Light drinkers	Moderate drinkers	N/A	N/A	N/A	Any alcoholic beverages
Iso	2002	Ischemic stroke	Japan	Prospective Cohort	Male	40.0	59.0	Self-report (human/environment)	Administrative medical records or disease registry	Morbidity	19544	Light drinkers	Heavy drinkers	N/A	N/A	N/A	Any alcoholic beverages
Kim	2016	Ischemic stroke	Republic of Korea	Prospective Cohort	Both	15.0	89.8	Self-report (human/environment)	Administrative medical records or disease registry	Morbidity	375710	Non-drinkers	Light drinkers	N/A	N/A	N/A	Any alcoholic beverages
Kim	2016	Ischemic stroke	Republic of Korea	Prospective Cohort	Both	15.0	92.5	Self-report (human/environment)	Administrative medical records or disease registry	Morbidity	375710	Non-drinkers	Light drinkers	N/A	N/A	N/A	Any alcoholic beverages
Lu	2020	Ischemic stroke	China	Prospective Cohort	Male	15.0	93.8	Self-report (human/environment)	Physician diagnosis	Morbidity	1736	Non-drinkers	Moderate drinkers	N/A	N/A	N/A	Any alcoholic beverages
Millwood	2017	Ischemic stroke	China	Prospective Cohort	Male	19.0	85.0	Self-report (human/environment)	Administrative medical records or disease registry	Morbidity	210205	N/A	Heavy drinkers	N/A	N/A	N/A	Any alcoholic beverages
Millwood	2017	Ischemic stroke	China	Prospective Cohort	Female	19.0	85.0	Self-report (human/environment)	Administrative medical records or disease registry	Morbidity	302510	N/A	Heavy drinkers	N/A	N/A	N/A	Any alcoholic beverages

Table S9. Summary of study characteristics

Author	Year	Health outcome	Location	Study design	Sex	Age Start	Age End	Exposure Assessment	Outcome Assessment Method	Outcome type	Sample Size	Reference	Exposed group	Cases	Controls	Control Pool	Alcohol Type
Rist	2008	Ischemic stroke	United States of America	Prospective Cohort	Male	15.0	99.0	N/A	N/A	Morbidity	N/A	Light drinkers	Light drinkers	N/A	N/A	N/A	Any alcoholic beverages
Sacco	1997	Ischemic stroke	United States of America	Case-control	Both	31.0	99.0	Self-report (human/environment)	Physician diagnosis	Morbidity	1156	Moderate drinkers	Heavy drinkers	N/A	N/A	From the community, general population	Any alcoholic beverages
Yang	2013	Ischemic stroke	Republic of Korea	Prospective Cohort	Both	28.9	71.5	Self-report (human/environment)	Administrative medical records or disease registry	Morbidity	152469	Non-drinkers	Moderate drinkers	N/A	N/A	N/A	Any alcoholic beverages
Cho	2013	Larynx cancer	Republic of Korea	Prospective Cohort	Both	40.0	79.0	Self-report (human/environment)	Administrative medical records or disease registry	Morbidity	53093	Non-drinkers	Moderate drinkers	N/A	N/A	N/A	Any alcoholic beverages
Flanders	1979	Larynx cancer	United States of America	Case-control	Both	15.0	99.0	N/A	N/A	Morbidity	N/A	Light drinkers	Moderate drinkers	37	71	N/A	Any alcoholic beverages
Kawakita	2009	Larynx cancer	Global	Case-control	Both	18.0	99.0	Self-report (human/environment)	Administrative medical records or disease registry	Morbidity	12248	Non-drinkers	Light drinkers	N/A	N/A	Hospital Patients	Any alcoholic beverages
Lee	2018	Larynx cancer	Republic of Korea	Retrospective Cohort	Both	20.0	99.0	Self-report (human/environment)	Administrative medical records or disease registry	Morbidity	9957059	Non-drinkers	Light drinkers	N/A	N/A	N/A	Any alcoholic beverages
Lubin	2011	Larynx cancer	Global	Case-control	Female	18.0	99.0	Self-report (human/environment)	Administrative medical records or disease registry	Morbidity	2304	Light drinkers	Heavy drinkers	N/A	N/A	Hospital Patients	Any alcoholic beverages
Lubin	2011	Larynx cancer	Global	Case-control	Male	18.0	99.0	Self-report (human/environment)	Administrative medical records or disease registry	Morbidity	7552	Light drinkers	Heavy drinkers	N/A	N/A	Hospital Patients	Any alcoholic beverages

Table S9. Summary of study characteristics

Author	Year	Health outcome	Location	Study design	Sex	Age Start	Age End	Exposure Assessment	Outcome Assessment Method	Outcome type	Sample Size	Reference	Exposed group	Cases	Controls	Control Pool	Alcohol Type
Maasland	2003	Larynx cancer	Netherlands	Prospective Cohort	Both	15.0	99.0	N/A	N/A	Morbidity	N/A	Non-drinkers	Moderate drinkers	47	N/A	N/A	Any alcoholic beverages
Menvielle	1991	Larynx cancer	France	Case-control	Both	15.0	99.0	N/A	N/A	Morbidity	N/A	Light drinkers	Light drinkers	35	N/A	N/A	Any alcoholic beverages
Menvielle	1991	Larynx cancer	France	Case-control	Both	15.0	99.0	N/A	N/A	Morbidity	N/A	Light drinkers	Heavy drinkers	41	N/A	N/A	Any alcoholic beverages
Menvielle	1991	Larynx cancer	France	Case-control	Both	15.0	99.0	N/A	N/A	Morbidity	N/A	Light drinkers	Heavy drinkers	40	N/A	N/A	Any alcoholic beverages
Menvielle	2007	Larynx cancer	France	Case-control	Male	32.8	84.7	Self-report (human/environment)	Administrative medical records or disease registry	Morbidity	2686	Non-drinkers	Heavy drinkers	N/A	N/A	From the community, general population	Any alcoholic beverages
Menvielle	2007	Larynx cancer	France	Case-control	Male	32.8	84.7	Self-report (human/environment)	Administrative medical records or disease registry	Morbidity	2686	Non-drinkers	Moderate drinkers	N/A	N/A	From the community, general population	Any alcoholic beverages
Muscat	1990	Larynx cancer	United States of America	Case-control	Both	15.0	99.0	N/A	N/A	Morbidity	N/A	Light drinkers	Heavy drinkers	N/A	N/A	N/A	Any alcoholic beverages
Ramroth	2000	Larynx cancer	Global	Case-control	Both	15.0	99.0	N/A	N/A	Morbidity	N/A	Light drinkers	Moderate drinkers	122	532	N/A	Any alcoholic beverages
Ramroth	2000	Larynx cancer	Germany	Case-control	Both	18.0	80.0	Self-report (human/environment)	Administrative medical records or disease registry	Morbidity	769	Light drinkers	Heavy drinkers	N/A	N/A	N/A	Any alcoholic beverages
Santi	2000	Larynx cancer	Germany	Case-control	Male	15.0	99.0	Self-report (human/environment)	Administrative medical records or disease registry	Morbidity	702	Non-drinkers	Heavy drinkers	N/A	N/A	From the community, general population	Any alcoholic beverages
Zheng	1990	Larynx cancer	China	Case-control	Both	15.0	99.0	N/A	N/A	Morbidity	N/A	Non-drinkers	Moderate drinkers	102	179	N/A	Any alcoholic beverages

Table S9. Summary of study characteristics

Author	Year	Health outcome	Location	Study design	Sex	Age Start	Age End	Exposure Assessment	Outcome Assessment Method	Outcome type	Sample Size	Reference	Exposed group	Cases	Controls	Control Pool	Alcohol Type
Chen	2017	Lip and oral cavity cancer	China	Case-control	Male	20.0	80.0	Self-report (human/environment)	Administrative medical records or disease registry	Morbidity	1308	Non-drinkers	Light drinkers	N/A	N/A	Facility Visitors	Any alcoholic beverages
Chen	2017	Lip and oral cavity cancer	China	Case-control	Male	20.0	80.0	Self-report (human/environment)	Administrative medical records or disease registry	Morbidity	1308	Non-drinkers	Heavy drinkers	N/A	N/A	Facility Visitors	Any alcoholic beverages
Herrero	1999	Lip and oral cavity cancer	Global	Case-control	Both	15.0	99.0	N/A	N/A	Morbidity	N/A	Non-drinkers	Moderate drinkers	826	1195	N/A	Any alcoholic beverages
Huang	2016	Lip and oral cavity cancer	Taiwan	Case-control	Both	20.0	99.0	Self-report (human/environment)	Administrative medical records or disease registry	Morbidity	940	Non-drinkers	Light drinkers	N/A	N/A	Hospital Patients	Any alcoholic beverages
Radoï	2007	Lip and oral cavity cancer	France	Case-control	Both	18.0	75.0	Self-report (human/environment)	Administrative medical records or disease registry	Morbidity	3555	Non-drinkers	Light drinkers	N/A	N/A	From the community, general population	Any alcoholic beverages
Radoï	2007	Lip and oral cavity cancer	France	Case-control	Both	50.0	75.0	Self-report (human/environment)	Administrative medical records or disease registry	Morbidity	3481	Non-drinkers	Moderate drinkers	N/A	N/A	From the community, general population	Any alcoholic beverages
Radoï	2007	Lip and oral cavity cancer	France	Case-control	Both	18.0	70.0	Self-report (human/environment)	Administrative medical records or disease registry	Morbidity	3481	Non-drinkers	Moderate drinkers	N/A	N/A	From the community, general population	Any alcoholic beverages
Shanmugham	2006	Lip and oral cavity cancer	United States of America	Prospective Cohort	Female	30.0	55.0	Self-report (human/environment)	Self-report	Morbidity	87621	Non-drinkers	Light drinkers	N/A	N/A	N/A	Any alcoholic beverages
Werbrouck	2006	Lip and oral cavity cancer	Belgium	Case-control	Both	15.0	99.0	N/A	N/A	Morbidity	N/A	Non-drinkers	Light drinkers	10	76	N/A	Any alcoholic beverages

Table S9. Summary of study characteristics

Author	Year	Health outcome	Location	Study design	Sex	Age Start	Age End	Exposure Assessment	Outcome Assessment Method	Outcome type	Sample Size	Reference	Exposed group	Cases	Controls	Control Pool	Alcohol Type
Asim	2007	Liver cancer	India	Case-control	Both	18.0	99.0	Self-report (human/environment)	Physician diagnosis	Morbidity	525	Moderate drinkers	Heavy drinkers	N/A	N/A	Hospital Patients	Any alcoholic beverages
Bui	2018	Liver cancer	Republic of Korea	Prospective Cohort	Male	20.0	99.0	Self-report (human/environment)	Administrative medical records or disease registry	Morbidity	2839332	Non-drinkers	Light drinkers	N/A	N/A	N/A	Any alcoholic beverages
Bui	2018	Liver cancer	Republic of Korea	Prospective Cohort	Male	20.0	99.0	Self-report (human/environment)	Administrative medical records or disease registry	Morbidity	2839332	Non-drinkers	Moderate drinkers	N/A	N/A	N/A	Any alcoholic beverages
Bui	2018	Liver cancer	Republic of Korea	Prospective Cohort	Male	20.0	99.0	Self-report (human/environment)	Administrative medical records or disease registry	Morbidity	2839332	Non-drinkers	Heavy drinkers	N/A	N/A	N/A	Any alcoholic beverages
Cho	2013	Liver cancer	Republic of Korea	Prospective Cohort	Both	21.2	79.4	Self-report (human/environment)	Administrative medical records or disease registry	Morbidity	510217	Non-drinkers	Moderate drinkers	N/A	N/A	N/A	Any alcoholic beverages
Cococcia	2017	Liver cancer	United Kingdom	Retrospective Cohort	Both	15.0	95.9	Self-report (human/environment)	Physician diagnosis	Morbidity	109	Light drinkers	Moderate drinkers	N/A	N/A	N/A	Any alcoholic beverages
Floud	2001	Liver cancer	United Kingdom	Prospective Cohort	Female	41.9	70.7	Self-report (human/environment)	Administrative medical records or disease registry	Morbidity	795121	Light drinkers	Light drinkers	N/A	N/A	N/A	Any alcoholic beverages
Floud	2001	Liver cancer	United Kingdom	Prospective Cohort	Female	42.0	70.2	Self-report (human/environment)	Administrative medical records or disease registry	Morbidity	795121	Light drinkers	Light drinkers	N/A	N/A	N/A	Any alcoholic beverages
Floud	2001	Liver cancer	United Kingdom	Prospective Cohort	Female	42.0	69.6	Self-report (human/environment)	Administrative medical records or	Morbidity	795121	Light drinkers	Light drinkers	N/A	N/A	N/A	Any alcoholic beverages

Table S9. Summary of study characteristics

Author	Year	Health outcome	Location	Study design	Sex	Age Start	Age End	Exposure Assessment	Outcome Assessment Method	Outcome type	Sample Size	Reference	Exposed group	Cases	Controls	Control Pool	Alcohol Type
									disease registry								
Floud	2001	Liver cancer	United Kingdom	Prospective Cohort	Female	41.9	70.7	Self-report (human/environment)	Administrative medical records or disease registry	Morbidity	795121	Light drinkers	Moderate drinkers	N/A	N/A	N/A	Any alcoholic beverages
Floud	2001	Liver cancer	United Kingdom	Prospective Cohort	Female	42.0	70.2	Self-report (human/environment)	Administrative medical records or disease registry	Morbidity	795121	Light drinkers	Moderate drinkers	N/A	N/A	N/A	Any alcoholic beverages
Floud	2001	Liver cancer	United Kingdom	Prospective Cohort	Female	42.0	69.6	Self-report (human/environment)	Administrative medical records or disease registry	Morbidity	795121	Light drinkers	Moderate drinkers	N/A	N/A	N/A	Any alcoholic beverages
Goodman	1989	Liver cancer	Japan	Prospective Cohort	Male	18.0	99.0	Self-report (human/environment)	Administrative medical records or disease registry	Morbidity	36133	Non-drinkers	Light drinkers	N/A	N/A	N/A	Any alcoholic beverages
Jang	2008	Liver cancer	Republic of Korea	Case-control	Both	15.0	78.9	Self-report (human/environment)	Physician diagnosis	Morbidity	480	Non-drinkers	Heavy drinkers	N/A	N/A	Facility Visitors	Any alcoholic beverages
Jang	2008	Liver cancer	Republic of Korea	Case-control	Both	15.0	90.4	Self-report (human/environment)	Physician diagnosis	Morbidity	480	Non-drinkers	Heavy drinkers	N/A	N/A	Hospital Patients	Any alcoholic beverages
Jang	2008	Liver cancer	Republic of Korea	Case-control	Both	15.0	78.9	Self-report (human/environment)	Physician diagnosis	Morbidity	626	Non-drinkers	Heavy drinkers	N/A	N/A	Facility Visitors	Any alcoholic beverages
Jang	2008	Liver cancer	Republic of Korea	Case-control	Both	15.0	90.4	Self-report (human/environment)	Physician diagnosis	Morbidity	626	Non-drinkers	Heavy drinkers	N/A	N/A	Hospital Patients	Any alcoholic beverages
Jee	2002	Liver cancer	Republic of Korea	Prospective Cohort	Both	15.0	99.0	N/A	N/A	Morbidity	N/A	Non-drinkers	Moderate drinkers	1227	N/A	N/A	Any alcoholic beverages

Table S9. Summary of study characteristics

Author	Year	Health outcome	Location	Study design	Sex	Age Start	Age End	Exposure Assessment	Outcome Assessment Method	Outcome type	Sample Size	Reference	Exposed group	Cases	Controls	Control Pool	Alcohol Type
Jee	2002	Liver cancer	Republic of Korea	Prospective Cohort	Both	15.0	99.0	N/A	N/A	Morbidity	N/A	Non-drinkers	Light drinkers	1139	N/A	N/A	Any alcoholic beverages
Joshi	2004	Liver cancer	Republic of Korea	Prospective Cohort	Both	15.0	99.0	N/A	N/A	Morbidity	N/A	Non-drinkers	Heavy drinkers	N/A	N/A	N/A	Any alcoholic beverages
Joshi	2004	Liver cancer	Republic of Korea	Prospective Cohort	Both	15.0	99.0	N/A	N/A	Morbidity	N/A	Non-drinkers	Moderate drinkers	N/A	N/A	N/A	Any alcoholic beverages
Jung	2004	Liver cancer	Republic of Korea	Prospective Cohort	Both	20.0	99.0	Self-report (human/environment)	Death certificates	Morbidity	16320	Light drinkers	Heavy drinkers	N/A	N/A	N/A	Any alcoholic beverages
Koh	2007	Liver cancer	Singapore	Case-control	Both	15.0	99.0	N/A	N/A	Morbidity	N/A	Non-drinkers	Light drinkers	315	N/A	N/A	Any alcoholic beverages
Minami	2018	Liver cancer	Japan	Retrospective Cohort	Both	18.0	99.0	Self-report (human/environment)	Physician diagnosis	Morbidity	3101	Moderate drinkers	Heavy drinkers	N/A	N/A	N/A	Any alcoholic beverages
Ogimoto	1999	Liver cancer	Japan	Prospective Cohort	Male	40.0	59.0	Self-report (human/environment)	Death certificates	Morbidity	16715	Non-drinkers	Light drinkers	N/A	N/A	N/A	Any alcoholic beverages
Ogimoto	1999	Liver cancer	Japan	Prospective Cohort	Male	60.0	79.0	Self-report (human/environment)	Death certificates	Morbidity	16715	Non-drinkers	Light drinkers	N/A	N/A	N/A	Any alcoholic beverages
Ogimoto	1999	Liver cancer	Japan	Prospective Cohort	Male	40.0	59.0	Self-report (human/environment)	Death certificates	Morbidity	11628	Non-drinkers	Light drinkers	N/A	N/A	N/A	Any alcoholic beverages
Ogimoto	1999	Liver cancer	Japan	Prospective Cohort	Male	60.0	79.0	Self-report (human/environment)	Death certificates	Morbidity	11628	Non-drinkers	Light drinkers	N/A	N/A	N/A	Any alcoholic beverages
Ohishi	2002	Liver cancer	Japan	Case-control	Both	15.0	99.0	N/A	N/A	Morbidity	N/A	Non-drinkers	Light drinkers	N/A	N/A	N/A	Any alcoholic beverages
Ohishi	2002	Liver cancer	Japan	Case-control	Both	15.0	99.0	N/A	N/A	Morbidity	N/A	Non-drinkers	Moderate drinkers	N/A	N/A	N/A	Any alcoholic beverages
Ohishi	2002	Liver cancer	Japan	Case-control	Both	37.3	97.9	Self-report (human/environment)	Physician diagnosis	Morbidity	644	N/A	Light drinkers	N/A	N/A	From the community, general population	Any alcoholic beverages

Table S9. Summary of study characteristics

Author	Year	Health outcome	Location	Study design	Sex	Age Start	Age End	Exposure Assessment	Outcome Assessment Method	Outcome type	Sample Size	Reference	Exposed group	Cases	Controls	Control Pool	Alcohol Type
Ozasa	2005	Liver cancer	Japan	Prospective Cohort	Both	15.0	99.0	N/A	N/A	Morbidity	N/A	Non-drinkers	Heavy drinkers	142	N/A	N/A	Any alcoholic beverages
Persson	2006	Liver cancer	United States of America	Prospective Cohort	Both	50.0	71.0	Self-report (human/environment)	Administrative medical records or disease registry	Morbidity	492704	Light drinkers	Moderate drinkers	N/A	N/A	N/A	Any alcoholic beverages
Petrick	2010	Liver cancer	United States of America	Case-cohort	Both	30.0	99.0	Self-report (human/environment)	Administrative medical records or disease registry	Morbidity	1518331	Non-drinkers	Moderate drinkers	N/A	N/A	N/A	Any alcoholic beverages
Petrick	2010	Liver cancer	United States of America	Case-cohort	Both	30.0	99.0	Self-report (human/environment)	Administrative medical records or disease registry	Morbidity	1518331	Non-drinkers	Heavy drinkers	N/A	N/A	N/A	Any alcoholic beverages
Pyong	1992	Liver cancer	Japan	Case-control	Both	15.0	99.0	N/A	N/A	Morbidity	N/A	Non-drinkers	Heavy drinkers	20	107	N/A	Any alcoholic beverages
Pyong	1992	Liver cancer	Japan	Case-control	Both	15.0	99.0	N/A	N/A	Morbidity	N/A	Non-drinkers	Moderate drinkers	31	87	N/A	Any alcoholic beverages
Sarich	2013	Liver cancer	Australia	Prospective Cohort	Both	25.0	85.0	Self-report (human/environment)	Administrative medical records or disease registry	Morbidity	226162	Light drinkers	Light drinkers	N/A	N/A	N/A	Any alcoholic beverages
Sarich	2013	Liver cancer	Australia	Prospective Cohort	Both	45.0	99.0	Self-report (human/environment)	Administrative medical records or disease registry	Morbidity	226162	Light drinkers	Light drinkers	N/A	N/A	N/A	Any alcoholic beverages
Sarich	2013	Liver cancer	Australia	Prospective Cohort	Both	25.0	85.0	Self-report (human/environment)	Administrative medical records or disease registry	Morbidity	226162	Light drinkers	Moderate drinkers	N/A	N/A	N/A	Any alcoholic beverages

Table S9. Summary of study characteristics

Author	Year	Health outcome	Location	Study design	Sex	Age Start	Age End	Exposure Assessment	Outcome Assessment Method	Outcome type	Sample Size	Reference	Exposed group	Cases	Controls	Control Pool	Alcohol Type
Sarich	2013	Liver cancer	Australia	Prospective Cohort	Both	45.0	99.0	Self-report (human/environment)	Administrative medical records or disease registry	Morbidity	226162	Light drinkers	Moderate drinkers	N/A	N/A	N/A	Any alcoholic beverages
Schwartz	2009	Liver cancer	Finland	Randomized Control Trial	Male	50.0	69.0	Self-report (human/environment)	Administrative medical records or disease registry	Morbidity	27086	Light drinkers	Moderate drinkers	N/A	N/A	N/A	Any alcoholic beverages
Shimazu	2004	Liver cancer	Japan	Prospective Cohort	Male	40.0	79.0	Self-report (human/environment)	Administrative medical records or disease registry	Morbidity	89863	Light drinkers	Light drinkers	N/A	N/A	N/A	Any alcoholic beverages
Stemhagen	1980	Liver cancer	United States of America	Case-control	Both	15.0	99.0	N/A	N/A	Morbidity	N/A	Non-drinkers	Heavy drinkers	43	60	N/A	Any alcoholic beverages
Stemhagen	1980	Liver cancer	United States of America	Case-control	Both	15.0	99.0	N/A	N/A	Morbidity	N/A	Non-drinkers	Light drinkers	73	185	N/A	Any alcoholic beverages
Yi	2005	Liver cancer	Republic of Korea	Prospective Cohort	Both	15.0	99.0	N/A	N/A	Morbidity	N/A	Non-drinkers	Light drinkers	6	N/A	N/A	Any alcoholic beverages
Yi	2005	Liver cancer	Republic of Korea	Prospective Cohort	Both	15.0	99.0	N/A	N/A	Morbidity	N/A	Non-drinkers	Heavy drinkers	21	N/A	N/A	Any alcoholic beverages
Yi	2005	Liver cancer	Republic of Korea	Prospective Cohort	Both	15.0	99.0	N/A	N/A	Morbidity	N/A	Non-drinkers	Light drinkers	21	N/A	N/A	Any alcoholic beverages
Yi	2013	Liver cancer	Republic of Korea	Prospective Cohort	Both	23.9	82.1	Self-report (human/environment)	Administrative medical records or disease registry	Morbidity	492955	Non-drinkers	Light drinkers	N/A	N/A	N/A	Any alcoholic beverages
Yi	2013	Liver cancer	Republic of Korea	Prospective Cohort	Both	23.9	82.1	Self-report (human/environment)	Administrative medical records or disease registry	Morbidity	492955	Non-drinkers	Moderate drinkers	N/A	N/A	N/A	Any alcoholic beverages

Table S9. Summary of study characteristics

Author	Year	Health outcome	Location	Study design	Sex	Age Start	Age End	Exposure Assessment	Outcome Assessment Method	Outcome type	Sample Size	Reference	Exposed group	Cases	Controls	Control Pool	Alcohol Type
Yi	2013	Liver cancer	Republic of Korea	Prospective Cohort	Both	23.9	82.1	Self-report (human/environment)	Administrative medical records or disease registry	Morbidity	492955	Non-drinkers	Heavy drinkers	N/A	N/A	N/A	Any alcoholic beverages
Yuan	2001	Liver cancer	China	Prospective Cohort	Both	15.0	99.0	N/A	N/A	Morbidity	N/A	Non-drinkers	Moderate drinkers	192	655	N/A	Any alcoholic beverages
Zhang	1995	Liver cancer	China	Case-control	Both	15.0	99.0	N/A	N/A	Morbidity	N/A	Non-drinkers	Heavy drinkers	84	78	N/A	Any alcoholic beverages
Almirall	2000	Lower respiratory infections	Spain	Case-control	Male	15.0	99.0	Self-report (human/environment)	Administrative medical records or disease registry	Morbidity	696	Non-drinkers	Heavy drinkers	N/A	N/A	Facility Visitors	Any alcoholic beverages
Almirall	2000	Lower respiratory infections	Spain	Case-control	Female	15.0	99.0	Self-report (human/environment)	Administrative medical records or disease registry	Morbidity	630	Non-drinkers	Moderate drinkers	N/A	N/A	Facility Visitors	Any alcoholic beverages
Almirall	2000	Lower respiratory infections	Spain	Case-control	Male	15.0	99.0	Self-report (human/environment)	Administrative medical records or disease registry	Morbidity	696	Non-drinkers	Light drinkers	N/A	N/A	Facility Visitors	Any alcoholic beverages
Almirall	2000	Lower respiratory infections	Spain	Case-control	Female	15.0	99.0	Self-report (human/environment)	Administrative medical records or disease registry	Morbidity	630	Non-drinkers	Light drinkers	N/A	N/A	Facility Visitors	Any alcoholic beverages
Baik	1992	Lower respiratory infections	United States of America	Prospective Cohort	Male	40.0	75.0	Self-report (human/environment)	Self-report	Morbidity	26429	Non-drinkers	Light drinkers	N/A	N/A	N/A	Any alcoholic beverages
Baik	1992	Lower respiratory infections	United States of America	Prospective Cohort	Male	25.0	42.0	Self-report (human/environment)	Self-report	Morbidity	26429	Non-drinkers	Light drinkers	N/A	N/A	N/A	Any alcoholic beverages
Baik	1992	Lower respiratory infections	United States of America	Prospective Cohort	Female	40.0	75.0	Self-report (human/environment)	Self-report	Morbidity	78062	Non-drinkers	Light drinkers	N/A	N/A	N/A	Any alcoholic beverages

Table S9. Summary of study characteristics

Author	Year	Health outcome	Location	Study design	Sex	Age Start	Age End	Exposure Assessment	Outcome Assessment Method	Outcome type	Sample Size	Reference	Exposed group	Cases	Controls	Control Pool	Alcohol Type
Baik	1992	Lower respiratory infections	United States of America	Prospective Cohort	Female	25.0	42.0	Self-report (human/environment)	Self-report	Morbidity	78062	Non-drinkers	Light drinkers	N/A	N/A	N/A	Any alcoholic beverages
Baik	1992	Lower respiratory infections	United States of America	Prospective Cohort	Female	40.0	75.0	Self-report (human/environment)	Self-report	Morbidity	78062	Non-drinkers	Moderate drinkers	N/A	N/A	N/A	Any alcoholic beverages
Baik	1992	Lower respiratory infections	United States of America	Prospective Cohort	Female	25.0	42.0	Self-report (human/environment)	Self-report	Morbidity	78062	Non-drinkers	Moderate drinkers	N/A	N/A	N/A	Any alcoholic beverages
Conway	2014	Other pharynx cancer	Global	Case-control	Both	18.0	99.0	Self-report (human/environment)	Administrative medical records or disease registry	Morbidity	31954	Non-drinkers	Light drinkers	N/A	N/A	From the community, general population	Any alcoholic beverages
Lubin	2011	Other pharynx cancer	Global	Case-control	Male	18.0	99.0	Self-report (human/environment)	Administrative medical records or disease registry	Morbidity	7552	Light drinkers	Moderate drinkers	N/A	N/A	Hospital Patients	Any alcoholic beverages
Tota	2015	Other pharynx cancer	United States of America	Case-control	Both	30.0	69.0	Self-report (human/environment)	Administrative medical records or disease registry	Morbidity	9327	Light drinkers	Moderate drinkers	N/A	N/A	N/A	Any alcoholic beverages
Turati	2008	Other pharynx cancer	Italy	Case-control	Both	18.0	76.0	Self-report (human/environment)	Administrative medical records or disease registry	Morbidity	594	Light drinkers	Moderate drinkers	N/A	N/A	Hospital Patients	Any alcoholic beverages
Turati	2008	Other pharynx cancer	Italy	Case-control	Both	18.0	76.0	Self-report (human/environment)	Administrative medical records or disease registry	Morbidity	594	Light drinkers	Heavy drinkers	N/A	N/A	Hospital Patients	Any alcoholic beverages
Werbrouck	2006	Other pharynx cancer	Belgium	Case-control	Both	15.0	99.0	N/A	N/A	Morbidity	N/A	Non-drinkers	Moderate drinkers	24	84	N/A	Any alcoholic beverages

Table S9. Summary of study characteristics

Author	Year	Health outcome	Location	Study design	Sex	Age Start	Age End	Exposure Assessment	Outcome Assessment Method	Outcome type	Sample Size	Reference	Exposed group	Cases	Controls	Control Pool	Alcohol Type
Bui	2018	Pancreatic cancer	Republic of Korea	Prospective Cohort	Male	20.0	99.0	Self-report (human/environment)	Physician diagnosis	Morbidity	2839332	Non-drinkers	Light drinkers	N/A	N/A	N/A	Soju
Bui	2018	Pancreatic cancer	Republic of Korea	Prospective Cohort	Male	20.0	99.0	Self-report (human/environment)	Physician diagnosis	Morbidity	2839332	Non-drinkers	Moderate drinkers	N/A	N/A	N/A	Soju
Bui	2018	Pancreatic cancer	Republic of Korea	Prospective Cohort	Male	20.0	99.0	Self-report (human/environment)	Physician diagnosis	Morbidity	2839332	Non-drinkers	Heavy drinkers	N/A	N/A	N/A	Soju
Floud	2001	Pancreatic cancer	United Kingdom	Prospective Cohort	Female	41.9	70.7	Self-report (human/environment)	Administrative medical records or disease registry	Morbidity	795121	Light drinkers	Light drinkers	N/A	N/A	N/A	Any alcoholic beverages
Floud	2001	Pancreatic cancer	United Kingdom	Prospective Cohort	Female	42.0	70.2	Self-report (human/environment)	Administrative medical records or disease registry	Morbidity	795121	Light drinkers	Light drinkers	N/A	N/A	N/A	Any alcoholic beverages
Floud	2001	Pancreatic cancer	United Kingdom	Prospective Cohort	Female	42.0	69.6	Self-report (human/environment)	Administrative medical records or disease registry	Morbidity	795121	Light drinkers	Light drinkers	N/A	N/A	N/A	Any alcoholic beverages
Floud	2001	Pancreatic cancer	United Kingdom	Prospective Cohort	Female	41.9	70.7	Self-report (human/environment)	Administrative medical records or disease registry	Morbidity	795121	Light drinkers	Moderate drinkers	N/A	N/A	N/A	Any alcoholic beverages
Floud	2001	Pancreatic cancer	United Kingdom	Prospective Cohort	Female	42.0	70.2	Self-report (human/environment)	Administrative medical records or disease registry	Morbidity	795121	Light drinkers	Moderate drinkers	N/A	N/A	N/A	Any alcoholic beverages
Floud	2001	Pancreatic cancer	United Kingdom	Prospective Cohort	Female	42.0	69.6	Self-report (human/environment)	Administrative medical records or disease registry	Morbidity	795121	Light drinkers	Moderate drinkers	N/A	N/A	N/A	Any alcoholic beverages

Table S9. Summary of study characteristics

Author	Year	Health outcome	Location	Study design	Sex	Age Start	Age End	Exposure Assessment	Outcome Assessment Method	Outcome type	Sample Size	Reference	Exposed group	Cases	Controls	Control Pool	Alcohol Type
Floud	2001	Pancreatic cancer	United Kingdom	Prospective Cohort	Female	41.9	70.7	Self-report (human/environment)	Administrative medical records or disease registry	Morbidity	502872	Light drinkers	Light drinkers	N/A	N/A	N/A	Any alcoholic beverages
Floud	2001	Pancreatic cancer	United Kingdom	Prospective Cohort	Female	42.0	70.2	Self-report (human/environment)	Administrative medical records or disease registry	Morbidity	502872	Light drinkers	Light drinkers	N/A	N/A	N/A	Any alcoholic beverages
Floud	2001	Pancreatic cancer	United Kingdom	Prospective Cohort	Female	42.0	69.6	Self-report (human/environment)	Administrative medical records or disease registry	Morbidity	502872	Light drinkers	Light drinkers	N/A	N/A	N/A	Any alcoholic beverages
Floud	2001	Pancreatic cancer	United Kingdom	Prospective Cohort	Female	41.9	70.7	Self-report (human/environment)	Administrative medical records or disease registry	Morbidity	502872	Light drinkers	Moderate drinkers	N/A	N/A	N/A	Any alcoholic beverages
Floud	2001	Pancreatic cancer	United Kingdom	Prospective Cohort	Female	42.0	70.2	Self-report (human/environment)	Administrative medical records or disease registry	Morbidity	502872	Light drinkers	Moderate drinkers	N/A	N/A	N/A	Any alcoholic beverages
Floud	2001	Pancreatic cancer	United Kingdom	Prospective Cohort	Female	42.0	69.6	Self-report (human/environment)	Administrative medical records or disease registry	Morbidity	502872	Light drinkers	Moderate drinkers	N/A	N/A	N/A	Any alcoholic beverages
Jayasekara	2015	Pancreatic cancer	Australia	Prospective Cohort	Both	29.4	81.0	Self-report (human/environment)	Administrative medical records or disease registry	Morbidity	38472	Non-drinkers	Moderate drinkers	N/A	N/A	N/A	Any alcoholic beverages
Kono	1983	Pancreatic cancer	Japan	Prospective Cohort	Both	15.0	99.0	N/A	N/A	Morbidity	N/A	Non-drinkers	N/A	4	N/A	N/A	Any alcoholic beverages

Table S9. Summary of study characteristics

Author	Year	Health outcome	Location	Study design	Sex	Age Start	Age End	Exposure Assessment	Outcome Assessment Method	Outcome type	Sample Size	Reference	Exposed group	Cases	Controls	Control Pool	Alcohol Type
Koyanagi	2013	Pancreatic cancer	Japan	Case-control	Both	15.0	99.0	Self-report (human/environment)	Administrative medical records or disease registry	Morbidity	1456	Non-drinkers	Light drinkers	N/A	N/A	From the community, general population	Any alcoholic beverages
Koyanagi	2013	Pancreatic cancer	Japan	Case-control	Both	15.0	99.0	Self-report (human/environment)	Administrative medical records or disease registry	Morbidity	1882	Non-drinkers	Light drinkers	N/A	N/A	From the community, general population	Any alcoholic beverages
Naudin	2000	Pancreatic cancer	Global	Prospective Cohort	Female	22.0	82.0	Self-report (human/environment)	Administrative medical records or disease registry	Morbidity	333887	Light drinkers	Moderate drinkers	N/A	N/A	N/A	Any alcoholic beverages
Naudin	2000	Pancreatic cancer	Global	Prospective Cohort	Female	21.0	81.0	Self-report (human/environment)	Administrative medical records or disease registry	Morbidity	333887	Light drinkers	Moderate drinkers	N/A	N/A	N/A	Any alcoholic beverages
Naudin	2000	Pancreatic cancer	Global	Prospective Cohort	Female	22.0	82.0	Self-report (human/environment)	Administrative medical records or disease registry	Morbidity	142219	Light drinkers	Heavy drinkers	N/A	N/A	N/A	Any alcoholic beverages
Naudin	2000	Pancreatic cancer	Global	Prospective Cohort	Female	21.0	81.0	Self-report (human/environment)	Administrative medical records or disease registry	Morbidity	142219	Light drinkers	Heavy drinkers	N/A	N/A	N/A	Any alcoholic beverages
Okita	1995	Pancreatic cancer	Japan	Prospective Cohort	Male	15.0	99.0	Self-report (human/environment)	Administrative medical records or disease registry	Morbidity	45577	Non-drinkers	Light drinkers	N/A	N/A	N/A	Any alcoholic beverages
Okita	1995	Pancreatic cancer	Japan	Prospective Cohort	Female	15.0	99.0	Self-report (human/environment)	Administrative medical records or disease registry	Morbidity	45577	Non-drinkers	Light drinkers	N/A	N/A	N/A	Any alcoholic beverages

Table S9. Summary of study characteristics

Author	Year	Health outcome	Location	Study design	Sex	Age Start	Age End	Exposure Assessment	Outcome Assessment Method	Outcome type	Sample Size	Reference	Exposed group	Cases	Controls	Control Pool	Alcohol Type
Ozasa	2005	Pancreatic cancer	Japan	Prospective Cohort	Both	15.0	99.0	N/A	N/A	Morbidity	N/A	Non-drinkers	Heavy drinkers	139	N/A	N/A	Any alcoholic beverages
Ozasa	2005	Pancreatic cancer	Japan	Prospective Cohort	Both	15.0	99.0	N/A	N/A	Morbidity	N/A	Non-drinkers	Moderate drinkers	105	N/A	N/A	Any alcoholic beverages
Ozasa	2005	Pancreatic cancer	Japan	Prospective Cohort	Both	15.0	99.0	N/A	N/A	Morbidity	N/A	Non-drinkers	Heavy drinkers	87	N/A	N/A	Any alcoholic beverages
Pang	2008	Pancreatic cancer	China	Case-cohort	Male	19.4	83.6	Self-report (human/environment)	Administrative medical records or disease registry	Morbidity	209290	Light drinkers	Heavy drinkers	N/A	N/A	N/A	Any alcoholic beverages
Pang	2008	Pancreatic cancer	China	Case-cohort	Male	19.4	83.6	Self-report (human/environment)	Administrative medical records or disease registry	Morbidity	209290	Light drinkers	Light drinkers	N/A	N/A	N/A	Any alcoholic beverages
Park	2017	Pancreatic cancer	Republic of Korea	Prospective Cohort	Both	15.0	86.2	Self-report (human/environment)	Administrative medical records or disease registry	Morbidity	2956902	Non-drinkers	Light drinkers	N/A	N/A	N/A	Any alcoholic beverages
Park	2017	Pancreatic cancer	Republic of Korea	Prospective Cohort	Both	15.0	89.2	Self-report (human/environment)	Administrative medical records or disease registry	Morbidity	2956902	Non-drinkers	Light drinkers	N/A	N/A	N/A	Any alcoholic beverages
Park	2017	Pancreatic cancer	Republic of Korea	Prospective Cohort	Both	21.2	93.2	Self-report (human/environment)	Administrative medical records or disease registry	Morbidity	2956902	Non-drinkers	Light drinkers	N/A	N/A	N/A	Any alcoholic beverages
Park	2017	Pancreatic cancer	Republic of Korea	Prospective Cohort	Both	15.0	86.2	Self-report (human/environment)	Administrative medical records or disease registry	Morbidity	2956902	Non-drinkers	Moderate drinkers	N/A	N/A	N/A	Any alcoholic beverages

Table S9. Summary of study characteristics

Author	Year	Health outcome	Location	Study design	Sex	Age Start	Age End	Exposure Assessment	Outcome Assessment Method	Outcome type	Sample Size	Reference	Exposed group	Cases	Controls	Control Pool	Alcohol Type
Park	2017	Pancreatic cancer	Republic of Korea	Prospective Cohort	Both	15.0	89.2	Self-report (human/environment)	Administrative medical records or disease registry	Morbidity	2956902	Non-drinkers	Moderate drinkers	N/A	N/A	N/A	Any alcoholic beverages
Park	2017	Pancreatic cancer	Republic of Korea	Prospective Cohort	Both	21.2	93.2	Self-report (human/environment)	Administrative medical records or disease registry	Morbidity	2956902	Non-drinkers	Moderate drinkers	N/A	N/A	N/A	Any alcoholic beverages
Sarich	2013	Pancreatic cancer	Australia	Prospective Cohort	Both	25.0	85.0	Self-report (human/environment)	Administrative medical records or disease registry	Morbidity	226162	Light drinkers	Heavy drinkers	N/A	N/A	N/A	Any alcoholic beverages
Sarich	2013	Pancreatic cancer	Australia	Prospective Cohort	Both	28.6	98.2	Self-report (human/environment)	Administrative medical records or disease registry	Morbidity	226162	Light drinkers	Heavy drinkers	N/A	N/A	N/A	Any alcoholic beverages
Sarich	2013	Pancreatic cancer	Australia	Prospective Cohort	Both	29.1	94.5	Self-report (human/environment)	Administrative medical records or disease registry	Morbidity	226162	Light drinkers	Heavy drinkers	N/A	N/A	N/A	Any alcoholic beverages
Sarich	2013	Pancreatic cancer	Australia	Prospective Cohort	Both	30.3	91.5	Self-report (human/environment)	Administrative medical records or disease registry	Morbidity	226162	Light drinkers	Heavy drinkers	N/A	N/A	N/A	Any alcoholic beverages
Sarich	2013	Pancreatic cancer	Australia	Prospective Cohort	Both	31.3	90.1	Self-report (human/environment)	Administrative medical records or disease registry	Morbidity	226162	Light drinkers	Heavy drinkers	N/A	N/A	N/A	Any alcoholic beverages
Sarich	2013	Pancreatic cancer	Australia	Prospective Cohort	Both	32.7	87.3	Self-report (human/environment)	Administrative medical records or disease registry	Morbidity	226162	Light drinkers	Heavy drinkers	N/A	N/A	N/A	Any alcoholic beverages

Table S9. Summary of study characteristics

Author	Year	Health outcome	Location	Study design	Sex	Age Start	Age End	Exposure Assessment	Outcome Assessment Method	Outcome type	Sample Size	Reference	Exposed group	Cases	Controls	Control Pool	Alcohol Type
Sarich	2013	Pancreatic cancer	Australia	Prospective Cohort	Both	25.0	85.0	Self-report (human/environment)	Administrative medical records or disease registry	Morbidity	226162	Light drinkers	Light drinkers	N/A	N/A	N/A	Any alcoholic beverages
Sarich	2013	Pancreatic cancer	Australia	Prospective Cohort	Both	28.6	98.2	Self-report (human/environment)	Administrative medical records or disease registry	Morbidity	226162	Light drinkers	Light drinkers	N/A	N/A	N/A	Any alcoholic beverages
Sarich	2013	Pancreatic cancer	Australia	Prospective Cohort	Both	29.1	94.5	Self-report (human/environment)	Administrative medical records or disease registry	Morbidity	226162	Light drinkers	Light drinkers	N/A	N/A	N/A	Any alcoholic beverages
Sarich	2013	Pancreatic cancer	Australia	Prospective Cohort	Both	30.3	91.5	Self-report (human/environment)	Administrative medical records or disease registry	Morbidity	226162	Light drinkers	Light drinkers	N/A	N/A	N/A	Any alcoholic beverages
Sarich	2013	Pancreatic cancer	Australia	Prospective Cohort	Both	31.3	90.1	Self-report (human/environment)	Administrative medical records or disease registry	Morbidity	226162	Light drinkers	Light drinkers	N/A	N/A	N/A	Any alcoholic beverages
Sarich	2013	Pancreatic cancer	Australia	Prospective Cohort	Both	32.7	87.3	Self-report (human/environment)	Administrative medical records or disease registry	Morbidity	226162	Light drinkers	Light drinkers	N/A	N/A	N/A	Any alcoholic beverages
Yallew	2009	Pancreatic cancer	United States of America	Case-control	Both	15.0	99.0	Self-report (human/environment)	Administrative medical records or disease registry	Morbidity	983	Light drinkers	Heavy drinkers	N/A	N/A	From the community, general population	Any alcoholic beverages
Yallew	2009	Pancreatic cancer	United States of America	Case-control	Both	15.0	99.0	Self-report (human/environment)	Administrative medical records or disease registry	Morbidity	2708	Light drinkers	Heavy drinkers	N/A	N/A	From the community, general population	Any alcoholic beverages

Table S9. Summary of study characteristics

Author	Year	Health outcome	Location	Study design	Sex	Age Start	Age End	Exposure Assessment	Outcome Assessment Method	Outcome type	Sample Size	Reference	Exposed group	Cases	Controls	Control Pool	Alcohol Type
Yallew	2009	Pancreatic cancer	United States of America	Case-control	Both	15.0	99.0	Self-report (human/environment)	Administrative medical records or disease registry	Morbidity	983	Non-drinkers	Light drinkers	N/A	N/A	From the community, general population	Any alcoholic beverages
Yallew	2009	Pancreatic cancer	United States of America	Case-control	Both	15.0	99.0	Self-report (human/environment)	Administrative medical records or disease registry	Morbidity	2708	Non-drinkers	Light drinkers	N/A	N/A	From the community, general population	Any alcoholic beverages
Yallew	2009	Pancreatic cancer	United States of America	Case-control	Both	15.0	99.0	Self-report (human/environment)	Administrative medical records or disease registry	Morbidity	983	Light drinkers	Light drinkers	N/A	N/A	From the community, general population	Any alcoholic beverages
Yallew	2009	Pancreatic cancer	United States of America	Case-control	Both	15.0	99.0	Self-report (human/environment)	Administrative medical records or disease registry	Morbidity	2708	Light drinkers	Light drinkers	N/A	N/A	From the community, general population	Any alcoholic beverages
Kristiansen	2003	Pancreatitis	Denmark	Prospective Cohort	Both	15.0	99.0	N/A	N/A	Morbidity	N/A	Non-drinkers	Light drinkers	N/A	N/A	N/A	Any alcoholic beverages
Kristiansen	2003	Pancreatitis	Denmark	Prospective Cohort	Both	15.0	99.0	N/A	N/A	Morbidity	N/A	Non-drinkers	Moderate drinkers	N/A	N/A	N/A	Any alcoholic beverages
Kume	2010	Pancreatitis	Japan	Case-control	Both	15.0	99.0	Self-report (human/environment)	Physician diagnosis	Morbidity	1015	Non-drinkers	Light drinkers	N/A	N/A	Hospital Patients	Any alcoholic beverages
Prizment	2004	Pancreatitis	United States of America	Prospective Cohort	Female	55.0	69.0	Self-report (human/environment)	Administrative medical records or disease registry	Morbidity	36346	Light drinkers	Light drinkers	N/A	N/A	N/A	Any alcoholic beverages
Setiawan	2012	Pancreatitis	United States of America	Prospective Cohort	Male	45.0	75.0	Self-report (human/environment)	Administrative medical records or disease registry	Morbidity	65063	Non-drinkers	Light drinkers	N/A	N/A	N/A	Any alcoholic beverages

Table S9. Summary of study characteristics

Author	Year	Health outcome	Location	Study design	Sex	Age Start	Age End	Exposure Assessment	Outcome Assessment Method	Outcome type	Sample Size	Reference	Exposed group	Cases	Controls	Control Pool	Alcohol Type
Setiawan	2012	Pancreatitis	United States of America	Prospective Cohort	Male	45.0	75.0	Self-report (human/environment)	Administrative medical records or disease registry	Morbidity	65063	Non-drinkers	Heavy drinkers	N/A	N/A	N/A	Any alcoholic beverages
Setiawan	2012	Pancreatitis	United States of America	Prospective Cohort	Female	45.0	75.0	Self-report (human/environment)	Administrative medical records or disease registry	Morbidity	80823	Non-drinkers	Light drinkers	N/A	N/A	N/A	Any alcoholic beverages
Yang	2007	Pancreatitis	China	Retrospective Cohort	Both	43.9	83.5	Self-report (human/environment)	Self-report	Morbidity	23294	Non-drinkers	Light drinkers	N/A	N/A	N/A	Any alcoholic beverages
Albertsen	1994	Prostate cancer	Denmark	Prospective Cohort	Male	20.0	98.0	Self-report (human/environment)	Administrative medical records or disease registry	Morbidity	12989	Light drinkers	Moderate drinkers	N/A	N/A	N/A	Any alcoholic beverages
Albertsen	1994	Prostate cancer	Denmark	Prospective Cohort	Male	20.0	98.0	Self-report (human/environment)	Administrative medical records or disease registry	Morbidity	12989	Light drinkers	Moderate drinkers	N/A	N/A	N/A	Beer, normal strength
Albertsen	1994	Prostate cancer	Denmark	Prospective Cohort	Male	20.0	98.0	Self-report (human/environment)	Administrative medical records or disease registry	Morbidity	12989	Light drinkers	Moderate drinkers	N/A	N/A	N/A	Wine
Albertsen	1994	Prostate cancer	Denmark	Prospective Cohort	Male	20.0	98.0	Self-report (human/environment)	Administrative medical records or disease registry	Morbidity	12989	Light drinkers	Moderate drinkers	N/A	N/A	N/A	Spirits
Albertsen	1994	Prostate cancer	Denmark	Prospective Cohort	Male	20.0	98.0	Self-report (human/environment)	Administrative medical records or disease registry	Morbidity	12989	Light drinkers	Heavy drinkers	N/A	N/A	N/A	Any alcoholic beverages

Table S9. Summary of study characteristics

Author	Year	Health outcome	Location	Study design	Sex	Age Start	Age End	Exposure Assessment	Outcome Assessment Method	Outcome type	Sample Size	Reference	Exposed group	Cases	Controls	Control Pool	Alcohol Type
Albertsen	1994	Prostate cancer	Denmark	Prospective Cohort	Male	20.0	98.0	Self-report (human/environment)	Administrative medical records or disease registry	Morbidity	12989	Light drinkers	Heavy drinkers	N/A	N/A	N/A	Beer, normal strength
Albertsen	1994	Prostate cancer	Denmark	Prospective Cohort	Male	20.0	98.0	Self-report (human/environment)	Administrative medical records or disease registry	Morbidity	12989	Light drinkers	Heavy drinkers	N/A	N/A	N/A	Wine
Albertsen	1994	Prostate cancer	Denmark	Prospective Cohort	Male	20.0	98.0	Self-report (human/environment)	Administrative medical records or disease registry	Morbidity	12989	Light drinkers	Heavy drinkers	N/A	N/A	N/A	Spirits
Albertsen	1994	Prostate cancer	Denmark	Prospective Cohort	Male	20.0	98.0	Self-report (human/environment)	Administrative medical records or disease registry	Morbidity	12989	Light drinkers	Light drinkers	N/A	N/A	N/A	Any alcoholic beverages
Albertsen	1994	Prostate cancer	Denmark	Prospective Cohort	Male	20.0	98.0	Self-report (human/environment)	Administrative medical records or disease registry	Morbidity	12989	Light drinkers	Light drinkers	N/A	N/A	N/A	Beer, normal strength
Albertsen	1994	Prostate cancer	Denmark	Prospective Cohort	Male	20.0	98.0	Self-report (human/environment)	Administrative medical records or disease registry	Morbidity	12989	Light drinkers	Light drinkers	N/A	N/A	N/A	Wine
Albertsen	1994	Prostate cancer	Denmark	Prospective Cohort	Male	20.0	98.0	Self-report (human/environment)	Administrative medical records or disease registry	Morbidity	12989	Light drinkers	Light drinkers	N/A	N/A	N/A	Spirits
Albertsen	1994	Prostate cancer	Denmark	Prospective Cohort	Male	20.0	98.0	Self-report (human/environment)	Administrative medical records or disease registry	Morbidity	12989	Non-drinkers	Moderate drinkers	N/A	N/A	N/A	Any alcoholic beverages

Table S9. Summary of study characteristics

Author	Year	Health outcome	Location	Study design	Sex	Age Start	Age End	Exposure Assessment	Outcome Assessment Method	Outcome type	Sample Size	Reference	Exposed group	Cases	Controls	Control Pool	Alcohol Type
Albertsen	1994	Prostate cancer	Denmark	Prospective Cohort	Male	20.0	98.0	Self-report (human/environment)	Administrative medical records or disease registry	Morbidity	12989	Non-drinkers	Moderate drinkers	N/A	N/A	N/A	Beer, normal strength
Albertsen	1994	Prostate cancer	Denmark	Prospective Cohort	Male	20.0	98.0	Self-report (human/environment)	Administrative medical records or disease registry	Morbidity	12989	Non-drinkers	Moderate drinkers	N/A	N/A	N/A	Wine
Albertsen	1994	Prostate cancer	Denmark	Prospective Cohort	Male	20.0	98.0	Self-report (human/environment)	Administrative medical records or disease registry	Morbidity	12989	Non-drinkers	Moderate drinkers	N/A	N/A	N/A	Spirits
Albertsen	1994	Prostate cancer	Denmark	Prospective Cohort	Male	20.0	98.0	Self-report (human/environment)	Administrative medical records or disease registry	Morbidity	12989	Non-drinkers	Light drinkers	N/A	N/A	N/A	Any alcoholic beverages
Albertsen	1994	Prostate cancer	Denmark	Prospective Cohort	Male	20.0	98.0	Self-report (human/environment)	Administrative medical records or disease registry	Morbidity	12989	Non-drinkers	Light drinkers	N/A	N/A	N/A	Beer, normal strength
Albertsen	1994	Prostate cancer	Denmark	Prospective Cohort	Male	20.0	98.0	Self-report (human/environment)	Administrative medical records or disease registry	Morbidity	12989	Non-drinkers	Light drinkers	N/A	N/A	N/A	Wine
Albertsen	1994	Prostate cancer	Denmark	Prospective Cohort	Male	20.0	98.0	Self-report (human/environment)	Administrative medical records or disease registry	Morbidity	12989	Non-drinkers	Light drinkers	N/A	N/A	N/A	Spirits
Andersson	1991	Prostate cancer	Sweden	Case-control	Male	51.7	88.3	Self-report (human/environment)	Administrative medical records or disease registry	Morbidity	252	Non-drinkers	Light drinkers	N/A	N/A	From the community, general population	Any alcoholic beverages

Table S9. Summary of study characteristics

Author	Year	Health outcome	Location	Study design	Sex	Age Start	Age End	Exposure Assessment	Outcome Assessment Method	Outcome type	Sample Size	Reference	Exposed group	Cases	Controls	Control Pool	Alcohol Type
Baglietto	1994	Prostate cancer	Australia	Prospective Cohort	Male	27.0	70.0	Self-report (human/environment)	Administrative medical records or disease registry	Morbidity	16872	Non-drinkers	Light drinkers	N/A	N/A	N/A	Any alcoholic beverages
Baglietto	1994	Prostate cancer	Australia	Prospective Cohort	Male	27.0	70.0	Self-report (human/environment)	Administrative medical records or disease registry	Morbidity	16872	Non-drinkers	Moderate drinkers	N/A	N/A	N/A	Any alcoholic beverages
Baglietto	1994	Prostate cancer	Australia	Prospective Cohort	Male	27.0	70.0	Self-report (human/environment)	Administrative medical records or disease registry	Morbidity	16872	Non-drinkers	Heavy drinkers	N/A	N/A	N/A	Any alcoholic beverages
Breslow	1992	Prostate cancer	United States of America	Prospective Cohort	Male	25.0	75.0	Self-report (human/environment)	Administrative medical records or disease registry	Morbidity	5766	Non-drinkers	Light drinkers	N/A	N/A	N/A	Any alcoholic beverages
Breslow	1992	Prostate cancer	United States of America	Prospective Cohort	Male	25.0	75.0	Self-report (human/environment)	Administrative medical records or disease registry	Morbidity	5766	Non-drinkers	Moderate drinkers	N/A	N/A	N/A	Any alcoholic beverages
Chang	2002	Prostate cancer	Sweden	Case-control	Male	44.5	88.3	Self-report (human/environment)	Administrative medical records or disease registry	Morbidity	1130	Non-drinkers	Light drinkers	N/A	N/A	From the community, general population	Any alcoholic beverages
Chang	2002	Prostate cancer	Sweden	Case-control	Male	44.5	88.3	Self-report (human/environment)	Administrative medical records or disease registry	Morbidity	1130	Non-drinkers	Moderate drinkers	N/A	N/A	From the community, general population	Any alcoholic beverages
Chao	2007	Prostate cancer	United States of America	Prospective Cohort	Male	45.0	69.0	Self-report (human/environment)	Administrative medical records or disease registry	Morbidity	65972	Non-drinkers	Light drinkers	N/A	N/A	N/A	Red wine

Table S9. Summary of study characteristics

Author	Year	Health outcome	Location	Study design	Sex	Age Start	Age End	Exposure Assessment	Outcome Assessment Method	Outcome type	Sample Size	Reference	Exposed group	Cases	Controls	Control Pool	Alcohol Type
Chao	2007	Prostate cancer	United States of America	Prospective Cohort	Male	45.0	69.0	Self-report (human/environment)	Administrative medical records or disease registry	Morbidity	65972	Non-drinkers	Light drinkers	N/A	N/A	N/A	Beer or cider
Chao	2007	Prostate cancer	United States of America	Prospective Cohort	Male	45.0	69.0	Self-report (human/environment)	Administrative medical records or disease registry	Morbidity	65972	Non-drinkers	Light drinkers	N/A	N/A	N/A	White wine
Chao	2007	Prostate cancer	United States of America	Prospective Cohort	Male	45.0	69.0	Self-report (human/environment)	Administrative medical records or disease registry	Morbidity	65972	Non-drinkers	Light drinkers	N/A	N/A	N/A	Hard liquor
Hayes	1989	Prostate cancer	United States of America	Case-control	Male	40.0	79.0	Self-report (human/environment)	Administrative medical records or disease registry	Morbidity	1315	Non-drinkers	Heavy drinkers	N/A	N/A	From the community, general population	Any alcoholic beverages
Hayes	1989	Prostate cancer	United States of America	Case-control	Male	40.0	79.0	Self-report (human/environment)	Administrative medical records or disease registry	Morbidity	1315	Non-drinkers	Heavy drinkers	N/A	N/A	From the community, general population	Beer, normal strength
Hayes	1989	Prostate cancer	United States of America	Case-control	Male	40.0	79.0	Self-report (human/environment)	Administrative medical records or disease registry	Morbidity	1315	Non-drinkers	Heavy drinkers	N/A	N/A	From the community, general population	Spirits
Hayes	1989	Prostate cancer	United States of America	Case-control	Male	40.0	79.0	Self-report (human/environment)	Administrative medical records or disease registry	Morbidity	1315	Non-drinkers	Heavy drinkers	N/A	N/A	From the community, general population	Wine
Hayes	1989	Prostate cancer	United States of America	Case-control	Male	40.0	79.0	Self-report (human/environment)	Administrative medical records or disease registry	Morbidity	1315	Non-drinkers	Light drinkers	N/A	N/A	From the community, general population	Any alcoholic beverages

Table S9. Summary of study characteristics

Author	Year	Health outcome	Location	Study design	Sex	Age Start	Age End	Exposure Assessment	Outcome Assessment Method	Outcome type	Sample Size	Reference	Exposed group	Cases	Controls	Control Pool	Alcohol Type
Hayes	1989	Prostate cancer	United States of America	Case-control	Male	40.0	79.0	Self-report (human/environment)	Administrative medical records or disease registry	Morbidity	1315	Non-drinkers	Light drinkers	N/A	N/A	From the community, general population	Beer, normal strength
Hayes	1989	Prostate cancer	United States of America	Case-control	Male	40.0	79.0	Self-report (human/environment)	Administrative medical records or disease registry	Morbidity	1315	Non-drinkers	Light drinkers	N/A	N/A	From the community, general population	Spirits
Hayes	1989	Prostate cancer	United States of America	Case-control	Male	40.0	79.0	Self-report (human/environment)	Administrative medical records or disease registry	Morbidity	1315	Non-drinkers	Light drinkers	N/A	N/A	From the community, general population	Wine
Hayes	1989	Prostate cancer	United States of America	Case-control	Male	40.0	79.0	Self-report (human/environment)	Administrative medical records or disease registry	Morbidity	1315	Non-drinkers	Moderate drinkers	N/A	N/A	From the community, general population	Any alcoholic beverages
Hayes	1989	Prostate cancer	United States of America	Case-control	Male	40.0	79.0	Self-report (human/environment)	Administrative medical records or disease registry	Morbidity	1315	Non-drinkers	Moderate drinkers	N/A	N/A	From the community, general population	Beer, normal strength
Hayes	1989	Prostate cancer	United States of America	Case-control	Male	40.0	79.0	Self-report (human/environment)	Administrative medical records or disease registry	Morbidity	1315	Non-drinkers	Moderate drinkers	N/A	N/A	From the community, general population	Spirits
Hayes	1989	Prostate cancer	United States of America	Case-control	Male	40.0	79.0	Self-report (human/environment)	Administrative medical records or disease registry	Morbidity	1315	Non-drinkers	Moderate drinkers	N/A	N/A	From the community, general population	Wine
Hiatt	1985	Prostate cancer	United States of America	Prospective Cohort	Male	39.0	87.0	Self-report (human/environment)	Administrative medical records or disease registry	Morbidity	43432	Non-drinkers	Light drinkers	N/A	N/A	N/A	Any alcoholic beverages

Table S9. Summary of study characteristics

Author	Year	Health outcome	Location	Study design	Sex	Age Start	Age End	Exposure Assessment	Outcome Assessment Method	Outcome type	Sample Size	Reference	Exposed group	Cases	Controls	Control Pool	Alcohol Type
Hiatt	1985	Prostate cancer	United States of America	Prospective Cohort	Male	39.0	87.0	Self-report (human/environment)	Administrative medical records or disease registry	Morbidity	43432	Non-drinkers	Heavy drinkers	N/A	N/A	N/A	Any alcoholic beverages
Lumey	1991	Prostate cancer	United States of America	Case-control	Male	42.8	82.4	Self-report (human/environment)	Administrative medical records or disease registry	Morbidity	2041	Non-drinkers	Moderate drinkers	N/A	N/A	Hospital Patients	Any alcoholic beverages
Lumey	1991	Prostate cancer	United States of America	Case-control	Male	42.8	82.4	Self-report (human/environment)	Administrative medical records or disease registry	Morbidity	2041	Non-drinkers	Heavy drinkers	N/A	N/A	Hospital Patients	Any alcoholic beverages
Olmedo-Requena	2019	Prostate cancer	Spain	Case-control	Male	40.0	80.0	Self-report (human/environment)	Administrative medical records or disease registry	Morbidity	302	Non-drinkers	Light drinkers	N/A	N/A	From the community, general population	Any alcoholic beverages
Rohrmann	2000	Prostate cancer	Global	Prospective Cohort	Male	54.1	78.7	Self-report (human/environment)	Administrative medical records or disease registry	Morbidity	142607	Light drinkers	Light drinkers	N/A	N/A	N/A	Any alcoholic beverages
Rohrmann	2000	Prostate cancer	Global	Prospective Cohort	Male	54.1	78.7	Self-report (human/environment)	Administrative medical records or disease registry	Morbidity	142607	Light drinkers	Heavy drinkers	N/A	N/A	N/A	Any alcoholic beverages
Rohrmann	2000	Prostate cancer	Global	Prospective Cohort	Male	54.1	78.7	Self-report (human/environment)	Administrative medical records or disease registry	Morbidity	142607	Light drinkers	Moderate drinkers	N/A	N/A	N/A	Any alcoholic beverages
Sarich	2013	Prostate cancer	Australia	Prospective Cohort	Both	25.0	85.0	Self-report (human/environment)	Administrative medical records or disease registry	Morbidity	226162	Light drinkers	Light drinkers	N/A	N/A	N/A	Any alcoholic beverages

Table S9. Summary of study characteristics

Author	Year	Health outcome	Location	Study design	Sex	Age Start	Age End	Exposure Assessment	Outcome Assessment Method	Outcome type	Sample Size	Reference	Exposed group	Cases	Controls	Control Pool	Alcohol Type
Schoonen	1996	Prostate cancer	United States of America	Case-control	Male	40.0	64.0	Self-report (human/environment)	Administrative medical records or disease registry	Morbidity	703	Light drinkers	Light drinkers	N/A	N/A	From the community, neighborhood	Any alcoholic beverages
Schoonen	1996	Prostate cancer	United States of America	Case-control	Male	40.0	64.0	Self-report (human/environment)	Administrative medical records or disease registry	Morbidity	703	Light drinkers	Light drinkers	N/A	N/A	From the community, neighborhood	Wine
Schoonen	1996	Prostate cancer	United States of America	Case-control	Male	40.0	64.0	Self-report (human/environment)	Administrative medical records or disease registry	Morbidity	703	Light drinkers	Light drinkers	N/A	N/A	From the community, neighborhood	Spirits
Schoonen	1996	Prostate cancer	United States of America	Case-control	Male	40.0	64.0	Self-report (human/environment)	Administrative medical records or disease registry	Morbidity	703	Light drinkers	Light drinkers	N/A	N/A	From the community, neighborhood	Beer or cider
Schuurman	1992	Prostate cancer	Netherlands	Case-cohort	Male	50.0	69.0	Self-report (human/environment)	Administrative medical records or disease registry	Morbidity	1591	Non-drinkers	Moderate drinkers	N/A	N/A	N/A	Any alcoholic beverages
Schuurman	1992	Prostate cancer	Netherlands	Case-cohort	Male	50.0	69.0	Self-report (human/environment)	Administrative medical records or disease registry	Morbidity	1591	Non-drinkers	Moderate drinkers	N/A	N/A	N/A	Beer, normal strength
Schuurman	1992	Prostate cancer	Netherlands	Case-cohort	Male	50.0	69.0	Self-report (human/environment)	Administrative medical records or disease registry	Morbidity	1591	Non-drinkers	Moderate drinkers	N/A	N/A	N/A	Wine
Schuurman	1992	Prostate cancer	Netherlands	Case-cohort	Male	50.0	69.0	Self-report (human/environment)	Administrative medical records or disease registry	Morbidity	1591	Non-drinkers	Moderate drinkers	N/A	N/A	N/A	Spirits

Table S9. Summary of study characteristics

Author	Year	Health outcome	Location	Study design	Sex	Age Start	Age End	Exposure Assessment	Outcome Assessment Method	Outcome type	Sample Size	Reference	Exposed group	Cases	Controls	Control Pool	Alcohol Type
Schuurman	1992	Prostate cancer	Netherlands	Case-cohort	Male	50.0	69.0	Self-report (human/environment)	Administrative medical records or disease registry	Morbidity	1591	Non-drinkers	Light drinkers	N/A	N/A	N/A	Any alcoholic beverages
Schuurman	1992	Prostate cancer	Netherlands	Case-cohort	Male	50.0	69.0	Self-report (human/environment)	Administrative medical records or disease registry	Morbidity	1591	Non-drinkers	Light drinkers	N/A	N/A	N/A	Beer, normal strength
Schuurman	1992	Prostate cancer	Netherlands	Case-cohort	Male	50.0	69.0	Self-report (human/environment)	Administrative medical records or disease registry	Morbidity	1591	Non-drinkers	Light drinkers	N/A	N/A	N/A	Wine
Schuurman	1992	Prostate cancer	Netherlands	Case-cohort	Male	50.0	69.0	Self-report (human/environment)	Administrative medical records or disease registry	Morbidity	1591	Non-drinkers	Light drinkers	N/A	N/A	N/A	Spirits
Sutcliffe	2002	Prostate cancer	United States of America	Prospective Cohort	Male	40.0	75.0	Self-report (human/environment)	Self-report	Morbidity	51529	Non-drinkers	Light drinkers	N/A	N/A	N/A	Any alcoholic beverages
Velicer	2002	Prostate cancer	United States of America	Prospective Cohort	Male	50.0	76.0	Self-report (human/environment)	Administrative medical records or disease registry	Morbidity	34565	Light drinkers	Moderate drinkers	N/A	N/A	N/A	Any alcoholic beverages
Wang	2004	Prostate cancer	Global	Case-control	Both	15.0	99.0	N/A	N/A	Morbidity	N/A	Non-drinkers	Light drinkers	124	682	N/A	Any alcoholic beverages
Wang	2004	Prostate cancer	Global	Case-control	Both	15.0	99.0	N/A	N/A	Morbidity	N/A	Non-drinkers	Light drinkers	75	461	N/A	Any alcoholic beverages
Watters	2003	Prostate cancer	United States of America	Prospective Cohort	Male	50.0	71.0	Self-report (human/environment)	Administrative medical records or disease registry	Morbidity	294707	Non-drinkers	Light drinkers	N/A	N/A	N/A	Any alcoholic beverages

Table S9. Summary of study characteristics

Author	Year	Health outcome	Location	Study design	Sex	Age Start	Age End	Exposure Assessment	Outcome Assessment Method	Outcome type	Sample Size	Reference	Exposed group	Cases	Controls	Control Pool	Alcohol Type
Watters	2003	Prostate cancer	United States of America	Prospective Cohort	Male	50.0	71.0	Self-report (human/environment)	Administrative medical records or disease registry	Morbidity	294707	Non-drinkers	Moderate drinkers	N/A	N/A	N/A	Any alcoholic beverages
Lee	2017	Stomach cancer	Republic of Korea	Prospective Cohort	Female	40.0	69.0	Self-report (human/environment)	Administrative medical records or disease registry	Morbidity	128218	Non-drinkers	Light drinkers	N/A	N/A	N/A	Any alcoholic beverages
Nakaya	1997	Stomach cancer	Japan	Prospective Cohort	Both	15.0	99.0	N/A	N/A	Morbidity	N/A	Non-drinkers	Light drinkers	91	N/A	N/A	Any alcoholic beverages
Steevens	2002	Stomach cancer	Netherlands	Prospective Cohort	Both	15.0	99.0	N/A	N/A	Morbidity	N/A	Non-drinkers	Moderate drinkers	166	N/A	N/A	Any alcoholic beverages
Sung	2002	Stomach cancer	Republic of Korea	Prospective Cohort	Male	17.3	70.7	Self-report (human/environment)	Administrative medical records or disease registry	Morbidity	669634	Non-drinkers	Light drinkers	N/A	N/A	N/A	Any alcoholic beverages
Soh	2014	Tuberculosis	Singapore	Prospective Cohort	Both	45.0	74.0	Self-report (human/environment)	Administrative medical records or disease registry	Morbidity	60245	Non-drinkers	Light drinkers	N/A	N/A	N/A	Any alcoholic beverages
Soh	2014	Tuberculosis	Singapore	Prospective Cohort	Both	45.0	74.0	Self-report (human/environment)	Administrative medical records or disease registry	Morbidity	60245	Non-drinkers	Moderate drinkers	N/A	N/A	N/A	Any alcoholic beverages

N/A, not available

B. Summary of Data Inputs

Table S10. Summary of Data Inputs

Author	Year	Health outcome	Sex	Reference	Exposed group	Natural Log of Effect Size	Standard Error of Natural Log of Effect Size	Covariates Adjusted for in Source
Filippini	2019	Alzheimer's disease and other dementias	Both	Light drinkers	Moderate drinkers	0.04	0.01	representativeness, age, sex, bmi, education, income, physical activity, smoking, menopause, other drug use, dietary factors, other, hypertension, diabetes, vitamin deficiency, other diagnosed disease, APOE, family history, hypercholesterolemia
Handing	2010	Alzheimer's disease and other dementias	Both	Light drinkers	Light drinkers	0.05	0.03	representativeness, age, sex, bmi, education, income, physical activity, smoking, menopause, other drug use, dietary factors, other, hypertension, diabetes, vitamin deficiency, other diagnosed disease, APOE, family history, hypercholesterolemia
Handing	2010	Alzheimer's disease and other dementias	Both	Light drinkers	Light drinkers	-0.02	0.03	representativeness, age, sex, bmi, education, income, physical activity, smoking, menopause, other drug use, dietary factors, other, hypertension, diabetes, vitamin deficiency, other diagnosed disease, APOE, family history, hypercholesterolemia
Handing	2010	Alzheimer's disease and other dementias	Both	Light drinkers	Light drinkers	0.10	0.04	representativeness, age, sex, bmi, education, income, physical activity, smoking, menopause, other drug use, dietary factors, other, hypertension, diabetes, vitamin deficiency, other diagnosed disease, APOE, family history, hypercholesterolemia
Heffernan	2015	Alzheimer's disease and other dementias	Both	Non-drinkers	Heavy drinkers	-0.58	0.56	representativeness, age, sex, bmi, education, income, physical activity, smoking, menopause, other drug use, dietary factors, other, hypertension, diabetes, vitamin deficiency, other diagnosed disease, APOE, family history, hypercholesterolemia
Järvenpää	2005	Alzheimer's disease and other dementias	Both	Non-drinkers	Light drinkers	0.00	0.35	representativeness, age, sex, bmi, education, income, physical activity, smoking, menopause, other drug use, dietary factors, other, hypertension, diabetes, vitamin deficiency, other diagnosed disease, APOE, family history, hypercholesterolemia
Järvenpää	2005	Alzheimer's disease and other dementias	Both	Non-drinkers	Moderate drinkers	0.88	0.64	representativeness, age, sex, bmi, education, income, physical activity, smoking, menopause, other drug use, dietary factors, other, hypertension, diabetes, vitamin deficiency, other diagnosed disease, APOE, family history, hypercholesterolemia
Kawakami	2020	Alzheimer's disease and other dementias	Both	Non-drinkers	Moderate drinkers	-0.53	0.22	representativeness, age, sex, bmi, education, income, physical activity, smoking, menopause, other drug use, dietary factors, other, hypertension, diabetes, vitamin deficiency, other diagnosed disease, APOE, family history, hypercholesterolemia
Kawakami	2020	Alzheimer's disease and other dementias	Male	Non-drinkers	Heavy drinkers	-0.94	0.29	representativeness, age, sex, bmi, education, income, physical activity, smoking, menopause, other drug use, dietary factors, other, hypertension, diabetes, vitamin deficiency, other diagnosed disease, APOE, family history, hypercholesterolemia
Kawakami	2020	Alzheimer's disease and other dementias	Female	Non-drinkers	Heavy drinkers	0.38	0.73	representativeness, age, sex, bmi, education, income, physical activity, smoking, menopause, other drug use, dietary factors, other, hypertension, diabetes, vitamin deficiency, other diagnosed disease, APOE, family history, hypercholesterolemia
Kawakami	2020	Alzheimer's disease and other dementias	Both	Non-drinkers	Heavy drinkers	0.06	0.22	representativeness, age, sex, bmi, education, income, physical activity, smoking, menopause, other drug use, dietary factors, other, hypertension, diabetes, vitamin deficiency, other diagnosed disease, APOE, family history, hypercholesterolemia

Table S10. Summary of Data Inputs

Author	Year	Health outcome	Sex	Reference	Exposed group	Natural Log of Effect Size	Standard Error of Natural Log of Effect Size	Covariates Adjusted for in Source
Larrieu	2004	Alzheimer's disease and other dementias	Both	Light drinkers	Heavy drinkers	-0.58	0.24	representativeness, age, sex, bmi, education, income, physical activity, smoking, menopause, other drug use, dietary factors, other, hypertension, diabetes, vitamin deficiency, other diagnosed disease, APOE, family history, hypercholesterolemia
Mewton	2022	Alzheimer's disease and other dementias	Both	Non-drinkers	Light drinkers	-0.16	0.06	representativeness, age, sex, bmi, education, income, physical activity, smoking, menopause, other drug use, dietary factors, other, hypertension, diabetes, vitamin deficiency, other diagnosed disease, APOE, family history, hypercholesterolemia
Ruitenberg	1999	Alzheimer's disease and other dementias	Both	Non-drinkers	Moderate drinkers	-0.54	0.22	representativeness, age, sex, bmi, education, income, physical activity, smoking, menopause, other drug use, dietary factors, other, hypertension, diabetes, vitamin deficiency, other diagnosed disease, APOE, family history, hypercholesterolemia
Ruitenberg	1999	Alzheimer's disease and other dementias	Both	Non-drinkers	Heavy drinkers	0.00	0.48	representativeness, age, sex, bmi, education, income, physical activity, smoking, menopause, other drug use, dietary factors, other, hypertension, diabetes, vitamin deficiency, other diagnosed disease, APOE, family history, hypercholesterolemia
Sabia	2017	Alzheimer's disease and other dementias	Both	Light drinkers	Light drinkers	0.37	0.13	representativeness, age, sex, bmi, education, income, physical activity, smoking, menopause, other drug use, dietary factors, other, hypertension, diabetes, vitamin deficiency, other diagnosed disease, APOE, family history, hypercholesterolemia
Sabia	2017	Alzheimer's disease and other dementias	Both	Light drinkers	Moderate drinkers	0.02	0.14	representativeness, age, sex, bmi, education, income, physical activity, smoking, menopause, other drug use, dietary factors, other, hypertension, diabetes, vitamin deficiency, other diagnosed disease, APOE, family history, hypercholesterolemia
Weyerer	2010	Alzheimer's disease and other dementias	Both	Non-drinkers	Moderate drinkers	-0.92	0.44	representativeness, age, sex, bmi, education, income, physical activity, smoking, menopause, other drug use, dietary factors, other, hypertension, diabetes, vitamin deficiency, other diagnosed disease, APOE, family history, hypercholesterolemia
Jeon	2023	Alzheimer's disease and other dementias	Males	Non Drinker	Current drinker, Light	-0.2357223	0.01938416	age, sex, bmi, education, smoking, physical activity, diet, other diagnosed disease
Jeon	2023	Alzheimer's disease and other dementias	Males	Non Drinker	Current drinker, Moderate	-0.1863296	0.02752269	age, sex, bmi, education, smoking, physical activity, diet, other diagnosed disease
Jeon	2023	Alzheimer's disease and other dementias	Males	Non Drinker	Current drinker, Heavy	0.07696104	0.02363746	age, sex, bmi, education, smoking, physical activity, diet, other diagnosed disease
Jeon	2023	Alzheimer's disease and other dementias	Females	Non Drinker	Current drinker, Light	-0.2484614	0.02927947	age, sex, bmi, education, smoking, physical activity, diet, other diagnosed disease
Jeon	2023	Alzheimer's disease and other dementias	Females	Non Drinker	Current drinker, Moderate	-0.1392621	0.11297261	age, sex, bmi, education, smoking, physical activity, diet, other diagnosed disease
Jeon	2023	Alzheimer's disease and other dementias	Females	Non Drinker	Current drinker, Heavy	0.17395331	0.15250944	age, sex, bmi, education, smoking, physical activity, diet, other diagnosed disease

Table S10. Summary of Data Inputs

Author	Year	Health outcome	Sex	Reference	Exposed group	Natural Log of Effect Size	Standard Error of Natural Log of Effect Size	Covariates Adjusted for in Source
Feter	2022	Alzheimer's disease and other dementias	Both	Current drinker, Moderate	Current drinker, Heavy	0	0.16244751	age, sex, bmi, income, smoking, physical activity, diet,hypertension, other diagnosed disease
Koch	2019	Alzheimer's disease and other dementias	Both	Non drinker	Current drinker, Light	-0.3710637	0.12244898	age, sex, bmi, education, smoking, hypertension
Koch	2019	Alzheimer's disease and other dementias	Both	Current drinker, Light	Current drinker, Light	-0.6348783	0.18112245	age, sex, bmi, education, smoking, hypertension
Koch	2019	Alzheimer's disease and other dementias	Both	Current drinker, Moderate	Current drinker, Light	0.15700375	0.19897959	age, sex, bmi, education, smoking, hypertension
Koch	2019	Alzheimer's disease and other dementias	Both	Current drinker, Heavy (consumes a lot, consistently)	Current drinker, Light	-0.1278334	0.17091837	age, sex, bmi, education, smoking, hypertension
Shimizu	2023	Alzheimer's disease and other dementias	Both	Current drinker, Light	Non Drinker	0.27002714	0.06778142	age, sex, bmi, education, smoking, hypertension
Shimizu	2023	Alzheimer's disease and other dementias	Both	Current drinker, Light	Current drinker, Light	0.13976194	0.09257793	age, sex, bmi, education, smoking, hypertension
Shimizu	2023	Alzheimer's disease and other dementias	Both	Current drinker, Light	Current drinker, Moderate	0.06765865	0.08785138	age, sex, bmi, education, smoking, hypertension
Shimizu	2023	Alzheimer's disease and other dementias	Both	Current drinker, Light	Current drinker, Heavy	0.19885086	0.08973493	age, sex, bmi, education, smoking, hypertension
Shimizu	2023	Alzheimer's disease and other dementias	Both	Current drinker, Light	Current drinker, Heavy	0.41871034	0.09038519	age, sex, bmi, education, smoking, hypertension
Jung	2024	Alzheimer's disease and other dementias	Both	Non Drinker	Current drinker, Light	-0.1508229	0.01785714	age, sex, bmi, education, smoking, hypertension, other disease
Jung	2024	Alzheimer's disease and other dementias	Both	Non Drinker	Current drinker, Moderate	-0.0512933	0.0255102	age, sex, bmi, education, smoking, hypertension, other disease
Jung	2024	Alzheimer's disease and other dementias	Both	Non Drinker	Current drinker, Heavy (consumes a lot, consistently)	0.12221763	0.03061224	age, sex, bmi, education, smoking, hypertension, other disease
Jung	2024	Alzheimer's disease and other dementias	Both	Non Drinker	Current drinker, Light	-0.0943107	0.00510204	age, sex, bmi, education, smoking, hypertension, other disease
Jung	2024	Alzheimer's disease and other dementias	Both	Non Drinker	Current drinker, Moderate	-0.0512933	0.01020408	age, sex, bmi, education, smoking, hypertension, other disease

Table S10. Summary of Data Inputs

Author	Year	Health outcome	Sex	Reference	Exposed group	Natural Log of Effect Size	Standard Error of Natural Log of Effect Size	Covariates Adjusted for in Source
Jung	2024	Alzheimer's disease and other dementias	Both	Non Drinker	Current drinker, Heavy (consumes a lot, consistently)	0.06765865	0.0127551	age, sex, bmi, education, smoking, hypertension, other disease
Topiwala	2025	Alzheimer's disease and other dementias	Both	Current drinker, Light	Non drinker	0.31481074	0.03061224	age, sex, bmi, education, income, smoking
Topiwala	2025	Alzheimer's disease and other dementias	Both	Current drinker, Light	Current drinker, Moderate	-0.0618754	0.05612245	age, sex, bmi, education, income, smoking
Topiwala	2025	Alzheimer's disease and other dementias	Both	Current drinker, Light	Current drinker, Moderate	0.10436002	0.11479592	age, sex, bmi, education, income, smoking
Topiwala	2025	Alzheimer's disease and other dementias	Both	Current drinker, Light	Current drinker, Heavy (consumes a lot, consistently)	0.13102826	0.20918367	age, sex, bmi, education, income, smoking
Topiwala	2025	Alzheimer's disease and other dementias	Both	Current drinker, Light	Current drinker, Heavy (consumes a lot, consistently)	0.47623418	0.23469388	age, sex, bmi, education, income, smoking
Topiwala	2025	Alzheimer's disease and other dementias	Both	Current drinker, Light	Never drinker	0.27002714	0.06377551	age, sex, bmi, education, income, smoking
Topiwala	2025	Alzheimer's disease and other dementias	Both	Current drinker, Light	Current drinker, Moderate	-0.0943107	0.06122449	age, sex, bmi, education, income, smoking
Topiwala	2025	Alzheimer's disease and other dementias	Both	Current drinker, Light	Current drinker, Moderate	-0.0725707	0.06122449	age, sex, bmi, education, income, smoking
Topiwala	2025	Alzheimer's disease and other dementias	Both	Current drinker, Light	Current drinker, Heavy (consumes a lot, consistently)	-0.0512933	0.06122449	age, sex, bmi, education, income, smoking
Topiwala	2025	Alzheimer's disease and other dementias	Both	Current drinker, Light	Current drinker, Heavy (consumes a lot, consistently)	0.26236426	0.13265306	age, sex, bmi, education, income, smoking
Larsson	2018	Alzheimer's disease and other dementias	Females	Never drinker	Non Drinker	-0.0202027	0.06122449	age, sex, bmi, education, income, smoking
Larsson	2018	Alzheimer's disease and other dementias	Females	Never drinker	Current drinker, Light	-0.0304592	0.06122449	age, sex, bmi, education, income, smoking
Larsson	2018	Alzheimer's disease and other dementias	Females	Never drinker	Current drinker, Moderate	-0.1625189	0.10459184	age, sex, bmi, education, income, smoking

Table S10. Summary of Data Inputs

Author	Year	Health outcome	Sex	Reference	Exposed group	Natural Log of Effect Size	Standard Error of Natural Log of Effect Size	Covariates Adjusted for in Source
Larsson	2018	Alzheimer's disease and other dementias	Females	Never drinker	Current drinker, Heavy	-0.0618754	0.32142857	age, sex, bmi, education, income, smoking
Larsson	2018	Alzheimer's disease and other dementias	Females	Never drinker	Current drinker, Heavy (consumes a lot, consistently)	-0.4462871	0.29081633	age, sex, bmi, education, income, smoking
Larsson	2018	Alzheimer's disease and other dementias	Males	Never drinker	Non Drinker	0.16551444	0.13520408	age, sex, bmi, education, income, smoking
Larsson	2018	Alzheimer's disease and other dementias	Males	Never drinker	Current drinker, Light	0.00995033	0.10204082	age, sex, bmi, education, income, smoking
Larsson	2018	Alzheimer's disease and other dementias	Males	Never drinker	Current drinker, Moderate	0.0295588	0.11479592	age, sex, bmi, education, income, smoking
Larsson	2018	Alzheimer's disease and other dementias	Males	Never drinker	Current drinker, Heavy	0.00995033	0.15561224	age, sex, bmi, education, income, smoking
Larsson	2018	Alzheimer's disease and other dementias	Males	Never drinker	Current drinker, Heavy (consumes a lot, consistently)	0.05826891	0.16836735	age, sex, bmi, education, income, smoking
Ariansen	2019	Atrial fibrillation and flutter	Both	Light drinkers	Light drinkers	-0.03	0.03	representativeness, age, sex, bmi, education, income, physical activity, smoking, menopause, other drug use, dietary factors, other, hypertension, diabetes, vitamin deficiency, other diagnosed disease, APOE, family history, hypercholesterolemia
Ariansen	2019	Atrial fibrillation and flutter	Both	Light drinkers	Light drinkers	0.10	0.06	representativeness, age, sex, bmi, education, income, physical activity, smoking, menopause, other drug use, dietary factors, other, hypertension, diabetes, vitamin deficiency, other diagnosed disease, APOE, family history, hypercholesterolemia
Bazal	2017	Atrial fibrillation and flutter	Both	Non-drinkers	Light drinkers	0.07	0.18	representativeness, age, sex, bmi, education, income, physical activity, smoking, menopause, other drug use, dietary factors, other, hypertension, diabetes, vitamin deficiency, other diagnosed disease, APOE, family history, hypercholesterolemia
Bazal	2017	Atrial fibrillation and flutter	Both	Non-drinkers	Heavy drinkers	0.03	0.22	representativeness, age, sex, bmi, education, income, physical activity, smoking, menopause, other drug use, dietary factors, other, hypertension, diabetes, vitamin deficiency, other diagnosed disease, APOE, family history, hypercholesterolemia
Bazal	2017	Atrial fibrillation and flutter	Both	Non-drinkers	Moderate drinkers	0.06	0.28	representativeness, age, sex, bmi, education, income, physical activity, smoking, menopause, other drug use, dietary factors, other, hypertension, diabetes, vitamin deficiency, other diagnosed disease, APOE, family history, hypercholesterolemia
Biddinger	2016	Atrial fibrillation and flutter	Both	Non-drinkers	Light drinkers	0.00	0.06	representativeness, age, sex, bmi, education, income, physical activity, smoking, menopause, other drug use, dietary factors, other, hypertension, diabetes, vitamin deficiency, other diagnosed disease, APOE, family history, hypercholesterolemia

Table S10. Summary of Data Inputs

Author	Year	Health outcome	Sex	Reference	Exposed group	Natural Log of Effect Size	Standard Error of Natural Log of Effect Size	Covariates Adjusted for in Source
Cha	2015	Atrial fibrillation and flutter	Both	Light drinkers	Light drinkers	0.62	0.26	representativeness, age, sex, bmi, education, income, physical activity, smoking, menopause, other drug use, dietary factors, other, hypertension, diabetes, vitamin deficiency, other diagnosed disease, APOE, family history, hypercholesterolemia
Csengeri	2010	Atrial fibrillation and flutter	Both	Non-drinkers	Light drinkers	0.01	0.01	representativeness, age, sex, bmi, education, income, physical activity, smoking, menopause, other drug use, dietary factors, other, hypertension, diabetes, vitamin deficiency, other diagnosed disease, APOE, family history, hypercholesterolemia
Csengeri	2010	Atrial fibrillation and flutter	Both	Non-drinkers	Light drinkers	0.02	0.01	representativeness, age, sex, bmi, education, income, physical activity, smoking, menopause, other drug use, dietary factors, other, hypertension, diabetes, vitamin deficiency, other diagnosed disease, APOE, family history, hypercholesterolemia
Csengeri	2010	Atrial fibrillation and flutter	Both	Non-drinkers	Light drinkers	0.04	0.01	representativeness, age, sex, bmi, education, income, physical activity, smoking, menopause, other drug use, dietary factors, other, hypertension, diabetes, vitamin deficiency, other diagnosed disease, APOE, family history, hypercholesterolemia
Csengeri	2010	Atrial fibrillation and flutter	Both	Non-drinkers	Light drinkers	0.05	0.01	representativeness, age, sex, bmi, education, income, physical activity, smoking, menopause, other drug use, dietary factors, other, hypertension, diabetes, vitamin deficiency, other diagnosed disease, APOE, family history, hypercholesterolemia
Csengeri	2010	Atrial fibrillation and flutter	Both	Non-drinkers	Light drinkers	0.06	0.01	representativeness, age, sex, bmi, education, income, physical activity, smoking, menopause, other drug use, dietary factors, other, hypertension, diabetes, vitamin deficiency, other diagnosed disease, APOE, family history, hypercholesterolemia
Csengeri	2010	Atrial fibrillation and flutter	Both	Non-drinkers	Light drinkers	0.07	0.01	representativeness, age, sex, bmi, education, income, physical activity, smoking, menopause, other drug use, dietary factors, other, hypertension, diabetes, vitamin deficiency, other diagnosed disease, APOE, family history, hypercholesterolemia
Csengeri	2010	Atrial fibrillation and flutter	Both	Non-drinkers	Moderate drinkers	0.42	0.06	representativeness, age, sex, bmi, education, income, physical activity, smoking, menopause, other drug use, dietary factors, other, hypertension, diabetes, vitamin deficiency, other diagnosed disease, APOE, family history, hypercholesterolemia
Di Benedetto	2017	Atrial fibrillation and flutter	Female	Light drinkers	Moderate drinkers	0.03	0.12	representativeness, age, sex, bmi, education, income, physical activity, smoking, menopause, other drug use, dietary factors, other, hypertension, diabetes, vitamin deficiency, other diagnosed disease, APOE, family history, hypercholesterolemia
Di Castelnuovo	2015	Atrial fibrillation and flutter	Both	Non-drinkers	Light drinkers	-0.02	0.14	representativeness, age, sex, bmi, education, income, physical activity, smoking, menopause, other drug use, dietary factors, other, hypertension, diabetes, vitamin deficiency, other diagnosed disease, APOE, family history, hypercholesterolemia
Di Castelnuovo	2015	Atrial fibrillation and flutter	Both	Non-drinkers	Light drinkers	-0.08	0.18	representativeness, age, sex, bmi, education, income, physical activity, smoking, menopause, other drug use, dietary factors, other, hypertension, diabetes, vitamin deficiency, other diagnosed disease, APOE, family history, hypercholesterolemia
Di Castelnuovo	2015	Atrial fibrillation and flutter	Both	Non-drinkers	Light drinkers	-0.04	0.14	representativeness, age, sex, bmi, education, income, physical activity, smoking, menopause, other drug use, dietary factors, other, hypertension, diabetes, vitamin deficiency, other diagnosed disease, APOE, family history, hypercholesterolemia

Table S10. Summary of Data Inputs

Author	Year	Health outcome	Sex	Reference	Exposed group	Natural Log of Effect Size	Standard Error of Natural Log of Effect Size	Covariates Adjusted for in Source
Di Castelnuovo	2015	Atrial fibrillation and flutter	Both	Non-drinkers	Heavy drinkers	-0.16	0.18	representativeness, age, sex, bmi, education, income, physical activity, smoking, menopause, other drug use, dietary factors, other, hypertension, diabetes, vitamin deficiency, other diagnosed disease, APOE, family history, hypercholesterolemia
Dixit	2013	Atrial fibrillation and flutter	Both	Non-drinkers	Light drinkers	-0.07	0.08	representativeness, age, sex, bmi, education, income, physical activity, smoking, menopause, other drug use, dietary factors, other, hypertension, diabetes, vitamin deficiency, other diagnosed disease, APOE, family history, hypercholesterolemia
Djousse	1998	Atrial fibrillation and flutter	Both	Non-drinkers	Light drinkers	0.06	0.14	age, sex, bmi, education, income, physical activity, other, hypertension, other diagnosed disease
Djousse	1998	Atrial fibrillation and flutter	Both	Non-drinkers	Heavy drinkers	0.29	0.14	age, sex, bmi, education, income, physical activity, other, hypertension, other diagnosed disease
Djousse	1998	Atrial fibrillation and flutter	Both	Non-drinkers	Light drinkers	-0.03	0.11	age, sex, bmi, education, income, physical activity, other, hypertension, other diagnosed disease
Frederiksen	2017	Atrial fibrillation and flutter	Both	Light drinkers	Light drinkers	-0.01	0.07	representativeness, age, sex, bmi, education, income, physical activity, smoking, menopause, other drug use, dietary factors, other, hypertension, diabetes, vitamin deficiency, other diagnosed disease, APOE, family history, hypercholesterolemia
Frederiksen	2017	Atrial fibrillation and flutter	Both	Light drinkers	Moderate drinkers	0.01	0.11	representativeness, age, sex, bmi, education, income, physical activity, smoking, menopause, other drug use, dietary factors, other, hypertension, diabetes, vitamin deficiency, other diagnosed disease, APOE, family history, hypercholesterolemia
Frederiksen	2017	Atrial fibrillation and flutter	Both	Light drinkers	Light drinkers	0.02	0.06	representativeness, age, sex, bmi, education, income, physical activity, smoking, menopause, other drug use, dietary factors, other, hypertension, diabetes, vitamin deficiency, other diagnosed disease, APOE, family history, hypercholesterolemia
Frederiksen	2017	Atrial fibrillation and flutter	Both	Moderate drinkers	Light drinkers	-0.07	0.09	representativeness, age, sex, bmi, education, income, physical activity, smoking, menopause, other drug use, dietary factors, other, hypertension, diabetes, vitamin deficiency, other diagnosed disease, APOE, family history, hypercholesterolemia
Frederiksen	2017	Atrial fibrillation and flutter	Both	Moderate drinkers	Moderate drinkers	-0.03	0.09	representativeness, age, sex, bmi, education, income, physical activity, smoking, menopause, other drug use, dietary factors, other, hypertension, diabetes, vitamin deficiency, other diagnosed disease, APOE, family history, hypercholesterolemia
Frederiksen	2017	Atrial fibrillation and flutter	Both	Heavy drinkers	Moderate drinkers	0.24	0.12	representativeness, age, sex, bmi, education, income, physical activity, smoking, menopause, other drug use, dietary factors, other, hypertension, diabetes, vitamin deficiency, other diagnosed disease, APOE, family history, hypercholesterolemia
Frederiksen	2017	Atrial fibrillation and flutter	Both	Light drinkers	Moderate drinkers	0.08	0.12	representativeness, age, sex, bmi, education, income, physical activity, smoking, menopause, other drug use, dietary factors, other, hypertension, diabetes, vitamin deficiency, other diagnosed disease, APOE, family history, hypercholesterolemia

Table S10. Summary of Data Inputs

Author	Year	Health outcome	Sex	Reference	Exposed group	Natural Log of Effect Size	Standard Error of Natural Log of Effect Size	Covariates Adjusted for in Source
Frederiksen	2017	Atrial fibrillation and flutter	Both	Light drinkers	Moderate drinkers	0.08	0.08	representativeness, age, sex, bmi, education, income, physical activity, smoking, menopause, other drug use, dietary factors, other, hypertension, diabetes, vitamin deficiency, other diagnosed disease, APOE, family history, hypercholesterolemia
Frost	1997	Atrial fibrillation and flutter	Both	Light drinkers	Moderate drinkers	0.13	0.25	age, sex, bmi, education, income, physical activity, smoking, other, hypertension
Frost	1997	Atrial fibrillation and flutter	Both	Light drinkers	Heavy drinkers	0.38	0.17	age, sex, bmi, education, income, physical activity, smoking, other, hypertension
Frost	1997	Atrial fibrillation and flutter	Both	Light drinkers	Light drinkers	0.04	0.18	age, sex, bmi, education, income, physical activity, smoking, other, hypertension
Frost	1997	Atrial fibrillation and flutter	Both	Light drinkers	Light drinkers	0.24	0.24	age, sex, bmi, education, income, physical activity, smoking, other, hypertension
Frost	1997	Atrial fibrillation and flutter	Both	Light drinkers	Light drinkers	0.09	0.24	age, sex, bmi, education, income, physical activity, smoking, other, hypertension
Gorecka	2014	Atrial fibrillation and flutter	Both	Non-drinkers	Light drinkers	0.06	0.08	representativeness, age, sex, bmi, education, income, physical activity, smoking, menopause, other drug use, dietary factors, other, hypertension, diabetes, vitamin deficiency, other diagnosed disease, APOE, family history, hypercholesterolemia
Gorecka	2014	Atrial fibrillation and flutter	Both	Non-drinkers	Moderate drinkers	0.01	0.20	representativeness, age, sex, bmi, education, income, physical activity, smoking, menopause, other drug use, dietary factors, other, hypertension, diabetes, vitamin deficiency, other diagnosed disease, APOE, family history, hypercholesterolemia
Gémes,	2015	Atrial fibrillation and flutter	Both	Non-drinkers	Light drinkers	0.04	0.09	representativeness, age, sex, bmi, education, income, physical activity, smoking, menopause, other drug use, dietary factors, other, hypertension, diabetes, vitamin deficiency, other diagnosed disease, APOE, family history, hypercholesterolemia
Gémes,	2015	Atrial fibrillation and flutter	Both	Non-drinkers	Light drinkers	0.00	0.16	representativeness, age, sex, bmi, education, income, physical activity, smoking, menopause, other drug use, dietary factors, other, hypertension, diabetes, vitamin deficiency, other diagnosed disease, APOE, family history, hypercholesterolemia
Gémes,	2015	Atrial fibrillation and flutter	Both	Non-drinkers	Light drinkers	0.05	0.10	representativeness, age, sex, bmi, education, income, physical activity, smoking, menopause, other drug use, dietary factors, other, hypertension, diabetes, vitamin deficiency, other diagnosed disease, APOE, family history, hypercholesterolemia
Gémes,	2015	Atrial fibrillation and flutter	Both	Non-drinkers	Light drinkers	0.02	0.08	representativeness, age, sex, bmi, education, income, physical activity, smoking, menopause, other drug use, dietary factors, other, hypertension, diabetes, vitamin deficiency, other diagnosed disease, APOE, family history, hypercholesterolemia
Gémes,	2015	Atrial fibrillation and flutter	Both	Non-drinkers	Light drinkers	0.06	0.11	representativeness, age, sex, bmi, education, income, physical activity, smoking, menopause, other drug use, dietary factors, other, hypertension, diabetes, vitamin deficiency, other diagnosed disease, APOE, family history, hypercholesterolemia

Table S10. Summary of Data Inputs

Author	Year	Health outcome	Sex	Reference	Exposed group	Natural Log of Effect Size	Standard Error of Natural Log of Effect Size	Covariates Adjusted for in Source
Gémes,	2015	Atrial fibrillation and flutter	Both	Non-drinkers	Light drinkers	0.06	0.09	representativeness, age, sex, bmi, education, income, physical activity, smoking, menopause, other drug use, dietary factors, other, hypertension, diabetes, vitamin deficiency, other diagnosed disease, APOE, family history, hypercholesterolemia
Halford	2019	Atrial fibrillation and flutter	Both	Non-drinkers	Heavy drinkers	0.10	0.03	representativeness, age, sex, bmi, education, income, physical activity, smoking, menopause, other drug use, dietary factors, other, hypertension, diabetes, vitamin deficiency, other diagnosed disease, APOE, family history, hypercholesterolemia
Halford	2019	Atrial fibrillation and flutter	Both	Non-drinkers	Heavy drinkers	0.00	0.61	representativeness, age, sex, bmi, education, income, physical activity, smoking, menopause, other drug use, dietary factors, other, hypertension, diabetes, vitamin deficiency, other diagnosed disease, APOE, family history, hypercholesterolemia
Hamada	2015	Atrial fibrillation and flutter	Both	Non-drinkers	Moderate drinkers	0.08	0.14	representativeness, age, sex, bmi, education, income, physical activity, smoking, menopause, other drug use, dietary factors, other, hypertension, diabetes, vitamin deficiency, other diagnosed disease, APOE, family history, hypercholesterolemia
Hamada	2015	Atrial fibrillation and flutter	Female	Light drinkers	Light drinkers	-0.04	0.61	representativeness, age, sex, bmi, education, income, physical activity, smoking, menopause, other drug use, dietary factors, other, hypertension, diabetes, vitamin deficiency, other diagnosed disease, APOE, family history, hypercholesterolemia
Han	2012	Atrial fibrillation and flutter	Both	Non-drinkers	Light drinkers	-0.03	0.05	representativeness, age, sex, bmi, education, income, physical activity, smoking, menopause, other drug use, dietary factors, other, hypertension, diabetes, vitamin deficiency, other diagnosed disease, APOE, family history, hypercholesterolemia
Han	2012	Atrial fibrillation and flutter	Both	Non-drinkers	Moderate drinkers	0.04	0.06	representativeness, age, sex, bmi, education, income, physical activity, smoking, menopause, other drug use, dietary factors, other, hypertension, diabetes, vitamin deficiency, other diagnosed disease, APOE, family history, hypercholesterolemia
Johansson	2014	Atrial fibrillation and flutter	Female	Light drinkers	Light drinkers	-0.04	0.07	representativeness, age, sex, bmi, education, income, physical activity, smoking, menopause, other drug use, dietary factors, other, hypertension, diabetes, vitamin deficiency, other diagnosed disease, APOE, family history, hypercholesterolemia
Johansson	2014	Atrial fibrillation and flutter	Female	Light drinkers	Light drinkers	-0.16	0.07	representativeness, age, sex, bmi, education, income, physical activity, smoking, menopause, other drug use, dietary factors, other, hypertension, diabetes, vitamin deficiency, other diagnosed disease, APOE, family history, hypercholesterolemia
Johansson	2014	Atrial fibrillation and flutter	Female	Light drinkers	Light drinkers	-0.09	0.07	representativeness, age, sex, bmi, education, income, physical activity, smoking, menopause, other drug use, dietary factors, other, hypertension, diabetes, vitamin deficiency, other diagnosed disease, APOE, family history, hypercholesterolemia
Kim	2017	Atrial fibrillation and flutter	Both	Light drinkers	Light drinkers	0.07	0.01	representativeness, age, sex, bmi, education, income, physical activity, smoking, menopause, other drug use, dietary factors, other, hypertension, diabetes, vitamin deficiency, other diagnosed disease, APOE, family history, hypercholesterolemia
Kim	2017	Atrial fibrillation and flutter	Both	Light drinkers	Moderate drinkers	0.07	0.01	representativeness, age, sex, bmi, education, income, physical activity, smoking, menopause, other drug use, dietary factors, other, hypertension, diabetes, vitamin deficiency, other diagnosed disease, APOE, family history, hypercholesterolemia

Table S10. Summary of Data Inputs

Author	Year	Health outcome	Sex	Reference	Exposed group	Natural Log of Effect Size	Standard Error of Natural Log of Effect Size	Covariates Adjusted for in Source
Kim	2017	Atrial fibrillation and flutter	Both	Light drinkers	Moderate drinkers	0.21	0.01	representativeness, age, sex, bmi, education, income, physical activity, smoking, menopause, other drug use, dietary factors, other, hypertension, diabetes, vitamin deficiency, other diagnosed disease, APOE, family history, hypercholesterolemia
Lee	2015	Atrial fibrillation and flutter	Both	Light drinkers	Moderate drinkers	0.04	0.02	representativeness, age, sex, bmi, education, income, physical activity, smoking, menopause, other drug use, dietary factors, other, hypertension, diabetes, vitamin deficiency, other diagnosed disease, APOE, family history, hypercholesterolemia
Marcus	2007	Atrial fibrillation and flutter	Both	Non-drinkers	Light drinkers	0.83	0.60	age, sex, bmi, education, income, physical activity, other, hypertension, other diagnosed disease
Marcus	2007	Atrial fibrillation and flutter	Both	Non-drinkers	Light drinkers	0.92	0.73	age, sex, bmi, education, income, physical activity, other, hypertension, other diagnosed disease
Marcus	2007	Atrial fibrillation and flutter	Both	Non-drinkers	Light drinkers	-0.03	0.60	age, sex, bmi, education, income, physical activity, other, hypertension, other diagnosed disease
Martín-Pérez	2005	Atrial fibrillation and flutter	Both	Non-drinkers	Moderate drinkers	0.08	0.12	representativeness, age, sex, bmi, education, income, physical activity, smoking, menopause, other drug use, dietary factors, other, hypertension, diabetes, vitamin deficiency, other diagnosed disease, APOE, family history, hypercholesterolemia
McManus	2013	Atrial fibrillation and flutter	Both	Light drinkers	Light drinkers	0.05	0.02	representativeness, age, sex, bmi, education, income, physical activity, smoking, menopause, other drug use, dietary factors, other, hypertension, diabetes, vitamin deficiency, other diagnosed disease, APOE, family history, hypercholesterolemia
Morseth	2020	Atrial fibrillation and flutter	Both	Light drinkers	Moderate drinkers	-0.08	0.08	representativeness, age, sex, bmi, education, income, physical activity, smoking, menopause, other drug use, dietary factors, other, hypertension, diabetes, vitamin deficiency, other diagnosed disease, APOE, family history, hypercholesterolemia
Morseth	2020	Atrial fibrillation and flutter	Both	Light drinkers	Moderate drinkers	-0.08	0.08	representativeness, age, sex, bmi, education, income, physical activity, smoking, menopause, other drug use, dietary factors, other, hypertension, diabetes, vitamin deficiency, other diagnosed disease, APOE, family history, hypercholesterolemia
Morseth	2020	Atrial fibrillation and flutter	Both	Light drinkers	Moderate drinkers	-0.08	0.08	representativeness, age, sex, bmi, education, income, physical activity, smoking, menopause, other drug use, dietary factors, other, hypertension, diabetes, vitamin deficiency, other diagnosed disease, APOE, family history, hypercholesterolemia
Morseth	2020	Atrial fibrillation and flutter	Both	Light drinkers	Moderate drinkers	-0.08	0.08	representativeness, age, sex, bmi, education, income, physical activity, smoking, menopause, other drug use, dietary factors, other, hypertension, diabetes, vitamin deficiency, other diagnosed disease, APOE, family history, hypercholesterolemia
Morseth	2020	Atrial fibrillation and flutter	Both	Light drinkers	Moderate drinkers	-0.08	0.08	representativeness, age, sex, bmi, education, income, physical activity, smoking, menopause, other drug use, dietary factors, other, hypertension, diabetes, vitamin deficiency, other diagnosed disease, APOE, family history, hypercholesterolemia

Table S10. Summary of Data Inputs

Author	Year	Health outcome	Sex	Reference	Exposed group	Natural Log of Effect Size	Standard Error of Natural Log of Effect Size	Covariates Adjusted for in Source
Park	2012	Atrial fibrillation and flutter	Both	Non-drinkers	Light drinkers	0.01	0.01	representativeness, age, sex, bmi, education, income, physical activity, smoking, menopause, other drug use, dietary factors, other, hypertension, diabetes, vitamin deficiency, other diagnosed disease, APOE, family history, hypercholesterolemia
Psaty	1993	Atrial fibrillation and flutter	Both	Light drinkers	Light drinkers	-0.06	0.02	representativeness, age, sex, bmi, education, income, physical activity, smoking, menopause, other drug use, dietary factors, other, hypertension, diabetes, vitamin deficiency, other diagnosed disease, APOE, family history, hypercholesterolemia
Ruigomez	1997	Atrial fibrillation and flutter	Both	Non-drinkers	Heavy drinkers	0.88	0.27	age, sex, bmi, education, income, physical activity, other diagnosed disease
Ruigomez	1997	Atrial fibrillation and flutter	Both	Non-drinkers	Moderate drinkers	0.26	0.18	age, sex, bmi, education, income, physical activity, other diagnosed disease
Ruigomez	1997	Atrial fibrillation and flutter	Both	Non-drinkers	Light drinkers	0.10	0.14	age, sex, bmi, education, income, physical activity, other diagnosed disease
Siddiqi	2020	Atrial fibrillation and flutter	Female	Light drinkers	Light drinkers	0.02	0.12	representativeness, age, sex, bmi, education, income, physical activity, smoking, menopause, other drug use, dietary factors, other, hypertension, diabetes, vitamin deficiency, other diagnosed disease, APOE, family history, hypercholesterolemia
Siddiqi	2020	Atrial fibrillation and flutter	Male	Light drinkers	Light drinkers	0.17	0.13	representativeness, age, sex, bmi, education, income, physical activity, smoking, menopause, other drug use, dietary factors, other, hypertension, diabetes, vitamin deficiency, other diagnosed disease, APOE, family history, hypercholesterolemia
Siddiqi	2020	Atrial fibrillation and flutter	Male	Light drinkers	Light drinkers	0.17	0.19	representativeness, age, sex, bmi, education, income, physical activity, smoking, menopause, other drug use, dietary factors, other, hypertension, diabetes, vitamin deficiency, other diagnosed disease, APOE, family history, hypercholesterolemia
Tu	2020	Atrial fibrillation and flutter	Both	Light drinkers	Light drinkers	-0.05	0.02	representativeness, age, sex, bmi, education, income, physical activity, smoking, menopause, other drug use, dietary factors, other, hypertension, diabetes, vitamin deficiency, other diagnosed disease, APOE, family history, hypercholesterolemia
Tu	2020	Atrial fibrillation and flutter	Both	Light drinkers	Light drinkers	0.01	0.03	representativeness, age, sex, bmi, education, income, physical activity, smoking, menopause, other drug use, dietary factors, other, hypertension, diabetes, vitamin deficiency, other diagnosed disease, APOE, family history, hypercholesterolemia
Tu	2020	Atrial fibrillation and flutter	Both	Light drinkers	Moderate drinkers	0.09	0.03	representativeness, age, sex, bmi, education, income, physical activity, smoking, menopause, other drug use, dietary factors, other, hypertension, diabetes, vitamin deficiency, other diagnosed disease, APOE, family history, hypercholesterolemia
Adami	1985	Breast cancer	Both	Non-drinkers	Light drinkers	0.10	0.40	age, sex, bmi, education, income, physical activity, smoking, other drug use, other, family history
Bassett	1994	Breast cancer	Female	Non-drinkers	Light drinkers	-0.05	0.06	representativeness, age, sex, bmi, education, income, physical activity, smoking, menopause, other drug use, dietary factors, other, hypertension, diabetes, vitamin deficiency, other diagnosed disease, APOE, family history, hypercholesterolemia

Table S10. Summary of Data Inputs

Author	Year	Health outcome	Sex	Reference	Exposed group	Natural Log of Effect Size	Standard Error of Natural Log of Effect Size	Covariates Adjusted for in Source
Bassett	1994	Breast cancer	Female	Non-drinkers	Moderate drinkers	0.26	0.10	representativeness, age, sex, bmi, education, income, physical activity, smoking, menopause, other drug use, dietary factors, other, hypertension, diabetes, vitamin deficiency, other diagnosed disease, APOE, family history, hypercholesterolemia
Benzon	2006	Breast cancer	Both	Non-drinkers	Light drinkers	-0.07	0.32	age, sex, bmi, education, income, physical activity, menopause, other
Berstad	2003	Breast cancer	Both	Non-drinkers	Moderate drinkers	0.60	0.30	age, sex, bmi, education, income, physical activity, smoking, other, other diagnosed disease, family history
Bowlin	1986	Breast cancer	Both	Non-drinkers	Light drinkers	0.38	0.13	age, sex, bmi, education, income, physical activity, smoking, other, other diagnosed disease, family history
Chen	2008	Breast cancer	Both	Non-drinkers	Light drinkers	0.07	0.03	age, sex, bmi, education, income, physical activity, smoking, menopause, other drug use, other, other diagnosed disease
Donat-Vargas	2011	Breast cancer	Female	Light drinkers	Light drinkers	-0.04	0.16	representativeness, age, sex, bmi, education, income, physical activity, smoking, menopause, other drug use, dietary factors, other, hypertension, diabetes, vitamin deficiency, other diagnosed disease, APOE, family history, hypercholesterolemia
Dumeaux	2001	Breast cancer	Both	Non-drinkers	Light drinkers	0.52	0.12	representativeness, age, sex, bmi, education, income, physical activity, menopause, other drug use, other, family history
Ericson	2003	Breast cancer	Both	Non-drinkers	Moderate drinkers	0.92	0.33	age, sex, bmi, education, income, physical activity, menopause, other diagnosed disease
Ewertz	1984	Breast cancer	Both	Non-drinkers	Light drinkers	-0.06	0.09	age, sex, bmi, education, income, physical activity, other diagnosed disease
Ewertz	1984	Breast cancer	Both	Non-drinkers	Light drinkers	0.26	0.20	age, sex, bmi, education, income, physical activity, other diagnosed disease
Ewertz	1984	Breast cancer	Both	Non-drinkers	Light drinkers	0.17	0.18	age, sex, bmi, education, income, physical activity, other diagnosed disease
Ewertz	1984	Breast cancer	Both	Non-drinkers	Light drinkers	-0.04	0.09	age, sex, bmi, education, income, physical activity, other diagnosed disease
Ewertz	1984	Breast cancer	Both	Non-drinkers	Light drinkers	0.03	0.09	age, sex, bmi, education, income, physical activity, other diagnosed disease
Ewertz	1984	Breast cancer	Both	Non-drinkers	Light drinkers	0.01	0.21	age, sex, bmi, education, income, physical activity, other diagnosed disease
Ewertz	1984	Breast cancer	Both	Non-drinkers	Light drinkers	0.00	0.11	age, sex, bmi, education, income, physical activity, other diagnosed disease
Ewertz	1984	Breast cancer	Both	Non-drinkers	Light drinkers	0.24	0.25	age, sex, bmi, education, income, physical activity, other diagnosed disease
Ewertz	1984	Breast cancer	Both	Non-drinkers	Light drinkers	-0.01	0.16	age, sex, bmi, education, income, physical activity, other diagnosed disease
Ewertz	1984	Breast cancer	Both	Non-drinkers	Light drinkers	-0.01	0.15	age, sex, bmi, education, income, physical activity, other diagnosed disease
Ewertz	1984	Breast cancer	Both	Non-drinkers	Light drinkers	-0.01	0.16	age, sex, bmi, education, income, physical activity, other diagnosed disease
Ewertz	1984	Breast cancer	Both	Non-drinkers	Light drinkers	-0.01	0.15	age, sex, bmi, education, income, physical activity, other diagnosed disease
Ferraroni	1985	Breast cancer	Both	Non-drinkers	Light drinkers	0.10	0.38	age, sex, bmi, education, income, physical activity, menopause, other, other diagnosed disease, family history

Table S10. Summary of Data Inputs

Author	Year	Health outcome	Sex	Reference	Exposed group	Natural Log of Effect Size	Standard Error of Natural Log of Effect Size	Covariates Adjusted for in Source
Floud	2001	Breast cancer	Female	Light drinkers	Light drinkers	0.05	0.01	representativeness, age, sex, bmi, education, income, physical activity, smoking, menopause, other drug use, dietary factors, other, hypertension, diabetes, vitamin deficiency, other diagnosed disease, APOE, family history, hypercholesterolemia
Floud	2001	Breast cancer	Female	Light drinkers	Moderate drinkers	0.24	0.02	representativeness, age, sex, bmi, education, income, physical activity, smoking, menopause, other drug use, dietary factors, other, hypertension, diabetes, vitamin deficiency, other diagnosed disease, APOE, family history, hypercholesterolemia
Gapstur	1989	Breast cancer	Both	Non-drinkers	Light drinkers	0.38	0.17	age, sex, bmi, education, income, physical activity, menopause, other, other diagnosed disease, family history
Harris	2008	Breast cancer	Both	Non-drinkers	Light drinkers	-0.06	0.15	age, sex, bmi, education, income, physical activity, smoking, menopause, other
Harris	2008	Breast cancer	Both	Non-drinkers	Light drinkers	-0.05	0.13	age, sex, bmi, education, income, physical activity, smoking, menopause, other
Hirose	2000	Breast cancer	Both	Non-drinkers	Light drinkers	0.10	0.09	age, sex, bmi, education, income, physical activity
Hoyer	1989	Breast cancer	Both	Non-drinkers	Light drinkers	0.26	0.32	age, sex, bmi, education, income, physical activity, smoking, menopause, dietary factors, other
Jain	1993	Breast cancer	Both	Non-drinkers	Moderate drinkers	0.06	0.02	age, sex, bmi, education, income, physical activity, smoking, menopause, dietary factors, other, family history
Jain	1993	Breast cancer	Both	Non-drinkers	Light drinkers	0.04	0.02	age, sex, bmi, education, income, physical activity, smoking, menopause, dietary factors, other, family history
Jain	1993	Breast cancer	Both	Non-drinkers	Light drinkers	0.01	0.01	age, sex, bmi, education, income, physical activity, smoking, menopause, dietary factors, other, family history
Karavasiloglou	2010	Breast cancer	Female	Moderate drinkers	Light drinkers	0.08	0.09	representativeness, age, sex, bmi, education, income, physical activity, smoking, menopause, other drug use, dietary factors, other, hypertension, diabetes, vitamin deficiency, other diagnosed disease, APOE, family history, hypercholesterolemia
Kinney	1996	Breast cancer	Both	Non-drinkers	Moderate drinkers	0.00	0.25	age, sex, bmi, education, income, physical activity, smoking, other, family history
Kwan	2013	Breast cancer	Female	Light drinkers	Light drinkers	0.04	0.13	representativeness, age, sex, bmi, education, income, physical activity, smoking, menopause, other drug use, dietary factors, other, hypertension, diabetes, vitamin deficiency, other diagnosed disease, APOE, family history, hypercholesterolemia
La	1988	Breast cancer	Both	Non-drinkers	Light drinkers	0.26	0.08	age, sex, bmi, education, income, physical activity, smoking, menopause, other drug use, dietary factors, other, family history
La	1988	Breast cancer	Both	Non-drinkers	Light drinkers	0.26	0.10	age, sex, bmi, education, income, physical activity, smoking, menopause, other drug use, dietary factors, other, family history
Le	1980	Breast cancer	Both	Non-drinkers	Light drinkers	0.04	0.15	age, sex, bmi, education, income, physical activity
Le	1980	Breast cancer	Both	Non-drinkers	Light drinkers	-0.59	0.33	age, sex, bmi, education, income, physical activity
Le	1980	Breast cancer	Both	Non-drinkers	Light drinkers	0.29	0.13	age, sex, bmi, education, income, physical activity
Le	1980	Breast cancer	Both	Non-drinkers	Light drinkers	-0.08	0.26	age, sex, bmi, education, income, physical activity

Table S10. Summary of Data Inputs

Author	Year	Health outcome	Sex	Reference	Exposed group	Natural Log of Effect Size	Standard Error of Natural Log of Effect Size	Covariates Adjusted for in Source
Le	1980	Breast cancer	Both	Non-drinkers	Light drinkers	0.80	0.34	age, sex, bmi, education, income, physical activity
Levi	1995	Breast cancer	Both	Non-drinkers	Light drinkers	0.26	0.22	age, sex, bmi, education, income, physical activity, smoking, menopause, other drug use, other, other diagnosed disease, family history
Li	2005	Breast cancer	Both	Non-drinkers	Light drinkers	0.24	0.10	age, sex, bmi, education, income, physical activity, smoking, menopause, other drug use, other, family history
Li	2005	Breast cancer	Both	Non-drinkers	Light drinkers	0.05	0.10	age, sex, bmi, education, income, physical activity, smoking, menopause, other drug use, other, family history
Li	2005	Breast cancer	Both	Non-drinkers	Light drinkers	0.10	0.08	age, sex, bmi, education, income, physical activity, smoking, menopause, other drug use, other, family history
Li	2005	Breast cancer	Both	Non-drinkers	Light drinkers	0.02	0.08	age, sex, bmi, education, income, physical activity, smoking, menopause, other drug use, other, family history
Li	2004	Breast cancer	Both	Non-drinkers	Light drinkers	0.10	0.07	age, sex, bmi, education, income, physical activity, smoking, other, family history
Li	2004	Breast cancer	Both	Non-drinkers	Light drinkers	0.10	0.05	age, sex, bmi, education, income, physical activity, smoking, other, family history
Li	2004	Breast cancer	Both	Non-drinkers	Light drinkers	0.10	0.07	age, sex, bmi, education, income, physical activity, smoking, other, family history
Li	2004	Breast cancer	Both	Non-drinkers	Light drinkers	0.10	0.05	age, sex, bmi, education, income, physical activity, smoking, other, family history
Li	1999	Breast cancer	Both	Non-drinkers	Light drinkers	0.26	0.19	age, sex, bmi, education, income, physical activity, menopause, family history
Li	1999	Breast cancer	Both	Non-drinkers	Light drinkers	0.10	0.13	age, sex, bmi, education, income, physical activity, menopause, family history
Lucas	1993	Breast cancer	Both	Non-drinkers	Light drinkers	-0.34	1.11	age, sex, bmi, education, income, physical activity, menopause, family history
Lucas	1993	Breast cancer	Both	Non-drinkers	Light drinkers	-0.02	0.57	age, sex, bmi, education, income, physical activity, menopause, family history
McDonald	1998	Breast cancer	Both	Non-drinkers	Light drinkers	0.00	0.05	age, sex, bmi, education, income, physical activity, menopause, other drug use, other, family history
Meara	1984	Breast cancer	Both	Non-drinkers	Light drinkers	0.00	0.25	age, sex, bmi, education, income, physical activity, smoking, menopause, other drug use, other, family history
Meara	1984	Breast cancer	Both	Non-drinkers	Light drinkers	0.00	0.19	age, sex, bmi, education, income, physical activity, smoking, menopause, other drug use, other, family history
Meara	1984	Breast cancer	Both	Non-drinkers	N/A	0.10	0.27	age, sex, bmi, education, income, physical activity, smoking, menopause, other drug use, other, family history
Meara	1984	Breast cancer	Both	Non-drinkers	Light drinkers	0.10	0.63	age, sex, bmi, education, income, physical activity, smoking, menopause, other drug use, other, family history
Meara	1984	Breast cancer	Both	Non-drinkers	Moderate drinkers	0.10	0.25	age, sex, bmi, education, income, physical activity, smoking, menopause, other drug use, other, family history
Meara	1984	Breast cancer	Both	Non-drinkers	Light drinkers	0.10	0.18	age, sex, bmi, education, income, physical activity, smoking, menopause, other drug use, other, family history
Meulepas	2007	Breast cancer	Both	Non-drinkers	N/A	0.15	0.10	age, sex, bmi, education, income, physical activity
Morch	2001	Breast cancer	Both	Light drinkers	Light drinkers	0.24	0.16	age, sex, bmi, education, income, physical activity, other, other diagnosed disease, family history
Morch	2001	Breast cancer	Both	Light drinkers	Light drinkers	0.10	0.16	age, sex, bmi, education, income, physical activity, other, other diagnosed disease, family history

Table S10. Summary of Data Inputs

Author	Year	Health outcome	Sex	Reference	Exposed group	Natural Log of Effect Size	Standard Error of Natural Log of Effect Size	Covariates Adjusted for in Source
Morch	2001	Breast cancer	Both	Light drinkers	Light drinkers	-0.01	0.17	age, sex, bmi, education, income, physical activity, other, other diagnosed disease, family history
Morch	2001	Breast cancer	Both	Light drinkers	Heavy drinkers	0.83	0.20	age, sex, bmi, education, income, physical activity, other, other diagnosed disease, family history
Newcomb	2000	Breast cancer	Both	Non-drinkers	Light drinkers	0.02	0.07	age, sex, bmi, education, income, physical activity, menopause, other drug use, other, family history
Newcomb	2000	Breast cancer	Both	Non-drinkers	Light drinkers	-0.01	0.06	age, sex, bmi, education, income, physical activity, menopause, other drug use, other, family history
Newcomb	2000	Breast cancer	Both	Non-drinkers	Light drinkers	-0.06	0.05	age, sex, bmi, education, income, physical activity, menopause, other drug use, other, family history
Nielsen	2002	Breast cancer	Both	Light drinkers	Light drinkers	0.17	0.14	age, sex, bmi, education, income, physical activity, smoking, menopause, other drug use, other
Ong	2015	Breast cancer	Female	Non-drinkers	Light drinkers	0.09	0.02	representativeness, age, sex, bmi, education, income, physical activity, smoking, menopause, other drug use, dietary factors, other, hypertension, diabetes, vitamin deficiency, other diagnosed disease, APOE, family history, hypercholesterolemia
Ong	2015	Breast cancer	Female	Non-drinkers	Light drinkers	0.07	0.05	representativeness, age, sex, bmi, education, income, physical activity, smoking, menopause, other drug use, dietary factors, other, hypertension, diabetes, vitamin deficiency, other diagnosed disease, APOE, family history, hypercholesterolemia
Ong	2015	Breast cancer	Female	Non-drinkers	Light drinkers	0.04	0.01	representativeness, age, sex, bmi, education, income, physical activity, smoking, menopause, other drug use, dietary factors, other, hypertension, diabetes, vitamin deficiency, other diagnosed disease, APOE, family history, hypercholesterolemia
Richardson	1986	Breast cancer	Both	Light drinkers	Moderate drinkers	0.83	0.24	age, sex, bmi, education, income, physical activity
Rosenberg	1986	Breast cancer	Both	Light drinkers	Moderate drinkers	0.00	0.19	age, sex, bmi, education, income, physical activity, smoking, menopause, other drug use, dietary factors, other, other diagnosed disease, family history
Rosenberg	1986	Breast cancer	Both	Light drinkers	Light drinkers	0.00	0.18	age, sex, bmi, education, income, physical activity, smoking, menopause, other drug use, dietary factors, other, other diagnosed disease, family history
Rosenberg	1986	Breast cancer	Both	Light drinkers	Moderate drinkers	0.00	0.19	age, sex, bmi, education, income, physical activity, smoking, menopause, other drug use, dietary factors, other, other diagnosed disease, family history
Rosenberg	1986	Breast cancer	Both	Light drinkers	Light drinkers	0.00	0.18	age, sex, bmi, education, income, physical activity, smoking, menopause, other drug use, dietary factors, other, other diagnosed disease, family history
Rosenberg	1995	Breast cancer	Both	Non-drinkers	Light drinkers	0.26	0.16	age, sex, bmi, education, income, physical activity, other
Rosenberg	1995	Breast cancer	Both	Non-drinkers	Light drinkers	0.00	0.25	age, sex, bmi, education, income, physical activity, other
Rosenberg	1995	Breast cancer	Both	Non-drinkers	Light drinkers	0.00	0.10	age, sex, bmi, education, income, physical activity, other
Rosenberg	1995	Breast cancer	Both	Non-drinkers	Light drinkers	0.00	0.25	age, sex, bmi, education, income, physical activity, other
Rosenberg	1995	Breast cancer	Both	Non-drinkers	Light drinkers	0.00	0.10	age, sex, bmi, education, income, physical activity, other

Table S10. Summary of Data Inputs

Author	Year	Health outcome	Sex	Reference	Exposed group	Natural Log of Effect Size	Standard Error of Natural Log of Effect Size	Covariates Adjusted for in Source
Rosenberg	1995	Breast cancer	Both	Non-drinkers	Light drinkers	0.10	0.24	age, sex, bmi, education, income, physical activity, other
Rosenberg	1995	Breast cancer	Both	Non-drinkers	Light drinkers	0.10	0.09	age, sex, bmi, education, income, physical activity, other
Rosenberg	1995	Breast cancer	Both	Non-drinkers	Light drinkers	0.10	0.16	age, sex, bmi, education, income, physical activity, other
Rosenberg	1995	Breast cancer	Both	Non-drinkers	Light drinkers	0.10	0.24	age, sex, bmi, education, income, physical activity, other
Rosenberg	1995	Breast cancer	Both	Non-drinkers	Light drinkers	0.10	0.09	age, sex, bmi, education, income, physical activity, other
Rosenberg	1995	Breast cancer	Both	Non-drinkers	Light drinkers	0.10	0.16	age, sex, bmi, education, income, physical activity, other
Rosenberg	1995	Breast cancer	Both	Non-drinkers	Light drinkers	0.10	0.24	age, sex, bmi, education, income, physical activity, other
Rosenberg	1995	Breast cancer	Both	Non-drinkers	Light drinkers	0.10	0.09	age, sex, bmi, education, income, physical activity, other
Rosenberg	1995	Breast cancer	Both	Non-drinkers	Light drinkers	0.10	0.16	age, sex, bmi, education, income, physical activity, other
Rustagi	2013	Breast cancer	Female	Non-drinkers	Light drinkers	0.13	0.06	representativeness, age, sex, bmi, education, income, physical activity, smoking, menopause, other drug use, dietary factors, other, hypertension, diabetes, vitamin deficiency, other diagnosed disease, APOE, family history, hypercholesterolemia
Sarich	2013	Breast cancer	Female	Light drinkers	Light drinkers	-0.09	0.06	representativeness, age, sex, bmi, education, income, physical activity, smoking, menopause, other drug use, dietary factors, other, hypertension, diabetes, vitamin deficiency, other diagnosed disease, APOE, family history, hypercholesterolemia
Sarich	2013	Breast cancer	Female	Light drinkers	Light drinkers	0.02	0.07	representativeness, age, sex, bmi, education, income, physical activity, smoking, menopause, other drug use, dietary factors, other, hypertension, diabetes, vitamin deficiency, other diagnosed disease, APOE, family history, hypercholesterolemia
Simon	1987	Breast cancer	Both	Non-drinkers	Light drinkers	0.08	0.27	age, sex, bmi, education, income, physical activity, smoking, other, family history
Smith	1985	Breast cancer	Both	Non-drinkers	Light drinkers	0.08	0.14	age, sex, bmi, education, income, physical activity, smoking, other drug use, other, other diagnosed disease, family history
Sneyd	1987	Breast cancer	Both	Non-drinkers	Light drinkers	0.10	0.24	age, sex, bmi, education, income, physical activity, smoking, other
Sneyd	1987	Breast cancer	Both	Non-drinkers	Light drinkers	-0.09	0.29	age, sex, bmi, education, income, physical activity, smoking, other
Sneyd	1987	Breast cancer	Both	Non-drinkers	Light drinkers	-0.09	0.26	age, sex, bmi, education, income, physical activity, smoking, other
Sneyd	1987	Breast cancer	Both	Non-drinkers	Light drinkers	-0.09	0.29	age, sex, bmi, education, income, physical activity, smoking, other
Sneyd	1987	Breast cancer	Both	Non-drinkers	Light drinkers	-0.09	0.26	age, sex, bmi, education, income, physical activity, smoking, other
Song	2013	Breast cancer	Female	Non-drinkers	Light drinkers	-0.94	0.58	representativeness, age, sex, bmi, education, income, physical activity, smoking, menopause, other drug use, dietary factors, other, hypertension, diabetes, vitamin deficiency, other diagnosed disease, APOE, family history, hypercholesterolemia

Table S10. Summary of Data Inputs

Author	Year	Health outcome	Sex	Reference	Exposed group	Natural Log of Effect Size	Standard Error of Natural Log of Effect Size	Covariates Adjusted for in Source
Suzuki	2006	Breast cancer	Both	Non-drinkers	Light drinkers	0.06	0.16	age, sex, bmi, education, income, physical activity, smoking, menopause, other drug use, dietary factors, other
Suzuki	2004	Breast cancer	Both	Non-drinkers	Light drinkers	0.08	0.07	age, sex, bmi, education, income, physical activity, menopause, other drug use, dietary factors, other, other diagnosed disease, family history
Suzuki	2004	Breast cancer	Both	Non-drinkers	Light drinkers	0.10	0.08	age, sex, bmi, education, income, physical activity, menopause, other drug use, dietary factors, other, other diagnosed disease, family history
Thun	1991	Breast cancer	Both	Non-drinkers	Light drinkers	0.10	0.09	age, sex, bmi, education, income, physical activity, smoking
Thun	1991	Breast cancer	Both	Non-drinkers	Heavy drinkers	0.00	0.18	age, sex, bmi, education, income, physical activity, smoking
Tjonneland	2004	Breast cancer	Both	Light drinkers	Heavy drinkers	0.09	0.08	age, sex, bmi, education, income, physical activity, smoking, menopause, other drug use, other
Tjonneland	2004	Breast cancer	Both	Light drinkers	Light drinkers	0.01	0.06	age, sex, bmi, education, income, physical activity, smoking, menopause, other drug use, other
Tjonneland	2004	Breast cancer	Both	Light drinkers	Light drinkers	-0.02	0.05	age, sex, bmi, education, income, physical activity, smoking, menopause, other drug use, other
Tjonneland	2004	Breast cancer	Both	Light drinkers	Moderate drinkers	0.08	0.08	age, sex, bmi, education, income, physical activity, smoking, menopause, other drug use, other
Tjonneland	2004	Breast cancer	Both	Light drinkers	Light drinkers	-0.03	0.05	age, sex, bmi, education, income, physical activity, smoking, menopause, other drug use, other
Tjonneland	2004	Breast cancer	Both	Light drinkers	Moderate drinkers	0.03	0.09	age, sex, bmi, education, income, physical activity, smoking, menopause, other drug use, other
Tjonneland	2004	Breast cancer	Both	Light drinkers	Light drinkers	0.07	0.05	age, sex, bmi, education, income, physical activity, smoking, menopause, other drug use, other
Toniolo	1984	Breast cancer	Both	Non-drinkers	Moderate drinkers	0.00	0.21	age, sex, bmi, education, income, physical activity, menopause, dietary factors
Viladiu	1989	Breast cancer	Both	Non-drinkers	Light drinkers	0.26	0.23	age, sex, bmi, education, income, physical activity, menopause, other, family history
Viladiu	1989	Breast cancer	Both	Non-drinkers	Light drinkers	-0.92	0.18	age, sex, bmi, education, income, physical activity, menopause, other, family history
Wang	2004	Breast cancer	Both	Non-drinkers	Light drinkers	0.02	0.14	age, sex, bmi, education, income, physical activity
Zeinomar	2019	Breast cancer	Female	Non-drinkers	Light drinkers	0.06	0.23	representativeness, age, sex, bmi, education, income, physical activity, smoking, menopause, other drug use, dietary factors, other, hypertension, diabetes, vitamin deficiency, other diagnosed disease, APOE, family history, hypercholesterolemia
Zhang	2005	Breast cancer	Both	Non-drinkers	Light drinkers	-0.54	0.26	age, sex, bmi, education, income, physical activity, menopause, other drug use, dietary factors, family history
Zhang	2005	Breast cancer	Both	Non-drinkers	Light drinkers	-0.58	0.11	age, sex, bmi, education, income, physical activity, menopause, other drug use, dietary factors, family history
van den Brandt	1989	Breast cancer	Both	Non-drinkers	Light drinkers	0.09	0.21	age, sex, bmi, education, income, physical activity, smoking, menopause, other drug use, dietary factors, other, other diagnosed disease, family history
van't Veer	1987	Breast cancer	Both	Non-drinkers	Light drinkers	0.00	0.63	age, sex, bmi, education, income, physical activity, smoking, menopause, dietary factors, other, family history
van't Veer	1987	Breast cancer	Both	Non-drinkers	Moderate drinkers	0.10	0.68	age, sex, bmi, education, income, physical activity, smoking, menopause, dietary factors, other, family history

Table S10. Summary of Data Inputs

Author	Year	Health outcome	Sex	Reference	Exposed group	Natural Log of Effect Size	Standard Error of Natural Log of Effect Size	Covariates Adjusted for in Source
van't Veer	1987	Breast cancer	Both	Non-drinkers	Moderate drinkers	0.83	1.06	age, sex, bmi, education, income, physical activity, smoking, menopause, dietary factors, other, family history
Becker	1992	Cirrhosis and other chronic liver diseases	Both	Light drinkers	Moderate drinkers	0.85	0.35	age, sex, bmi, education, income, physical activity, smoking, other
Becker	1998	Cirrhosis and other chronic liver diseases	Both	Light drinkers	Light drinkers	0.12	0.59	age, sex, bmi, education, income, physical activity
Becker	1998	Cirrhosis and other chronic liver diseases	Both	Light drinkers	Moderate drinkers	-0.02	0.32	age, sex, bmi, education, income, physical activity
Fuchs	1992	Cirrhosis and other chronic liver diseases	Female	Non-drinkers	Light drinkers	-0.37	0.54	representativeness, age, sex, bmi, education, income, physical activity, smoking, menopause, other drug use, dietary factors, other, hypertension, diabetes, vitamin deficiency, other diagnosed disease, APOE, family history, hypercholesterolemia
Fuchs	1992	Cirrhosis and other chronic liver diseases	Female	Non-drinkers	Light drinkers	0.24	0.44	representativeness, age, sex, bmi, education, income, physical activity, smoking, menopause, other drug use, dietary factors, other, hypertension, diabetes, vitamin deficiency, other diagnosed disease, APOE, family history, hypercholesterolemia
Fuchs	1992	Cirrhosis and other chronic liver diseases	Female	Non-drinkers	Moderate drinkers	0.62	0.46	representativeness, age, sex, bmi, education, income, physical activity, smoking, menopause, other drug use, dietary factors, other, hypertension, diabetes, vitamin deficiency, other diagnosed disease, APOE, family history, hypercholesterolemia
Setiawan	2013	Cirrhosis and other chronic liver diseases	Both	Non-drinkers	Light drinkers	0.07	0.23	representativeness, age, sex, bmi, education, income, physical activity, smoking, menopause, other drug use, dietary factors, other, hypertension, diabetes, vitamin deficiency, other diagnosed disease, APOE, family history, hypercholesterolemia
Tao	2018	Cirrhosis and other chronic liver diseases	Both	Non-drinkers	Moderate drinkers	0.04	0.14	representativeness, age, sex, bmi, education, income, physical activity, smoking, menopause, other drug use, dietary factors, other, hypertension, diabetes, vitamin deficiency, other diagnosed disease, APOE, family history, hypercholesterolemia
Choi	1990	Colon and rectum cancer	Male	Non-drinkers	Moderate drinkers	0.09	0.44	representativeness, age, sex, bmi, education, income, physical activity, smoking, menopause, other drug use, dietary factors, other, hypertension, diabetes, vitamin deficiency, other diagnosed disease, APOE, family history, hypercholesterolemia
Choi	1990	Colon and rectum cancer	Male	Non-drinkers	Heavy drinkers	-0.01	0.43	representativeness, age, sex, bmi, education, income, physical activity, smoking, menopause, other drug use, dietary factors, other, hypertension, diabetes, vitamin deficiency, other diagnosed disease, APOE, family history, hypercholesterolemia
Choi	1990	Colon and rectum cancer	Male	Non-drinkers	Moderate drinkers	0.69	0.46	representativeness, age, sex, bmi, education, income, physical activity, smoking, menopause, other drug use, dietary factors, other, hypertension, diabetes, vitamin deficiency, other diagnosed disease, APOE, family history, hypercholesterolemia
Choi	1990	Colon and rectum cancer	Male	Non-drinkers	Heavy drinkers	0.90	0.42	representativeness, age, sex, bmi, education, income, physical activity, smoking, menopause, other drug use, dietary factors, other, hypertension, diabetes, vitamin deficiency, other diagnosed disease, APOE, family history, hypercholesterolemia

Table S10. Summary of Data Inputs

Author	Year	Health outcome	Sex	Reference	Exposed group	Natural Log of Effect Size	Standard Error of Natural Log of Effect Size	Covariates Adjusted for in Source
Choi	1990	Colon and rectum cancer	Male	Non-drinkers	Heavy drinkers	1.56	0.19	representativeness, age, sex, bmi, education, income, physical activity, smoking, menopause, other drug use, dietary factors, other, hypertension, diabetes, vitamin deficiency, other diagnosed disease, APOE, family history, hypercholesterolemia
Chyou	1995	Colon and rectum cancer	Both	Non-drinkers	Light drinkers	0.15	0.07	age, sex, bmi, education, income, physical activity
Crockett	2006	Colon and rectum cancer	Both	Non-drinkers	Light drinkers	-0.07	0.14	age, sex, bmi, education, income, physical activity, smoking, dietary factors, other, family history
Flood	1998	Colon and rectum cancer	Both	Non-drinkers	Moderate drinkers	0.07	0.36	age, sex, bmi, education, income, physical activity, smoking, other drug use, dietary factors, other
Flood	1998	Colon and rectum cancer	Both	Non-drinkers	Light drinkers	-0.04	0.17	age, sex, bmi, education, income, physical activity, smoking, other drug use, dietary factors, other
Floud	2001	Colon and rectum cancer	Female	Light drinkers	Light drinkers	0.05	0.01	representativeness, age, sex, bmi, education, income, physical activity, smoking, menopause, other drug use, dietary factors, other, hypertension, diabetes, vitamin deficiency, other diagnosed disease, APOE, family history, hypercholesterolemia
Floud	2001	Colon and rectum cancer	Female	Light drinkers	Moderate drinkers	0.24	0.02	representativeness, age, sex, bmi, education, income, physical activity, smoking, menopause, other drug use, dietary factors, other, hypertension, diabetes, vitamin deficiency, other diagnosed disease, APOE, family history, hypercholesterolemia
Goldbohm	1989	Colon and rectum cancer	Both	Non-drinkers	Light drinkers	0.00	0.23	age, sex, bmi, education, income, physical activity, smoking, dietary factors, other
Goldbohm	1989	Colon and rectum cancer	Both	Non-drinkers	Moderate drinkers	0.24	0.37	age, sex, bmi, education, income, physical activity, smoking, dietary factors, other
Goldbohm	1989	Colon and rectum cancer	Both	Non-drinkers	Moderate drinkers	0.05	0.22	age, sex, bmi, education, income, physical activity, smoking, dietary factors, other
Goldbohm	1989	Colon and rectum cancer	Both	Non-drinkers	Moderate drinkers	0.01	0.27	age, sex, bmi, education, income, physical activity, smoking, dietary factors, other
Goldbohm	1989	Colon and rectum cancer	Both	Non-drinkers	Light drinkers	-0.34	0.18	age, sex, bmi, education, income, physical activity, smoking, dietary factors, other
Hatime	2020	Colon and rectum cancer	Both	Light drinkers	Light drinkers	0.00	0.63	representativeness, age, sex, bmi, education, income, physical activity, smoking, menopause, other drug use, dietary factors, other, hypertension, diabetes, vitamin deficiency, other diagnosed disease, APOE, family history, hypercholesterolemia
Hur	2021	Colon and rectum cancer	Female	Non-drinkers	Light drinkers	0.03	0.15	representativeness, age, sex, bmi, education, income, physical activity, smoking, menopause, other drug use, dietary factors, other, hypertension, diabetes, vitamin deficiency, other diagnosed disease, APOE, family history, hypercholesterolemia
Hur	2021	Colon and rectum cancer	Female	Non-drinkers	Light drinkers	-0.01	0.08	representativeness, age, sex, bmi, education, income, physical activity, smoking, menopause, other drug use, dietary factors, other, hypertension, diabetes, vitamin deficiency, other diagnosed disease, APOE, family history, hypercholesterolemia
Hur	2021	Colon and rectum cancer	Female	Non-drinkers	Light drinkers	0.08	0.10	representativeness, age, sex, bmi, education, income, physical activity, smoking, menopause, other drug use, dietary factors, other, hypertension, diabetes, vitamin deficiency, other diagnosed disease, APOE, family history, hypercholesterolemia
Kabat	2000	Colon and rectum cancer	Both	Non-drinkers	Moderate drinkers	0.02	0.18	age, sex, bmi, education, income, physical activity, smoking, menopause, other drug use, dietary factors

Table S10. Summary of Data Inputs

Author	Year	Health outcome	Sex	Reference	Exposed group	Natural Log of Effect Size	Standard Error of Natural Log of Effect Size	Covariates Adjusted for in Source
Kabat	2000	Colon and rectum cancer	Both	Non-drinkers	Light drinkers	0.04	0.14	age, sex, bmi, education, income, physical activity, smoking, menopause, other drug use, dietary factors
Kabat	2000	Colon and rectum cancer	Both	Non-drinkers	Light drinkers	-0.08	0.11	age, sex, bmi, education, income, physical activity, smoking, menopause, other drug use, dietary factors
Kabat	2000	Colon and rectum cancer	Both	Non-drinkers	Light drinkers	-0.07	0.14	age, sex, bmi, education, income, physical activity, smoking, menopause, other drug use, dietary factors
Kono	1983	Colon and rectum cancer	Both	Non-drinkers	Moderate drinkers	0.09	0.46	age, sex, bmi, education, income, physical activity, smoking
Kono	1983	Colon and rectum cancer	Both	Non-drinkers	Light drinkers	0.10	0.51	age, sex, bmi, education, income, physical activity, smoking
Lightfoot	2000	Colon and rectum cancer	Both	Non-drinkers	Light drinkers	0.09	0.17	age, sex, bmi, education, income, physical activity
Murata	1997	Colon and rectum cancer	Both	Non-drinkers	Moderate drinkers	-0.16	0.22	age, sex, bmi, education, income, physical activity
Nakaya	1997	Colon and rectum cancer	Both	Non-drinkers	Light drinkers	0.26	0.39	age, sex, bmi, education, income, physical activity, smoking, dietary factors
Newcomb	1991	Colon and rectum cancer	Both	Non-drinkers	Light drinkers	0.05	0.18	age, sex, bmi, education, income, physical activity, family history
Newcomb	1991	Colon and rectum cancer	Both	Non-drinkers	Light drinkers	0.06	0.15	age, sex, bmi, education, income, physical activity, family history
Newcomb	1991	Colon and rectum cancer	Both	Non-drinkers	Light drinkers	-0.03	0.12	age, sex, bmi, education, income, physical activity, family history
Pedersen	1993	Colon and rectum cancer	Both	Light drinkers	Moderate drinkers	0.03	0.07	age, sex, bmi, education, income, physical activity, other
Pedersen	1993	Colon and rectum cancer	Both	Light drinkers	Heavy drinkers	0.00	0.18	age, sex, bmi, education, income, physical activity, other
Pedersen	1993	Colon and rectum cancer	Both	Light drinkers	Light drinkers	-0.04	0.05	age, sex, bmi, education, income, physical activity, other
Pedersen	1993	Colon and rectum cancer	Both	Light drinkers	Light drinkers	-0.04	0.07	age, sex, bmi, education, income, physical activity, other
Peters	1982	Colon and rectum cancer	Both	Light drinkers	Moderate drinkers	0.00	0.33	age, sex, bmi, education, income, physical activity
Sarich	2013	Colon and rectum cancer	Both	Light drinkers	Light drinkers	0.03	0.07	representativeness, age, sex, bmi, education, income, physical activity, smoking, menopause, other drug use, dietary factors, other, hypertension, diabetes, vitamin deficiency, other diagnosed disease, APOE, family history, hypercholesterolemia
Sarich	2013	Colon and rectum cancer	Both	Light drinkers	Light drinkers	-0.03	0.08	representativeness, age, sex, bmi, education, income, physical activity, smoking, menopause, other drug use, dietary factors, other, hypertension, diabetes, vitamin deficiency, other diagnosed disease, APOE, family history, hypercholesterolemia
Sarich	2013	Colon and rectum cancer	Both	Light drinkers	Light drinkers	0.13	0.08	representativeness, age, sex, bmi, education, income, physical activity, smoking, menopause, other drug use, dietary factors, other, hypertension, diabetes, vitamin deficiency, other diagnosed disease, APOE, family history, hypercholesterolemia
Sarich	2013	Colon and rectum cancer	Both	Light drinkers	Heavy drinkers	0.26	0.12	representativeness, age, sex, bmi, education, income, physical activity, smoking, menopause, other drug use, dietary factors, other, hypertension, diabetes, vitamin deficiency, other diagnosed disease, APOE, family history, hypercholesterolemia

Table S10. Summary of Data Inputs

Author	Year	Health outcome	Sex	Reference	Exposed group	Natural Log of Effect Size	Standard Error of Natural Log of Effect Size	Covariates Adjusted for in Source
Seol	2004	Colon and rectum cancer	Both	Non-drinkers	Light drinkers	0.10	0.14	representativeness, age, sex, bmi, education, income, physical activity, smoking, menopause, other drug use, dietary factors, other, hypertension, diabetes, vitamin deficiency, other diagnosed disease, APOE, family history, hypercholesterolemia
Sharpe	1985	Colon and rectum cancer	Both	Non-drinkers	Moderate drinkers	0.37	0.22	age, sex, bmi, education, income, physical activity, smoking, other
Stern	2002	Colon and rectum cancer	Both	Non-drinkers	Light drinkers	0.05	0.19	age, sex, bmi, education, income, physical activity
Stern	2002	Colon and rectum cancer	Both	Non-drinkers	Light drinkers	0.79	0.26	age, sex, bmi, education, income, physical activity
Tsong	2004	Colon and rectum cancer	Both	Non-drinkers	Light drinkers	-0.04	0.14	age, sex, bmi, education, income, physical activity, smoking, menopause, other, diabetes, family history
Tverdal	2018	Colon and rectum cancer	Male	Non-drinkers	Light drinkers	0.13	0.07	representativeness, age, sex, bmi, education, income, physical activity, smoking, menopause, other drug use, dietary factors, other, hypertension, diabetes, vitamin deficiency, other diagnosed disease, APOE, family history, hypercholesterolemia
Tverdal	2018	Colon and rectum cancer	Female	Non-drinkers	Light drinkers	0.05	0.06	representativeness, age, sex, bmi, education, income, physical activity, smoking, menopause, other drug use, dietary factors, other, hypertension, diabetes, vitamin deficiency, other diagnosed disease, APOE, family history, hypercholesterolemia
Tverdal	2018	Colon and rectum cancer	Male	Non-drinkers	Light drinkers	0.37	0.13	representativeness, age, sex, bmi, education, income, physical activity, smoking, menopause, other drug use, dietary factors, other, hypertension, diabetes, vitamin deficiency, other diagnosed disease, APOE, family history, hypercholesterolemia
Wang	2004	Colon and rectum cancer	Both	Non-drinkers	Light drinkers	0.29	0.15	age, sex, bmi, education, income, physical activity
Wang	2004	Colon and rectum cancer	Both	Non-drinkers	Light drinkers	0.07	0.15	age, sex, bmi, education, income, physical activity
Wang	2004	Colon and rectum cancer	Both	Non-drinkers	Light drinkers	-0.08	0.15	age, sex, bmi, education, income, physical activity
Wu	1985	Colon and rectum cancer	Both	Non-drinkers	Moderate drinkers	0.37	0.30	age, sex, bmi, education, income, physical activity
Wu	1985	Colon and rectum cancer	Both	Non-drinkers	Moderate drinkers	0.88	0.32	age, sex, bmi, education, income, physical activity
Yamada	1993	Colon and rectum cancer	Both	Non-drinkers	Light drinkers	0.10	0.52	age, sex, bmi, education, income, physical activity, smoking
Yi	2005	Colon and rectum cancer	Both	Non-drinkers	Heavy drinkers	0.96	0.56	age, sex, bmi, education, income, physical activity, smoking, other
Baik	2012	Diabetes mellitus type 2	Both	Non-drinkers	Light drinkers	0.07	0.24	representativeness, age, sex, bmi, education, income, physical activity, smoking, menopause, other drug use, dietary factors, other, hypertension, diabetes, vitamin deficiency, other diagnosed disease, APOE, family history, hypercholesterolemia
Beulens	2000	Diabetes mellitus type 2	Male	Light drinkers	Light drinkers	-0.06	0.07	representativeness, exposure_quality, outcome_quality, confounder_quality, reverse_causation, selection_bias, sex
Beulens	2000	Diabetes mellitus type 2	Male	Light drinkers	Heavy drinkers	0.07	0.17	representativeness, exposure_quality, outcome_quality, confounder_quality, reverse_causation, selection_bias, sex

Table S10. Summary of Data Inputs

Author	Year	Health outcome	Sex	Reference	Exposed group	Natural Log of Effect Size	Standard Error of Natural Log of Effect Size	Covariates Adjusted for in Source
Beulens	2000	Diabetes mellitus type 2	Male	Light drinkers	Light drinkers	-0.06	0.07	representativeness, exposure_quality, outcome_quality, confounder_quality, reverse_causation, selection_bias, sex
Beulens	2000	Diabetes mellitus type 2	Male	Light drinkers	Heavy drinkers	0.07	0.17	representativeness, exposure_quality, outcome_quality, confounder_quality, reverse_causation, selection_bias, sex
Beulens	2000	Diabetes mellitus type 2	Male	Light drinkers	Light drinkers	-0.06	0.07	representativeness, exposure_quality, outcome_quality, confounder_quality, reverse_causation, selection_bias, sex
Beulens	2000	Diabetes mellitus type 2	Male	Light drinkers	Heavy drinkers	0.07	0.17	representativeness, exposure_quality, outcome_quality, confounder_quality, reverse_causation, selection_bias, sex
Beulens	2000	Diabetes mellitus type 2	Male	Light drinkers	Light drinkers	-0.06	0.07	representativeness, exposure_quality, outcome_quality, confounder_quality, reverse_causation, selection_bias, sex
Beulens	2000	Diabetes mellitus type 2	Male	Light drinkers	Heavy drinkers	0.07	0.17	representativeness, exposure_quality, outcome_quality, confounder_quality, reverse_causation, selection_bias, sex
Beulens	2000	Diabetes mellitus type 2	Male	Light drinkers	Light drinkers	-0.06	0.07	representativeness, exposure_quality, outcome_quality, confounder_quality, reverse_causation, selection_bias, sex
Beulens	2000	Diabetes mellitus type 2	Male	Light drinkers	Heavy drinkers	0.07	0.17	representativeness, exposure_quality, outcome_quality, confounder_quality, reverse_causation, selection_bias, sex
Beulens	2000	Diabetes mellitus type 2	Male	Light drinkers	Light drinkers	-0.06	0.07	representativeness, exposure_quality, outcome_quality, confounder_quality, reverse_causation, selection_bias, sex
Beulens	2000	Diabetes mellitus type 2	Male	Light drinkers	Heavy drinkers	0.07	0.17	representativeness, exposure_quality, outcome_quality, confounder_quality, reverse_causation, selection_bias, sex
Beulens	2000	Diabetes mellitus type 2	Male	Light drinkers	Light drinkers	-0.06	0.07	representativeness, exposure_quality, outcome_quality, confounder_quality, reverse_causation, selection_bias, sex
Beulens	2000	Diabetes mellitus type 2	Male	Light drinkers	Heavy drinkers	0.07	0.17	representativeness, exposure_quality, outcome_quality, confounder_quality, reverse_causation, selection_bias, sex
Beulens	2000	Diabetes mellitus type 2	Male	Light drinkers	Light drinkers	-0.06	0.07	representativeness, exposure_quality, outcome_quality, confounder_quality, reverse_causation, selection_bias, sex
Beulens	2000	Diabetes mellitus type 2	Male	Light drinkers	Heavy drinkers	0.07	0.17	representativeness, exposure_quality, outcome_quality, confounder_quality, reverse_causation, selection_bias, sex
Beulens	2002	Diabetes mellitus type 2	Female	Non-drinkers	Light drinkers	0.04	0.13	representativeness, age, sex, bmi, education, income, physical activity, smoking, menopause, other drug use, dietary factors, other, hypertension, diabetes, vitamin deficiency, other diagnosed disease, APOE, family history, hypercholesterolemia

Table S10. Summary of Data Inputs

Author	Year	Health outcome	Sex	Reference	Exposed group	Natural Log of Effect Size	Standard Error of Natural Log of Effect Size	Covariates Adjusted for in Source
Choi	2008	Diabetes mellitus type 2	Both	Non-drinkers	Light drinkers	0.10	0.02	representativeness, age, sex, bmi, education, income, physical activity, smoking, menopause, other drug use, dietary factors, other, hypertension, diabetes, vitamin deficiency, other diagnosed disease, APOE, family history, hypercholesterolemia
Choi	2008	Diabetes mellitus type 2	Both	Non-drinkers	Light drinkers	-0.02	0.02	representativeness, age, sex, bmi, education, income, physical activity, smoking, menopause, other drug use, dietary factors, other, hypertension, diabetes, vitamin deficiency, other diagnosed disease, APOE, family history, hypercholesterolemia
Choi	2008	Diabetes mellitus type 2	Both	Non-drinkers	Light drinkers	-0.02	0.08	representativeness, age, sex, bmi, education, income, physical activity, smoking, menopause, other drug use, dietary factors, other, hypertension, diabetes, vitamin deficiency, other diagnosed disease, APOE, family history, hypercholesterolemia
Freisling	2007	Diabetes mellitus type 2	Both	Light drinkers	Light drinkers	0.00	0.01	representativeness, age, sex, bmi, education, income, physical activity, smoking, menopause, other drug use, dietary factors, other, hypertension, diabetes, vitamin deficiency, other diagnosed disease, APOE, family history, hypercholesterolemia
He	2013	Diabetes mellitus type 2	Female	Light drinkers	Light drinkers	-0.02	0.07	representativeness, age, sex, bmi, education, income, physical activity, smoking, menopause, other drug use, dietary factors, other, hypertension, diabetes, vitamin deficiency, other diagnosed disease, APOE, family history, hypercholesterolemia
He	2013	Diabetes mellitus type 2	Female	Light drinkers	Moderate drinkers	-0.09	0.20	representativeness, age, sex, bmi, education, income, physical activity, smoking, menopause, other drug use, dietary factors, other, hypertension, diabetes, vitamin deficiency, other diagnosed disease, APOE, family history, hypercholesterolemia
He	2013	Diabetes mellitus type 2	Male	Light drinkers	Light drinkers	-0.04	0.09	representativeness, age, sex, bmi, education, income, physical activity, smoking, menopause, other drug use, dietary factors, other, hypertension, diabetes, vitamin deficiency, other diagnosed disease, APOE, family history, hypercholesterolemia
He	2013	Diabetes mellitus type 2	Male	Light drinkers	Light drinkers	-0.07	0.07	representativeness, age, sex, bmi, education, income, physical activity, smoking, menopause, other drug use, dietary factors, other, hypertension, diabetes, vitamin deficiency, other diagnosed disease, APOE, family history, hypercholesterolemia
Hinkle	2017	Diabetes mellitus type 2	Female	Non-drinkers	Light drinkers	0.02	0.09	representativeness, age, sex, bmi, education, income, physical activity, smoking, menopause, other drug use, dietary factors, other, hypertension, diabetes, vitamin deficiency, other diagnosed disease, APOE, family history, hypercholesterolemia
Hinkle	2017	Diabetes mellitus type 2	Female	Non-drinkers	Light drinkers	-0.53	0.17	representativeness, age, sex, bmi, education, income, physical activity, smoking, menopause, other drug use, dietary factors, other, hypertension, diabetes, vitamin deficiency, other diagnosed disease, APOE, family history, hypercholesterolemia
Joosten	2006	Diabetes mellitus type 2	Female	Non-drinkers	Light drinkers	-0.62	0.14	representativeness, exposure_quality, outcome_quality, confounder_quality, reverse_causation, selection_bias, sex
Joosten	2006	Diabetes mellitus type 2	Both	Non-drinkers	Light drinkers	-0.62	0.14	representativeness, age, sex, bmi, education, income, physical activity, smoking, menopause, other drug use, dietary factors, other, hypertension, diabetes, vitamin deficiency, other diagnosed disease, APOE, family history, hypercholesterolemia

Table S10. Summary of Data Inputs

Author	Year	Health outcome	Sex	Reference	Exposed group	Natural Log of Effect Size	Standard Error of Natural Log of Effect Size	Covariates Adjusted for in Source
Kao	1998	Diabetes mellitus type 2	Female	Light drinkers	Light drinkers	-0.02	0.17	representativeness, age, sex, bmi, education, income, physical activity, smoking, menopause, other drug use, dietary factors, other, hypertension, diabetes, vitamin deficiency, other diagnosed disease, APOE, family history, hypercholesterolemia
Kao	1998	Diabetes mellitus type 2	Female	Light drinkers	Moderate drinkers	-0.62	0.50	representativeness, age, sex, bmi, education, income, physical activity, smoking, menopause, other drug use, dietary factors, other, hypertension, diabetes, vitamin deficiency, other diagnosed disease, APOE, family history, hypercholesterolemia
Kao	1998	Diabetes mellitus type 2	Male	Light drinkers	Moderate drinkers	-0.09	0.25	representativeness, age, sex, bmi, education, income, physical activity, smoking, menopause, other drug use, dietary factors, other, hypertension, diabetes, vitamin deficiency, other diagnosed disease, APOE, family history, hypercholesterolemia
Li	2012	Diabetes mellitus type 2	Both	Non-drinkers	Light drinkers	-0.34	0.15	representativeness, age, sex, bmi, education, income, physical activity, smoking, menopause, other drug use, dietary factors, other, hypertension, diabetes, vitamin deficiency, other diagnosed disease, APOE, family history, hypercholesterolemia
Li	2012	Diabetes mellitus type 2	Both	Non-drinkers	Light drinkers	-0.02	0.07	representativeness, age, sex, bmi, education, income, physical activity, smoking, menopause, other drug use, dietary factors, other, hypertension, diabetes, vitamin deficiency, other diagnosed disease, APOE, family history, hypercholesterolemia
Li	2015	Diabetes mellitus type 2	Both	Non-drinkers	Heavy drinkers	-0.03	0.18	representativeness, age, sex, bmi, education, income, physical activity, smoking, menopause, other drug use, dietary factors, other, hypertension, diabetes, vitamin deficiency, other diagnosed disease, APOE, family history, hypercholesterolemia
Lu	2008	Diabetes mellitus type 2	Both	Non-drinkers	Light drinkers	-0.07	0.01	representativeness, age, sex, bmi, education, income, physical activity, smoking, menopause, other drug use, dietary factors, other, hypertension, diabetes, vitamin deficiency, other diagnosed disease, APOE, family history, hypercholesterolemia
Lu	2008	Diabetes mellitus type 2	Both	Non-drinkers	Light drinkers	-0.08	0.01	representativeness, age, sex, bmi, education, income, physical activity, smoking, menopause, other drug use, dietary factors, other, hypertension, diabetes, vitamin deficiency, other diagnosed disease, APOE, family history, hypercholesterolemia
Lu	2008	Diabetes mellitus type 2	Both	Non-drinkers	Light drinkers	-0.09	0.01	representativeness, age, sex, bmi, education, income, physical activity, smoking, menopause, other drug use, dietary factors, other, hypertension, diabetes, vitamin deficiency, other diagnosed disease, APOE, family history, hypercholesterolemia
Lv	2013	Diabetes mellitus type 2	Both	Non-drinkers	Light drinkers	-0.34	0.16	representativeness, age, sex, bmi, education, income, physical activity, smoking, menopause, other drug use, dietary factors, other, hypertension, diabetes, vitamin deficiency, other diagnosed disease, APOE, family history, hypercholesterolemia
Maty	1999	Diabetes mellitus type 2	Both	Non-drinkers	Light drinkers	0.00	0.19	representativeness, exposure_quality, outcome_quality, confounder_quality, reverse_causation, selection_bias, sex
Maty	1999	Diabetes mellitus type 2	Both	Light drinkers	Light drinkers	0.00	0.18	representativeness, exposure_quality, outcome_quality, confounder_quality, reverse_causation, selection_bias, sex

Table S10. Summary of Data Inputs

Author	Year	Health outcome	Sex	Reference	Exposed group	Natural Log of Effect Size	Standard Error of Natural Log of Effect Size	Covariates Adjusted for in Source
Mejldal	2020	Diabetes mellitus type 2	Both	Light drinkers	Heavy drinkers	-0.53	0.23	representativeness, age, sex, bmi, education, income, physical activity, smoking, menopause, other drug use, dietary factors, other, hypertension, diabetes, vitamin deficiency, other diagnosed disease, APOE, family history, hypercholesterolemia
Nagaya	2001	Diabetes mellitus type 2	Male	Non-drinkers	Moderate drinkers	0.00	0.09	representativeness, exposure_quality, outcome_quality, confounder_quality, reverse_causation, selection_bias, sex
Nakanishi	2001	Diabetes mellitus type 2	Male	Moderate drinkers	Light drinkers	0.41	0.18	representativeness, age, sex, bmi, education, income, physical activity, smoking, menopause, other drug use, dietary factors, other, hypertension, diabetes, vitamin deficiency, other diagnosed disease, APOE, family history, hypercholesterolemia
Okamura	2016	Diabetes mellitus type 2	Male	Non-drinkers	Light drinkers	0.06	0.13	representativeness, age, sex, bmi, education, income, physical activity, smoking, menopause, other drug use, dietary factors, other, hypertension, diabetes, vitamin deficiency, other diagnosed disease, APOE, family history, hypercholesterolemia
Strodl	1999	Diabetes mellitus type 2	Female	Non-drinkers	Light drinkers	0.00	0.16	representativeness, exposure_quality, outcome_quality, confounder_quality, reverse_causation, selection_bias, sex
Waki	2000	Diabetes mellitus type 2	Male	Non-drinkers	Light drinkers	0.08	0.11	representativeness, exposure_quality, outcome_quality, confounder_quality, reverse_causation, selection_bias, sex
Waki	2000	Diabetes mellitus type 2	Male	Non-drinkers	Light drinkers	0.08	0.11	representativeness, age, sex, bmi, education, income, physical activity, smoking, menopause, other drug use, dietary factors, other, hypertension, diabetes, vitamin deficiency, other diagnosed disease, APOE, family history, hypercholesterolemia
Wändell	2004	Diabetes mellitus type 2	Male	Non-drinkers	Light drinkers	-0.16	0.71	representativeness, age, sex, bmi, education, income, physical activity, smoking, menopause, other drug use, dietary factors, other, hypertension, diabetes, vitamin deficiency, other diagnosed disease, APOE, family history, hypercholesterolemia
Wändell	2004	Diabetes mellitus type 2	Female	Non-drinkers	Light drinkers	-0.37	0.65	representativeness, age, sex, bmi, education, income, physical activity, smoking, menopause, other drug use, dietary factors, other, hypertension, diabetes, vitamin deficiency, other diagnosed disease, APOE, family history, hypercholesterolemia
Wändell	2004	Diabetes mellitus type 2	Female	Non-drinkers	Moderate drinkers	0.38	0.77	representativeness, age, sex, bmi, education, income, physical activity, smoking, menopause, other drug use, dietary factors, other, hypertension, diabetes, vitamin deficiency, other diagnosed disease, APOE, family history, hypercholesterolemia
Almodova	2012	Esophageal cancer	Both	Non-drinkers	Light drinkers	-0.02	0.65	representativeness, age, sex, bmi, education, income, physical activity, smoking, menopause, other drug use, dietary factors, other, hypertension, diabetes, vitamin deficiency, other diagnosed disease, APOE, family history, hypercholesterolemia
Chen	2006	Esophageal cancer	Both	Non-drinkers	Heavy drinkers	0.07	0.15	representativeness, age, sex, bmi, education, income, physical activity, smoking, menopause, other drug use, dietary factors, other, hypertension, diabetes, vitamin deficiency, other diagnosed disease, APOE, family history, hypercholesterolemia

Table S10. Summary of Data Inputs

Author	Year	Health outcome	Sex	Reference	Exposed group	Natural Log of Effect Size	Standard Error of Natural Log of Effect Size	Covariates Adjusted for in Source
Choi	2016	Esophageal cancer	Both	Non-drinkers	Light drinkers	0.42	0.03	representativeness, age, sex, bmi, education, income, physical activity, smoking, menopause, other drug use, dietary factors, other, hypertension, diabetes, vitamin deficiency, other diagnosed disease, APOE, family history, hypercholesterolemia
De Stefani	2004	Esophageal cancer	Both	Non-drinkers	Moderate drinkers	0.03	0.20	age, sex, bmi, education, income, physical activity
De Stefani	2004	Esophageal cancer	Both	Non-drinkers	Heavy drinkers	0.92	0.23	age, sex, bmi, education, income, physical activity
Freedman	2000	Esophageal cancer	Both	Light drinkers	Light drinkers	0.24	0.28	age, sex, bmi, education, income, physical activity, smoking, dietary factors, other
Freedman	2000	Esophageal cancer	Both	Light drinkers	Light drinkers	0.29	0.17	age, sex, bmi, education, income, physical activity, smoking, dietary factors, other
Freedman	2000	Esophageal cancer	Both	Light drinkers	Light drinkers	0.07	0.27	age, sex, bmi, education, income, physical activity, smoking, dietary factors, other
Freedman	2000	Esophageal cancer	Both	Light drinkers	Light drinkers	-0.07	0.18	age, sex, bmi, education, income, physical activity, smoking, dietary factors, other
Freedman	2000	Esophageal cancer	Both	Light drinkers	Light drinkers	-0.16	0.18	age, sex, bmi, education, income, physical activity, smoking, dietary factors, other
Freedman	2000	Esophageal cancer	Both	Light drinkers	Heavy drinkers	-0.16	0.37	age, sex, bmi, education, income, physical activity, smoking, dietary factors, other
Freedman	2000	Esophageal cancer	Both	Light drinkers	Light drinkers	-0.16	0.18	age, sex, bmi, education, income, physical activity, smoking, dietary factors, other
Freedman	2000	Esophageal cancer	Both	Light drinkers	Heavy drinkers	-0.16	0.37	age, sex, bmi, education, income, physical activity, smoking, dietary factors, other
Gao	1993	Esophageal cancer	Both	Non-drinkers	Light drinkers	0.10	0.50	age, sex, bmi, education, income, physical activity, smoking, dietary factors, other
Garidou	1991	Esophageal cancer	Both	Non-drinkers	Light drinkers	0.07	0.06	age, sex, bmi, education, income, physical activity
Han	2019	Esophageal cancer	Both	Non-drinkers	Light drinkers	0.03	0.12	representativeness, age, sex, bmi, education, income, physical activity, smoking, menopause, other drug use, dietary factors, other, hypertension, diabetes, vitamin deficiency, other diagnosed disease, APOE, family history, hypercholesterolemia
Hashibe	2002	Esophageal cancer	Both	Non-drinkers	Light drinkers	0.06	0.74	age, sex, bmi, education, income, physical activity, smoking, dietary factors
Ishiguro	2004	Esophageal cancer	Both	Non-drinkers	Light drinkers	0.49	0.27	age, sex, bmi, education, income, physical activity, smoking, dietary factors, other
Jayaprakash	1998	Esophageal cancer	Both	Non-drinkers	Light drinkers	-0.09	0.23	age, sex, bmi, education, income, physical activity
Kabat	1990	Esophageal cancer	Both	Light drinkers	Heavy drinkers	1.34	0.74	age, sex, bmi, education, income, physical activity, smoking, other
Kabat	1990	Esophageal cancer	Both	Light drinkers	Heavy drinkers	0.83	0.31	age, sex, bmi, education, income, physical activity, smoking, other
Kabat	1990	Esophageal cancer	Both	Light drinkers	Moderate drinkers	0.83	0.43	age, sex, bmi, education, income, physical activity, smoking, other
Kimm	2006	Esophageal cancer	Both	Non-drinkers	Heavy drinkers	1.34	0.12	age, sex, bmi, education, income, physical activity

Table S10. Summary of Data Inputs

Author	Year	Health outcome	Sex	Reference	Exposed group	Natural Log of Effect Size	Standard Error of Natural Log of Effect Size	Covariates Adjusted for in Source
Laaksonen	2018	Esophageal cancer	Both	Non-drinkers	Light drinkers	-0.54	0.31	representativeness, age, sex, bmi, education, income, physical activity, smoking, menopause, other drug use, dietary factors, other, hypertension, diabetes, vitamin deficiency, other diagnosed disease, APOE, family history, hypercholesterolemia
Lin	1997	Esophageal cancer	Both	Non-drinkers	Light drinkers	-0.08	0.17	representativeness, age, sex, bmi, education, income, physical activity, smoking, menopause, other drug use, dietary factors, other, hypertension, diabetes, vitamin deficiency, other diagnosed disease, APOE, family history, hypercholesterolemia
Lin	1997	Esophageal cancer	Both	Non-drinkers	Light drinkers	0.52	0.18	representativeness, age, sex, bmi, education, income, physical activity, smoking, menopause, other drug use, dietary factors, other, hypertension, diabetes, vitamin deficiency, other diagnosed disease, APOE, family history, hypercholesterolemia
Pandeya	2005	Esophageal cancer	Both	Non-drinkers	Light drinkers	-0.37	0.25	age, sex, bmi, education, income, physical activity, smoking, other drug use, other diagnosed disease
Pandeya	2005	Esophageal cancer	Both	Non-drinkers	Light drinkers	-0.34	0.28	age, sex, bmi, education, income, physical activity, smoking, other drug use, other diagnosed disease
Steevens	2002	Esophageal cancer	Both	Non-drinkers	Moderate drinkers	0.04	0.34	age, sex, bmi, education, income, physical activity, smoking, dietary factors, other
Steevens	2002	Esophageal cancer	Both	Non-drinkers	Light drinkers	-0.09	0.29	age, sex, bmi, education, income, physical activity, smoking, dietary factors, other
Steevens	2002	Esophageal cancer	Both	Non-drinkers	Moderate drinkers	0.01	0.30	age, sex, bmi, education, income, physical activity, smoking, dietary factors, other
Steevens	2002	Esophageal cancer	Both	Non-drinkers	Light drinkers	-0.16	0.36	age, sex, bmi, education, income, physical activity, smoking, dietary factors, other
Wu	2007	Esophageal cancer	Both	Non-drinkers	Light drinkers	-0.04	0.18	representativeness, age, sex, bmi, education, income, physical activity, smoking, menopause, other drug use, dietary factors, other, hypertension, diabetes, vitamin deficiency, other diagnosed disease, APOE, family history, hypercholesterolemia
Yang	2005	Esophageal cancer	Male	Non-drinkers	Light drinkers	0.17	0.13	representativeness, age, sex, bmi, education, income, physical activity, smoking, menopause, other drug use, dietary factors, other, hypertension, diabetes, vitamin deficiency, other diagnosed disease, APOE, family history, hypercholesterolemia
Yang	2004	Esophageal cancer	Both	Non-drinkers	Light drinkers	-0.16	0.56	age, sex, bmi, education, income, physical activity, smoking, dietary factors, other, family history
Yi	2005	Esophageal cancer	Both	Non-drinkers	Light drinkers	0.04	0.92	age, sex, bmi, education, income, physical activity, smoking, other
Yi	2005	Esophageal cancer	Both	Non-drinkers	Light drinkers	0.04	0.92	representativeness, age, sex, bmi, education, income, physical activity, smoking, menopause, other drug use, dietary factors, other, hypertension, diabetes, vitamin deficiency, other diagnosed disease, APOE, family history, hypercholesterolemia
Yu	2015	Esophageal cancer	Both	Light drinkers	Light drinkers	0.13	0.26	representativeness, age, sex, bmi, education, income, physical activity, smoking, menopause, other drug use, dietary factors, other, hypertension, diabetes, vitamin deficiency, other diagnosed disease, APOE, family history, hypercholesterolemia
Zhao	2017	Esophageal cancer	Both	Non-drinkers	Moderate drinkers	0.71	0.28	representativeness, age, sex, bmi, education, income, physical activity, smoking, menopause, other drug use, dietary factors, other, hypertension, diabetes, vitamin deficiency, other diagnosed disease, APOE, family history, hypercholesterolemia

Table S10. Summary of Data Inputs

Author	Year	Health outcome	Sex	Reference	Exposed group	Natural Log of Effect Size	Standard Error of Natural Log of Effect Size	Covariates Adjusted for in Source
Askgaard	2013	Haemorrhagic stroke	Both	Light drinkers	Light drinkers	0.01	0.22	representativeness, age, sex, bmi, education, income, physical activity, smoking, menopause, other drug use, dietary factors, other, hypertension, diabetes, vitamin deficiency, other diagnosed disease, APOE, family history, hypercholesterolemia
Askgaard	2013	Haemorrhagic stroke	Both	Light drinkers	Light drinkers	0.09	0.23	representativeness, age, sex, bmi, education, income, physical activity, smoking, menopause, other drug use, dietary factors, other, hypertension, diabetes, vitamin deficiency, other diagnosed disease, APOE, family history, hypercholesterolemia
Askgaard	2013	Haemorrhagic stroke	Both	Light drinkers	Heavy drinkers	0.10	0.52	representativeness, age, sex, bmi, education, income, physical activity, smoking, menopause, other drug use, dietary factors, other, hypertension, diabetes, vitamin deficiency, other diagnosed disease, APOE, family history, hypercholesterolemia
Goldberg	1980	Haemorrhagic stroke	Male	Non-drinkers	Moderate drinkers	0.00	0.51	representativeness, exposure_quality, outcome_quality, confounder_quality, reverse_causation, selection_bias, sex, hypertension
Gordon	1972	Haemorrhagic stroke	Male	Non-drinkers	Moderate drinkers	0.00	0.51	representativeness, exposure_quality, outcome_quality, confounder_quality, reverse_causation, selection_bias, sex, hypertension
Iso	2002	Haemorrhagic stroke	Male	Light drinkers	Moderate drinkers	0.83	0.28	representativeness, age, sex, bmi, education, income, physical activity, smoking, menopause, other drug use, dietary factors, other, hypertension, diabetes, vitamin deficiency, other diagnosed disease, APOE, family history, hypercholesterolemia
Kiyohara	1987	Haemorrhagic stroke	Both	Non-drinkers	Heavy drinkers	0.52	0.54	representativeness, age, sex, bmi, education, income, physical activity, smoking, menopause, other drug use, dietary factors, other, hypertension, diabetes, vitamin deficiency, other diagnosed disease, APOE, family history, hypercholesterolemia
Klatsky	1996	Haemorrhagic stroke	Both	Non-drinkers	Light drinkers	0.00	0.19	representativeness, exposure_quality, outcome_quality, confounder_quality, reverse_causation, selection_bias, sex, hypertension
Lu	2020	Haemorrhagic stroke	Male	Non-drinkers	Light drinkers	-0.53	0.37	representativeness, age, sex, bmi, education, income, physical activity, smoking, menopause, other drug use, dietary factors, other, hypertension, diabetes, vitamin deficiency, other diagnosed disease, APOE, family history, hypercholesterolemia
Millwood	2017	Haemorrhagic stroke	Female	N/A	Heavy drinkers	-0.04	0.08	representativeness, age, sex, bmi, education, income, physical activity, smoking, menopause, other drug use, dietary factors, other, hypertension, diabetes, vitamin deficiency, other diagnosed disease, APOE, family history, hypercholesterolemia
Sankai	1993	Haemorrhagic stroke	Both	Non-drinkers	Moderate drinkers	0.00	0.39	representativeness, age, sex, bmi, education, income, physical activity, smoking, menopause, other drug use, dietary factors, other, hypertension, diabetes, vitamin deficiency, other diagnosed disease, APOE, family history, hypercholesterolemia
Biddinger	2016	Ischemic heart disease	Both	Non-drinkers	Light drinkers	-0.02	0.03	representativeness, age, sex, bmi, education, income, physical activity, smoking, menopause, other drug use, dietary factors, other, hypertension, diabetes, vitamin deficiency, other diagnosed disease, APOE, family history, hypercholesterolemia

Table S10. Summary of Data Inputs

Author	Year	Health outcome	Sex	Reference	Exposed group	Natural Log of Effect Size	Standard Error of Natural Log of Effect Size	Covariates Adjusted for in Source
Biddinger	2016	Ischemic heart disease	Both	Non-drinkers	Moderate drinkers	0.03	0.03	representativeness, age, sex, bmi, education, income, physical activity, smoking, menopause, other drug use, dietary factors, other, hypertension, diabetes, vitamin deficiency, other diagnosed disease, APOE, family history, hypercholesterolemia
Bos	2006	Ischemic heart disease	Female	Non-drinkers	Light drinkers	-0.02	0.13	representativeness, age, sex, bmi, education, income, physical activity, smoking, menopause, other drug use, dietary factors, other, hypertension, diabetes, vitamin deficiency, other diagnosed disease, APOE, family history, hypercholesterolemia
Bos	2006	Ischemic heart disease	Female	Non-drinkers	Light drinkers	-0.08	0.15	representativeness, age, sex, bmi, education, income, physical activity, smoking, menopause, other drug use, dietary factors, other, hypertension, diabetes, vitamin deficiency, other diagnosed disease, APOE, family history, hypercholesterolemia
Britton	2007	Ischemic heart disease	Male	Light drinkers	Light drinkers	0.05	0.10	representativeness, age, sex, bmi, education, income, physical activity, smoking, menopause, other drug use, dietary factors, other, hypertension, diabetes, vitamin deficiency, other diagnosed disease, APOE, family history, hypercholesterolemia
Bryson	2001	Ischemic heart disease	Both	Non-drinkers	Moderate drinkers	-0.03	0.16	representativeness, age, sex, bmi, education, income, physical activity, smoking, menopause, other drug use, dietary factors, other, hypertension, diabetes, vitamin deficiency, other diagnosed disease, APOE, family history, hypercholesterolemia
Camargo	1993	Ischemic heart disease	Male	Light drinkers	Light drinkers	0.00	0.08	representativeness, exposure_quality, outcome_quality, confounder_quality, reverse_causation, selection_bias, sex, hypertension
Cho	2017	Ischemic heart disease	Both	Non-drinkers	Moderate drinkers	-0.34	0.01	representativeness, age, sex, bmi, education, income, physical activity, smoking, menopause, other drug use, dietary factors, other, hypertension, diabetes, vitamin deficiency, other diagnosed disease, APOE, family history, hypercholesterolemia
Cullen	1989	Ischemic heart disease	Both	Non-drinkers	Light drinkers	-0.08	0.15	representativeness, age, sex, bmi, education, income, physical activity, smoking, menopause, other drug use, dietary factors, other, hypertension, diabetes, vitamin deficiency, other diagnosed disease, APOE, family history, hypercholesterolemia
Dam	2012	Ischemic heart disease	Female	Light drinkers	Light drinkers	0.00	0.07	representativeness, exposure_quality, outcome_quality, confounder_quality, reverse_causation, selection_bias, sex, hypertension
Friedman	1962	Ischemic heart disease	Female	Non-drinkers	Moderate drinkers	0.00	0.46	representativeness, exposure_quality, outcome_quality, confounder_quality, reverse_causation, selection_bias, sex, hypertension
Friedman	1962	Ischemic heart disease	Female	Non-drinkers	Heavy drinkers	0.00	0.76	representativeness, exposure_quality, outcome_quality, confounder_quality, reverse_causation, selection_bias, sex, hypertension
Fuchs	1992	Ischemic heart disease	Female	Non-drinkers	Moderate drinkers	-0.53	0.27	representativeness, age, sex, bmi, education, income, physical activity, smoking, menopause, other drug use, dietary factors, other, hypertension, diabetes, vitamin deficiency, other diagnosed disease, APOE, family history, hypercholesterolemia
Garg	1987	Ischemic heart disease	Female	Non-drinkers	Moderate drinkers	-0.34	0.22	representativeness, age, sex, bmi, education, income, physical activity, smoking, menopause, other drug use, dietary factors, other, hypertension, diabetes, vitamin deficiency, other diagnosed disease, APOE, family history, hypercholesterolemia

Table S10. Summary of Data Inputs

Author	Year	Health outcome	Sex	Reference	Exposed group	Natural Log of Effect Size	Standard Error of Natural Log of Effect Size	Covariates Adjusted for in Source
Han	2017	Ischemic heart disease	Both	Light drinkers	Moderate drinkers	0.03	0.02	representativeness, age, sex, bmi, education, income, physical activity, smoking, menopause, other drug use, dietary factors, other, hypertension, diabetes, vitamin deficiency, other diagnosed disease, APOE, family history, hypercholesterolemia
Hart	2003	Ischemic heart disease	Male	Non-drinkers	Moderate drinkers	-0.62	0.29	representativeness, age, sex, bmi, education, income, physical activity, smoking, menopause, other drug use, dietary factors, other, hypertension, diabetes, vitamin deficiency, other diagnosed disease, APOE, family history, hypercholesterolemia
Hein	1986	Ischemic heart disease	Male	Non-drinkers	Heavy drinkers	0.10	0.68	representativeness, age, sex, bmi, education, income, physical activity, smoking, menopause, other drug use, dietary factors, other, hypertension, diabetes, vitamin deficiency, other diagnosed disease, APOE, family history, hypercholesterolemia
Hein	1986	Ischemic heart disease	Male	Non-drinkers	Light drinkers	-0.92	0.41	representativeness, age, sex, bmi, education, income, physical activity, smoking, menopause, other drug use, dietary factors, other, hypertension, diabetes, vitamin deficiency, other diagnosed disease, APOE, family history, hypercholesterolemia
Higashiyama	2007	Ischemic heart disease	Male	Non-drinkers	Light drinkers	-0.54	0.33	representativeness, age, sex, bmi, education, income, physical activity, smoking, menopause, other drug use, dietary factors, other, hypertension, diabetes, vitamin deficiency, other diagnosed disease, APOE, family history, hypercholesterolemia
Higashiyama	2007	Ischemic heart disease	Male	Non-drinkers	Heavy drinkers	-0.09	0.48	representativeness, age, sex, bmi, education, income, physical activity, smoking, menopause, other drug use, dietary factors, other, hypertension, diabetes, vitamin deficiency, other diagnosed disease, APOE, family history, hypercholesterolemia
Hu	2015	Ischemic heart disease	Male	Non-drinkers	Heavy drinkers	0.04	0.16	representativeness, age, sex, bmi, education, income, physical activity, smoking, menopause, other drug use, dietary factors, other, hypertension, diabetes, vitamin deficiency, other diagnosed disease, APOE, family history, hypercholesterolemia
Jiang	2008	Ischemic heart disease	Both	Non-drinkers	Light drinkers	0.14	0.11	representativeness, age, sex, bmi, education, income, physical activity, smoking, menopause, other drug use, dietary factors, other, hypertension, diabetes, vitamin deficiency, other diagnosed disease, APOE, family history, hypercholesterolemia
Kim	2016	Ischemic heart disease	Both	Non-drinkers	Light drinkers	-0.07	0.05	representativeness, age, sex, bmi, education, income, physical activity, smoking, menopause, other drug use, dietary factors, other, hypertension, diabetes, vitamin deficiency, other diagnosed disease, APOE, family history, hypercholesterolemia
Kivela	1984	Ischemic heart disease	Male	Non-drinkers	Light drinkers	0.00	0.27	representativeness, exposure_quality, outcome_quality, confounder_quality, reverse_causation, selection_bias, sex, hypertension
Klatsky	1991	Ischemic heart disease	Male	Non-drinkers	Light drinkers	-0.07	0.08	representativeness, age, sex, bmi, education, income, physical activity, smoking, menopause, other drug use, dietary factors, other, hypertension, diabetes, vitamin deficiency, other diagnosed disease, APOE, family history, hypercholesterolemia
Klatsky	1991	Ischemic heart disease	Male	Non-drinkers	Heavy drinkers	-0.34	0.10	representativeness, age, sex, bmi, education, income, physical activity, smoking, menopause, other drug use, dietary factors, other, hypertension, diabetes, vitamin deficiency, other diagnosed disease, APOE, family history, hypercholesterolemia

Table S10. Summary of Data Inputs

Author	Year	Health outcome	Sex	Reference	Exposed group	Natural Log of Effect Size	Standard Error of Natural Log of Effect Size	Covariates Adjusted for in Source
Leong	2003	Ischemic heart disease	Both	Non-drinkers	Light drinkers	-0.12	0.05	representativeness, age, sex, bmi, education, income, physical activity, smoking, menopause, other drug use, dietary factors, other, hypertension, diabetes, vitamin deficiency, other diagnosed disease, APOE, family history, hypercholesterolemia
Malyutina	1998	Ischemic heart disease	Male	Non-drinkers	Moderate drinkers	0.00	0.20	representativeness, exposure_quality, outcome_quality, confounder_quality, reverse_causation, selection_bias, sex, hypertension
Mukamal	1998	Ischemic heart disease	Male	Non-drinkers	Light drinkers	-0.02	0.08	representativeness, age, sex, bmi, education, income, physical activity, smoking, menopause, other drug use, dietary factors, other, hypertension, diabetes, vitamin deficiency, other diagnosed disease, APOE, family history, hypercholesterolemia
Mukamal	1998	Ischemic heart disease	Male	Non-drinkers	Light drinkers	-0.37	0.10	representativeness, age, sex, bmi, education, income, physical activity, smoking, menopause, other drug use, dietary factors, other, hypertension, diabetes, vitamin deficiency, other diagnosed disease, APOE, family history, hypercholesterolemia
Mukamal	2001	Ischemic heart disease	Both	Non-drinkers	Light drinkers	-0.07	0.13	representativeness, age, sex, bmi, education, income, physical activity, smoking, menopause, other drug use, dietary factors, other, hypertension, diabetes, vitamin deficiency, other diagnosed disease, APOE, family history, hypercholesterolemia
Mukamal	2001	Ischemic heart disease	Both	Non-drinkers	Moderate drinkers	-0.62	0.20	representativeness, age, sex, bmi, education, income, physical activity, smoking, menopause, other drug use, dietary factors, other, hypertension, diabetes, vitamin deficiency, other diagnosed disease, APOE, family history, hypercholesterolemia
Mukamal	2000	Ischemic heart disease	Female	Non-drinkers	Light drinkers	-0.04	0.05	representativeness, age, sex, bmi, education, income, physical activity, smoking, menopause, other drug use, dietary factors, other, hypertension, diabetes, vitamin deficiency, other diagnosed disease, APOE, family history, hypercholesterolemia
Mukamal	2000	Ischemic heart disease	Female	Non-drinkers	Light drinkers	0.42	0.35	representativeness, age, sex, bmi, education, income, physical activity, smoking, menopause, other drug use, dietary factors, other, hypertension, diabetes, vitamin deficiency, other diagnosed disease, APOE, family history, hypercholesterolemia
Mukamal	2000	Ischemic heart disease	Female	Non-drinkers	Light drinkers	-0.58	0.37	representativeness, age, sex, bmi, education, income, physical activity, smoking, menopause, other drug use, dietary factors, other, hypertension, diabetes, vitamin deficiency, other diagnosed disease, APOE, family history, hypercholesterolemia
Mukamal	2000	Ischemic heart disease	Male	Non-drinkers	Light drinkers	0.07	0.25	representativeness, age, sex, bmi, education, income, physical activity, smoking, menopause, other drug use, dietary factors, other, hypertension, diabetes, vitamin deficiency, other diagnosed disease, APOE, family history, hypercholesterolemia
Muraki	1990	Ischemic heart disease	Male	Non-drinkers	Light drinkers	-0.34	0.15	representativeness, age, sex, bmi, education, income, physical activity, smoking, menopause, other drug use, dietary factors, other, hypertension, diabetes, vitamin deficiency, other diagnosed disease, APOE, family history, hypercholesterolemia
Murray	1999	Ischemic heart disease	Female	Light drinkers	Light drinkers	0.52	0.48	representativeness, age, sex, bmi, education, income, physical activity, smoking, menopause, other drug use, dietary factors, other, hypertension, diabetes, vitamin deficiency, other diagnosed disease, APOE, family history, hypercholesterolemia

Table S10. Summary of Data Inputs

Author	Year	Health outcome	Sex	Reference	Exposed group	Natural Log of Effect Size	Standard Error of Natural Log of Effect Size	Covariates Adjusted for in Source
Murray	1998	Ischemic heart disease	Male	Non-drinkers	Moderate drinkers	-0.37	0.12	representativeness, age, sex, bmi, education, income, physical activity, smoking, menopause, other drug use, dietary factors, other, hypertension, diabetes, vitamin deficiency, other diagnosed disease, APOE, family history, hypercholesterolemia
Palmer	1993	Ischemic heart disease	Male	Non-drinkers	Moderate drinkers	0.05	0.19	representativeness, age, sex, bmi, education, income, physical activity, smoking, menopause, other drug use, dietary factors, other, hypertension, diabetes, vitamin deficiency, other diagnosed disease, APOE, family history, hypercholesterolemia
Palmer	1993	Ischemic heart disease	Female	Non-drinkers	Light drinkers	0.03	0.20	representativeness, age, sex, bmi, education, income, physical activity, smoking, menopause, other drug use, dietary factors, other, hypertension, diabetes, vitamin deficiency, other diagnosed disease, APOE, family history, hypercholesterolemia
Palmer	1993	Ischemic heart disease	Female	Non-drinkers	Light drinkers	-0.16	0.33	representativeness, age, sex, bmi, education, income, physical activity, smoking, menopause, other drug use, dietary factors, other, hypertension, diabetes, vitamin deficiency, other diagnosed disease, APOE, family history, hypercholesterolemia
Reddiess	2017	Ischemic heart disease	Both	Non-drinkers	Light drinkers	-0.94	0.35	representativeness, age, sex, bmi, education, income, physical activity, smoking, menopause, other drug use, dietary factors, other, hypertension, diabetes, vitamin deficiency, other diagnosed disease, APOE, family history, hypercholesterolemia
Renaud	1996	Ischemic heart disease	Male	Non-drinkers	Light drinkers	-0.01	0.14	representativeness, age, sex, bmi, education, income, physical activity, smoking, menopause, other drug use, dietary factors, other, hypertension, diabetes, vitamin deficiency, other diagnosed disease, APOE, family history, hypercholesterolemia
Renaud	1996	Ischemic heart disease	Male	Non-drinkers	Heavy drinkers	-0.65	0.10	representativeness, age, sex, bmi, education, income, physical activity, smoking, menopause, other drug use, dietary factors, other, hypertension, diabetes, vitamin deficiency, other diagnosed disease, APOE, family history, hypercholesterolemia
Renaud	1996	Ischemic heart disease	Male	Non-drinkers	Heavy drinkers	-0.16	0.24	representativeness, age, sex, bmi, education, income, physical activity, smoking, menopause, other drug use, dietary factors, other, hypertension, diabetes, vitamin deficiency, other diagnosed disease, APOE, family history, hypercholesterolemia
Renaud	1996	Ischemic heart disease	Male	Non-drinkers	Heavy drinkers	0.08	0.14	representativeness, age, sex, bmi, education, income, physical activity, smoking, menopause, other drug use, dietary factors, other, hypertension, diabetes, vitamin deficiency, other diagnosed disease, APOE, family history, hypercholesterolemia
Renaud	1996	Ischemic heart disease	Male	Non-drinkers	Heavy drinkers	-0.54	0.41	representativeness, age, sex, bmi, education, income, physical activity, smoking, menopause, other drug use, dietary factors, other, hypertension, diabetes, vitamin deficiency, other diagnosed disease, APOE, family history, hypercholesterolemia
Renaud	1996	Ischemic heart disease	Male	Non-drinkers	Heavy drinkers	0.05	0.28	representativeness, age, sex, bmi, education, income, physical activity, smoking, menopause, other drug use, dietary factors, other, hypertension, diabetes, vitamin deficiency, other diagnosed disease, APOE, family history, hypercholesterolemia
Renaud	1996	Ischemic heart disease	Male	Non-drinkers	Heavy drinkers	-0.37	0.10	representativeness, age, sex, bmi, education, income, physical activity, smoking, menopause, other drug use, dietary factors, other, hypertension, diabetes, vitamin deficiency, other diagnosed disease, APOE, family history, hypercholesterolemia

Table S10. Summary of Data Inputs

Author	Year	Health outcome	Sex	Reference	Exposed group	Natural Log of Effect Size	Standard Error of Natural Log of Effect Size	Covariates Adjusted for in Source
Renaud	1996	Ischemic heart disease	Male	Non-drinkers	Heavy drinkers	-0.37	0.24	representativeness, age, sex, bmi, education, income, physical activity, smoking, menopause, other drug use, dietary factors, other, hypertension, diabetes, vitamin deficiency, other diagnosed disease, APOE, family history, hypercholesterolemia
Ruidavets	2004	Ischemic heart disease	Male	Light drinkers	Moderate drinkers	0.00	0.23	representativeness, exposure_quality, outcome_quality, confounder_quality, reverse_causation, selection_bias, sex, hypertension
Schooling	2003	Ischemic heart disease	Male	Non-drinkers	Light drinkers	0.00	0.31	representativeness, exposure_quality, outcome_quality, confounder_quality, reverse_causation, selection_bias, sex, hypertension
Schutte	2019	Ischemic heart disease	Both	Non-drinkers	Moderate drinkers	-0.01	0.12	representativeness, age, sex, bmi, education, income, physical activity, smoking, menopause, other drug use, dietary factors, other, hypertension, diabetes, vitamin deficiency, other diagnosed disease, APOE, family history, hypercholesterolemia
Shaper	1980	Ischemic heart disease	Male	Light drinkers	Light drinkers	0.00	0.26	representativeness, exposure_quality, outcome_quality, confounder_quality, reverse_causation, selection_bias, sex, hypertension
Shaper	1980	Ischemic heart disease	Male	Light drinkers	Light drinkers	0.00	0.26	representativeness, exposure_quality, outcome_quality, confounder_quality, reverse_causation, selection_bias, sex, hypertension
Shaper	1980	Ischemic heart disease	Male	Light drinkers	Light drinkers	0.00	0.26	representativeness, exposure_quality, outcome_quality, confounder_quality, reverse_causation, selection_bias, sex, hypertension
Smith	2017	Ischemic heart disease	Both	Non-drinkers	Moderate drinkers	-0.53	0.18	representativeness, age, sex, bmi, education, income, physical activity, smoking, menopause, other drug use, dietary factors, other, hypertension, diabetes, vitamin deficiency, other diagnosed disease, APOE, family history, hypercholesterolemia
Tolstrup	2004	Ischemic heart disease	Male	Light drinkers	Light drinkers	-0.04	0.36	representativeness, age, sex, bmi, education, income, physical activity, smoking, menopause, other drug use, dietary factors, other, hypertension, diabetes, vitamin deficiency, other diagnosed disease, APOE, family history, hypercholesterolemia
Tolstrup	2004	Ischemic heart disease	Male	Light drinkers	Heavy drinkers	-0.03	0.25	representativeness, age, sex, bmi, education, income, physical activity, smoking, menopause, other drug use, dietary factors, other, hypertension, diabetes, vitamin deficiency, other diagnosed disease, APOE, family history, hypercholesterolemia
Valmadrid	1996	Ischemic heart disease	Both	Non-drinkers	Light drinkers	-0.62	0.26	representativeness, age, sex, bmi, education, income, physical activity, smoking, menopause, other drug use, dietary factors, other, hypertension, diabetes, vitamin deficiency, other diagnosed disease, APOE, family history, hypercholesterolemia
Vliegenthart	2003	Ischemic heart disease	Both	Non-drinkers	Light drinkers	-0.84	0.22	representativeness, age, sex, bmi, education, income, physical activity, smoking, menopause, other drug use, dietary factors, other, hypertension, diabetes, vitamin deficiency, other diagnosed disease, APOE, family history, hypercholesterolemia
Walsh	2001	Ischemic heart disease	Female	Non-drinkers	Light drinkers	0.04	0.31	representativeness, age, sex, bmi, education, income, physical activity, smoking, menopause, other drug use, dietary factors, other, hypertension, diabetes, vitamin deficiency, other diagnosed disease, APOE, family history, hypercholesterolemia

Table S10. Summary of Data Inputs

Author	Year	Health outcome	Sex	Reference	Exposed group	Natural Log of Effect Size	Standard Error of Natural Log of Effect Size	Covariates Adjusted for in Source
Wannamethee	2010	Ischemic heart disease	Male	Light drinkers	Light drinkers	-0.05	0.26	representativeness, age, sex, bmi, education, income, physical activity, smoking, menopause, other drug use, dietary factors, other, hypertension, diabetes, vitamin deficiency, other diagnosed disease, APOE, family history, hypercholesterolemia
Wannamethee	2010	Ischemic heart disease	Male	Light drinkers	Light drinkers	-0.01	0.22	representativeness, age, sex, bmi, education, income, physical activity, smoking, menopause, other drug use, dietary factors, other, hypertension, diabetes, vitamin deficiency, other diagnosed disease, APOE, family history, hypercholesterolemia
Wellmann	1997	Ischemic heart disease	Male	Non-drinkers	Moderate drinkers	-0.84	0.33	representativeness, age, sex, bmi, education, income, physical activity, smoking, menopause, other drug use, dietary factors, other, hypertension, diabetes, vitamin deficiency, other diagnosed disease, APOE, family history, hypercholesterolemia
Cho	2017	Ischemic stroke	Both	Non-drinkers	Light drinkers	-0.09	0.01	representativeness, age, sex, bmi, education, income, physical activity, smoking, menopause, other drug use, dietary factors, other, hypertension, diabetes, vitamin deficiency, other diagnosed disease, APOE, family history, hypercholesterolemia
Cho	2017	Ischemic stroke	Both	Non-drinkers	Moderate drinkers	-0.06	0.01	representativeness, age, sex, bmi, education, income, physical activity, smoking, menopause, other drug use, dietary factors, other, hypertension, diabetes, vitamin deficiency, other diagnosed disease, APOE, family history, hypercholesterolemia
Cho	2017	Ischemic stroke	Both	Non-drinkers	Moderate drinkers	0.04	0.01	representativeness, age, sex, bmi, education, income, physical activity, smoking, menopause, other drug use, dietary factors, other, hypertension, diabetes, vitamin deficiency, other diagnosed disease, APOE, family history, hypercholesterolemia
Donahue	1977	Ischemic stroke	Male	Non-drinkers	Light drinkers	0.00	0.18	representativeness, exposure_quality, outcome_quality, confounder_quality, reverse_causation, selection_bias, sex, hypertension
Goldberg	1980	Ischemic stroke	Male	Non-drinkers	Moderate drinkers	0.00	0.51	representativeness, exposure_quality, outcome_quality, confounder_quality, reverse_causation, selection_bias, sex, hypertension
Iso	2002	Ischemic stroke	Male	Light drinkers	Light drinkers	-0.53	0.24	representativeness, age, sex, bmi, education, income, physical activity, smoking, menopause, other drug use, dietary factors, other, hypertension, diabetes, vitamin deficiency, other diagnosed disease, APOE, family history, hypercholesterolemia
Iso	2002	Ischemic stroke	Male	Light drinkers	Moderate drinkers	-0.08	0.21	representativeness, age, sex, bmi, education, income, physical activity, smoking, menopause, other drug use, dietary factors, other, hypertension, diabetes, vitamin deficiency, other diagnosed disease, APOE, family history, hypercholesterolemia
Iso	2002	Ischemic stroke	Male	Light drinkers	Heavy drinkers	-0.06	0.23	representativeness, age, sex, bmi, education, income, physical activity, smoking, menopause, other drug use, dietary factors, other, hypertension, diabetes, vitamin deficiency, other diagnosed disease, APOE, family history, hypercholesterolemia
Iso	2002	Ischemic stroke	Male	Light drinkers	Heavy drinkers	0.03	0.22	representativeness, age, sex, bmi, education, income, physical activity, smoking, menopause, other drug use, dietary factors, other, hypertension, diabetes, vitamin deficiency, other diagnosed disease, APOE, family history, hypercholesterolemia

Table S10. Summary of Data Inputs

Author	Year	Health outcome	Sex	Reference	Exposed group	Natural Log of Effect Size	Standard Error of Natural Log of Effect Size	Covariates Adjusted for in Source
Kim	2016	Ischemic stroke	Both	Non-drinkers	Light drinkers	-0.01	0.05	representativeness, age, sex, bmi, education, income, physical activity, smoking, menopause, other drug use, dietary factors, other, hypertension, diabetes, vitamin deficiency, other diagnosed disease, APOE, family history, hypercholesterolemia
Lu	2020	Ischemic stroke	Male	Non-drinkers	Moderate drinkers	-0.07	0.22	representativeness, age, sex, bmi, education, income, physical activity, smoking, menopause, other drug use, dietary factors, other, hypertension, diabetes, vitamin deficiency, other diagnosed disease, APOE, family history, hypercholesterolemia
Millwood	2017	Ischemic stroke	Male	N/A	Heavy drinkers	0.24	0.06	representativeness, age, sex, bmi, education, income, physical activity, smoking, menopause, other drug use, dietary factors, other, hypertension, diabetes, vitamin deficiency, other diagnosed disease, APOE, family history, hypercholesterolemia
Millwood	2017	Ischemic stroke	Female	N/A	Heavy drinkers	-0.02	0.05	representativeness, age, sex, bmi, education, income, physical activity, smoking, menopause, other drug use, dietary factors, other, hypertension, diabetes, vitamin deficiency, other diagnosed disease, APOE, family history, hypercholesterolemia
Rist	2008	Ischemic stroke	Male	Light drinkers	Light drinkers	0.00	0.09	representativeness, exposure_quality, outcome_quality, confounder_quality, reverse_causation, selection_bias, sex, hypertension
Sacco	1997	Ischemic stroke	Both	Moderate drinkers	Heavy drinkers	0.83	0.41	representativeness, age, sex, bmi, education, income, physical activity, smoking, menopause, other drug use, dietary factors, other, hypertension, diabetes, vitamin deficiency, other diagnosed disease, APOE, family history, hypercholesterolemia
Yang	2013	Ischemic stroke	Both	Non-drinkers	Moderate drinkers	0.09	0.13	representativeness, age, sex, bmi, education, income, physical activity, smoking, menopause, other drug use, dietary factors, other, hypertension, diabetes, vitamin deficiency, other diagnosed disease, APOE, family history, hypercholesterolemia
Cho	2013	Larynx cancer	Both	Non-drinkers	Moderate drinkers	0.29	0.31	representativeness, age, sex, bmi, education, income, physical activity, smoking, menopause, other drug use, dietary factors, other, hypertension, diabetes, vitamin deficiency, other diagnosed disease, APOE, family history, hypercholesterolemia
Cho	2013	Larynx cancer	Both	Non-drinkers	Moderate drinkers	0.06	0.29	representativeness, age, sex, bmi, education, income, physical activity, smoking, menopause, other drug use, dietary factors, other, hypertension, diabetes, vitamin deficiency, other diagnosed disease, APOE, family history, hypercholesterolemia
Flanders	1979	Larynx cancer	Both	Light drinkers	Moderate drinkers	0.37	0.41	age, sex, bmi, education, income, physical activity
Kawakita	2009	Larynx cancer	Both	Non-drinkers	Light drinkers	0.05	0.10	representativeness, age, sex, bmi, education, income, physical activity, smoking, menopause, other drug use, dietary factors, other, hypertension, diabetes, vitamin deficiency, other diagnosed disease, APOE, family history, hypercholesterolemia
Lee	2018	Larynx cancer	Both	Non-drinkers	Light drinkers	0.38	0.04	representativeness, age, sex, bmi, education, income, physical activity, smoking, menopause, other drug use, dietary factors, other, hypertension, diabetes, vitamin deficiency, other diagnosed disease, APOE, family history, hypercholesterolemia

Table S10. Summary of Data Inputs

Author	Year	Health outcome	Sex	Reference	Exposed group	Natural Log of Effect Size	Standard Error of Natural Log of Effect Size	Covariates Adjusted for in Source
Lubin	2011	Larynx cancer	Female	Light drinkers	Heavy drinkers	-0.65	0.84	representativeness, age, sex, bmi, education, income, physical activity, smoking, menopause, other drug use, dietary factors, other, hypertension, diabetes, vitamin deficiency, other diagnosed disease, APOE, family history, hypercholesterolemia
Lubin	2011	Larynx cancer	Male	Light drinkers	Heavy drinkers	0.64	0.26	representativeness, age, sex, bmi, education, income, physical activity, smoking, menopause, other drug use, dietary factors, other, hypertension, diabetes, vitamin deficiency, other diagnosed disease, APOE, family history, hypercholesterolemia
Maasland	2003	Larynx cancer	Both	Non-drinkers	Moderate drinkers	-0.04	0.33	age, sex, bmi, education, income, physical activity, smoking
Menvielle	1991	Larynx cancer	Both	Light drinkers	Light drinkers	0.26	0.10	age, sex, bmi, education, income, physical activity
Menvielle	1991	Larynx cancer	Both	Light drinkers	Heavy drinkers	0.83	0.33	age, sex, bmi, education, income, physical activity
Menvielle	1991	Larynx cancer	Both	Light drinkers	Heavy drinkers	0.83	0.38	age, sex, bmi, education, income, physical activity
Menvielle	1991	Larynx cancer	Both	Light drinkers	Heavy drinkers	0.83	0.33	age, sex, bmi, education, income, physical activity
Menvielle	1991	Larynx cancer	Both	Light drinkers	Heavy drinkers	0.83	0.38	age, sex, bmi, education, income, physical activity
Menvielle	2007	Larynx cancer	Male	Non-drinkers	Heavy drinkers	0.13	0.29	representativeness, age, sex, bmi, education, income, physical activity, smoking, menopause, other drug use, dietary factors, other, hypertension, diabetes, vitamin deficiency, other diagnosed disease, APOE, family history, hypercholesterolemia
Menvielle	2007	Larynx cancer	Male	Non-drinkers	Moderate drinkers	0.13	0.29	representativeness, age, sex, bmi, education, income, physical activity, smoking, menopause, other drug use, dietary factors, other, hypertension, diabetes, vitamin deficiency, other diagnosed disease, APOE, family history, hypercholesterolemia
Menvielle	2007	Larynx cancer	Male	Non-drinkers	Heavy drinkers	0.92	0.29	representativeness, age, sex, bmi, education, income, physical activity, smoking, menopause, other drug use, dietary factors, other, hypertension, diabetes, vitamin deficiency, other diagnosed disease, APOE, family history, hypercholesterolemia
Muscat	1990	Larynx cancer	Both	Light drinkers	Heavy drinkers	0.10	0.34	age, sex, bmi, education, income, physical activity, smoking
Ramroth	2000	Larynx cancer	Both	Light drinkers	Moderate drinkers	0.26	0.25	age, sex, bmi, education, income, physical activity, smoking
Ramroth	2000	Larynx cancer	Both	Light drinkers	Heavy drinkers	0.26	0.20	representativeness, age, sex, bmi, education, income, physical activity, smoking, menopause, other drug use, dietary factors, other, hypertension, diabetes, vitamin deficiency, other diagnosed disease, APOE, family history, hypercholesterolemia
Ramroth	2000	Larynx cancer	Both	Light drinkers	Heavy drinkers	0.88	0.21	representativeness, age, sex, bmi, education, income, physical activity, smoking, menopause, other drug use, dietary factors, other, hypertension, diabetes, vitamin deficiency, other diagnosed disease, APOE, family history, hypercholesterolemia
Santi	2000	Larynx cancer	Male	Non-drinkers	Heavy drinkers	1.29	0.21	representativeness, age, sex, bmi, education, income, physical activity, smoking, menopause, other drug use, dietary factors, other, hypertension, diabetes, vitamin deficiency, other diagnosed disease, APOE, family history, hypercholesterolemia

Table S10. Summary of Data Inputs

Author	Year	Health outcome	Sex	Reference	Exposed group	Natural Log of Effect Size	Standard Error of Natural Log of Effect Size	Covariates Adjusted for in Source
Santi	2000	Larynx cancer	Male	Non-drinkers	Heavy drinkers	1.29	0.21	representativeness, age, sex, bmi, education, income, physical activity, smoking, menopause, other drug use, dietary factors, other, hypertension, diabetes, vitamin deficiency, other diagnosed disease, APOE, family history, hypercholesterolemia
Zheng	1990	Larynx cancer	Both	Non-drinkers	Moderate drinkers	0.00	0.35	age, sex, bmi, education, income, physical activity, smoking
Chen	2017	Lip and oral cavity cancer	Male	Non-drinkers	Light drinkers	0.05	0.16	representativeness, age, sex, bmi, education, income, physical activity, smoking, menopause, other drug use, dietary factors, other, hypertension, diabetes, vitamin deficiency, other diagnosed disease, APOE, family history, hypercholesterolemia
Chen	2017	Lip and oral cavity cancer	Male	Non-drinkers	Heavy drinkers	0.92	0.18	representativeness, age, sex, bmi, education, income, physical activity, smoking, menopause, other drug use, dietary factors, other, hypertension, diabetes, vitamin deficiency, other diagnosed disease, APOE, family history, hypercholesterolemia
Herrero	1999	Lip and oral cavity cancer	Both	Non-drinkers	Moderate drinkers	0.26	0.12	age, sex, bmi, education, income, physical activity, smoking, other
Huang	2016	Lip and oral cavity cancer	Both	Non-drinkers	Light drinkers	-0.16	0.20	representativeness, age, sex, bmi, education, income, physical activity, smoking, menopause, other drug use, dietary factors, other, hypertension, diabetes, vitamin deficiency, other diagnosed disease, APOE, family history, hypercholesterolemia
Radoï	2007	Lip and oral cavity cancer	Both	Non-drinkers	Light drinkers	-0.92	0.22	representativeness, age, sex, bmi, education, income, physical activity, smoking, menopause, other drug use, dietary factors, other, hypertension, diabetes, vitamin deficiency, other diagnosed disease, APOE, family history, hypercholesterolemia
Radoï	2007	Lip and oral cavity cancer	Both	Non-drinkers	Moderate drinkers	0.35	0.20	representativeness, age, sex, bmi, education, income, physical activity, smoking, menopause, other drug use, dietary factors, other, hypertension, diabetes, vitamin deficiency, other diagnosed disease, APOE, family history, hypercholesterolemia
Radoï	2007	Lip and oral cavity cancer	Both	Non-drinkers	Moderate drinkers	0.06	0.20	representativeness, age, sex, bmi, education, income, physical activity, smoking, menopause, other drug use, dietary factors, other, hypertension, diabetes, vitamin deficiency, other diagnosed disease, APOE, family history, hypercholesterolemia
Shanmugham	2006	Lip and oral cavity cancer	Female	Non-drinkers	Light drinkers	-0.53	0.20	representativeness, age, sex, bmi, education, income, physical activity, smoking, menopause, other drug use, dietary factors, other, hypertension, diabetes, vitamin deficiency, other diagnosed disease, APOE, family history, hypercholesterolemia
Werbrouck	2006	Lip and oral cavity cancer	Both	Non-drinkers	Light drinkers	0.07	0.73	age, sex, bmi, education, income, physical activity, smoking
Asim	2007	Liver cancer	Both	Moderate drinkers	Heavy drinkers	0.24	0.21	representativeness, age, sex, bmi, education, income, physical activity, smoking, menopause, other drug use, dietary factors, other, hypertension, diabetes, vitamin deficiency, other diagnosed disease, APOE, family history, hypercholesterolemia
Bui	2018	Liver cancer	Male	Non-drinkers	Light drinkers	-0.06	0.02	representativeness, age, sex, bmi, education, income, physical activity, smoking, menopause, other drug use, dietary factors, other, hypertension, diabetes, vitamin deficiency, other diagnosed disease, APOE, family history, hypercholesterolemia

Table S10. Summary of Data Inputs

Author	Year	Health outcome	Sex	Reference	Exposed group	Natural Log of Effect Size	Standard Error of Natural Log of Effect Size	Covariates Adjusted for in Source
Bui	2018	Liver cancer	Male	Non-drinkers	Moderate drinkers	0.13	0.03	representativeness, age, sex, bmi, education, income, physical activity, smoking, menopause, other drug use, dietary factors, other, hypertension, diabetes, vitamin deficiency, other diagnosed disease, APOE, family history, hypercholesterolemia
Bui	2018	Liver cancer	Male	Non-drinkers	Moderate drinkers	0.10	0.06	representativeness, age, sex, bmi, education, income, physical activity, smoking, menopause, other drug use, dietary factors, other, hypertension, diabetes, vitamin deficiency, other diagnosed disease, APOE, family history, hypercholesterolemia
Bui	2018	Liver cancer	Male	Non-drinkers	Heavy drinkers	0.37	0.03	representativeness, age, sex, bmi, education, income, physical activity, smoking, menopause, other drug use, dietary factors, other, hypertension, diabetes, vitamin deficiency, other diagnosed disease, APOE, family history, hypercholesterolemia
Cho	2013	Liver cancer	Both	Non-drinkers	Moderate drinkers	-0.04	0.05	representativeness, age, sex, bmi, education, income, physical activity, smoking, menopause, other drug use, dietary factors, other, hypertension, diabetes, vitamin deficiency, other diagnosed disease, APOE, family history, hypercholesterolemia
Cococcia	2017	Liver cancer	Both	Light drinkers	Moderate drinkers	1.14	0.52	representativeness, age, sex, bmi, education, income, physical activity, smoking, menopause, other drug use, dietary factors, other, hypertension, diabetes, vitamin deficiency, other diagnosed disease, APOE, family history, hypercholesterolemia
Floud	2001	Liver cancer	Female	Light drinkers	Light drinkers	0.05	0.06	representativeness, age, sex, bmi, education, income, physical activity, smoking, menopause, other drug use, dietary factors, other, hypertension, diabetes, vitamin deficiency, other diagnosed disease, APOE, family history, hypercholesterolemia
Floud	2001	Liver cancer	Female	Light drinkers	Light drinkers	0.06	0.06	representativeness, age, sex, bmi, education, income, physical activity, smoking, menopause, other drug use, dietary factors, other, hypertension, diabetes, vitamin deficiency, other diagnosed disease, APOE, family history, hypercholesterolemia
Floud	2001	Liver cancer	Female	Light drinkers	Moderate drinkers	0.17	0.10	representativeness, age, sex, bmi, education, income, physical activity, smoking, menopause, other drug use, dietary factors, other, hypertension, diabetes, vitamin deficiency, other diagnosed disease, APOE, family history, hypercholesterolemia
Goodman	1989	Liver cancer	Male	Non-drinkers	Light drinkers	0.09	0.26	representativeness, age, sex, bmi, education, income, physical activity, smoking, menopause, other drug use, dietary factors, other, hypertension, diabetes, vitamin deficiency, other diagnosed disease, APOE, family history, hypercholesterolemia
Jang	2008	Liver cancer	Both	Non-drinkers	Heavy drinkers	0.42	0.27	representativeness, age, sex, bmi, education, income, physical activity, smoking, menopause, other drug use, dietary factors, other, hypertension, diabetes, vitamin deficiency, other diagnosed disease, APOE, family history, hypercholesterolemia
Jang	2008	Liver cancer	Both	Non-drinkers	Heavy drinkers	0.88	0.29	representativeness, age, sex, bmi, education, income, physical activity, smoking, menopause, other drug use, dietary factors, other, hypertension, diabetes, vitamin deficiency, other diagnosed disease, APOE, family history, hypercholesterolemia
Jang	2008	Liver cancer	Both	Non-drinkers	Moderate drinkers	-0.08	0.18	representativeness, age, sex, bmi, education, income, physical activity, smoking, menopause, other drug use, dietary factors, other, hypertension, diabetes, vitamin deficiency, other diagnosed disease, APOE, family history, hypercholesterolemia

Table S10. Summary of Data Inputs

Author	Year	Health outcome	Sex	Reference	Exposed group	Natural Log of Effect Size	Standard Error of Natural Log of Effect Size	Covariates Adjusted for in Source
Jang	2008	Liver cancer	Both	Non-drinkers	Heavy drinkers	0.83	0.24	representativeness, age, sex, bmi, education, income, physical activity, smoking, menopause, other drug use, dietary factors, other, hypertension, diabetes, vitamin deficiency, other diagnosed disease, APOE, family history, hypercholesterolemia
Jee	2002	Liver cancer	Both	Non-drinkers	Moderate drinkers	0.10	0.09	age, sex, bmi, education, income, physical activity, smoking, other, diabetes
Jee	2002	Liver cancer	Both	Non-drinkers	Light drinkers	0.00	0.05	age, sex, bmi, education, income, physical activity, smoking, other, diabetes
Joshi	2004	Liver cancer	Both	Non-drinkers	Heavy drinkers	0.09	0.18	age, sex, bmi, education, income, physical activity, smoking, other
Joshi	2004	Liver cancer	Both	Non-drinkers	Heavy drinkers	-0.05	0.12	age, sex, bmi, education, income, physical activity, smoking, other
Joshi	2004	Liver cancer	Both	Non-drinkers	Moderate drinkers	-0.16	0.11	age, sex, bmi, education, income, physical activity, smoking, other
Jung	2004	Liver cancer	Both	Light drinkers	Heavy drinkers	0.69	0.50	representativeness, age, sex, bmi, education, income, physical activity, smoking, menopause, other drug use, dietary factors, other, hypertension, diabetes, vitamin deficiency, other diagnosed disease, APOE, family history, hypercholesterolemia
Koh	2007	Liver cancer	Both	Non-drinkers	Light drinkers	0.01	0.38	age, sex, bmi, education, income, physical activity, other drug use, diabetes
Minami	2018	Liver cancer	Both	Moderate drinkers	Heavy drinkers	0.94	0.41	representativeness, age, sex, bmi, education, income, physical activity, smoking, menopause, other drug use, dietary factors, other, hypertension, diabetes, vitamin deficiency, other diagnosed disease, APOE, family history, hypercholesterolemia
Ogimoto	1999	Liver cancer	Male	Non-drinkers	Light drinkers	0.03	1.24	representativeness, age, sex, bmi, education, income, physical activity, smoking, menopause, other drug use, dietary factors, other, hypertension, diabetes, vitamin deficiency, other diagnosed disease, APOE, family history, hypercholesterolemia
Ogimoto	1999	Liver cancer	Male	Non-drinkers	Light drinkers	0.60	0.60	representativeness, age, sex, bmi, education, income, physical activity, smoking, menopause, other drug use, dietary factors, other, hypertension, diabetes, vitamin deficiency, other diagnosed disease, APOE, family history, hypercholesterolemia
Ogimoto	1999	Liver cancer	Male	Non-drinkers	Light drinkers	-0.04	0.67	representativeness, age, sex, bmi, education, income, physical activity, smoking, menopause, other drug use, dietary factors, other, hypertension, diabetes, vitamin deficiency, other diagnosed disease, APOE, family history, hypercholesterolemia
Ogimoto	1999	Liver cancer	Male	Non-drinkers	Light drinkers	-0.58	0.44	representativeness, age, sex, bmi, education, income, physical activity, smoking, menopause, other drug use, dietary factors, other, hypertension, diabetes, vitamin deficiency, other diagnosed disease, APOE, family history, hypercholesterolemia
Ohishi	2002	Liver cancer	Both	Non-drinkers	Light drinkers	0.24	0.42	age, sex, bmi, education, income, physical activity, smoking, other drug use, other, diabetes, other diagnosed disease
Ohishi	2002	Liver cancer	Both	Non-drinkers	Moderate drinkers	0.02	0.56	age, sex, bmi, education, income, physical activity, smoking, other drug use, other, diabetes, other diagnosed disease

Table S10. Summary of Data Inputs

Author	Year	Health outcome	Sex	Reference	Exposed group	Natural Log of Effect Size	Standard Error of Natural Log of Effect Size	Covariates Adjusted for in Source
Ohishi	2002	Liver cancer	Both	Non-drinkers	Light drinkers	0.62	0.31	representativeness, age, sex, bmi, education, income, physical activity, smoking, menopause, other drug use, dietary factors, other, hypertension, diabetes, vitamin deficiency, other diagnosed disease, APOE, family history, hypercholesterolemia
Ozasa	2005	Liver cancer	Both	Non-drinkers	Heavy drinkers	0.02	1.01	age, sex, bmi, education, income, physical activity, other
Persson	2006	Liver cancer	Both	Light drinkers	Moderate drinkers	-0.03	0.16	representativeness, age, sex, bmi, education, income, physical activity, smoking, menopause, other drug use, dietary factors, other, hypertension, diabetes, vitamin deficiency, other diagnosed disease, APOE, family history, hypercholesterolemia
Petrick	2010	Liver cancer	Both	Non-drinkers	Moderate drinkers	-0.34	0.10	representativeness, age, sex, bmi, education, income, physical activity, smoking, menopause, other drug use, dietary factors, other, hypertension, diabetes, vitamin deficiency, other diagnosed disease, APOE, family history, hypercholesterolemia
Petrick	2010	Liver cancer	Both	Non-drinkers	Heavy drinkers	0.04	0.14	representativeness, age, sex, bmi, education, income, physical activity, smoking, menopause, other drug use, dietary factors, other, hypertension, diabetes, vitamin deficiency, other diagnosed disease, APOE, family history, hypercholesterolemia
Petrick	2010	Liver cancer	Both	Non-drinkers	Heavy drinkers	0.00	0.20	representativeness, age, sex, bmi, education, income, physical activity, smoking, menopause, other drug use, dietary factors, other, hypertension, diabetes, vitamin deficiency, other diagnosed disease, APOE, family history, hypercholesterolemia
Pyong	1992	Liver cancer	Both	Non-drinkers	Heavy drinkers	0.03	1.57	age, sex, bmi, education, income, physical activity
Pyong	1992	Liver cancer	Both	Non-drinkers	Moderate drinkers	-0.02	0.42	age, sex, bmi, education, income, physical activity
Sarich	2013	Liver cancer	Both	Light drinkers	Light drinkers	0.09	0.35	representativeness, age, sex, bmi, education, income, physical activity, smoking, menopause, other drug use, dietary factors, other, hypertension, diabetes, vitamin deficiency, other diagnosed disease, APOE, family history, hypercholesterolemia
Sarich	2013	Liver cancer	Both	Light drinkers	Moderate drinkers	0.17	0.38	representativeness, age, sex, bmi, education, income, physical activity, smoking, menopause, other drug use, dietary factors, other, hypertension, diabetes, vitamin deficiency, other diagnosed disease, APOE, family history, hypercholesterolemia
Schwartz	2009	Liver cancer	Male	Light drinkers	Moderate drinkers	0.42	0.18	representativeness, age, sex, bmi, education, income, physical activity, smoking, menopause, other drug use, dietary factors, other, hypertension, diabetes, vitamin deficiency, other diagnosed disease, APOE, family history, hypercholesterolemia
Shimazu	2004	Liver cancer	Male	Light drinkers	Light drinkers	0.04	0.26	representativeness, age, sex, bmi, education, income, physical activity, smoking, menopause, other drug use, dietary factors, other, hypertension, diabetes, vitamin deficiency, other diagnosed disease, APOE, family history, hypercholesterolemia
Stemhagen	1980	Liver cancer	Both	Non-drinkers	Heavy drinkers	0.92	0.49	age, sex, bmi, education, income, physical activity, smoking
Stemhagen	1980	Liver cancer	Both	Non-drinkers	Light drinkers	0.01	0.38	age, sex, bmi, education, income, physical activity, smoking
Yi	2005	Liver cancer	Both	Non-drinkers	Light drinkers	0.06	1.07	age, sex, bmi, education, income, physical activity, smoking, other

Table S10. Summary of Data Inputs

Author	Year	Health outcome	Sex	Reference	Exposed group	Natural Log of Effect Size	Standard Error of Natural Log of Effect Size	Covariates Adjusted for in Source
Yi	2005	Liver cancer	Both	Non-drinkers	Heavy drinkers	-0.06	0.46	age, sex, bmi, education, income, physical activity, smoking, other
Yi	2005	Liver cancer	Both	Non-drinkers	Light drinkers	-0.09	0.45	age, sex, bmi, education, income, physical activity, smoking, other
Yi	2013	Liver cancer	Both	Non-drinkers	Light drinkers	-0.09	0.05	representativeness, age, sex, bmi, education, income, physical activity, smoking, menopause, other drug use, dietary factors, other, hypertension, diabetes, vitamin deficiency, other diagnosed disease, APOE, family history, hypercholesterolemia
Yi	2013	Liver cancer	Both	Non-drinkers	Moderate drinkers	0.04	0.06	representativeness, age, sex, bmi, education, income, physical activity, smoking, menopause, other drug use, dietary factors, other, hypertension, diabetes, vitamin deficiency, other diagnosed disease, APOE, family history, hypercholesterolemia
Yi	2013	Liver cancer	Both	Non-drinkers	Heavy drinkers	0.38	0.07	representativeness, age, sex, bmi, education, income, physical activity, smoking, menopause, other drug use, dietary factors, other, hypertension, diabetes, vitamin deficiency, other diagnosed disease, APOE, family history, hypercholesterolemia
Yuan	2001	Liver cancer	Both	Non-drinkers	Moderate drinkers	0.09	0.19	age, sex, bmi, education, income, physical activity
Zhang	1995	Liver cancer	Both	Non-drinkers	Heavy drinkers	0.24	0.45	age, sex, bmi, education, income, physical activity
Almirall	2000	Lower respiratory infections	Male	Non-drinkers	Heavy drinkers	0.85	0.37	representativeness, age, sex, bmi, education, income, physical activity, smoking, menopause, other drug use, dietary factors, other, hypertension, diabetes, vitamin deficiency, other diagnosed disease, APOE, family history, hypercholesterolemia
Almirall	2000	Lower respiratory infections	Female	Non-drinkers	Moderate drinkers	0.09	0.44	representativeness, age, sex, bmi, education, income, physical activity, smoking, menopause, other drug use, dietary factors, other, hypertension, diabetes, vitamin deficiency, other diagnosed disease, APOE, family history, hypercholesterolemia
Almirall	2000	Lower respiratory infections	Male	Non-drinkers	Heavy drinkers	0.85	0.37	representativeness, age, sex, bmi, education, income, physical activity, smoking, menopause, other drug use, dietary factors, other, hypertension, diabetes, vitamin deficiency, other diagnosed disease, APOE, family history, hypercholesterolemia
Almirall	2000	Lower respiratory infections	Female	Non-drinkers	Moderate drinkers	0.09	0.44	representativeness, age, sex, bmi, education, income, physical activity, smoking, menopause, other drug use, dietary factors, other, hypertension, diabetes, vitamin deficiency, other diagnosed disease, APOE, family history, hypercholesterolemia
Almirall	2000	Lower respiratory infections	Male	Non-drinkers	Light drinkers	-0.05	0.12	representativeness, age, sex, bmi, education, income, physical activity, smoking, menopause, other drug use, dietary factors, other, hypertension, diabetes, vitamin deficiency, other diagnosed disease, APOE, family history, hypercholesterolemia
Almirall	2000	Lower respiratory infections	Male	Non-drinkers	Light drinkers	-0.05	0.12	representativeness, age, sex, bmi, education, income, physical activity, smoking, menopause, other drug use, dietary factors, other, hypertension, diabetes, vitamin deficiency, other diagnosed disease, APOE, family history, hypercholesterolemia
Almirall	2000	Lower respiratory infections	Female	Non-drinkers	Light drinkers	-0.02	0.12	representativeness, age, sex, bmi, education, income, physical activity, smoking, menopause, other drug use, dietary factors, other, hypertension, diabetes, vitamin deficiency, other diagnosed disease, APOE, family history, hypercholesterolemia

Table S10. Summary of Data Inputs

Author	Year	Health outcome	Sex	Reference	Exposed group	Natural Log of Effect Size	Standard Error of Natural Log of Effect Size	Covariates Adjusted for in Source
Almirall	2000	Lower respiratory infections	Female	Non-drinkers	Light drinkers	-0.02	0.12	representativeness, age, sex, bmi, education, income, physical activity, smoking, menopause, other drug use, dietary factors, other, hypertension, diabetes, vitamin deficiency, other diagnosed disease, APOE, family history, hypercholesterolemia
Almirall	2000	Lower respiratory infections	Male	Non-drinkers	Heavy drinkers	0.85	0.37	representativeness, exposure_quality, outcome_quality, confounder_quality, reverse_causation, selection_bias, sex
Almirall	2000	Lower respiratory infections	Female	Non-drinkers	Moderate drinkers	0.09	0.44	representativeness, exposure_quality, outcome_quality, confounder_quality, reverse_causation, selection_bias, sex
Almirall	2000	Lower respiratory infections	Male	Non-drinkers	Light drinkers	-0.05	0.12	representativeness, exposure_quality, outcome_quality, confounder_quality, reverse_causation, selection_bias, sex
Almirall	2000	Lower respiratory infections	Female	Non-drinkers	Light drinkers	-0.02	0.12	representativeness, exposure_quality, outcome_quality, confounder_quality, reverse_causation, selection_bias, sex
Baik	1992	Lower respiratory infections	Male	Non-drinkers	Moderate drinkers	0.02	0.37	representativeness, exposure_quality, outcome_quality, confounder_quality, reverse_causation, selection_bias, sex
Baik	1992	Lower respiratory infections	Male	Non-drinkers	Light drinkers	0.04	0.21	representativeness, exposure_quality, outcome_quality, confounder_quality, reverse_causation, selection_bias, sex
Baik	1992	Lower respiratory infections	Male	Non-drinkers	Light drinkers	-0.09	0.13	representativeness, exposure_quality, outcome_quality, confounder_quality, reverse_causation, selection_bias, sex
Baik	1992	Lower respiratory infections	Male	Non-drinkers	Light drinkers	0.04	0.21	representativeness, age, sex, bmi, education, income, physical activity, smoking, menopause, other drug use, dietary factors, other, hypertension, diabetes, vitamin deficiency, other diagnosed disease, APOE, family history, hypercholesterolemia
Baik	1992	Lower respiratory infections	Female	Non-drinkers	Light drinkers	-0.09	0.13	representativeness, age, sex, bmi, education, income, physical activity, smoking, menopause, other drug use, dietary factors, other, hypertension, diabetes, vitamin deficiency, other diagnosed disease, APOE, family history, hypercholesterolemia
Baik	1992	Lower respiratory infections	Female	Non-drinkers	Moderate drinkers	0.02	0.37	representativeness, age, sex, bmi, education, income, physical activity, smoking, menopause, other drug use, dietary factors, other, hypertension, diabetes, vitamin deficiency, other diagnosed disease, APOE, family history, hypercholesterolemia
Conway	2014	Other pharynx cancer	Both	Non-drinkers	Light drinkers	0.04	0.03	representativeness, age, sex, bmi, education, income, physical activity, smoking, menopause, other drug use, dietary factors, other, hypertension, diabetes, vitamin deficiency, other diagnosed disease, APOE, family history, hypercholesterolemia
Lubin	2011	Other pharynx cancer	Male	Light drinkers	Moderate drinkers	0.38	0.10	representativeness, age, sex, bmi, education, income, physical activity, smoking, menopause, other drug use, dietary factors, other, hypertension, diabetes, vitamin deficiency, other diagnosed disease, APOE, family history, hypercholesterolemia

Table S10. Summary of Data Inputs

Author	Year	Health outcome	Sex	Reference	Exposed group	Natural Log of Effect Size	Standard Error of Natural Log of Effect Size	Covariates Adjusted for in Source
Tota	2015	Other pharynx cancer	Both	Light drinkers	Moderate drinkers	0.60	0.27	representativeness, age, sex, bmi, education, income, physical activity, smoking, menopause, other drug use, dietary factors, other, hypertension, diabetes, vitamin deficiency, other diagnosed disease, APOE, family history, hypercholesterolemia
Turati	2008	Other pharynx cancer	Both	Light drinkers	Moderate drinkers	-0.08	0.21	representativeness, age, sex, bmi, education, income, physical activity, smoking, menopause, other drug use, dietary factors, other, hypertension, diabetes, vitamin deficiency, other diagnosed disease, APOE, family history, hypercholesterolemia
Turati	2008	Other pharynx cancer	Both	Light drinkers	Heavy drinkers	0.24	0.19	representativeness, age, sex, bmi, education, income, physical activity, smoking, menopause, other drug use, dietary factors, other, hypertension, diabetes, vitamin deficiency, other diagnosed disease, APOE, family history, hypercholesterolemia
Werbrouck	2006	Other pharynx cancer	Both	Non-drinkers	Moderate drinkers	0.42	0.56	age, sex, bmi, education, income, physical activity, smoking
Bui	2018	Pancreatic cancer	Male	Non-drinkers	Light drinkers	-0.03	0.04	representativeness, age, sex, bmi, education, income, physical activity, smoking, menopause, other drug use, dietary factors, other, hypertension, diabetes, vitamin deficiency, other diagnosed disease, APOE, family history, hypercholesterolemia
Bui	2018	Pancreatic cancer	Male	Non-drinkers	Light drinkers	0.03	0.04	representativeness, age, sex, bmi, education, income, physical activity, smoking, menopause, other drug use, dietary factors, other, hypertension, diabetes, vitamin deficiency, other diagnosed disease, APOE, family history, hypercholesterolemia
Bui	2018	Pancreatic cancer	Male	Non-drinkers	Moderate drinkers	0.04	0.06	representativeness, age, sex, bmi, education, income, physical activity, smoking, menopause, other drug use, dietary factors, other, hypertension, diabetes, vitamin deficiency, other diagnosed disease, APOE, family history, hypercholesterolemia
Bui	2018	Pancreatic cancer	Male	Non-drinkers	Heavy drinkers	0.09	0.07	representativeness, age, sex, bmi, education, income, physical activity, smoking, menopause, other drug use, dietary factors, other, hypertension, diabetes, vitamin deficiency, other diagnosed disease, APOE, family history, hypercholesterolemia
Floud	2001	Pancreatic cancer	Female	Light drinkers	Light drinkers	0.02	0.04	representativeness, age, sex, bmi, education, income, physical activity, smoking, menopause, other drug use, dietary factors, other, hypertension, diabetes, vitamin deficiency, other diagnosed disease, APOE, family history, hypercholesterolemia
Floud	2001	Pancreatic cancer	Female	Light drinkers	Light drinkers	0.08	0.03	representativeness, age, sex, bmi, education, income, physical activity, smoking, menopause, other drug use, dietary factors, other, hypertension, diabetes, vitamin deficiency, other diagnosed disease, APOE, family history, hypercholesterolemia
Floud	2001	Pancreatic cancer	Female	Light drinkers	Moderate drinkers	0.08	0.03	representativeness, age, sex, bmi, education, income, physical activity, smoking, menopause, other drug use, dietary factors, other, hypertension, diabetes, vitamin deficiency, other diagnosed disease, APOE, family history, hypercholesterolemia
Floud	2001	Pancreatic cancer	Female	Light drinkers	Light drinkers	0.08	0.03	representativeness, age, sex, bmi, education, income, physical activity, smoking, menopause, other drug use, dietary factors, other, hypertension, diabetes, vitamin deficiency, other diagnosed disease, APOE, family history, hypercholesterolemia

Table S10. Summary of Data Inputs

Author	Year	Health outcome	Sex	Reference	Exposed group	Natural Log of Effect Size	Standard Error of Natural Log of Effect Size	Covariates Adjusted for in Source
Floud	2001	Pancreatic cancer	Female	Light drinkers	Moderate drinkers	0.08	0.03	representativeness, age, sex, bmi, education, income, physical activity, smoking, menopause, other drug use, dietary factors, other, hypertension, diabetes, vitamin deficiency, other diagnosed disease, APOE, family history, hypercholesterolemia
Jayasekara	2015	Pancreatic cancer	Both	Non-drinkers	Moderate drinkers	-0.06	0.26	representativeness, age, sex, bmi, education, income, physical activity, smoking, menopause, other drug use, dietary factors, other, hypertension, diabetes, vitamin deficiency, other diagnosed disease, APOE, family history, hypercholesterolemia
Kono	1983	Pancreatic cancer	Both	Non-drinkers	Light drinkers	-0.92	0.94	age, sex, bmi, education, income, physical activity, smoking
Koyanagi	2013	Pancreatic cancer	Both	Non-drinkers	Light drinkers	-0.01	0.15	representativeness, age, sex, bmi, education, income, physical activity, smoking, menopause, other drug use, dietary factors, other, hypertension, diabetes, vitamin deficiency, other diagnosed disease, APOE, family history, hypercholesterolemia
Koyanagi	2013	Pancreatic cancer	Both	Non-drinkers	Light drinkers	-0.01	0.15	representativeness, age, sex, bmi, education, income, physical activity, smoking, menopause, other drug use, dietary factors, other, hypertension, diabetes, vitamin deficiency, other diagnosed disease, APOE, family history, hypercholesterolemia
Naudin	2000	Pancreatic cancer	Female	Light drinkers	Moderate drinkers	0.06	0.12	representativeness, age, sex, bmi, education, income, physical activity, smoking, menopause, other drug use, dietary factors, other, hypertension, diabetes, vitamin deficiency, other diagnosed disease, APOE, family history, hypercholesterolemia
Naudin	2000	Pancreatic cancer	Female	Light drinkers	Heavy drinkers	-0.07	0.35	representativeness, age, sex, bmi, education, income, physical activity, smoking, menopause, other drug use, dietary factors, other, hypertension, diabetes, vitamin deficiency, other diagnosed disease, APOE, family history, hypercholesterolemia
Okita	1995	Pancreatic cancer	Male	Non-drinkers	Light drinkers	-0.03	0.23	representativeness, age, sex, bmi, education, income, physical activity, smoking, menopause, other drug use, dietary factors, other, hypertension, diabetes, vitamin deficiency, other diagnosed disease, APOE, family history, hypercholesterolemia
Okita	1995	Pancreatic cancer	Female	Non-drinkers	Light drinkers	0.26	0.21	representativeness, age, sex, bmi, education, income, physical activity, smoking, menopause, other drug use, dietary factors, other, hypertension, diabetes, vitamin deficiency, other diagnosed disease, APOE, family history, hypercholesterolemia
Ozasa	2005	Pancreatic cancer	Both	Non-drinkers	Heavy drinkers	0.05	1.02	age, sex, bmi, education, income, physical activity, other
Ozasa	2005	Pancreatic cancer	Both	Non-drinkers	Moderate drinkers	0.13	0.21	age, sex, bmi, education, income, physical activity, other
Ozasa	2005	Pancreatic cancer	Both	Non-drinkers	Heavy drinkers	0.42	0.23	age, sex, bmi, education, income, physical activity, other
Pang	2008	Pancreatic cancer	Male	Light drinkers	Heavy drinkers	0.13	0.21	representativeness, age, sex, bmi, education, income, physical activity, smoking, menopause, other drug use, dietary factors, other, hypertension, diabetes, vitamin deficiency, other diagnosed disease, APOE, family history, hypercholesterolemia
Pang	2008	Pancreatic cancer	Male	Light drinkers	Light drinkers	0.52	0.17	representativeness, age, sex, bmi, education, income, physical activity, smoking, menopause, other drug use, dietary factors, other, hypertension, diabetes, vitamin deficiency, other diagnosed disease, APOE, family history, hypercholesterolemia

Table S10. Summary of Data Inputs

Author	Year	Health outcome	Sex	Reference	Exposed group	Natural Log of Effect Size	Standard Error of Natural Log of Effect Size	Covariates Adjusted for in Source
Park	2017	Pancreatic cancer	Both	Non-drinkers	Light drinkers	-0.04	0.03	representativeness, age, sex, bmi, education, income, physical activity, smoking, menopause, other drug use, dietary factors, other, hypertension, diabetes, vitamin deficiency, other diagnosed disease, APOE, family history, hypercholesterolemia
Park	2017	Pancreatic cancer	Both	Non-drinkers	Moderate drinkers	0.04	0.05	representativeness, age, sex, bmi, education, income, physical activity, smoking, menopause, other drug use, dietary factors, other, hypertension, diabetes, vitamin deficiency, other diagnosed disease, APOE, family history, hypercholesterolemia
Sarich	2013	Pancreatic cancer	Both	Light drinkers	Heavy drinkers	-0.08	0.24	representativeness, age, sex, bmi, education, income, physical activity, smoking, menopause, other drug use, dietary factors, other, hypertension, diabetes, vitamin deficiency, other diagnosed disease, APOE, family history, hypercholesterolemia
Sarich	2013	Pancreatic cancer	Both	Light drinkers	Heavy drinkers	-0.08	0.31	representativeness, age, sex, bmi, education, income, physical activity, smoking, menopause, other drug use, dietary factors, other, hypertension, diabetes, vitamin deficiency, other diagnosed disease, APOE, family history, hypercholesterolemia
Sarich	2013	Pancreatic cancer	Both	Light drinkers	Light drinkers	0.09	0.16	representativeness, age, sex, bmi, education, income, physical activity, smoking, menopause, other drug use, dietary factors, other, hypertension, diabetes, vitamin deficiency, other diagnosed disease, APOE, family history, hypercholesterolemia
Sarich	2013	Pancreatic cancer	Both	Light drinkers	Light drinkers	0.09	0.18	representativeness, age, sex, bmi, education, income, physical activity, smoking, menopause, other drug use, dietary factors, other, hypertension, diabetes, vitamin deficiency, other diagnosed disease, APOE, family history, hypercholesterolemia
Yallew	2009	Pancreatic cancer	Both	Non-drinkers	Heavy drinkers	-0.06	0.24	representativeness, age, sex, bmi, education, income, physical activity, smoking, menopause, other drug use, dietary factors, other, hypertension, diabetes, vitamin deficiency, other diagnosed disease, APOE, family history, hypercholesterolemia
Yallew	2009	Pancreatic cancer	Both	Light drinkers	Heavy drinkers	-0.06	0.24	representativeness, age, sex, bmi, education, income, physical activity, smoking, menopause, other drug use, dietary factors, other, hypertension, diabetes, vitamin deficiency, other diagnosed disease, APOE, family history, hypercholesterolemia
Yallew	2009	Pancreatic cancer	Both	Non-drinkers	Heavy drinkers	-0.06	0.24	representativeness, age, sex, bmi, education, income, physical activity, smoking, menopause, other drug use, dietary factors, other, hypertension, diabetes, vitamin deficiency, other diagnosed disease, APOE, family history, hypercholesterolemia
Yallew	2009	Pancreatic cancer	Both	Light drinkers	Heavy drinkers	-0.06	0.24	representativeness, age, sex, bmi, education, income, physical activity, smoking, menopause, other drug use, dietary factors, other, hypertension, diabetes, vitamin deficiency, other diagnosed disease, APOE, family history, hypercholesterolemia
Yallew	2009	Pancreatic cancer	Both	Non-drinkers	Light drinkers	0.00	0.23	representativeness, age, sex, bmi, education, income, physical activity, smoking, menopause, other drug use, dietary factors, other, hypertension, diabetes, vitamin deficiency, other diagnosed disease, APOE, family history, hypercholesterolemia
Yallew	2009	Pancreatic cancer	Both	Non-drinkers	Light drinkers	0.00	0.24	representativeness, age, sex, bmi, education, income, physical activity, smoking, menopause, other drug use, dietary factors, other, hypertension, diabetes, vitamin deficiency, other diagnosed disease, APOE, family history, hypercholesterolemia

Table S10. Summary of Data Inputs

Author	Year	Health outcome	Sex	Reference	Exposed group	Natural Log of Effect Size	Standard Error of Natural Log of Effect Size	Covariates Adjusted for in Source
Yallew	2009	Pancreatic cancer	Both	Light drinkers	Heavy drinkers	0.00	0.23	representativeness, age, sex, bmi, education, income, physical activity, smoking, menopause, other drug use, dietary factors, other, hypertension, diabetes, vitamin deficiency, other diagnosed disease, APOE, family history, hypercholesterolemia
Yallew	2009	Pancreatic cancer	Both	Light drinkers	Heavy drinkers	0.00	0.24	representativeness, age, sex, bmi, education, income, physical activity, smoking, menopause, other drug use, dietary factors, other, hypertension, diabetes, vitamin deficiency, other diagnosed disease, APOE, family history, hypercholesterolemia
Yallew	2009	Pancreatic cancer	Both	Non-drinkers	Light drinkers	0.00	0.23	representativeness, age, sex, bmi, education, income, physical activity, smoking, menopause, other drug use, dietary factors, other, hypertension, diabetes, vitamin deficiency, other diagnosed disease, APOE, family history, hypercholesterolemia
Yallew	2009	Pancreatic cancer	Both	Non-drinkers	Light drinkers	0.00	0.24	representativeness, age, sex, bmi, education, income, physical activity, smoking, menopause, other drug use, dietary factors, other, hypertension, diabetes, vitamin deficiency, other diagnosed disease, APOE, family history, hypercholesterolemia
Yallew	2009	Pancreatic cancer	Both	Light drinkers	Heavy drinkers	0.00	0.23	representativeness, age, sex, bmi, education, income, physical activity, smoking, menopause, other drug use, dietary factors, other, hypertension, diabetes, vitamin deficiency, other diagnosed disease, APOE, family history, hypercholesterolemia
Yallew	2009	Pancreatic cancer	Both	Light drinkers	Heavy drinkers	0.00	0.24	representativeness, age, sex, bmi, education, income, physical activity, smoking, menopause, other drug use, dietary factors, other, hypertension, diabetes, vitamin deficiency, other diagnosed disease, APOE, family history, hypercholesterolemia
Yallew	2009	Pancreatic cancer	Both	Non-drinkers	Light drinkers	0.10	0.23	representativeness, age, sex, bmi, education, income, physical activity, smoking, menopause, other drug use, dietary factors, other, hypertension, diabetes, vitamin deficiency, other diagnosed disease, APOE, family history, hypercholesterolemia
Yallew	2009	Pancreatic cancer	Both	Light drinkers	Light drinkers	0.10	0.23	representativeness, age, sex, bmi, education, income, physical activity, smoking, menopause, other drug use, dietary factors, other, hypertension, diabetes, vitamin deficiency, other diagnosed disease, APOE, family history, hypercholesterolemia
Yallew	2009	Pancreatic cancer	Both	Non-drinkers	Light drinkers	0.10	0.23	representativeness, age, sex, bmi, education, income, physical activity, smoking, menopause, other drug use, dietary factors, other, hypertension, diabetes, vitamin deficiency, other diagnosed disease, APOE, family history, hypercholesterolemia
Yallew	2009	Pancreatic cancer	Both	Light drinkers	Light drinkers	0.10	0.23	representativeness, age, sex, bmi, education, income, physical activity, smoking, menopause, other drug use, dietary factors, other, hypertension, diabetes, vitamin deficiency, other diagnosed disease, APOE, family history, hypercholesterolemia
Kristiansen	2003	Pancreatitis	Both	Non-drinkers	Light drinkers	0.10	0.18	age, sex, bmi, education, income, physical activity, smoking
Kristiansen	2003	Pancreatitis	Both	Non-drinkers	Moderate drinkers	0.26	0.25	age, sex, bmi, education, income, physical activity, smoking
Kristiansen	2003	Pancreatitis	Both	Non-drinkers	Moderate drinkers	0.26	0.29	age, sex, bmi, education, income, physical activity, smoking

Table S10. Summary of Data Inputs

Author	Year	Health outcome	Sex	Reference	Exposed group	Natural Log of Effect Size	Standard Error of Natural Log of Effect Size	Covariates Adjusted for in Source
Kume	2010	Pancreatitis	Both	Non-drinkers	Light drinkers	0.00	0.18	representativeness, age, sex, bmi, education, income, physical activity, smoking, menopause, other drug use, dietary factors, other, hypertension, diabetes, vitamin deficiency, other diagnosed disease, APOE, family history, hypercholesterolemia
Prizment	2004	Pancreatitis	Female	Light drinkers	Light drinkers	-0.16	0.21	representativeness, age, sex, bmi, education, income, physical activity, smoking, menopause, other drug use, dietary factors, other, hypertension, diabetes, vitamin deficiency, other diagnosed disease, APOE, family history, hypercholesterolemia
Setiawan	2012	Pancreatitis	Male	Non-drinkers	Light drinkers	-0.16	0.10	representativeness, age, sex, bmi, education, income, physical activity, smoking, menopause, other drug use, dietary factors, other, hypertension, diabetes, vitamin deficiency, other diagnosed disease, APOE, family history, hypercholesterolemia
Setiawan	2012	Pancreatitis	Male	Non-drinkers	Heavy drinkers	0.06	0.18	representativeness, age, sex, bmi, education, income, physical activity, smoking, menopause, other drug use, dietary factors, other, hypertension, diabetes, vitamin deficiency, other diagnosed disease, APOE, family history, hypercholesterolemia
Setiawan	2012	Pancreatitis	Female	Non-drinkers	Light drinkers	-0.37	0.23	representativeness, age, sex, bmi, education, income, physical activity, smoking, menopause, other drug use, dietary factors, other, hypertension, diabetes, vitamin deficiency, other diagnosed disease, APOE, family history, hypercholesterolemia
Yang	2007	Pancreatitis	Both	Non-drinkers	Light drinkers	-0.09	0.49	representativeness, age, sex, bmi, education, income, physical activity, smoking, menopause, other drug use, dietary factors, other, hypertension, diabetes, vitamin deficiency, other diagnosed disease, APOE, family history, hypercholesterolemia
Albertsen	1994	Prostate cancer	Male	Light drinkers	Moderate drinkers	-0.09	0.23	representativeness, age, sex, bmi, education, income, physical activity, smoking, menopause, other drug use, dietary factors, other, hypertension, diabetes, vitamin deficiency, other diagnosed disease, APOE, family history, hypercholesterolemia
Albertsen	1994	Prostate cancer	Male	Light drinkers	Heavy drinkers	-0.07	0.23	representativeness, age, sex, bmi, education, income, physical activity, smoking, menopause, other drug use, dietary factors, other, hypertension, diabetes, vitamin deficiency, other diagnosed disease, APOE, family history, hypercholesterolemia
Albertsen	1994	Prostate cancer	Male	Light drinkers	Light drinkers	0.03	0.18	representativeness, age, sex, bmi, education, income, physical activity, smoking, menopause, other drug use, dietary factors, other, hypertension, diabetes, vitamin deficiency, other diagnosed disease, APOE, family history, hypercholesterolemia
Albertsen	1994	Prostate cancer	Male	Non-drinkers	Moderate drinkers	-0.02	0.23	representativeness, age, sex, bmi, education, income, physical activity, smoking, menopause, other drug use, dietary factors, other, hypertension, diabetes, vitamin deficiency, other diagnosed disease, APOE, family history, hypercholesterolemia
Albertsen	1994	Prostate cancer	Male	Non-drinkers	Moderate drinkers	-0.08	0.40	representativeness, age, sex, bmi, education, income, physical activity, smoking, menopause, other drug use, dietary factors, other, hypertension, diabetes, vitamin deficiency, other diagnosed disease, APOE, family history, hypercholesterolemia
Albertsen	1994	Prostate cancer	Male	Non-drinkers	Light drinkers	-0.05	0.16	representativeness, age, sex, bmi, education, income, physical activity, smoking, menopause, other drug use, dietary factors, other, hypertension, diabetes, vitamin deficiency, other diagnosed disease, APOE, family history, hypercholesterolemia

Table S10. Summary of Data Inputs

Author	Year	Health outcome	Sex	Reference	Exposed group	Natural Log of Effect Size	Standard Error of Natural Log of Effect Size	Covariates Adjusted for in Source
Albertsen	1994	Prostate cancer	Male	Non-drinkers	Moderate drinkers	0.02	0.34	representativeness, age, sex, bmi, education, income, physical activity, smoking, menopause, other drug use, dietary factors, other, hypertension, diabetes, vitamin deficiency, other diagnosed disease, APOE, family history, hypercholesterolemia
Andersson	1991	Prostate cancer	Male	Non-drinkers	Light drinkers	0.10	0.32	representativeness, age, sex, bmi, education, income, physical activity, smoking, menopause, other drug use, dietary factors, other, hypertension, diabetes, vitamin deficiency, other diagnosed disease, APOE, family history, hypercholesterolemia
Baglietto	1994	Prostate cancer	Male	Non-drinkers	Light drinkers	-0.02	0.11	representativeness, age, sex, bmi, education, income, physical activity, smoking, menopause, other drug use, dietary factors, other, hypertension, diabetes, vitamin deficiency, other diagnosed disease, APOE, family history, hypercholesterolemia
Baglietto	1994	Prostate cancer	Male	Non-drinkers	Moderate drinkers	-0.04	0.13	representativeness, age, sex, bmi, education, income, physical activity, smoking, menopause, other drug use, dietary factors, other, hypertension, diabetes, vitamin deficiency, other diagnosed disease, APOE, family history, hypercholesterolemia
Baglietto	1994	Prostate cancer	Male	Non-drinkers	Heavy drinkers	-0.05	0.16	representativeness, age, sex, bmi, education, income, physical activity, smoking, menopause, other drug use, dietary factors, other, hypertension, diabetes, vitamin deficiency, other diagnosed disease, APOE, family history, hypercholesterolemia
Baglietto	1994	Prostate cancer	Male	Non-drinkers	Heavy drinkers	-0.06	0.17	representativeness, age, sex, bmi, education, income, physical activity, smoking, menopause, other drug use, dietary factors, other, hypertension, diabetes, vitamin deficiency, other diagnosed disease, APOE, family history, hypercholesterolemia
Breslow	1992	Prostate cancer	Male	Non-drinkers	Light drinkers	-0.03	0.19	representativeness, age, sex, bmi, education, income, physical activity, smoking, menopause, other drug use, dietary factors, other, hypertension, diabetes, vitamin deficiency, other diagnosed disease, APOE, family history, hypercholesterolemia
Breslow	1992	Prostate cancer	Male	Non-drinkers	Moderate drinkers	-0.04	0.23	representativeness, age, sex, bmi, education, income, physical activity, smoking, menopause, other drug use, dietary factors, other, hypertension, diabetes, vitamin deficiency, other diagnosed disease, APOE, family history, hypercholesterolemia
Breslow	1992	Prostate cancer	Male	Non-drinkers	Moderate drinkers	-0.16	0.37	representativeness, age, sex, bmi, education, income, physical activity, smoking, menopause, other drug use, dietary factors, other, hypertension, diabetes, vitamin deficiency, other diagnosed disease, APOE, family history, hypercholesterolemia
Chang	2002	Prostate cancer	Male	Non-drinkers	Light drinkers	0.10	0.14	representativeness, age, sex, bmi, education, income, physical activity, smoking, menopause, other drug use, dietary factors, other, hypertension, diabetes, vitamin deficiency, other diagnosed disease, APOE, family history, hypercholesterolemia
Chang	2002	Prostate cancer	Male	Non-drinkers	Light drinkers	0.26	0.16	representativeness, age, sex, bmi, education, income, physical activity, smoking, menopause, other drug use, dietary factors, other, hypertension, diabetes, vitamin deficiency, other diagnosed disease, APOE, family history, hypercholesterolemia
Chang	2002	Prostate cancer	Male	Non-drinkers	Moderate drinkers	0.26	0.14	representativeness, age, sex, bmi, education, income, physical activity, smoking, menopause, other drug use, dietary factors, other, hypertension, diabetes, vitamin deficiency, other diagnosed disease, APOE, family history, hypercholesterolemia

Table S10. Summary of Data Inputs

Author	Year	Health outcome	Sex	Reference	Exposed group	Natural Log of Effect Size	Standard Error of Natural Log of Effect Size	Covariates Adjusted for in Source
Chao	2007	Prostate cancer	Male	Non-drinkers	Light drinkers	-0.01	0.09	representativeness, age, sex, bmi, education, income, physical activity, smoking, menopause, other drug use, dietary factors, other, hypertension, diabetes, vitamin deficiency, other diagnosed disease, APOE, family history, hypercholesterolemia
Chao	2007	Prostate cancer	Male	Non-drinkers	Light drinkers	0.00	0.08	representativeness, age, sex, bmi, education, income, physical activity, smoking, menopause, other drug use, dietary factors, other, hypertension, diabetes, vitamin deficiency, other diagnosed disease, APOE, family history, hypercholesterolemia
Chao	2007	Prostate cancer	Male	Non-drinkers	Light drinkers	-0.06	0.09	representativeness, age, sex, bmi, education, income, physical activity, smoking, menopause, other drug use, dietary factors, other, hypertension, diabetes, vitamin deficiency, other diagnosed disease, APOE, family history, hypercholesterolemia
Chao	2007	Prostate cancer	Male	Non-drinkers	Light drinkers	-0.05	0.09	representativeness, age, sex, bmi, education, income, physical activity, smoking, menopause, other drug use, dietary factors, other, hypertension, diabetes, vitamin deficiency, other diagnosed disease, APOE, family history, hypercholesterolemia
Chao	2007	Prostate cancer	Male	Non-drinkers	Light drinkers	0.05	0.12	representativeness, age, sex, bmi, education, income, physical activity, smoking, menopause, other drug use, dietary factors, other, hypertension, diabetes, vitamin deficiency, other diagnosed disease, APOE, family history, hypercholesterolemia
Chao	2007	Prostate cancer	Male	Non-drinkers	Light drinkers	-0.04	0.08	representativeness, age, sex, bmi, education, income, physical activity, smoking, menopause, other drug use, dietary factors, other, hypertension, diabetes, vitamin deficiency, other diagnosed disease, APOE, family history, hypercholesterolemia
Chao	2007	Prostate cancer	Male	Non-drinkers	Light drinkers	-0.04	0.11	representativeness, age, sex, bmi, education, income, physical activity, smoking, menopause, other drug use, dietary factors, other, hypertension, diabetes, vitamin deficiency, other diagnosed disease, APOE, family history, hypercholesterolemia
Chao	2007	Prostate cancer	Male	Non-drinkers	Light drinkers	0.10	0.16	representativeness, age, sex, bmi, education, income, physical activity, smoking, menopause, other drug use, dietary factors, other, hypertension, diabetes, vitamin deficiency, other diagnosed disease, APOE, family history, hypercholesterolemia
Chao	2007	Prostate cancer	Male	Non-drinkers	Light drinkers	0.10	0.09	representativeness, age, sex, bmi, education, income, physical activity, smoking, menopause, other drug use, dietary factors, other, hypertension, diabetes, vitamin deficiency, other diagnosed disease, APOE, family history, hypercholesterolemia
Hayes	1989	Prostate cancer	Male	Non-drinkers	Heavy drinkers	0.26	0.18	representativeness, age, sex, bmi, education, income, physical activity, smoking, menopause, other drug use, dietary factors, other, hypertension, diabetes, vitamin deficiency, other diagnosed disease, APOE, family history, hypercholesterolemia
Hayes	1989	Prostate cancer	Male	Non-drinkers	Light drinkers	0.10	0.16	representativeness, age, sex, bmi, education, income, physical activity, smoking, menopause, other drug use, dietary factors, other, hypertension, diabetes, vitamin deficiency, other diagnosed disease, APOE, family history, hypercholesterolemia
Hayes	1989	Prostate cancer	Male	Non-drinkers	Moderate drinkers	0.10	0.16	representativeness, age, sex, bmi, education, income, physical activity, smoking, menopause, other drug use, dietary factors, other, hypertension, diabetes, vitamin deficiency, other diagnosed disease, APOE, family history, hypercholesterolemia

Table S10. Summary of Data Inputs

Author	Year	Health outcome	Sex	Reference	Exposed group	Natural Log of Effect Size	Standard Error of Natural Log of Effect Size	Covariates Adjusted for in Source
Hiatt	1985	Prostate cancer	Male	Non-drinkers	Light drinkers	0.26	0.26	representativeness, age, sex, bmi, education, income, physical activity, smoking, menopause, other drug use, dietary factors, other, hypertension, diabetes, vitamin deficiency, other diagnosed disease, APOE, family history, hypercholesterolemia
Hiatt	1985	Prostate cancer	Male	Non-drinkers	Heavy drinkers	0.10	0.31	representativeness, age, sex, bmi, education, income, physical activity, smoking, menopause, other drug use, dietary factors, other, hypertension, diabetes, vitamin deficiency, other diagnosed disease, APOE, family history, hypercholesterolemia
Hiatt	1985	Prostate cancer	Male	Non-drinkers	Heavy drinkers	0.00	0.50	representativeness, age, sex, bmi, education, income, physical activity, smoking, menopause, other drug use, dietary factors, other, hypertension, diabetes, vitamin deficiency, other diagnosed disease, APOE, family history, hypercholesterolemia
Lumey	1991	Prostate cancer	Male	Non-drinkers	Moderate drinkers	0.10	0.16	representativeness, age, sex, bmi, education, income, physical activity, smoking, menopause, other drug use, dietary factors, other, hypertension, diabetes, vitamin deficiency, other diagnosed disease, APOE, family history, hypercholesterolemia
Lumey	1991	Prostate cancer	Male	Non-drinkers	Heavy drinkers	0.26	0.23	representativeness, age, sex, bmi, education, income, physical activity, smoking, menopause, other drug use, dietary factors, other, hypertension, diabetes, vitamin deficiency, other diagnosed disease, APOE, family history, hypercholesterolemia
Olmedo-Requena	2019	Prostate cancer	Male	Non-drinkers	Light drinkers	-0.01	0.20	representativeness, age, sex, bmi, education, income, physical activity, smoking, menopause, other drug use, dietary factors, other, hypertension, diabetes, vitamin deficiency, other diagnosed disease, APOE, family history, hypercholesterolemia
Rohrmann	2000	Prostate cancer	Male	Light drinkers	Light drinkers	-0.01	0.06	representativeness, age, sex, bmi, education, income, physical activity, smoking, menopause, other drug use, dietary factors, other, hypertension, diabetes, vitamin deficiency, other diagnosed disease, APOE, family history, hypercholesterolemia
Rohrmann	2000	Prostate cancer	Male	Light drinkers	Heavy drinkers	0.03	0.07	representativeness, age, sex, bmi, education, income, physical activity, smoking, menopause, other drug use, dietary factors, other, hypertension, diabetes, vitamin deficiency, other diagnosed disease, APOE, family history, hypercholesterolemia
Rohrmann	2000	Prostate cancer	Male	Light drinkers	Light drinkers	-0.05	0.08	representativeness, age, sex, bmi, education, income, physical activity, smoking, menopause, other drug use, dietary factors, other, hypertension, diabetes, vitamin deficiency, other diagnosed disease, APOE, family history, hypercholesterolemia
Rohrmann	2000	Prostate cancer	Male	Light drinkers	Moderate drinkers	-0.05	0.06	representativeness, age, sex, bmi, education, income, physical activity, smoking, menopause, other drug use, dietary factors, other, hypertension, diabetes, vitamin deficiency, other diagnosed disease, APOE, family history, hypercholesterolemia
Sarich	2013	Prostate cancer	Both	Light drinkers	Light drinkers	0.02	0.05	representativeness, age, sex, bmi, education, income, physical activity, smoking, menopause, other drug use, dietary factors, other, hypertension, diabetes, vitamin deficiency, other diagnosed disease, APOE, family history, hypercholesterolemia
Sarich	2013	Prostate cancer	Both	Light drinkers	Light drinkers	0.08	0.05	representativeness, age, sex, bmi, education, income, physical activity, smoking, menopause, other drug use, dietary factors, other, hypertension, diabetes, vitamin deficiency, other diagnosed disease, APOE, family history, hypercholesterolemia

Table S10. Summary of Data Inputs

Author	Year	Health outcome	Sex	Reference	Exposed group	Natural Log of Effect Size	Standard Error of Natural Log of Effect Size	Covariates Adjusted for in Source
Schoonen	1996	Prostate cancer	Male	Light drinkers	Light drinkers	-0.06	0.17	representativeness, age, sex, bmi, education, income, physical activity, smoking, menopause, other drug use, dietary factors, other, hypertension, diabetes, vitamin deficiency, other diagnosed disease, APOE, family history, hypercholesterolemia
Schoonen	1996	Prostate cancer	Male	Light drinkers	Light drinkers	0.06	0.19	representativeness, age, sex, bmi, education, income, physical activity, smoking, menopause, other drug use, dietary factors, other, hypertension, diabetes, vitamin deficiency, other diagnosed disease, APOE, family history, hypercholesterolemia
Schoonen	1996	Prostate cancer	Male	Light drinkers	Light drinkers	0.13	0.19	representativeness, age, sex, bmi, education, income, physical activity, smoking, menopause, other drug use, dietary factors, other, hypertension, diabetes, vitamin deficiency, other diagnosed disease, APOE, family history, hypercholesterolemia
Schuurman	1992	Prostate cancer	Male	Non-drinkers	Moderate drinkers	0.83	0.43	representativeness, age, sex, bmi, education, income, physical activity, smoking, menopause, other drug use, dietary factors, other, hypertension, diabetes, vitamin deficiency, other diagnosed disease, APOE, family history, hypercholesterolemia
Schuurman	1992	Prostate cancer	Male	Non-drinkers	Moderate drinkers	0.00	0.35	representativeness, age, sex, bmi, education, income, physical activity, smoking, menopause, other drug use, dietary factors, other, hypertension, diabetes, vitamin deficiency, other diagnosed disease, APOE, family history, hypercholesterolemia
Schuurman	1992	Prostate cancer	Male	Non-drinkers	Light drinkers	0.00	0.18	representativeness, age, sex, bmi, education, income, physical activity, smoking, menopause, other drug use, dietary factors, other, hypertension, diabetes, vitamin deficiency, other diagnosed disease, APOE, family history, hypercholesterolemia
Schuurman	1992	Prostate cancer	Male	Non-drinkers	Light drinkers	0.10	0.16	representativeness, age, sex, bmi, education, income, physical activity, smoking, menopause, other drug use, dietary factors, other, hypertension, diabetes, vitamin deficiency, other diagnosed disease, APOE, family history, hypercholesterolemia
Schuurman	1992	Prostate cancer	Male	Non-drinkers	Moderate drinkers	0.10	0.14	representativeness, age, sex, bmi, education, income, physical activity, smoking, menopause, other drug use, dietary factors, other, hypertension, diabetes, vitamin deficiency, other diagnosed disease, APOE, family history, hypercholesterolemia
Schuurman	1992	Prostate cancer	Male	Non-drinkers	Moderate drinkers	0.10	0.18	representativeness, age, sex, bmi, education, income, physical activity, smoking, menopause, other drug use, dietary factors, other, hypertension, diabetes, vitamin deficiency, other diagnosed disease, APOE, family history, hypercholesterolemia
Schuurman	1992	Prostate cancer	Male	Non-drinkers	Light drinkers	0.10	0.21	representativeness, age, sex, bmi, education, income, physical activity, smoking, menopause, other drug use, dietary factors, other, hypertension, diabetes, vitamin deficiency, other diagnosed disease, APOE, family history, hypercholesterolemia
Schuurman	1992	Prostate cancer	Male	Non-drinkers	Light drinkers	0.10	0.14	representativeness, age, sex, bmi, education, income, physical activity, smoking, menopause, other drug use, dietary factors, other, hypertension, diabetes, vitamin deficiency, other diagnosed disease, APOE, family history, hypercholesterolemia
Schuurman	1992	Prostate cancer	Male	Non-drinkers	Moderate drinkers	0.10	0.24	representativeness, age, sex, bmi, education, income, physical activity, smoking, menopause, other drug use, dietary factors, other, hypertension, diabetes, vitamin deficiency, other diagnosed disease, APOE, family history, hypercholesterolemia

Table S10. Summary of Data Inputs

Author	Year	Health outcome	Sex	Reference	Exposed group	Natural Log of Effect Size	Standard Error of Natural Log of Effect Size	Covariates Adjusted for in Source
Schuurman	1992	Prostate cancer	Male	Non-drinkers	Moderate drinkers	0.10	0.21	representativeness, age, sex, bmi, education, income, physical activity, smoking, menopause, other drug use, dietary factors, other, hypertension, diabetes, vitamin deficiency, other diagnosed disease, APOE, family history, hypercholesterolemia
Schuurman	1992	Prostate cancer	Male	Non-drinkers	Moderate drinkers	0.10	0.31	representativeness, age, sex, bmi, education, income, physical activity, smoking, menopause, other drug use, dietary factors, other, hypertension, diabetes, vitamin deficiency, other diagnosed disease, APOE, family history, hypercholesterolemia
Sutcliffe	2002	Prostate cancer	Male	Non-drinkers	Light drinkers	0.02	0.09	representativeness, age, sex, bmi, education, income, physical activity, smoking, menopause, other drug use, dietary factors, other, hypertension, diabetes, vitamin deficiency, other diagnosed disease, APOE, family history, hypercholesterolemia
Sutcliffe	2002	Prostate cancer	Male	Non-drinkers	N/A	0.13	0.07	representativeness, age, sex, bmi, education, income, physical activity, smoking, menopause, other drug use, dietary factors, other, hypertension, diabetes, vitamin deficiency, other diagnosed disease, APOE, family history, hypercholesterolemia
Velicer	2002	Prostate cancer	Male	Light drinkers	Moderate drinkers	0.08	0.11	representativeness, age, sex, bmi, education, income, physical activity, smoking, menopause, other drug use, dietary factors, other, hypertension, diabetes, vitamin deficiency, other diagnosed disease, APOE, family history, hypercholesterolemia
Wang	2004	Prostate cancer	Both	Non-drinkers	Light drinkers	0.01	0.31	age, sex, bmi, education, income, physical activity
Wang	2004	Prostate cancer	Both	Non-drinkers	Light drinkers	-0.12	0.32	age, sex, bmi, education, income, physical activity
Watters	2003	Prostate cancer	Male	Non-drinkers	Light drinkers	0.06	0.02	representativeness, age, sex, bmi, education, income, physical activity, smoking, menopause, other drug use, dietary factors, other, hypertension, diabetes, vitamin deficiency, other diagnosed disease, APOE, family history, hypercholesterolemia
Watters	2003	Prostate cancer	Male	Non-drinkers	Moderate drinkers	0.07	0.03	representativeness, age, sex, bmi, education, income, physical activity, smoking, menopause, other drug use, dietary factors, other, hypertension, diabetes, vitamin deficiency, other diagnosed disease, APOE, family history, hypercholesterolemia
Lee	2017	Stomach cancer	Female	Non-drinkers	Light drinkers	0.00	0.19	representativeness, age, sex, bmi, education, income, physical activity, smoking, menopause, other drug use, dietary factors, other, hypertension, diabetes, vitamin deficiency, other diagnosed disease, APOE, family history, hypercholesterolemia
Nakaya	1997	Stomach cancer	Both	Non-drinkers	Light drinkers	0.00	0.23	age, sex, bmi, education, income, physical activity, smoking, dietary factors
Steevens	2002	Stomach cancer	Both	Non-drinkers	Moderate drinkers	0.00	0.20	age, sex, bmi, education, income, physical activity, smoking, dietary factors, other
Sung	2002	Stomach cancer	Male	Non-drinkers	Light drinkers	0.00	0.05	representativeness, age, sex, bmi, education, income, physical activity, smoking, menopause, other drug use, dietary factors, other, hypertension, diabetes, vitamin deficiency, other diagnosed disease, APOE, family history, hypercholesterolemia
Soh	2014	Tuberculosis	Both	Non-drinkers	Light drinkers	0.06	0.19	representativeness, exposure_quality, outcome_quality, confounder_quality, reverse_causation, selection_bias, sex

Table S10. Summary of Data Inputs

Author	Year	Health outcome	Sex	Reference	Exposed group	Natural Log of Effect Size	Standard Error of Natural Log of Effect Size	Covariates Adjusted for in Source
Soh	2014	Tuberculosis	Both	Non-drinkers	Moderate drinkers	0.37	0.14	representativeness, exposure_quality, outcome_quality, confounder_quality, reverse_causation, selection_bias, sex
Soh	2014	Tuberculosis	Both	Non-drinkers	Light drinkers	0.06	0.19	representativeness, age, sex, bmi, education, income, physical activity, smoking, menopause, other drug use, dietary factors, other, hypertension, diabetes, vitamin deficiency, other diagnosed disease, APOE, family history, hypercholesterolemia
Soh	2014	Tuberculosis	Both	Non-drinkers	Moderate drinkers	0.37	0.14	representativeness, age, sex, bmi, education, income, physical activity, smoking, menopause, other drug use, dietary factors, other, hypertension, diabetes, vitamin deficiency, other diagnosed disease, APOE, family history, hypercholesterolemia

N/A, not available

C. Risk Of Bias By Study

Table S11. Bias covariates

Author	Year	Study design	Health outcome	incidence	age	sex	bmi	in-come	smoking	hyper-tension	APOE	other disease	diet	education	high loss to follow-up	prevalent disease population
Adami	1985	case-control	Breast cancer	1	1	0	0	0	1	0	0	0	0	0	0	0
Albertsen	1994	prospective cohort	Prostate cancer	1	1	0	0	0	0	0	0	0	0	0	0	0
Almirall	2000	case-control	Lower respiratory infections	1	1	0	0	0	0	0	0	1	1	0	0	0
Almirall	2000	case-control	Lower respiratory infections	1	0	0	0	0	0	0	0	0	0	0	0	0
Almirall	2000	prospective cohort	Lower respiratory infections	1	1	0	0	0	0	0	0	1	1	0	0	0
Almirall	2000	prospective cohort	Lower respiratory infections	1	0	0	0	0	0	0	0	0	0	0	0	0
Almodova	2012	case-control	Esophageal cancer	1	0	0	0	0	0	0	0	0	0	0	0	0
Andersson	1991	case-control	Prostate cancer	1	1	0	0	0	0	0	0	0	0	0	0	0
Ariansen	2019	prospective cohort	Atrial fibrillation and flutter	1	1	0	0	0	0	0	0	0	0	0	0	0
Asim	2007	case-control	Liver cancer	1	1	0	0	0	0	0	0	0	0	0	0	0
Askgaard	2013	prospective cohort	Haemorrhagic stroke	0	1	1	0	0	0	0	0	0	0	0	0	0
Baglietto	1994	prospective cohort	Prostate cancer	1	1	0	0	0	0	0	0	0	0	0	0	0
Baik	2012	prospective cohort	Diabetes mellitus type 2	1	1	0	0	0	0	0	0	0	0	0	0	0

Table S11. Bias covariates

Author	Year	Study design	Health outcome	incidence	age	sex	bmi	in-come	smoking	hyper-tension	APOE	other disease	diet	education	high loss to follow-up	prevalent disease population
Baik	1992	prospective cohort	Lower respiratory infections	0	1	0	0	0	0	0	0	0	1	0	0	0
Bassett	1994	prospective cohort	Breast cancer	1	1	0	0	0	1	0	0	0	0	0	0	0
Bazal	2017	randomized control trial	Atrial fibrillation and flutter	1	1	0	0	0	0	0	0	0	0	0	0	0
Becker	1998	prospective cohort	Cirrhosis and other chronic liver diseases	0	0	1	0	0	0	0	0	0	0	0	0	0
Becker	1992	prospective cohort	Cirrhosis and other chronic liver diseases	1	1	1	0	0	0	0	0	0	0	0	0	0
Benzon	2006	nested case-control	Breast cancer	1	1	0	0	0	0	0	0	0	0	0	0	0
Berstad	2003	case-control	Breast cancer	1	1	0	0	0	1	0	0	0	0	0	0	0
Beulens	2002	prospective cohort	Diabetes mellitus type 2	1	1	0	0	0	0	0	0	0	0	0	0	0
Beulens	2000	prospective cohort	Diabetes mellitus type 2	1	1	0	0	0	0	0	0	0	0	0	0	0
Biddinger	2016	prospective cohort	Atrial fibrillation and flutter	0	1	0	0	0	0	0	0	0	0	0	0	0
Biddinger	2016	prospective cohort	Ischemic heart disease	1	1	0	1	0	0	0	0	0	0	0	0	0
Bos	2006	prospective cohort	Ischemic heart disease	0	1	0	1	0	0	0	0	0	0	0	0	0
Bowlin	1986	case-control	Breast cancer	1	1	0	0	0	1	0	0	0	0	0	0	0
Breslow	1992	prospective cohort	Prostate cancer	1	0	0	0	0	0	0	0	0	0	0	0	0

Table S11. Bias covariates

Author	Year	Study design	Health outcome	incidence	age	sex	bmi	in-come	smoking	hyper-tension	APOE	other disease	diet	education	high loss to follow-up	prevalent disease population
Britton	2007	prospective cohort	Ischemic heart disease	1	1	0	1	0	0	0	0	0	0	0	0	0
Bryson	2001	prospective cohort	Ischemic heart disease	1	1	0	1	0	0	0	0	0	0	0	0	0
Bui	2018	prospective cohort	Liver cancer	1	1	0	0	0	0	0	0	0	0	0	0	0
Bui	2018	prospective cohort	Pancreatic cancer	1	1	0	0	0	0	0	0	0	0	0	0	0
Camargo	1993	prospective cohort	Ischemic heart disease	1	0	0	0	0	0	0	0	0	0	0	0	0
Cha	2015	prospective cohort	Atrial fibrillation and flutter	1	1	0	0	0	0	0	0	0	0	0	0	0
Chang	2002	case-control	Prostate cancer	1	1	0	0	0	0	0	0	0	0	0	0	0
Chao	2007	prospective cohort	Prostate cancer	1	1	0	0	0	0	0	0	0	0	0	0	0
Chen	2017	case-control	Lip and oral cavity cancer	0	1	0	0	0	0	0	0	0	0	0	0	0
Chen	2008	prospective cohort	Breast cancer	1	1	0	0	0	1	0	0	0	0	0	0	0
Chen	2006	case-control	Esophageal cancer	1	0	0	0	0	0	0	0	0	0	0	0	0
Cho	2017	prospective cohort	Ischemic heart disease	0	1	0	1	0	0	0	0	0	0	0	0	0
Cho	2017	prospective cohort	Ischemic stroke	0	1	0	0	1	0	0	0	0	0	0	0	0
Cho	2013	prospective cohort	Liver cancer	1	1	0	0	0	0	0	0	0	0	0	0	0
Cho	2013	prospective cohort	Larynx cancer	0	1	0	0	0	0	0	0	0	0	0	0	0

Table S11. Bias covariates

Author	Year	Study design	Health outcome	incidence	age	sex	bmi	in-come	smoking	hyper-tension	APOE	other disease	diet	education	high loss to follow-up	prevalent disease population
Choi	2016	prospective cohort	Esophageal cancer	1	1	0	0	0	0	0	0	0	0	0	0	0
Choi	2008	prospective cohort	Diabetes mellitus type 2	1	1	0	0	0	0	0	0	0	0	0	0	0
Choi	1990	case-control	Colon and rectum cancer	1	1	0	0	0	0	0	0	0	0	1	0	0
Chyou	1995	prospective cohort	Colon and rectum cancer	1	1	0	0	0	0	0	0	0	0	0	0	0
Cococcia	2017	retrospective cohort	Liver cancer	0	1	0	0	0	0	0	0	0	0	0	0	0
Conway	2014	case-control	Other pharynx cancer	0	0	0	0	0	0	0	0	0	0	0	0	0
Crockett	2006	case-control	Colon and rectum cancer	1	1	0	0	0	0	0	0	0	0	1	0	0
Csengeri	2010	prospective cohort	Atrial fibrillation and flutter	1	1	0	0	0	0	0	0	0	0	0	0	0
Cullen	1989	prospective cohort	Ischemic heart disease	0	1	0	0	0	0	0	0	0	0	0	0	0
Dam	2012	prospective cohort	Ischemic heart disease	1	0	0	0	0	0	0	0	0	0	0	0	0
De Stefani	2004	case-control	Esophageal cancer	1	0	0	0	0	0	0	0	0	0	0	0	0
Di Benedetto	2017	prospective cohort	Atrial fibrillation and flutter	1	1	0	0	0	0	0	0	0	0	0	0	0
Di Castelnuovo	2015	prospective cohort	Atrial fibrillation and flutter	0	1	0	0	0	0	0	0	0	0	0	0	0

Table S11. Bias covariates

Author	Year	Study design	Health outcome	incidence	age	sex	bmi	in-come	smoking	hyper-tension	APOE	other disease	diet	education	high loss to follow-up	prevalent disease population
Dixit	2013	prospective cohort	Atrial fibrillation and flutter	0	1	0	0	0	0	0	0	0	0	0	0	0
Djousse	1998	case-control	Atrial fibrillation and flutter	1	1	0	0	0	0	0	0	0	0	0	0	0
Donahue	1977	prospective cohort	Ischemic stroke	1	0	0	0	0	0	0	0	0	0	0	0	0
Donat-Vargas	2011	case-control	Breast cancer	1	1	0	0	0	1	0	0	0	0	0	0	0
Dumeaux	2001	prospective cohort	Breast cancer	1	1	0	0	0	0	0	0	0	0	0	0	0
Ericson	2003	prospective cohort	Breast cancer	1	1	0	0	0	0	0	0	0	0	0	0	0
Ewertz	1984	case-control	Breast cancer	1	1	0	0	0	0	0	0	0	0	0	0	0
Ferraroni	1985	case-control	Breast cancer	1	1	0	0	0	0	0	0	0	0	0	0	0
Feter	2022	Prospective Cohort	Alzheimer's disease and other dementias	0	1	1	0	0	0	0	0	0	1	1	0	0
Filippini	2019	case-control	Alzheimer's disease and other dementias	1	1	0	0	0	0	0	0	0	0	0	0	0
Flanders	1979	case-control	Larynx cancer	1	0	0	0	0	0	0	0	0	0	0	0	0
Flood	1998	case-control	Colon and rectum cancer	1	0	0	0	0	0	0	0	0	0	0	0	0
Floud	2001	prospective cohort	Breast cancer	1	1	0	0	0	1	0	0	0	0	0	0	0
Floud	2001	prospective cohort	Colon and rectum cancer	1	1	0	0	0	0	0	0	0	0	1	0	0

Table S11. Bias covariates

Author	Year	Study design	Health outcome	incidence	age	sex	bmi	in-come	smoking	hyper-tension	APOE	other disease	diet	education	high loss to follow-up	prevalent disease population
Floud	2001	prospective cohort	Liver cancer	1	1	0	0	0	0	0	0	0	0	0	0	0
Floud	2001	prospective cohort	Pancreatic cancer	1	1	1	0	0	0	0	0	0	0	0	0	0
Floud	2001	prospective cohort	Pancreatic cancer	1	0	0	0	0	0	0	0	0	0	0	0	0
Frederiksen	2017	prospective cohort	Atrial fibrillation and flutter	1	1	0	0	0	0	0	0	0	0	0	0	0
Freedman	2000	prospective cohort	Esophageal cancer	1	1	0	0	0	0	0	0	0	0	0	0	0
Freisling	2007	prospective cohort	Diabetes mellitus type 2	1	1	0	0	0	0	0	0	0	0	0	0	0
Friedman	1962	prospective cohort	Ischemic heart disease	1	0	0	0	0	0	0	0	0	0	0	0	0
Friedman	1962	prospective cohort	Ischemic heart disease	0	0	0	0	0	0	0	0	0	0	0	0	0
Frost	1997	prospective cohort	Atrial fibrillation and flutter	1	1	0	0	0	0	0	0	0	0	0	0	0
Fuchs	1992	prospective cohort	Cirrhosis and other chronic liver diseases	0	1	0	0	0	0	0	0	0	0	0	0	0
Fuchs	1992	prospective cohort	Ischemic heart disease	0	1	0	1	0	0	0	0	0	0	0	0	0
Gao	1993	case-control	Esophageal cancer	1	1	0	0	0	0	0	0	0	0	0	0	0
Gapstur	1989	prospective cohort	Breast cancer	1	1	0	0	0	0	0	0	0	0	0	0	0
Garg	1987	prospective cohort	Ischemic heart disease	0	1	0	1	0	0	0	0	0	0	0	0	0

Table S11. Bias covariates

Author	Year	Study design	Health outcome	incidence	age	sex	bmi	in-come	smoking	hyper-tension	APOE	other disease	diet	education	high loss to follow-up	prevalent disease population
Garidou	1991	case-control	Esophageal cancer	1	0	0	0	0	0	0	0	0	0	0	0	0
Gémes,	2015	prospective cohort	Atrial fibrillation and flutter	1	1	0	0	0	0	0	0	0	0	0	0	0
Goldberg	1980	prospective cohort	Haemorrhagic stroke	1	0	0	0	0	0	0	0	0	0	0	0	0
Goldberg	1980	prospective cohort	Ischemic stroke	1	0	0	0	0	0	0	0	0	0	0	0	0
Goldbohm	1989	case-control	Colon and rectum cancer	1	1	0	0	0	0	0	0	0	0	1	0	0
Goodman	1989	prospective cohort	Liver cancer	0	1	0	0	0	0	0	0	0	0	0	0	0
Gordon	1972	prospective cohort	Haemorrhagic stroke	0	0	0	0	0	0	0	0	0	0	0	0	0
Gorecka	2014	prospective cohort	Atrial fibrillation and flutter	1	1	0	0	0	0	0	0	0	0	0	0	0
Halford	2019	prospective cohort	Atrial fibrillation and flutter	1	1	0	0	0	0	0	0	0	0	0	0	0
Hamada	2015	retrospective cohort	Atrial fibrillation and flutter	1	1	0	0	0	0	0	0	0	0	0	0	0
Han	2019	prospective cohort	Esophageal cancer	0	1	0	0	0	0	0	0	0	0	0	0	0
Han	2017	prospective cohort	Ischemic heart disease	1	1	0	0	0	0	0	0	0	0	0	0	0
Han	2012	retrospective cohort	Atrial fibrillation and flutter	1	1	0	0	0	0	0	0	0	0	0	0	0

Table S11. Bias covariates

Author	Year	Study design	Health outcome	incidence	age	sex	bmi	in-come	smoking	hyper-tension	APOE	other disease	diet	education	high loss to follow-up	prevalent disease population
Handing	2010	prospective cohort	Alzheimer's disease and other dementias	1	1	0	0	0	0	0	0	0	0	0	1	0
Harris	2008	prospective cohort	Breast cancer	0	1	0	0	0	1	0	0	0	0	0	0	0
Hart	2003	prospective cohort	Ischemic heart disease	0	1	0	1	0	0	0	0	0	0	0	0	0
Hashibe	2002	case-control	Esophageal cancer	1	1	0	0	0	0	0	0	0	0	0	0	0
Hatime	2020	case-control	Colon and rectum cancer	1	0	0	0	0	0	0	0	0	0	0	0	0
Hayes	1989	case-control	Prostate cancer	1	1	0	0	0	0	0	0	0	0	0	0	0
He	2013	prospective cohort	Diabetes mellitus type 2	1	1	0	0	0	0	0	0	0	0	0	0	0
Heffernan	2015	prospective cohort	Alzheimer's disease and other dementias	1	1	0	0	0	0	0	0	0	0	0	0	0
Hein	1986	prospective cohort	Ischemic heart disease	1	1	0	0	0	0	0	0	0	0	0	0	0
Herrero	1999	case-control	Lip and oral cavity cancer	0	1	0	0	0	0	0	0	0	0	0	0	0
Hiatt	1985	prospective cohort	Prostate cancer	1	0	0	0	0	0	0	0	0	0	0	0	0
Higashiyama	2007	prospective cohort	Ischemic heart disease	1	1	0	1	0	0	0	0	0	0	0	0	0
Hinkle	2017	prospective cohort	Diabetes mellitus type 2	1	1	0	0	0	0	0	0	0	0	0	0	0
Hirose	2000	case-control	Breast cancer	1	0	0	0	0	0	0	0	0	0	0	0	0

Table S11. Bias covariates

Author	Year	Study design	Health outcome	incidence	age	sex	bmi	in-come	smoking	hyper-tension	APOE	other disease	diet	education	high loss to follow-up	prevalent disease population
Hoyer	1989	prospective cohort	Breast cancer	1	1	0	0	0	1	0	0	0	0	0	0	0
Hu	2015	prospective cohort	Ischemic heart disease	1	1	0	0	0	0	0	0	0	0	0	0	0
Huang	2016	case-control	Lip and oral cavity cancer	0	1	0	0	0	0	0	0	0	0	0	0	0
Hur	2021	prospective cohort	Colon and rectum cancer	1	1	0	0	0	0	0	0	0	0	1	0	0
Ishiguro	2004	prospective cohort	Esophageal cancer	1	1	0	0	0	0	0	0	0	0	0	0	0
Iso	2002	prospective cohort	Haemorrhagic stroke	0	1	0	0	0	0	0	0	0	0	0	0	0
Iso	2002	prospective cohort	Ischemic stroke	0	1	0	0	0	0	0	0	0	0	0	0	0
Jain	1993	prospective cohort	Breast cancer	0	1	0	0	0	1	0	0	0	0	0	0	0
Jang	2008	case-control	Liver cancer	1	0	0	0	0	0	0	0	0	0	0	0	0
Järvenpää	2005	prospective cohort	Alzheimer's disease and other dementias	1	1	0	0	0	0	0	0	0	0	0	1	0
Jayaprakash	1998	case-control	Esophageal cancer	1	0	0	0	0	0	0	0	0	0	0	0	0
Jayasekara	2015	prospective cohort	Pancreatic cancer	0	1	1	0	0	0	0	0	0	0	0	0	0
Jee	2002	prospective cohort	Liver cancer	0	1	0	0	0	0	0	0	0	0	0	0	0
Jeon	2023	Prospective Cohort	Alzheimer's disease and other dementias	0	1	1	1	0	1	0	0	0	1	1	0	0

Table S11. Bias covariates

Author	Year	Study design	Health outcome	incidence	age	sex	bmi	in-come	smoking	hyper-tension	APOE	other disease	diet	education	high loss to follow-up	prevalent disease population
Jiang	2008	case-control	Ischemic heart disease	1	1	0	1	0	0	0	0	0	0	0	0	0
Johansson	2014	prospective cohort	Atrial fibrillation and flutter	1	1	0	0	0	0	0	0	0	0	0	0	0
Joosten	2006	prospective cohort	Diabetes mellitus type 2	1	1	0	0	0	0	0	0	0	0	0	0	0
Joshi	2004	prospective cohort	Liver cancer	1	1	0	0	0	0	0	0	0	0	0	0	0
Jung	2024	Prospective Cohort	Alzheimer's disease and other dementias	0	1	1	1	0	1	1	0	1	0	0	0	0
Jung	2004	prospective cohort	Liver cancer	0	1	0	0	0	0	0	0	0	0	0	0	0
Kabat	2000	prospective cohort	Colon and rectum cancer	0	1	0	0	0	0	0	0	0	0	1	0	0
Kabat	1990	case-control	Esophageal cancer	1	1	0	0	0	0	0	0	0	0	0	0	0
Kao	1998	prospective cohort	Diabetes mellitus type 2	1	1	0	0	0	0	0	0	0	0	0	0	0
Karavasiloglou	2010	prospective cohort	Breast cancer	1	0	0	0	0	1	0	0	0	0	0	0	0
Kawakami	2020	prospective cohort	Alzheimer's disease and other dementias	1	1	0	0	0	0	0	0	0	0	0	0	0
Kawakita	2009	case-control	Larynx cancer	1	0	0	0	0	0	0	0	0	0	0	0	0
Kim	2017	retrospective cohort	Atrial fibrillation and flutter	1	1	0	0	0	0	0	0	0	0	0	0	0

Table S11. Bias covariates

Author	Year	Study design	Health outcome	incidence	age	sex	bmi	in-come	smoking	hyper-tension	APOE	other disease	diet	education	high loss to follow-up	prevalent disease population
Kim	2016	prospective cohort	Ischemic heart disease	1	1	0	1	0	0	0	0	0	0	0	0	0
Kim	2016	prospective cohort	Ischemic stroke	0	1	0	0	1	0	0	0	0	0	0	0	0
Kimm	2006	prospective cohort	Esophageal cancer	1	1	0	0	0	0	0	0	0	0	0	0	0
Kimm	2006	prospective cohort	Esophageal cancer	0	1	0	0	0	0	0	0	0	0	0	0	0
Kinney	1996	case-control	Breast cancer	1	1	0	0	0	1	0	0	0	0	0	0	0
Kivela	1984	prospective cohort	Ischemic heart disease	0	0	0	0	0	0	0	0	0	0	0	0	0
Kiyohara	1987	prospective cohort	Haemorrhagic stroke	0	1	1	0	0	0	0	0	0	0	0	0	0
Klatsky	1996	prospective cohort	Haemorrhagic stroke	1	0	1	0	0	0	0	0	0	0	0	0	0
Klatsky	1991	prospective cohort	Ischemic heart disease	0	1	0	1	0	0	0	0	0	0	0	0	0
Koch	2019	Prospective Cohort	Alzheimer's disease and other dementias	0	1	1	1	0	1	1	0	1	0	1	0	0
Koh	2007	case-control	Liver cancer	1	1	0	0	0	0	0	0	0	0	0	0	0
Kono	1983	prospective cohort	Colon and rectum cancer	1	1	0	0	0	0	0	0	0	0	0	0	0
Kono	1983	prospective cohort	Colon and rectum cancer	0	1	0	0	0	0	0	0	0	0	0	0	0
Kono	1983	prospective cohort	Pancreatic cancer	0	1	1	0	0	0	0	0	0	0	0	0	0

Table S11. Bias covariates

Author	Year	Study design	Health outcome	incidence	age	sex	bmi	in-come	smoking	hyper-tension	APOE	other disease	diet	education	high loss to follow-up	prevalent disease population
Koyanagi	2013	case-control	Pancreatic cancer	1	1	1	0	0	0	0	0	0	0	0	0	0
Kristiansen	2003	prospective cohort	Pancreatitis	0	1	0	0	0	0	0	0	0	0	0	0	0
Kume	2010	case-control	Pancreatitis	0	1	0	0	0	0	0	0	0	0	0	0	0
Kwan	2013	prospective cohort	Breast cancer	0	1	0	0	0	1	0	0	0	0	0	0	0
La	1988	case-control	Breast cancer	1	1	0	0	0	1	0	0	0	0	0	0	0
Laaksonen	2018	prospective cohort	Esophageal cancer	0	1	0	0	0	0	0	0	0	0	0	0	0
Larrieu	2004	prospective cohort	Alzheimer's disease and other dementias	1	1	0	0	0	0	0	0	0	0	0	0	0
Larsson	2018	Prospective Cohort	Alzheimer's disease and other dementias	0	1	1	1	1	1	0	0	0	0	1	0	0
Le	1980	case-control	Breast cancer	1	1	0	0	0	0	0	0	0	0	0	0	0
Lee	2018	retrospective cohort	Larynx cancer	1	0	0	0	0	0	0	0	0	0	0	0	0
Lee	2017	prospective cohort	Stomach cancer	1	0	1	0	0	0	0	0	0	0	0	0	0
Lee	2015	prospective cohort	Atrial fibrillation and flutter	1	0	0	0	0	0	0	0	0	0	0	0	0
Leong	2003	case-control	Ischemic heart disease	1	1	0	1	0	0	0	0	0	0	0	0	0
Levi	1995	case-control	Breast cancer	1	1	0	0	0	1	0	0	0	0	0	0	0
Li	2015	prospective cohort	Diabetes mellitus type 2	1	1	0	0	0	0	0	0	0	0	0	0	0

Table S11. Bias covariates

Author	Year	Study design	Health outcome	incidence	age	sex	bmi	in-come	smoking	hyper-tension	APOE	other disease	diet	education	high loss to follow-up	prevalent disease population
Li	2012	prospective cohort	Diabetes mellitus type 2	1	1	0	0	0	0	0	0	0	0	0	0	0
Li	2005	prospective cohort	Breast cancer	1	1	0	0	0	1	0	0	0	0	0	0	0
Li	2004	prospective cohort	Breast cancer	1	1	0	0	0	1	0	0	0	0	0	0	0
Li	1999	case-control	Breast cancer	1	1	0	0	0	0	0	0	0	0	0	0	0
Lightfoot	2000	case-control	Colon and rectum cancer	1	1	0	0	0	0	0	0	0	0	0	0	0
Lin	1997	case-control	Esophageal cancer	1	0	0	0	0	0	0	0	0	0	0	0	0
Lu	2020	prospective cohort	Haemorrhagic stroke	0	1	0	0	0	0	0	0	0	0	0	0	0
Lu	2020	prospective cohort	Ischemic stroke	0	1	0	0	0	0	0	0	0	0	0	0	0
Lu	2008	prospective cohort	Diabetes mellitus type 2	1	0	0	0	0	0	0	0	0	0	0	0	0
Lubin	2011	case-control	Larynx cancer	1	1	0	0	0	0	0	0	0	0	0	0	0
Lubin	2011	case-control	Other pharynx cancer	0	1	0	0	0	0	0	0	0	0	0	0	0
Lucas	1993	case-control	Breast cancer	1	0	0	0	0	0	0	0	0	0	0	0	0
Lumey	1991	case-control	Prostate cancer	1	1	0	0	0	0	0	0	0	0	0	0	0
Lv	2013	prospective cohort	Diabetes mellitus type 2	1	1	0	0	0	0	0	0	0	0	0	0	0
Maasland	2003	prospective cohort	Larynx cancer	1	1	0	0	0	0	0	0	0	0	0	0	0

Table S11. Bias covariates

Author	Year	Study design	Health outcome	incidence	age	sex	bmi	in-come	smoking	hyper-tension	APOE	other disease	diet	education	high loss to follow-up	prevalent disease population
Malyutina	1998	prospective cohort	Ischemic heart disease	0	0	0	0	0	0	0	0	0	0	0	0	0
Marcus	2007	case-control	Atrial fibrillation and flutter	1	1	0	0	0	0	0	0	0	0	0	0	0
Martín-Pérez	2005	prospective cohort	Atrial fibrillation and flutter	0	1	0	0	0	0	0	0	0	0	0	0	0
Maty	1999	prospective cohort	Diabetes mellitus type 2	1	0	0	0	0	0	0	0	0	0	0	0	0
McDonald	1998	case-control	Breast cancer	1	1	0	0	0	0	0	0	0	0	0	0	0
McManus	2013	prospective cohort	Atrial fibrillation and flutter	1	1	0	0	0	0	0	0	0	0	0	0	0
Meara	1984	case-control	Breast cancer	1	1	0	0	0	1	0	0	0	0	0	0	0
Mejldal	2020	prospective cohort	Diabetes mellitus type 2	1	1	0	0	0	0	0	0	0	0	0	0	0
Menvielle	2007	case-control	Larynx cancer	1	1	0	0	0	0	0	0	0	0	0	0	0
Menvielle	1991	case-control	Larynx cancer	1	1	0	0	0	0	0	0	0	0	0	0	0
Meulepas	2007	case-control	Breast cancer	1	1	0	0	0	0	0	0	0	0	0	0	0
Mewton	2022	prospective cohort	Alzheimer's disease and other dementias	0	1	0	0	0	0	0	0	0	0	0	1	0
Millwood	2017	prospective cohort	Haemorrhagic stroke	1	1	0	0	0	0	0	0	0	0	0	0	0
Millwood	2017	prospective cohort	Ischemic stroke	1	1	0	0	0	0	0	0	0	0	0	0	0

Table S11. Bias covariates

Author	Year	Study design	Health outcome	incidence	age	sex	bmi	in-come	smoking	hyper-tension	APOE	other disease	diet	education	high loss to follow-up	prevalent disease population
Minami	2018	retrospective cohort	Liver cancer	1	1	0	0	0	0	0	0	0	0	0	0	0
Morch	2001	prospective cohort	Breast cancer	1	1	0	0	0	0	0	0	0	0	0	0	0
Morseth	2020	prospective cohort	Atrial fibrillation and flutter	1	1	0	0	0	0	0	0	0	0	0	0	0
Mukamal	2001	prospective cohort	Ischemic heart disease	0	1	0	1	0	0	0	0	0	0	0	0	0
Mukamal	2000	case-control	Ischemic heart disease	0	1	0	1	0	0	0	0	0	0	0	0	0
Mukamal	1998	prospective cohort	Ischemic heart disease	0	1	0	1	0	0	0	0	0	0	0	0	0
Muraki	1990	prospective cohort	Ischemic heart disease	0	1	0	1	0	0	0	0	0	0	0	0	0
Murata	1997	case-control	Colon and rectum cancer	1	1	0	0	0	0	0	0	0	0	0	0	0
Murray	1999	prospective cohort	Ischemic heart disease	0	1	0	0	0	0	0	0	0	0	0	0	0
Murray	1998	prospective cohort	Ischemic heart disease	0	1	0	1	0	0	0	0	0	0	0	0	0
Muscat	1990	case-control	Larynx cancer	1	0	0	0	0	0	0	0	0	0	0	0	0
Nagaya	2001	prospective cohort	Diabetes mellitus type 2	1	0	0	0	0	0	0	0	0	0	0	0	0
Nakanishi	2001	prospective cohort	Diabetes mellitus type 2	1	1	0	0	0	0	0	0	0	0	0	0	0
Nakaya	1997	prospective cohort	Colon and rectum cancer	1	1	0	0	0	0	0	0	0	0	1	0	0

Table S11. Bias covariates

Author	Year	Study design	Health outcome	incidence	age	sex	bmi	in-come	smoking	hyper-tension	APOE	other disease	diet	education	high loss to follow-up	prevalent disease population
Nakaya	1997	prospective cohort	Stomach cancer	1	1	0	0	0	0	0	0	0	0	0	0	0
Naudin	2000	prospective cohort	Pancreatic cancer	1	1	1	0	0	0	0	0	0	0	0	0	0
Newcomb	2000	case-control	Breast cancer	1	1	0	0	0	0	0	0	0	0	0	0	0
Newcomb	1991	case-control	Colon and rectum cancer	1	1	0	0	0	0	0	0	0	0	0	0	0
Nielsen	2002	prospective cohort	Breast cancer	1	1	0	0	0	1	0	0	0	0	0	0	0
Ogimoto	1999	prospective cohort	Liver cancer	0	0	0	0	0	0	0	0	0	0	0	0	0
Ohishi	2002	case-control	Liver cancer	1	0	0	0	0	0	0	0	0	0	0	0	0
Okamura	2016	prospective cohort	Diabetes mellitus type 2	1	1	0	0	0	0	0	0	0	0	0	0	0
Okita	1995	prospective cohort	Pancreatic cancer	1	1	0	0	0	0	0	0	0	0	0	0	0
Olmedo-Requena	2019	case-control	Prostate cancer	1	0	0	0	0	0	0	0	0	0	0	0	0
Ong	2015	prospective cohort	Breast cancer	1	0	0	0	0	0	0	0	0	0	0	0	0
Ozasa	2005	prospective cohort	Liver cancer	1	1	0	0	0	0	0	0	0	0	0	0	0
Ozasa	2005	prospective cohort	Pancreatic cancer	1	1	0	0	0	0	0	0	0	0	0	0	0
Palmer	1993	prospective cohort	Ischemic heart disease	0	1	0	0	0	0	0	0	0	0	0	0	0
Pandeya	2005	case-control	Esophageal cancer	1	1	0	0	0	0	0	0	0	0	0	0	0

Table S11. Bias covariates

Author	Year	Study design	Health outcome	incidence	age	sex	bmi	in-come	smoking	hyper-tension	APOE	other disease	diet	education	high loss to follow-up	prevalent disease population
Pang	2008	case-cohort	Pancreatic cancer	0	1	1	0	0	0	0	0	0	0	0	0	0
Park	2017	prospective cohort	Pancreatic cancer	1	1	1	0	0	0	0	0	0	0	0	0	0
Park	2012	prospective cohort	Atrial fibrillation and flutter	1	1	0	0	0	0	0	0	0	0	0	0	0
Pedersen	1993	prospective cohort	Colon and rectum cancer	1	1	0	0	0	0	0	0	0	0	0	0	0
Persson	2006	prospective cohort	Liver cancer	1	1	0	0	0	0	0	0	0	0	0	0	0
Peters	1982	prospective cohort	Colon and rectum cancer	1	1	0	0	0	0	0	0	0	0	1	0	0
Petrick	2010	case-cohort	Liver cancer	1	1	0	0	0	0	0	0	0	0	0	0	0
Prizment	2004	prospective cohort	Pancreatitis	0	1	0	0	0	0	0	0	0	0	0	0	0
Psaty	1993	prospective cohort	Atrial fibrillation and flutter	1	1	0	0	0	0	0	0	0	0	0	0	0
Pyong	1992	case-control	Liver cancer	1	0	0	0	0	0	0	0	0	0	0	0	0
Radoï	2007	case-control	Lip and oral cavity cancer	0	1	0	0	0	0	0	0	0	0	0	0	0
Radoï	2007	case-control	Lip and oral cavity cancer	0	0	0	0	0	0	0	0	0	0	0	0	0
Ramroth	2000	case-control	Larynx cancer	1	1	0	0	0	0	0	0	0	0	0	0	0
Reddiess	2017	prospective cohort	Ischemic heart disease	1	1	0	1	0	0	0	0	0	0	0	0	0
Renaud	1996	prospective cohort	Ischemic heart disease	0	1	0	1	0	0	0	0	0	0	0	0	0
Richardson	1986	case-control	Breast cancer	1	1	0	0	0	0	0	0	0	0	0	0	0

Table S11. Bias covariates

Author	Year	Study design	Health outcome	incidence	age	sex	bmi	in-come	smoking	hyper-tension	APOE	other disease	diet	education	high loss to follow-up	prevalent disease population
Rist	2008	prospective cohort	Ischemic stroke	1	0	0	0	0	0	0	0	0	0	0	0	0
Rohrmann	2000	prospective cohort	Prostate cancer	1	1	0	0	0	0	0	0	0	0	0	0	0
Rosenberg	1995	case-control	Breast cancer	1	1	0	0	0	0	0	0	0	0	0	0	0
Rosenberg	1986	case-control	Breast cancer	1	1	0	0	0	1	0	0	0	0	0	0	0
Ruidavets	2004	prospective cohort	Ischemic heart disease	1	0	0	0	0	0	0	0	0	0	0	0	0
Ruigomez	1997	case-control	Atrial fibrillation and flutter	1	1	0	0	0	0	0	0	0	0	0	0	0
Ruitenberg	1999	prospective cohort	Alzheimer's disease and other dementias	1	1	0	0	0	0	0	0	0	0	0	0	0
Rustagi	2013	case-control	Breast cancer	1	1	0	0	0	0	0	0	0	0	0	0	0
Sabia	2017	prospective cohort	Alzheimer's disease and other dementias	1	1	0	0	0	0	0	0	0	0	0	0	0
Sacco	1997	case-control	Ischemic stroke	1	1	0	0	1	0	0	0	0	0	0	0	0
Sankai	1993	prospective cohort	Haemorrhagic stroke	0	1	0	0	0	0	0	0	0	0	0	0	0
Santi	2000	case-control	Larynx cancer	1	1	0	0	0	0	0	0	0	0	0	0	0
Santi	2000	case-control	Larynx cancer	1	0	0	0	0	0	0	0	0	0	0	0	0
Sarich	2013	prospective cohort	Breast cancer	1	1	0	0	0	1	0	0	0	0	0	0	0
Sarich	2013	prospective cohort	Colon and rectum cancer	1	1	0	0	0	0	0	0	0	0	1	0	0

Table S11. Bias covariates

Author	Year	Study design	Health outcome	incidence	age	sex	bmi	in-come	smoking	hyper-tension	APOE	other disease	diet	education	high loss to follow-up	prevalent disease population
Sarich	2013	prospective cohort	Liver cancer	1	1	0	0	0	0	0	0	0	0	0	0	0
Sarich	2013	prospective cohort	Pancreatic cancer	1	1	1	0	0	0	0	0	0	0	0	0	0
Sarich	2013	prospective cohort	Pancreatic cancer	1	0	0	0	0	0	0	0	0	0	0	0	0
Sarich	2013	prospective cohort	Prostate cancer	1	1	1	0	0	0	0	0	0	0	0	0	0
Schooling	2003	prospective cohort	Ischemic heart disease	0	0	0	0	0	0	0	0	0	0	0	0	0
Schoonen	1996	case-control	Prostate cancer	1	1	0	0	0	0	0	0	0	0	0	0	0
Schutte	2019	prospective cohort	Ischemic heart disease	1	1	0	1	0	0	0	0	0	0	0	0	0
Schuurman	1992	case-cohort	Prostate cancer	1	1	0	0	0	0	0	0	0	0	0	0	0
Schwartz	2009	prospective cohort	Liver cancer	1	1	0	0	0	0	0	0	0	0	0	0	0
Seol	2004	case-control	Colon and rectum cancer	1	1	0	0	0	0	0	0	0	0	1	0	0
Setiawan	2013	prospective cohort	Cirrhosis and other chronic liver diseases	0	1	0	0	0	0	0	0	0	0	0	0	0
Setiawan	2012	prospective cohort	Pancreatitis	0	0	0	0	0	0	0	0	0	0	0	0	0
Shanmugham	2006	prospective cohort	Lip and oral cavity cancer	0	1	0	0	0	0	0	0	0	0	0	0	0
Shaper	1980	prospective cohort	Ischemic heart disease	0	0	0	0	0	0	0	0	0	0	0	0	0

Table S11. Bias covariates

Author	Year	Study design	Health outcome	incidence	age	sex	bmi	in-come	smoking	hyper-tension	APOE	other disease	diet	education	high loss to follow-up	prevalent disease population
Sharpe	1985	case-control	Colon and rectum cancer	1	1	0	0	0	0	0	0	0	0	1	0	0
Shimazu	2004	prospective cohort	Liver cancer	1	1	0	0	0	0	0	0	0	0	0	0	0
Shimizu	2023	Prospective Cohort	Alzheimer's disease and other dementias	0	1	1	1	0	1	1	0	0	0	1	0	0
Siddiqi	2020	prospective cohort	Atrial fibrillation and flutter	1	1	0	0	0	0	0	0	0	0	0	0	0
Simon	1987	prospective cohort	Breast cancer	1	1	0	0	0	1	0	0	0	0	0	0	0
Smith	2017	prospective cohort	Ischemic heart disease	1	1	0	1	0	0	0	0	0	0	0	0	0
Smith	1985	case-control	Breast cancer	1	1	0	0	0	1	0	0	0	0	0	0	0
Sneyd	1987	case-control	Breast cancer	1	1	0	0	0	1	0	0	0	0	0	0	0
Soh	2014	prospective cohort	Tuberculosis	0	1	0	0	0	0	0	0	0	0	0	0	0
Song	2013	case-control	Breast cancer	1	1	0	0	0	1	0	0	0	0	0	0	0
Steevens	2002	prospective cohort	Esophageal cancer	1	1	0	0	0	0	0	0	0	0	0	0	0
Steevens	2002	prospective cohort	Stomach cancer	1	1	1	0	0	0	0	0	0	0	0	0	0
Stemhagen	1980	case-control	Liver cancer	1	1	0	0	0	0	0	0	0	0	0	0	0
Stern	2002	case-control	Colon and rectum cancer	1	0	0	0	0	0	0	0	0	0	0	0	0
Strodl	1999	prospective cohort	Diabetes mellitus type 2	1	0	0	0	0	0	0	0	0	0	0	0	0

Table S11. Bias covariates

Author	Year	Study design	Health outcome	incidence	age	sex	bmi	in-come	smoking	hyper-tension	APOE	other disease	diet	education	high loss to follow-up	prevalent disease population
Sung	2002	prospective cohort	Stomach cancer	1	1	0	0	0	0	0	0	0	0	0	0	0
Sutcliffe	2002	prospective cohort	Prostate cancer	1	1	0	0	0	0	0	0	0	0	0	0	0
Suzuki	2006	prospective cohort	Breast cancer	1	1	0	0	0	1	0	0	0	0	0	0	0
Suzuki	2004	prospective cohort	Breast cancer	1	1	0	0	0	0	0	0	0	0	0	0	0
Tao	2018	prospective cohort	Cirrhosis and other chronic liver diseases	1	0	0	0	0	0	0	0	0	0	0	0	0
Thun	1991	prospective cohort	Breast cancer	0	1	0	0	0	1	0	0	0	0	0	0	0
Tjonneland	2004	prospective cohort	Breast cancer	1	1	0	0	0	1	0	0	0	0	0	0	0
Tolstrup	2004	case-cohort	Ischemic heart disease	0	0	0	1	0	0	0	0	0	0	0	0	0
Toniolo	1984	case-control	Breast cancer	1	1	0	0	0	0	0	0	0	0	0	0	0
Topiwala	2025	Prospective Cohort	Alzheimer's disease and other dementias	0	1	1	1	1	1	0	0	0	0	1	0	0
Tota	2015	case-control	Other pharynx cancer	0	1	0	0	0	0	0	0	0	0	0	0	0
Tsong	2004	prospective cohort	Colon and rectum cancer	1	1	0	0	0	0	0	0	0	0	1	0	0
Tu	2020	prospective cohort	Atrial fibrillation and flutter	0	1	0	0	0	0	0	0	0	0	0	0	0

Table S11. Bias covariates

Author	Year	Study design	Health outcome	incidence	age	sex	bmi	in-come	smoking	hyper-tension	APOE	other disease	diet	education	high loss to follow-up	prevalent disease population
Turati	2008	case-control	Other pharynx cancer	0	0	0	0	0	0	0	0	0	0	0	0	0
Tverdal	2018	prospective cohort	Colon and rectum cancer	1	1	0	0	0	0	0	0	0	0	1	0	0
Valmadrid	1996	prospective cohort	Ischemic heart disease	0	1	0	0	0	0	0	0	0	0	0	0	0
van den Brandt	1989	case-cohort	Breast cancer	1	1	0	0	0	1	0	0	0	0	0	0	0
van't Veer	1987	case-control	Breast cancer	1	1	0	0	0	1	0	0	0	0	0	0	0
Velicer	2002	prospective cohort	Prostate cancer	1	1	0	0	0	0	0	0	0	0	0	0	0
Viladiu	1989	case-control	Breast cancer	1	1	0	0	0	0	0	0	0	0	0	0	0
Vliegenthart	2003	prospective cohort	Ischemic heart disease	1	1	0	1	0	0	0	0	0	0	0	0	0
Waki	2000	prospective cohort	Diabetes mellitus type 2	1	0	0	0	0	0	0	0	0	0	0	0	0
Walsh	2001	prospective cohort	Ischemic heart disease	1	1	0	1	0	0	0	0	0	0	0	0	0
Wändell	2004	prospective cohort	Diabetes mellitus type 2	1	0	0	0	0	0	0	0	0	0	0	0	0
Wang	2004	case-control	Breast cancer	1	0	0	0	0	0	0	0	0	0	0	0	0
Wang	2004	case-control	Colon and rectum cancer	1	0	0	0	0	0	0	0	0	0	0	0	0
Wang	2004	case-control	Prostate cancer	1	0	0	0	0	0	0	0	0	0	0	0	0
Wannamethee	2010	prospective cohort	Ischemic heart disease	1	1	0	1	0	0	0	0	0	0	0	0	0

Table S11. Bias covariates

Author	Year	Study design	Health outcome	incidence	age	sex	bmi	in-come	smoking	hyper-tension	APOE	other disease	diet	education	high loss to follow-up	prevalent disease population
Watters	2003	prospective cohort	Prostate cancer	1	1	0	0	0	0	0	0	0	0	0	0	0
Wellmann	1997	prospective cohort	Ischemic heart disease	0	1	0	1	0	0	0	0	0	0	0	0	0
Werbrouck	2006	case-control	Lip and oral cavity cancer	0	0	0	0	0	0	0	0	0	0	0	0	0
Werbrouck	2006	case-control	Other pharynx cancer	0	0	0	0	0	0	0	0	0	0	0	0	0
Weyerer	2010	prospective cohort	Alzheimer's disease and other dementias	1	1	0	0	0	0	0	0	0	0	0	0	0
Wu	2007	case-control	Esophageal cancer	1	1	0	0	0	0	0	0	0	0	0	0	0
Wu	1985	prospective cohort	Colon and rectum cancer	1	1	0	0	0	0	0	0	0	0	0	0	0
Yallew	2009	case-control	Pancreatic cancer	1	1	1	0	0	0	0	0	0	0	0	0	0
Yamada	1993	case-control	Colon and rectum cancer	1	1	0	0	0	0	0	0	0	0	0	0	0
Yang	2013	prospective cohort	Ischemic stroke	1	1	0	0	0	0	0	0	0	0	0	0	0
Yang	2007	retrospective cohort	Pancreatitis	0	1	0	0	0	0	0	0	0	0	0	0	0
Yang	2005	prospective cohort	Esophageal cancer	0	1	0	0	0	0	0	0	0	0	0	0	0
Yang	2004	case-control	Esophageal cancer	1	0	0	0	0	0	0	0	0	0	0	0	0
Yi	2013	prospective cohort	Liver cancer	1	1	0	0	0	0	0	0	0	0	0	0	0

Table S11. Bias covariates

Author	Year	Study design	Health outcome	incidence	age	sex	bmi	in-come	smoking	hyper-tension	APOE	other disease	diet	education	high loss to follow-up	prevalent disease population
Yi	2005	prospective cohort	Colon and rectum cancer	0	1	0	0	0	0	0	0	0	0	1	0	0
Yi	2005	prospective cohort	Esophageal cancer	0	1	0	0	0	0	0	0	0	0	0	0	0
Yi	2005	prospective cohort	Liver cancer	0	1	0	0	0	0	0	0	0	0	0	0	0
Yu	2015	prospective cohort	Esophageal cancer	0	1	0	0	0	0	0	0	0	0	0	0	0
Yuan	2001	prospective cohort	Liver cancer	1	1	0	0	0	0	0	0	0	0	0	0	0
Zeinomar	2019	prospective cohort	Breast cancer	0	1	0	0	0	1	0	0	0	0	0	0	0
Zhang	2005	case-control	Breast cancer	1	1	0	0	0	0	0	0	0	0	0	0	0
Zhang	1995	case-control	Liver cancer	1	1	0	0	0	0	0	0	0	0	0	0	0
Zhao	2017	case-control	Esophageal cancer	1	1	0	0	0	0	0	0	0	0	0	0	0
Zheng	1990	case-control	Larynx cancer	1	1	0	0	0	0	0	0	0	0	0	0	0

X. Model Specifications

A. MR-BRT Model Specifications by Risk-Outcome Pair

Table S12. MR-BRT spline and prior specifications

Health outcome	Spline degree, number of interior knots	Priors & constraints
Cirrhosis and other chronic liver diseases	Quadratic, 2 interior knots	Monotonic increasing, right linear tail, Gaussian max derivative prior on the right tail (0, 0.001)
Atrial fibrillation and flutter	Quadratic, 2 interior knots	Monotonic increasing, right linear tail, Gaussian max derivative prior on the right tail (0, 0.001)
Ischemic heart disease	Quadratic, 2 interior knots	Left linear tail, Gaussian max derivative prior on the right tail (0, 0.001)
Hemorrhagic stroke	Quadratic, 2 interior knots	Right linear tail, Gaussian max derivative prior on the right tail (0, 0.001)
Ischemic stroke	Quadratic, 2 interior knots	Right linear tail, Gaussian max derivative prior on the right tail (0, 0.001)
Type 2 diabetes	Quadratic, 2 interior knots	Right linear tail, Gaussian max derivative prior on the right tail (0, 0.001)
Pancreatitis	Quadratic, 2 interior knots	Monotonic increasing, Gaussian max derivative prior on the right tail (0, 0.001)
Lower respiratory infections	Quadratic, 2 interior knots	Monotonic increasing, Gaussian max derivative prior on the right tail (0, 0.001)
Breast cancer	Quadratic, 2 interior knots	Monotonic increasing, right linear tail, Gaussian max derivative prior on the right tail (0, 0.001)
Colon and rectum cancer	Quadratic, 2 interior knots	Monotonic increasing, right linear tail, Gaussian max derivative prior on the right tail (0, 0.001)
Esophageal cancer	Quadratic, 2 interior knots	Monotonic increasing, right linear tail, Gaussian max derivative prior on the right tail (0, 0.001)
Larynx cancer	Quadratic, 2 interior knots	Monotonic increasing, right linear tail, Gaussian max derivative prior on the right tail (0, 0.001)
Liver cancer	Quadratic, 2 interior knots	Monotonic increasing, right linear tail, Gaussian max derivative prior on the right tail (0, 0.001)
Lip and oral cavity cancer	Quadratic, 2 interior knots	Monotonic increasing, Gaussian max derivative prior on the right tail (0, 0.001)

Table S12. MR-BRT spline and prior specifications

Health outcome	Spline degree, number of interior knots	Priors & constraints
Other pharynx cancer	Quadratic, 2 interior knots	Monotonic increasing, right linear tail, Gaussian max derivative prior on the right tail (0, 0.001)
Pancreatic cancer	Quadratic, 2 interior knots	Monotonic increasing, right linear tail, Gaussian max derivative prior on the right tail (0, 0.001)
Prostate cancer	Quadratic, 2 interior knots	Monotonic increasing, right linear tail, Gaussian max derivative prior on the right tail (0, 0.001)
Stomach cancer	Quadratic, 2 interior knots	Monotonic increasing, right linear tail, Gaussian max derivative prior on the right tail (0, 0.001)
Alzheimer's disease and other dementias	Quadratic, 2 interior knots	No constraint, Gaussian max derivative prior on the right tail (0, 0.001)
Tuberculosis	Quadratic, 2 interior knots	Monotonic increasing, right linear tail, Gaussian max derivative prior on the right tail (0, 0.001)

B. Selected Bias Covariates and Gamma Solution by Risk-Outcome Pair

Table S13. Selected bias covariates and gamma solution

Health outcome	Selected bias covariates	Gamma solution (mean and standard deviation)
Cirrhosis and other chronic liver diseases	age, incidence	0.02 (0.03)
Atrial fibrillation and flutter	incidence, age	0.02 (0.03)
Ischemic heart disease	None	0.25 (0.06)
Hemorrhagic stroke	sex	0.38 (0.22)
Ischemic stroke	None	0.4 (0.2)
Type 2 diabetes	None	0.08 (0.08)
Pancreatitis	None	0 (0.02)
Lower respiratory infections	None	0 (0.09)
Breast cancer	None	0 (0.02)
Colon and rectum cancer	incidence	0 (0.02)
Esophageal cancer	age	0.24 (0.07)
Larynx cancer	age, incidence	0.03 (0.04)
Liver cancer	incidence, age	0.35 (0.14)
Lip and oral cavity cancer	None	0.03 (0.04)
Other pharynx cancer	None	0 (0)
Pancreatic cancer	None	0 (0.07)
Prostate cancer	None	0 (0.09)
Stomach cancer	None	0 (0.24)
Alzheimer's disease and other dementias	None	0.02 (0.03)
Tuberculosis	age, representativeness	0.12 (0.15)

C. Definitions of Considered Bias Covariates

Table S14. Definitions of considered bias covariates

Bias Covariate Name	Details
age	Study controls for age in relative risk estimate.<br>(0 = controls for age, 1= does not control for age)
incidence	Study reports relative risk for incidence of outcome<br>(0= outcome is incidence, 1=outcome is not incidence)
representativeness	Study population is/is not representative of location<br>(0= population is representative, 1= population is not representative)
sex	Study controls for sex in relative risk estimate<br>(0= study controls for sex, 1= study does not control for sex)
followup_excl	Study had substantial (>40%) number of participants that were lost to follow up or the group lost to follow-up was significantly different than the overall group (e.g. one sex, older age, etc.)<br>(0= not significant loss to follow up, 1=significant loss to follow up)
hypertension	Study controls for hypertension in relative risk estimate<br>(0= study controls for hypertension, 1= study does not control for hypertension)
bmi	Study controls for bmi (body mass index) in relative risk estimate<br>(0= study controls for bmi, 1= study does not control for bmi)
income	Study controls for income in relative risk estimate<br>(0= study controls for income, 1= study does not control for income)
smoking	Study controls for smoking in relative risk estimate<br>(0= study controls for smoking, 1= study does not control for smoking)
APOE	Study controls for APOE genetic marker in relative risk estimate<br>(0= study controls for APOE marker, 1= study does not control for APOE marker)
other disease	Study controls for other (non-hypertension, not diabetes) disease in relative risk estimate<br>(0= study controls for other disease, 1= study does not control for other disease)
diet	Study controls for total calorie intake or other major dietary factors in relative risk estimate

Table S14. Definitions of considered bias covariates

Bias Covariate Name	Details
	(0= study controls for diet, 1= study does not control for diet)
education	Study controls for education in relative risk estimate<br><br>(0= study controls for education, 1= study does not control for education)

XI. Sensitivity Analyses

A. Model Settings

For each outcome, four model settings were tested: no enforced parameters, right linear tail enforced for all outcomes, monotonicity enforced (for non-J-shaped curves), right linear tail and monotonicity enforced (for non-J-shaped curves), left linear tail enforced (J-shaped curves), left and right linear tails enforced (J-shaped curves). These models were compared to select the parameters with the best model fit.

The following risk curves are computed relative to no alcohol consumption. In panels (a) and (b), the dark line indicates mean relative risk across alcohol consumption levels (in g/day); the light and dark shading show 95% uncertainty intervals with and without between-study heterogeneity, respectively, with the red line highlighting the burden of proof function; the size of the data points corresponds to the inverse of the standard error, with those trimmed during the model fitting process marked by a red x; and the dashed lines represent the 15th percentile of the reference exposure and the 85th percentile of the alternative exposure. To visualize log-relative- risk points in panel (a), we plotted each data point with the x-value at the midpoint of the alternative group and the y-value offset by the difference between the reported and predicted log risk. In panel (b), we exponentiated the y-values from panel (a) to yield the relative risk curve. Panel (c) depicts a customized funnel plot, with the x-axis representing residuals between predicted and observed relative risks, and the y-axis representing uncertainty from both measurement error and between-study heterogeneity.

RR relative risk, UI uncertainty interval.

Figure S2.01. Atrial fibrillation

No parameters enforced

Score: 0.03, Star Rating: 2

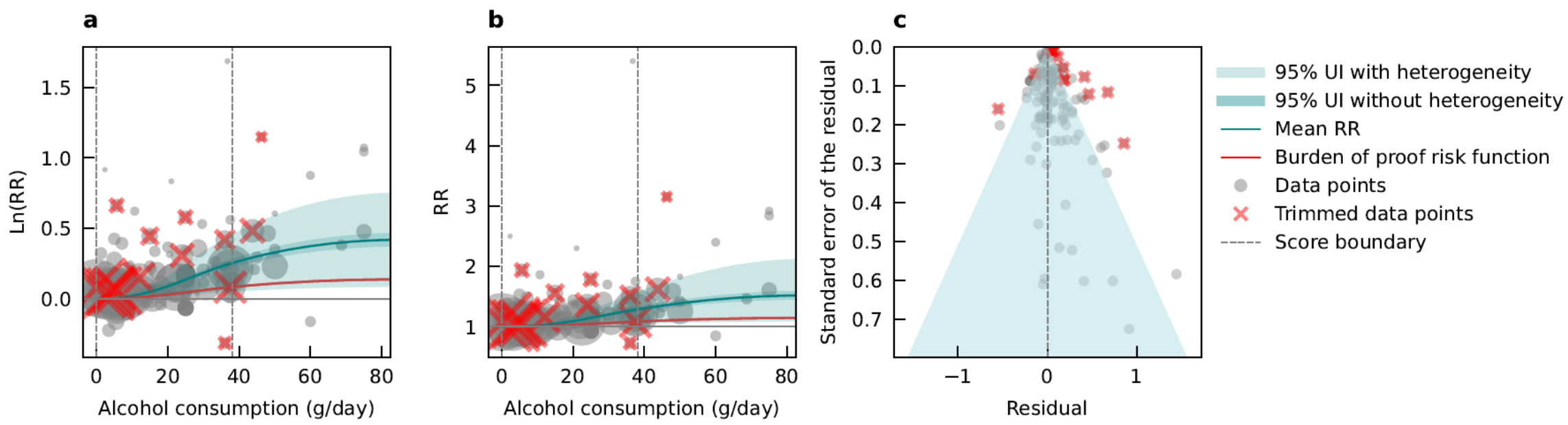

Right linear tail enforced

Score: 0.04, Star Rating: 2

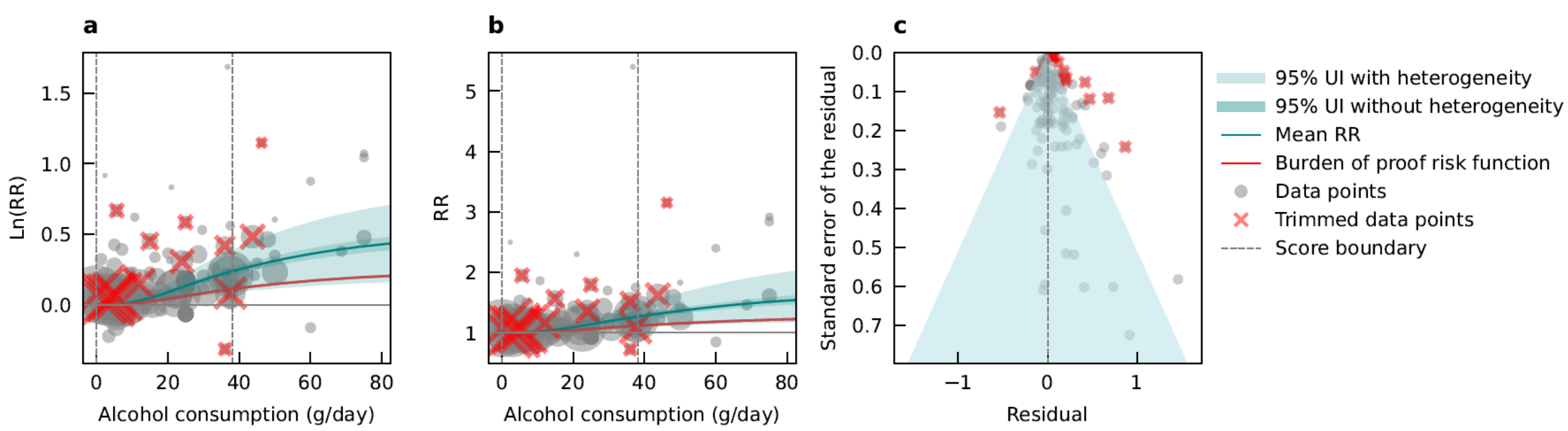

Monotonicity increasing enforced

Score: 0.06, Star Rating: 2

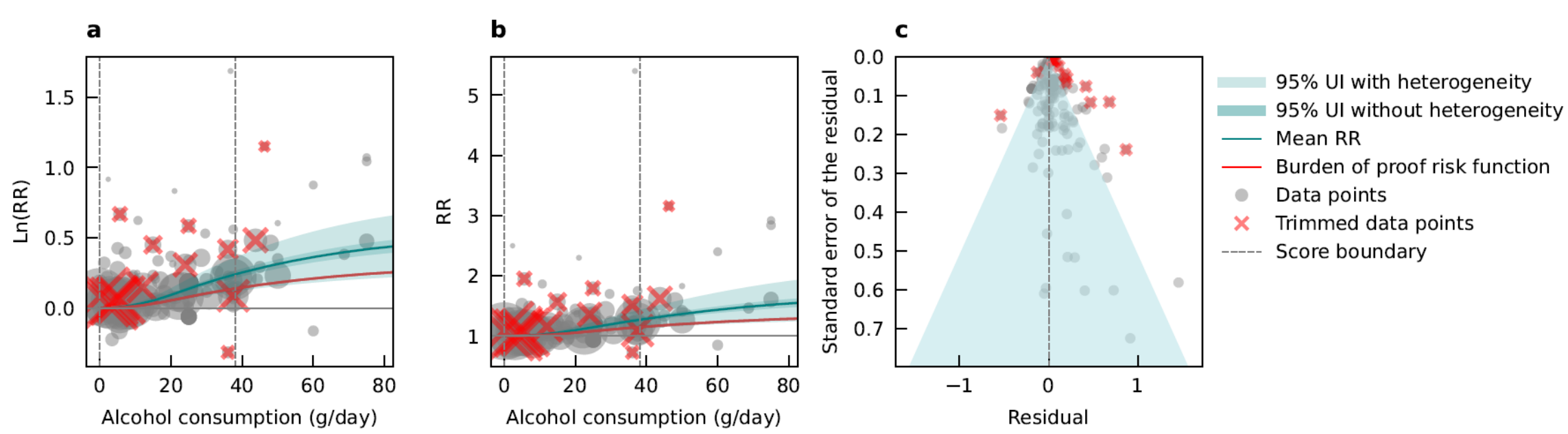

Right linear tail and monotonicity increasing enforced

Score: 0.06, Star Rating: 2

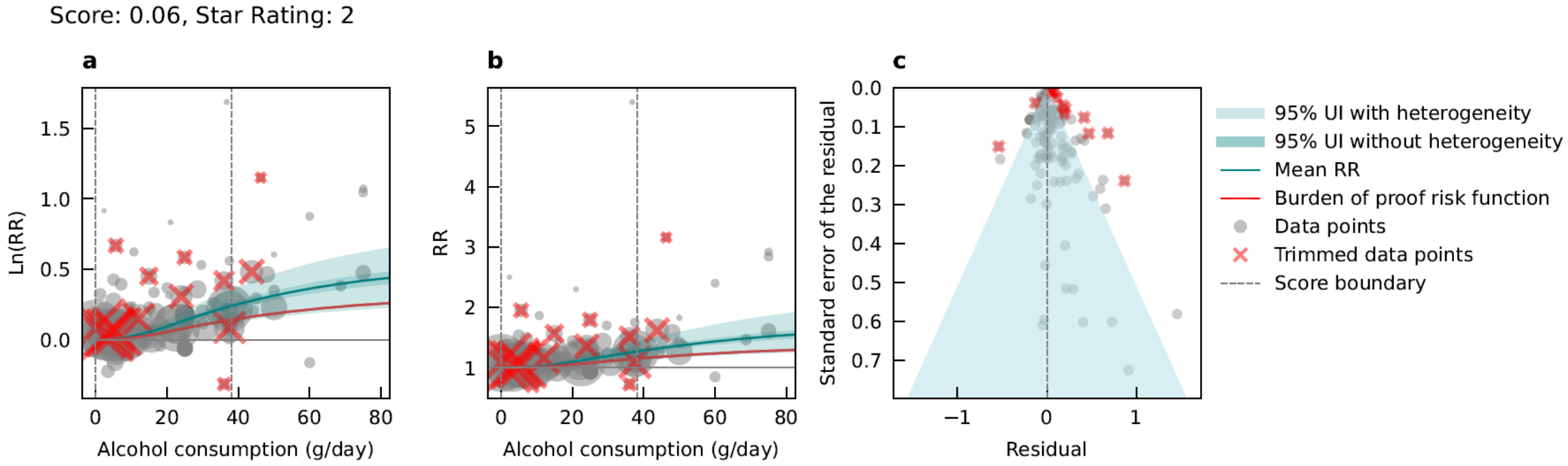

Figure S2.02. Breast cancer

No parameters enforced

Score: 0.1, Star Rating: 2

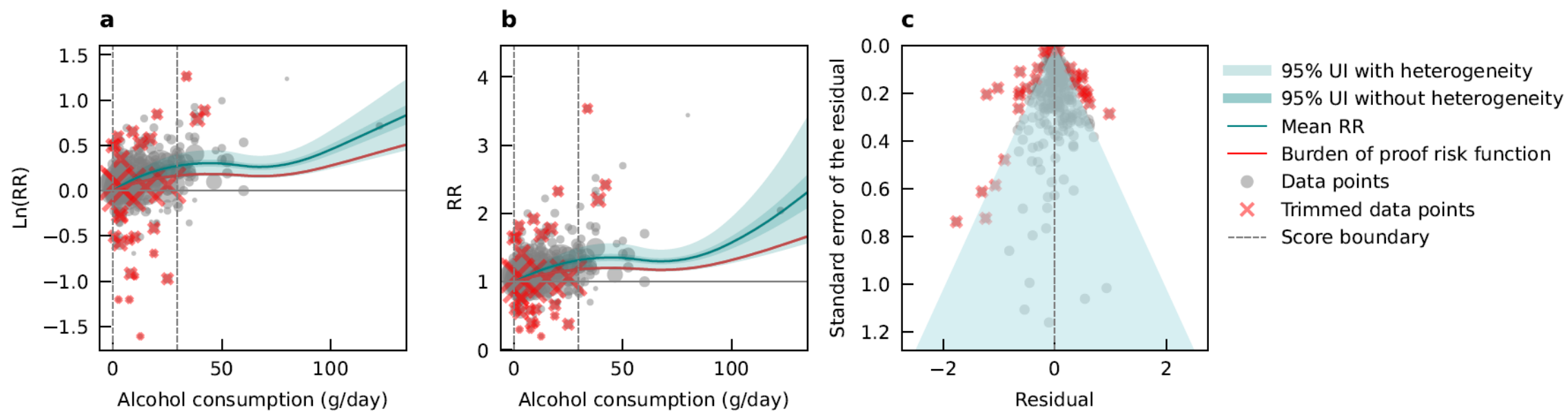

Right linear tail enforced

Score: 0.11, Star Rating: 2

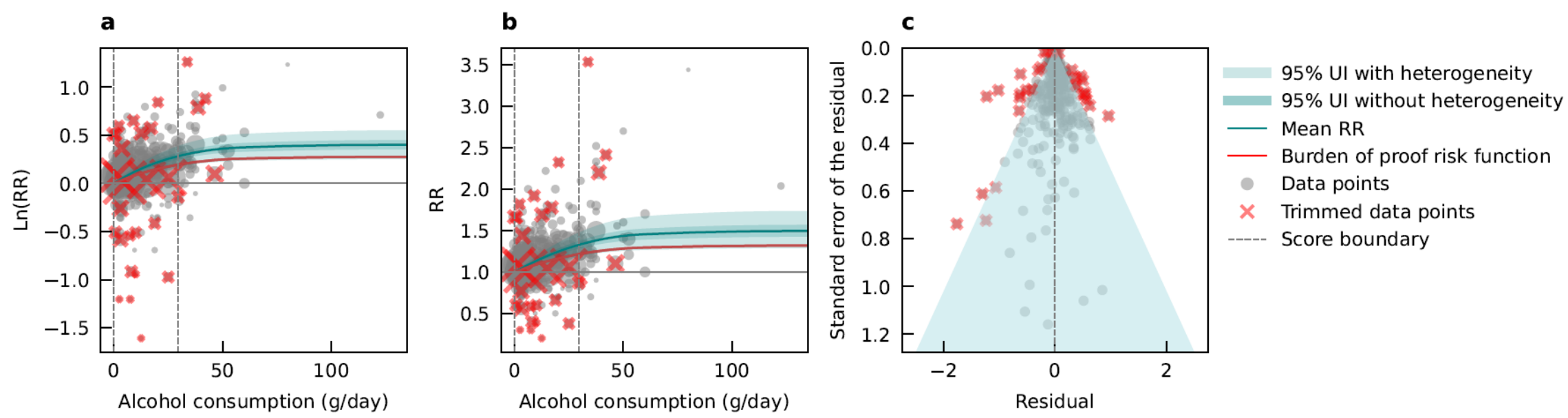

Monotonicity increasing enforced

Score: 0.11, Star Rating: 2

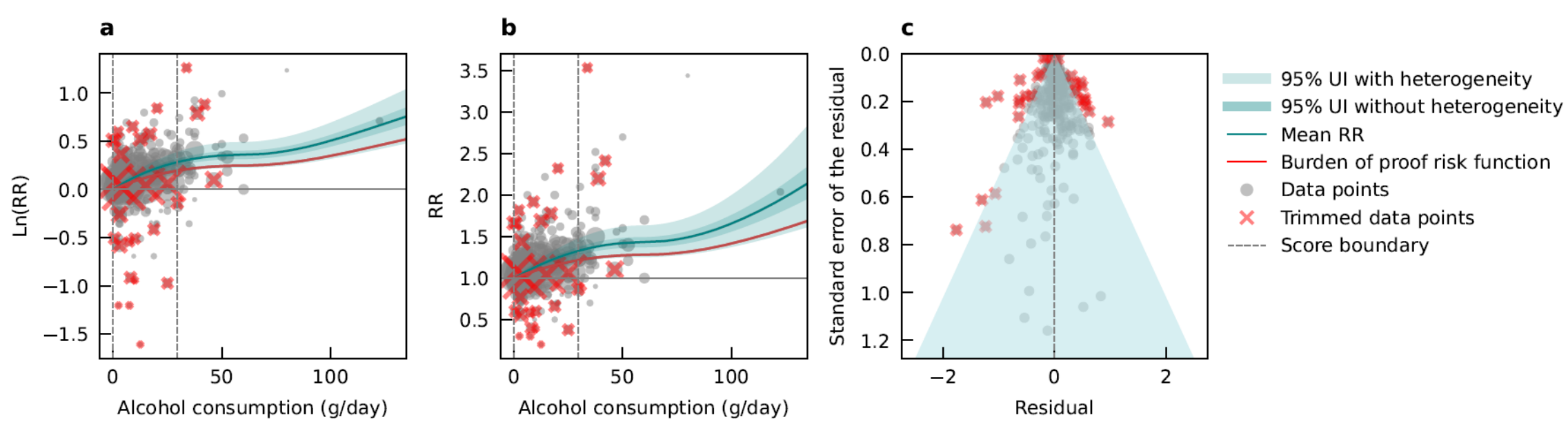

Right linear tail and monotonicity increasing enforced

Score: 0.11, Star Rating: 2

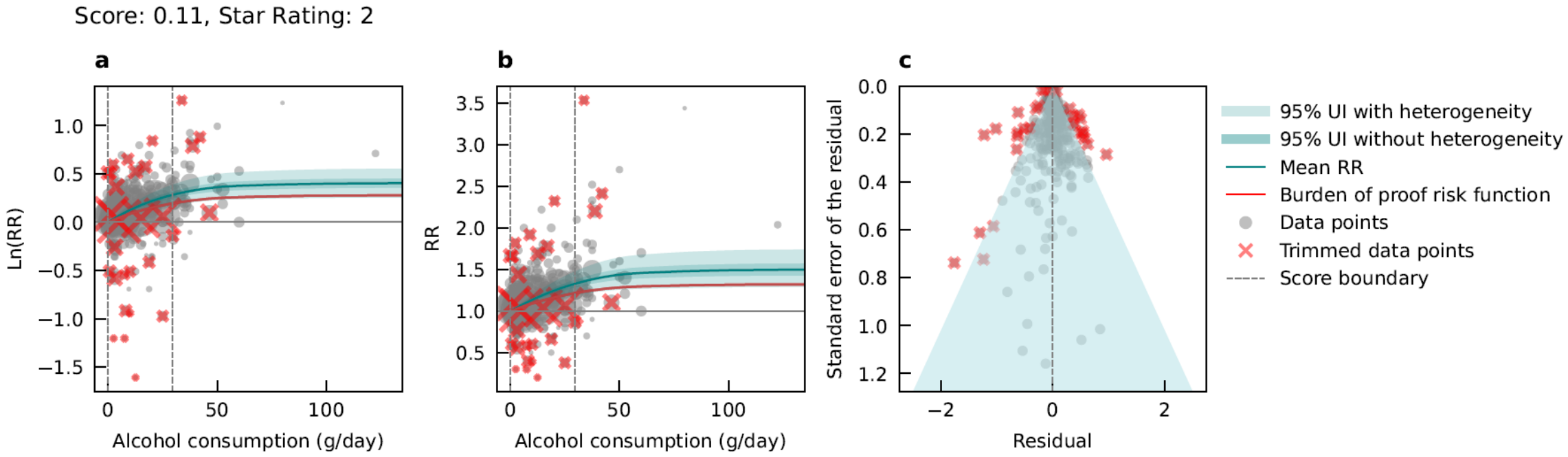

Figure S2.03. Cirrhosis and other chronic liver diseases

No parameters enforced

Score: 0.28, Star Rating: 3

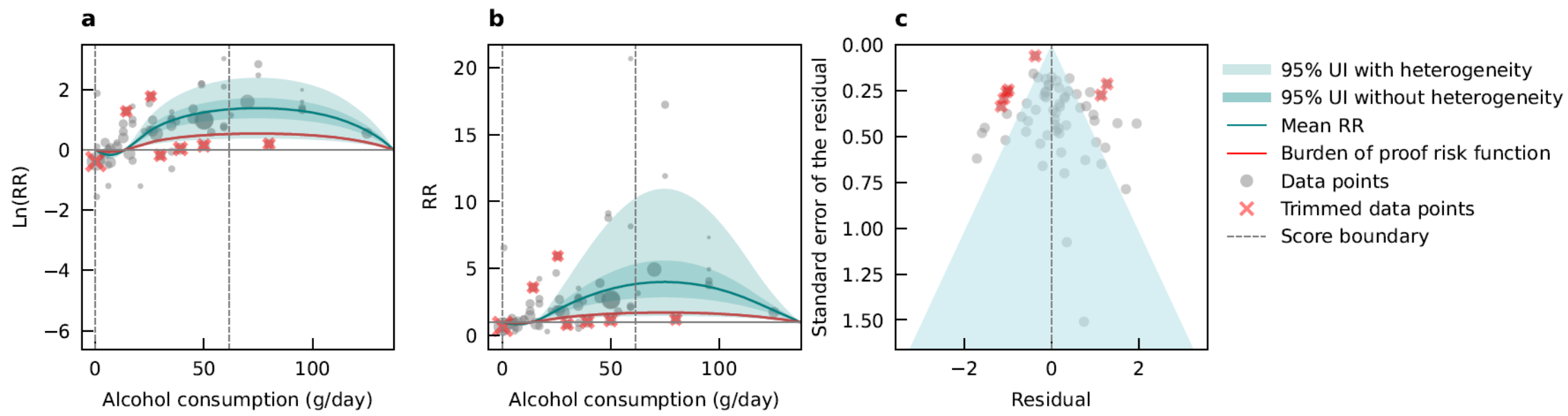

Right linear tail enforced

Score: 0.01, Star Rating: 2

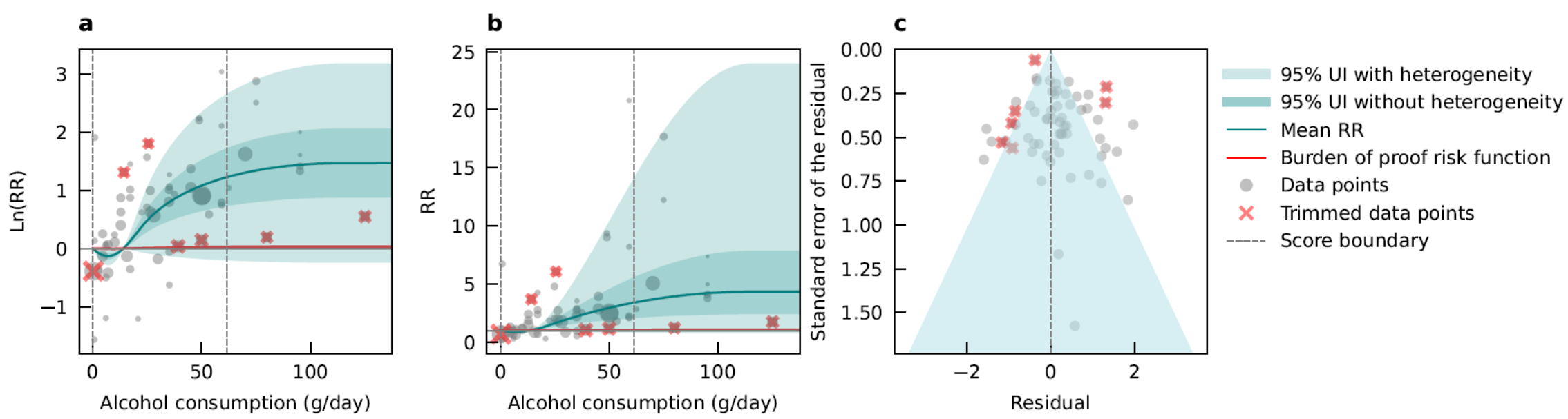

Monotonicity increasing enforced

Score: 0.3, Star Rating: 3

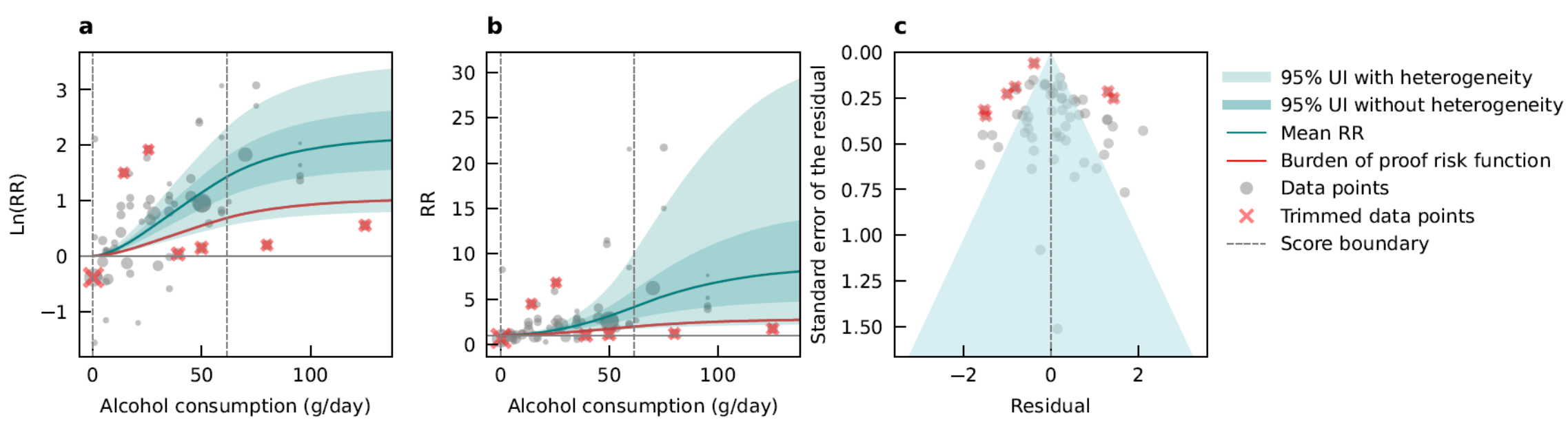

Right linear tail and monotonicity increasing enforced

Score: 0.34, Star Rating: 3

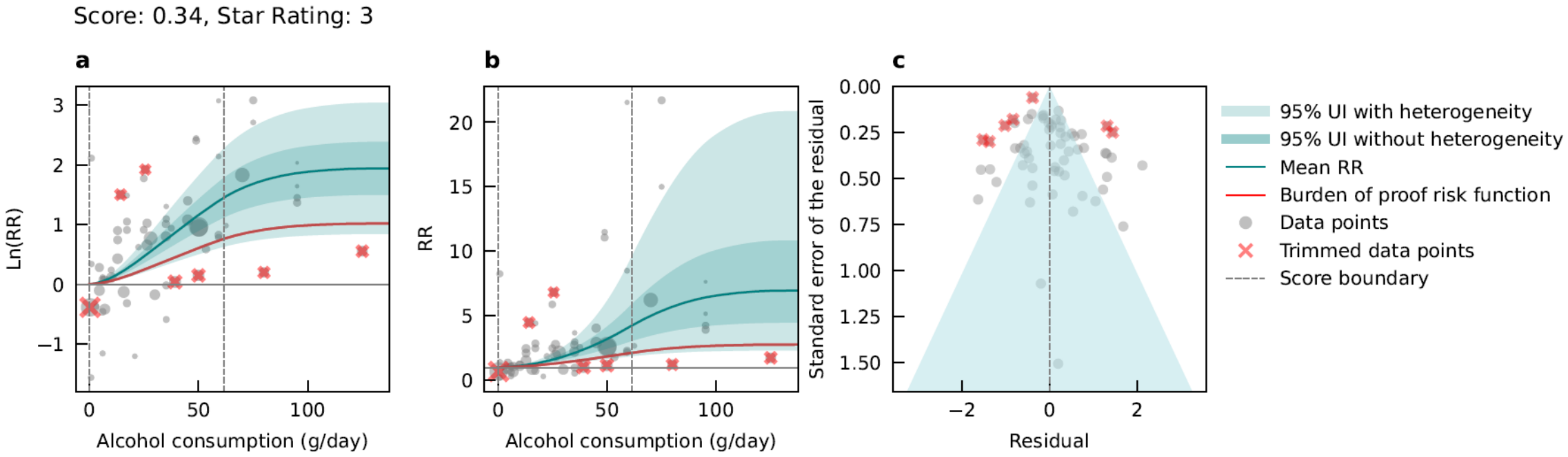

Figure S2.04. Colorectal cancer

No parameters enforced

Score: 0.15, Star Rating: 3

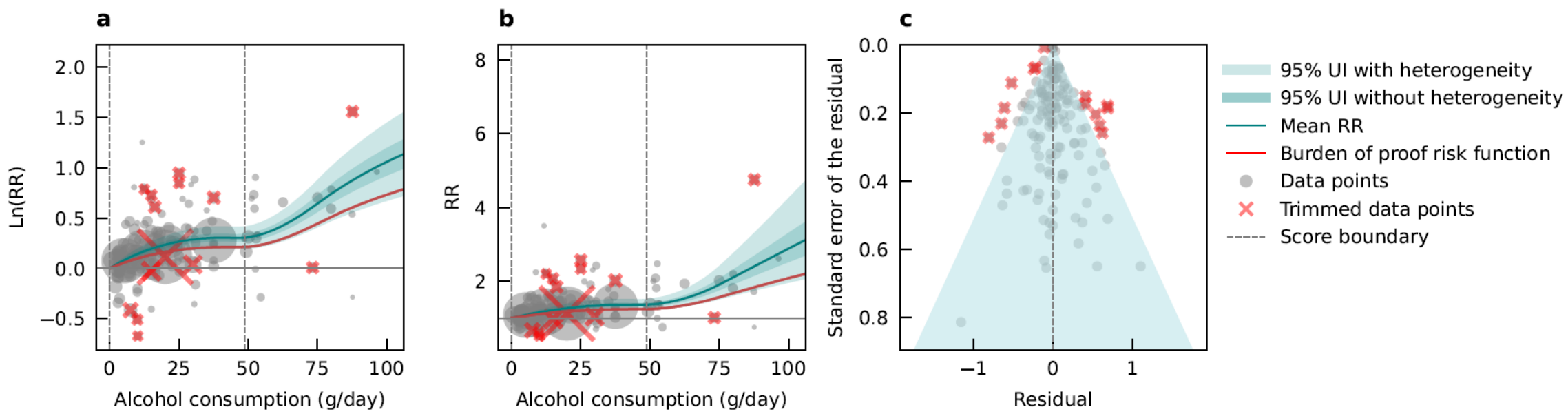

Right linear tail enforced

Score: 0.17, Star Rating: 3

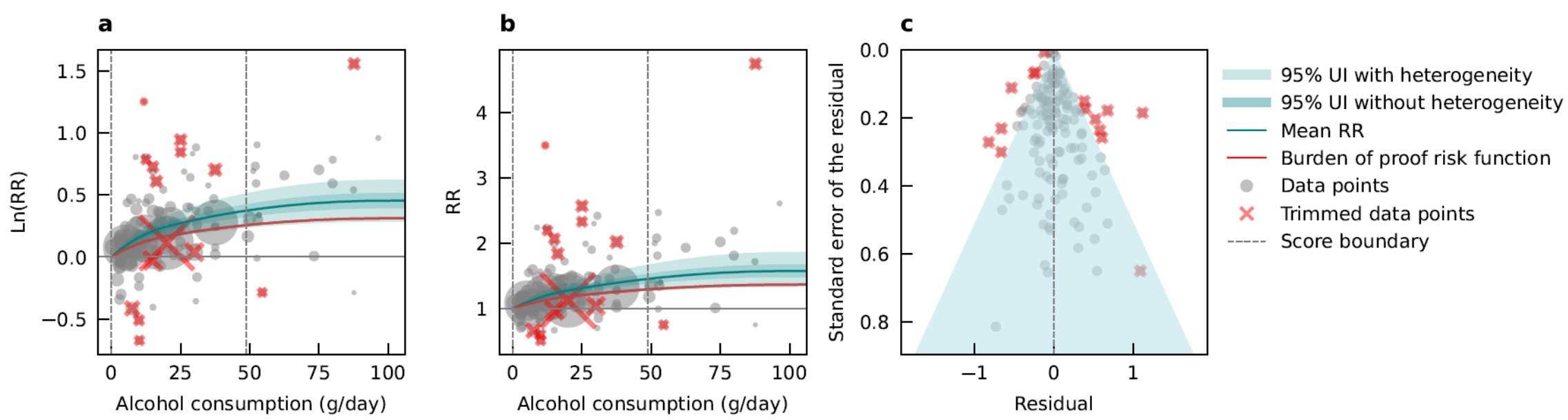

Monotonicity increasing enforced

Score: 0.16, Star Rating: 3

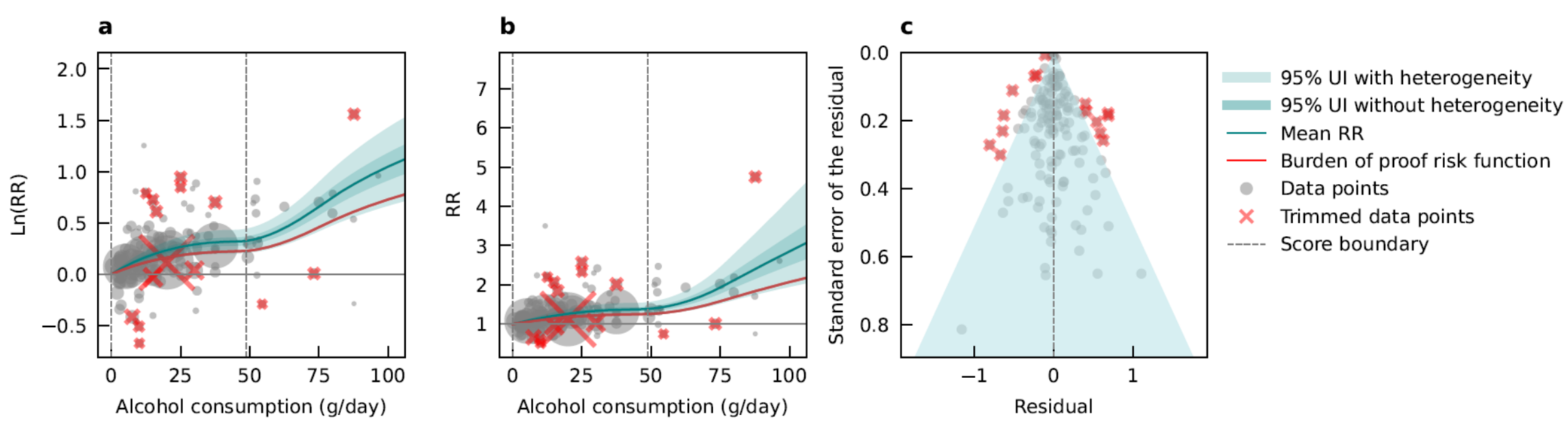

Right linear tail and monotonicity increasing enforced

Score: 0.17, Star Rating: 3

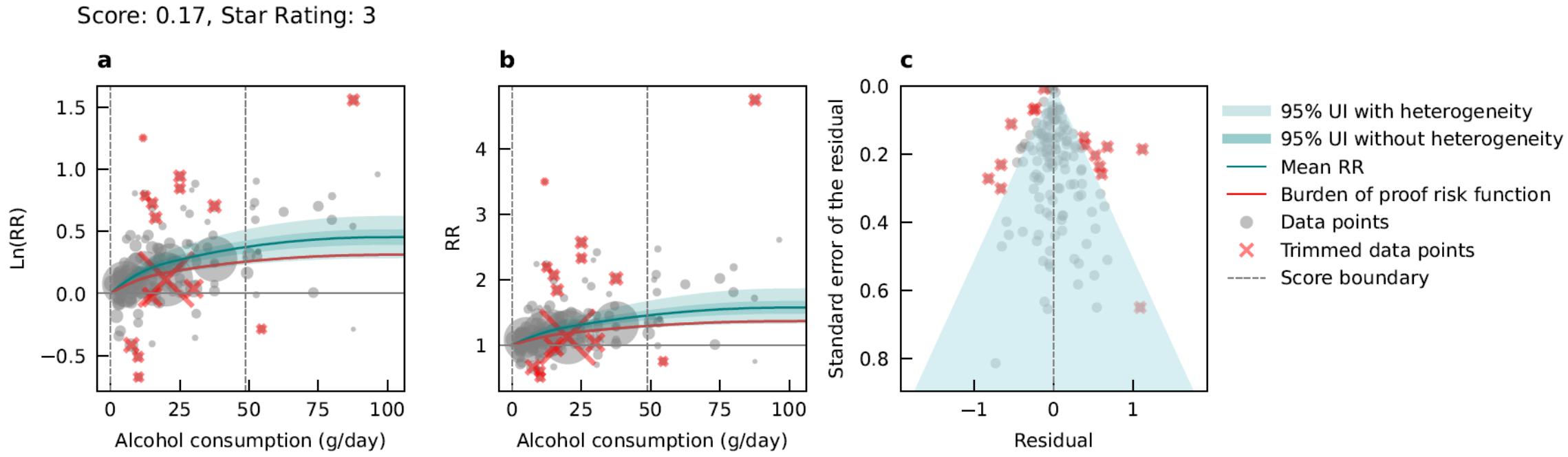

Figure S2.05. Alzheimer's disease and other dementias

No parameters enforced

Score: 0.07, Star Rating: 2

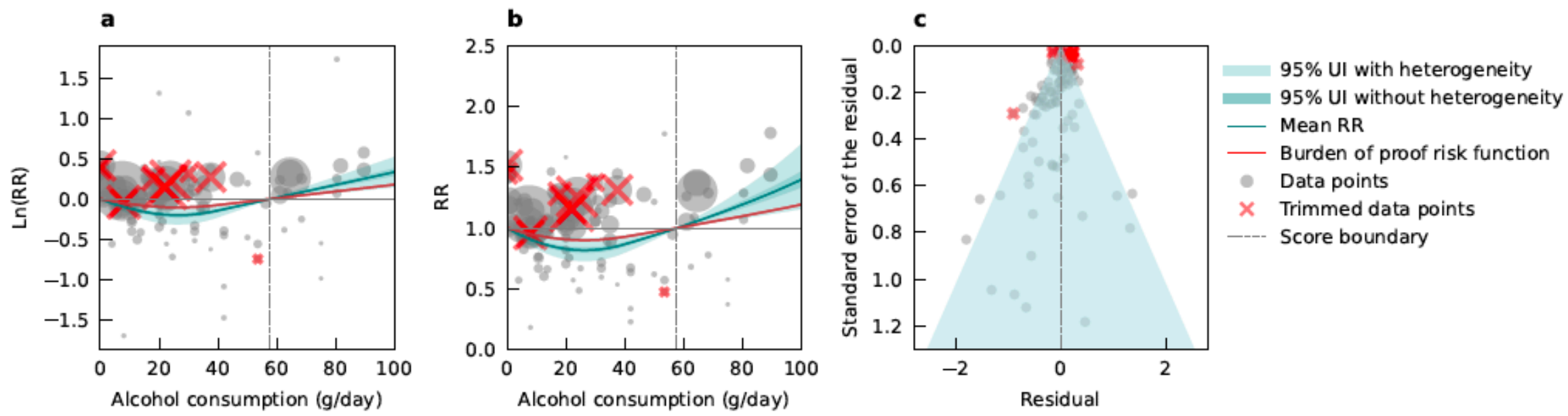

Right linear Tail enforced

Score: 0.09, Star Rating: 2

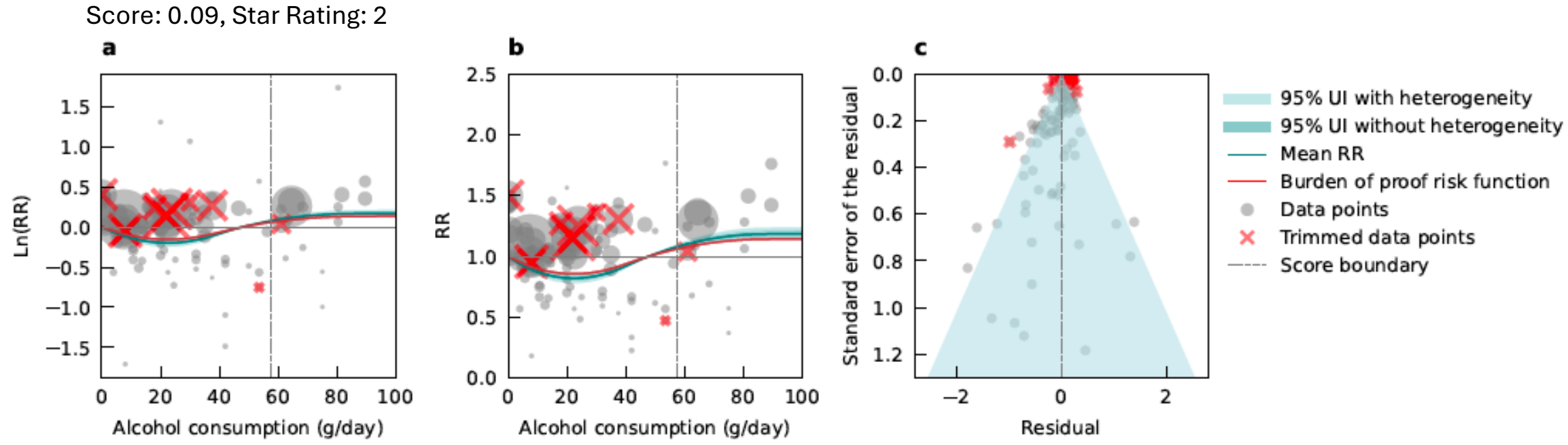

Left linear Tail enforced

Score: 0.04, Star Rating: 2

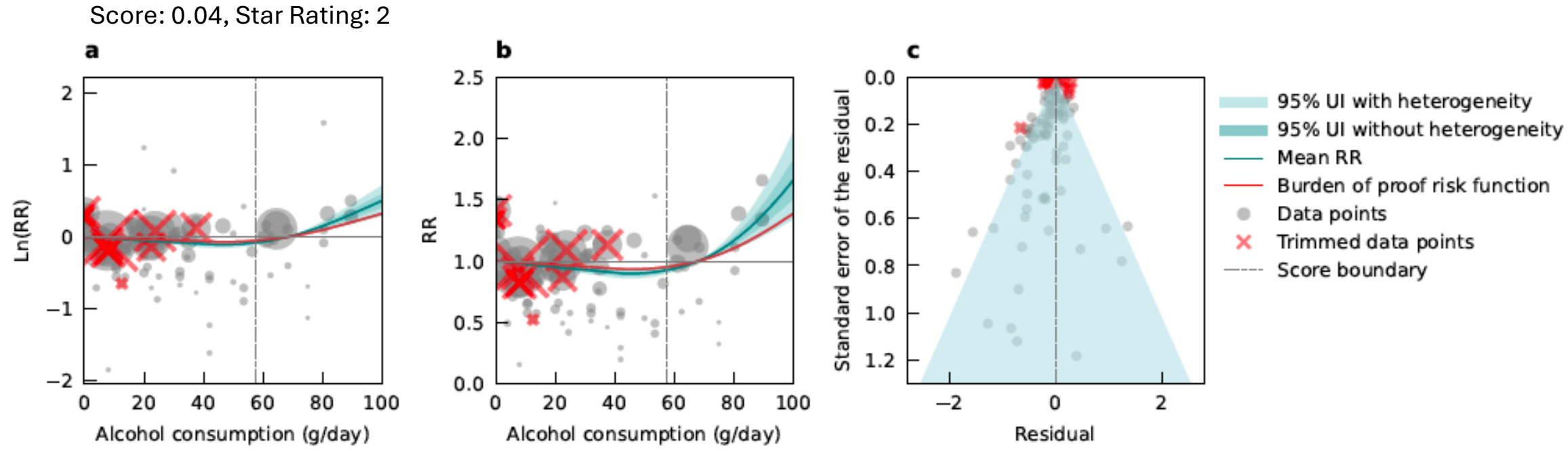

Left and right linear tails enforced

Score: 0.02, Star Rating: 2

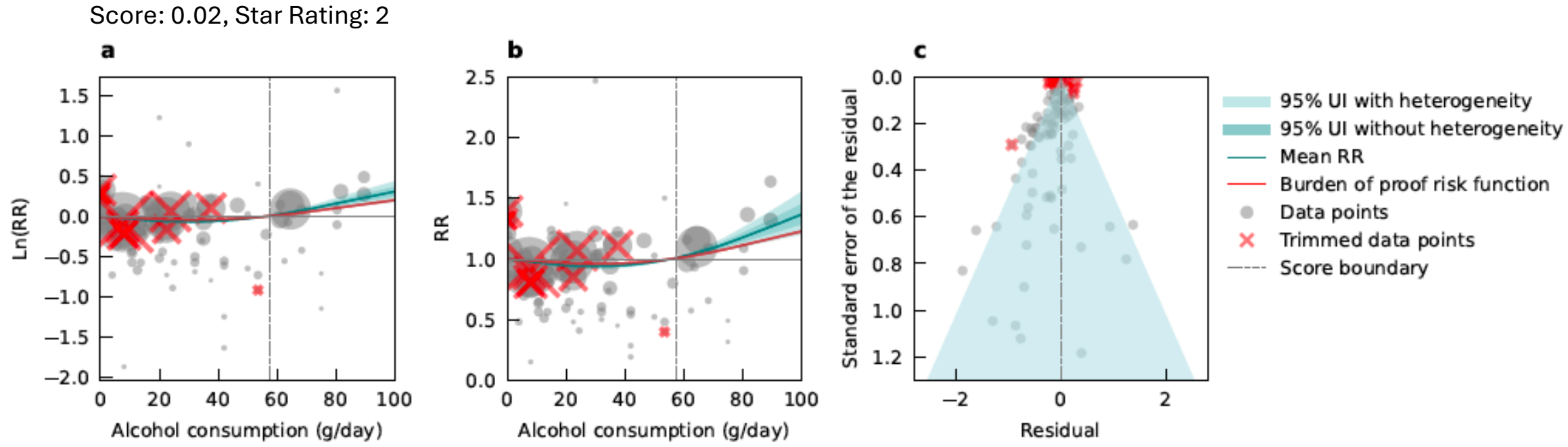

Figure S2.06. Type 2 diabetes

No parameters enforced

Score: 0.05, Star Rating: 2

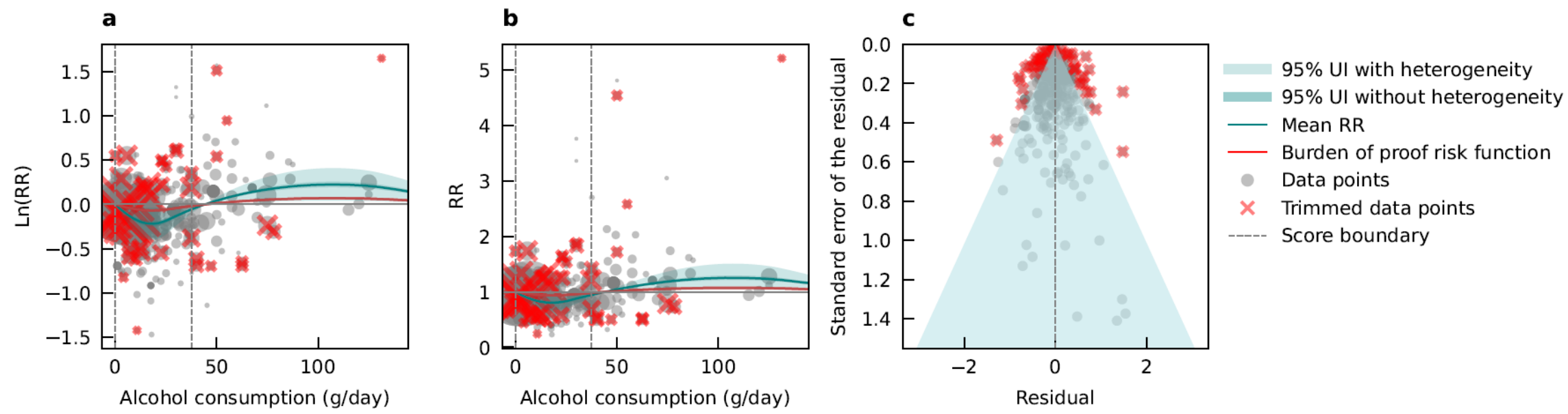

Right linear tail enforced

Score: 0.05, Star Rating: 2

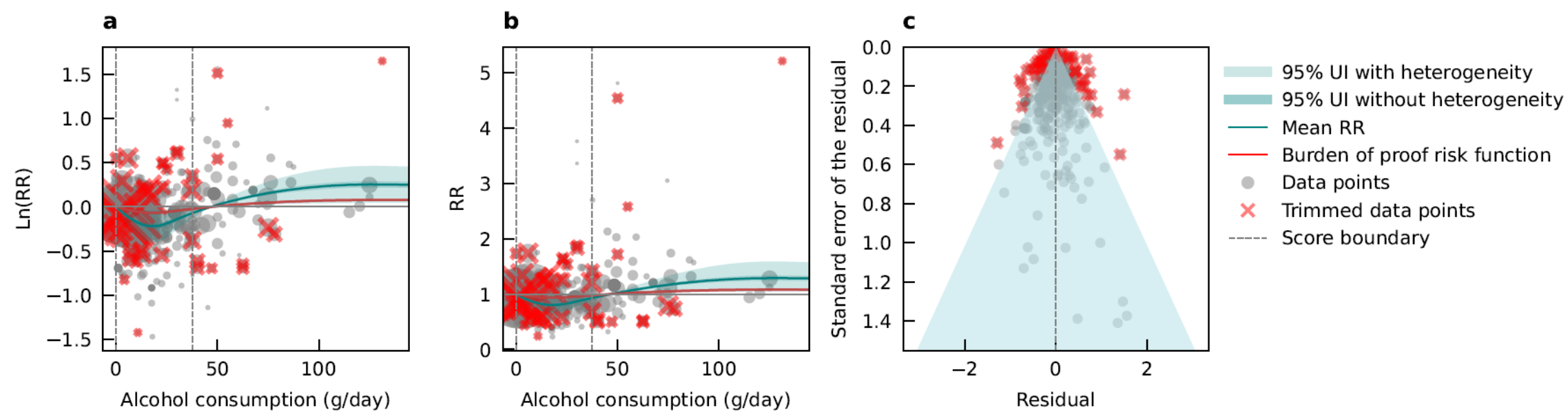

Left linear tail enforced

Score: -0.14, Star Rating: 1

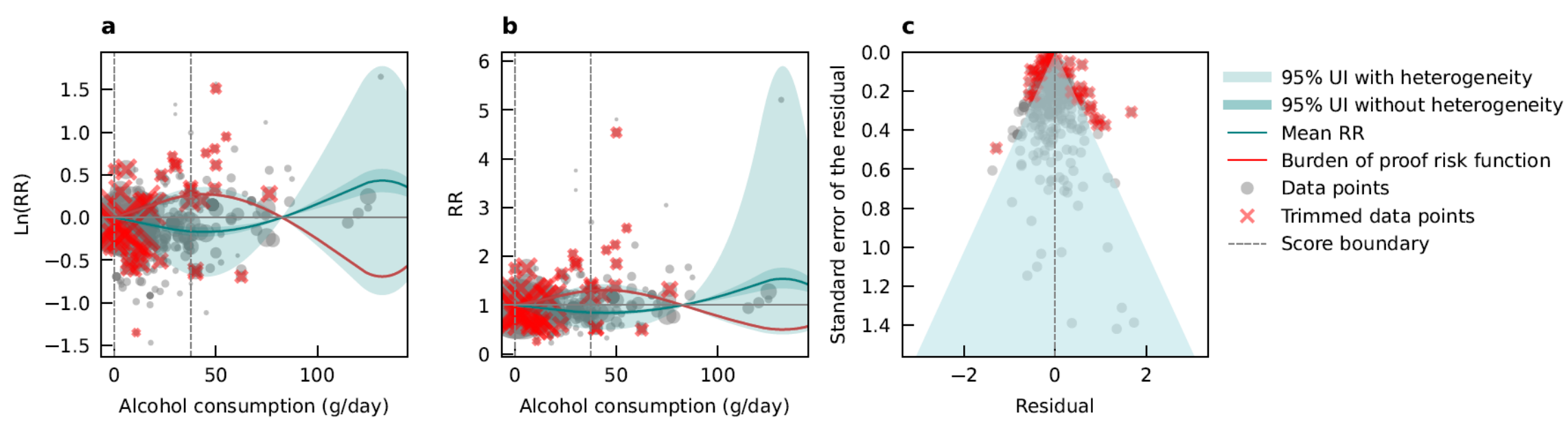

Left and right linear tails enforced

Score: -0.18, Star Rating: 1

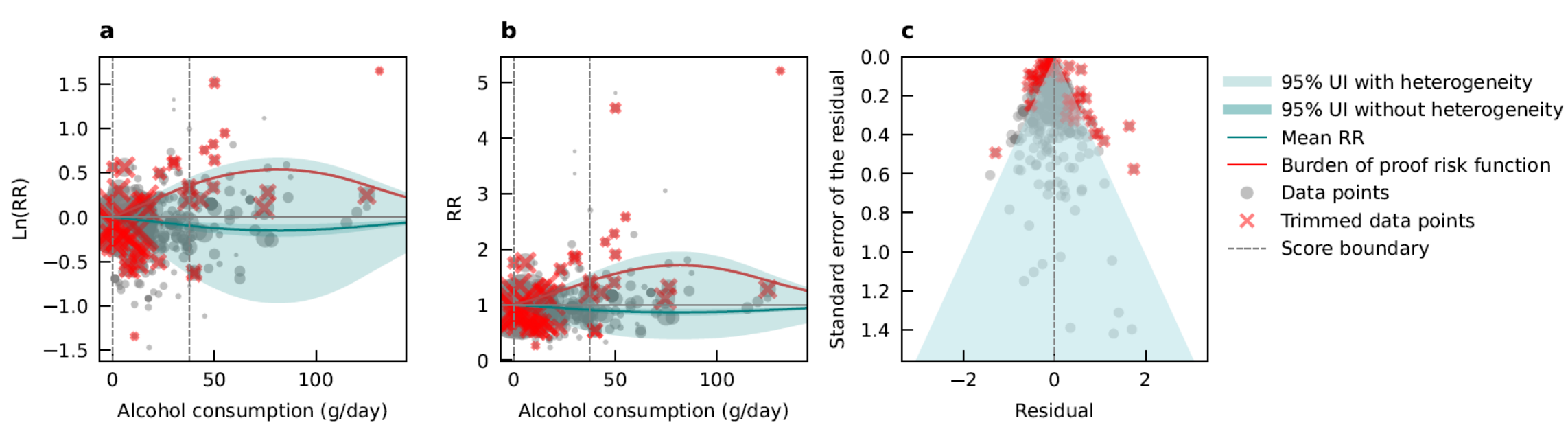

Figure S2.07. Esophageal cancer

No parameters enforced

Score: 0.17, Star Rating: 3

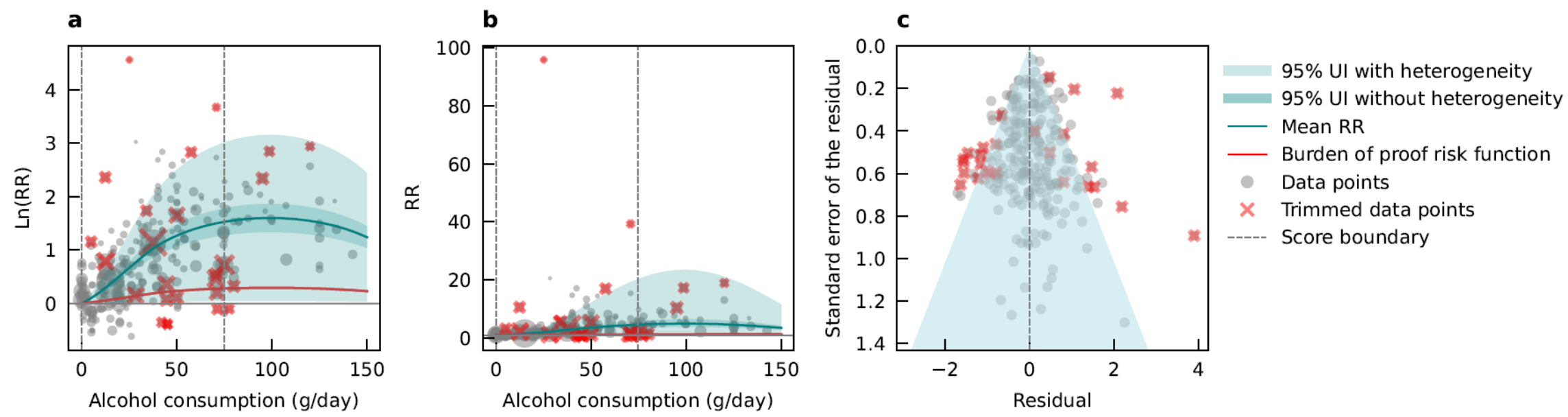

Right linear tail enforced

Score: 0.14, Star Rating: 3

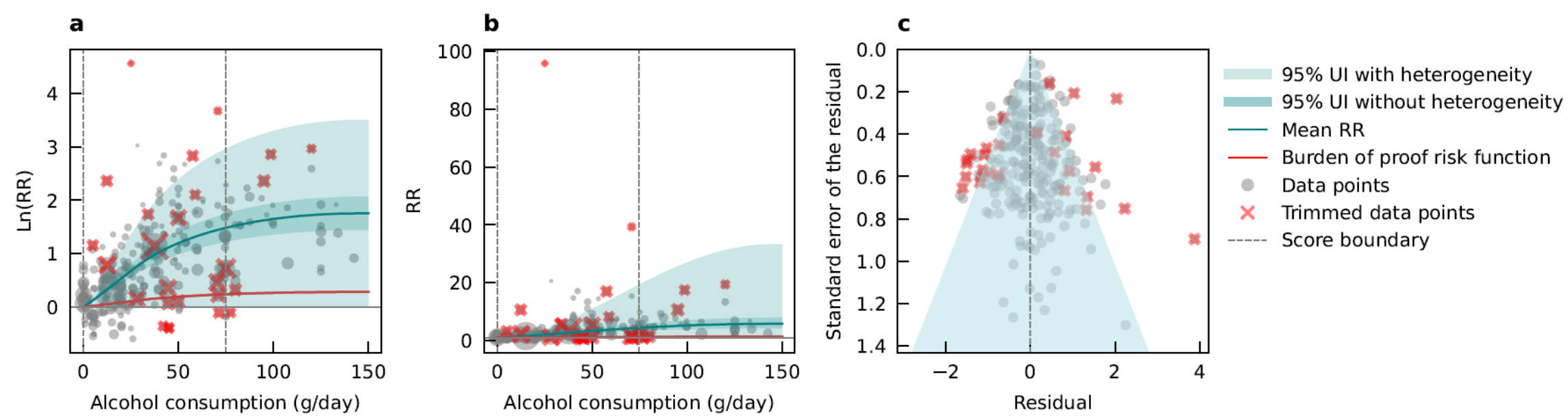

Monotonicity increasing enforced

Score: 0.14, Star Rating: 2

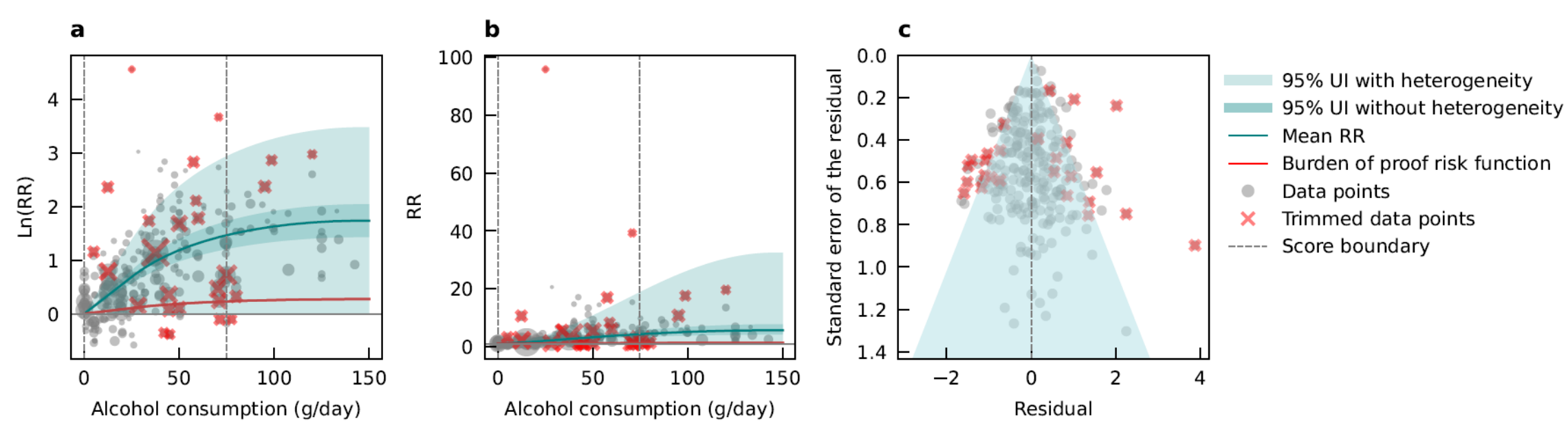

Right linear tail and monotonicity increasing enforced

Score: 0.14, Star Rating: 2

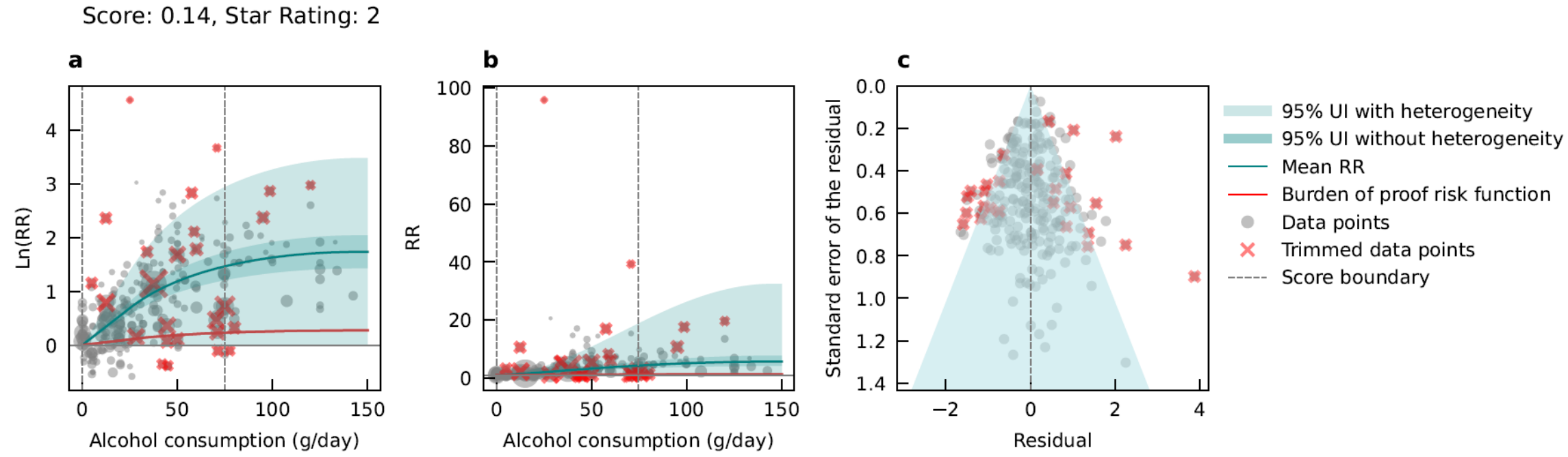

Figure S2.08. Larynx cancer

No parameters enforced

Score: 0.43, Star Rating: 4

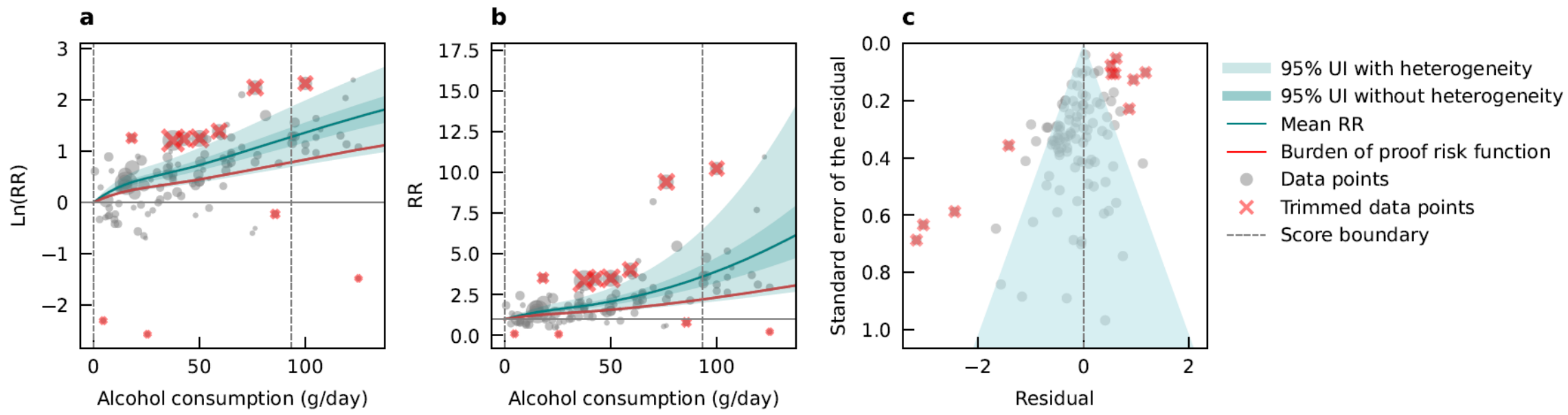

Right linear tail enforced

Score: 0.36, Star Rating: 3

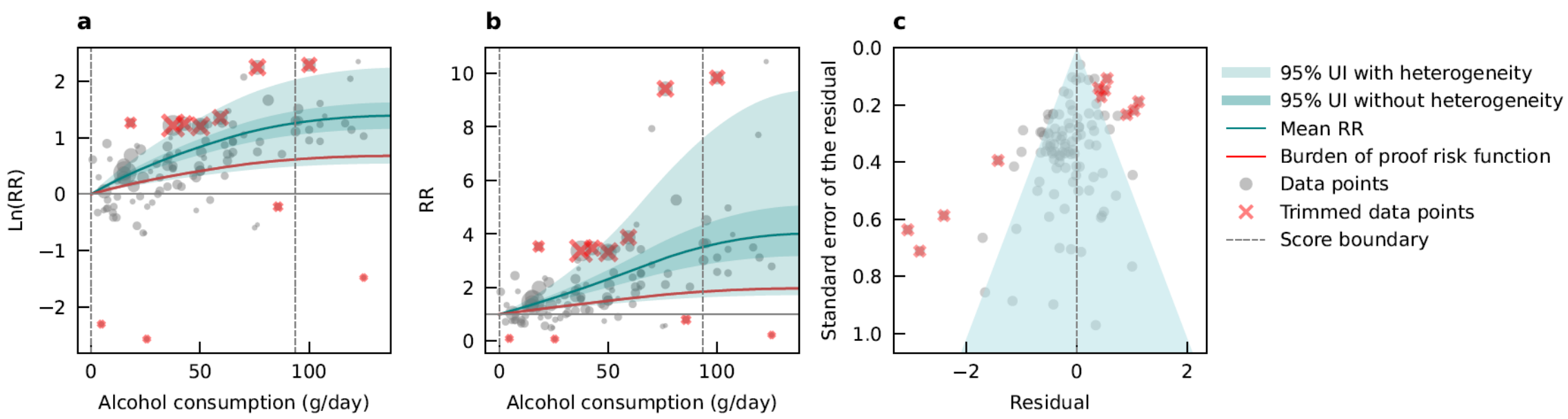

Monotonicity increasing enforced

Score: 0.43, Star Rating: 4

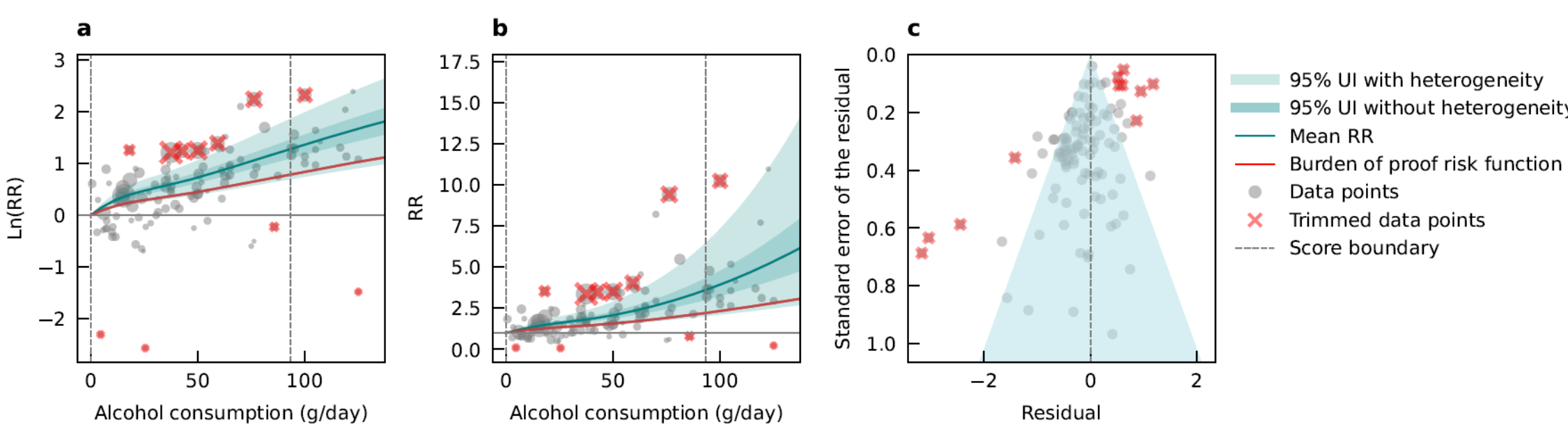

Right linear tail and monotonicity increasing enforced

Score: 0.36, Star Rating: 3

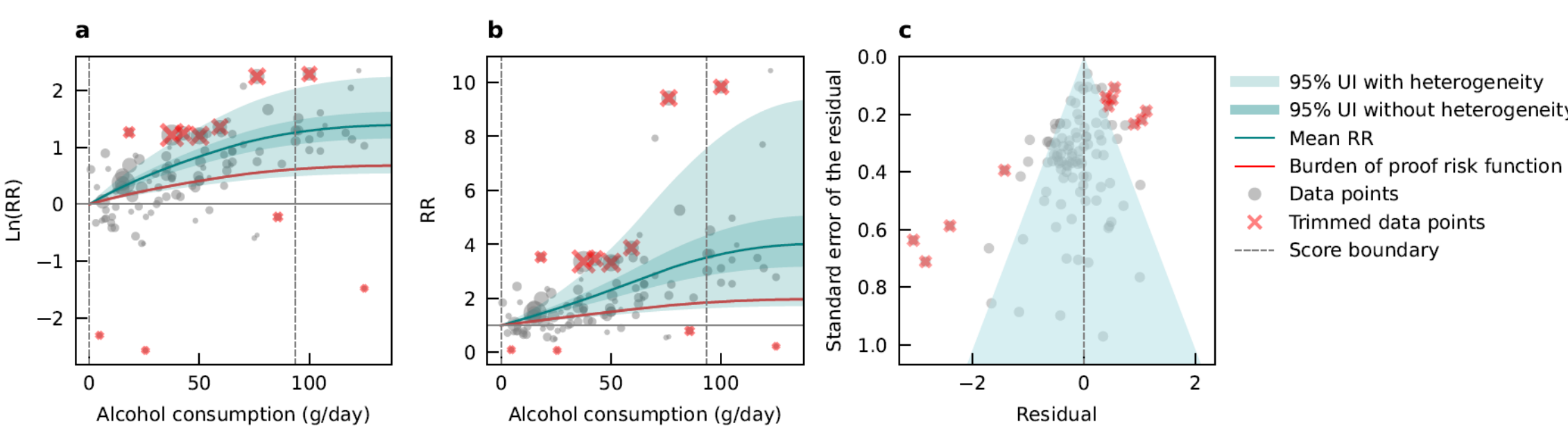

Figure S2.09. Mouth cancer

No parameters enforced

Score: 0.11, Star Rating: 2

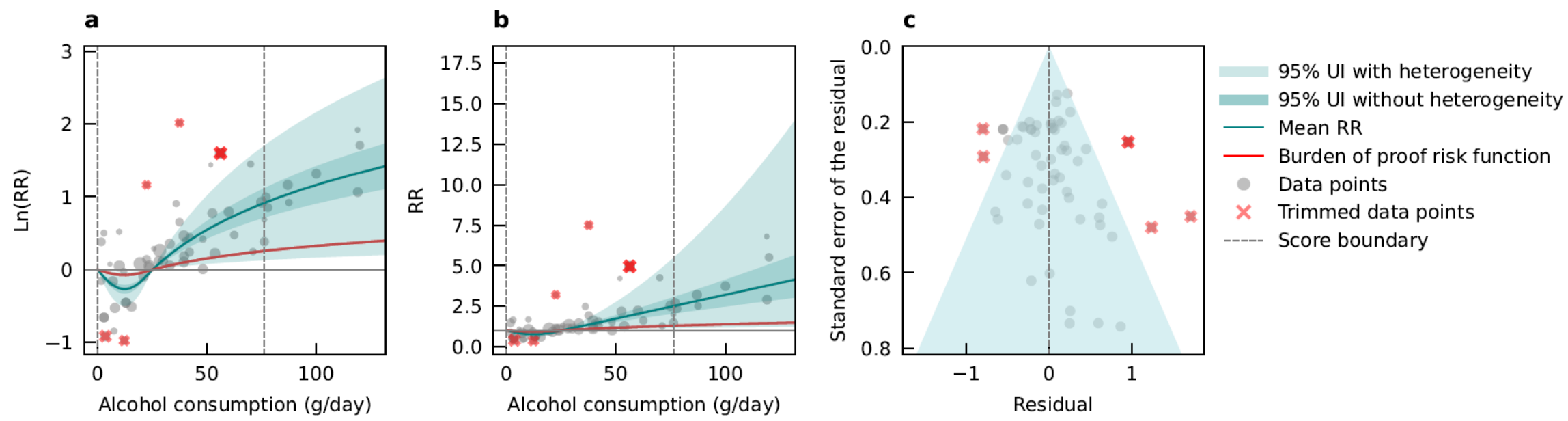

Right linear tail enforced

Score: 0.13, Star Rating: 2

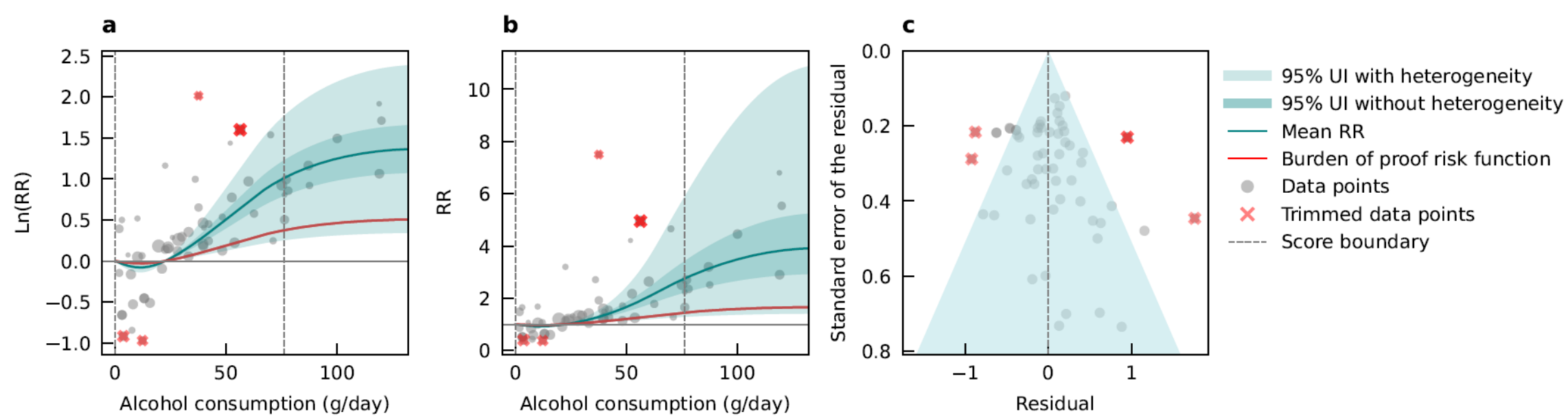

Monotonicity increasing enforced

Score: 0.18, Star Rating: 3

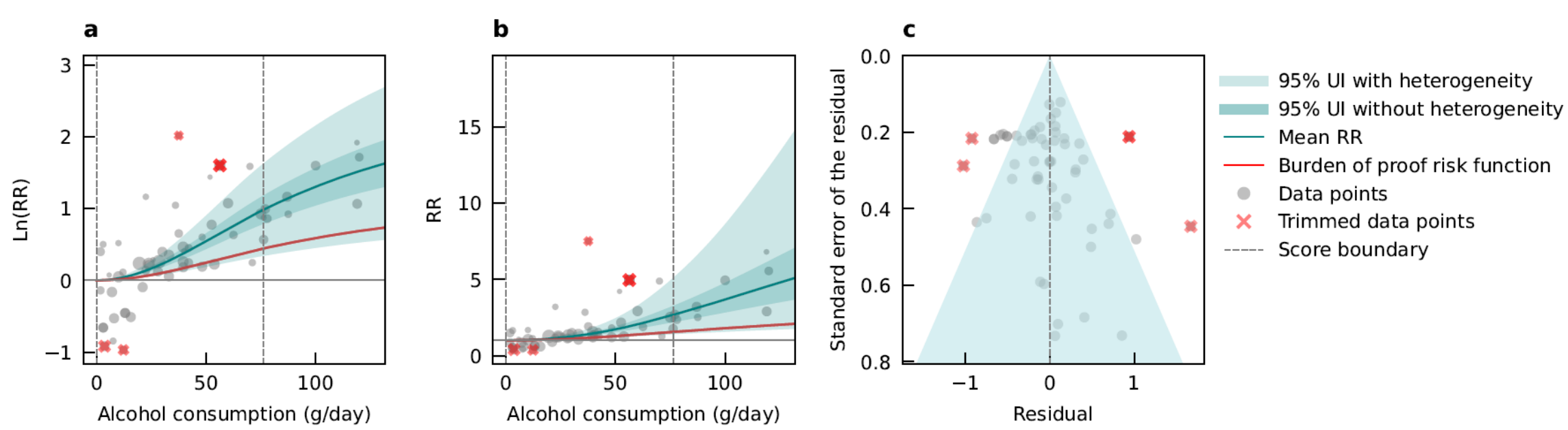

Right linear tail and monotonicity increasing enforced

Score: 0.18, Star Rating: 3

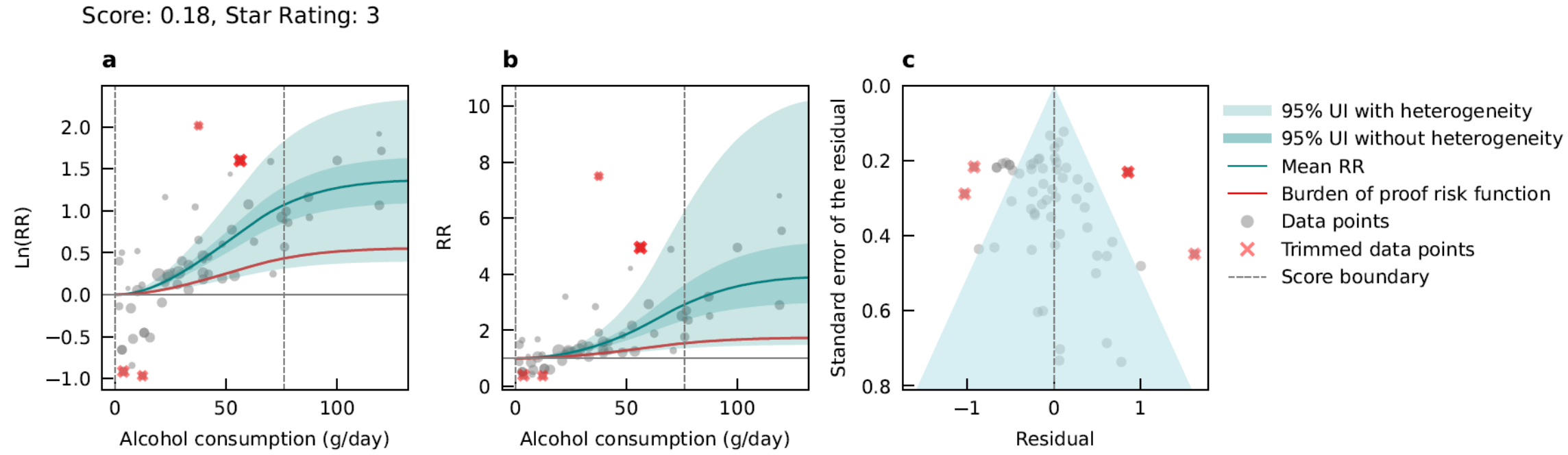

Figure S2.10. Other pharynx cancer

No parameters enforced

Score: 0.63, Star Rating: 5

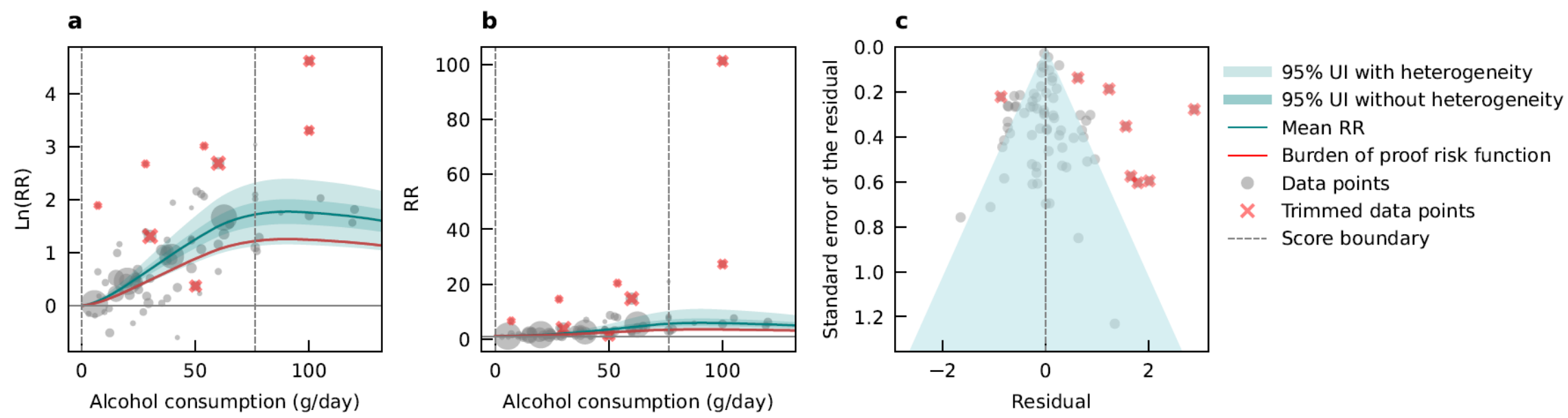

Right linear tail enforced

Score: 0.72, Star Rating: 5

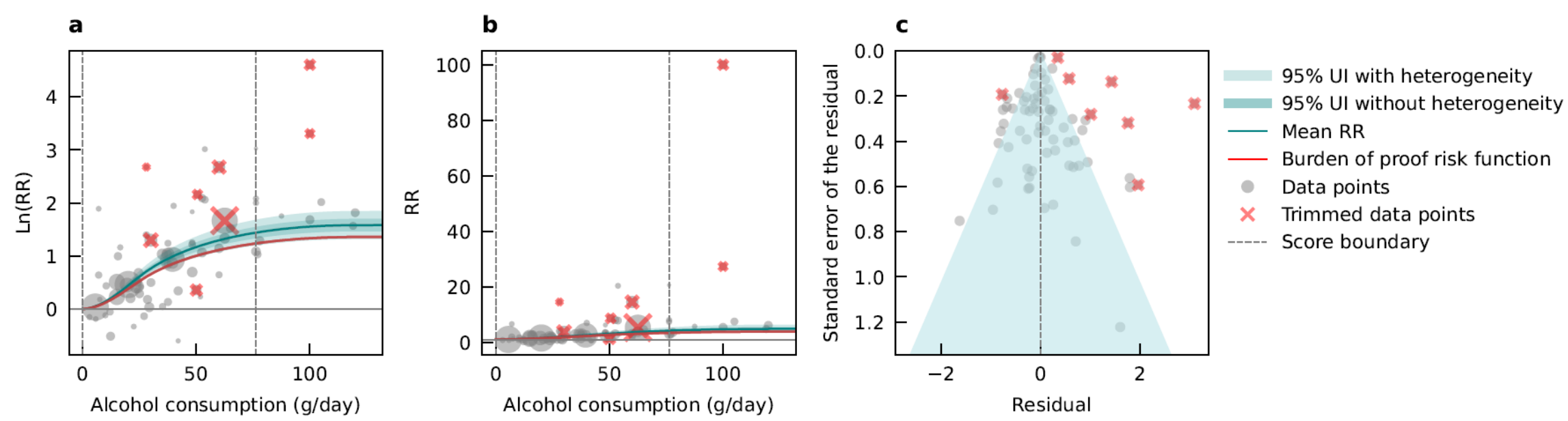

Monotonicity increasing enforced

Score: 0.61, Star Rating: 4

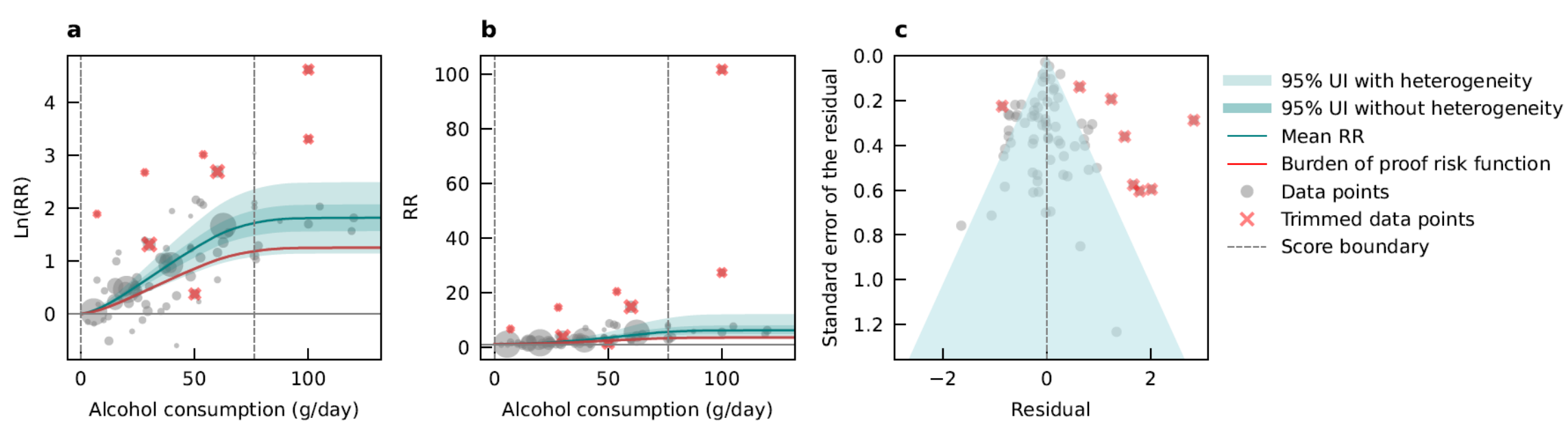

Right linear tail and monotonicity increasing enforced

Score: 0.72, Star Rating: 5

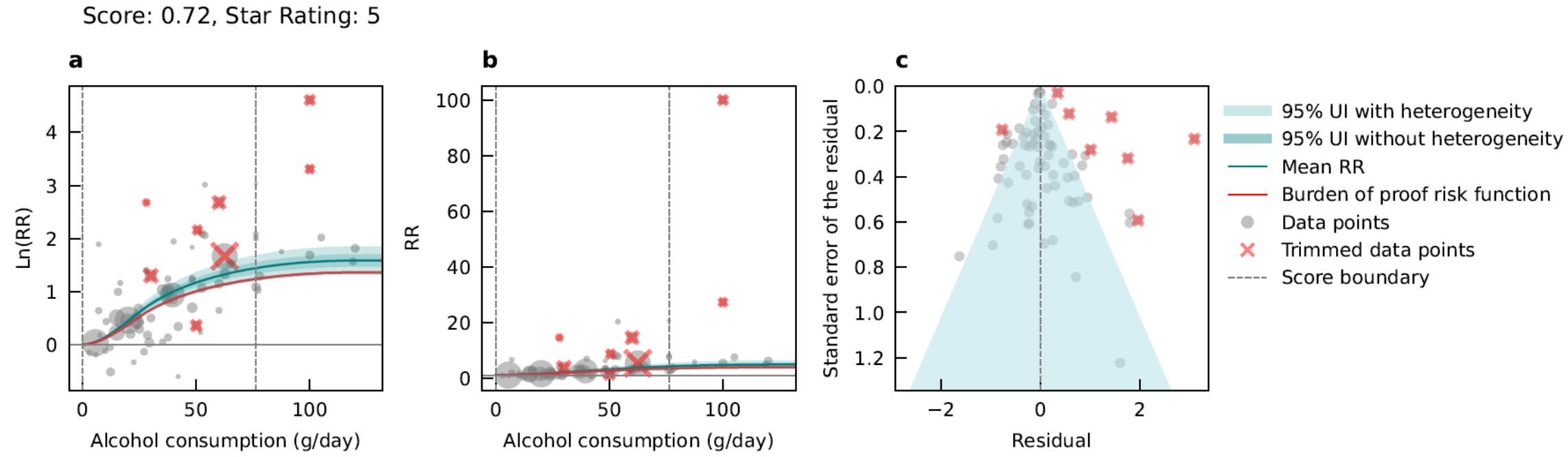

Figure S2.11. Ischemic heart disease

No parameters enforced

Score: 0.07, Star Rating: 2

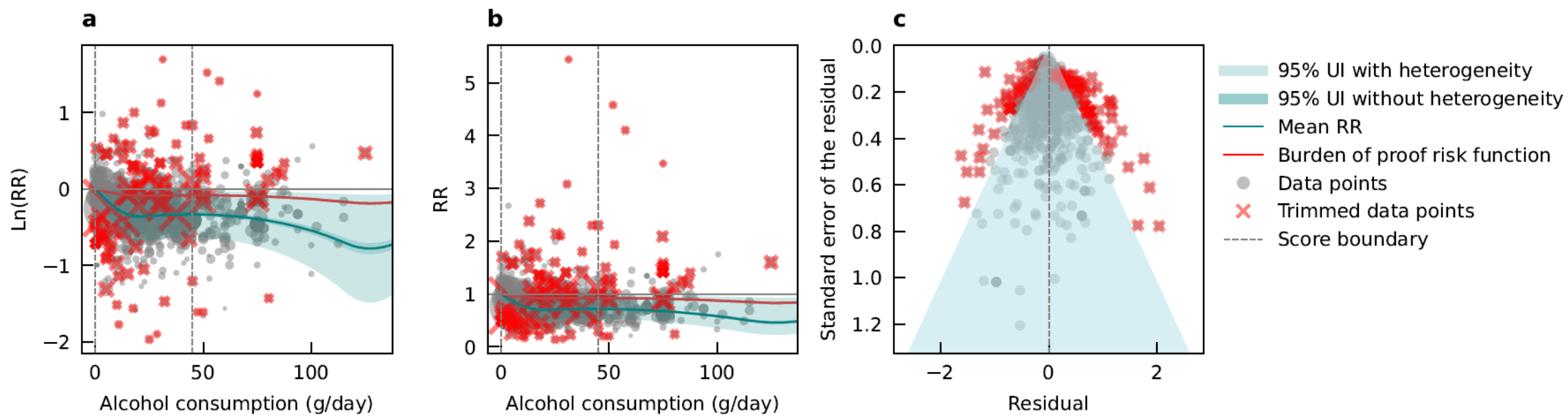

Right linear tail enforced

Score: 0.06, Star Rating: 2

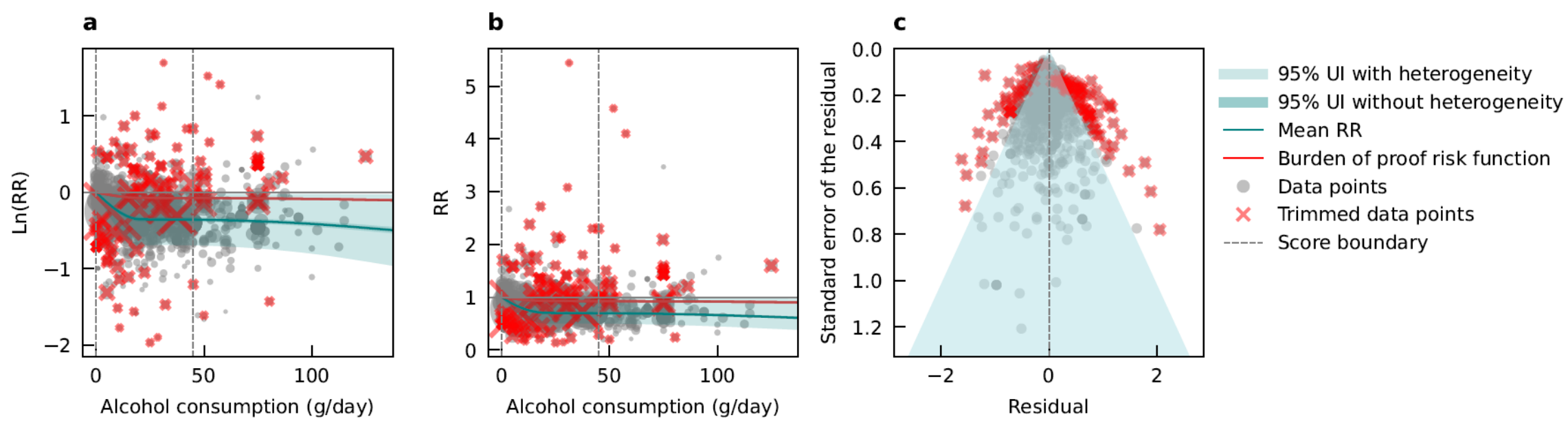

Left linear tail enforced

Score: -0.07, Star Rating: 1

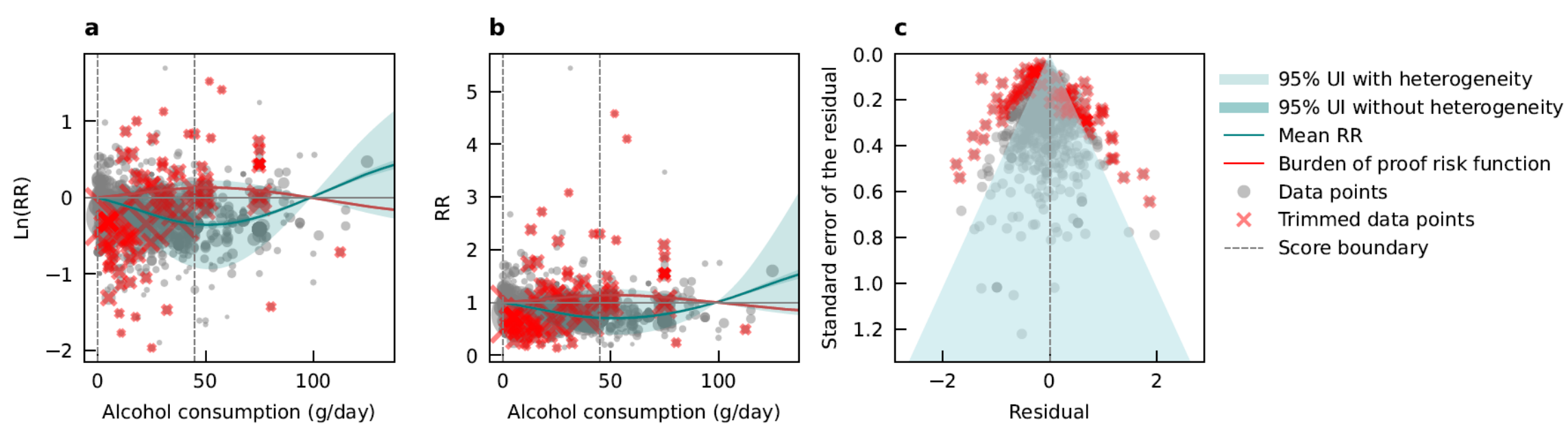

Left and right linear tails enforced

Score: -0.1, Star Rating: 1

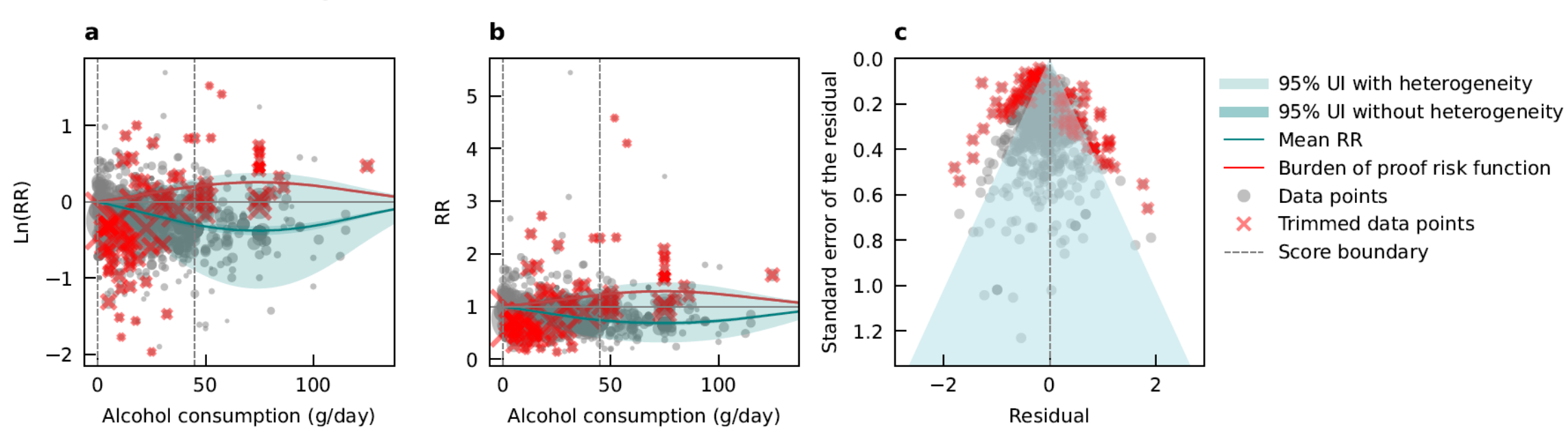

Figure S2.12. Liver cancer

No parameters enforced

Score: 0.05, Star Rating: 2

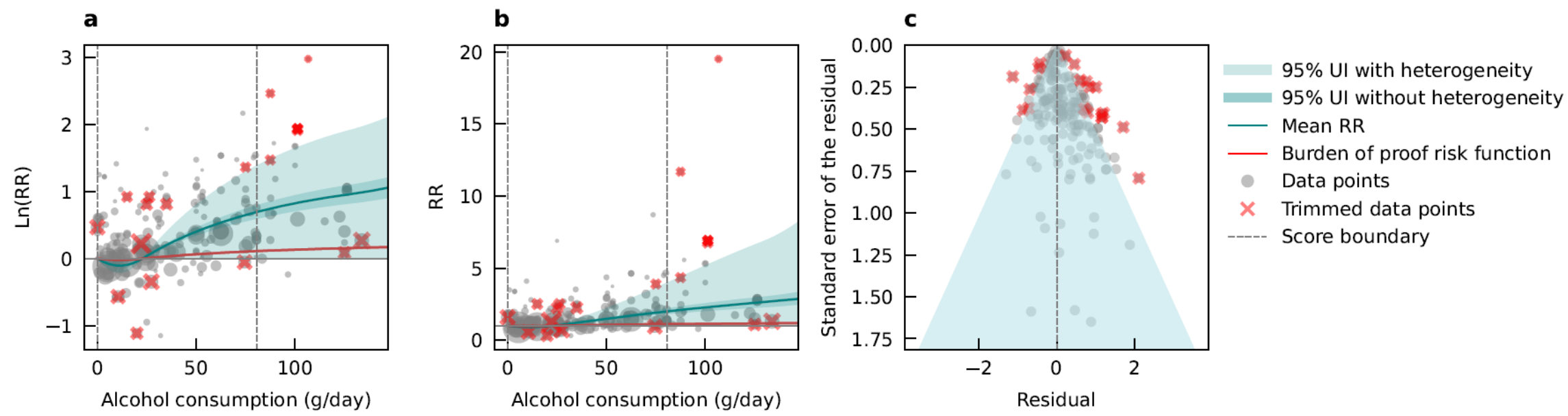

Right linear tail enforced

Score: 0.04, Star Rating: 2

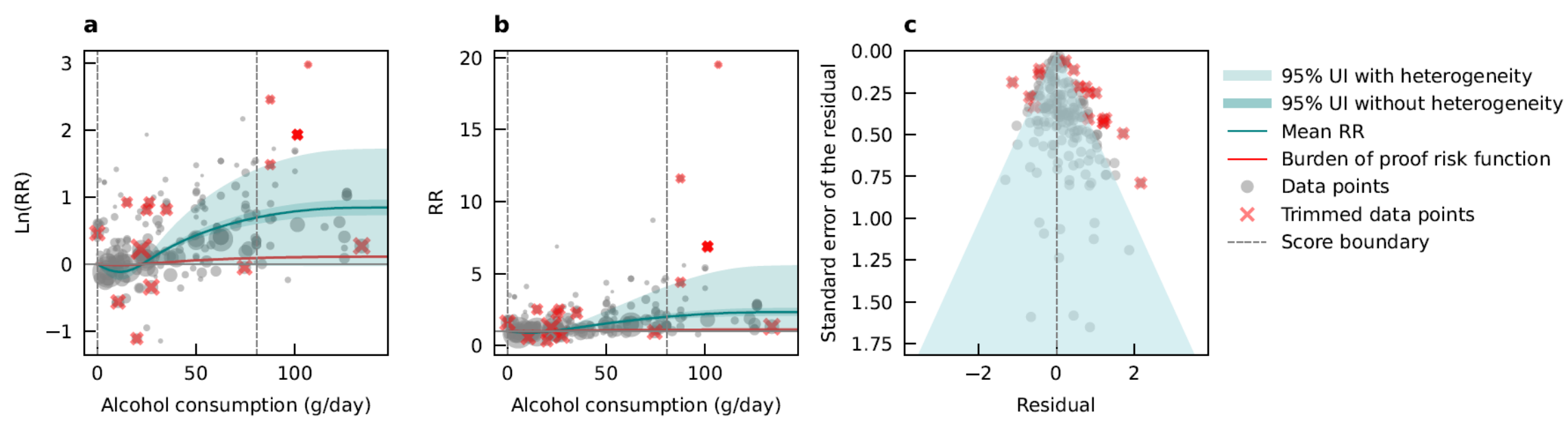

Monotonicity increasing enforced

Score: 0.05, Star Rating: 2

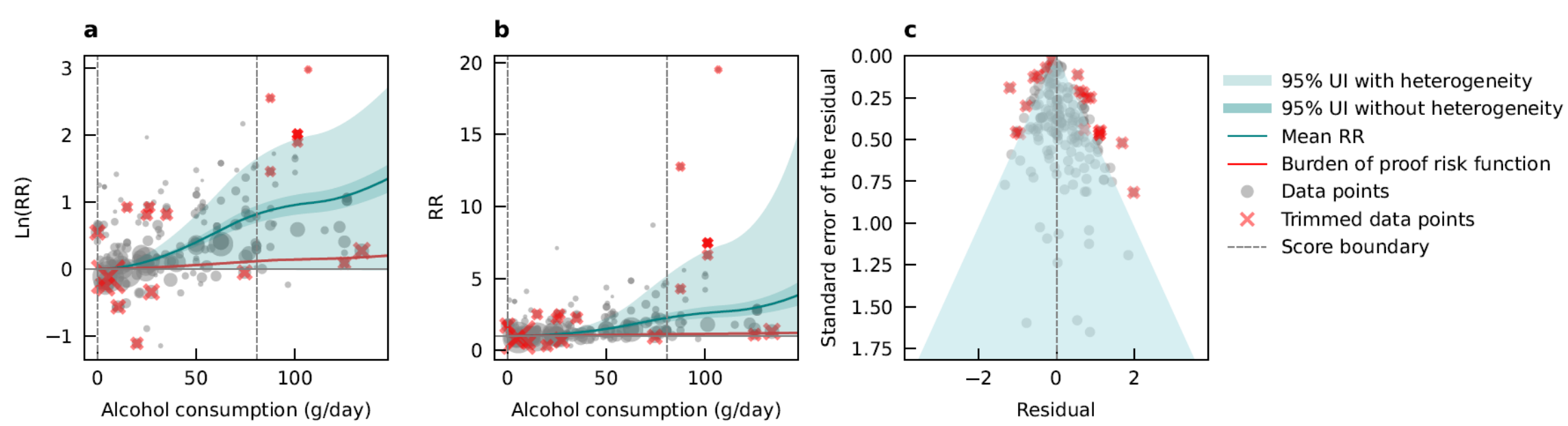

Right linear tail and monotonicity increasing enforced

Score: 0.03, Star Rating: 2

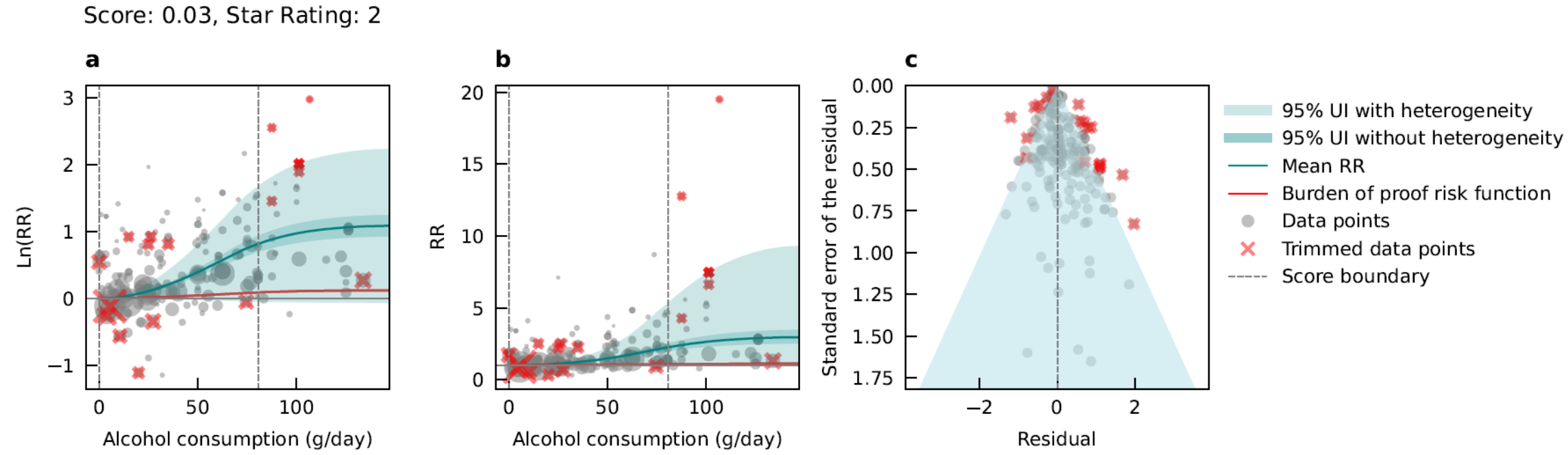

Figure S2.13. Lower respiratory infections

No parameters enforced

Score: 0.03, Star Rating: 2

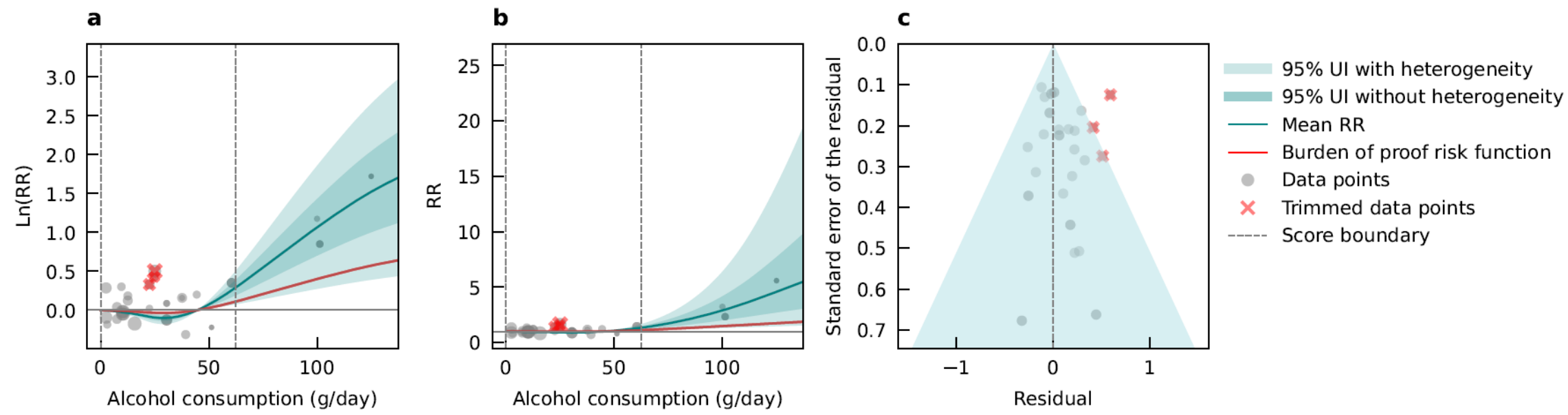

Right linear tail enforced

Score: 0.01, Star Rating: 2

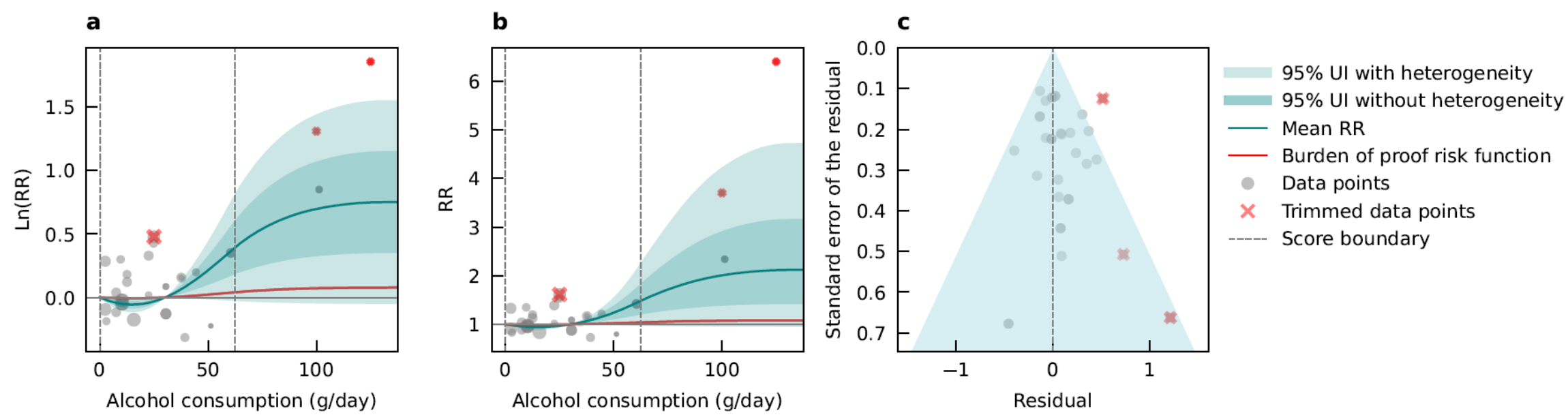

Monotonicity increasing enforced

Score: 0.02, Star Rating: 2

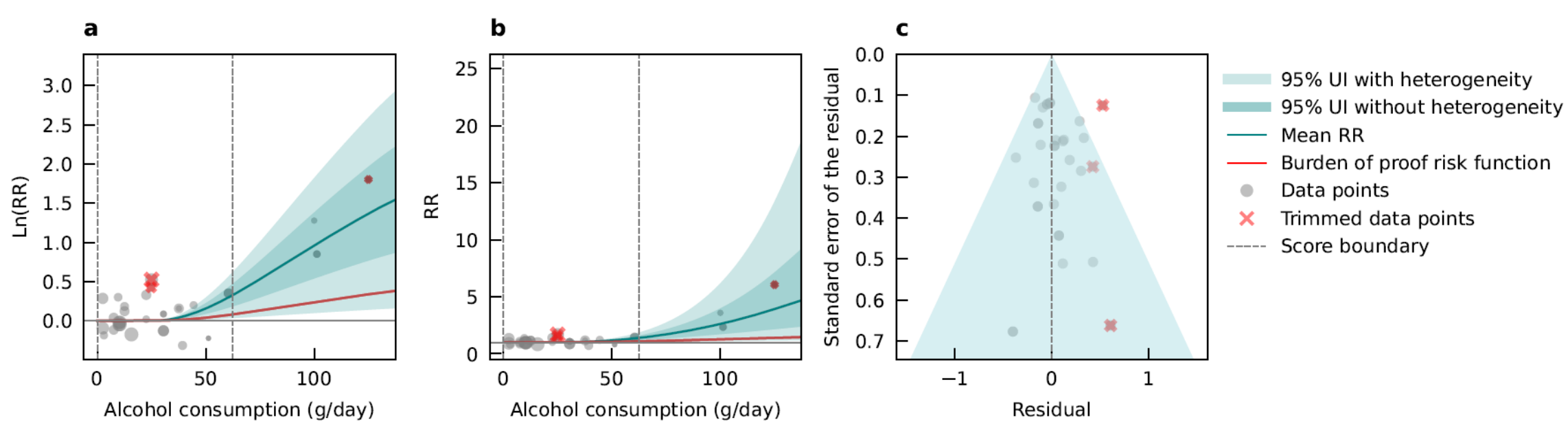

Right linear tail and monotonicity increasing enforced

Score: 0.0, Star Rating: 2

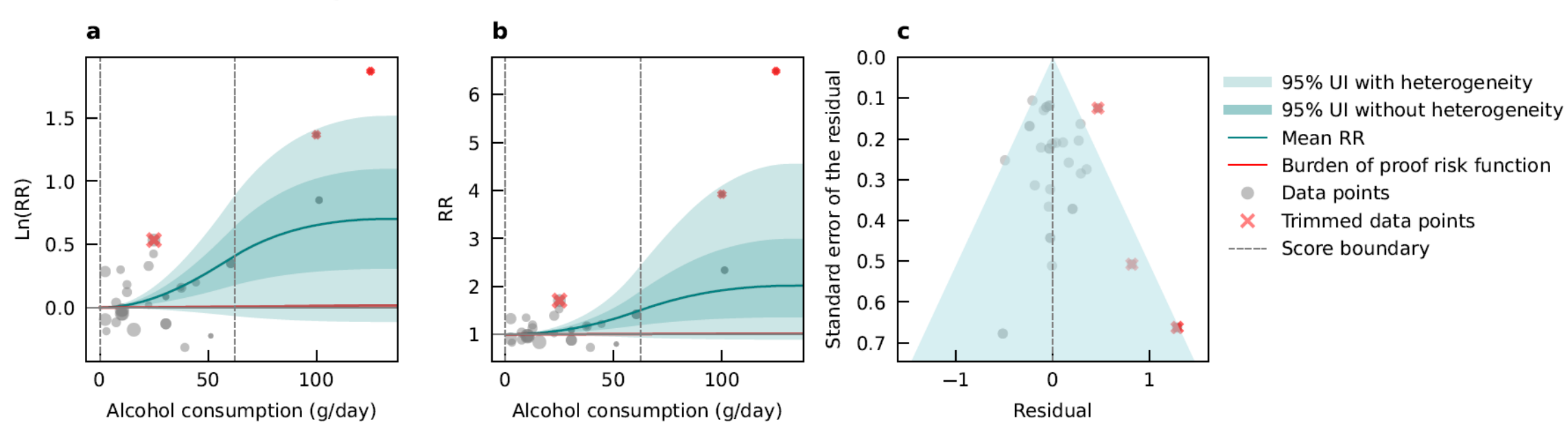

Figure S2.14. Pancreatitis

No parameters enforced

Score: 0.05, Star Rating: 2

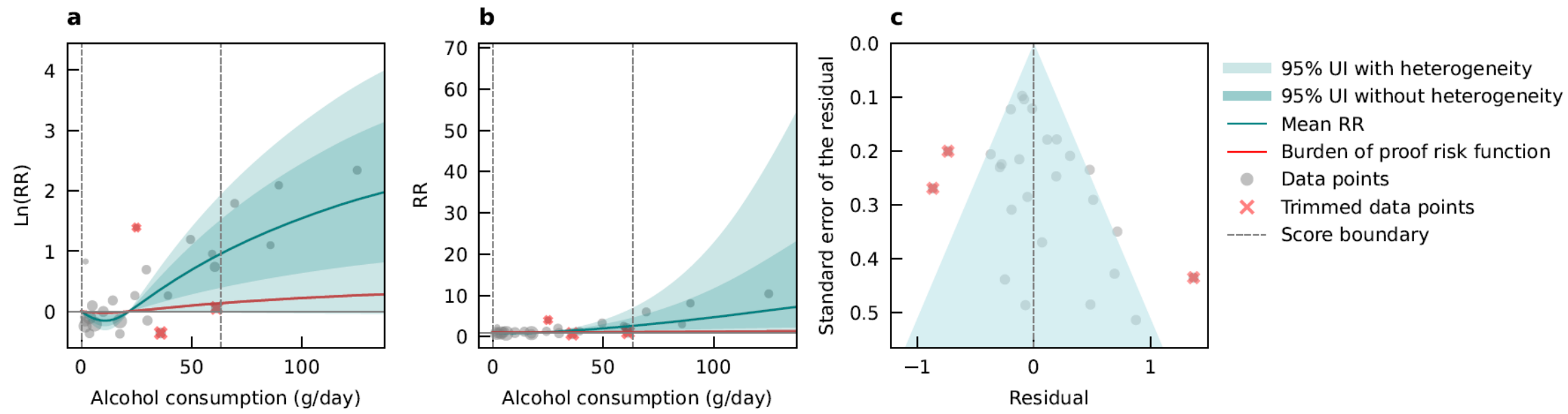

Right linear tail enforced

Score: 0.0, Star Rating: 2

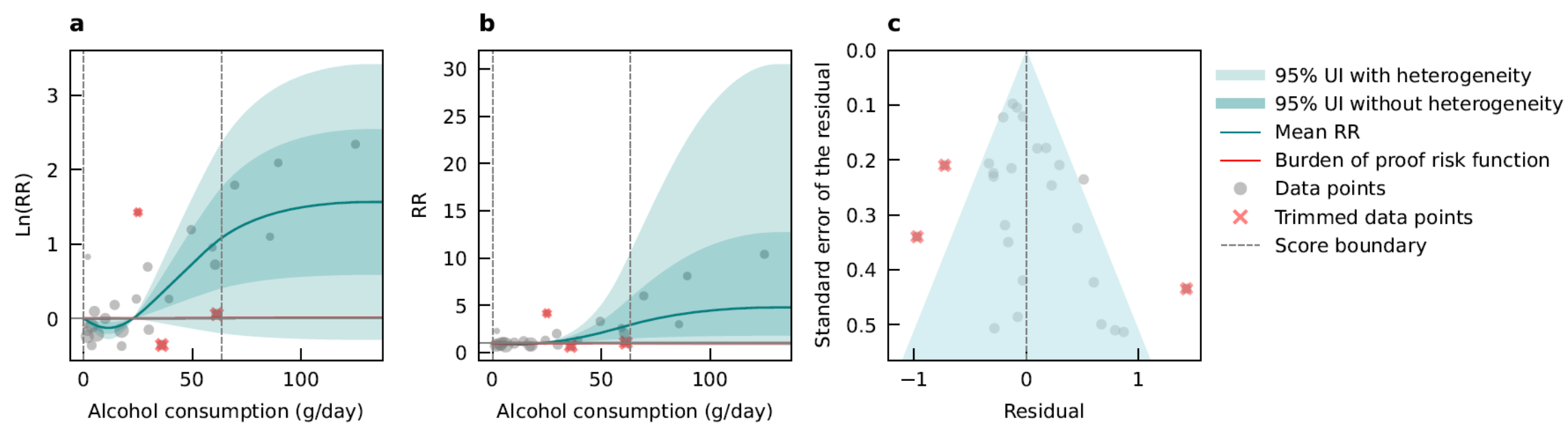

Monotonicity increasing enforced

Score: 0.16, Star Rating: 3

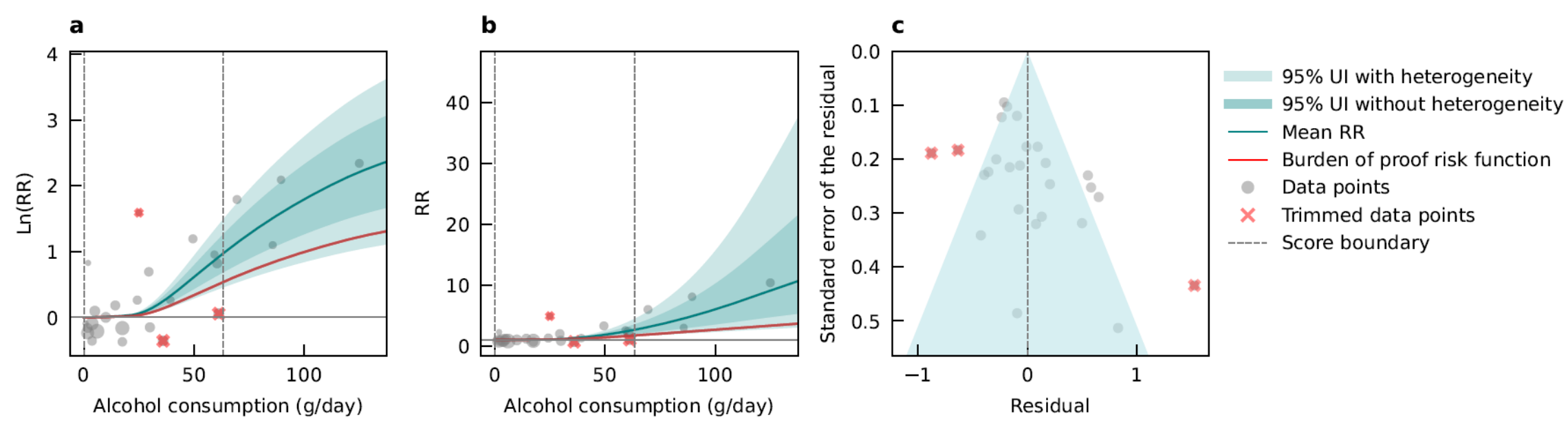

Right linear tail and monotonicity increasing enforced

Score: -0.11, Star Rating: 1

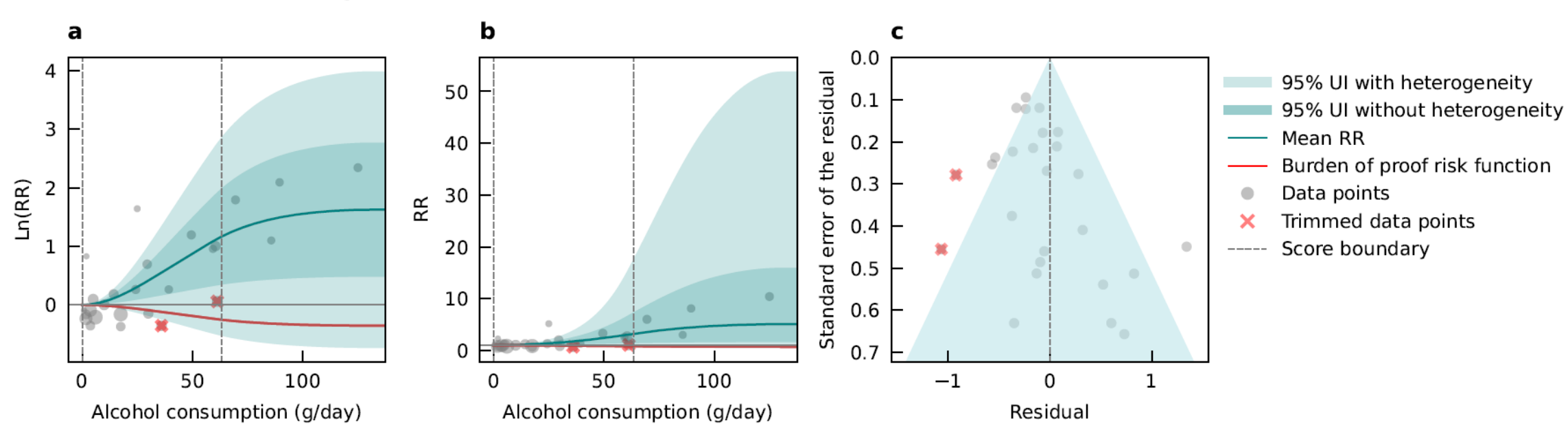

Figure S2.15. Pancreatic cancer

No parameters enforced

Score: 0.02, Star Rating: 2

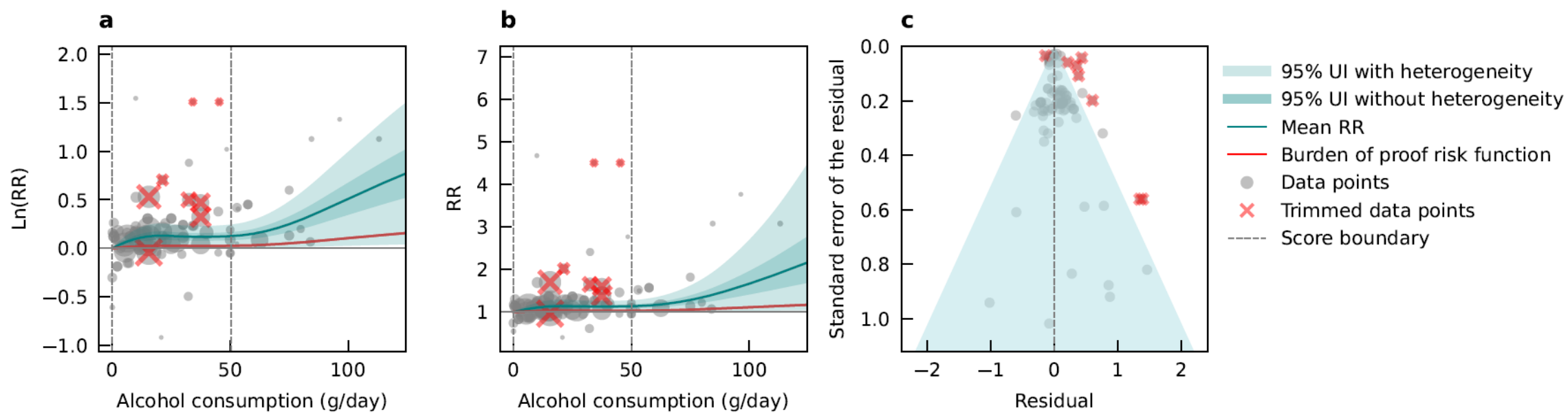

Right linear tail enforced

Score: 0.03, Star Rating: 2

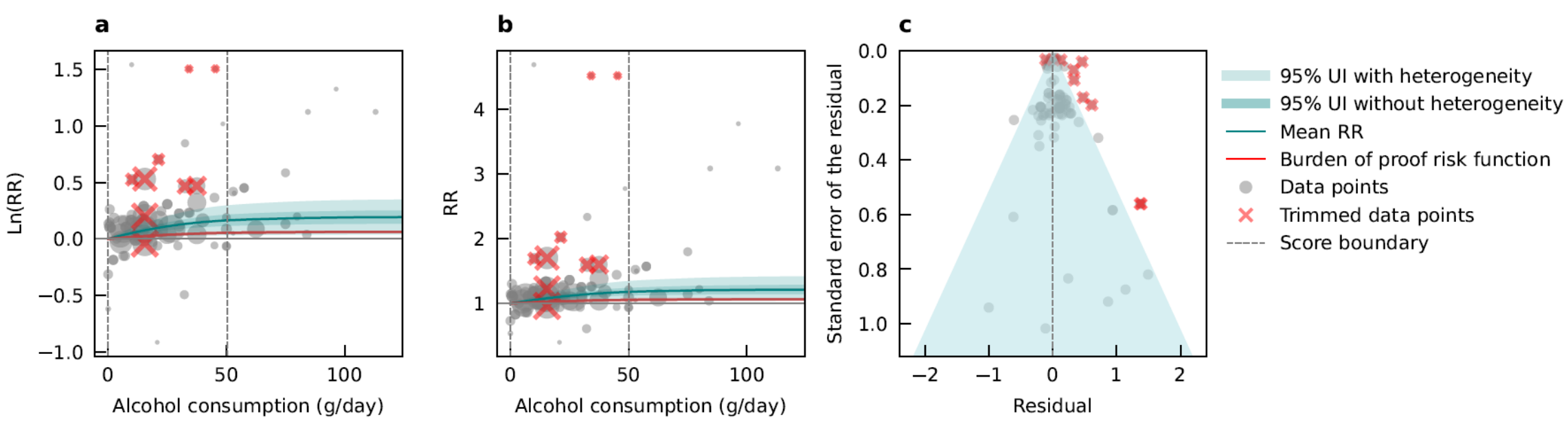

Monotonicity increasing enforced

Score: 0.01, Star Rating: 2

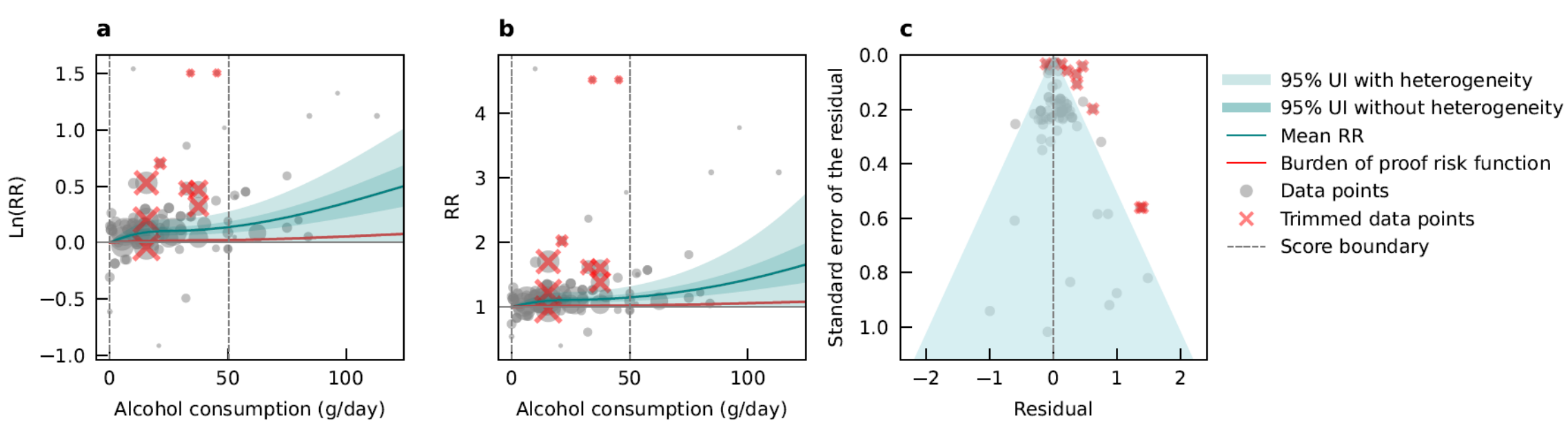

Right linear tail and monotonicity increasing enforced

Score: 0.03, Star Rating: 2

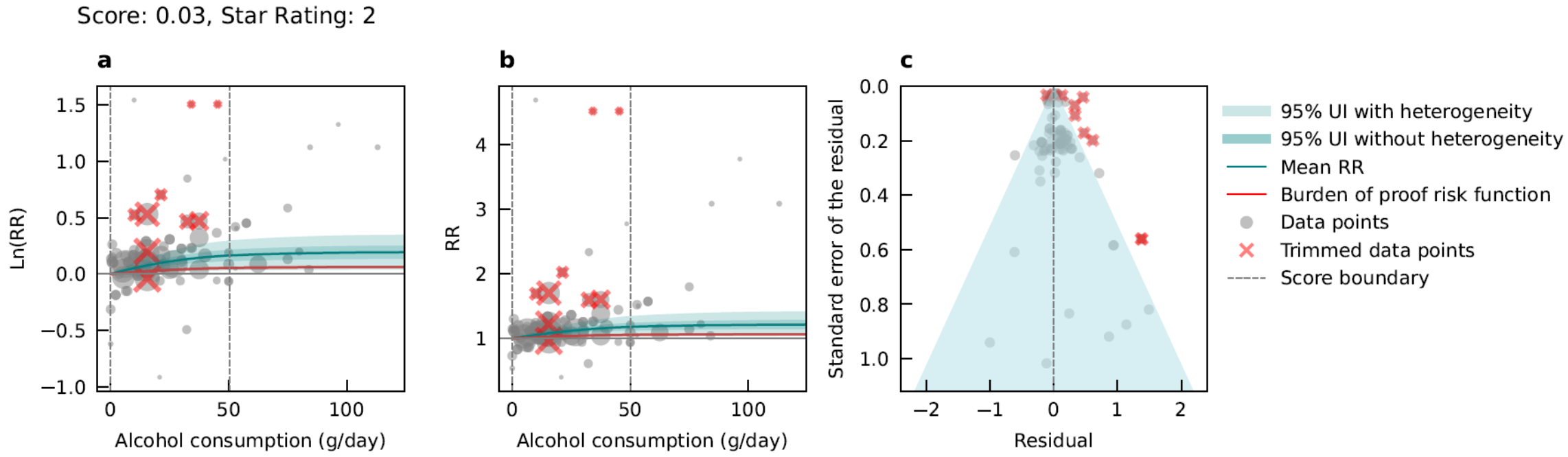

Figure S2.16. Prostate cancer

No parameters enforced

Score: 0.01, Star Rating: 2

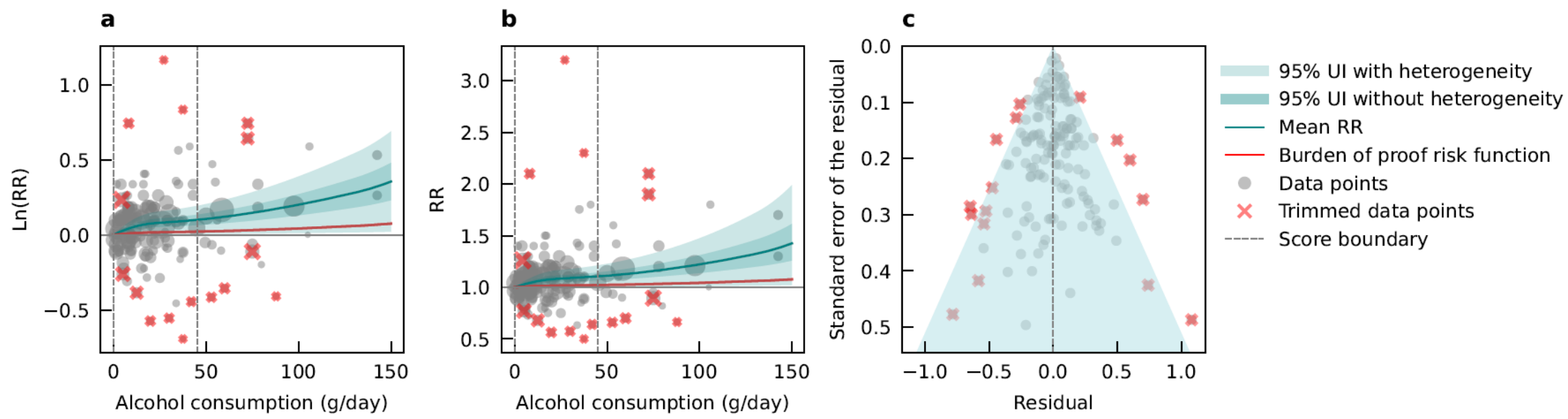

Right linear tail enforced

Score: 0.01, Star Rating: 2

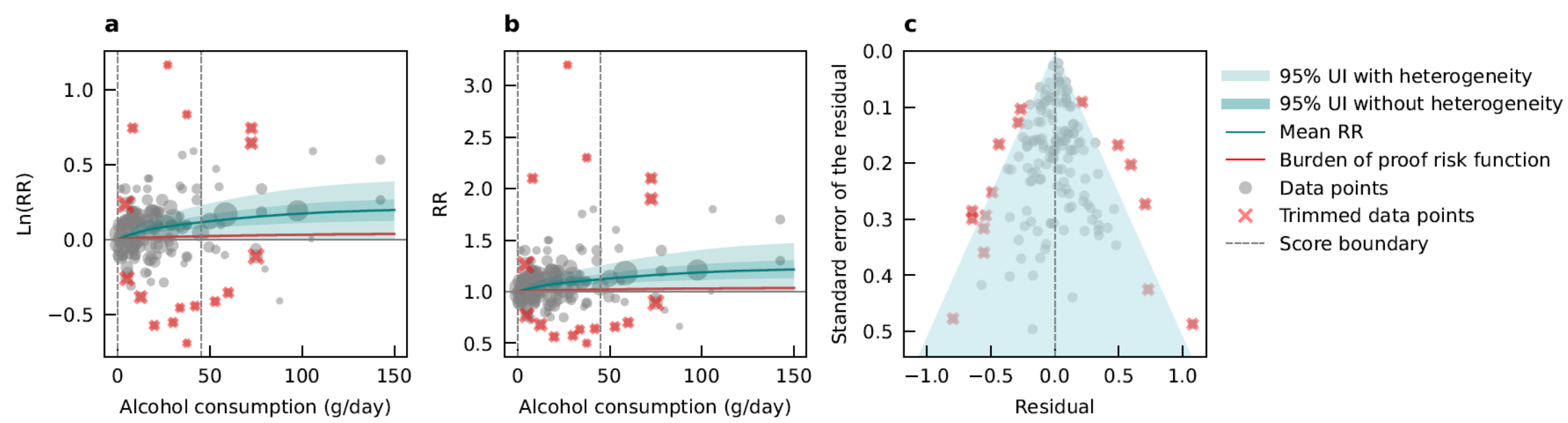

Monotonicity increasing enforced

Score: 0.01, Star Rating: 2

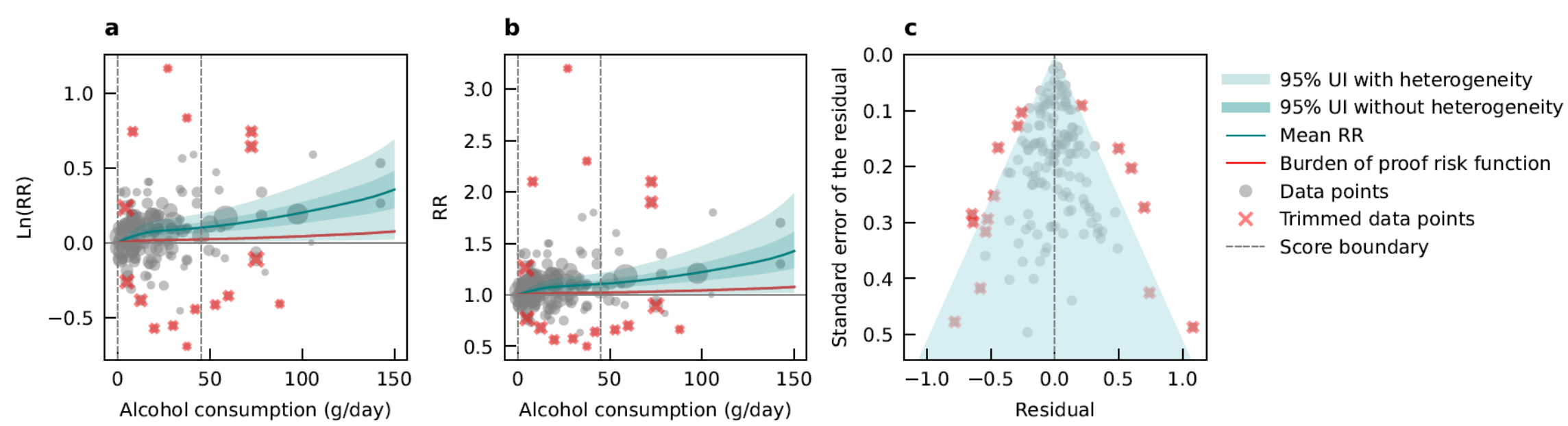

Right linear tail and monotonicity increasing enforced

Score: 0.01, Star Rating: 2

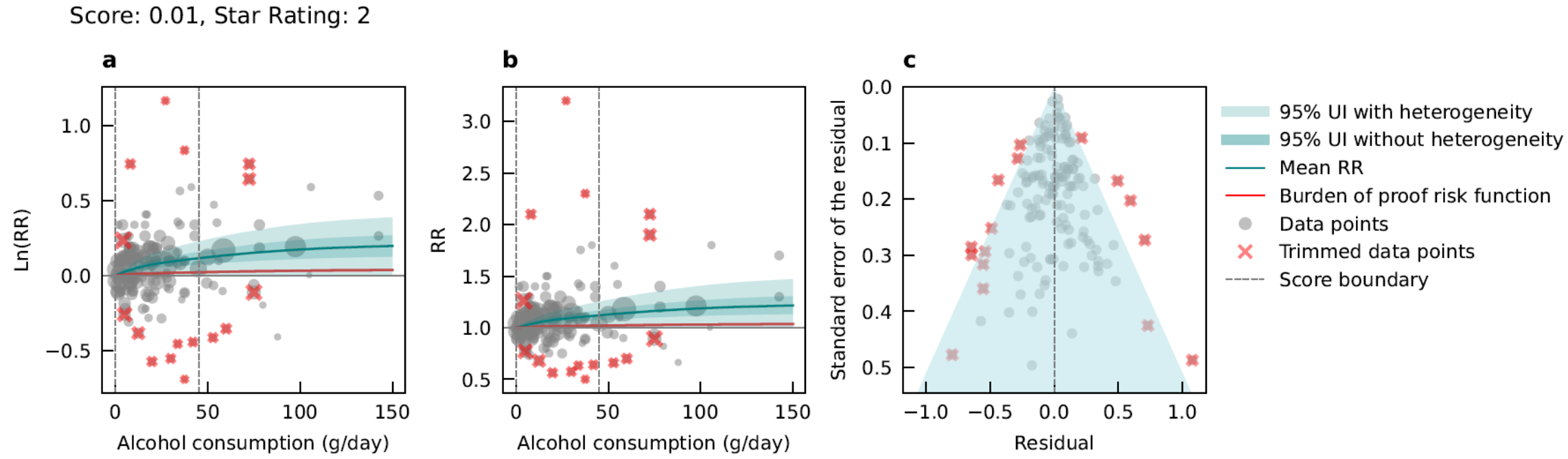

Figure S2.17. Stomach cancer

No parameters enforced

Score: 0.0, Star Rating: 2

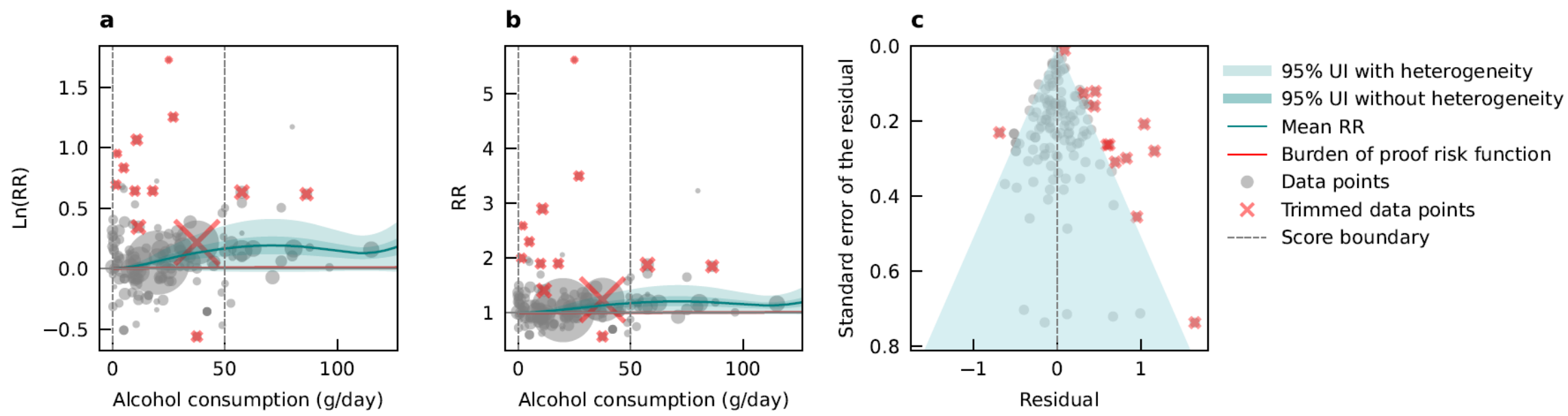

Right linear tail enforced

Score: -0.0, Star Rating: 1

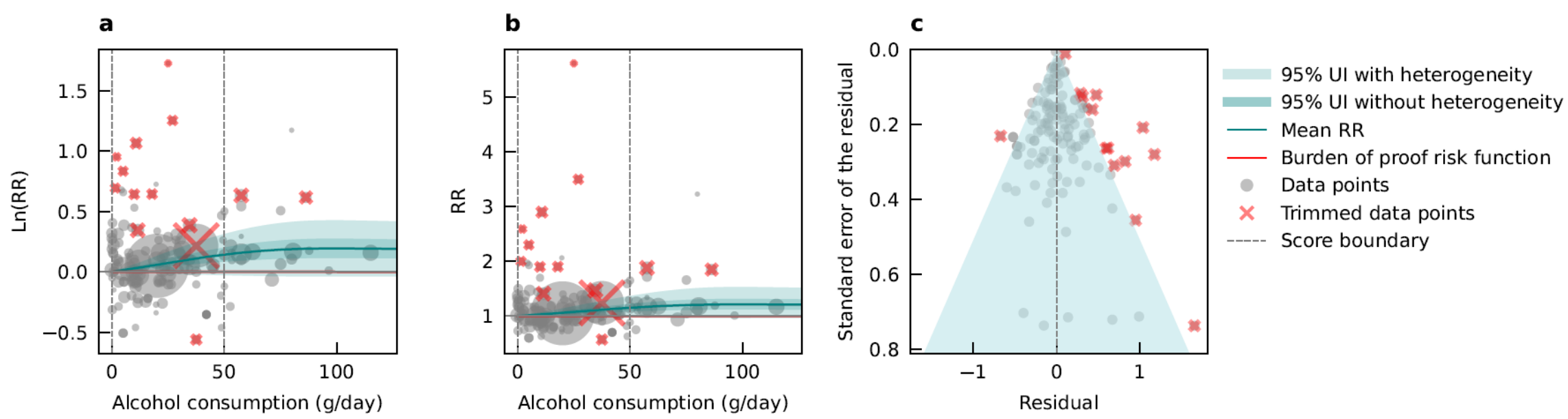

Monotonicity increasing enforced

Score: -0.0, Star Rating: 1

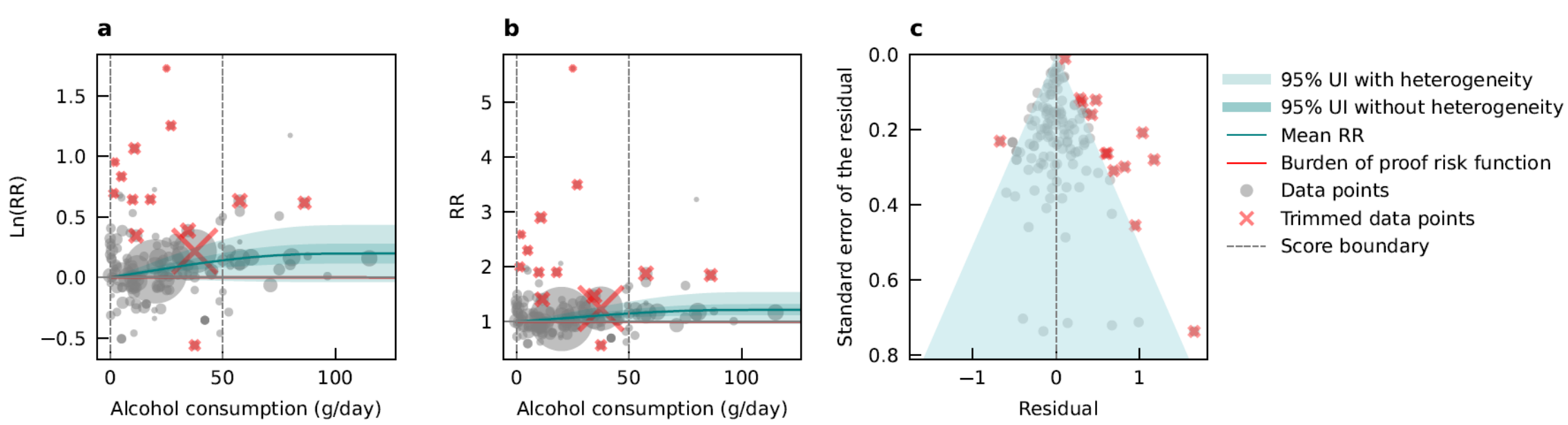

Right linear tail and monotonicity increasing enforced

Score: -0.0, Star Rating: 1

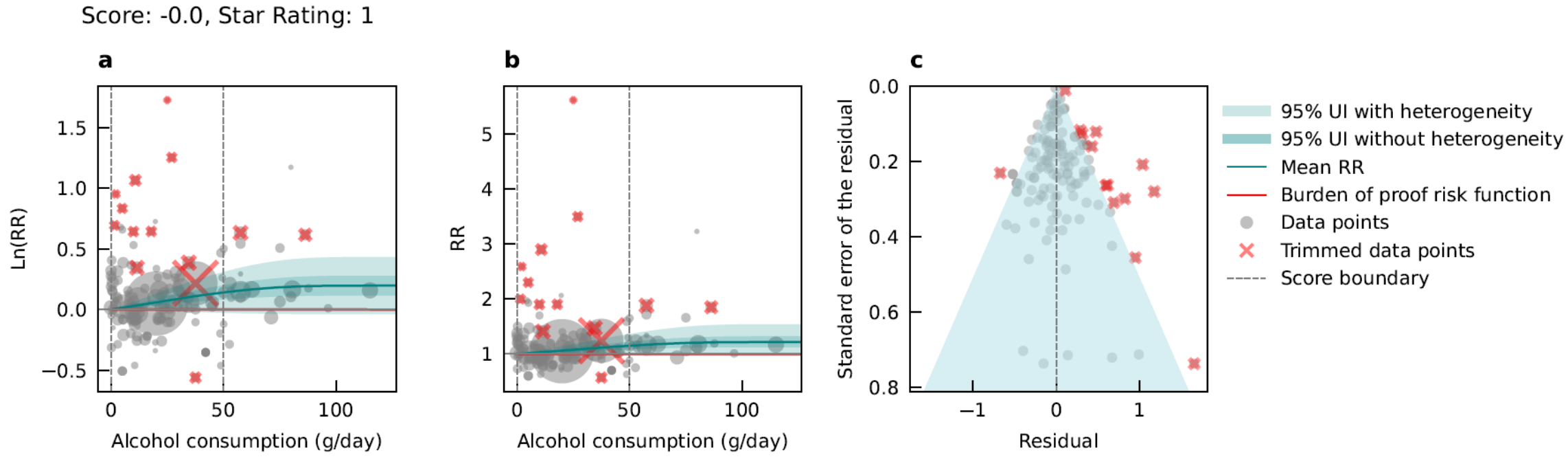

Figure S2.18. Ischemic stroke

No parameters enforced

Score: -0.01, Star Rating: 1

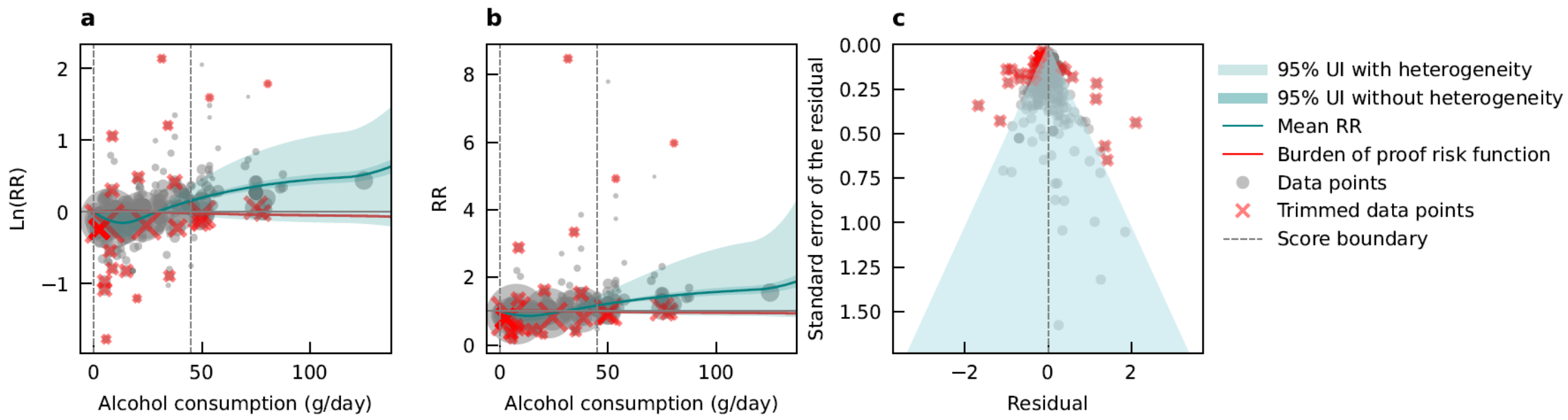

Right linear tail enforced

Score: -0.01, Star Rating: 1

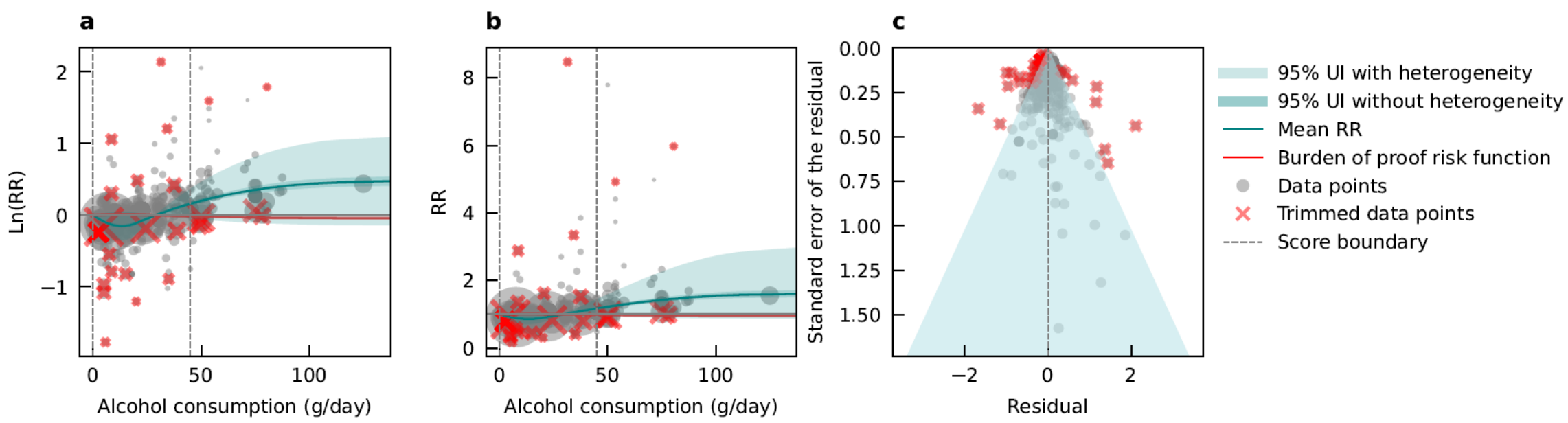

Monotonicity increasing enforced

Score: 0.0, Star Rating: 2

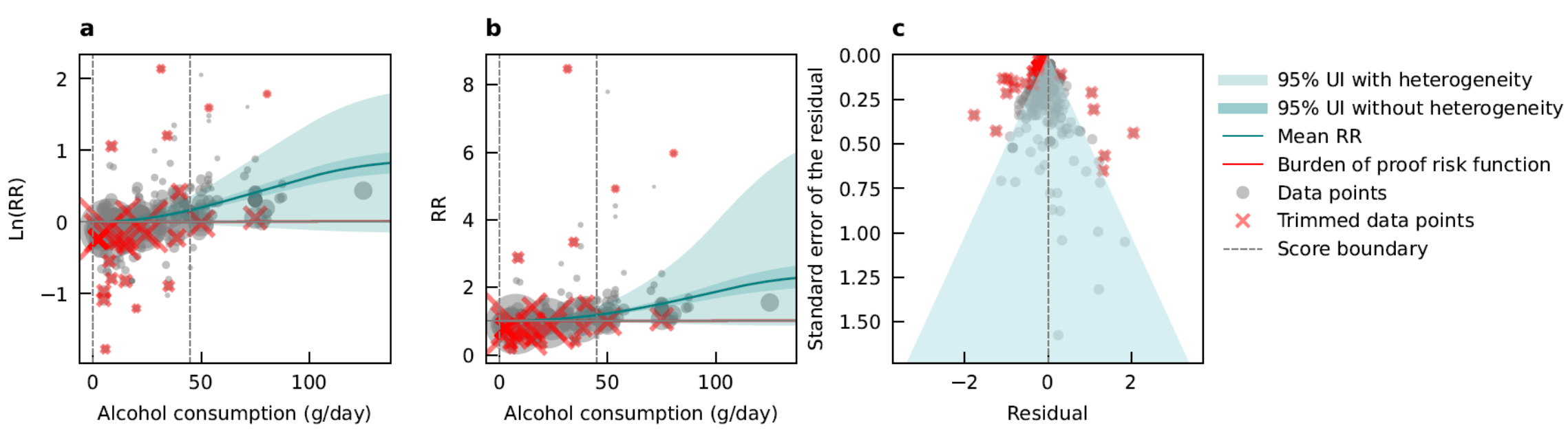

Right linear tail and monotonicity increasing enforced

Score: 0.0, Star Rating: 2

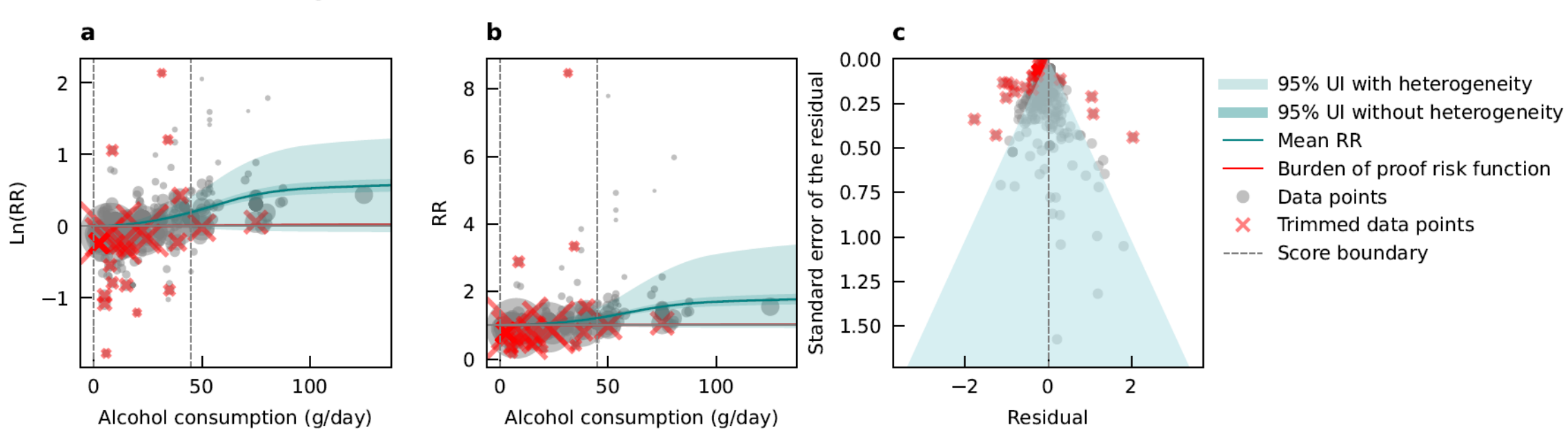

Figure S2.19. Hemorrhagic stroke

No parameters enforced

Score: -0.0, Star Rating: 1

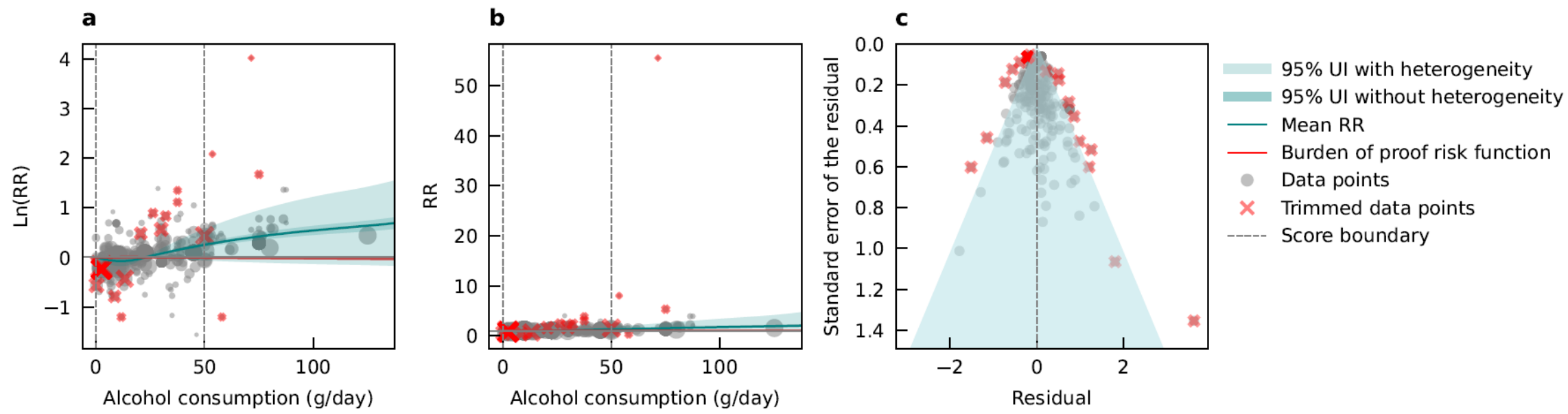

Right linear tail enforced

Score: -0.0, Star Rating: 1

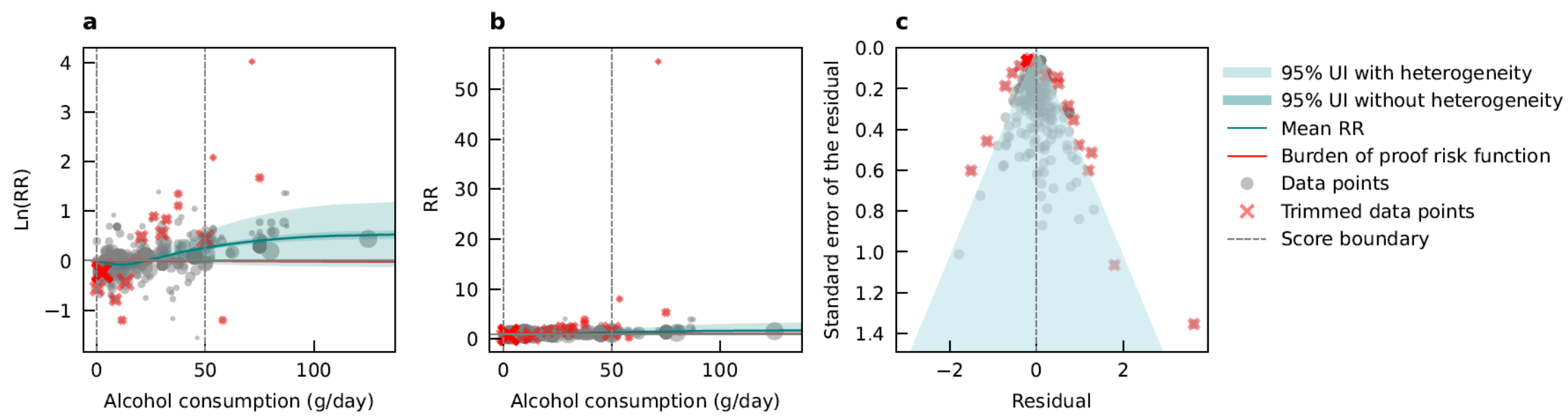

Monotonicity increasing enforced

Score: 0.01, Star Rating: 2

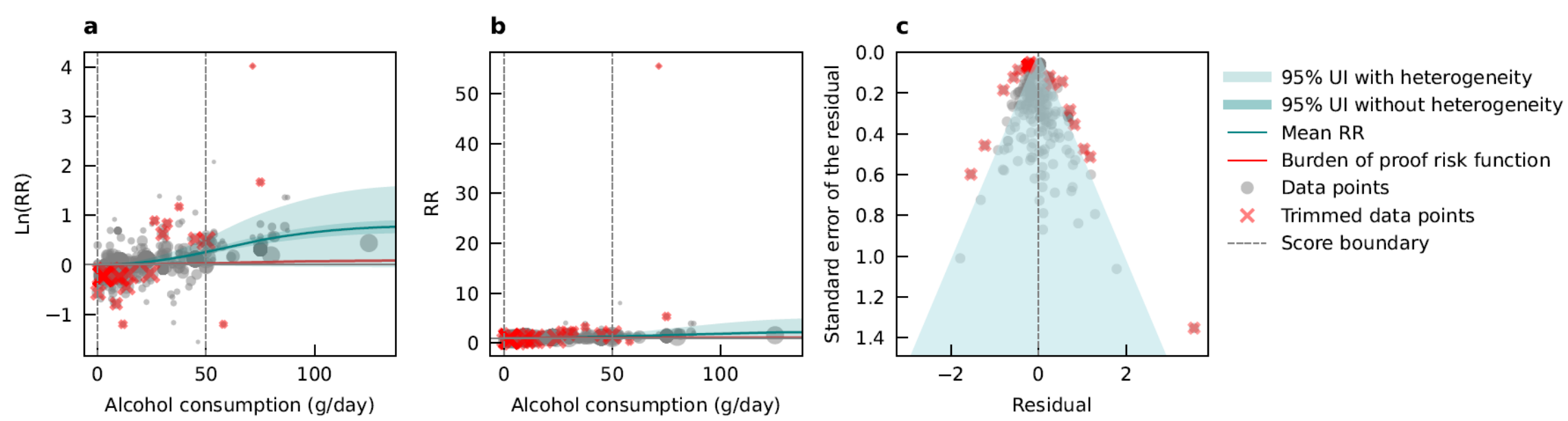

Right linear tail and monotonicity increasing enforced

Score: 0.01, Star Rating: 2

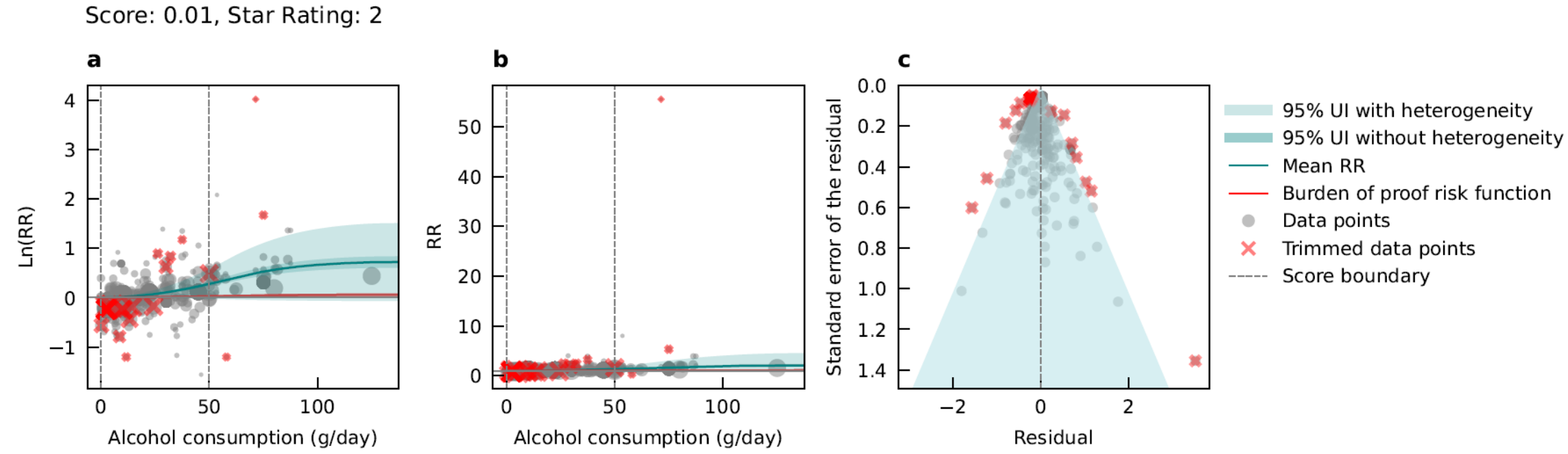

Figure S2.20. Tuberculosis

No parameters enforced

Score: 0.14, Star Rating: 2

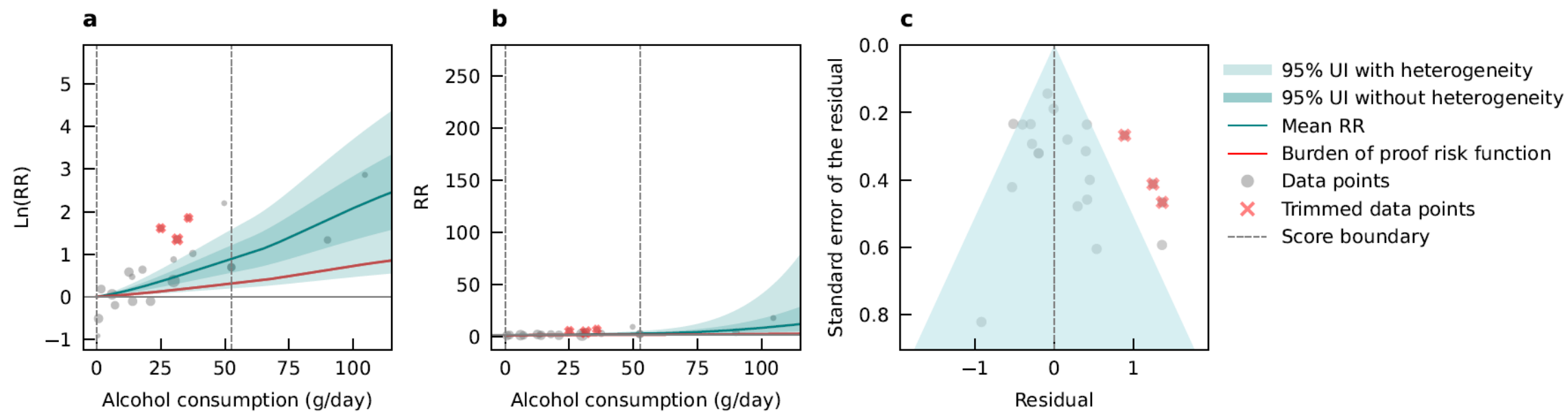

Right linear tail enforced

Score: -0.01, Star Rating: 1

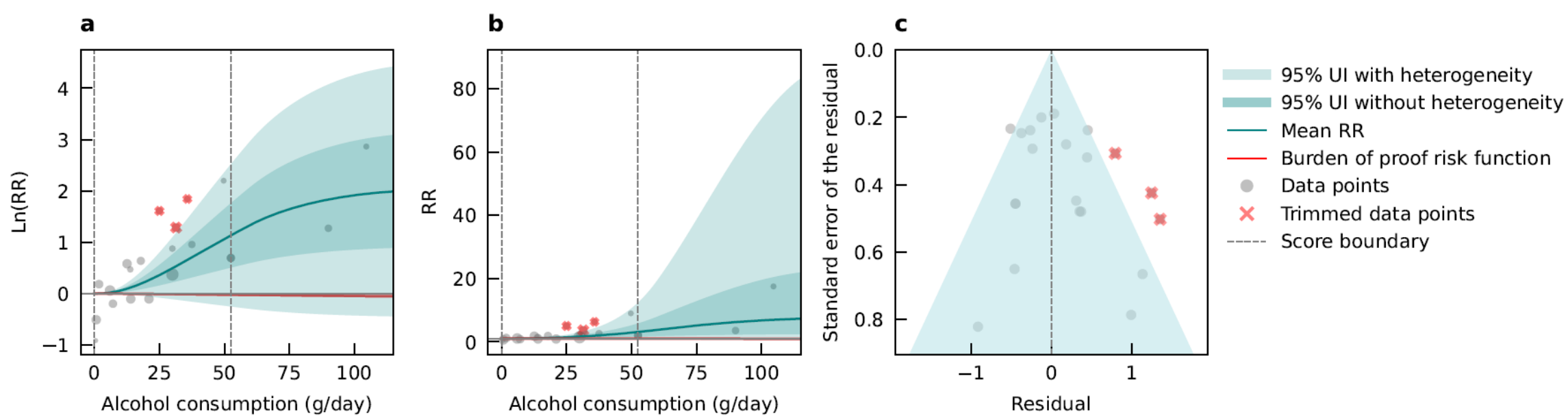

Monotonicity increasing enforced

Score: 0.14, Star Rating: 2

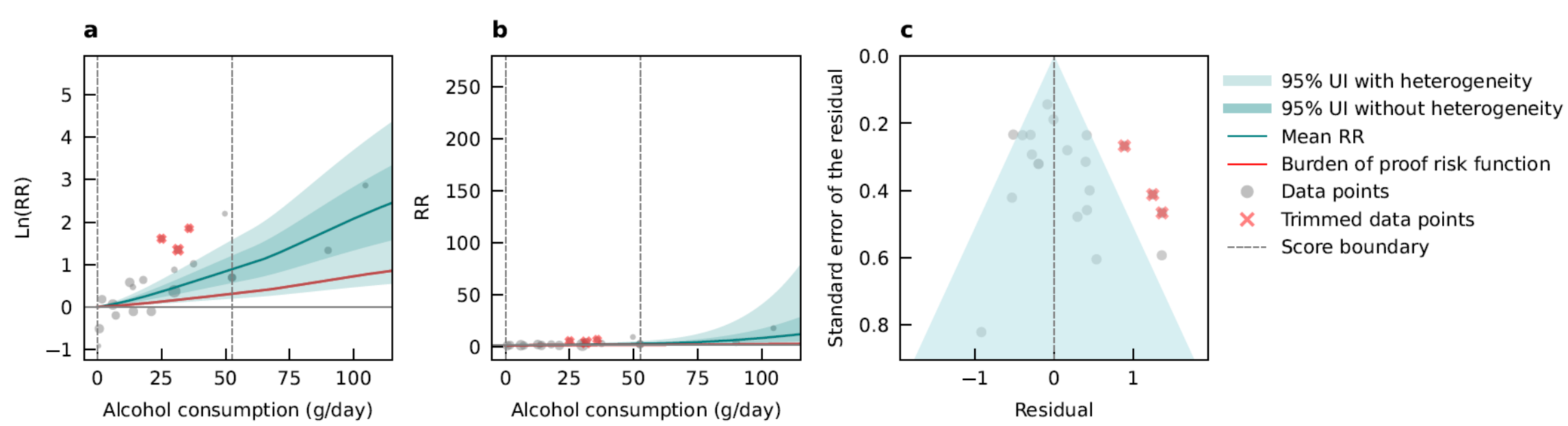

Right linear tail and monotonicity increasing enforced

Score: -0.02, Star Rating: 1

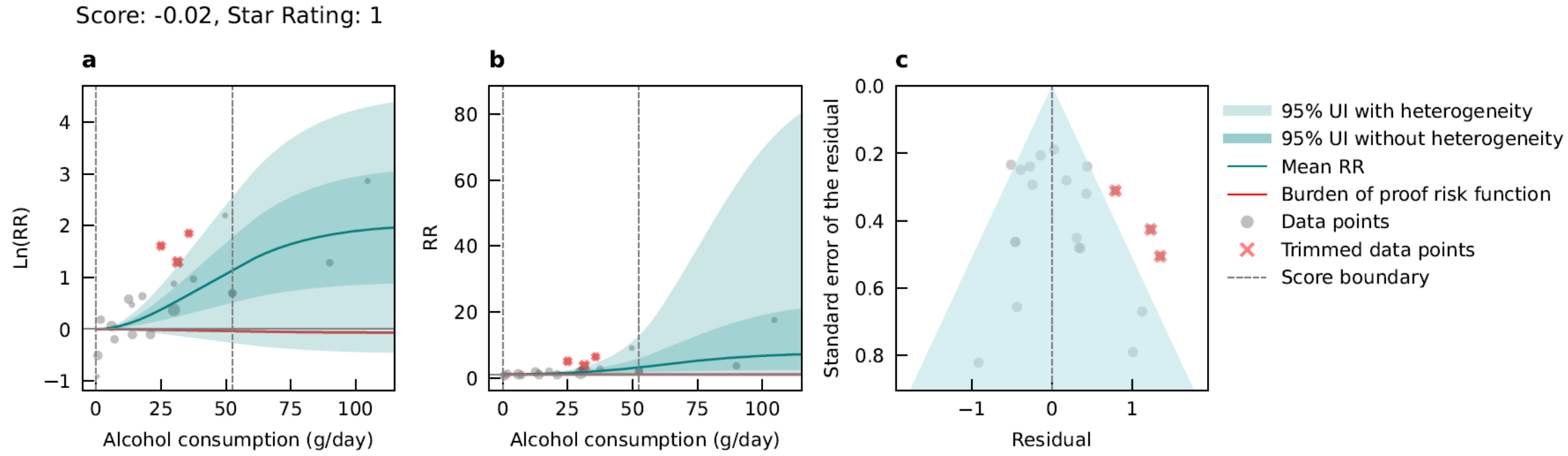

B. Relative Risk Models Without Trimming

The following risk curves are computed relative to no alcohol consumption. In panels (a) and (b), the dark line indicates mean relative risk across alcohol consumption levels (in g/day); the light and dark shading show 95% uncertainty intervals with and without between-study heterogeneity, respectively, with the red line highlighting the burden of proof function; the size of the data points corresponds to the inverse of the standard error, with those trimmed during the model fitting process marked by a red x; and the dashed lines represent the 15th percentile of the reference exposure and the 85th percentile of the alternative exposure. To visualize log-relative- risk points in panel (a), we plotted each data point with the x-value at the midpoint of the alternative group and the y-value offset by the difference between the reported and predicted log risk. In panel (b), we exponentiated the y-values from panel (a) to yield the relative risk curve. Panel (c) depicts a customized funnel plot, with the x-axis representing residuals between predicted and observed relative risks, and the y-axis representing uncertainty from both measurement error and between-study heterogeneity.

RR relative risk, UI uncertainty interval.

Figure S3.01. Atrial fibrillation

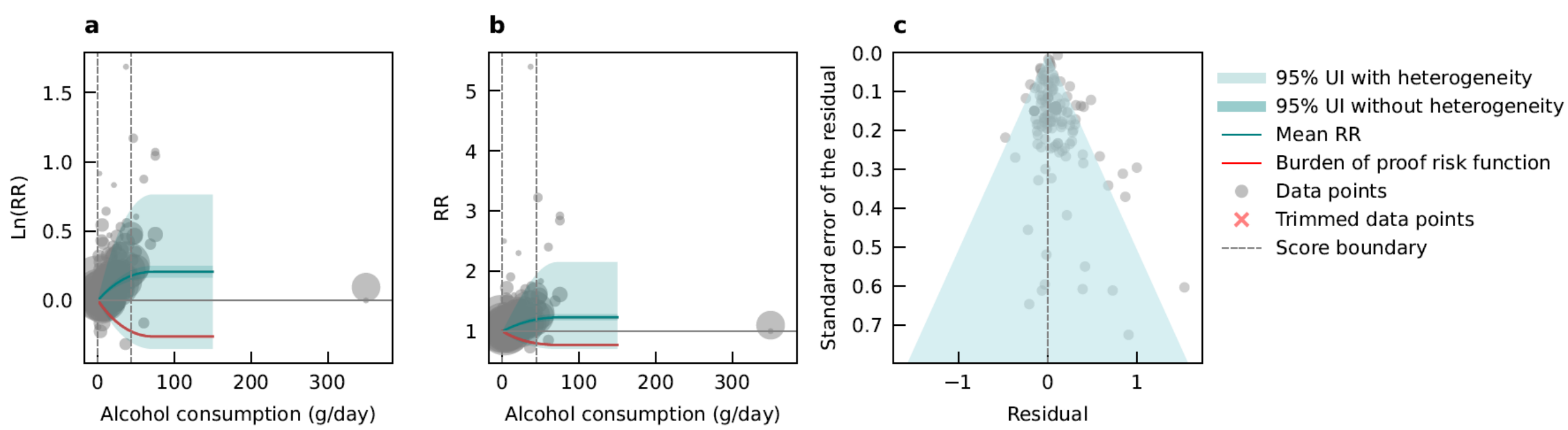

Figure S3.02. Breast cancer

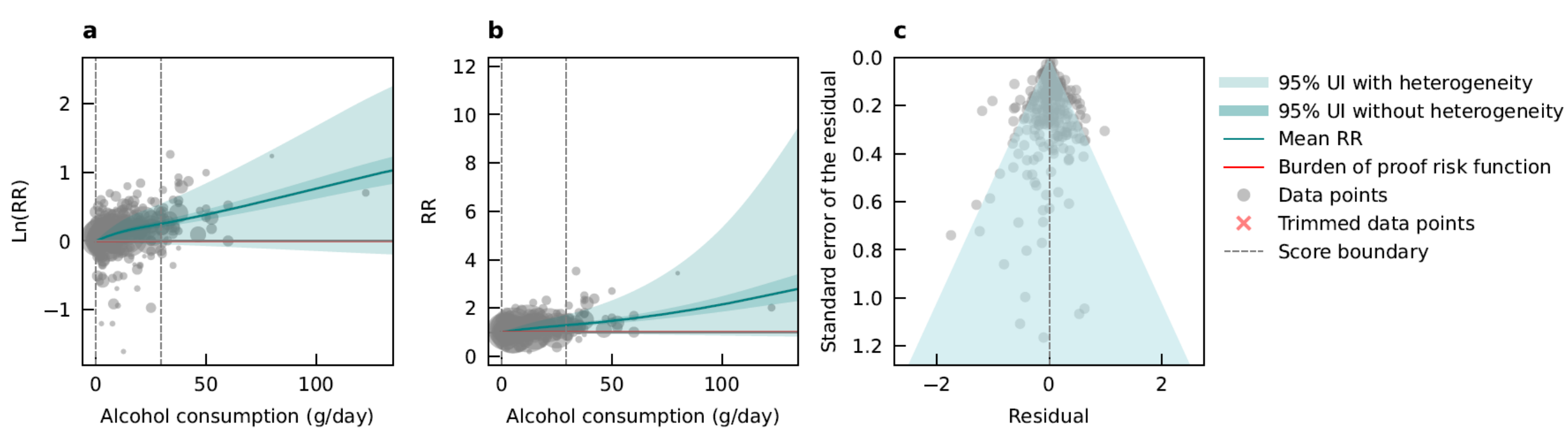

Figure S3.03. Cirrhosis and other chronic liver diseases

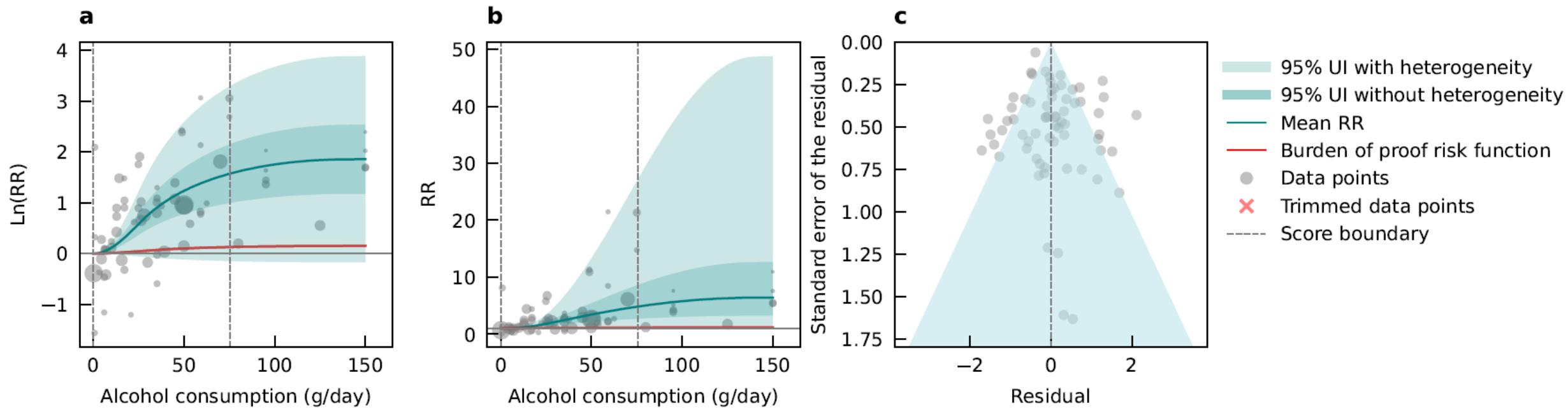

Figure S3.04. Colorectal cancer

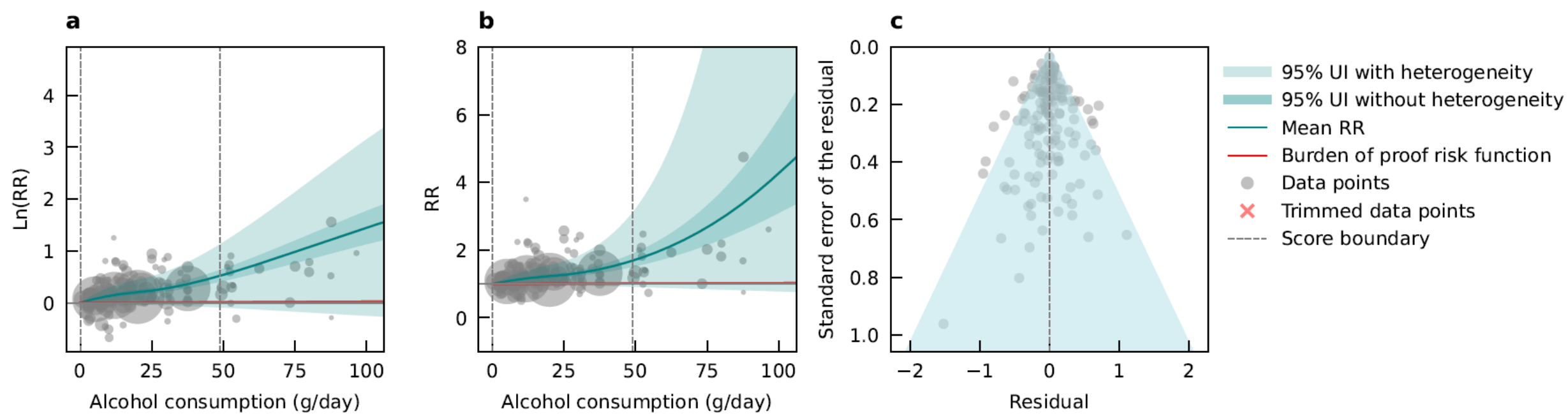

Figure S3.05. Alzheimer's disease and other dementias

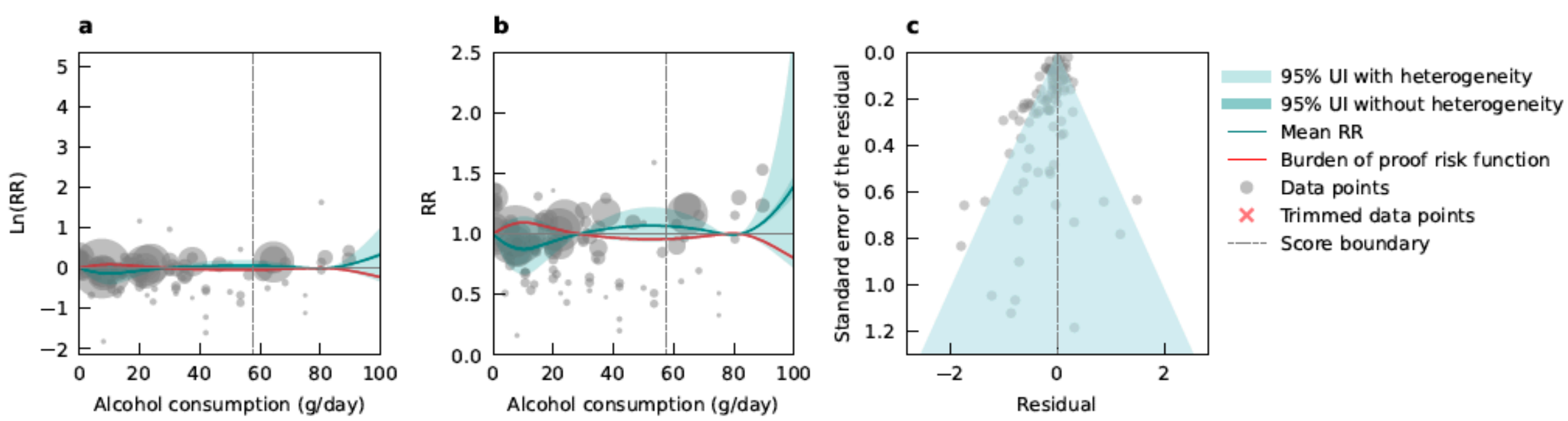

Figure S3.06. Type 2 diabetes

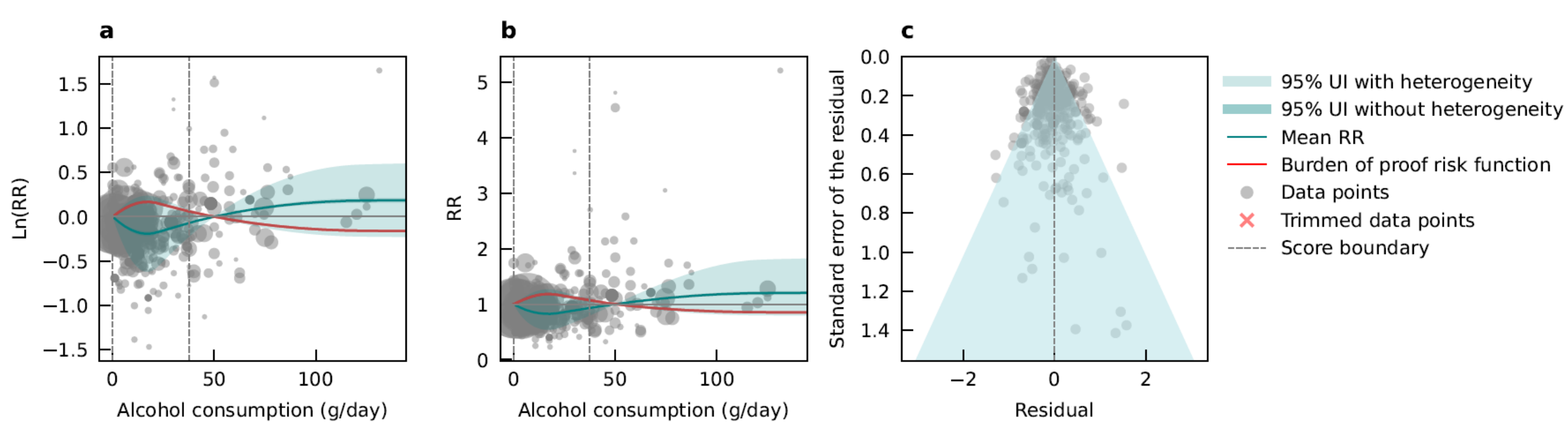

Figure S3.07. Esophageal cancer

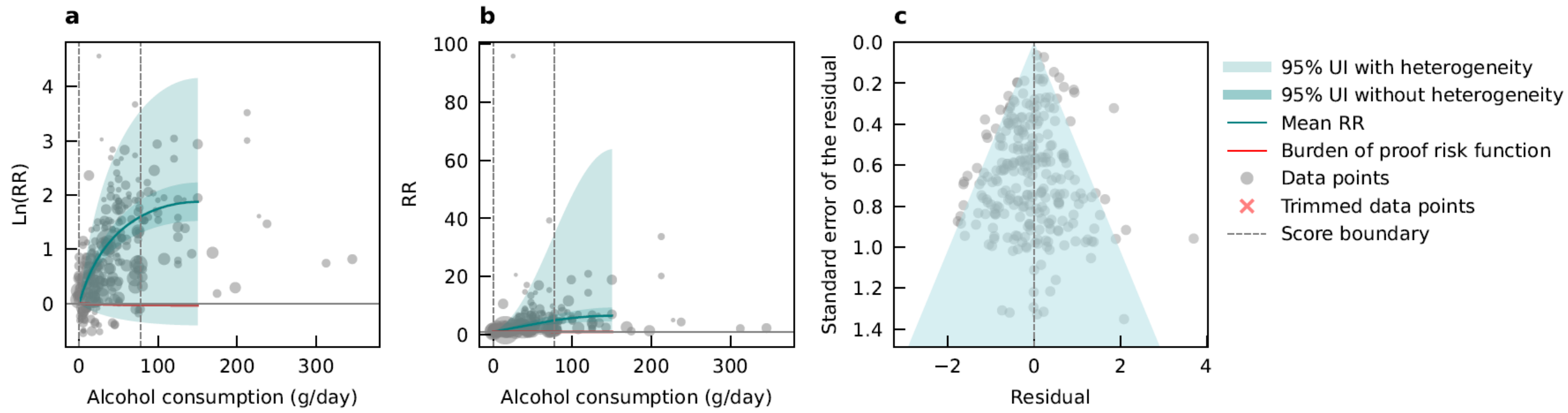

Figure S3.08. Larynx cancer

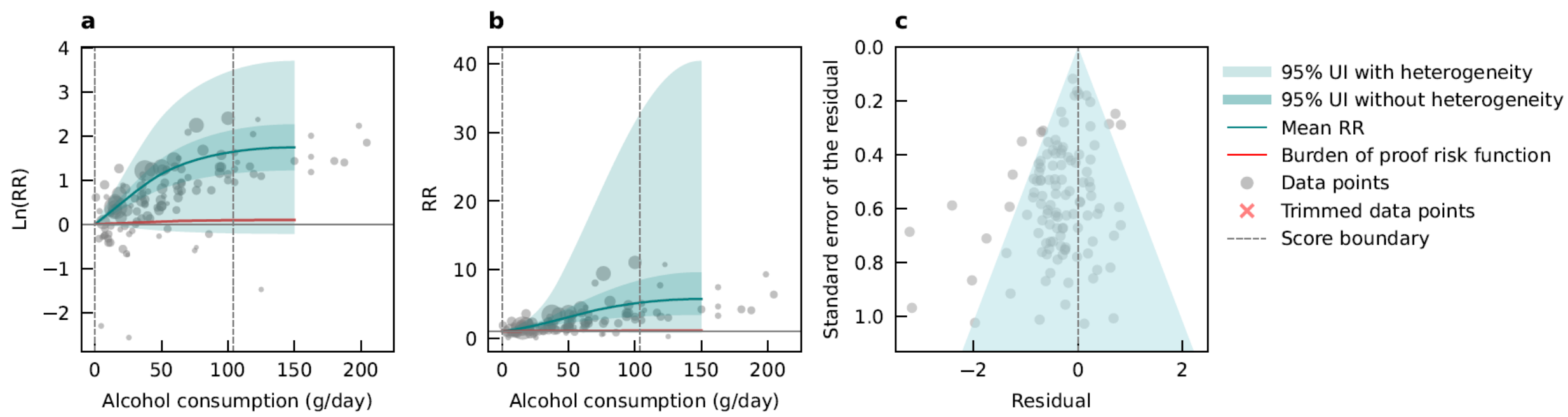

Figure S3.09. Mouth cancer

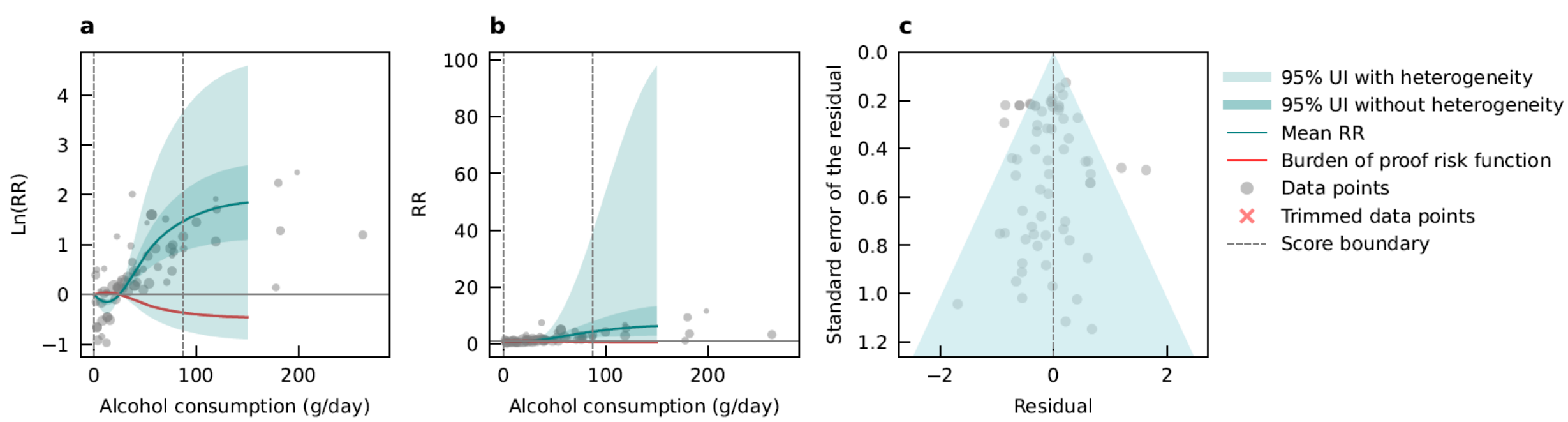

Figure S3.10. Other pharynx cancer

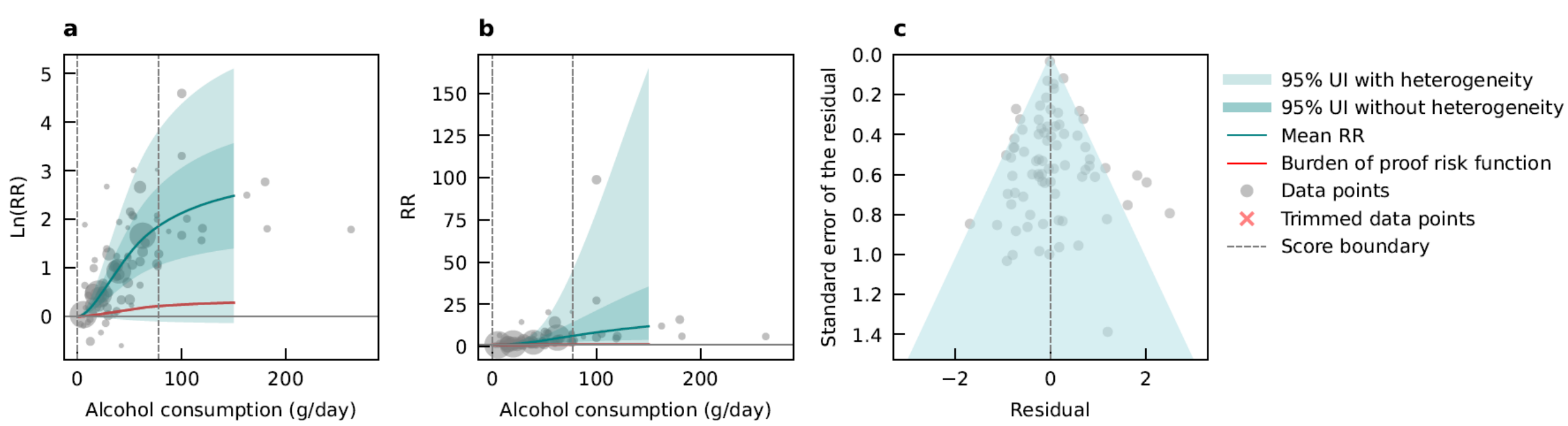

Figure S3.11. Ischemic heart disease

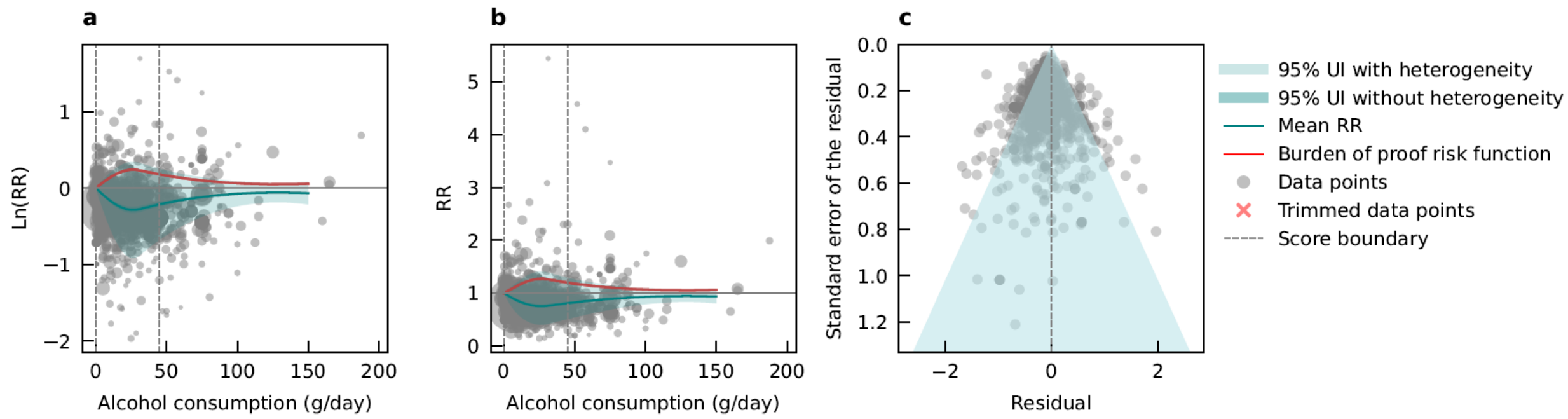

Figure S3.12. Liver cancer

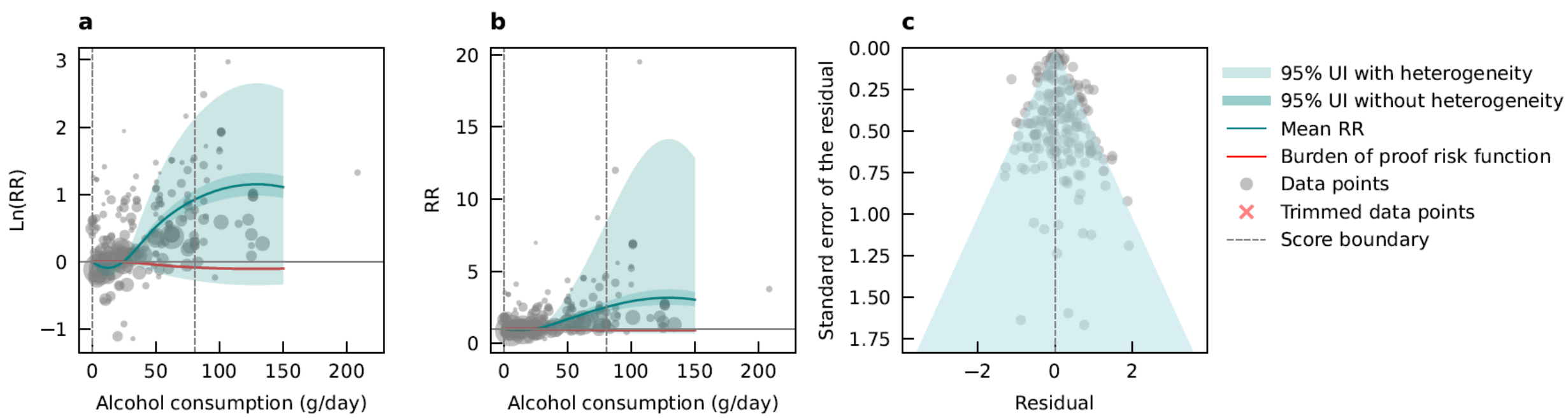

Figure S3.13. Lower respiratory infections

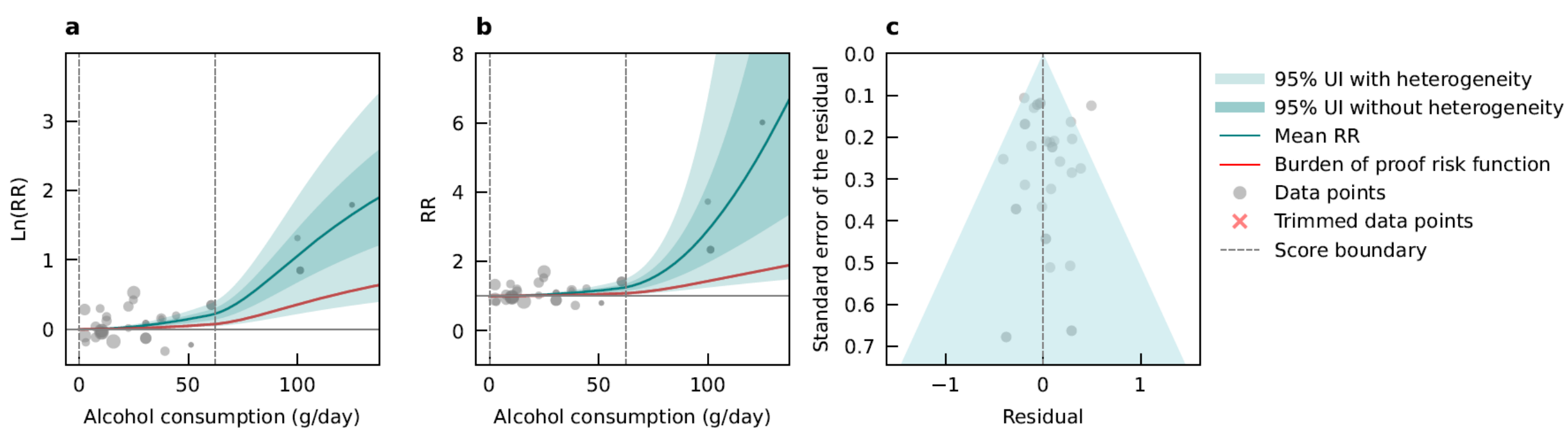

Figure S3.14. Pancreatitis

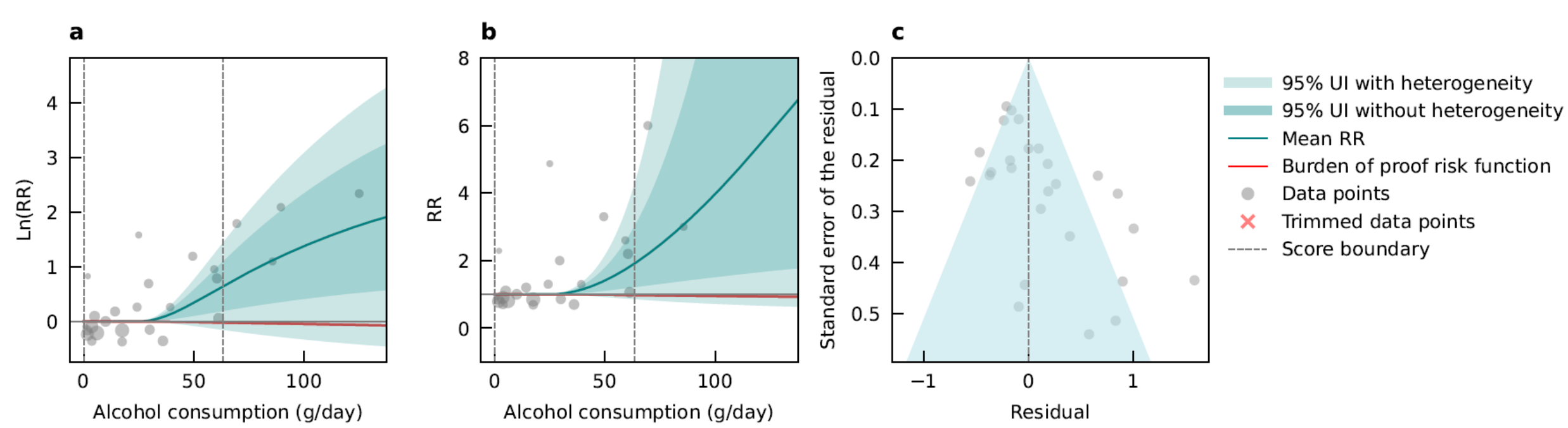

Figure S3.15. Pancreatic cancer

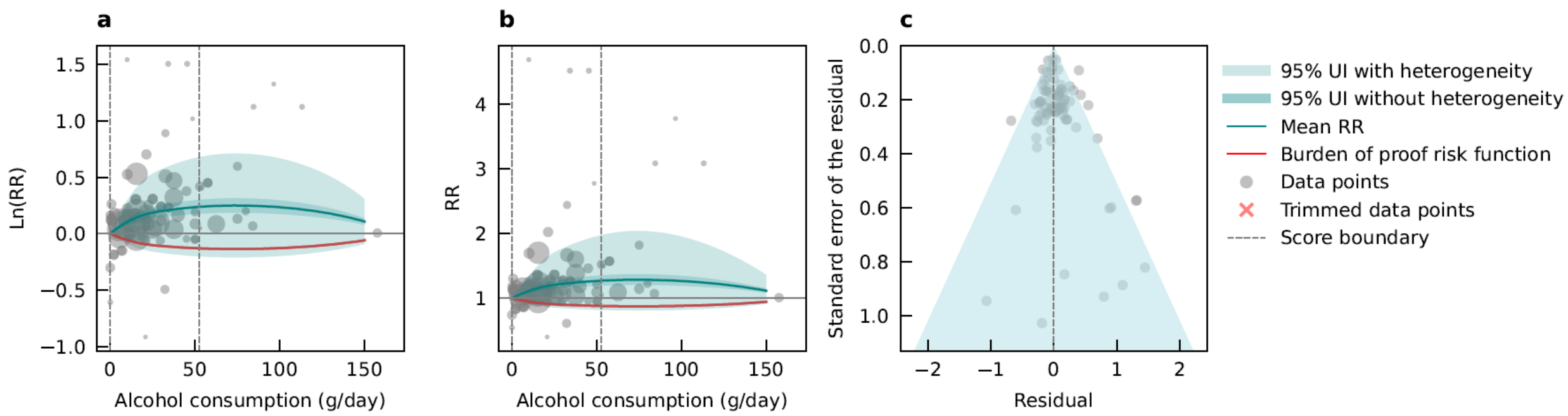

Figure S3.16. Prostate cancer

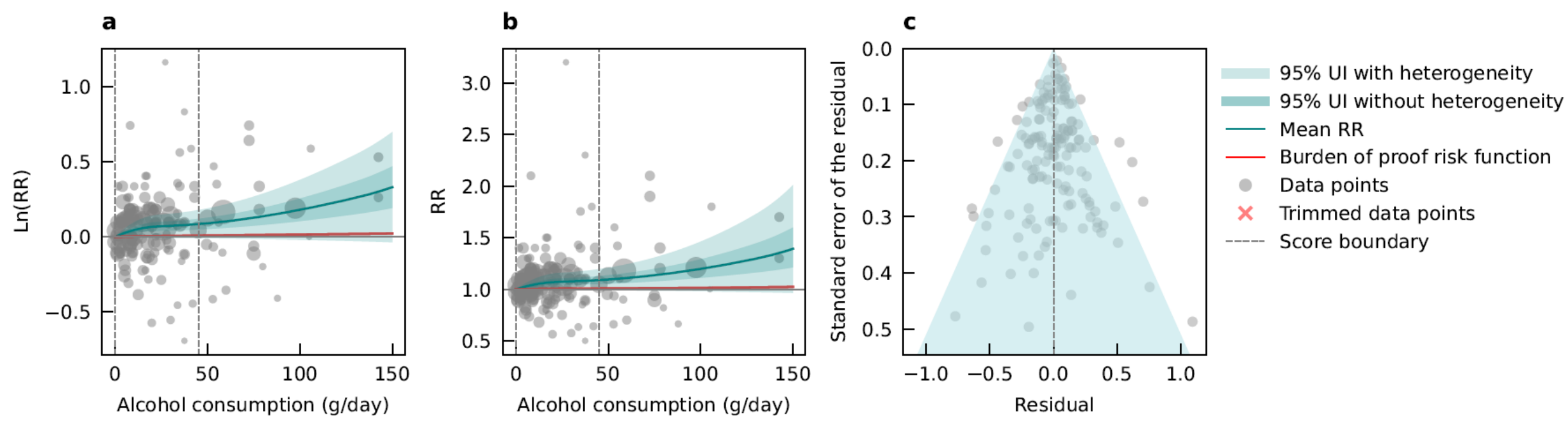

Figure S3.17. Stomach cancer

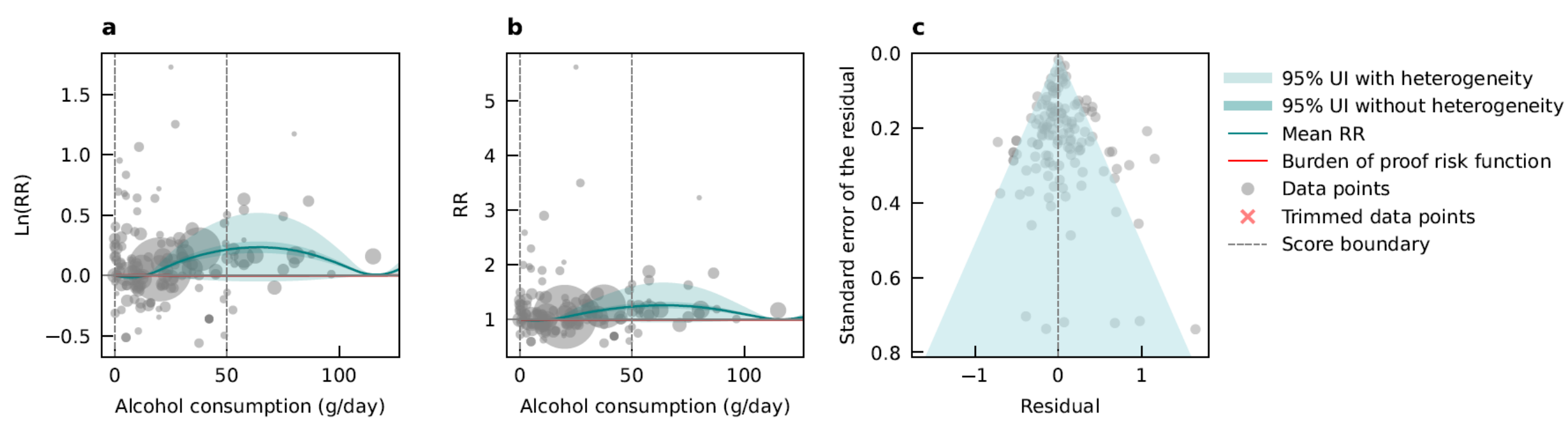

Figure S3.18. Ischemic stroke

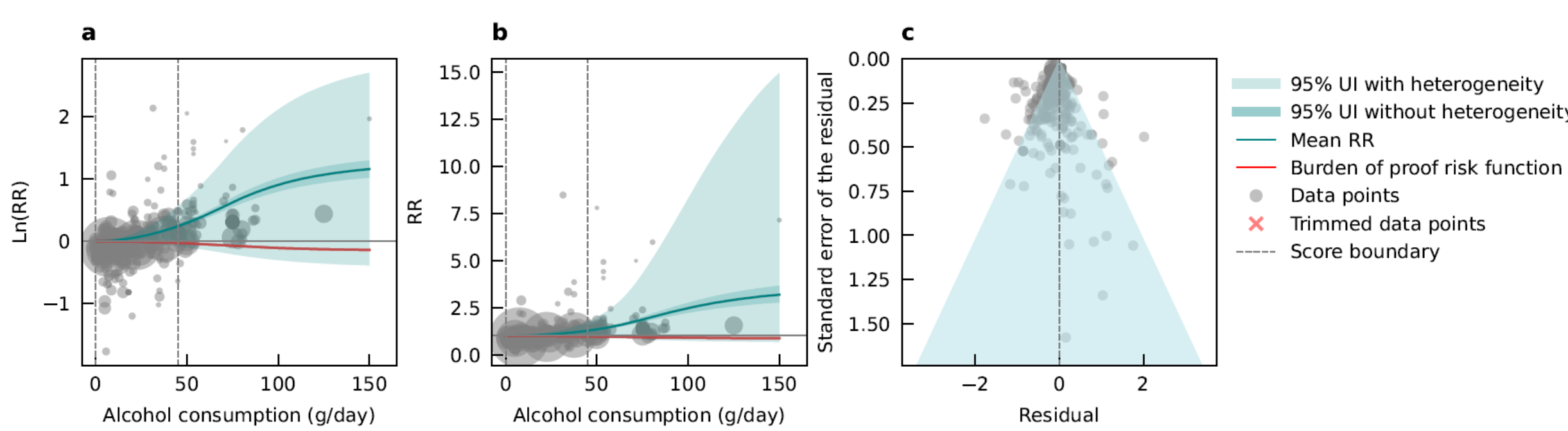

Figure S3.19. Hemorrhagic stroke

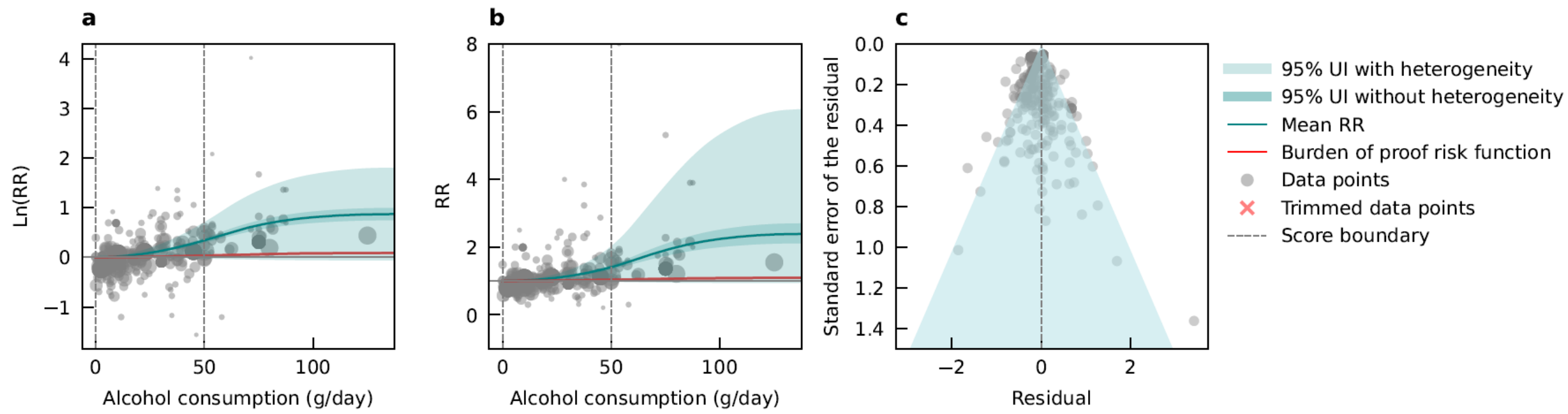

Figure S3.20. Tuberculosis

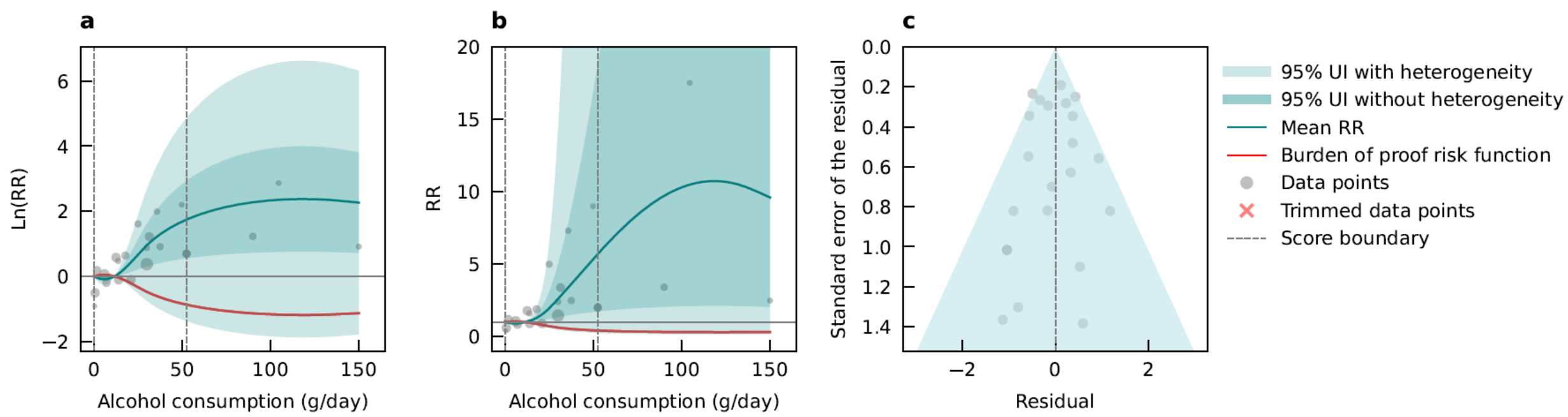

XII. Mean Risk at Different Levels of Consumption with Conventional 95% Uncertainty Intervals

Table S15. Relative risk of alcohol consumption at 1 standard drink (10 grams of alcohol per day) intervals for each health outcome

	Health outcome	Number of standard drinks									
		1	2	3	4	5	6	7	8	9	10
Cancers	Breast cancer	*1.13 (1.12, 1.15)*	*1.25 (1.21, 1.28)*	*1.33 (1.29, 1.38)*	*1.40 (1.34, 1.45)*	*1.44 (1.38, 1.50)*	*1.46 (1.39, 1.52)*	*1.47 (1.40, 1.54)*	*1.48 (1.41, 1.55)*	*1.49 (1.42, 1.56)*	*1.49 (1.42, 1.56)*
	Colon and rectum cancer	*1.17 (1.15, 1.20)*	*1.28 (1.24, 1.32)*	*1.35 (1.29, 1.40)*	*1.41 (1.34, 1.48)*	*1.46 (1.39, 1.54)*	*1.50 (1.42, 1.59)*	*1.53 (1.45, 1.63)*	*1.56 (1.47, 1.65)*	*1.57 (1.48, 1.67)*	*1.57 (1.48, 1.67)*
	Esophageal cancer	*1.32 (1.26, 1.39)*	*1.74 (1.58, 1.92)*	*2.26 (1.96, 2.61)*	*2.79 (2.32, 3.34)*	*3.28 (2.66, 4.04)*	*3.73 (2.96, 4.71)*	*4.14 (3.23, 5.33)*	*4.51 (3.46, 5.89)*	*4.83 (3.66, 6.38)*	*5.11 (3.83, 6.81)*
	Larynx cancer	*1.23 (1.19, 1.28)*	*1.48 (1.39, 1.58)*	*1.74 (1.59, 1.91)*	*2.02 (1.80, 2.27)*	*2.31 (2.01, 2.66)*	*2.61 (2.23, 3.07)*	*2.92 (2.44, 3.50)*	*3.20 (2.64, 3.89)*	*3.44 (2.80, 4.23)*	*3.64 (2.93, 4.51)*
	Lip and oral cavity cancer	*1.03 (1.02, 1.03)*	*1.11 (1.09, 1.14)*	*1.26 (1.20, 1.32)*	*1.46 (1.35, 1.58)*	*1.73 (1.55, 1.93)*	*2.05 (1.78, 2.37)*	*2.43 (2.03, 2.90)*	*2.83 (2.30, 3.48)*	*3.24 (2.56, 4.11)*	*3.68 (2.83, 4.78)*
	Liver cancer	*1.02 (1.02, 1.02)*	*1.07 (1.06, 1.08)*	*1.17 (1.14, 1.19)*	*1.30 (1.25, 1.35)*	*1.48 (1.40, 1.57)*	*1.71 (1.58, 1.85)*	*1.98 (1.79, 2.20)*	*2.24 (1.99, 2.53)*	*2.46 (2.16, 2.82)*	*2.64 (2.29, 3.05)*
	Pancreatic cancer	*1.05 (1.04, 1.07)*	*1.10 (1.07, 1.13)*	*1.13 (1.09, 1.18)*	*1.16 (1.11, 1.21)*	*1.18 (1.12, 1.24)*	*1.19 (1.13, 1.25)*	*1.20 (1.13, 1.26)*	*1.20 (1.14, 1.27)*	*1.21 (1.14, 1.28)*	*1.21 (1.14, 1.28)*
	Other pharynx cancer	*1.16 (1.14, 1.17)*	*1.56 (1.51, 1.61)*	*2.16 (2.04, 2.29)*	*2.73 (2.53, 2.94)*	*3.23 (2.96, 3.52)*	*3.67 (3.33, 4.04)*	*4.04 (3.65, 4.48)*	*4.35 (3.90, 4.85)*	*4.58 (4.10, 5.13)*	*4.75 (4.24, 5.33)*
	Prostate cancer	*1.04*	*1.08*	*1.10*	*1.11*	*1.13*	*1.14*	*1.16*	*1.17*	*1.18*	*1.19*

		(1.03, 1.06)	(1.05, 1.10)	(1.06, 1.13)	(1.07, 1.16)	(1.08, 1.18)	(1.09, 1.20)	(1.10, 1.22)	(1.10, 1.24)	(1.11, 1.25)	(1.12, 1.26)
	Stomach cancer	1.03<br>(1.02, 1.04)	1.06<br>(1.04, 1.09)	1.09<br>(1.05, 1.14)	1.12<br>(1.07, 1.18)	1.15<br>(1.09, 1.22)	1.17<br>(1.10, 1.26)	1.19<br>(1.11, 1.28)	1.21<br>(1.12, 1.30)	1.21<br>(1.12, 1.32)	1.22<br>(1.12, 1.32)
Cardiovascular Diseases	Atrial fibrillation and flutter	1.02<br>(1.02, 1.03)	1.10<br>(1.09, 1.11)	1.20<br>(1.18, 1.22)	1.29<br>(1.26, 1.32)	1.37<br>(1.33, 1.41)	1.44<br>(1.39, 1.49)	1.50<br>(1.44, 1.56)	1.54<br>(1.48, 1.61)	1.58<br>(1.51, 1.65)	1.60<br>(1.53, 1.68)
	Ischemic heart disease	0.92<br>(0.91, 0.93)	0.84<br>(0.82, 0.86)	0.77<br>(0.74, 0.79)	0.72<br>(0.69, 0.75)	0.70<br>(0.67, 0.73)	0.71<br>(0.68, 0.74)	0.75<br>(0.72, 0.78)	0.81<br>(0.79, 0.84)	0.90<br>(0.89, 0.91)	1.01<br>(1.01, 1.02)
	Ischemic stroke	0.87<br>(0.85, 0.88)	0.89<br>(0.88, 0.91)	1.01<br>(1.01, 1.01)	1.12<br>(1.10, 1.14)	1.22<br>(1.19, 1.26)	1.31<br>(1.26, 1.36)	1.39<br>(1.33, 1.45)	1.46<br>(1.38, 1.53)	1.51<br>(1.43, 1.59)	1.55<br>(1.46, 1.64)
	Haemorrhagic stroke	0.92<br>(0.91, 0.93)	0.97<br>(0.96, 0.97)	1.09<br>(1.07, 1.10)	1.20<br>(1.16, 1.23)	1.30<br>(1.24, 1.35)	1.38<br>(1.32, 1.46)	1.46<br>(1.38, 1.55)	1.53<br>(1.43, 1.63)	1.58<br>(1.47, 1.70)	1.62<br>(1.50, 1.75)
Other Diseases	Alzheimer's disease and other dementias	0.89<br>(0.87, 0.90)	0.83<br>(0.81, 0.85)	0.82<br>(0.80, 0.84)	0.87<br>(0.85, 0.88)	0.94<br>(0.93, 0.95)	1.02<br>(1.01, 1.02)	1.10<br>(1.09, 1.12)	1.19<br>(1.16, 1.22)	1.29<br>(1.24, 1.34)	1.40<br>(1.33, 1.46)
	Cirrhosis and other chronic liver diseases	1.12<br>(1.09, 1.15)	1.40<br>(1.29, 1.51)	1.83<br>(1.60, 2.11)	2.43<br>(1.99, 2.98)	3.19<br>(2.45, 4.16)	4.11<br>(2.98, 5.68)	5.00<br>(3.46, 7.22)	5.71<br>(3.83, 8.49)	6.23<br>(4.10, 9.46)	6.56<br>(4.27, 10.09)
	Type 2 diabetes	0.84<br>(0.82, 0.86)	0.80<br>(0.78, 0.83)	0.87<br>(0.85, 0.89)	0.95<br>(0.94, 0.96)	1.02<br>(1.02, 1.03)	1.09<br>(1.08, 1.10)	1.14<br>(1.12, 1.17)	1.19<br>(1.16, 1.22)	1.23<br>(1.19, 1.26)	1.26<br>(1.22, 1.30)
	Lower respiratory infections	1.00<br>(1.00, 1.00)	1.00<br>(1.00, 1.00)	1.01<br>(1.00, 1.01)	1.06<br>(1.03, 1.09)	1.17<br>(1.09, 1.25)	1.34<br>(1.18, 1.52)	1.56<br>(1.29, 1.90)	1.85<br>(1.41, 2.42)	2.20<br>(1.56, 3.11)	2.61<br>(1.71, 3.98)
	Pancreatitis	1.01	1.03	1.13	1.41	1.85	2.43	3.15	3.99	4.95	6.02

		(1.01, 1.01)	*(1.02, 1.04)*	*(1.09, 1.18)*	*(1.27, 1.56)*	*(1.54, 2.21)*	*(1.87, 3.16)*	*(2.24, 4.41)*	*(2.65, 6.00)*	*(3.08, 7.94)*	*(3.54, 10.24)*
	Tuberculosis	*1.07*<br>*(1.03, 1.11)*	*1.29*<br>*(1.12, 1.48)*	*1.66*<br>*(1.26, 2.20)*	*2.20*<br>*(1.43, 3.40)*	*2.92*<br>*(1.62, 5.27)*	*3.83*<br>*(1.83, 8.02)*	*4.76*<br>*(2.02, 11.24)*	*5.55*<br>*(2.16, 14.26)*	*6.19*<br>*(2.27, 16.89)*	*6.67*<br>*(2.35, 18.97)*

The reported relative risk (RR) and its 95% uncertainty interval (UI) reflect the risk of developing each outcome of interest when comparing levels of current alcohol use versus no alcohol use.

XIII. PRISMA Checklists

A. PRISMA 2020 Abstract Checklist

Table S16. PRISMA 2020 Abstract Checklist

Section and Topic	Item #	Checklist item	Reported (Yes/No)
**TITLE**			
Title	1	Identify the report as a systematic review.	Identified in the abstract but not the title. The title reflects the review and meta-analysis methods.
**BACKGROUND**			
Objectives	2	Provide an explicit statement of the main objective(s) or question(s) the review addresses.	Yes
**METHODS**			
Eligibility criteria	3	Specify the inclusion and exclusion criteria for the review.	Yes
Information sources	4	Specify the information sources (e.g. databases, registers) used to identify studies and the date when each was last searched.	Number of databases is provided; due to space limitations, specific information is provided in the main text and Supplementary Information section IV.
Risk of bias	5	Specify the methods used to assess risk of bias in the included studies.	Yes; due to space limitations, information is provided in the main text and Supplementary Information sections VIII and X.

Synthesis of results	6	Specify the methods used to present and synthesise results.	Yes, more information provided in the main text and Supplementary Information section X.
**RESULTS**			
Included studies	7	Give the total number of included studies and participants and summarise relevant characteristics of studies.	Yes, number of studies is provided but additional details can be found in the main text and Supplementary Information sections V, VIII, IX.
Synthesis of results	8	Present results for main outcomes, preferably indicating the number of included studies and participants for each. If meta-analysis was done, report the summary estimate and confidence/credible interval. If comparing groups, indicate the direction of the effect (i.e. which group is favoured).	Due to space limitations, a general summary of results is provided. Specific details are included in the main text, in Table 2, and in Supplementary Information section IX.
**DISCUSSION**			
Limitations of evidence	9	Provide a brief summary of the limitations of the evidence included in the review (e.g. study risk of bias, inconsistency and imprecision).	Not in the abstract, described in detail within the main text.
Interpretation	10	Provide a general interpretation of the results and important implications.	Yes.
**OTHER**			
Funding	11	Specify the primary source of funding for the review.	Not in the abstract, described in detail in the "Acknowledgements" section.

Registration	12	Provide the register name and registration number.	Not in abstract, provided in the main text, methods section.

B. PRISMA 2020 manuscript checklist

Table S17. PRISMA 2020 manuscript checklist

Section and Topic	Item #	Checklist item	Location where item is reported
**TITLE**			
Title	1	Identify the report as a systematic review.	Not in the title, which reflects analytical framework and scope of this review. This is explicitly stated in the abstract, introduction, methods, and discussion sections of the manuscript.
**ABSTRACT**			
Abstract	2	See the PRISMA 2020 for Abstracts checklist.	See Supplementary Information Table S18
**INTRODUCTION**			
Rationale	3	Describe the rationale for the review in the context of existing knowledge.	Main (paragraphs 2 and 3)
Objectives	4	Provide an explicit statement of the objective(s) or question(s) the review addresses.	Main (paragraph 4)
**METHODS**			
Eligibility criteria	5	Specify the inclusion and exclusion criteria for the review and how studies were grouped for the syntheses.	Methods ("The Pragmatic Systematic Review"); Supplementary Information Table S2
Information sources	6	Specify all databases, registers, websites, organisations, reference lists and other sources searched or consulted to identify studies. Specify the date when each source was last searched or consulted.	Supplementary Information section IV

Search strategy	7	Present the full search strategies for all databases, registers and websites, including any filters and limits used.	Methods ("The Pragmatic Systematic Review"); Supplementary Information section IV
Selection process	8	Specify the methods used to decide whether a study met the inclusion criteria of the review, including how many reviewers screened each record and each report retrieved, whether they worked independently, and if applicable, details of automation tools used in the process.	Methods ("The Pragmatic Systematic Review"); Supplementary Information section III
Data collection process	9	Specify the methods used to collect data from reports, including how many reviewers collected data from each report, whether they worked independently, any processes for obtaining or confirming data from study investigators, and if applicable, details of automation tools used in the process.	Methods ("The Pragmatic Systematic Review"); Supplementary Information section VI
Data items	10a	List and define all outcomes for which data were sought. Specify whether all results that were compatible with each outcome domain in each study were sought (e.g. for all measures, time points, analyses), and if not, the methods used to decide which results to collect.	Methods ("Overview"); Supplementary Information sections IV-VI
	10b	List and define all other variables for which data were sought (e.g. participant and intervention characteristics, funding sources). Describe any assumptions made about any missing or unclear information.	Supplementary Information sections VI and VIII

Study risk of bias assessment	11	Specify the methods used to assess risk of bias in the included studies, including details of the tool(s) used, how many reviewers assessed each study and whether they worked independently, and if applicable, details of automation tools used in the process.	Methods ("Testing and adjusting for biases across study designs and characteristics"); Supplementary Information Table S12
Effect measures	12	Specify for each outcome the effect measure(s) (e.g. risk ratio, mean difference) used in the synthesis or presentation of results.	Methods ("Estimating the shape of the exposure-relative risk relationship" and "Estimating the burden of proof risk function"); Table 2
Synthesis methods	13a	Describe the processes used to decide which studies were eligible for each synthesis (e.g. tabulating the study intervention characteristics and comparing against the planned groups for each synthesis (item #5)).	Methods ("The Pragmatic Systematic Review" and "Estimating the shape of the exposure-relative risk relationship"); Supplementary Information V
	13b	Describe any methods required to prepare the data for presentation or synthesis, such as handling of missing summary statistics, or data conversions.	Methods ("Estimating the shape of the exposure-relative risk relationship" and "Estimating the burden of proof risk function"); Supplementary Information Section VII
	13c	Describe any methods used to tabulate or visually display results of individual studies and syntheses.	Methods ("Evaluating the potential publication and reporting biases" and "Estimating the burden of proof risk function"); Supplementary Information section IX and XII
	13d	Describe any methods used to synthesize results and provide a rationale for the choice(s). If meta-analysis was performed, describe the model(s), method(s) to identify the	Methods ("Estimating the shape of the exposure-relative risk relationship" and "Estimating the burden of proof risk function")

		presence and extent of statistical heterogeneity, and software package(s) used.	
	13e	Describe any methods used to explore possible causes of heterogeneity among study results (e.g. subgroup analysis, meta-regression).	Methods ("Testing and adjusting for biases across study designs and characteristics")
	13f	Describe any sensitivity analyses conducted to assess robustness of the synthesized results.	Methods ("Estimating the shape of the exposure-relative risk relationship"); Supplementary Information section XII
Reporting bias assessment	14	Describe any methods used to assess risk of bias due to missing results in a synthesis (arising from reporting biases).	Methods ("Testing and adjusting for biases across study designs and characteristics"); Supplementary Information section X
Certainty assessment	15	Describe any methods used to assess certainty (or confidence) in the body of evidence for an outcome.	Methods ("Estimating the burden of proof risk function"); Table 2; Supplementary Information Table S14
**RESULTS**			
Study selection	16a	Describe the results of the search and selection process, from the number of records identified in the search to the number of studies included in the review, ideally using a flow diagram.	Supplementary Information section V
	16b	Cite studies that might appear to meet the inclusion criteria, but which were excluded, and explain why they were excluded.	N/A
Study characteristics	17	Cite each included study and present its characteristics.	Supplementary Information Tables S10-11

Risk of bias in studies	18	Present assessments of risk of bias for each included study.	Supplementary Information Table S12
Results of individual studies	19	For all outcomes, present, for each study: (a) summary statistics for each group (where appropriate) and (b) an effect estimate and its precision (e.g. confidence/credible interval), ideally using structured tables or plots.	Figures 1-5; Table 2; Supplementary Information Table S11
Results of syntheses	20a	For each synthesis, briefly summarise the characteristics and risk of bias among contributing studies.	Results; Table 2; Supplementary Information Table S12
	20b	Present results of all statistical syntheses conducted. If meta-analysis was done, present for each the summary estimate and its precision (e.g. confidence/credible interval) and measures of statistical heterogeneity. If comparing groups, describe the direction of the effect.	Results; Figures 1-5; Table 2; Supplementary Information sections IX, XI and XII
	20c	Present results of all investigations of possible causes of heterogeneity among study results.	Results; Table 2; Supplementary Information Table S14
	20d	Present results of all sensitivity analyses conducted to assess the robustness of the synthesized results.	Supplementary Information section XII
Reporting biases	21	Present assessments of risk of bias due to missing results (arising from reporting biases) for each synthesis assessed.	Results; Supplementary Information section X
Certainty of evidence	22	Present assessments of certainty (or confidence) in the body of evidence for each outcome assessed.	95% uncertainty intervals are given alongside all mean relative risk estimates; all relative risk curve figures include shading to depict uncertainty intervals (both

			with and without between-study heterogeneity)
**DISCUSSION**			
Discussion	23a	Provide a general interpretation of the results in the context of other evidence.	Table 1; Discussion (paragraphs 2-4)
	23b	Discuss any limitations of the evidence included in the review.	Table 1; Discussion (paragraph 10)
	23c	Discuss any limitations of the review processes used.	Table 1; Discussion (paragraph 10)
	23d	Discuss implications of the results for practice, policy, and future research.	Table 1; Discussion (paragraphs 6-9)
**OTHER INFORMATION**			
Registration and protocol	24a	Provide registration information for the review, including register name and registration number, or state that the review was not registered.	Methods ("Overview")
	24b	Indicate where the review protocol can be accessed, or state that a protocol was not prepared.	Methods ("Overview")
	24c	Describe and explain any amendments to information provided at registration or in the protocol.	N/A
Support	25	Describe sources of financial or non-financial support for the review, and the role of the funders or sponsors in the review.	Acknowledgements statement
Competing interests	26	Declare any competing interests of review authors.	Competing interests statement
Availability of data, code and other materials	27	Report which of the following are publicly available and where they can be found: template data collection	Data availability and code availability statements

		forms; data extracted from included studies; data used for all analyses; analytic code; any other materials used in the review.	

XIV. GATHER Checklist

Table S18. GATHER checklist

Item #	Checklist item	Reported on page #
**Objectives and funding**		
**1**	Define the indicator(s), populations (including age, sex, and geographic entities), and time period(s) for which estimates were made.	Main (paragraph 4)
**2**	List the funding sources for the work.	Acknowledgements statement
**Data Inputs**		
For all data inputs from multiple sources that are synthesized as part of the study:		
**3**	Describe how the data were identified and how the data were accessed.	Methods ("The Pragmatic Systematic Review"); Supplementary Information section V
**4**	Specify the inclusion and exclusion criteria. Identify all ad-hoc exclusions.	Methods ("The Pragmatic Systematic Review"); Supplementary Information sections III and V
**5**	Provide information on all included data sources and their main characteristics. For each data source used, report reference information or contact name/institution, population represented, data collection method, year(s) of data collection, sex and age range, diagnostic criteria or measurement method, and sample size, as relevant.	Supplementary Information Table S10-12 and Burden of Proof VizHub https://vizhub.healthdata.org/burden-of-proof/
**6**	Identify and describe any categories of input data that have potentially important biases (e.g., based on characteristics listed in item 5).	Methods ("Testing and adjusting for biases across study designs and characteristics"); Supplementary Information Table S12
For data inputs that contribute to the analysis but were not synthesized as part of the study:		
**7**	Describe and give sources for any other data inputs.	N/A

For all data inputs:		
**8**	Provide all data inputs in a file format from which data can be efficiently extracted (e.g., a spreadsheet rather than a PDF), including all relevant meta-data listed in item 5. For any data inputs that cannot be shared because of ethical or legal reasons, such as third-party ownership, provide a contact name or the name of the institution that retains the right to the data.	Data inputs can be found in the Burden of Proof VizHub https://vizhub.healthdata.org/burden-of-proof/
**Data analysis**		
**9**	Provide a conceptual overview of the data analysis method. A diagram may be helpful.	Methods ("Overview")
**10**	Provide a detailed description of all steps of the analysis, including mathematical formulae. This description should cover, as relevant, data cleaning, data pre-processing, data adjustments and weighting of data sources, and mathematical or statistical model(s).	Methods ("Overview", "Estimating the shape of the exposure-relative risk relationship", "Testing and adjusting for biases across study designs and characteristics", "Quantifying between-study heterogeneity", and "Estimating the burden of proof risk function")
**11**	Describe how candidate models were evaluated and how the final model(s) were selected.	Methods ("Estimating the shape of the exposure-relative risk relationship"); Supplementary Information section XII
**12**	Provide the results of an evaluation of model performance, if done, as well as the results of any relevant sensitivity analysis.	Supplementary Information section XII
**13**	Describe methods for calculating uncertainty of the estimates. State which sources of uncertainty were, and were not, accounted for in the uncertainty analysis.	Methods ("Overview" and "Quantifying between-study heterogeneity")
**14**	State how analytic or statistical source code used to generate estimates can be accessed.	All code used for these analyses is publicly available online (https://github.com/ihmeuw-msca/burden-of-proof/tree/main)

Results and Discussion		
**15**	Provide published estimates in a file format from which data can be efficiently extracted.	Available via the Burden of Proof VizHub https://vizhub.healthdata.org/burden-of-proof/
**16**	Report a quantitative measure of the uncertainty of the estimates (e.g. uncertainty intervals).	95% uncertainty intervals are given for all findings, including in the text, figures, and tables in the main text and Supplementary Information
**17**	Interpret results in light of existing evidence. If updating a previous set of estimates, describe the reasons for changes in estimates.	Table 1; Discussion (paragraphs 2-4)
**18**	Discuss limitations of the estimates. Include a discussion of any modelling assumptions or data limitations that affect interpretation of the estimates.	Table 1; Discussion (paragraph 10)
